# Probiotics synergized with conventional regimen in managing Parkinson’s disease

**DOI:** 10.1038/s41531-022-00327-6

**Published:** 2022-05-24

**Authors:** Hairong Sun, Feiyan Zhao, Yuanyuan Liu, Teng Ma, Hao Jin, Keyu Quan, Bing Leng, Junwu Zhao, Xiaoling Yuan, Zhenguang Li, Fang Li, Lai-Yu Kwok, Shukun Zhang, Zhihong Sun, Jinbiao Zhang, Heping Zhang

**Affiliations:** 1 Inner Mongolia Key Laboratory of Dairy Biotechnology and Engineering; Key Laboratory of Dairy Products Processing, Ministry of Agriculture and Rural Affairs; Key Laboratory of Dairy Biotechnology and Engineering, Ministry of Education, Inner Mongolia Agricultural University, Hohhot, Inner Mongolia 010018 China; 2 Department of neurology, Weihai Municipal Hospital, Cheeloo College of Medicine, Shandong University, Weihai, Shandong 264200 China; 3 Department of Neurology, Liaocheng People’s Hospital and Liaocheng Clinical School of Taishan Medical University, Liaocheng, Shandong 264200 China; 4 Department of Neurology, The First Affiliated Hospital of Jinzhou Medical University, Jinzhou, Liaoning 121000 China; 5 Department of Pathology, Weihai Municipal Hospital, Cheeloo College of Medicine, Shandong University, Weihai, Shandong 264200 China

**Keywords:** Parkinson's disease, Parkinson's disease

## Abstract

Parkinson’s disease (PD) is mainly managed by pharmacological therapy (e.g., Benserazide and dopamine agonists). However, prolonged use of these drugs would gradually diminish their dopaminergic effect. Gut dysbiosis was observed in some patients with PD, suggesting close association between the gut microbiome and PD. Probiotics modulate the host’s gut microbiota beneficially. A 3-month randomized, double-blind, placebo-controlled clinical trial was conducted to investigate the beneficial effect of probiotic co-administration in patients with PD. Eighty-two PD patients were recruited and randomly divided into probiotic [*n* = 48; *Bifidobacterium animalis* subsp. *lactis* Probio-M8 (Probio-M8), Benserazide, dopamine agonists] and placebo (*n* = 34; placebo, Benserazide, dopamine agonists) groups. Finally, 45 and 29 patients from Probio-M8 and placebo groups provided complete fecal and serum samples for further omics analysis, respectively. The results showed that Probio-M8 co-administration conferred added benefits by improving sleep quality, alleviating anxiety, and gastrointestinal symptoms. Metagenomic analysis showed that, after the intervention, there were significantly more species-level genome bins (SGBs) of *Bifidobacterium animalis*, *Ruminococcaceae*, and *Lachnospira*, while less *Lactobacillus fermentum* and *Klebsiella oxytoca* in Probio-M8 group (*P* < 0.05). Interestingly, *Lactobacillus fermentum* correlated positively with the scores of UPDRS-III, HAMA, HAMD-17, and negatively with MMSE. *Klebsiella oxytoca* correlated negatively with feces hardness. Moreover, co-administering Probio-M8 increased SGBs involved in tryptophan degradation, gamma-aminobutyric acid, short-chain fatty acids, and secondary bile acid biosynthesis, as well as serum acetic acid and dopamine levels (*P* < 0.05). Taken together, Probio-M8 synergized with the conventional regimen and strengthened the clinical efficacy in managing PD, accompanied by modifications of the host’s gut microbiome, gut microbial metabolic potential, and serum metabolites.

## Introduction

Parkinson’s disease (PD) is a neurodegenerative disease that afflicts seven to ten million people all over the world^1^. It is considered a multifactorial disease that is resulted from both genetic and environmental reasons. Patients with PD are characterized by classic motor symptoms, such as resting tremor, bradykinesia, and rigidity, as well as gastrointestinal (GI) symptoms, including constipation, slower colonic transit time, and small intestinal bacterial overgrowth^2^. Notably, the GI symptoms of PD patients often precede the motor signs, suggesting an association between gut abnormalities and the onset of PD^3^.

The gut has been proposed as the second brain of humans, harboring a dynamic gut microbiome that possesses microbial genomes over 100 times larger than that of the human genome^4^. Alterations in the gut microbiota and metabolome have often been observed in patients with PD. For example, the proportion of opportunistic pathogens, such as *Anaerococcus*, *Campylobacter*, and *Lactobacillus*, was found to be elevated, while the polymicrobial cluster of *Blautia*, *Butyricicoccus, Lachnospira*, and other short-chain fatty acids (SCFA)-producing bacteria was diminished in patients with PD^5,6^. Moreover, the abundance of some gut microbes (e.g., *Lachnospiraceae* and *Enterobacteriaceae*) was reported to be significantly associated with the severity of PD and its associated symptoms^7^.

The “gut-brain-axis” is a relatively new concept, proposing the existence of bidirectional communication pathways between the intestine and the brain; and misregulation of the communications and interactions between the two body compartments might be related to neurodegenerative diseases including PD^8^. Sampson et al.^9^ found that alpha-synuclein, the most important substance in PD pathology, could be transmitted from the gut to the brain through the vagus nerve causing dyskinesia, confirming an active role of the gut in driving the development and pathogenesis of PD^10^. Mounting evidence suggests that the gut microbiome has the ability to synthesize and/or regulate various neurochemicals and neurometabolites that may directly or indirectly impact the physiological process of the organism, as well as the onset and development of PD^11^. Marcus et al. (2021) showed that transplanting microbiota from young donors to aging recipient mice reversed aging-associated impairments in cognitive and behavioral deficits, partly via modulating the production of SCFAs and neurotransmitters^12^. Additionally, levodopa (the precursor of dopamine), gamma-aminobutyric acid (GABA), and 5-hydroxytryptamine (5-HT) could be produced by certain microbes in the gut and transmitted to the brain through the systemic circulation, finally relieving neurodegenerative diseases^13–15^.

Currently, the main management for PD is administering anti-Parkinson medications, such as Levodopa, Benserazide, Sirelin, and Pramipexole. Pharmacological therapy can improve PD-associated symptoms, but the dopaminergic treatment effect would gradually diminish and larger doses of medication would be needed eventually^16–18^. Therefore, many alternative and/or adjuvant therapies (such as exercise training^17^ and acupuncture^18^) have been developed and applied in the management of PD. Owing to the existence of the gut-brain-axis and the close link between gut dysbiosis and pathology of PD, target modulation of the gut microbiome might be an interesting alternative/adjuvant management approach that could alleviate PD.

Probiotics are “live microorganisms that confer a benefit on the host when administered in adequate amounts”^19^. A previous report showed that daily administration of complex probiotics [including *Bifidobacterium* (*B*.) *bifidum*, *B. longum*, *Lactobacillus* (*L*.) *rhamnosus* GG] for 16 weeks conferred protective effects on dopamine neurons and significantly improved motor dyskinesia (e.g., gait pattern and body balance) in a PD mouse model^20^. An engineered glucagon-like peptide-1-producing probiotic strain could mitigate neuroinflammation in PD model mice, evidenced by the alleviation of lipopolysaccharide-induced memory impairment and 1-methyl-4-phenyl-1,2,3,6-tetrahydropyridine (MPTP)-related dyskinesia^21^. Besides murine models, several double-blind randomized placebo-controlled trials (RCTs) have shown that probiotics conferred beneficial effects to patients with PD^22–27^, including the relief of GI symptoms (including abdominal pain, abdominal distension, nausea, constipation, and spontaneous defecation). On the other hand, investigations of the effects of probiotics on PD-related motor and non-motor symptoms, as well as inflammation factors, have yielded inconsistent results. One study showed that administering complex probiotics (containing *L. acidophilus*, *B. bifidum*, and *L. fermentum*) improved the Movement Disorders Society-Unified Parkinson’s Disease Rating Scale (MDS-UPDRS) scores, blood glutathione, high-sensitivity C-reactive protein, malondialdehyde, and insulin of patients with PD^24^. However, other studies found that the scores of the MDS-UPDRS and Non-Motor Symptom Scale (NMSS), biomarkers of inflammation and oxidative stress(complex probiotics containing *L. acidophilus*, *L. casei*, *B. bifidum*, and *L. fermentum*)^28^, and fecal calprotectin [complex probiotics containing *Enterococcus* (*E*.) *faecium*, *L. acidophilus*, *L. paracasei*, *L. rhamnosus*, *B. longum*, *B. bifidum*, *L. reuteri*, and *E. faecalis*]^25^ were not affected by probiotic administration. These reports serve as supporting evidence of the positive impacts of probiotic intake in patients in PD; however, it is likely that factors such as probiotic strain specificity and host conditions would influence the final clinical outcomes. Thus, it would be necessary to perform individual trials to confirm the health-promoting effects of specific probiotics in each case. Moreover, since conventional medications for managing PD may cause significant side effects, it would be of interest to explore the clinical effect of adjuvant probiotic therapy and to find a new candidate strain that synergizes with conventional therapeutics for the management of PD. Few studies have addressed such aspects.

*Bifidobacterium animalis* subsp. *lactis* Probio-M8 (Probio-M8) is a novel probiotic strain that was previously isolated from the breast milk of a healthy woman. The administration of Probio-M8 could alleviate cognitive impairment in APP/PS1 transgenic mice, an animal model of Alzheimer’s disease^29,30^, and it has also been used as one of the strains in a complex probiotic formulation, which relieved anxiety and stress state of long-term sailors^31^. Based on these reports, we hypothesized that Probio-M8 was a potential candidate psychobiotic for alleviating neurodegenerative diseases and other neurological conditions. This study aimed to assess the added beneficial effect and mechanism of administering Probio-M8 as adjuvant treatment when given to patients with PD together with a conventional regimen (Benserazide and dopamine agonists) in a double-blind RCT. The clinical outcomes were evaluated by changes in a number of clinical indices, gut microbiome, and serum metabolome of the participants during/after the course of intervention.

## Results

### Co-administrating Probio-M8 alleviated PD-associated symptoms

**Fig. 1:**
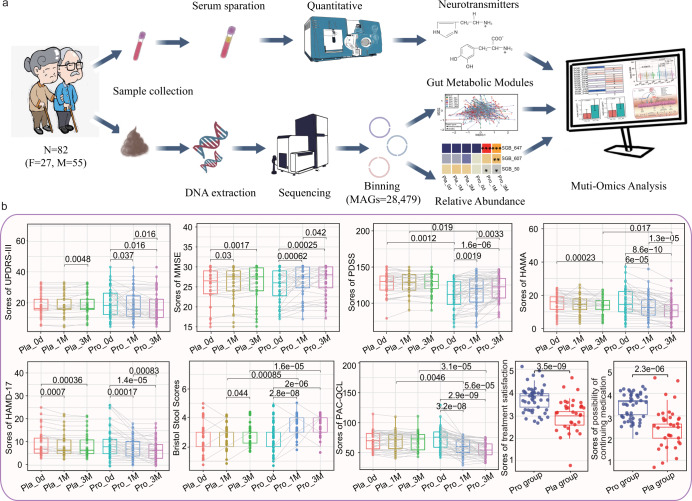
Experimental design, data analysis, and clinical indexes of Parkinson’s disease. **a** The workflow of sampling, sequencing and metagenome-assembled genomes (MAGs) binning of gut microbiota and serum neurotransmitters, all elements in this image were the graph of results of this article or created by in iPad. **b** Statistical differences in the clinical parameters within and between groups were evaluated with paired *t*-tests and *t*-tests, respectively. Significant *p* values between sample pairs are shown. Boxplot elements: center line, median; box limits, upper and lower quartiles; whiskers, 1.5x interquartile range; points, outliers. Each dot represents a data point of a participant, and data of the same participant at different time points are connected by straight lines. “Pro” and “Pla” represent the Probio-M8 group and Placebo group, respectively. “0d”, “1 M”, and “3 M” represent the baseline before the intervention, 1 and 3 months after intervention, respectively. UPDRS-III, MMSE, PDSS, HAMA, HAMD-17, PAC-QOL represent Unified PD Rating Scale-III, Mini-mental State Examination, Parkinson’s Disease Sleep Scale, Hamilton Anxiety Scale, Hamilton Depression Scale-17, Patient-Assessment of Constipation Quality of Life, respectively.

On day 0, no significant difference was observed in all the monitored parameters between Probio-M8 and placebo groups except the scores of PDSS. The sleep quality of patients in the Probio-M8 group was not as good as those in the placebo group. During/after the course of the intervention, some of the monitored parameters showed significant improvements for both groups (e.g., decrease in UPDRS-III scores, increase in MMSE scores, decrease in HAMA scores, decrease in HAMD-17 scores, and so on; Fig. 1b). Notably, for some of the parameters, a larger magnitude of improvement was observed in the M8 group than the placebo group, e.g., the decrease in HAMA scores (Probio-M8 group: 15.65 at baseline to 11.17 at 3 M, *P* = 8.6e-10; placebo group: 14.91 at baseline to 13.85, *P* < 0.001) and the increase in PDSS scores (Probio-M8 group: 111.08 at baseline to 121.02 at 3 M, *P* < 0.001; placebo group: 126.41 at baseline to 127.85, *P* = 0.095). Interestingly, improvement in GI-related symptoms was mostly observed in the Probio-M8 group. Marked increases were observed in Bristol scores in Probio-M8 group (2.73 at baseline to3.40 at 1 M, *P* < 0.001; 3.58 at 3 M, *P* < 0.001), though placebo group also showed significant increase from 2.79 at 1 M to 2.91 at 3 M (*P* = 0.044). Other GI-related parameters, including the times of spontaneous defecations and completed defecation per week, feces hardness, and difficulty in defecation, also improved in greater magnitudes in the Probio-M8 group (Supplementary Table 5). Moreover, the scores of PAC-QOL at 1 M and 3 M significantly decreased only in the Probio-M8 group (*P* < 0.001 and *P* < 0.001, respectively) but not the placebo group. These results suggested that, compared with a conventional regimen, adjuvant Probio-M8 treatment conferred added clinical effects on improving anxiety, sleep quality, and GI symptoms of PD patients (Fig. 1b and Supplementary Table 5).

The UPDRS-III scores, reflecting the severity of PD were significantly reduced in the Probio-M8 group at 1 M and 3 M versus baseline (*P* = 0.037, *P* = 0.016, respectively), while the scores only dropped at 3 M (*P* = 0.037) but not earlier in the placebo group. Besides, the magnitudes of improvement in sleep quality, anxiety state, mental state (measured by MMSE), and depression (measured by HAMD-17) of patients were greater in the Probio-M8 group than in the placebo group (Fig. 1b). The probio-M8 group also showed significantly higher scores for the satisfaction of treatment and the possibility of continuing medication than the placebo group (*P* < 0.001 in both cases, Fig. 1b).

Altogether, changes in the clinical parameters and symptom-related scores in the patients during/after the intervention reflected that co-supplementation of the probiotic strain, Probio-M8, offered added beneficial effects, particularly in stress-/sleep-related issues and constipation-associated issues, when taken as an adjunct to conventional drugs.

### Probio-M8 modified the key gut bacteria in patients with PD

**Fig. 2:**
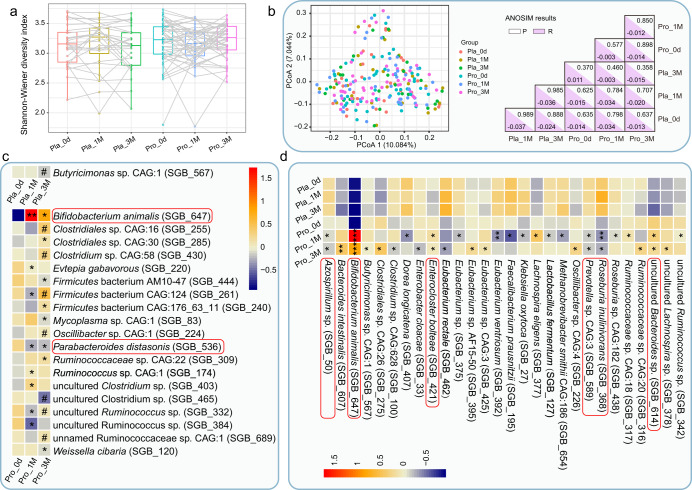
Microbial diversity and differentially abundant species-level genome bins (SGBs) between groups. **a** Shannon diversity index of the fecal microbiome at 0d, 1 M, and 3 M in placebo and Probio-M8 groups, respectively. Statistical differences in the Shannon diversity index within groups were evaluated with paired *t*-tests. Each dot represents a data point of a participant, and data of the same participant at different time points are connected by straight lines. Boxplot elements: center line, median; box limits, upper and lower quartiles; whiskers, 1.5x interquartile range; points, outliers. **b** Left panel, principal coordinates analysis (PCoA) score plots of two groups at different time points. Samples of each subgroup are represented by a different color. Right panel, *P* value and R value of analysis of similarities (ANOSIM, 999 permutations). **c** Longitudinal comparisons of an abundance of SGBs of the same group between different time points. All the shown SGBs were not significantly different between groups at baseline but only increased/decreased significantly during the trial. Significant differences in specific SGBs between 0d and other time points are represented by *P < 0.05 and **P < 0.01; between 1 M and 3 M are represented by ^#^*P* < 0.05. **d** Horizontal comparisons between an abundance of SGBs of Probio-M8 and placebo groups at the same time point. All the shown SGBs were not significantly different between groups at baseline but became significantly differential abundant during/after the intervention. Significant differences: *P < 0.05, **P < 0.01, and ***P < 0.001. The red corner rectangles in (**c**) and (**d**) represents an abundance of SGBs on an arbitrary scale. “Pro” and “Pla” represent the Probio-M8 group and Placebo group, respectively. “0d”, “1 M”, and “3 M” represent the baseline before intervention, 1 and 3 months after intervention, respectively.

The Shannon diversity index has been used to evaluate the richness and diversity of fecal microbiota. No significant change was observed in the Shannon diversity index in both longitudinal (different time points of the same group) and horizontal (same time point between different groups) comparisons (Fig. 2a). Meanwhile, the PCoA (Bray-Curtis distance) score plot did not show any time-based clustering patterns (*P* > 0.05, Fig. 2b). These results suggested that the drug treatment with or without probiotic supplementation did not cause drastic changes in subjects’ gut microbiota diversity and structure. In addition, no significant difference was found in age-based comparisons of the gut microbiota structure (adult: 45 to 59 years old; younger elderly: 60 to 74 years old; elderly: 75–87 years old; ANOSIM test, *P* = 0.414, *R* = 0.006; Supplementary Fig. 2).

However, probiotic-driven modulations of fecal microbiota were revealed at the compositional level by a finer taxonomic analysis. The abundance of *B. animalis* (SGB_647) was at a very low level across all samples at day 0 (0.15; detected only in two patients in the placebo group and one patient in the Probio-M8 group). Throughout the intervention period, the abundance of *B. animalis* remained low in placebo group (0.059 at 1 M and 0.044 at 3 M, only detected in the two aforementioned patients) but increased significantly in the Probio-M8 group over time (4.57 at 1 M, *P* = 0.003; 2.52 at 3 M, *P* = 0.048 versus baseline level of Probio-M8 group). Horizontal comparisons between intervention groups at the same time points also showed consistent and significant increases in the abundance of *B. animalis* in the Probio-M8 group (*P* = 2.8e-08 and *P* = 1.1e-05, at 1 M and 3 M, respectively). Although the standard sequencing depth employed in this study (5.08 ± 1.11 Gbp per sample) like most metagenomic sequencing studies of similar nature was not adequate for strain-level tracking of a target microbe in fecal metagenomic datasets, all participants were requested to refrain from taking major food sources of *B. animalis*, such as yogurt, fermented milk drinks, probiotic preparations, etc. Thus, the significant increases in SGBs of *B. animalis* were likely a reflection of amounts of the provided strain (Probio-M8) in patients’ guts. Meanwhile, these results confirmed the participant's adherence during the trial.

A total of 48 responsive SGBs were identified, which did not show significant differential abundance between Probio-M8 and placebo groups at baseline but only became differentially abundant during/after the course of intervention (Fig. 2c, d and Supplementary Table 6). Longitudinal comparisons of SGBs of the same group at different time points revealed a significant decrease in only one SGB_567 (representing *Butyricimonas* sp. CAG:1) in control group at 3 M, while another 19 SGBs showed significant changes in abundance in Probio-M8 group in later time points (Fig. 2c), such as the abundance of *Parabacteroides* (*P*.) *distasonis* (SGB_536), *Mycoplasma* sp. CAG:1 (SGB_83), and *Evtepia gabavorous* (SGB_220) decreased in probiotic group at 1 M and 3 M (Fig. 2c). Horizontal comparisons between SGBs of Probio-M8 and placebo groups at the same time point revealed 28 significantly differential abundant SGBs (Fig. 2d). The levels of SGBs representing *Klebsiella* (*K*.) *oxytoca* (SGB_27) and *L. fermentum* (SGB_127) were diminished. In contrast, the levels of SGBs representing *Ruminococcaceae* [*Ruminococcaceae* sp. CAG:18 (SGB_317), *Ruminococcaceae* sp. CAG:20 (SGB_316), uncultured *Ruminococcus* sp. (SGB_342)], *Lachnospira* [*Lachnospira eligens* (SGB_377), uncultured *Lachnospira* sp. (SGB_378)], and *Butyricimonas* [*Butyricimonas* sp. CAG:1(SGB_567)] increased in Probio-M8 group (Fig. 2d). Our results suggested that co-supplementation of Probio-M8 modulated the patients’ gut microbiota desirably by suppressing some potentially pathogenic taxa while increasing the beneficial ones.

### Multivariable association between clinical metadata and gut microbiota of patients with PD

**Fig. 3:**
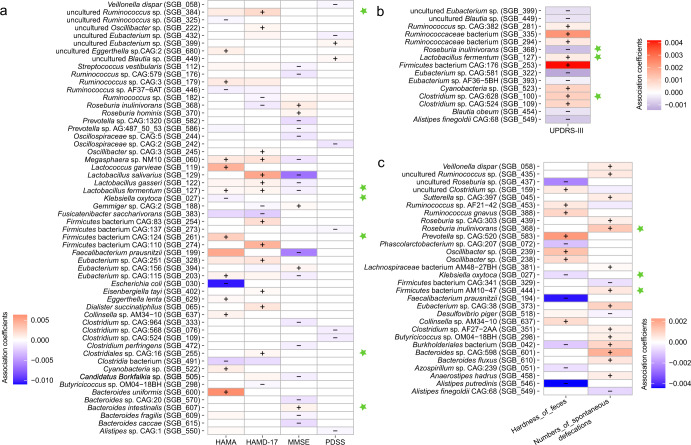
Association between fecal species-level genome bins (SGBs) of patients with Parkinson’s disease (PD) and clinical indicators of Parksoion-associated symptoms. Association between gut microbiota and **a** mental state (represented by Hamilton Anxiety Scale, Hamilton Depression Scale-17, and Mini-mental State Examination) and sleep quality (represented by PDSS) of patients, **b** patient’s disease progression (Unified Parkinson Disease Rating Scale-III), and **c** constipation-related symptoms (feces hardness and numbers of spontaneous defecation). Marker SGBs for PD were marked by a green star. “+” and “−” represent a positive and negative association, respectively.

The multivariable association analysis between gut microbiota and UPDRS, mental metadata (MMSE, PDSS, HAMA, and HMAD), and defecation-related clinical indicators were implemented by MaAsLin2. Our results showed a strong correlation between seven clinical indicators, namely the scores of MMSE, PDSS, HAMA, HAMD, UPDRS (Fig. 3a, b), as well as feces hardness and the number of spontaneous defecation (Fig. 3c), with 90 gut SGBs (the relative abundance >0.2%; *P* < 0.005).

Nine of them were biomarker species responsive specifically to probiotic supplementation, including *Bacteroides* (*B*.) *intestinalis* (SGB_607), *Clostridiales* sp. CAG:16 (SGB_255), *Clostridium* sp. CAG:628 (SGB_100), Firmicutes bacterium AM10 − 47 (SGB_444), Firmicutes bacterium CAG:124 (SGB_261), *K. oxytoca* (SGB_027), *L. fermentum* (SGB_127), *Roseburia inulinivorans* (SGB_368), and uncultured *Ruminococcus* sp (SGB_384). Of particular interest was *L. fermentum* (SGB_127) that was diminished in the Probio-M8 group, and it was found to associate with multiple clinical parameters: significant positive correlation with the scores of UPDRS, HAMA, and HAMD-17; significant negative correlation with the scores of MMSE (Fig. 3a, b). *Klebsiella oxytoca* (SGB_27) was another interesting SGB that correlated negatively with two different clinical indexes, i.e., HAMA scores and feces hardness (Fig. 3a, c).

### Probio-M8 modulated gut microbiota-related neuroactive modules

**Fig. 4:**
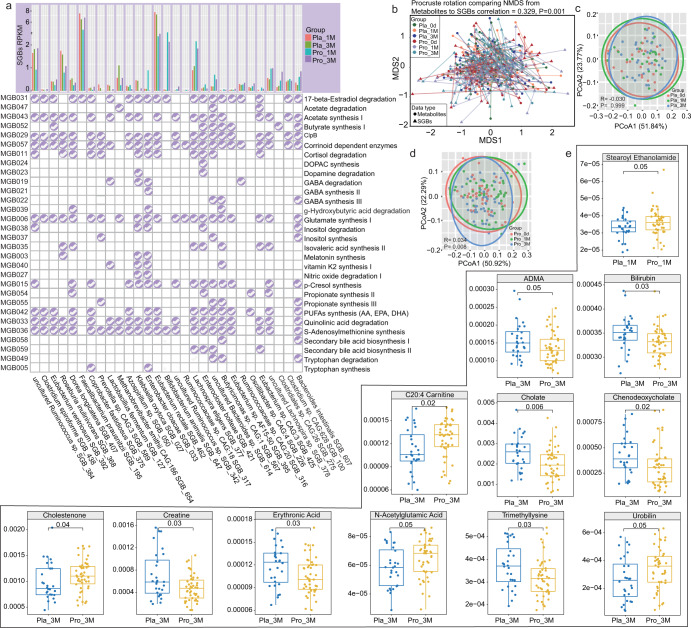
Profiles of gut metabolic modules (GMMs) and predicted metabolites after 1- and 3-month intervention. **a** Distribution of selected GMMs (relating to the development, pathophysiology, and immunity related to Parkinson’s disease) across significant differential species-level genome bins (SGBs) between probio-M8 (Pro) and placebo (Pla) groups. **b** Procrustes analyses performed on the predicted microbiomes and metabolomes of the probio-M8 (Pro) and placebo (Pla) groups at day 0 (0d), 1 month (1 M), and 3 months (3 M), showing positive cooperativity between the microbiome and metabolome profiles (correlation = 0.329; *P* = 0.001). **c**, **d** Principal coordinates analysis (PCoA) score plots and Adonis test of the Probio-M8 (Pro) and placebo (Pla) groups at days 0 (0d), 1 month (1 M), and 3 months (3 M) showing changes in predicted bioactive metabolites. Symbols representing samples of individuals at different time points are shown in different colors. Significant differences between groups were evaluated by the adonis test. **e** Boxplots showing the contents of predicted differential bioactive metabolites that were responsive to Probio-M8 adjuvant treatment, *P* < 0.05 was considered statistically significant. Boxplot elements: center line, median; box limits, upper and lower quartiles; whiskers, 1.5x interquartile range; points, outliers.

Our study profiled the metabolic modules of neuroactive compounds encoded by the gut microbiota according to the methods described previously by Valles-Colomer^32^. Forty-eight modules were identified, Corrinoid dependent enzymes, *S*-Adenosylmethionine (SAM) synthesis, Glutamate synthesis II, Acetate synthesis I, and Glutamate synthesis I were the five metabolic modules having the most coverage across the gut microbiota dataset (Supplementary Table 7). To pinpoint the Probio-M8-specific effect on the gut microbiota-related neuroactive compounds, metabolic modules among differential abundant SGBs between probiotic and placebo groups were identified. Thirty metabolic modules could be identified from the 29 probiotic responsive SGBs (Fig. 4a). Intake of Probio-M8 increased the diversity of SGBs encoding modules participating in tryptophan degradation, GABA, SCFAs (isovalerate, butyrate, and propionate), and secondary bile acid biosynthesis; meanwhile, the placebo group had more diverse SGBs encoding modules participating in vitamin K2 synthesis, tryptophan synthesis, and inositol degradation. Moreover, after three months of intervention, more patients in the probiotic group had PUFAs-synthesizing SGBs. Interestingly, compared with the probiotic receivers, the patients in placebo groups had fewer SGBs involved in GABA synthesis, but more SGBs encoding GABA degradation (Fig. 4a).

### Probio-M8 enhanced the production of bioactive metabolites by gut microbiota

Gut microbiota-originated metabolites were predicted by MelonnPan. A total of 80 metabolites were identified, and deoxycholic acid and glutamate were the most abundant metabolites. Procrustes analysis is a statistical method that displays multi-omics datasets in low-dimensional space after data dimensionality reduction, and it has been increasingly used in microbiome and metabolomics research to evaluate the cohesiveness between datasets^33^. The concordance between the gut microbiome and predicted metabolome was evaluated by Procrustes analysis, and positive cooperativity was found between the two datasets (correlation = 0.329, *P* = 0.001, Fig. 4b). Changes in patients’ gut metabolome were assessed by PCoA analysis, and no specific time-based clustering pattern was observed on the PCoA score plots of both groups (Fig. 4c, d). However, significant difference was found in the gut microbiota community between time points in Probio-M8 (baseline versus 1 M, *P* = 0.233, R = 0.006; baseline versus 3 M, *P* = 0.003, R = 0.075; adonis test; Fig. 4d) but not placebo group (Fig. 4c).

To pinpoint the spectrum of probiotic-regulated gut bioactive metabolites, the differential predicted metabolites between the Probio-M8 group and placebo group at 1 M and 3 M were identified. Only one differential predicted metabolite was identified after one month of treatment, which was stearoyl ethanolamide; significantly more stearoyl ethanolamide (*P* = 0.05) was found in the probiotic group than placebo group at 1 M. More obvious changes were detected at 3 M, which were observed in 11 predicted metabolites. At 3 M, the predicted abundances of asymmetrical dimethylarginine (ADMA), bilirubin, cholate, chenodeoxycholate, creatine, erythronic acid, and trimethyllysine (*P* ≤ 0.05 in all cases, Fig. 4e and Supplementary Table 8) dropped significantly in Probio-M8 group compared with the placebo group, and opposite trends were observed in C20:4 carnitine, cholestenone, *N*-acetylglutamic acid, and urobilin.

### Probio-M8 affected serum SCFAs and neurotransmitters in patients with PD

To confirm the results generated by MelonnPan and Procrustes analyses, GC-MS/MS was used to determine the levels of serum SCFAs and neurotransmitters before and during the course of intervention. However, due to the cost of analysis, only 26 serum samples (13 random serum samples in each group) were analyzed.

**Fig. 5:**
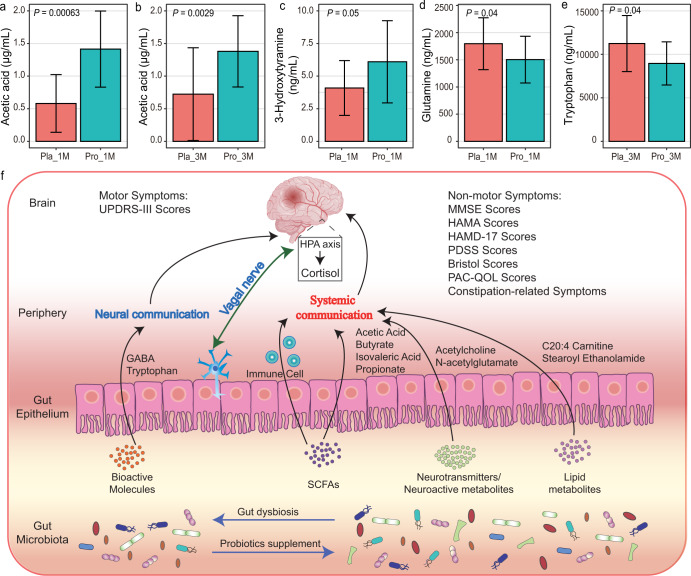
Differences in serum short-chain fatty acids (SCFAs) and neuroactive compounds between Probio-M8 (Pro) and placebo (pla) groups and proposed model of probiotic-driven pathways modulating the gut-brain-axis in patients with Parkinson’s disease. **a**–**e** Significant differences in serum SCFAs and neuroactive compounds between the probiotic and placebo groups at 1 month (1 M) and 3 months (3 M), error bars represented SD; *P* < 0.05 was considered statistically significant. **f** Schematic diagram illustrating key probiotic-driven pathways that modulated the gut-brain-axis and host response. UPDRS-III, MMSE, HAMA, HAMD-17, PDSS, and PAC-QOL represent Unified PD Rating Scale-III, Mini-mental State Examination, Hamilton Anxiety Scale, Hamilton Depression Scale-17, Parkinson’s Disease Sleep Scale, Patient-Assessment of Constipation Quality of Life, respectively. GABA and SCFAs represent gamma-aminobutyric acid and short-chain fatty acids.

Seven kinds of SCFAs (including acetic acid, propionic acid, isobutyric acid, butyric acid, isovaleric acid, valeric acid, and heptanoic acid) and 12 types of neurotransmitters (including acetylcholine, choline, glutamic acid, phenylalanine, kynurenine, tyrosine, histidine, arginine, serotonin, tryptophan, 3-Hydroxytyramine, and glutamine) were detected across samples. The serum concentration of acetic acid was significantly higher in the patients of the probiotic group at 1 M and 3 M (*P* < 0.05, Fig. 5a, b and Supplementary Table 9). Moreover, significantly more dopamine (3-hydroxytyramine) was detected in the serum samples of patients of the probiotic group compared with the placebo group at 1 M (*P* = 0.05, Fig. 5c, Supplementary Table 9), while the serum concentrations of glutamine and tryptophan (Fig. 5d, e, respectively; Supplementary Table 9) were significantly lower in the probiotic group compared with the placebo group at 1 M. Unfortunately, no significant difference was detected in another 14 neurotransmitters between groups and time points. These results confirmed that adjuvant Probio-M8 therapy regulated the serum profile of SCFAs and neurotransmitters in patients with PD.

### Adverse events

No additional adverse event of any level of severity was noted in the Probio-M8 group compared with the placebo group during the trial period, demonstrating the clinical safety of Probio-M8 co-administration with conventional medication for PD.

## Discussion

The main finding of this double-blind RCT was that Probio-M8 significantly alleviated the severity of PD, improved PD-associated problems (e.g., poor sleep quality, mental state, and defecation), and enhanced the quality of life of patients with PD. The disease improvement was accompanied by changes in the patients’ gut microbiome driven by probiotic application. Probiotic-driven improvement of PD-associated symptoms, such as relief of abdominal pain and bloating, decrease in Bristol scores, increase in the number of spontaneous bowel movements per week, and increases in hs-CRP, Malondialdehyde, and decrease in glutathione and insulin metabolism, have previously been reported^21–27^; however, to the best of our knowledge, the current study was the first report that investigated the effect of probiotic adjuvant treatment on non-motor symptoms in patients with PD. This study also aimed to investigate the probiotic mechanisms from the perspective of the host’s gut microbiota and serum metabolome.

Our study showed that the administration of Parkinson’s drugs (Benserazide and dopamine agonists) along with probiotics significantly improved patients’ UPDRS-III scores. Similar observations were reported in previous works^24,34^. One possible mechanism of probiotic-driven alleviation of PD-related symptoms was the increase in activity of tyrosine hydroxylase, which is a key factor for L-tyrosine-L-dopa transformation^35^. Apart from motor symptoms, constipation is prevalent in patients with PD, and it might have occurred 15–24 years before the diagnosis of PD^36^. Our literature search identified only six studies that investigated the beneficial effect of probiotic administration/ probiotic fermented milk on patients with PD^23–28^, and four of which indicated that probiotic consumption increased Bristol scores and improved defecation habits^23,25–27^. As expected, our data showed that administering Probio-M8 also improved stool consistency, the number of spontaneous and completed defecation per week, parameters of difficulty in defecation, incomplete defecation, assisted defecation by hand, times of drain, and hardening of stool, and the scores of PA-CQL.

In addition to constipation-related symptoms, non-motor symptoms, such as depression, anxiety, sleep disorders, and cognitive disorders, also cause significant distress to many patients with PD^37^. Surprisingly, co-administering Probio-M8 for 3 months alleviated anxiety and depression, improved sleep state, and reduced cognitive dysfunction in patients with PD. Adjuvant probiotic treatment has been reported to alleviate symptoms of neurological problems/diseases like anxiety^38,39^ and Alzheimer’s disease^40,41^ through gut microbiota modulation. Our previous study has demonstrated that probiotic consumption for 12 weeks could alleviate stress-/anxiety-related symptoms^42^ in stressed adults through probiotic-induced changes in gut microbiota diversity and functional metagenomic potential^43^, suggesting that the gut microbiota and its environment are important in influencing the development and severity of neurological diseases. The application of probiotics in improving the gut microbiome is thus a promising way to manage problems as such.

The diversity and structure of fecal microbiota have not changed significantly during/after the course of intervention. A relatively high gut microbiota diversity is often considered a healthy physiological state, while a lower gut microbiota diversity is generally associated with unhealthy conditions, such as irritable bowel syndrome^44^ and mild cognitive impairment^45^. However, a low stool consistency, which is common in patients with PD, has been reported to be associated with a relatively high species richness^46^. Meanwhile, a previous 2-year trial investigated the gut microbiota of patients with PD and found no significant difference in the Shannon index and beta diversity between different time points^47^, which is consistent with our results. Thus, probiotic-driven regulation of the gut microbiota did not seem to be obvious on the level of microbiota diversity and structure, but on key species responsible for a specific function in the gut. For example, *Bifidobacterium* have been considered as pathogens in patients with PD, as they were found to correlate with the pathology of PD^47^. However, in our study, the relative abundance of *B. animalis* was found to increase significantly after probiotic intervention, accompanied by the remission of clinical symptoms, suggesting that an increase in *B. animalis* might mitigate PD-associated symptoms.

The gut microbiota of patients with PD was reported to have reduced carbohydrate fermentation and butyrate synthesis capacity and increased proteolytic fermentation compared with healthy individuals^6^. Some taxa in the phylum Firmicutes, such as *Butyricimonas* and *Clostridiam* clusters IV and XIV, have been considered as protagonists of carbohydrate fermentation and butyrate production^48,49^. Our study found that some SGBs in Firmicutes (*Butyricimonas* sp. CAG:1, *Lachnospira eligens*, *Clostridiales* sp. CAG:26, etc.) increased after probiotic intake. Moreover, SGBs involved in SCFAs (butyrate, isovaleric acid, and propionate) synthesis increased in the Probio-M8 group after intervention. *Bacteroides* were reported to be GABA-producers, and their relative abundance correlated negatively with the brain signatures associated with depression^15^. Interestingly, our data showed that more GABA synthesis modules-encoding SGBs were identified in the probiotic group; and more *Bacteroides intestinalis* was found in the Probio-M8 group, which was associated with increased MMSE scores as well. Our data also showed that more SGBs representing *Prevotella* and *Lactobacillus* were found in the placebo group after the course of intervention. *Prevotella* were found to increase in patients with PD, and they played important roles in the metabolism of mucus layer glycoprotein, the permeability of the intestinal barrier, and inflammation^50,51^. Some *Lactobacillus* could produce specific enzymes that convert levodopa into dopamine before it reaches the brain, thus decreasing the drug efficacy and increasing the required dose for clinical effectiveness^13,52^. Our results showed that the abundance of *L. fermentum* in the placebo group was significantly associated with a less fit mental state (characterized by higher scores of HAMA and HAMD; lower scores of MMSE) and more serious illness (characterized by higher scores of UPDRS). In addition, some neuroinflammation-associated pathogens decreased after Probio-M8 intervention, such as *P. distasonis, Evtepia gabavorous*, and, *K. oxytoca*. The species, *P. distasonis*, correlated negatively with hippocampal function, while *K. oxytoca* could induce anxiety and colitis, and the population of apoptotic neuron cells in the brain of mice via production of lipopolysaccharide and enterotoxins^53–55^. In our study, *K. oxytoca* was found to be significantly and negatively associated with feces hardness in patients with PD. Therefore, it was likely that the clinical remissive effect of Probio-M8 was related to the regulation of specific gut microbiota in patients with PD.

The alterations in the gut microbiota were accompanied by considerable changes in the gut metabolome. Changes in predicted metabolites in each group were calculated, and significant differences were found in the gut metabolites among the three monitored time points (0d, 1 M, and 3 M; *P* = 0.008) of the probiotic group but not the placebo group (*P* = 0.999), suggesting a more obvious regulatory effect of Probio-M8 on the gut microbial metabolites. It was previously shown that the alterations in lipid metabolism were related to the carnitine shuttle, sphingolipid metabolism, arachidonic acid metabolism, and fatty acid biosynthesis in patients with PD^56^. Our study also observed probiotic-driven regulation of lipid metabolism, evidenced by increases in the fecal levels of predicted levels of stearoyl ethanolamide and C20:4 carnitine, as well as a significant increase in the serum acetic acid level. Stearoyl ethanolamide inhibits the conversion of ceramide into sphingosine, and sphingosine has been reported as a neurodegeneration compound in patients with PD^57^. Moreover, a *Caenorhabditis elegans* model of PD showed that probiotic administration modified host sphingolipid metabolism and inhibited α-synuclein aggregation^58^. It has been reported that patients with PD have less carnitine compared with healthy individuals^59^. *Bifidobacterium* are acetic acid-producers in the gut^60^, and it was possible that the high abundance of *B. animalis* observed during the course of probiotic intervention contributed to the increase in serum acetic acid. Plasma acetic acid concentration was found to be negatively correlated with colonic transit time^61^, which was consistent with our observation of an increased number of spontaneous defecations per week subject to probiotic treatment.

Another group of microbial neuroactive metabolites, namely amino acids and bile acids, also constitutes information flow along the gut-brain-axis^62^. Our data showed that the predicted levels of cholestenone and *N*-acetylglutamic acid increased in the Probio-M8 group, while cholate, chenodeoxycholate, trimethyllysine, and ADMA significantly increased in the placebo group. Cholate and chenodeoxycholate are *N*-acetylglutamate that are the first intermediates in the arginine biosynthetic pathway^63^; ADMA decreases nitric oxide (NO) by inhibiting NO synthase^64^. Faraco et al^65^. showed that administering l-arginine reversed the cognitive dysfunction of mice by promoting NO production. Cholestenone is the oxidation product of cholesterol, which can be produced by bacteria such as *Bacteroides*. Up to now, little is known about the effects of cholestenone on health, except that a few studies showed that cholestenone correlated positively with the rapid growth of newborns^66^. Cholestenone could also be used in treating *Helicobacter pylori-*infected patients^67^, and it might affect the function of the immune system^68^.

Last but not least, we found that the serum level of dopa increased at 1 M in the Probio-M8 group. Dopamine reduction is the main reason for the pathology of PD. Human dopamine is generated from l-phenylalanine (l-Phe), through the conversion into l-tyrosine (Tyr), followed by levodopa (l-dopa), and further converted into dopamine. However, only l-dopa (but not dopamine) could pass through the blood–brain barrier and relieve PD-associated symptoms^35^. The increased level of serum dopa after one month of Probio-M8 treatment might suggest that more l-dopa entered the circulatory system, suggesting Probio-M8 administration potentially increased the utilization of drugs prescribed to patients with PD and in turn enhanced clinical efficacy. However, such inference needs further experimental verification.

Taken together, a 3-month RCT was conducted in this work to investigate the beneficial effect of adjunctive probiotic treatment in patients with PD. In summary, probiotics Probio-M8 ameliorated the severity of PD and improved the anxiety and depression state of the patients. The potential mechanism of symptom alleviation was via Probio-M8-mediated regulation of the gut microbiota of patients, further regulating the host’s metabolism of lipid, SCFAs, and neurotransmitters, and thus increasing patients’ serum level of dopamine (Fig. 5f). Our results offer new options for managing PD.

## Methods

### Ethics approval and consent to participate

This study complied with the ethical requirements of the Declaration of Helsinki and the regulations of the Good Clinical Practice. This study was approved by the Ethical Committee of Weihai Municipal Hospital (project number 201816). The trial was registered in the Chinese Clinical Trial Registry (http://www.chictr.org.cn/; identifier number: ChiCTR1800016977). Informed consent was signed by all PD patients before the trial started.

### Trial design and subject recruitment

A three-month double-blind RCT was performed and the experimental processes were shown in Fig. 1a. All recruited patients received the pharmacological regimen (Benserazide and dopamine agonists, the dosage determined by severity of PD and physical condition of patients, supplementary Table 1) at the beginning of the trial (June 2019 to June 2020) in three hospitals (Weihai Municipal Hospital, Liaocheng People’s Hospital, and The First Affiliated Hospital of Jinzhou Medical University). Criteria for inclusion: (1) male or female were diagnosed according to the Movement Disorder Society Clinical Diagnostic Criteria for Parkinson’s disease in 2015 (MDS-PD Criteria, International Parkinson and Movement Disorder Society)^69^; (2) patients have clear consciousness and can complete the examination, questionnaire, and medical history collection on their own or with the help of their families; (3) patients who fulfilled functional constipation according to Rome IV criteria, including less than three spontaneous bowel movements per week for the past 3 months with symptom duration of at least 6 months^70^; (4) patients who agreed to participate in the study and signed informed consent form. Criteria for exclusion: (1) serious cognitive dysfunction that affected written and verbal expression; (2) severe aphasia or dysarthria; (3) mentally unfit; (4) serious physical diseases, including severe abnormal liver or kidney functions; (5) chronic digestive system diseases or tumors (e.g., digestive tract ulcers, inflammatory enteritis, and severe liver disease); (6) took immunosuppressive agents for a long time or antibiotics within one month prior to this study; (7) declined to participate.

One hundred and thirty-three PD patients (female to male = 50:83; age = 69.41 ± 6.05 years old; time after diagnosis of PD: 4.67 ± 2.28 years) were recruited. Thirty-three patients did not meet the inclusion criteria and were thus excluded. The remaining subjects were randomized into Probio-M8 (receiving both probiotic strains plus conventional drug, *n* = 50; female to male = 32:18) and probiotic (receiving only conventional drug, *n* = 50; female to male = 30:20) groups, respectively. The subjects in the Probio-M8 group took two grams of Probio-M8 powder (3 × 10^10^ CFU/day; maltodextrin as excipient) daily, while the subjects in the placebo group took two grams of placebo powder (maltodextrin only). The probiotic bacteria and placebo materials were prepared as powder of identical appearance and taste and were provided in individually sealed plastic sachets (JinHua YinHe Biological Technology Co., Ltd., Zhejiang, China; prepared under ISO9001 and HALAL standards). Another 18 subjects were further excluded from the study during the course of the intervention, including two in the Probio-M8 group (decline participation, *n* = 1; irregular dietary habits, *n* = 1) and 16 in the placebo group (decline participation, *n* = 1; irregular dietary habits, *n* = 3; took defecation-promoting drugs, *n* = 12). A total of 82subjects completed the trial (Supplementary Fig. 1).

### Samples collection and clinical parameters

The treatments continued for 3 months. Clinical improvement was the primary outcome of this study, while changes in the fecal microbiota and serum metabolites were the secondary outcomes. Patients were assessed by Unified PD Rating Scale-III (UPDRS- III) for the overall condition of PD^71^; Mini-mental State Examination (MMSE), Hamilton Anxiety Scale (HAMA), and Hamilton Depression Scale-17 (HADM-17) for their mental state^72^; Parkinson’s Disease Sleep Scale (PDSS) for sleep quality^73^; Visual Analog Scale (VAS) for the degree of pain^74^; Activities of Daily Living (ADL) for their daily living ability^75^; and Patient-Assessment of Constipation Quality of Life (PAC-QCL) for their GI symptoms^76^ and other constipation-related issues, including Bristol scores, difficulty in defecation, feces hardness, incomplete defecation, assisted defecation by hand, number/week of spontaneous and completed defecation.

Self-administered questionnaires for recording clinical symptoms and GI-/constipation-related issues were filled out at the clinic at three time points [baseline before intervention (0d), 1 month after the intervention started (1 M), and 3 months after the intervention started (3 M)], the satisfaction of treatment and possibility of continuing medication were quantified and investigated at 3 M and serum samples were collected at the same visit to the clinic (Supplementary Table 1). Fecal samples were collected at the same time points at home by the participants with the provided sterile stool samplers and a DNA protection solution was added after sampling (Guangdong Longsee Biomedical Co., Ltd, Guangzhou, China). Participants received brief training on the usage of the sterile stool samplers to ensure minimal contamination during the sampling process. Collected samples were transported to the hospital with ice packs and all samples were stored in a −80 °C refrigerator before sequencing.

### Extraction of DNA and metagenomic sequencing

Metagenomic DNA was extracted from the patients’ stool by QIAamp Fast DNA Stool Mini Kit (Qiagen GmbH, Hilden, Germany) in accordance with the manufacturer’s instruction; and the quality/integrity/purity of extracted metagenomic DNA and DNA concentration were assessed by 1% agarose gel electrophoresis, Nanodrop spectrophotometer, and the Qubit^®^ dsDNA Assay Kit in combination with a Qubit^®^ 2.0 fluorometer (Life Technologies, CA, USA). Metagenomic DNA samples with a DNA concentration >20 ng/μL and an optical density ratio (260 to 280 nm) between 1.8 and 2.0 were used for sequencing. Sequencing libraries were generated by NEBNext^®^ Ultra™ DNA Library Prep Kit for Illumina (New England Biolabs, Inc., USA), and index codes were added to attribute sequences to each sample. The built DNA library preparations were sequenced on an Illumina NovaSeq platform to generate paired-end reads (Tianjin Novogene Technology Co., Ltd., Tianjin, China).

### DNA quality control

A total of 222 samples were sequenced (*n* = 45 and 29 for Probio-M8 and placebo groups, sampling at 0d, 1M, and 3M, respectively), generating 1.14 Tbp of high-quality paired-end reads (5.14 ± 1.13 Gbp per sample) for gut microbiota analysis. Raw metagenomic reads were quality controlled with the KneadData quality control pipeline (http://huttenhower.sph.harvard.edu/kneaddata; v0.7.5), which filtered out low-quality reads (length of reads <60 nt) by Trimmomatic (a flexible trimmer for sequence data generated by Illumina)^77^. Meanwhile, human contaminating reads were removed by Bowtie2 (v2.3.5.1)^78^. A total of 1.13 Tbp of clean data (5.08 ± 1.11 Gbp per sample, Supplementary Table 2) remained in the dataset for downstream analysis after the quality control steps.

### Metagenomic assembly, contig binning, genome dereplication

Reads of each sample were assembled into contigs using MEGAHIT, with an average N50 length of 20.57 Kbp (Supplementary Table 2). Contigs larger than 2000 bp were extracted for binning to obtain metagenome-assembled genomes (MAGs) using MetaBAT2^79^ with default options. Reads were mapped back to the corresponding contigs using BWA-MEM2^80^, and the contig depth was calculated using Samtools^81^ and the jgi_summarize_bam_contig_depths function in MetaBAT2. The completeness and contamination of MAGs were evaluated using CheckM^82^, and MAGs were divided as high-quality (completeness ≥80%, contamination ≤5%), medium-quality (completeness ≥70%, contamination ≤10%), and partial-quality (completeness ≥50%, contamination ≤5%)^83^. Totally 7538 high-quality genomes (Supplementary Table 3) were gained and clustered, then the most representative genomes from each replicate set were selected by dRep to extract 691 species-level genomic bins (SGBs, Supplementary Table 4), using the parameters -pa 0.95 and -sa 0.95^84^.

### Taxonomic annotation and abundance of SGBs

Kraken2 tool and NCBI nonredundant Nucleotide Sequence Database were used to annotate the MAG contigs with default parameters^85^. Prodigal was used to predict putative genes in the contigs^86^. Then the predicted genes were searched against the UniProt Knowledgebase using the blastp function of DIAMON.

The abundance of each SGBs was calculated using a normalized method, CoverM (https://github.com/wwood/CoverM), using the parameter “–min-read-percent-identity 0.95–min-covered-fraction 0.4”. The abundance level was expressed in reads per kilobase million (RPKM). Then, the sample diversity was calculated using two R packages (vegan and optparse) based on SGB abundance in RPKM.

### Prediction of gut metabolic modules (GMMs) and bioactive Fmetabolites

A module-based analytical framework described by Valles-Colomer^32^ and MetaCyc metabolic database were used to predict SGBs encoding related GMMs. For each SGB, the predicted open reading frames (ORFs) were compared with the Kyoto Encyclopedia of Genes and Genomes (KEGG) Orthologies (KOs) database to annotate the key gut-brain metabolic modules. The SGBs encoding related modules were identified by Omixer-RPM using the parameter -c 0.66^87^.

Gut metabolites were predicted based on high-quality sequences. One million reads per sample were subsampled using seqtk (https://github.com/lh3/seqtk), and the subsamples reads were compared by the blastx function of DIAMON “-query-cover 90-id 50”. The best hit of each gene was selected for the calculation of the gene abundance profile of each sample. Then the MelonnPan-predict workflow was used to convert gene abundances into a predicted metabolomic table^88^.

### Detection of serum SCFAs and neurotransmitters

Serum samples were thawed and well mixed prior to analysis. Each sample (50 uL) was vortex mixed with 100 μL of phosphoric acid solution (36% v/v) for three minutes, followed by adding 150 μL of methyl tert-butyl ether analytical standard (MTBE standard solution). The mixture was vortexed for 3 min again and ultrasonicated for 5 min. After that, the mixture was centrifuged, and the supernatant was collected for GC-MS analysis. An Agilent 7890B GC system coupled to a 7000D GC/MS with a DB-FFAP column (30 m length × 0.25 mm inner diameter × 0.25 μm film thickness; J&W Scientific, USA) was used to detect the SCFAs and neurotransmitters in the serum samples.

### Statistical analyses

All statistical analyses were performed using the R software (v.4.0.3), and data were expressed as mean ± SD. The Shannon index and principal coordinates analysis (PCoA) were used to assess changes in the microbiota diversity and structure in fecal samples of patients during the course of intervention; the analyses were performed with R package vegan (v.2.5-1) and ggpubr. Analysis of similarities (ANOSIM; 999 permutations) was used to evaluate differences in gut microbiota communities between Probio-M8 and placebo groups at different time points. Wilcoxon test and t-test were used to evaluate differences in the fecal microbiome and neurometabolites between groups/subgroups. In addition, a multivariable association between clinical metadata and gut microbiota was calculated and visualized by R package MaAsLin2, tidyverse, and ggplot2. Procrustes analysis based on the vegan package was used to determine the similarity between two multivariate axes, and *P* values were generated based on 999 permutations. All graphical presentations were generated under R and Adobe Illustrator (AI) environment.

### Adverse events

Patients were actively monitored for GI disorders, including common adverse events. The occurrence of any unexpected adverse event was recorded.

### Classification of evidence

The primary research question was whether the co-supplementation of the probiotic strain, Probio-M8, offered added beneficial effects when taken as an adjunct to conventional drugs (namely Benserazide and dopamine agonists) for treating PD. This study provided Class I evidence that adjuvant probiotic treatment enhanced the clinical effects (alleviated non-motor symptoms and GI symptoms) of conventional regimens in the management of PD.

## Supplementary information

Supplementary Information files

## Data Availability

The sequence dataset was deposited in the National Center for Biotechnology Information (NCBI) Sequence Read Archive (SRA) database (accession number PRJNA769968).

## Code availability

The code in our analysis is available on Github (https://github.com/TengMa-Cleap/).
