# Supplementary material for: Probiotics synergized with conventional regimen in managing Parkinson’s disease
Source: NPJ Parkinsons Dis. 2022 May 24;8:62. doi: 10.1038/s41531-022-00327-6 (PMC9130297; doi:10.1038/s41531-022-00327-6)
Supplement: Supplementary file 1 — Supplementary Information files [file 41531_2022_327_MOESM1_ESM.pdf]

Supplementary Figure 1. Flowchart of patient recruitment and study process

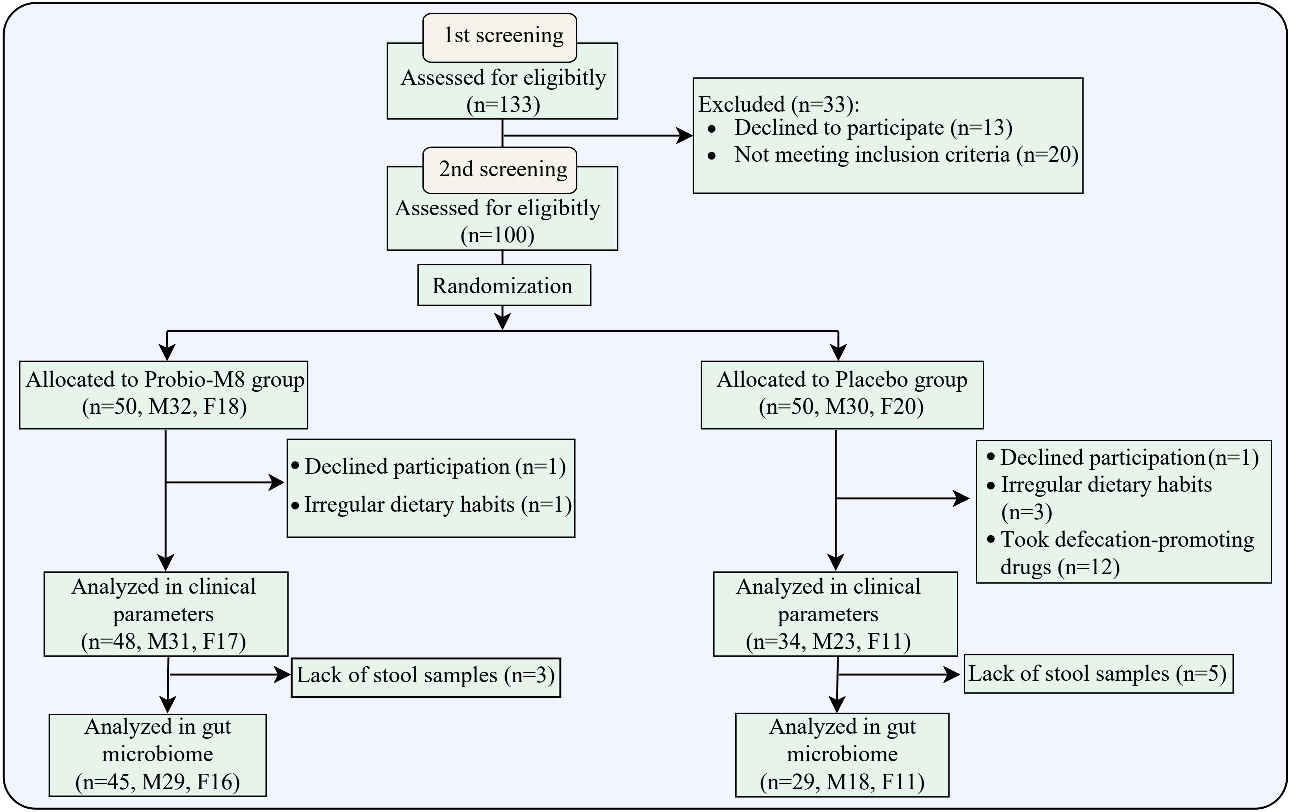

Supplementary Figure 2. The effect of age on the gut microbiota structure

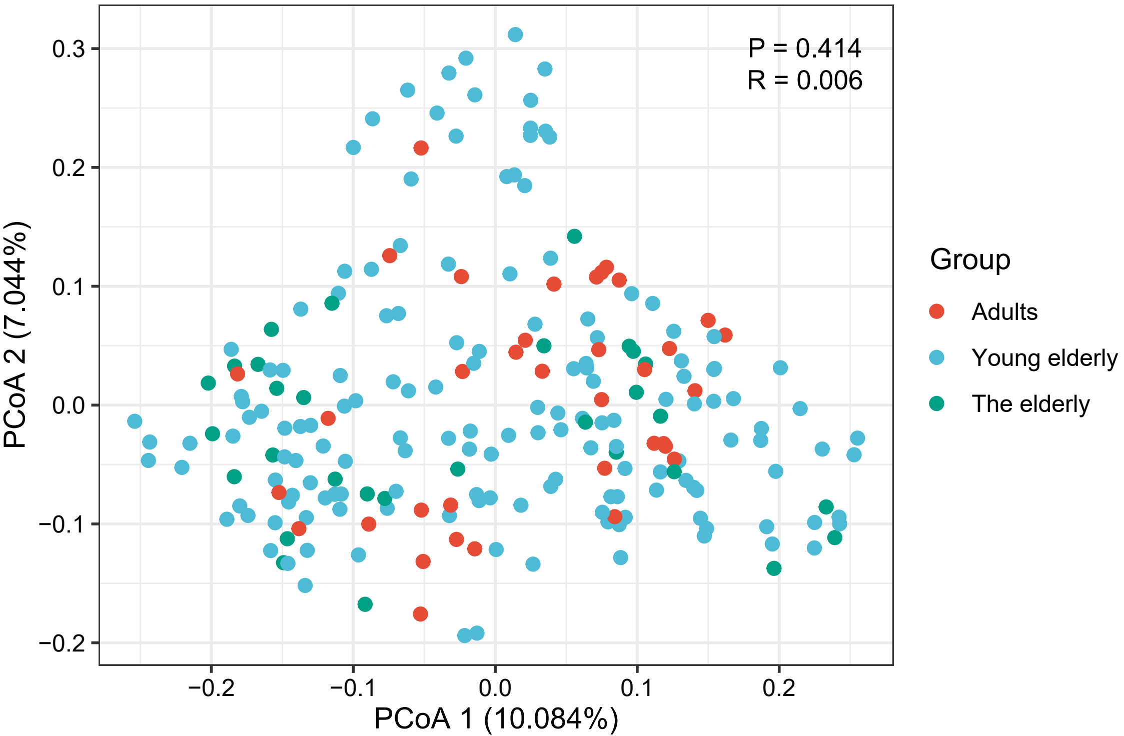

Supplementary Table 1 Information of subjects and Parkinson's disease related questionnaire scores

Subject Information

Sample_ID	Gender	Age (year)	Duration of PD (year)	Hoehn-Yahr grading	Levodopa equivalent daily dosage (mg/d)	Dopamine agonists	Group
Sample_SPA03<br>Sample_SPB03<br>Sample_SPC03	Female	72	2	1	300	-	Probiotics
Sample_SPA04<br>Sample_SPB04<br>Sample_SPC04	Female	58	2.5	1	375	√	Probiotics
Sample_SPA06<br>Sample_SPB06<br>Sample_SPC06	Male	58	5	1.5	375	√	Probiotics
Sample_SPA07<br>Sample_SPB07<br>Sample_SPC07	Female	62	9	2.5	750	-	Probiotics
Sample_SPA08<br>Sample_SPB08<br>Sample_SPC08	Female	67	7	2.5	750	√	Probiotics
Sample_SPA09<br>Sample_SPB09<br>Sample_SPC09	Female	59	6	1.5	500	√	Probiotics
Sample_SPA10<br>Sample_SPB10<br>Sample_SPC10	Female	72	3	3	1250	-	Probiotics
Sample_SPA11<br>Sample_SPB11<br>Sample_SPC11	Male	56	3	1	500	-	Probiotics
Sample_SPA12<br>Sample_SPB12<br>Sample_SPC12	Male	60	9	2.5	750	√	Probiotics
Sample_SPA13<br>Sample_SPB13<br>Sample_SPC13	Female	62	2.5	1	750	√	Probiotics
Sample_SPA15<br>Sample_SPB15<br>Sample_SPC15	Female	68	8	2.5	1125	-	Probiotics
Sample_SPA16<br>Sample_SPB16<br>Sample_SPC16	Male	66	2	1	225	-	Probiotics

Sample_SPA17<br>Sample_SPB17<br>Sample_SPC17	Female	69	9	2.5	750	√	Probiotics
Sample_SPA20<br>Sample_SPB20<br>Sample_SPC20	Female	74	3	1	500	√	Probiotics
Sample_SPA21<br>Sample_SPB21<br>Sample_SPC21	Male	59	4	2.5	1250	-	Probiotics
Sample_SPA22<br>Sample_SPB22<br>Sample_SPC22	Female	65	8	2.5	750	√	Probiotics
Sample_SPA24<br>Sample_SPB24<br>Sample_SPC24	Male	58	2.5	1.5	500	√	Probiotics
Sample_SPA25<br>Sample_SPB25<br>Sample_SPC25	Male	77	7	1.5	750	√	Probiotics
Sample_SPA26<br>Sample_SPB26<br>Sample_SPC26	Male	70	6	1.5	750	√	Probiotics
Sample_SPA27<br>Sample_SPB27<br>Sample_SPC27	Male	70	3	1	500	-	Probiotics
Sample_SPA28<br>Sample_SPB28<br>Sample_SPC28	Male	61	5	2.5	1250	√	Probiotics
Sample_SPA30<br>Sample_SPB30<br>Sample_SPC30	Female	61	7	1.5	750	-	Probiotics
Sample_SPA31<br>Sample_SPB31<br>Sample_SPC31	Male	73	3	2.5	1250	√	Probiotics
Sample_SPA32<br>Sample_SPB32<br>Sample_SPC32	Male	62	3.5	1	375	-	Probiotics
Sample_SPA33<br>Sample_SPB33<br>Sample_SPC33	Female	64	3	2.5	500	-	Probiotics
Sample_SPA34<br>Sample_SPB34<br>Sample_SPC34	Male	65	2	2	750	√	Probiotics
Sample_SPA35<br>Sample_SPB35	Male	67	3	2.5	1000	-	Probiotics

Sample_SPC35							
Sample_SPA36							
Sample_SPB36	Male	55	8	2.5	300	√	Probiotics
Sample_SPC36							
Sample_SPA37							
Sample_SPB37	Male	65	4	1.5	375	√	Probiotics
Sample_SPC37							
Sample_SPA38							
Sample_SPB38	Male	63	4	2	500	√	Probiotics
Sample_SPC38							
Sample_SPA39							
Sample_SPB39	Male	71	3	2	375	-	Probiotics
Sample_SPC39							
Sample_SPA41							
Sample_SPB41	Male	77	7	3	1250	√	Probiotics
Sample_SPC41							
Sample_SPA42							
Sample_SPB42	Male	71	10	3	225	-	Probiotics
Sample_SPC42							
Sample_SPA45							
Sample_SPB45	Male	63	2	1.5	375	√	Probiotics
Sample_SPC45							
Sample_SPA47							
Sample_SPB47	Male	73	2	1	300	√	Probiotics
Sample_SPC47							
Sample_LPA01							
Sample_LPB01	Male	52	2	2	500	-	Probiotics
Sample_LPC01							
Sample_LPA02							
Sample_LPB02	Male	58	2	1.5	500	√	Probiotics
Sample_LPC02							
Sample_LPA03							
Sample_LPB03	Male	72	5	3	750	-	Probiotics
Sample_LPC03							
Sample_LPA04							
Sample_LPB04	Female	87	6	2.5	750	√	Probiotics
Sample_LPC04							
Sample_LPA05							
Sample_LPB05	Female	70	5	1.5	500	√	Probiotics
Sample_LPC05							
Sample_LPA08							
Sample_LPB08	Female	68	3	2.5	1125	-	Probiotics
Sample_LPC08							
Sample_YPA1							

Sample_YPB1	Male	77	8	2.5	1125	-	Probiotics
Sample_YPC1							
Sample_YPA2							
Sample_YPB2	Male	65	4	1	0	√	Probiotics
Sample_YPC2							
Sample_YPA3							
Sample_YPB3	Male	72	5	2.5	750	√	Probiotics
Sample_YPC3							
Sample_YPA4							
Sample_YPB4	Male	69	6	3	1250	-	Probiotics
Sample_YPC4							
Sample_YPA5							
Sample_YPB5	Male	59	5	2	500	√	Probiotics
Sample_YPC5							
Sample_YPA6							
Sample_YPB6	Male	72	7	2.5	750	-	Probiotics
Sample_YPC6							
Sample_YPA7							
Sample_YPB7	Male	76	2	2.5	750	√	Probiotics
Sample_YPC7							
Sample_SCA55							
Sample_SCB55	Male	70	7	3	1000	√	placebo
Sample_SCC55							
Sample_SCA56							
Sample_SCB56	Male	77	5	1.5	500	-	placebo
Sample_SCC56							
Sample_SCA57							
Sample_SCB57	Male	64	3	1.5	750	√	placebo
Sample_SCC57							
Sample_SCA58							
Sample_SCB58	Female	65	2	2	750	√	placebo
Sample_SCC58							
Sample_SCA59							
Sample_SCB59	Male	62	2	3	1250	√	placebo
Sample_SCC59							
Sample_SCA60							
Sample_SCB60	Male	75	5	2.5	750	-	placebo
Sample_SCC60							
Sample_SCA61							
Sample_SCB61	Male	62	3	2.5	500	√	placebo
Sample_SCC61							
Sample_SCA62							
Sample_SCB62	Female	64	5	2	375	-	placebo
Sample_SCC62							

Sample_SCA63<br>Sample_SCB63<br>Sample_SCC63	Male	80	7	2	750	-	placebo
Sample_SCA64<br>Sample_SCB64<br>Sample_SCC64	Female	62	5	2	500	√	placebo
Sample_SCA65<br>Sample_SCB65<br>Sample_SCC65	Male	70	5	2	375	√	placebo
Sample_SCA66<br>Sample_SCB66<br>Sample_SCC66	Male	68	3	2	500	-	placebo
Sample_SCA67<br>Sample_SCB67<br>Sample_SCC67	Female	71	6	1	300	-	placebo
Sample_SCA68<br>Sample_SCB68<br>Sample_SCC68	Female	66	4	2	500	√	placebo
Sample_SCA69<br>Sample_SCB69<br>Sample_SCC69	Female	80	11	3	1250	-	placebo
Sample_SCA70<br>Sample_SCB70<br>Sample_SCC70	Male	75	7	2	375	√	placebo
Sample_LCA06<br>Sample_LCB06<br>Sample_LCC06	Female	65	2.5	2	375	√	placebo
Sample_LCA07<br>Sample_LCB07<br>Sample_LCC07	Female	69	4	3	750	√	placebo
Sample_LCA09<br>Sample_LCB09<br>Sample_LCC09	Male	74	4	1.5	300	-	placebo
Sample_LCA10<br>Sample_LCB10<br>Sample_LCC10	Male	55	4	1	225	√	placebo
Sample_LCA11<br>Sample_LCB11<br>Sample_LCC11	Male	70	9	3	1000	-	placebo
Sample_LCA12<br>Sample_LCB12<br>Sample_LCC12	Male	69	2.5	1.5	375	√	placebo
Sample_LCA13<br>Sample_LCB13	Male	70	8	2.5	500	-	placebo

Sample_LCC13							
Sample_LCA14							
Sample_LCB14	Male	79	5	3	1000	-	placebo
Sample_LCC14							
Sample_LCA15							
Sample_LCB15	Male	75	5	3	1125	-	placebo
Sample_LCC15							
Sample_LCA16							
Sample_LCB16	Male	74	5	3	750	√	placebo
Sample_LCC16							
Sample_LCA17							
Sample_LCB17	Female	73	2	3	750	-	placebo
Sample_LCC17							
Sample_LCA19							
Sample_LCB19	Male	68	2.5	2	375	√	placebo
Sample_LCC19							
Sample_LCA20							
Sample_LCB20	Female	69	2	1.5	300	√	placebo
Sample_LCC20							
Sample_LCA21							
Sample_LCB21	Male	70	7	2.5	300	√	placebo
Sample_LCC21							
Sample_LCA22							
Sample_LCB22	Male	46	2.5	2	375	√	placebo
Sample_LCC22							
Sample_LCA23							
Sample_LCB23	Male	66	3	2.5	500	-	placebo
Sample_LCC23							
Sample_LCA24							
Sample_LCB24	Female	73	5	3	1000	√	placebo
Sample_LCC24							
Sample_LCA25							
Sample_LCB25	Male	62	2	1.5	375	-	placebo
Sample_LCC25							

Parkinson's disease related questionnaire scores

Group_time (P: probiotic; C: placebo; A: 0d; B: 1M; C: 3M)	MMSE	HAMA	HAMD-17	PDSS	VSA	UPDRS-III
PA	29	18	6	104	0	6
PB	30	12	4	112	0	6
PC	30	11	4	112	0	6
PA	26	12	11	100	0	9
PB	28	13	11	97	0	9
PC	29	12	9	98	0	9
PA	29	21	14	128	1	4
PB	26	17	11	130	1	4
PC	26	16	11	130	1	4
PA	30	12	4	117	0	14
PB	29	9	3	116	0	16
PC	30	8	3	116	0	16
PA	24	22	12	73	4	32
PB	28	21	12	72	4	32
PC	28	17	11	99	3	31
PA	20	24	7	83	4	22
PB	20	17	7	95	4	22
PC	20	10	7	105	2	22
PA	24	17	9	67	1	43
PB	26	17	8	67	1	43
PC	24	10	6	81	1	43
PA	19	12	6	137	0	14
PB	24	8	5	139	0	14
PC	28	7	4	145	0	12
PA	28	15	7	116	0	24
PB	30	14	5	115	0	24
PC	29	12	2	114	0	22
PA	26	10	3	135	0	7
PB	29	10	3	142	0	7
PC	29	10	3	142	0	7
PA	25	21	8	136	0	28
PB	25	21	7	136	0	28
PC	27	16	7	137	0	29
PA	25	11	1	120	0	6
PB	25	11	1	122	0	6
PC	25	11	1	121	0	6

PA	24	11	9	117	0	25
PB	26	11	9	106	0	25
PC	26	11	9	106	0	25
PA	19	22	14	111	4	11
PB	21	22	13	114	4	11
PC	21	17	13	127	3	11
PA	29	23	8	92	0	32
PB	30	21	8	92	0	32
PC	30	17	7	107	0	32
PA	21	24	11	78	4	21
PB	21	22	11	84	4	21
PC	22	19	9	84	4	21
PA	29	3	0	138	0	19
PB	30	3	0	144	0	18
PC	30	3	0	140	0	17
PA	26	37	9	91	0	19
PB	29	36	9	102	0	19
PC	29	29	9	100	0	19
PA	24	12	3	133	0	12
PB	24	11	3	131	0	12
PC	23	10	3	134	0	12
PA	28	7	0	137	0	7
PB	26	7	0	134	0	7
PC	26	7	0	134	0	7
PA	26	9	5	107	0	34.5
PB	26	9	5	107	0	34.5
PC	26	9	5	117	0	32.5
PA	28	14	7	83	0	11
PB	27	14	7	85	0	12
PC	27	12	6	87	0	12
PA	17	24	11	115	0	31
PB	19	20	9	117	0	30
PC	19	11	9	117	4	30
PA	28	15	7	137	0	12
PB	28	15	7	147	0	12
PC	28	13	7	147	0	14
PA	23	10	14	102	0	32
PB	23	8	14	100	0	32
PC	23	7	12	103	0	32
PA	17.5	21	10	117	0	17
PB	17	18	10	135	0	17
PC	17	15	9	136	0	17
PA	28	11	6	91	0	24.5
PB	26	11	7	86	0	28

PC	26	10	7	107	0	28
PA	22	25	20	82	0	26
PB	26	24	16	82	0	19
PC	29	21	16	116	0	19
PA	30	7	7	100	0	13
PB	30	7	6	100	0	13
PC	30	7	6	103	0	13
PA	27	18	8	83	4	18
PB	30	17	8	87	1	20
PC	30	13	7	111	1	20
PA	30	3	2	122	0	18
PB	30	3	2	140	0	18
PC	30	3	2	133	0	18
PA	23	5	4	109	0	37
PB	26	5	4	108	0	37
PC	26	3	4	108	0	37
PA	26	10	5	136	4	10
PB	27	7	3	136	3	10
PC	28	6	3	130	3	10
PA	28	1	3	129	0	14
PB	30	1	2	133	0	14
PC	30	1	2	135	0	14
PA	19	9	5	124	1	10
PB	20	8	5	124	1	10
PC	21	7	4	124	1	10
PA	30	16	7	125	0	13
PB	30	13	6	131	0	10
PC	30	11	2	131	0	10
PA	30	5	6	138	0	15
PB	30	5	6	144	0	15
PC	30	4	5	144	0	15
PA	29	12	8	138	0	22
PB	29	10	8	138	0	22
PC	30	9	6	138	0	19
PA	19	3	4	113	0	13
PB	19	3	4	113	0	10
PC	19	1	0	138	0	10
PA	21	28	26	110	0	35
PB	21	20	16	124	0	28
PC	20	12	8	128	0	21
PA	18	23	14	133	0	13
PB	20	21	14	133	0	13
PC	21	21	14	133	0	13
PA	30	14	11	79	0	16

PB	30	7	6	133	0	10
PC	30	6	6	134	0	10
PA	30	6	10	136	1	26
PB	30	6	4	125	0	11
PC	30	1	1	146	0	9
PA	27	23	12	106	1	31
PB	30	14	7	119	4	16
PC	30	12	7	121	2	15
PA	20	34	25	109	0	28
PB	28	28	14	121	0	13
PC	28	24	12	133	0	11
PA	30	29	26	78	7	24
PB	29	8	18	118	0	14
PC	30	18	18	103	3	9
PA	28	19	18	111	1	19
PB	28	16	11	124	0	29
PC	30	14	14	112	0	29
PA	23	23	17	106	0	12
PB	28	14	9	138	0	9
PC	28	12	8	142	0	9
CA	30	19	10	130	4	33
CB	30	16	10	125	4	33
CC	30	16	10	125	4	33
CA	30	18	8	141	0	17
CB	30	18	7	140	0	17
CC	30	17	7	140	0	19
CA	24	16	6	132	0	16
CB	25	26	6	132	0	16
CC	24	16	6	140	0	16
CA	25	20	11	125	2	32
CB	25	21	11	120	2	32
CC	26	19	11	120	2	32
CA	23	15	5	135	0	18
CB	24	15	5	135	0	18
CC	24	14	5	135	0	20
CA	23	17	6	118	0	22
CB	23	17	6	118	0	22
CC	24	16	6	124	0	22
CA	28	24	8	120	0	22
CB	28	24	8	120	0	22
CC	28	23	8	125	0	26
CA	28	16	5	140	0	16
CB	28	15	5	140	0	16
CC	28	15	5	140	0	16

CA	20	16	5	138	0	16
CB	20	16	5	138	0	16
CC	20	16	5	140	0	16
CA	30	19	7	117	0	16
CB	29	18	6	116	0	16
CC	30	18	6	116	0	16
CA	26	13	4	136	0	18
CB	26	13	4	136	0	18
CC	28	13	4	136	0	18
CA	27	15	5	128	0	16
CB	28	14	5	130	0	16
CC	27	14	5	130	0	16
CA	26	17	8	136	4	10
CB	27	16	7	136	3	10
CC	27	16	7	130	3	10
CA	26	13	6	138	0	16
CB	26	13	6	135	0	16
CC	27	13	5	130	0	16
CA	24	24	12	105	3	30
CB	24	13	12	110	3	30
CC	25	23	12	108	3	30
CA	30	11	4	118	0	16
CB	30	11	4	118	0	16
CC	29	11	4	120	0	16
CA	20	22	25	106	0	17
CB	20	18	22	103	0	17
CC	19	14	21	100	0	17
CA	15	13	13	150	0	15
CB	15	12	11	144	0	15
CC	15	12	11	144	0	15
CA	28	16	6	131	0	15
CB	28	16	6	140	0	15
CC	28	16	4	131	0	15
CA	30	10	7	120	0	5
CB	30	8	5	120	0	4
CC	30	8	5	130	0	6
CA	29	15	20	130	0	11
CB	30	13	19	125	0	10
CC	30	14	19	138	0	11
CA	27	21	13	79	0	14
CB	28	21	13	90	0	16
CC	28	21	13	90	0	16
CA	29	13	11	150	0	22
CB	28	13	11	150	0	22

CC	29	13	11	150	0	22
CA	17	23	17	130	0	29
CB	18	22	15	128	0	29
CC	17	22	15	126	0	32
CA	29	6	3	104	1	16
CB	29	6	3	110	1	16
CC	30	6	3	106	1	18
CA	23	5	3	106	0	18
CB	22	5	3	110	0	18
CC	23	5	3	110	0	18
CA	24	8	7	145	1	26
CB	24	7	6	145	1	26
CC	26	7	6	145	1	26
CA	27	4	5	141	1	9
CB	28	4	5	145	1	9
CC	28	4	5	145	1	9
CA	16	7	7	132	1	15
CB	16	7	7	140	1	15
CC	16	7	7	140	1	15
CA	29	16	12	130	0	24
CB	30	14	11	135	0	24
CC	30	14	11	135	0	25
CA	30	3	6	147	0	5
CB	30	3	6	145	0	5
CC	30	3	6	145	0	5
CA	26	19	19	111	0	20
CB	27	17	17	117	0	16
CC	26	17	17	117	0	16
CA	17	23	22	106	0	28
CB	18	21	21	110	0	28
CC	18	20	21	110	0	28
CA	30	10	7	123	0	13
CB	30	10	7	128	0	13
CC	30	8	7	126	0	14

			Constipation-related Symptoms scores					
ADL	PAC-QCL	Bristol scores	Difficulty in defecation	Hardness of feces	Incomplete defecation	Assisted defecation by hand	spontaneous defecation	completed defecation
17	78	3	2	2	3	1	6	0
17	73	3	2	1	2	1	7	2
17	61	4	2	1	1	1	7	3.5
18	78	3	2	3	0	1	3	3
18	68	3	1	1	0	0	3	3
18	64	4	1	1	0	0	3.5	3.5
14	83	4	2	2	3	1	3	0
14	64	4	1	1	1	0	3.5	2
14	50	4	1	1	1	0	3.5	3
15	59	4	3	2	1	2	6	4
14	55	4	2	1	1	1	6	5
14	52	4	1	0	1	1	7	5.5
24	90	1	3	3	2	3	1.5	0
24	70	2	3	2	1	1	2	1
24	59	4	2	1	0	0	2	2
15	76	5	2	1	3	1	6	0
15	56	5	1	1	2	0	6	1
15	46	4	1	1	1	0	6	3.5
31	87	2	3	3	3	3	5	0
31	75	3	2	2	2	2	7	2
31	61	3	2	2	1	2	7	3
16	59	3	3	1	0	1	5.5	5.5
16	50	3	2	1	0	1	6.5	6.5
16	45	4	1	1	0	0	7	7
14	95	2	3	3	2	2	4	1
14	58	4	2	1	1	0	4	2
14	51	4	2	1	0	0	4	4
15	55	4	1	1	0	0	2	2
15	58	4	1	1	1	0	1.5	0.5
15	48	4	1	1	1	0	2.5	2
20	82	2	3	3	0	0	2.5	2.5
20	65	3	2	1	0	0	3	3
20	46	4	1	1	0	0	3.5	3.5
14	75	3	3	2	3	0	7	0
14	49	4	2	1	2	0	7	2
14	43	4	1	1	2	0	7	3.5

14	78	3	2	3	1	1	2.5	2.5
14	49	4	0	0	0	0	7	7
14	41	4	0	0	0	0	7	7
14	69	3	2	2	3	0	2.5	1
14	58	4	1	1	2	0	3.5	2.5
14	63	4	1	1	1	0	3.5	2.5
14	78	1	3	3	0	1	1	1
14	70	3	2	2	0	1	2	2
14	66	4	3	1	0	0	3	3
17	79	4	3	1	1	1	2	1.5
17	71	4	2	2	0	0	3	3
17	65	3	1	1	1	0	3	3
14	65	2	3	3	2	3	2	1
14	56	3	2	2	2	2	2	1.5
14	54	3	2	2	1	1	3	2
14	91	2	3	3	3	1	1.5	0
14	80	3	2	2	1	1	3	1
14	73	3	2	2	1	0	3	2
18	77	3	3	3	2	1	2.5	1.5
18	66	4	2	2	2	1	3	2.5
18	64	4	2	2	1	1	3	2.5
15	85	2	3	3	0	1	2.5	2.5
15	66	3	2	2	0	1	4	4
15	50	4	1	2	0	0	4	4
14	92	3	3	3	1	1	2.5	2
14	78	3	2	2	1	1	3.5	3
14	74	3	2	2	0	0	3	3
17	52	3	3	2	3	0	1.5	0
17	58	4	2	1	2	0	3	1
17	54	4	2	1	2	0	3	1
42	79	2	2	3	2	1	2.5	1
42	72	3	2	2	0	0	4.5	4.5
42	70	3	2	1	0	0	4.5	4.5
17	88	3	3	3	0	2	3	3
17	75	4	2	2	0	0	6	6
16	74	4	2	2	0	0	6	6
18	74	4	2	2	2	0	7	4
17	66	4	2	2	0	0	7	7
17	60	4	2	2	0	0	7	7
17	75	3	3	3	3	2	1.5	0
17	68	3	2	3	1	2	3	1
17	65	3	2	2	1	1	2	1.5
36	46	1	3	3	1	1	3.5	3
36	47	2	2	3	1	1	3	1.5

43	46	2	2	3	1	1	3	2
17	70	4	2	0	3	0	3.5	0
17	67	4	2	1	1	0	4	2
17	66	4	2	1	1	0	3.5	1.5
14	81	3	3	3	2	1	1.5	0.5
14	77	3	2	2	1	1	3	2
14	73	3	2	2	1	1	2.5	1.5
23	64	4	2	1	1	0	3.5	3.5
23	62	4	2	1	0	0	4	4
23	59	4	2	1	0	0	4	4
14	84	2	3	3	1	0	1	0.5
14	75	3	2	2	0	0	3	3
14	74	3	2	1	0	0	2.5	2.5
36	85	1	3	3	3	0	1	0
36	77	3	2	1	0	0	3	3
36	75	3	2	2	0	0	3	3
18	57	3	2	2	0	0	3	3
18	52	3	2	2	0	0	4	4
18	50	4	2	2	0	0	5	5
14	49	5	3	2	0	0	2.5	2.5
14	55	4	2	2	0	0	4	4
14	50	4	2	2	0	0	5	5
16	86	1	2	2	2	0	2	0.5
16	71	3	2	2	1	0	3	2
16	70	4	1	1	0	0	4	4
14	41	1	2	1	3	0	3.5	0
14	40	3	2	1	3	0	3.5	2
14	33	3	1	0	3	0	5.5	3
16	60	4	2	2	0	0	3	1.5
16	58	4	2	1	0	0	3	2
16	58	3	2	1	0	0	3	2.5
24	69	2	2	2	1	1	3.5	0
24	45	3	1	1	0	0	3.5	1.5
24	54	3	2	2	1	1	5	3
29	46	2	2	3	2	2	6	2
29	46	3	2	3	2	2	6	4
29	34	4	1	1	0	0	8.5	5
38	110	3	3	3	3	3	1.5	0
38	78	4	2	2	3	2	1.5	0.5
36	36	4	1	1	0	0	2.5	0.5
24	51	2	0	0	0	0	2.5	1
24	51	3	0	0	0	0	2.5	1
24	51	3	0	0	0	0	2.5	1
20	54	3	3	2	3	0	1.5	0

15	33	3	2	2	1	1	3.5	3
14	37	4	2	1	2	0	3	1
24	86	3	2	3	2	1	2.5	1
15	29	4	2	1	2	0	3	1
14	33	3	2	1	0	0	4.5	4.5
20	68	2	3	3	0	2	3	3
19	42	3	2	2	0	0	4.5	4.5
17	45	4	2	2	0	0	6	6
35	129	3	2	2	2	0	7	4
20	77	4	2	2	0	0	6	6
19	61	4	2	2	0	0	7	7
39	49	4	3	3	3	2	1.5	0
32	38	4	2	2	0	0	7	7
25	42	3	2	2	1	1	2	1.5
28	45	3	3	3	1	1	3.5	3
36	45	3	2	3	1	2	3	1
31	48	2	2	3	1	1	3	2
21	52	1	2	0	3	0	3.5	0
21	42	2	2	3	1	1	3	1.5
24	40	4	2	1	1	0	3.5	1.5
25	82	2	3	3	1	1	1.5	1
25	76	2	3	3	1	1	2	1
25	75	2	3	3	1	1	2	1
15	78	1	3	3	1	0	1.5	0.5
15	82	2	3	3	1	0	1.5	0.5
15	80	2	3	3	1	0	1.5	0.5
16	79	3	3	2	1	0	1	0.5
16	79	3	3	2	1	0	2	1
16	79	3	3	2	1	0	2	1
25	76	5	2	1	3	1	6	0
25	83	4	2	2	2	1	4	0
25	80	4	2	2	2	1	4	2
16	87	2	3	3	3	3	5	0
16	85	3	3	3	2	3	6	0
16	83	3	2	3	2	3	6	2
18	59	3	3	1	0	1	5.5	5.5
18	72	3	3	2	0	1	6	6
18	70	3	3	2	0	1	6	6
16	88	3	3	3	0	2	3	3
16	79	3	3	3	0	2	3	3
20	80	3	3	3	0	2	3	3
14	69	3	2	2	3	0	2.5	1
14	90	4	3	2	2	0	3	1
14	88	4	3	2	2	0	3	1

20	65	2	3	3	2	3	2	1
20	76	2	2	3	2	2	2	1
20	76	3	2	3	1	2	2	1
14	59	4	3	2	1	2	6	4
14	67	3	3	2	1	2	5	4
14	67	3	3	2	1	2	5	4
14	70	4	2	0	3	0	3.5	0
14	72	3	2	0	2	0	4	0
14	70	3	2	0	2	0	4	1
14	81	3	3	3	2	1	1.5	0.5
14	83	3	3	3	2	1	1.5	0.5
14	83	3	3	3	1	1	1.5	0.5
18	84	2	3	3	1	0	1	0.5
18	83	3	3	3	1	0	2	0.5
18	83	3	3	3	1	0	2	0.5
14	64	4	2	1	1	0	3.5	3.5
14	80	4	2	2	1	0	3.5	3
14	86	4	2	2	1	0	3.5	3.5
42	85	1	3	3	3	0	1	0
42	80	1	3	3	2	0	2	0
42	78	2	2	3	2	0	2	0
15	86	1	3	3	0	0	1	1
15	77	2	3	3	0	0	1	1
15	79	2	3	2	0	0	1	1
15	80	2	2	2	1	1	2	1
15	61	2	1	1	1	0	2	1
15	50	2	1	0	0	0	3	1
33	62	1	2	2	0	0	3	0.5
33	60	2	2	2	0	0	3	1.5
33	50	2	2	2	0	0	3	1.5
34	66	3	2	2	2	3	3	0.5
34	60	3	2	2	2	3	3.5	0.5
34	66	3	2	2	2	3	3.5	1
14	55	5	2	3	2	0	2.5	1
14	55	4	2	2	2	0	2.5	1
14	50	4	2	2	2	0	2.5	1.5
19	54	2	2	2	2	0	2.5	1
19	50	3	2	2	1	0	2.5	1
19	58	3	2	1	1	0	3	0.5
15	66	3	2	2	2	2	2.5	1.5
15	64	3	2	2	2	2	2.5	1.5
15	64	3	2	2	2	1	2.5	1
14	87	3	2	2	0	0	1.5	1
14	87	3	1	2	0	0	1.5	0.5

14	80	3	1	1	0	0	1.5	1.5
50	82	3	0	1	1	0	3.5	2
50	80	4	0	1	1	0	3.5	2
50	80	4	0	1	1	0	3.5	2
26	46	2	3	0	0	1	1	1
26	46	2	3	1	0	0	1	1
26	50	3	3	0	0	0	1	1
23	46	4	1	1	1	1	2	0
23	40	3	1	1	1	1	2	0
23	48	3	1	1	1	1	2	0.5
30	51	4	2	2	0	0	4.5	1
30	47	3	2	2	0	0	4	1
30	50	3	2	2	0	0	5	1
22	54	3	2	2	1	0	3.5	0.5
22	54	3	2	2	1	0	4	1
22	60	3	2	2	1	0	4	1
26	53	2	2	2	1	0	3.5	0.5
26	53	3	2	2	1	0	3.5	0.5
26	53	3	2	2	1	0	3.5	0.5
42	70	4	3	2	2	1	2.5	3.5
42	70	4	3	2	1	0	2.5	1.5
42	75	4	3	2	1	0	3	1
14	48	1	1	1	0	0	4.5	2
14	43	1	1	1	0	0	5	2
14	43	2	1	1	0	0	5	1.5
29	70	1	2	3	2	1	2.5	1
29	70	2	2	3	2	0	3	1
29	65	2	1	3	1	0	3	1
45	114	3	3	3	3	3	2	1
40	110	3	3	3	3	0	2	0.5
40	110	3	3	3	3	0	2	1
16	54	2	2	2	2	0	2.5	1.5
16	50	2	2	2	2	0	2.5	1.5
16	50	2	3	2	2	0	3	1

**sample collection**		**demographic data comparison (Wilcox-test)**			
Fecal sample	Blood sample		Probio-M8 group	Placebo group	*P*-value
√	√	Male (%)	66.67	67.65	-
√	√	Female (%)	33.33	32.35	-
√	√	Age (years)	66.46±6.98	68.76±6.91	0.08
√	√	Disease duration (years)	4.75±2.34	4.56±2.18	0.75
√	√	Hoehn-Yahr grading	1.98±0.68	2.22±0.62	0.13
√	√	Medications for Parkinson's disease			
√	√	Levodopa equivalent daily dosage (mg/d)	671.35±320.81	611.76±291.50	0.32
√	√	Levodopa (%)	97.91	100	-
√	√	Dopamine agonists (%)	58.33	55.88	-
√	√				
√	√				
√	√				
√	√				
√	√				
√	√				
√	√				
√	√				
√	√				
√	√				
√	√				
√	√				
√	√				
√	√				
√	√				
√	√				
√	√				
√	√				
√	√				
√	√				
√	√				
√	√				
√	√				
√	√				
√	√				
√	√				
√	√				

√	√
√	√
√	√
√	√
√	√
√	√
√	√
√	√
√	√
√	√
√	√
√	√
-	-
-	-
-	-
√	√
√	√
√	√
√	√
√	√
√	√
√	√
√	√
√	√
√	√
√	√
√	√
√	√
√	√
√	√
√	√
√	√
√	√
√	√
√	√
√	√
-	-
-	-
-	-
√	√
√	√
√	√
√	√
√	√

√	√
√	√
√	√
√	√
√	√
√	√
√	√
√	√
√	√
√	√
√	√
√	√
√	√
√	√
√	√
√	√
√	√
√	√
√	√
√	√
√	√
√	√
√	√
√	√
√	√
√	√
√	√
√	√
√	√
√	√
√	√
√	√
√	√
√	√
√	√
√	√
√	√
√	√
√	√
√	√
√	√
√	√
√	√

√	√
√	√
√	√
√	√
√	√
√	√
√	√
√	√
√	√
√	√
√	√
√	√
√	√
√	√
√	√
√	√
√	√
√	√
√	√
√	√
√	√
√	√
√	√
-	-
-	-
-	-
-	-
-	-
-	-
√	√
√	√
√	√
√	√
√	√
√	√
√	√
√	√
√	√
√	√
√	√
√	√
-	-
-	-
-	-

-	-
-	-
-	-
√	√
√	√
√	√
√	√
√	√
√	√
√	√
√	√
√	√
√	√
√	√
√	√
√	√
√	√
√	√
√	√
√	√
√	√
√	√
√	√
√	√
-	-
-	-
-	-
-	-
-	-
-	-
√	√
√	√
√	√
√	√
√	√
√	√
√	√
√	√
√	√
√	√
√	√
√	√
√	√
√	√

√	√
√	√
√	√
√	√
√	√
√	√
√	√
√	√
√	√
√	√
√	√
√	√
√	√
√	√
√	√
√	√
√	√
√	√
√	√
√	√
√	√
√	√
√	√
√	√
√	√
√	√
√	√
√	√
√	√
√	√
√	√
√	√
√	√
√	√

Supplementary Table 2 Metagenome sequencing and assembly statistics

Sample ID	Amount of generated data		Assembly results				
	Raw data (Gbp)	Clean data (Gbp)	Number of contigs	Total length (bp)	N50 length (bp)	N90 length (bp)	Max length (bp)
Sample_SPA03	6.11905	6.02379	31362	287600850	22328	2989	538687
Sample_SPB03	5.39588	5.32104	30396	253910167	17160	2871	640918
Sample_SPC03	4.78865	4.72550	28146	220323249	17408	2699	670764
Sample_SPA04	5.66678	5.62312	34023	287141009	17135	2907	371797
Sample_SPB04	5.30639	5.24031	25494	170239853	10451	2587	446210
Sample_SPC04	5.29330	5.25554	29071	231274159	16240	2752	1332897
Sample_SPA06	7.93244	7.85500	30941	299952347	26392	3063	561709
Sample_SPB06	7.04168	6.96360	28443	302840408	28436	3333	637343
Sample_SPC06	5.27148	5.23029	18596	180302318	25052	3101	651458
Sample_SPA07	4.49438	4.45410	24635	221539434	20723	3019	534441
Sample_SPB07	5.55655	5.49638	28422	242868936	18898	2874	647783
Sample_SPC07	5.93264	5.84309	26968	270096764	27207	3165	914180
Sample_SPA08	4.41275	4.26606	34800	288405086	16985	2837	613077
Sample_SPB08	4.90034	4.80883	28422	242868936	18898	2874	647783
Sample_SPC08	6.14048	6.06589	35746	296218295	16858	2863	727784
Sample_SPA09	5.66873	5.49473	29814	240826223	16153	2844	631242
Sample_SPB09	5.01635	4.97242	25288	216332907	18774	2889	547901
Sample_SPC09	4.25467	4.22283	23756	199618492	17542	2884	889727
Sample_SPA10	4.69581	4.58175	14414	146501393	26855	3204	516591
Sample_SPB10	4.39159	4.16542	17604	173339557	27876	3144	752205
Sample_SPC10	6.21059	5.04686	15001	151045615	30026	3080	579622
Sample_SPA11	5.83793	5.76419	31922	291858365	19226	3093	584568
Sample_SPB11	3.85383	3.82304	22205	194186373	17414	3008	512289
Sample_SPC11	5.63395	5.56997	29382	286780153	23392	3185	657157
Sample_SPA12	4.97186	4.91577	52076	356691221	10473	2680	528650
Sample_SPB12	5.42410	5.38088	43355	318936801	12464	2711	804386
Sample_SPC12	5.00531	4.94353	38423	303572703	14829	2813	720644
Sample_SPA13	5.41307	5.34968	20526	199474980	32832	2970	514140
Sample_SPB13	4.84955	4.80911	17762	196225198	34644	3302	545232
Sample_SPC13	4.59426	4.54097	20520	225885770	38595	3229	778278
Sample_SPA15	6.62812	6.56276	31328	231478958	13270	2672	588242
Sample_SPB15	4.87495	4.82137	20389	150403900	13474	2702	440108
Sample_SPC15	5.08030	5.03515	24925	184418160	12898	2702	468562
Sample_SPA16	6.74309	6.57961	28365	226882299	14153	2927	604059
Sample_SPB16	4.94674	4.42473	17534	130885947	13245	2714	570725
Sample_SPC16	6.57864	6.44286	21944	179237839	16757	2839	530796
Sample_SPA17	5.77959	5.72146	16084	174978804	32372	3325	640900
Sample_SPB17	6.26904	6.20665	4098	64641746	57143	4625	473258
Sample_SPC17	4.98291	4.91383	11911	143509542	32298	3912	473176
Sample_SPA20	4.96702	4.91722	17336	134367844	14097	2773	392510
Sample_SPB20	6.63130	6.56431	29588	242905031	16145	2862	568666
Sample_SPC20	6.29785	6.23142	26522	206616295	14653	2799	718767
Sample_SPA21	5.92004	5.86939	31096	269935593	19417	2920	588090
Sample_SPB21	5.26843	5.21165	31513	270031679	21199	2806	535190
Sample_SPC21	5.14612	5.07907	27303	248513152	23952	2912	541533
Sample_SPA22	7.09997	6.99320	38141	287623454	14539	2698	546495
Sample_SPB22	5.35952	5.29867	28695	242419471	19154	2833	670013
Sample_SPC22	5.20169	5.13825	35429	311604639	20296	2943	539027
Sample_SPA25	5.87692	5.78603	26362	226191344	20439	2843	601534

Sample_SPB25	5.23351	5.17066	29967	242159764	15495	2837	593898
Sample_SPC25	5.83388	5.78171	39574	285467168	11802	2734	712304
Sample_SPA26	5.03873	4.97847	32734	260977975	15758	2797	563709
Sample_SPB26	6.14677	6.05499	28198	263474305	23552	3003	558789
Sample_SPC26	6.11784	6.04073	27476	246621985	20555	2986	1002984
Sample_SPA27	4.84508	4.76920	14070	139257982	21303	3400	569089
Sample_SPB27	5.00797	4.95554	18871	190201333	29892	3185	591822
Sample_SPC27	7.04562	6.94502	20936	249409118	34460	3640	582550
Sample_SPA28	4.43888	4.39652	25447	186684221	12256	2742	530745
Sample_SPB28	5.28296	5.09149	29633	219741453	13174	2730	647641
Sample_SPC28	5.53903	5.46823	26318	214393553	15421	2876	673704
Sample_SPA30	5.12908	5.02513	26294	210821343	16611	2805	726593
Sample_SPB30	5.10252	5.04170	26899	233501616	18316	2984	856709
Sample_SPC30	6.21337	6.04110	25225	236724348	18212	3231	526173
Sample_SPA31	6.89938	6.78475	18171	165375500	24875	2871	1495245
Sample_SPB31	0.30665	0.30336	27755	229325634	16692	2874	505922
Sample_SPC31	5.82225	5.74211	25795	212665991	17107	2828	785241
Sample_SPA32	5.75777	5.64613	22744	228893932	23721	3282	733045
Sample_SPB32	5.52080	5.47252	16545	193456620	32369	3636	732480
Sample_SPC32	5.60657	5.52733	16383	181350567	31148	3382	688307
Sample_SPA34	5.29960	5.23697	29248	269454600	22569	3001	637805
Sample_SPB34	4.78909	4.70892	30703	262138691	18110	2906	689143
Sample_SPC34	5.41456	5.33344	26735	230256435	21176	2852	727839
Sample_SPA35	5.02834	4.96619	28641	240810518	17526	2872	713013
Sample_SPB35	6.37430	6.25767	28641	240810518	17526	2872	713013
Sample_SPC35	4.80776	4.75310	27662	248510764	24171	2855	803478
Sample_SPA36	5.17088	5.12028	24773	191014725	14521	2733	637637
Sample_SPB36	6.08668	5.86982	31011	218090559	11890	2621	385966
Sample_SPC36	4.75531	4.69854	20036	168959706	17453	2867	612701
Sample_SPA37	4.97749	4.92431	33603	250972268	13254	2727	512656
Sample_SPB37	6.21078	6.15053	30100	281356756	21618	3089	737234
Sample_SPC37	4.75548	4.68911	26006	210317766	15604	2815	592231
Sample_SPA38	6.35352	6.29898	33862	300768361	18459	3037	588540
Sample_SPB38	4.87385	4.83918	23324	211738263	20418	3030	528401
Sample_SPC38	5.79927	5.74806	24463	210115175	19088	2872	898532
Sample_SPA39	6.90167	6.79357	26820	278676431	31373	3267	649968
Sample_SPB39	5.62322	5.58573	29928	242904969	15345	2876	691090
Sample_SPC39	4.01862	3.97120	26463	227246747	19378	2903	720051
Sample_SPA41	5.21061	5.15502	27894	229423557	15981	2884	637625
Sample_SPB41	6.41134	6.31435	25262	214911447	19633	2842	599109
Sample_SPC41	4.41800	4.35426	26042	194055326	14192	2678	470454
Sample_SPA42	4.80907	4.76389	35647	242104533	10448	2628	1025369
Sample_SPB42	6.36975	6.30231	39176	296975967	13070	2784	999242
Sample_SPC42	5.07576	5.02414	37546	263797147	11432	2654	681630
Sample_SPA45	5.15868	5.07465	30553	288564612	21825	3145	610536
Sample_SPB45	7.06092	6.99089	25270	231624063	22849	2970	594125
Sample_SPC45	4.65015	4.58890	32037	275419778	17568	2940	407948
Sample_SPA47	6.45666	6.36672	26176	260916878	25494	3141	638445
Sample_SPB47	6.32749	6.26077	28969	274349691	21354	3125	772588
Sample_SPC47	4.31934	4.19383	28315	230915240	16319	2846	545984
Sample_LPA01	4.95202	4.91610	26286	226506087	19056	2920	687625
Sample_LPB01	5.67695	5.62033	24271	214693541	19242	2976	819899
Sample_LPC01	5.81072	5.76807	30101	253803772	17913	2900	428691
Sample_LPA02	4.92592	4.85973	37420	290625431	14664	2783	672968
Sample_LPB02	5.55606	5.50548	34023	285127761	18007	2868	667645
Sample_LPC02	5.10960	5.07883	32589	284832936	18850	2973	573420

Sample_LPA03	3.56784	3.53768	15390	144775837	21763	3033	575014
Sample_LPB03	3.81948	3.78623	20490	188296226	20356	3013	623011
Sample_LPC03	5.58913	5.54438	31542	253417541	16958	2793	875092
Sample_LPA04	5.54553	5.40647	23698	195352512	18602	2751	564635
Sample_LPB04	5.04616	5.00140	24891	227709006	20673	3057	500033
Sample_LPC04	5.42732	5.37866	23962	242209248	26664	3168	534856
Sample_LPA05	3.03681	2.99817	29132	205485834	11152	2703	545162
Sample_LPB05	5.84765	5.78552	29722	272079459	21574	3013	696611
Sample_LPC05	6.22720	6.17341	30605	257516672	18456	2873	584318
Sample_LPA08	3.39861	3.37200	14993	161381522	27327	3442	725380
Sample_LPB08	3.82301	3.78752	16323	167647647	26562	3270	487224
Sample_LPC08	2.71452	2.69578	16277	150726907	19478	3163	637475
Sample_YPA1	3.54469	3.50839	3043	44256935	49540	4406	615990
Sample_YPB1	3.49147	3.43547	14611	154923020	36697	3230	527301
Sample_YPC1	3.20038	3.14965	9838	119776911	40957	3514	696195
Sample_YPA2	2.99763	2.96819	27381	209852179	13362	2788	675479
Sample_YPB2	3.05706	3.00736	27036	221306912	16751	2816	737555
Sample_YPC2	3.14535	3.08502	25024	200729173	18339	2715	737509
Sample_YPA3	2.76192	2.71064	15389	132482099	26648	2711	1069624
Sample_YPB3	3.05539	2.98992	12669	115148665	19915	3078	565635
Sample_YPC3	2.94181	2.88929	14154	123165158	22673	2825	753688
Sample_YPA4	2.81988	2.78296	17413	143437510	21520	2718	909276
Sample_YPB4	3.12534	3.07533	13033	128053043	26370	3122	834859
Sample_YPC4	3.49460	3.43224	22079	173100739	15696	2750	909276
Sample_YPA5	3.04797	3.01843	20647	207058421	28927	3089	464348
Sample_YPB5	2.71121	2.66974	19458	190709821	23429	3211	381643
Sample_YPC5	3.12284	3.07066	21130	199447533	24951	3007	493609
Sample_YPA6	3.42441	3.37704	21643	166265624	14104	2753	572825
Sample_YPB6	2.83400	2.80178	11139	121968998	38258	3324	799050
Sample_YPC6	2.85756	2.80396	21125	166053880	14513	2818	575344
Sample_YPA7	3.29505	3.25941	21576	204108473	23590	3073	612517
Sample_YPB7	3.49932	3.42928	22680	183688045	15148	2880	560875
Sample_YPC7	3.02865	2.97722	21444	158535129	13681	2674	909276
Sample_SCA55	6.16560	6.10961	28740	221031419	14530	2739	778371
Sample_SCB55	6.18423	6.10581	34915	248664164	12563	2612	778381
Sample_SCC55	4.59738	4.53112	22724	164798599	13877	2596	778381
Sample_SCA58	6.73700	6.67523	34103	287645695	18222	2866	921518
Sample_SCB58	6.27298	6.20058	40618	345711137	16863	2981	691450
Sample_SCC58	5.57315	5.52080	31987	264411708	16458	2873	477837
Sample_SCA59	4.50208	4.46951	39351	306792532	14609	2784	743981
Sample_SCB59	5.18800	5.12868	39530	312561966	15320	2795	832455
Sample_SCC59	2.77841	2.75937	27882	201502233	12410	2682	411272
Sample_SCA60	5.43013	5.38058	34550	264425856	14829	2727	416625
Sample_SCB60	6.59234	6.44059	35138	294459219	17422	2886	552690
Sample_SCC60	5.29149	5.15822	32287	252608786	15435	2773	493566
Sample_SCA61	6.11455	6.02166	21170	213322483	23793	3282	675371
Sample_SCB61	6.26271	6.20607	20989	217119614	27117	3238	688370
Sample_SCC61	5.86879	5.81642	16634	191653159	33008	3489	688153
Sample_SCA64	4.63295	4.59869	18803	210123166	32386	3453	739522
Sample_SCB64	5.54598	5.48302	25604	241819901	21843	3133	749244
Sample_SCC64	4.91693	4.85065	16469	175032706	32259	3246	558487
Sample_SCA65	6.31261	6.24944	22889	204311165	22517	2892	519561
Sample_SCB65	5.10966	5.05709	20735	187572756	22464	2936	533258
Sample_SCC65	3.97393	3.93438	18219	163902622	25447	2847	539740
Sample_SCA66	5.29863	5.24365	26010	221002250	18464	2887	648588
Sample_SCB66	5.22126	5.13344	25396	243699695	21512	3181	606694

Sample_SCC66	4.97205	4.92579	29311	250032514	17130	2944	602763
Sample_SCA67	5.84483	5.78412	38352	258620913	10501	2615	609133
Sample_SCB67	6.21176	6.12640	45018	324113458	11959	2723	649447
Sample_SCC67	4.60453	4.56204	38172	260530148	10503	2650	495751
Sample_SCA68	5.00330	4.89207	21601	176219759	18326	2784	416167
Sample_SCB68	5.71359	5.67924	18925	152898609	16945	2830	372379
Sample_SCC68	4.65627	4.61367	22593	170921862	13404	2773	397531
Sample_SCA69	6.03514	5.93103	26332	231723996	20322	2935	424840
Sample_SCB69	6.43796	6.34207	28539	264111796	22415	3000	467102
Sample_SCC69	6.24317	6.17781	22552	197598242	23505	2833	392926
Sample_SCA70	5.17107	5.10476	40010	296033288	13960	2653	319191
Sample_SCB70	5.10562	5.06185	31566	252977971	15504	2812	437715
Sample_SCC70	4.17968	4.13227	31625	256171187	16791	2813	772168
Sample_LCA09	5.86811	5.82479	26496	246121050	20855	3109	566805
Sample_LCB09	5.40434	5.34145	21834	204018319	19428	3203	516264
Sample_LCC09	6.99543	6.93432	27893	264542425	22013	3122	784595
Sample_LCA10	3.94486	3.89814	12937	161816777	35798	3982	704097
Sample_LCB10	5.37054	5.30861	23480	221129530	22948	3067	566783
Sample_LCC10	5.01276	4.68807	19876	195740550	25270	3099	566785
Sample_LCA11	5.88014	5.81806	29219	262969582	21038	2965	612280
Sample_LCB11	3.53140	3.50877	21492	188671639	19670	2916	603123
Sample_LCC11	4.56206	4.53279	23889	174512500	11767	2763	432633
Sample_LCA12	5.76344	5.71106	38729	336269038	18831	2935	502089
Sample_LCB12	5.61032	5.55313	37675	306108709	15518	2879	714828
Sample_LCC12	5.84876	5.79452	32642	239502485	14590	2623	501974
Sample_LCA13	5.57239	5.43423	29852	257631488	20535	2832	606829
Sample_LCB13	5.11191	5.07214	28908	258953916	21726	2944	768510
Sample_LCC13	4.80126	4.76368	29142	257353356	20423	2948	873667
Sample_LCA14	4.81958	4.67082	12849	149951394	36761	3540	841953
Sample_LCB14	5.70649	5.66741	11910	153017382	43384	3860	931079
Sample_LCC14	6.06208	6.01939	14115	159568629	33985	3460	1135479
Sample_LCA15	4.88008	4.85734	13736	137522989	27703	3114	595262
Sample_LCB15	5.72317	5.68147	9797	96944063	24365	3177	435789
Sample_LCC15	3.77199	3.75584	14297	62676084	4662	2332	138378
Sample_LCA16	5.19521	5.14576	21549	183421882	19672	2840	532124
Sample_LCB16	5.79259	5.75604	13978	136109476	21362	3241	680236
Sample_LCC16	5.49451	5.45521	10363	99334334	29678	3014	683062
Sample_LCA17	4.86522	4.84350	25575	210900324	16688	2893	372928
Sample_LCB17	5.78310	5.74656	31978	282138117	22896	2884	498784
Sample_LCC17	7.51697	7.44964	34214	286350295	17088	2889	570219
Sample_LCA19	5.46298	5.43028	17550	170136065	23547	3155	687809
Sample_LCB19	5.83150	5.78767	17373	173794512	28750	3070	655697
Sample_LCC19	5.64435	5.61510	18153	175547771	23148	3214	615950
Sample_LCA20	5.45989	5.40579	18308	187706392	22985	3417	596940
Sample_LCB20	4.89808	4.87453	21826	178289311	17640	2809	597183
Sample_LCC20	5.57042	5.52573	21973	187344340	18953	2856	625817
Sample_LCA21	5.02321	4.97515	27085	225652776	18675	2814	393533
Sample_LCB21	4.86050	4.82928	27447	230255405	18526	2840	533517
Sample_LCC21	3.55386	3.52532	27854	194542321	11096	2656	650607
Sample_LCA22	5.89769	5.85144	27894	236031107	17020	2946	663631
Sample_LCB22	6.13303	6.08032	27951	242383383	18558	2941	549211
Sample_LCC22	5.80640	5.76945	26556	233359388	19274	2985	549211
Sample_LCA23	5.66367	5.61054	23844	211270894	19282	2989	649551
Sample_LCB23	5.91758	5.87234	25059	219028742	18734	3000	673543
Sample_LCC23	5.49954	5.46784	27438	215171213	14549	2835	649890
Sample_LCA24	2.06275	2.05399	11744	107543484	22137	3009	464477

Sample_LCB24	5.67757	5.64406	20282	179152636	20649	2966	389762
Sample_LCC24	3.50606	3.48863	18111	159392974	18871	2984	472566
Sample_LCA25	5.30054	5.25982	14284	135142719	23713	3071	736183
Sample_LCB25	7.54966	7.48044	14293	157918983	29582	3554	736183
Sample_LCC25	5.74066	5.69490	15394	144118870	23151	3132	736183

Supplementary Table 3 Genomic information of 7538 high-quality metagenome-assembled genomes (MAGs)

MAG ID	Completeness (%)	Contamination (%)	Number of contigs
Sample_LCA09.Cluster12306	92.41	4.494	7
Sample_LCA09.Cluster1389cbin.1	94.35	0.806	105
Sample_LCA09.Cluster103	89.76	4.026	55
Sample_LCA09.Cluster29cbin.1	86.25	3.467	244
Sample_LCA09.Cluster9849cbin.1	85.76	2.908	334
Sample_LCA09.Cluster3712cbin.1	97.04	1.265	61
Sample_LCA09.Cluster3578	97.31	0.000	38
Sample_LCA09.mb.109	95.30	2.684	137
Sample_LCA09.mb.101cbin.1	93.95	4.138	74
Sample_LCA09.Cluster1791	94.96	0.000	11
Sample_LCA09.mb.39cbin.1	88.50	1.148	245
Sample_LCA09.mb.16cbin.1	97.31	0.826	147
Sample_LCA09.Cluster770cbin.1	94.93	2.003	334
Sample_LCA09.Cluster11946cbin.1	99.47	0.000	23
Sample_LCA09.mb.10	93.19	0.680	212
Sample_LCA09.Cluster9301	99.32	0.671	16
Sample_LCA09.mb.2cbin.1	97.76	0.671	88
Sample_LCA09.Cluster11194cbin.1	97.98	0.041	54
Sample_LCA09.Cluster961	98.18	0.303	22
Sample_LCA09.mb.53cbin.1	92.93	2.013	52
Sample_LCA09.mb.127	87.07	0.566	72
Sample_LCA09.Cluster74cbin.1	97.69	1.900	43
Sample_LCA09.mb.73	96.64	1.565	183
Sample_LCA09.mb.103	98.00	3.743	65
Sample_LCA09.mb.15cbin.1	96.93	2.013	29
Sample_LCA09.mb.45	90.25	2.684	306
Sample_LCA09.mb.75	96.81	2.684	91
Sample_LCA09.mb.17cbin.1	95.89	3.424	28
Sample_LCA09.mb.106cbin.1	99.27	3.846	32
Sample_LCA09.mb.66	84.84	2.531	183
Sample_LCA09.mb.79	97.76	0.000	73
Sample_LCA09.mb.82cbin.1	80.44	0.335	47
Sample_LCA09.Cluster3917	98.58	3.923	43
Sample_LCA09.mb.31cbin.1	98.65	0.000	47
Sample_LCA09.mb.99	86.57	2.726	119
Sample_LCA09.Cluster1997	97.69	0.128	57
Sample_LCA09.mb.64	94.47	2.073	141
Sample_LCA09.Cluster2032	96.23	0.537	222
Sample_LCA09.Cluster6035cbin.1	97.88	1.610	75
Sample_LCA09.Cluster9790cbin.1	92.48	1.127	123
Sample_LCA09.mb.86cbin.1	95.75	3.955	114
Sample_LCA09.mb.63	99.36	0.316	218
Sample_LCA09.mb.98cbin.1	98.79	0.724	86
Sample_LCA10.mb.27cbin.1	96.49	4.465	415
Sample_LCA10.Cluster26cbin.1	80.06	0.671	281
Sample_LCA10.Cluster330cbin.1	97.65	0.389	125
Sample_LCA10.Cluster54	100.00	0.000	17
Sample_LCA10.mb.10	92.61	2.013	29
Sample_LCA10.Cluster1853cbin.1	98.31	1.054	73
Sample_LCA10.Cluster2071cbin.1	99.18	0.000	21
Sample_LCA10.Cluster140	97.98	0.713	28
Sample_LCA10.mb.18	94.63	1.677	63
Sample_LCA10.mb.32cbin.1	98.73	0.021	112
Sample_LCA10.mb.1	97.95	0.680	242

Sample_LCA10.Cluster1516cbin.1	93.91	0.000	11
Sample_LCA10.mb.60cbin.1	87.90	0.806	36
Sample_LCA10.Cluster160cbin.1	92.15	1.041	264
Sample_LCA10.mb.51cbin.1	91.82	0.961	32
Sample_LCA10.mb.52	88.83	0.226	232
Sample_LCA10.mb.47	94.23	0.026	44
Sample_LCA10.Cluster63	99.53	0.000	21
Sample_LCA10.mb.55cbin.1	96.31	0.000	27
Sample_LCA10.Cluster332	97.24	0.961	22
Sample_LCA10.mb.39cbin.1	94.06	3.735	154
Sample_LCA10.mb.37cbin.1	99.36	0.105	191
Sample_LCA10.mb.44cbin.1	99.41	0.000	69
Sample_LCA10.Cluster399	99.79	1.923	23
Sample_LCA10.Cluster2087	97.04	1.075	252
Sample_LCA10.Cluster1830cbin.1	98.11	0.000	240
Sample_LCA10.Cluster1398cbin.1	98.71	2.848	114
Sample_LCA10.mb.48	81.82	3.295	127
Sample_LCA10.Cluster2295cbin.1	99.03	0.384	63
Sample_LCA10.mb.67	99.07	0.037	62
Sample_LCA11.mb.110cbin.1	87.60	1.612	22
Sample_LCA11.Cluster5845	98.52	0.000	13
Sample_LCA11.Cluster295	92.61	1.901	45
Sample_LCA11.mb.27	90.91	0.000	81
Sample_LCA11.mb.4	92.41	2.247	49
Sample_LCA11.Cluster7780cbin.1	94.14	1.796	217
Sample_LCA11.Cluster4752	81.10	2.348	15
Sample_LCA11.Cluster10191	98.65	1.006	18
Sample_LCA11.Cluster7430	93.95	1.342	31
Sample_LCA11.Cluster11756cbin.1	97.31	0.000	21
Sample_LCA11.Cluster2198	97.27	0.000	32
Sample_LCA11.Cluster12733cbin.1	82.77	2.784	417
Sample_LCA11.mb.24cbin.1	88.29	3.243	87
Sample_LCA11.mb.113	100.00	0.000	26
Sample_LCA11.mb.22	83.21	1.342	268
Sample_LCA11.mb.74	91.57	4.131	43
Sample_LCA11.Cluster9799cbin.1	93.27	0.000	245
Sample_LCA11.Cluster13155	100.00	0.943	80
Sample_LCA11.mb.37	83.44	1.677	208
Sample_LCA11.mb.39cbin.1	94.94	4.496	185
Sample_LCA11.mb.60cbin.1	82.38	1.367	176
Sample_LCA11.mb.88	80.02	1.342	22
Sample_LCA11.Cluster6942cbin.1	95.34	0.537	468
Sample_LCA11.Cluster9181cbin.1	97.15	1.265	461
Sample_LCA11.mb.96	92.95	1.006	44
Sample_LCA11.mb.44	95.49	0.335	83
Sample_LCA11.Cluster6646	95.64	0.000	44
Sample_LCA11.mb.62cbin.1	87.34	1.886	197
Sample_LCA11.mb.89cbin.1	97.04	2.109	240
Sample_LCA11.mb.85cbin.1	87.02	0.671	60
Sample_LCA11.Cluster9182cbin.1	97.83	0.897	94
Sample_LCA11.mb.29cbin.1	89.26	0.991	74
Sample_LCA11.mb.105cbin.1	99.24	0.188	72
Sample_LCA11.mb.81cbin.1	98.85	4.967	112
Sample_LCA11.mb.55	88.14	1.951	184
Sample_LCA11.Cluster4813cbin.1	98.67	0.192	57
Sample_LCA11.mb.75	96.47	0.660	303
Sample_LCA12.Cluster17512	85.81	0.111	108
Sample_LCA12.Cluster10145	96.64	0.000	23

Sample_LCA12.Cluster16605	87.24	0.000	44
Sample_LCA12.Cluster6897cbin.1	96.37	0.000	16
Sample_LCA12.mb.139cbin.1	91.29	1.685	27
Sample_LCA12.mb.111	99.30	1.165	95
Sample_LCA12.mb.136	98.38	0.000	41
Sample_LCA12.Cluster4404cbin.1	95.94	0.371	41
Sample_LCA12.mb.3	91.57	2.247	26
Sample_LCA12.Cluster12909	84.56	0.000	38
Sample_LCA12.Cluster3695cbin.1	81.25	0.480	34
Sample_LCA12.Cluster4266cbin.1	99.00	0.000	40
Sample_LCA12.mb.116	92.44	3.243	95
Sample_LCA12.Cluster10369	97.09	0.000	26
Sample_LCA12.Cluster3601	96.97	0.000	13
Sample_LCA12.Cluster10cbin.1	94.40	0.776	305
Sample_LCA12.Cluster4880cbin.1	92.57	0.671	27
Sample_LCA12.Cluster9314	98.99	0.000	45
Sample_LCA12.Cluster10866	84.62	0.000	21
Sample_LCA12.Cluster15	98.69	0.591	94
Sample_LCA12.Cluster717	97.95	1.020	86
Sample_LCA12.mb.49	90.99	2.419	87
Sample_LCA12.mb.22	86.68	3.467	149
Sample_LCA12.mb.112	98.07	1.442	20
Sample_LCA12.Cluster13856cbin.1	92.05	4.856	513
Sample_LCA12.mb.124cbin.1	94.55	2.040	297
Sample_LCA12.mb.129cbin.1	96.37	0.188	25
Sample_LCA12.mb.58cbin.1	97.84	0.000	53
Sample_LCA12.mb.110	87.34	0.099	72
Sample_LCA12.Cluster8154cbin.1	97.97	2.646	95
Sample_LCA12.mb.43	89.53	2.013	94
Sample_LCA12.mb.52cbin.1	88.36	1.845	100
Sample_LCA12.mb.4	96.04	0.894	158
Sample_LCA12.Cluster7146cbin.1	96.77	1.254	313
Sample_LCA12.mb.88	90.82	2.013	28
Sample_LCA12.mb.83	88.43	2.796	158
Sample_LCA12.mb.67	89.74	0.000	38
Sample_LCA12.mb.25cbin.1	83.04	0.000	58
Sample_LCA12.mb.95	90.60	0.000	35
Sample_LCA12.mb.85	95.65	0.640	64
Sample_LCA12.mb.65cbin.1	99.19	1.612	63
Sample_LCA12.mb.34	80.12	0.290	80
Sample_LCA12.mb.46	95.50	0.721	63
Sample_LCA12.mb.6	98.65	0.335	44
Sample_LCA12.mb.78	92.17	1.082	110
Sample_LCA12.mb.79cbin.1	98.88	0.370	42
Sample_LCA12.mb.143	94.72	2.756	340
Sample_LCA12.mb.76	96.09	0.671	74
Sample_LCA12.mb.97	98.23	0.961	38
Sample_LCA12.mb.69	97.76	2.560	60
Sample_LCA12.Cluster3297cbin.1	96.86	4.787	288
Sample_LCA12.mb.42cbin.1	98.33	0.128	167
Sample_LCA12.mb.47cbin.1	96.82	4.615	111
Sample_LCA12.Cluster256cbin.1	94.52	0.827	125
Sample_LCA13.Cluster6321cbin.1	95.69	1.075	43
Sample_LCA13.Cluster450cbin.1	80.01	0.732	55
Sample_LCA13.Cluster63cbin.1	86.88	1.677	41
Sample_LCA13.mb.105	90.62	2.848	362
Sample_LCA13.mb.14cbin.1	84.67	4.215	252
Sample_LCA13.Cluster97cbin.1	87.45	3.225	192

Sample_LCA13.Cluster5880	93.99	0.748	81
Sample_LCA13.Cluster4024	94.26	1.342	9
Sample_LCA13.mb.118	86.40	1.510	110
Sample_LCA13.mb.110cbin.1	87.71	1.400	359
Sample_LCA13.mb.27	85.19	3.914	211
Sample_LCA13.Cluster11949	99.32	1.006	27
Sample_LCA13.Cluster5089cbin.1	96.26	1.677	42
Sample_LCA13.Cluster9916cbin.1	97.98	0.335	45
Sample_LCA13.Cluster28	96.92	0.591	71
Sample_LCA13.mb.66	97.09	0.000	39
Sample_LCA13.mb.53	82.52	1.565	290
Sample_LCA13.mb.19	94.63	0.559	153
Sample_LCA13.Cluster10737	96.57	0.000	28
Sample_LCA13.Cluster57	99.94	0.000	34
Sample_LCA13.mb.4	87.11	3.766	374
Sample_LCA13.mb.22cbin.1	95.11	0.961	107
Sample_LCA13.mb.36	99.20	0.000	92
Sample_LCA13.mb.61	89.93	0.000	37
Sample_LCA13.Cluster3740cbin.1	97.54	4.310	228
Sample_LCA13.Cluster9471	96.28	0.362	181
Sample_LCA13.mb.74	89.55	1.510	59
Sample_LCA13.mb.107	97.31	2.684	60
Sample_LCA13.mb.116	98.24	0.000	58
Sample_LCA13.mb.86	95.30	0.671	45
Sample_LCA13.mb.63	91.82	4.195	255
Sample_LCA13.mb.109	98.49	0.377	44
Sample_LCA13.mb.65	86.06	1.360	372
Sample_LCA13.mb.78	99.03	1.698	83
Sample_LCA13.mb.90cbin.1	90.36	4.823	135
Sample_LCA13.Cluster8570	98.65	0.384	33
Sample_LCA13.Cluster12558cbin.1	96.52	1.321	106
Sample_LCA13.mb.102cbin.1	95.73	2.537	186
Sample_LCA13.mb.44	97.85	1.538	150
Sample_LCA13.mb.9cbin.1	99.46	0.000	67
Sample_LCA13.mb.67	98.14	1.115	30
Sample_LCA13.Cluster1780cbin.1	97.17	2.109	320
Sample_LCA13.mb.100cbin.1	96.98	0.939	173
Sample_LCA14.Cluster1346	93.95	0.832	16
Sample_LCA14.Cluster2622	99.18	0.000	25
Sample_LCA14.Cluster1004	95.30	0.000	29
Sample_LCA14.Cluster815cbin.1	87.87	4.026	80
Sample_LCA14.mb.51	94.63	0.020	32
Sample_LCA14.mb.61cbin.1	97.31	0.000	56
Sample_LCA14.Cluster4930	97.90	0.000	44
Sample_LCA14.Cluster1036cbin.1	99.18	0.546	63
Sample_LCA14.Cluster351cbin.1	98.80	0.000	70
Sample_LCA14.Cluster5	99.65	0.460	75
Sample_LCA14.mb.67	92.51	0.680	213
Sample_LCA14.mb.22	99.22	0.000	9
Sample_LCA14.Cluster964cbin.1	87.49	0.065	568
Sample_LCA14.Cluster308cbin.1	95.23	0.226	38
Sample_LCA14.mb.2	94.47	0.704	170
Sample_LCA14.mb.26	83.63	3.990	99
Sample_LCA14.Cluster362	98.79	0.480	20
Sample_LCA14.mb.14	92.39	0.000	57
Sample_LCA14.Cluster1967cbin.1	96.65	0.000	35
Sample_LCA14.mb.15cbin.1	92.56	0.000	34
Sample_LCA14.mb.65cbin.1	97.36	0.705	226

Sample_LCA14.mb.12	93.70	4.162	531
Sample_LCA14.mb.60	97.06	2.824	27
Sample_LCA15.mb.47	94.62	0.000	70
Sample_LCA15.mb.39cbin.1	85.43	2.684	260
Sample_LCA15.mb.46	99.32	0.671	32
Sample_LCA15.Cluster1357cbin.1	94.51	0.632	35
Sample_LCA15.mb.24	98.10	0.000	80
Sample_LCA15.mb.53cbin.1	82.20	0.680	353
Sample_LCA15.mb.12	97.31	0.000	10
Sample_LCA15.Cluster1569	91.61	1.497	54
Sample_LCA15.mb.20cbin.1	97.34	0.680	268
Sample_LCA15.Cluster3639	99.32	0.671	15
Sample_LCA15.Cluster2898cbin.1	88.62	1.971	393
Sample_LCA15.Cluster41cbin.1	89.65	1.670	295
Sample_LCA15.mb.1	84.03	2.674	112
Sample_LCA15.mb.45cbin.1	98.48	0.000	39
Sample_LCA15.Cluster5216cbin.1	97.31	0.000	30
Sample_LCA15.mb.18cbin.1	90.69	2.531	126
Sample_LCA15.Cluster3291	97.74	0.000	63
Sample_LCA15.mb.48	89.88	1.107	101
Sample_LCA15.mb.69	90.56	1.509	119
Sample_LCA15.mb.59	88.40	0.724	40
Sample_LCA15.Cluster1253cbin.1	95.92	0.867	75
Sample_LCA15.mb.33	95.98	1.486	152
Sample_LCA15.Cluster753cbin.1	97.73	1.981	209
Sample_LCA15.mb.61cbin.1	96.87	0.409	45
Sample_LCA15.Cluster35	89.05	0.768	51
Sample_LCA16.Cluster2110	96.77	0.000	47
Sample_LCA16.mb.26	93.93	1.342	37
Sample_LCA16.mb.32cbin.1	99.30	1.398	136
Sample_LCA16.Cluster1219	89.23	2.027	363
Sample_LCA16.Cluster252cbin.1	93.91	2.702	77
Sample_LCA16.mb.13	96.13	2.460	54
Sample_LCA16.Cluster6403cbin.1	92.56	1.789	234
Sample_LCA16.mb.56	96.97	0.335	99
Sample_LCA16.Cluster7169	97.98	0.671	22
Sample_LCA16.mb.14cbin.1	85.43	0.335	263
Sample_LCA16.mb.29	98.97	0.984	108
Sample_LCA16.mb.61	97.31	0.000	15
Sample_LCA16.mb.75cbin.1	98.14	0.509	59
Sample_LCA16.mb.6	99.32	0.000	26
Sample_LCA16.Cluster2784cbin.1	81.76	3.763	695
Sample_LCA16.Cluster4602	99.24	0.000	27
Sample_LCA16.Cluster3983cbin.1	94.05	0.185	27
Sample_LCA16.Cluster7377	99.62	0.187	26
Sample_LCA16.mb.9cbin.1	99.51	0.724	67
Sample_LCA16.Cluster3119cbin.1	94.62	2.150	376
Sample_LCA16.mb.12cbin.1	89.50	1.923	576
Sample_LCA16.Cluster777cbin.1	99.31	1.128	104
Sample_LCA16.mb.1cbin.1	98.75	0.551	53
Sample_LCA17.mb.41cbin.1	97.84	0.000	97
Sample_LCA17.mb.49	80.48	1.342	242
Sample_LCA17.Cluster8456cbin.1	97.98	0.518	95
Sample_LCA17.Cluster550cbin.1	92.61	4.316	88
Sample_LCA17.Cluster3336cbin.1	84.59	0.925	216
Sample_LCA17.Cluster2742cbin.1	92.42	0.000	58
Sample_LCA17.Cluster8642cbin.1	92.61	0.671	15
Sample_LCA17.mb.88	92.41	1.123	41

Sample_LCA17.mb.70	97.90	1.165	104
Sample_LCA17.mb.62cbin.1	85.41	0.447	278
Sample_LCA17.mb.56cbin.1	91.55	2.348	233
Sample_LCA17.Cluster10970cbin.1	93.30	4.107	423
Sample_LCA17.mb.60cbin.1	89.66	0.671	40
Sample_LCA17.mb.46	83.86	1.442	427
Sample_LCA17.mb.96	97.58	0.000	44
Sample_LCA17.mb.22cbin.1	96.90	1.303	175
Sample_LCA17.mb.38cbin.1	90.86	2.884	56
Sample_LCA17.Cluster49cbin.1	88.99	0.966	437
Sample_LCA17.mb.54cbin.1	97.58	3.019	69
Sample_LCA17.mb.58	98.79	1.442	84
Sample_LCA17.Cluster8124cbin.1	99.51	2.608	152
Sample_LCA17.Cluster8958cbin.1	97.35	3.207	198
Sample_LCA17.mb.61	87.92	0.000	41
Sample_LCA17.mb.24	97.44	2.798	53
Sample_LCA17.mb.76cbin.1	97.06	0.370	82
Sample_LCA17.mb.79cbin.1	90.82	0.671	243
Sample_LCA17.mb.73	91.94	1.342	64
Sample_LCA17.mb.97	87.01	2.016	270
Sample_LCA17.mb.80	92.61	0.335	46
Sample_LCA17.mb.94	96.63	1.415	35
Sample_LCA17.mb.9	97.27	0.000	22
Sample_LCA17.mb.50cbin.1	95.16	0.000	154
Sample_LCA17.mb.74cbin.1	89.12	3.713	174
Sample_LCA17.mb.3cbin.1	97.88	2.115	264
Sample_LCA17.mb.40	94.87	1.526	240
Sample_LCA17.Cluster534cbin.1	84.22	1.967	668
Sample_LCA19.Cluster6802cbin.1	86.16	1.123	147
Sample_LCA19.Cluster1769cbin.1	94.62	0.000	63
Sample_LCA19.Cluster1748	81.85	1.898	30
Sample_LCA19.mb.77cbin.1	90.26	0.000	23
Sample_LCA19.Cluster1073	94.96	0.000	5
Sample_LCA19.Cluster674cbin.1	94.39	3.365	36
Sample_LCA19.mb.41cbin.1	87.18	3.846	375
Sample_LCA19.mb.50cbin.1	80.47	0.403	347
Sample_LCA19.Cluster4112	99.32	3.355	17
Sample_LCA19.Cluster201cbin.1	94.89	2.040	239
Sample_LCA19.Cluster314cbin.1	92.50	1.770	243
Sample_LCA19.mb.60	93.98	0.000	70
Sample_LCA19.mb.88	89.21	2.777	129
Sample_LCA19.Cluster6064	97.31	0.000	26
Sample_LCA19.mb.39cbin.1	97.65	0.000	33
Sample_LCA19.Cluster4036cbin.1	97.09	0.966	100
Sample_LCA19.mb.75cbin.1	91.21	0.340	109
Sample_LCA19.mb.85	96.34	0.000	123
Sample_LCA19.mb.47	93.68	0.929	78
Sample_LCA19.mb.58cbin.1	94.72	3.415	200
Sample_LCA19.mb.48	98.11	1.257	186
Sample_LCA19.Cluster1508	86.02	0.371	67
Sample_LCA19.mb.52	85.74	4.384	253
Sample_LCA19.mb.23cbin.1	90.76	0.833	438
Sample_LCA19.mb.36	90.36	0.693	43
Sample_LCA20.mb.10cbin.1	91.57	2.247	11
Sample_LCA20.mb.30	90.66	0.000	29
Sample_LCA20.mb.57	91.57	2.247	19
Sample_LCA20.Cluster427cbin.1	95.45	2.564	91
Sample_LCA20.mb.34cbin.1	93.73	0.894	41

Sample_LCA20.mb.12	97.31	0.000	26
Sample_LCA20.Cluster408	90.60	0.671	82
Sample_LCA20.mb.104	83.27	0.671	97
Sample_LCA20.Cluster1109cbin.1	88.03	0.000	113
Sample_LCA20.mb.59	88.96	3.164	262
Sample_LCA20.Cluster2172cbin.1	93.28	3.355	25
Sample_LCA20.Cluster6765	97.09	0.000	22
Sample_LCA20.Cluster8336cbin.1	89.26	1.006	50
Sample_LCA20.Cluster5171	95.63	0.335	17
Sample_LCA20.mb.1cbin.1	92.85	1.020	224
Sample_LCA20.mb.6cbin.1	91.94	0.000	21
Sample_LCA20.mb.36	86.81	0.838	206
Sample_LCA20.Cluster6794cbin.1	99.32	2.013	26
Sample_LCA20.Cluster2547	95.26	4.362	18
Sample_LCA20.mb.43	99.51	0.961	30
Sample_LCA20.mb.44cbin.1	95.91	0.000	30
Sample_LCA20.Cluster133cbin.1	96.15	1.682	63
Sample_LCA20.mb.13	97.31	0.671	37
Sample_LCA20.mb.7	92.46	1.013	47
Sample_LCA20.mb.75cbin.1	100.00	0.000	48
Sample_LCA20.mb.98	87.57	2.203	428
Sample_LCA20.mb.102	99.62	0.000	19
Sample_LCA20.mb.56	82.72	2.168	184
Sample_LCA20.mb.66cbin.1	85.01	1.881	591
Sample_LCA20.Cluster4859cbin.1	96.04	1.730	360
Sample_LCA20.Cluster2539cbin.1	99.37	0.210	56
Sample_LCA20.mb.55	99.35	0.079	44
Sample_LCA21.mb.26cbin.1	94.35	1.075	74
Sample_LCA21.mb.108	91.92	2.034	273
Sample_LCA21.Cluster3243cbin.1	88.14	0.074	45
Sample_LCA21.Cluster461cbin.1	89.70	4.697	114
Sample_LCA21.mb.35	89.93	0.838	46
Sample_LCA21.mb.10	89.87	1.898	191
Sample_LCA21.Cluster4740	94.30	1.793	46
Sample_LCA21.Cluster3612cbin.1	91.77	0.021	84
Sample_LCA21.Cluster4672cbin.1	98.78	1.497	75
Sample_LCA21.mb.79	91.92	1.342	34
Sample_LCA21.mb.12	99.32	0.671	33
Sample_LCA21.mb.51	92.75	1.465	36
Sample_LCA21.mb.107	95.74	0.000	31
Sample_LCA21.mb.13	100.00	0.316	120
Sample_LCA21.mb.88cbin.1	91.72	1.342	79
Sample_LCA21.Cluster4089cbin.1	93.54	0.724	49
Sample_LCA21.mb.71cbin.1	82.90	0.671	370
Sample_LCA21.mb.61cbin.1	93.58	0.335	24
Sample_LCA21.mb.93	88.81	0.707	96
Sample_LCA21.mb.103cbin.1	93.67	0.632	66
Sample_LCA21.mb.59cbin.1	96.97	2.181	119
Sample_LCA21.mb.98	95.80	1.510	50
Sample_LCA21.mb.21	97.46	0.105	76
Sample_LCA21.mb.9	95.49	2.995	45
Sample_LCA21.mb.17cbin.1	89.58	3.915	133
Sample_LCA21.mb.66cbin.1	98.79	0.737	37
Sample_LCA21.mb.95cbin.1	83.06	1.578	259
Sample_LCA21.mb.90	100.00	1.499	176
Sample_LCA21.mb.99cbin.1	98.55	1.610	67
Sample_LCA21.mb.112cbin.1	99.42	0.961	150
Sample_LCA21.mb.69	94.66	4.115	206

Sample_LCA21.mb.67cbin.1	89.73	4.275	366
Sample_LCA21.Cluster5531cbin.1	98.95	2.581	260
Sample_LCA22.Cluster2200	96.77	0.161	33
Sample_LCA22.mb.26	91.85	1.123	88
Sample_LCA22.Cluster2096cbin.1	93.98	2.900	245
Sample_LCA22.Cluster321	89.93	0.335	53
Sample_LCA22.mb.21	95.63	0.335	113
Sample_LCA22.Cluster251	100.00	0.921	30
Sample_LCA22.mb.29	93.62	0.671	37
Sample_LCA22.Cluster3338	99.40	0.898	40
Sample_LCA22.Cluster5574	95.97	2.013	10
Sample_LCA22.mb.79	88.88	0.854	35
Sample_LCA22.Cluster227	97.95	0.000	30
Sample_LCA22.mb.74	80.08	2.848	493
Sample_LCA22.mb.93	94.35	1.478	130
Sample_LCA22.Cluster274cbin.1	99.44	0.795	80
Sample_LCA22.mb.73	90.26	1.067	24
Sample_LCA22.Cluster119cbin.1	96.75	1.250	84
Sample_LCA22.mb.58cbin.1	94.85	0.000	144
Sample_LCA22.mb.20cbin.1	81.20	1.510	23
Sample_LCA22.mb.92	93.15	1.165	286
Sample_LCA22.mb.45	91.78	2.173	64
Sample_LCA22.mb.36cbin.1	96.64	0.335	40
Sample_LCA22.mb.81cbin.1	96.07	1.201	84
Sample_LCA22.mb.44	95.65	1.690	56
Sample_LCA22.Cluster5426cbin.1	87.62	1.321	102
Sample_LCA22.Cluster8517cbin.1	98.68	3.091	104
Sample_LCA22.mb.77	95.97	0.000	14
Sample_LCA22.Cluster3734cbin.1	92.22	0.537	225
Sample_LCA22.mb.51	93.45	2.215	263
Sample_LCA22.mb.33cbin.1	98.07	0.128	89
Sample_LCA22.mb.22cbin.1	98.17	2.692	223
Sample_LCA22.mb.91cbin.1	90.38	3.141	192
Sample_LCA22.mb.15cbin.1	99.29	0.037	54
Sample_LCA23.mb.29cbin.1	80.48	0.894	343
Sample_LCA23.mb.11cbin.1	90.60	0.692	68
Sample_LCA23.Cluster2542	82.77	0.000	40
Sample_LCA23.Cluster263	100.00	0.806	29
Sample_LCA23.mb.33cbin.1	95.80	3.562	199
Sample_LCA23.Cluster3080cbin.1	98.40	0.898	104
Sample_LCA23.mb.28	91.87	2.215	138
Sample_LCA23.mb.54	90.60	1.677	48
Sample_LCA23.Cluster406	90.95	0.680	238
Sample_LCA23.Cluster8070	99.32	1.006	20
Sample_LCA23.mb.59	95.34	3.355	334
Sample_LCA23.Cluster213cbin.1	90.43	0.199	285
Sample_LCA23.Cluster5816cbin.1	98.65	0.755	24
Sample_LCA23.mb.53	98.55	0.961	30
Sample_LCA23.Cluster5538cbin.1	92.08	0.316	87
Sample_LCA23.Cluster8409cbin.1	96.08	0.558	132
Sample_LCA23.mb.67cbin.1	97.98	0.335	16
Sample_LCA23.mb.68	97.19	4.166	183
Sample_LCA23.Cluster3820cbin.1	91.27	3.188	162
Sample_LCA23.Cluster4195cbin.1	82.00	3.225	621
Sample_LCA23.mb.86	87.70	0.000	248
Sample_LCA23.mb.4cbin.1	96.27	0.621	85
Sample_LCA23.mb.70cbin.1	97.54	0.377	50
Sample_LCA23.mb.30	98.55	0.966	113

Sample_LCA23.mb.84	97.66	3.450	46
Sample_LCA23.mb.76cbin.1	88.32	2.721	238
Sample_LCA23.Cluster6963cbin.1	91.82	0.512	329
Sample_LCA23.mb.74cbin.1	94.69	2.153	249
Sample_LCA23.mb.40cbin.1	99.06	1.282	93
Sample_LCA23.mb.85cbin.1	99.45	1.146	195
Sample_LCA24.mb.24	83.39	3.571	106
Sample_LCA24.Cluster446cbin.1	90.93	0.000	292
Sample_LCA24.mb.16	97.04	1.728	116
Sample_LCA24.Cluster4405cbin.1	88.41	0.542	293
Sample_LCA24.Cluster1988cbin.1	96.64	0.335	17
Sample_LCA24.mb.42cbin.1	97.97	0.021	126
Sample_LCA24.mb.25	94.19	1.298	137
Sample_LCA24.mb.38cbin.1	88.51	1.166	115
Sample_LCA24.Cluster4798	99.47	1.570	28
Sample_LCA24.mb.40	99.07	1.612	18
Sample_LCA24.Cluster11	98.13	0.745	110
Sample_LCA24.mb.55	100.00	0.000	50
Sample_LCA24.mb.9	94.73	2.880	132
Sample_LCA24.mb.19	91.34	4.086	27
Sample_LCA24.mb.20	90.48	1.019	159
Sample_LCA24.Cluster1580cbin.1	81.45	0.681	50
Sample_LCA24.mb.33cbin.1	99.27	0.236	73
Sample_LCA24.mb.7	80.57	0.755	52
Sample_LCA25.mb.50	98.65	0.000	15
Sample_LCA25.mb.8	81.55	2.320	325
Sample_LCA25.mb.20	88.59	0.671	18
Sample_LCA25.Cluster2783	99.98	1.497	21
Sample_LCA25.mb.15	96.87	1.057	160
Sample_LCA25.mb.7	97.59	1.442	28
Sample_LCA25.Cluster326cbin.1	80.24	1.360	88
Sample_LCA25.mb.19cbin.1	82.65	1.698	429
Sample_LCA25.mb.35cbin.1	99.21	0.476	121
Sample_LCA25.Cluster1744cbin.1	87.81	0.000	60
Sample_LCA25.Cluster3706cbin.1	97.87	0.370	109
Sample_LCA25.Cluster6469	98.87	1.123	55
Sample_LCA25.mb.6cbin.1	91.27	1.942	130
Sample_LCA25.Cluster2442cbin.1	98.79	1.932	120
Sample_LCA25.mb.18cbin.1	100.00	0.632	72
Sample_LCA25.mb.26	99.03	0.845	78
Sample_LCA25.Cluster1321cbin.1	83.34	2.775	674
Sample_LCA25.Cluster192	98.70	0.418	99
Sample_LCB09.Cluster10320	92.41	4.494	8
Sample_LCB09.mb.33	99.19	1.075	75
Sample_LCB09.Cluster9274cbin.1	87.97	1.476	332
Sample_LCB09.mb.34	92.59	1.342	38
Sample_LCB09.mb.22	82.86	2.348	121
Sample_LCB09.mb.57	83.79	0.000	147
Sample_LCB09.Cluster2908cbin.1	97.31	0.000	56
Sample_LCB09.mb.19cbin.1	82.18	2.210	183
Sample_LCB09.Cluster66cbin.1	87.79	0.226	74
Sample_LCB09.mb.46cbin.1	92.22	4.629	292
Sample_LCB09.Cluster1827	85.86	0.000	15
Sample_LCB09.mb.58cbin.1	93.06	0.000	59
Sample_LCB09.Cluster7974	99.32	0.854	18
Sample_LCB09.mb.49cbin.1	95.56	3.481	182
Sample_LCB09.Cluster9335	81.83	1.287	281
Sample_LCB09.mb.78	89.11	0.000	54

Sample_LCB09.mb.65cbin.1	88.19	0.000	72
Sample_LCB09.Cluster862cbin.1	89.54	0.795	18
Sample_LCB09.mb.31cbin.1	95.63	1.360	276
Sample_LCB09.mb.70	86.91	3.825	89
Sample_LCB09.mb.42cbin.1	98.79	1.025	135
Sample_LCB09.mb.69cbin.1	90.60	0.671	64
Sample_LCB09.mb.18	97.76	2.013	72
Sample_LCB09.mb.90	90.32	0.782	316
Sample_LCB09.mb.2	86.64	2.046	262
Sample_LCB09.mb.54	98.24	1.932	72
Sample_LCB09.Cluster6670cbin.1	98.30	0.966	98
Sample_LCB09.mb.6	96.59	0.680	222
Sample_LCB09.Cluster345cbin.1	98.01	0.961	36
Sample_LCB09.mb.67	96.58	1.743	90
Sample_LCB09.mb.82cbin.1	96.97	0.000	13
Sample_LCB09.Cluster3531	93.47	2.354	45
Sample_LCB09.mb.73cbin.1	96.57	4.109	33
Sample_LCB09.Cluster3297cbin.1	95.36	0.123	81
Sample_LCB09.mb.50cbin.1	98.46	0.128	59
Sample_LCB09.mb.51cbin.1	98.49	1.127	112
Sample_LCB10.Cluster10459	92.41	4.494	7
Sample_LCB10.mb.100	94.40	0.167	49
Sample_LCB10.Cluster43cbin.1	80.18	4.089	292
Sample_LCB10.mb.113	87.91	1.006	116
Sample_LCB10.mb.30	98.38	0.000	46
Sample_LCB10.mb.20	95.30	1.342	44
Sample_LCB10.Cluster2571cbin.1	97.31	0.000	111
Sample_LCB10.mb.104cbin.1	97.09	0.000	73
Sample_LCB10.Cluster2169cbin.1	93.03	0.021	104
Sample_LCB10.Cluster49	95.04	0.921	32
Sample_LCB10.mb.71	93.71	1.677	100
Sample_LCB10.mb.8	92.94	4.545	425
Sample_LCB10.mb.58	88.54	1.304	203
Sample_LCB10.Cluster7878	97.98	0.671	16
Sample_LCB10.Cluster362	95.23	0.000	35
Sample_LCB10.mb.120cbin.1	97.15	0.632	69
Sample_LCB10.mb.106	95.23	0.680	216
Sample_LCB10.Cluster2330cbin.1	97.27	0.000	18
Sample_LCB10.Cluster10213	99.47	0.000	22
Sample_LCB10.mb.11	97.31	0.335	12
Sample_LCB10.mb.19cbin.1	91.72	2.380	174
Sample_LCB10.Cluster1270cbin.1	94.77	0.454	28
Sample_LCB10.mb.31cbin.1	92.62	2.415	273
Sample_LCB10.mb.67	98.94	0.632	85
Sample_LCB10.mb.63cbin.1	92.61	3.187	74
Sample_LCB10.Cluster7401cbin.1	98.65	0.000	56
Sample_LCB10.mb.89	99.19	1.075	74
Sample_LCB10.mb.52	92.31	3.628	190
Sample_LCB10.Cluster2199cbin.1	92.94	1.851	136
Sample_LCB10.mb.40cbin.1	99.51	1.201	113
Sample_LCB10.Cluster5231cbin.1	98.88	1.690	87
Sample_LCB10.mb.82cbin.1	99.10	0.732	71
Sample_LCB10.mb.44cbin.1	98.89	0.000	67
Sample_LCB10.mb.116cbin.1	96.51	1.054	211
Sample_LCB10.mb.81	91.57	2.380	129
Sample_LCB10.Cluster3344	98.52	0.371	43
Sample_LCB10.Cluster5766cbin.1	88.91	2.150	554
Sample_LCB10.Cluster2061	97.69	0.128	59

Sample_LCB10.mb.93	93.45	0.000	55
Sample_LCB10.mb.97cbin.1	98.63	1.369	28
Sample_LCB10.mb.99	99.27	0.961	27
Sample_LCB10.mb.75	93.79	0.123	56
Sample_LCB10.mb.61cbin.1	99.24	0.939	122
Sample_LCB11.mb.22cbin.1	89.88	3.981	106
Sample_LCB11.Cluster4735	92.93	1.342	22
Sample_LCB11.mb.57cbin.1	99.05	2.507	230
Sample_LCB11.Cluster68	84.73	2.348	128
Sample_LCB11.mb.30	95.56	3.117	104
Sample_LCB11.Cluster4219cbin.1	91.24	1.371	296
Sample_LCB11.Cluster626cbin.1	86.57	2.460	100
Sample_LCB11.mb.51	88.50	0.671	217
Sample_LCB11.mb.34	80.20	2.684	40
Sample_LCB11.mb.62	96.77	0.403	42
Sample_LCB11.mb.36cbin.1	87.02	0.000	42
Sample_LCB11.mb.55	99.26	0.000	26
Sample_LCB11.mb.45	97.31	1.342	50
Sample_LCB11.mb.47	97.98	0.671	119
Sample_LCB11.Cluster9145cbin.1	98.65	0.671	19
Sample_LCB11.Cluster802	100.00	0.000	25
Sample_LCB11.Cluster1742	97.27	0.000	30
Sample_LCB11.Cluster7542cbin.1	100.00	0.167	69
Sample_LCB11.mb.52	90.60	3.691	46
Sample_LCB11.mb.19cbin.1	94.63	1.342	57
Sample_LCB11.mb.35cbin.1	99.32	1.738	26
Sample_LCB11.Cluster7794cbin.1	87.84	3.801	422
Sample_LCB11.mb.77cbin.1	81.01	4.530	214
Sample_LCB11.mb.63	100.00	0.000	44
Sample_LCB11.mb.17	86.91	1.486	217
Sample_LCB11.Cluster6984cbin.1	94.35	1.025	412
Sample_LCB11.Cluster5870cbin.1	98.71	2.307	72
Sample_LCB11.mb.32	94.17	2.075	68
Sample_LCB11.mb.29	98.66	0.064	84
Sample_LCB12.Cluster2504cbin.1	97.76	0.000	32
Sample_LCB12.Cluster6052cbin.1	86.29	0.000	21
Sample_LCB12.mb.110	91.57	3.370	31
Sample_LCB12.Cluster14970cbin.1	100.00	0.671	29
Sample_LCB12.Cluster30	80.58	1.342	142
Sample_LCB12.mb.1	98.38	0.000	40
Sample_LCB12.mb.30	91.90	0.000	203
Sample_LCB12.mb.42	91.29	1.685	18
Sample_LCB12.mb.16	91.92	1.342	39
Sample_LCB12.mb.113cbin.1	96.97	0.671	41
Sample_LCB12.mb.116cbin.1	88.77	3.148	107
Sample_LCB12.Cluster8572cbin.1	98.99	0.000	47
Sample_LCB12.Cluster347cbin.1	97.59	0.370	58
Sample_LCB12.mb.125cbin.1	90.93	1.789	48
Sample_LCB12.Cluster6	91.91	0.591	215
Sample_LCB12.mb.13cbin.1	89.43	0.433	59
Sample_LCB12.Cluster3428cbin.1	88.05	4.026	31
Sample_LCB12.Cluster725	95.91	0.000	101
Sample_LCB12.Cluster10251cbin.1	98.24	2.898	48
Sample_LCB12.Cluster11873cbin.1	98.98	1.970	105
Sample_LCB12.mb.124	96.97	0.335	85
Sample_LCB12.mb.105cbin.1	98.65	0.427	19
Sample_LCB12.Cluster13525cbin.1	92.48	1.710	437
Sample_LCB12.mb.91	87.69	1.342	85

Sample_LCB12.mb.37	95.30	1.118	88
Sample_LCB12.mb.101	94.80	2.403	233
Sample_LCB12.mb.90	84.56	0.000	32
Sample_LCB12.mb.45	87.98	1.442	97
Sample_LCB12.mb.8	89.97	0.000	64
Sample_LCB12.mb.55cbin.1	91.40	2.721	295
Sample_LCB12.mb.54	99.32	0.671	67
Sample_LCB12.mb.75	98.38	1.612	60
Sample_LCB12.mb.77	88.25	0.000	9
Sample_LCB12.mb.92	89.21	0.640	38
Sample_LCB12.Cluster6103	86.44	1.858	85
Sample_LCB12.mb.32	94.73	0.000	61
Sample_LCB12.mb.93	80.54	2.013	248
Sample_LCB12.mb.82	90.84	2.551	128
Sample_LCB12.mb.80cbin.1	92.31	1.442	256
Sample_LCB12.mb.7cbin.1	93.06	0.000	30
Sample_LCB12.mb.94cbin.1	98.10	0.021	83
Sample_LCB12.mb.95	85.30	1.565	224
Sample_LCB12.mb.86cbin.1	99.03	3.846	20
Sample_LCB12.mb.56cbin.1	99.03	4.287	106
Sample_LCB12.mb.25	95.47	1.823	272
Sample_LCB12.mb.74	94.54	0.128	297
Sample_LCB12.mb.136cbin.1	96.24	1.440	281
Sample_LCB12.mb.33cbin.1	91.84	1.111	308
Sample_LCB13.Cluster4317cbin.1	96.77	1.075	44
Sample_LCB13.Cluster6252cbin.1	93.93	1.342	27
Sample_LCB13.mb.110	96.37	0.879	145
Sample_LCB13.mb.12	90.38	1.363	191
Sample_LCB13.Cluster228	86.99	1.342	42
Sample_LCB13.Cluster308cbin.1	89.28	4.697	52
Sample_LCB13.Cluster7562cbin.1	93.56	0.671	220
Sample_LCB13.mb.18cbin.1	87.58	2.929	249
Sample_LCB13.Cluster12208	94.36	0.335	31
Sample_LCB13.Cluster567cbin.1	97.58	1.209	43
Sample_LCB13.Cluster3631	94.63	0.671	9
Sample_LCB13.Cluster11305cbin.1	96.37	1.006	21
Sample_LCB13.mb.70	89.32	1.123	26
Sample_LCB13.Cluster799cbin.1	91.22	2.782	85
Sample_LCB13.mb.111cbin.1	90.77	1.172	280
Sample_LCB13.Cluster219cbin.1	96.15	0.480	112
Sample_LCB13.mb.112	98.40	0.000	63
Sample_LCB13.mb.41	91.04	1.342	53
Sample_LCB13.mb.105cbin.1	99.08	1.497	84
Sample_LCB13.mb.46	83.62	3.309	327
Sample_LCB13.Cluster10074cbin.1	83.77	2.830	561
Sample_LCB13.mb.27	96.15	1.442	96
Sample_LCB13.mb.68	87.91	0.671	30
Sample_LCB13.mb.72	80.51	2.425	252
Sample_LCB13.mb.71	94.78	0.671	88
Sample_LCB13.mb.34cbin.1	95.73	0.591	135
Sample_LCB13.mb.15	97.66	0.000	51
Sample_LCB13.mb.31	99.03	0.221	43
Sample_LCB13.mb.19	90.56	0.377	39
Sample_LCB13.mb.83	85.23	2.135	44
Sample_LCB13.Cluster7062	99.46	0.000	71
Sample_LCB13.mb.98	94.93	1.582	112
Sample_LCB13.mb.28	91.25	1.305	85
Sample_LCB13.mb.69	88.82	1.267	203

Sample_LCB13.mb.80	94.29	1.525	55
Sample_LCB13.mb.9cbin.1	98.63	0.000	46
Sample_LCB13.mb.36cbin.1	98.51	1.635	39
Sample_LCB13.mb.43	98.46	0.384	27
Sample_LCB13.mb.60	98.23	0.961	140
Sample_LCB13.Cluster5307cbin.1	98.40	1.029	193
Sample_LCB13.Cluster5606cbin.1	98.31	1.384	192
Sample_LCB14.mb.6	93.95	0.832	14
Sample_LCB14.Cluster696	85.90	0.000	27
Sample_LCB14.mb.43	97.98	0.000	57
Sample_LCB14.Cluster2207	99.18	0.000	22
Sample_LCB14.mb.45	93.17	1.363	296
Sample_LCB14.Cluster11cbin.1	97.29	0.671	52
Sample_LCB14.Cluster1090cbin.1	88.62	2.913	364
Sample_LCB14.mb.4cbin.1	92.51	0.680	226
Sample_LCB14.mb.63cbin.1	97.98	1.342	65
Sample_LCB14.Cluster704cbin.1	98.08	0.000	61
Sample_LCB14.mb.5	99.22	0.000	9
Sample_LCB14.Cluster16cbin.1	90.64	4.256	144
Sample_LCB14.Cluster238cbin.1	97.00	0.000	73
Sample_LCB14.mb.46cbin.1	98.95	0.000	33
Sample_LCB14.mb.55	82.16	0.000	583
Sample_LCB14.mb.65	91.83	0.000	47
Sample_LCB14.Cluster4494	96.47	0.704	25
Sample_LCB14.mb.52cbin.1	96.51	1.140	363
Sample_LCB14.Cluster247	99.27	0.480	23
Sample_LCB14.mb.36	84.35	1.858	103
Sample_LCB14.mb.21cbin.1	95.32	0.584	64
Sample_LCB14.Cluster1916cbin.1	91.79	0.000	77
Sample_LCB14.Cluster1757	98.18	0.185	26
Sample_LCB14.mb.3	88.10	0.000	20
Sample_LCB14.Cluster2653cbin.1	83.81	1.488	128
Sample_LCB14.Cluster2742cbin.1	99.03	0.320	149
Sample_LCB14.mb.50	90.84	0.000	25
Sample_LCB14.mb.25cbin.1	94.15	0.526	156
Sample_LCB14.mb.37	96.93	4.727	313
Sample_LCB15.mb.1	94.62	0.000	56
Sample_LCB15.mb.32	87.69	3.330	493
Sample_LCB15.mb.3cbin.1	91.63	1.442	24
Sample_LCB15.mb.28	92.39	4.006	287
Sample_LCB15.mb.48cbin.1	96.15	3.628	332
Sample_LCB15.mb.50cbin.1	96.64	2.369	129
Sample_LCB15.mb.24cbin.1	81.59	4.101	103
Sample_LCB15.mb.20cbin.1	97.82	1.060	142
Sample_LCB15.mb.23cbin.1	86.33	1.476	261
Sample_LCB15.mb.18cbin.1	92.39	1.342	215
Sample_LCB15.mb.43	85.02	0.000	44
Sample_LCB15.Cluster42cbin.1	90.64	0.850	110
Sample_LCB15.mb.33	91.57	3.988	305
Sample_LCB15.Cluster1020	93.03	0.495	74
Sample_LCB15.mb.31	92.49	3.345	204
Sample_LCB15.mb.49cbin.1	95.22	2.327	233
Sample_LCB15.mb.17cbin.1	89.64	0.409	44
Sample_LCB15.mb.34cbin.1	88.08	0.673	48
Sample_LCB16.mb.50cbin.1	85.24	1.075	253
Sample_LCB16.mb.28	83.30	1.265	259
Sample_LCB16.mb.41cbin.1	85.80	0.000	352
Sample_LCB16.Cluster692cbin.1	94.39	0.961	30

Sample_LCB16.Cluster819	93.23	0.898	60
Sample_LCB16.Cluster2885	98.65	0.671	16
Sample_LCB16.mb.54	91.83	0.850	56
Sample_LCB16.Cluster43	89.82	0.460	234
Sample_LCB16.mb.57	83.30	0.316	326
Sample_LCB16.mb.66cbin.1	98.41	1.020	238
Sample_LCB16.mb.14	92.93	2.499	66
Sample_LCB16.Cluster4450cbin.1	98.65	0.671	26
Sample_LCB16.Cluster4274cbin.1	99.03	1.207	71
Sample_LCB16.mb.64cbin.1	96.53	4.931	106
Sample_LCB16.mb.5cbin.1	86.63	1.889	381
Sample_LCB16.mb.26cbin.1	91.94	0.673	44
Sample_LCB17.Cluster2017	80.35	0.097	251
Sample_LCB17.Cluster12880cbin.1	98.65	0.000	12
Sample_LCB17.Cluster11076cbin.1	97.31	2.181	309
Sample_LCB17.mb.100	98.92	0.000	44
Sample_LCB17.Cluster58	83.17	0.469	169
Sample_LCB17.Cluster228	80.53	0.000	37
Sample_LCB17.Cluster2810	91.27	0.000	22
Sample_LCB17.Cluster5688	89.42	0.000	49
Sample_LCB17.mb.10cbin.1	100.00	0.335	22
Sample_LCB17.Cluster10309cbin.1	92.61	0.000	17
Sample_LCB17.Cluster3559cbin.1	92.42	0.632	81
Sample_LCB17.Cluster3277cbin.1	99.03	0.961	31
Sample_LCB17.Cluster245cbin.1	97.40	0.555	50
Sample_LCB17.mb.11	80.53	0.671	53
Sample_LCB17.Cluster13887cbin.1	92.60	2.508	455
Sample_LCB17.mb.4	92.41	1.123	16
Sample_LCB17.Cluster4	97.02	1.267	230
Sample_LCB17.mb.23	94.29	0.671	86
Sample_LCB17.mb.27	81.87	2.013	39
Sample_LCB17.Cluster5194cbin.1	88.70	1.932	257
Sample_LCB17.Cluster14164cbin.1	86.07	0.632	61
Sample_LCB17.Cluster4975	96.28	1.689	85
Sample_LCB17.mb.128	85.23	0.000	59
Sample_LCB17.Cluster8299	90.33	1.449	75
Sample_LCB17.mb.127	93.75	0.480	49
Sample_LCB17.mb.126	89.35	3.596	134
Sample_LCB17.mb.26	100.00	0.961	25
Sample_LCB17.mb.131cbin.1	99.54	0.075	28
Sample_LCB17.Cluster686cbin.1	97.92	0.345	242
Sample_LCB17.mb.67	85.24	2.828	187
Sample_LCB17.mb.108	90.23	0.000	37
Sample_LCB17.Cluster12249cbin.1	98.38	1.612	123
Sample_LCB17.mb.65	93.95	0.000	83
Sample_LCB17.mb.106	97.54	0.000	77
Sample_LCB17.mb.24cbin.1	87.35	0.000	37
Sample_LCB17.mb.94cbin.1	97.90	1.296	130
Sample_LCB17.mb.83cbin.1	88.24	2.118	137
Sample_LCB17.mb.59cbin.1	99.51	3.381	74
Sample_LCB17.Cluster5491cbin.1	97.15	1.153	164
Sample_LCB17.mb.21	89.81	1.865	51
Sample_LCB17.mb.88	95.69	2.580	195
Sample_LCB17.mb.71	97.27	1.342	26
Sample_LCB17.mb.73	97.78	1.360	250
Sample_LCB17.mb.80cbin.1	91.61	0.671	123
Sample_LCB17.mb.86cbin.1	92.48	1.006	45
Sample_LCB17.mb.84	97.98	0.503	40

Sample_LCB17.mb.98	96.97	0.671	21
Sample_LCB17.mb.118cbin.1	92.56	3.290	586
Sample_LCB17.mb.81cbin.1	92.74	0.746	99
Sample_LCB17.mb.62	97.20	1.185	374
Sample_LCB19.mb.11cbin.1	95.69	0.000	50
Sample_LCB19.Cluster50cbin.1	89.93	0.061	17
Sample_LCB19.mb.17cbin.1	86.77	2.013	273
Sample_LCB19.Cluster1720	97.04	0.632	38
Sample_LCB19.mb.91	81.76	1.237	78
Sample_LCB19.Cluster2356	99.40	0.967	18
Sample_LCB19.Cluster833	94.96	0.000	5
Sample_LCB19.mb.47cbin.1	91.03	0.210	71
Sample_LCB19.Cluster1786	92.49	1.497	40
Sample_LCB19.Cluster23cbin.1	86.79	3.766	337
Sample_LCB19.Cluster270cbin.1	94.11	1.250	189
Sample_LCB19.Cluster4285cbin.1	99.32	0.671	20
Sample_LCB19.mb.44cbin.1	93.58	0.671	215
Sample_LCB19.mb.49	83.75	0.181	36
Sample_LCB19.Cluster3608	96.57	0.000	27
Sample_LCB19.mb.65	81.03	0.680	76
Sample_LCB19.mb.58	94.59	3.639	228
Sample_LCB19.Cluster2409cbin.1	88.07	1.006	333
Sample_LCB19.mb.34	99.21	1.357	48
Sample_LCB19.mb.87	94.55	0.680	206
Sample_LCB19.mb.38	93.54	0.806	51
Sample_LCB19.mb.67	91.13	0.969	84
Sample_LCB19.mb.75cbin.1	97.31	0.000	30
Sample_LCB19.Cluster4209cbin.1	98.42	2.898	77
Sample_LCB19.Cluster3027cbin.1	92.45	0.557	82
Sample_LCB19.mb.41	91.72	2.241	228
Sample_LCB19.mb.1	86.10	2.407	150
Sample_LCB19.mb.61cbin.1	97.10	4.347	95
Sample_LCB19.Cluster959cbin.1	96.18	0.384	148
Sample_LCB19.mb.5	89.24	0.700	48
Sample_LCB20.Cluster10120	91.57	2.247	13
Sample_LCB20.Cluster9862	91.57	1.123	11
Sample_LCB20.mb.6	92.69	2.247	18
Sample_LCB20.mb.46	83.02	1.230	363
Sample_LCB20.Cluster531cbin.1	90.50	0.671	101
Sample_LCB20.mb.31cbin.1	80.53	0.209	36
Sample_LCB20.mb.55cbin.1	98.60	2.192	124
Sample_LCB20.mb.32	93.67	0.671	180
Sample_LCB20.mb.17	91.14	4.450	178
Sample_LCB20.Cluster3757cbin.1	89.55	2.365	293
Sample_LCB20.mb.52cbin.1	85.01	0.000	40
Sample_LCB20.mb.37	80.70	1.067	271
Sample_LCB20.mb.78	85.90	3.041	138
Sample_LCB20.Cluster7747	97.09	0.671	21
Sample_LCB20.mb.10cbin.1	97.98	0.335	16
Sample_LCB20.Cluster552cbin.1	97.93	1.013	52
Sample_LCB20.mb.64	84.22	1.812	394
Sample_LCB20.mb.20	91.73	1.121	106
Sample_LCB20.Cluster3414	96.60	1.006	18
Sample_LCB20.mb.16	97.38	0.671	105
Sample_LCB20.mb.68cbin.1	89.93	1.677	32
Sample_LCB20.mb.79cbin.1	95.97	0.000	21
Sample_LCB20.mb.19	91.05	0.523	28
Sample_LCB20.mb.65	99.51	0.961	27

Sample_LCB20.mb.54cbin.1	94.11	2.564	83
Sample_LCB20.Cluster5370cbin.1	99.62	0.000	20
Sample_LCB20.Cluster5551cbin.1	98.38	0.000	98
Sample_LCB20.Cluster6220cbin.1	91.09	0.769	497
Sample_LCB20.mb.30	98.52	0.384	70
Sample_LCB20.mb.23cbin.1	97.35	3.018	194
Sample_LCB20.mb.83	93.65	4.197	209
Sample_LCB20.Cluster2174	95.80	0.106	48
Sample_LCB20.mb.3	93.65	0.109	47
Sample_LCB21.Cluster12270cbin.1	90.66	1.333	150
Sample_LCB21.Cluster33cbin.1	83.60	3.205	308
Sample_LCB21.mb.108	93.93	2.013	34
Sample_LCB21.Cluster348	83.28	0.671	48
Sample_LCB21.Cluster3886cbin.1	86.20	0.733	178
Sample_LCB21.mb.18cbin.1	81.22	0.185	55
Sample_LCB21.Cluster3188cbin.1	95.97	1.342	16
Sample_LCB21.Cluster771cbin.1	91.94	3.020	142
Sample_LCB21.mb.95cbin.1	96.77	1.075	72
Sample_LCB21.Cluster9179	99.32	0.671	17
Sample_LCB21.Cluster6899cbin.1	89.13	1.845	215
Sample_LCB21.mb.56cbin.1	92.41	2.684	179
Sample_LCB21.Cluster7124cbin.1	99.05	0.949	166
Sample_LCB21.mb.76	86.92	0.707	91
Sample_LCB21.mb.98cbin.1	95.56	2.013	55
Sample_LCB21.mb.77	98.55	2.673	51
Sample_LCB21.mb.50cbin.1	96.93	0.335	29
Sample_LCB21.mb.70	93.14	2.531	73
Sample_LCB21.Cluster4525cbin.1	96.83	0.105	79
Sample_LCB21.mb.102cbin.1	98.79	1.442	36
Sample_LCB21.mb.36cbin.1	90.93	2.046	213
Sample_LCB21.Cluster8836cbin.1	97.58	2.415	263
Sample_LCB21.Cluster84cbin.1	91.94	2.315	180
Sample_LCB21.mb.9cbin.1	95.78	1.497	84
Sample_LCB21.mb.91	97.09	0.000	30
Sample_LCB21.mb.96	100.00	1.380	139
Sample_LCB21.mb.53cbin.1	92.81	4.232	236
Sample_LCB21.Cluster7184cbin.1	97.09	0.961	140
Sample_LCB21.mb.68	88.93	2.230	183
Sample_LCB21.mb.87	91.37	3.557	173
Sample_LCB21.mb.66	94.70	1.347	206
Sample_LCB21.Cluster4135cbin.1	99.81	0.477	94
Sample_LCB22.Cluster2302cbin.1	96.77	2.121	39
Sample_LCB22.mb.44	80.95	3.327	127
Sample_LCB22.Cluster11885	84.61	0.000	21
Sample_LCB22.mb.4cbin.1	93.93	2.684	37
Sample_LCB22.Cluster494cbin.1	98.43	1.006	160
Sample_LCB22.mb.92	91.29	1.123	60
Sample_LCB22.Cluster190cbin.1	92.47	0.806	155
Sample_LCB22.mb.15	94.12	0.671	110
Sample_LCB22.mb.7	99.30	1.165	100
Sample_LCB22.Cluster4801cbin.1	89.85	1.449	64
Sample_LCB22.Cluster3595	99.40	0.898	41
Sample_LCB22.mb.50cbin.1	97.98	0.671	44
Sample_LCB22.Cluster6899	95.97	0.000	10
Sample_LCB22.mb.16	93.95	1.006	85
Sample_LCB22.Cluster127	97.95	0.000	28
Sample_LCB22.mb.31	85.38	1.077	236
Sample_LCB22.Cluster9445	97.98	0.335	29

Sample_LCB22.mb.10	98.65	0.000	20
Sample_LCB22.mb.64cbin.1	94.95	1.805	90
Sample_LCB22.Cluster10629cbin.1	95.61	3.304	363
Sample_LCB22.Cluster7041cbin.1	91.30	1.560	82
Sample_LCB22.mb.24	93.28	0.000	21
Sample_LCB22.Cluster5886	95.49	0.784	76
Sample_LCB22.mb.74	97.37	1.250	86
Sample_LCB22.mb.58	85.50	1.449	84
Sample_LCB22.mb.71cbin.1	92.56	3.490	153
Sample_LCB22.Cluster2133cbin.1	84.53	3.522	175
Sample_LCB22.Cluster4267	99.23	0.512	74
Sample_LCB22.mb.63cbin.1	94.02	0.836	184
Sample_LCB22.mb.13cbin.1	97.15	2.371	215
Sample_LCB22.mb.91cbin.1	96.68	0.537	153
Sample_LCB22.mb.46cbin.1	99.29	0.079	57
Sample_LCB23.Cluster8702	97.98	1.677	14
Sample_LCB23.Cluster351cbin.1	94.63	0.020	39
Sample_LCB23.Cluster597cbin.1	94.36	2.348	113
Sample_LCB23.Cluster4176cbin.1	94.01	1.497	111
Sample_LCB23.Cluster153	98.92	3.225	27
Sample_LCB23.Cluster1202cbin.1	91.56	0.223	151
Sample_LCB23.mb.15cbin.1	90.00	0.000	53
Sample_LCB23.Cluster750cbin.1	98.55	1.923	50
Sample_LCB23.Cluster5199	84.56	0.000	21
Sample_LCB23.Cluster540	97.27	0.000	83
Sample_LCB23.Cluster10099cbin.1	85.47	2.034	280
Sample_LCB23.Cluster7723	99.32	4.362	22
Sample_LCB23.Cluster10438	91.50	0.000	121
Sample_LCB23.mb.5cbin.1	96.41	0.126	113
Sample_LCB23.Cluster37	96.27	0.621	81
Sample_LCB23.mb.87cbin.1	86.66	1.363	372
Sample_LCB23.mb.35	91.49	0.000	96
Sample_LCB23.Cluster527	97.20	0.718	74
Sample_LCB23.Cluster6112cbin.1	94.90	0.377	49
Sample_LCB23.Cluster647cbin.1	98.46	0.377	61
Sample_LCB23.mb.72	94.92	1.518	36
Sample_LCB23.mb.67cbin.1	91.13	1.898	77
Sample_LCB23.mb.73cbin.1	96.45	2.173	127
Sample_LCB23.mb.90cbin.1	96.37	0.584	76
Sample_LCB23.Cluster2610	99.06	0.897	72
Sample_LCB23.Cluster3712cbin.1	96.49	2.114	230
Sample_LCB23.mb.20cbin.1	99.37	1.218	185
Sample_LCB24.Cluster810	81.20	0.000	144
Sample_LCB24.Cluster528cbin.1	87.66	0.000	111
Sample_LCB24.mb.60cbin.1	88.04	0.671	122
Sample_LCB24.Cluster1115cbin.1	87.97	0.021	67
Sample_LCB24.Cluster162cbin.1	96.37	1.587	227
Sample_LCB24.Cluster253cbin.1	90.28	0.806	48
Sample_LCB24.Cluster3220cbin.1	97.98	0.671	15
Sample_LCB24.mb.56	98.38	1.612	92
Sample_LCB24.Cluster7479	99.47	0.000	30
Sample_LCB24.Cluster52	98.13	0.776	72
Sample_LCB24.Cluster4368cbin.1	99.27	0.966	64
Sample_LCB24.mb.61	98.63	0.546	47
Sample_LCB24.mb.70cbin.1	99.07	0.576	51
Sample_LCB24.Cluster4504cbin.1	81.67	3.812	578
Sample_LCB24.Cluster5212cbin.1	95.84	3.144	152
Sample_LCB24.mb.77	94.57	2.067	85

Sample_LCB24.mb.81cbin.1	98.80	0.000	44
Sample_LCB24.mb.50	93.82	1.461	185
Sample_LCB24.mb.7	95.10	2.818	97
Sample_LCB24.mb.71cbin.1	98.26	2.884	220
Sample_LCB24.mb.17cbin.1	97.72	1.347	397
Sample_LCB25.mb.71cbin.1	86.60	0.632	331
Sample_LCB25.Cluster768cbin.1	90.72	1.133	363
Sample_LCB25.Cluster4305cbin.1	89.51	0.838	269
Sample_LCB25.mb.21cbin.1	90.17	0.000	119
Sample_LCB25.Cluster468	94.83	0.961	36
Sample_LCB25.Cluster2594	99.98	1.497	22
Sample_LCB25.Cluster6031cbin.1	95.07	0.000	336
Sample_LCB25.Cluster2cbin.1	80.34	3.537	184
Sample_LCB25.mb.17	94.25	1.250	214
Sample_LCB25.mb.59	96.59	1.020	72
Sample_LCB25.mb.25	95.30	0.000	41
Sample_LCB25.mb.72	85.23	0.671	18
Sample_LCB25.Cluster14	99.76	0.000	20
Sample_LCB25.mb.65	96.04	0.000	38
Sample_LCB25.mb.70cbin.1	91.75	2.111	158
Sample_LCB25.mb.20cbin.1	87.69	3.762	77
Sample_LCB25.mb.22	80.28	2.898	104
Sample_LCB25.Cluster6084	98.87	1.123	58
Sample_LCB25.mb.45	95.28	0.566	117
Sample_LCB25.mb.58cbin.1	99.51	1.368	157
Sample_LCB25.mb.2cbin.1	100.00	0.632	68
Sample_LCB25.mb.10	96.52	0.362	74
Sample_LCB25.Cluster4572cbin.1	98.92	0.000	100
Sample_LCB25.Cluster1584cbin.1	97.05	3.793	227
Sample_LCB25.Cluster779cbin.1	99.44	0.381	111
Sample_LCC09.Cluster2953cbin.1	97.17	0.000	14
Sample_LCC09.Cluster1771cbin.1	95.84	1.612	150
Sample_LCC09.Cluster10253cbin.1	92.43	1.342	260
Sample_LCC09.mb.103cbin.1	97.42	0.335	51
Sample_LCC09.Cluster1325cbin.1	94.46	2.013	116
Sample_LCC09.Cluster32cbin.1	97.36	2.684	183
Sample_LCC09.mb.108cbin.1	94.40	0.000	71
Sample_LCC09.Cluster1576cbin.1	95.54	0.680	211
Sample_LCC09.Cluster1143cbin.1	91.92	3.401	190
Sample_LCC09.mb.110cbin.1	97.04	2.848	75
Sample_LCC09.mb.125	92.91	3.164	149
Sample_LCC09.Cluster11861	99.47	0.000	25
Sample_LCC09.Cluster2611	96.13	0.552	65
Sample_LCC09.mb.106cbin.1	94.63	0.000	15
Sample_LCC09.Cluster7300cbin.1	97.17	4.926	290
Sample_LCC09.Cluster9141	99.32	2.684	19
Sample_LCC09.mb.29cbin.1	87.24	0.000	71
Sample_LCC09.mb.59	93.93	1.342	34
Sample_LCC09.Cluster8951cbin.1	94.33	0.713	302
Sample_LCC09.mb.18	90.26	2.348	209
Sample_LCC09.mb.46cbin.1	90.60	1.764	52
Sample_LCC09.mb.128	95.71	3.623	45
Sample_LCC09.mb.137cbin.1	93.18	2.954	33
Sample_LCC09.Cluster898	95.43	2.003	31
Sample_LCC09.mb.49cbin.1	90.60	1.006	51
Sample_LCC09.mb.1cbin.1	96.57	0.000	29
Sample_LCC09.mb.41cbin.1	89.11	0.000	80
Sample_LCC09.Cluster5114cbin.1	89.61	2.366	40

Sample_LCC09.mb.96	94.02	1.342	199
Sample_LCC09.mb.79	85.51	3.322	380
Sample_LCC09.Cluster2591cbin.1	97.34	0.000	54
Sample_LCC09.mb.93	99.32	0.671	89
Sample_LCC09.mb.6	90.61	4.191	203
Sample_LCC09.mb.45	91.10	1.000	76
Sample_LCC09.mb.27cbin.1	98.73	1.371	129
Sample_LCC09.Cluster6301cbin.1	99.46	0.000	68
Sample_LCC09.mb.60cbin.1	94.00	1.382	29
Sample_LCC09.mb.20	80.19	0.123	62
Sample_LCC09.mb.56cbin.1	97.98	2.013	55
Sample_LCC09.mb.104	94.13	1.486	69
Sample_LCC09.mb.61cbin.1	96.49	0.292	77
Sample_LCC09.mb.64cbin.1	99.03	3.852	108
Sample_LCC09.mb.77	94.63	0.610	99
Sample_LCC10.Cluster8948	92.41	4.494	7
Sample_LCC10.mb.16	93.06	0.671	138
Sample_LCC10.Cluster705cbin.1	85.94	2.818	331
Sample_LCC10.Cluster634cbin.1	86.35	0.000	69
Sample_LCC10.mb.70	98.38	0.000	45
Sample_LCC10.mb.101cbin.1	95.74	3.489	82
Sample_LCC10.Cluster2822cbin.1	85.51	1.275	280
Sample_LCC10.mb.75	93.93	1.342	37
Sample_LCC10.mb.60cbin.1	91.94	3.020	236
Sample_LCC10.mb.46	93.28	0.167	66
Sample_LCC10.Cluster99	84.23	3.009	177
Sample_LCC10.Cluster1836cbin.1	89.24	0.065	74
Sample_LCC10.Cluster742	82.40	3.636	27
Sample_LCC10.mb.65cbin.1	88.79	1.687	273
Sample_LCC10.mb.102	93.21	2.040	155
Sample_LCC10.Cluster6772cbin.1	99.32	0.671	23
Sample_LCC10.Cluster620cbin.1	95.19	0.961	73
Sample_LCC10.mb.14cbin.1	87.43	1.690	37
Sample_LCC10.mb.47	87.18	0.680	348
Sample_LCC10.Cluster272	95.23	0.000	38
Sample_LCC10.mb.93	93.70	0.932	83
Sample_LCC10.Cluster8756cbin.1	99.47	0.000	24
Sample_LCC10.Cluster1767cbin.1	94.23	0.000	123
Sample_LCC10.Cluster6632cbin.1	89.48	2.263	381
Sample_LCC10.mb.63cbin.1	97.27	4.362	49
Sample_LCC10.mb.29	94.47	0.988	19
Sample_LCC10.mb.82cbin.1	97.31	0.000	12
Sample_LCC10.mb.84cbin.1	99.01	1.086	108
Sample_LCC10.Cluster5474cbin.1	96.72	2.957	44
Sample_LCC10.mb.89cbin.1	96.57	0.000	42
Sample_LCC10.Cluster3676cbin.1	96.08	0.123	72
Sample_LCC10.mb.83	99.36	0.105	125
Sample_LCC10.Cluster7124cbin.1	98.35	0.563	120
Sample_LCC10.mb.50cbin.1	96.50	0.000	234
Sample_LCC10.mb.38cbin.1	88.47	3.009	616
Sample_LCC10.mb.95	88.07	0.000	54
Sample_LCC10.mb.98cbin.1	92.75	4.757	167
Sample_LCC11.Cluster6383	89.91	1.342	23
Sample_LCC11.Cluster3915cbin.1	97.21	0.806	45
Sample_LCC11.mb.16cbin.1	81.50	1.944	228
Sample_LCC11.mb.47cbin.1	93.01	0.735	247
Sample_LCC11.mb.41cbin.1	96.37	2.956	183
Sample_LCC11.mb.11	82.66	0.000	39

Sample_LCC11.mb.64	85.39	1.398	431
Sample_LCC11.mb.33	86.29	3.355	134
Sample_LCC11.mb.52	87.58	0.671	48
Sample_LCC11.Cluster11053	97.31	0.000	35
Sample_LCC11.Cluster25cbin.1	94.95	0.268	175
Sample_LCC11.Cluster10094cbin.1	98.65	1.006	25
Sample_LCC11.Cluster10060cbin.1	97.31	0.111	53
Sample_LCC11.mb.62cbin.1	88.52	3.355	175
Sample_LCC11.Cluster5200	95.26	0.335	17
Sample_LCC11.Cluster2495	97.95	0.000	30
Sample_LCC11.mb.70	88.25	2.936	80
Sample_LCC11.mb.29	98.48	0.769	214
Sample_LCC11.mb.9cbin.1	87.02	2.839	202
Sample_LCC11.Cluster937	99.54	0.000	23
Sample_LCC11.Cluster8812	99.32	0.000	24
Sample_LCC11.Cluster8336cbin.1	84.78	2.309	477
Sample_LCC11.mb.80	82.51	4.697	435
Sample_LCC11.mb.54cbin.1	96.49	0.000	49
Sample_LCC11.Cluster6780	95.64	0.000	52
Sample_LCC11.mb.2	93.31	1.022	233
Sample_LCC11.mb.10	96.60	0.188	67
Sample_LCC12.Cluster3883	97.84	0.000	62
Sample_LCC12.Cluster3277	91.93	0.000	30
Sample_LCC12.mb.114	91.57	2.247	41
Sample_LCC12.Cluster5768	84.27	0.073	12
Sample_LCC12.mb.20	91.29	1.685	28
Sample_LCC12.Cluster12140	90.60	0.000	17
Sample_LCC12.mb.104	84.54	2.529	410
Sample_LCC12.Cluster15287cbin.1	98.65	1.447	43
Sample_LCC12.mb.117	90.87	0.671	119
Sample_LCC12.Cluster59cbin.1	88.92	4.870	208
Sample_LCC12.Cluster836cbin.1	93.47	4.194	78
Sample_LCC12.Cluster8334cbin.1	98.99	0.000	53
Sample_LCC12.Cluster1827	88.39	0.507	47
Sample_LCC12.mb.103cbin.1	88.36	0.041	49
Sample_LCC12.Cluster3878cbin.1	95.56	0.021	81
Sample_LCC12.mb.34cbin.1	80.53	1.174	176
Sample_LCC12.Cluster3959	90.67	2.013	18
Sample_LCC12.mb.111	98.38	2.419	70
Sample_LCC12.mb.91cbin.1	96.39	2.194	98
Sample_LCC12.mb.13	88.51	0.092	369
Sample_LCC12.mb.108cbin.1	96.77	0.000	115
Sample_LCC12.mb.58	87.08	0.061	48
Sample_LCC12.mb.16cbin.1	88.59	0.671	33
Sample_LCC12.mb.119	94.12	1.342	170
Sample_LCC12.mb.62cbin.1	83.89	4.250	50
Sample_LCC12.mb.19cbin.1	99.03	3.365	31
Sample_LCC12.mb.42	87.89	0.680	348
Sample_LCC12.mb.24	85.90	0.671	22
Sample_LCC12.Cluster4565cbin.1	87.86	2.487	280
Sample_LCC12.mb.29	97.20	0.000	83
Sample_LCC12.Cluster9948cbin.1	99.11	3.209	103
Sample_LCC12.mb.99	95.07	0.915	108
Sample_LCC12.mb.49cbin.1	95.74	0.000	80
Sample_LCC12.mb.6	93.71	1.207	51
Sample_LCC12.mb.69	93.04	2.380	266
Sample_LCC12.mb.72	89.71	1.020	168
Sample_LCC12.mb.77	96.64	0.335	52

Sample_LCC12.mb.47cbin.1	94.44	3.723	100
Sample_LCC12.mb.81	95.44	1.282	142
Sample_LCC12.mb.22	94.77	4.010	319
Sample_LCC12.mb.35cbin.1	98.71	1.486	79
Sample_LCC12.Cluster134cbin.1	97.06	0.619	249
Sample_LCC12.mb.68cbin.1	97.53	2.049	307
Sample_LCC13.Cluster3327cbin.1	96.77	1.075	42
Sample_LCC13.mb.15	81.03	3.448	129
Sample_LCC13.mb.10cbin.1	93.93	1.447	54
Sample_LCC13.Cluster148cbin.1	89.10	2.747	126
Sample_LCC13.Cluster203cbin.1	87.80	3.355	54
Sample_LCC13.mb.24	99.30	1.165	92
Sample_LCC13.mb.102cbin.1	83.30	2.013	77
Sample_LCC13.mb.107cbin.1	88.38	4.255	74
Sample_LCC13.mb.108cbin.1	86.35	0.488	46
Sample_LCC13.mb.2	88.92	1.510	60
Sample_LCC13.Cluster521cbin.1	98.38	0.806	51
Sample_LCC13.mb.22	81.44	1.812	292
Sample_LCC13.Cluster1228cbin.1	98.79	0.961	71
Sample_LCC13.Cluster2671	97.31	0.894	11
Sample_LCC13.mb.29	91.21	3.355	87
Sample_LCC13.Cluster13355	100.00	0.000	17
Sample_LCC13.Cluster9468cbin.1	85.90	1.921	41
Sample_LCC13.mb.20cbin.1	98.01	0.862	100
Sample_LCC13.Cluster283cbin.1	94.58	0.000	45
Sample_LCC13.mb.18	96.63	0.000	34
Sample_LCC13.Cluster6965cbin.1	81.40	1.364	319
Sample_LCC13.Cluster9914	98.65	1.006	20
Sample_LCC13.mb.1cbin.1	91.68	1.360	310
Sample_LCC13.Cluster19cbin.1	96.92	0.591	67
Sample_LCC13.Cluster477cbin.1	99.94	1.701	33
Sample_LCC13.Cluster8306	97.94	0.000	29
Sample_LCC13.mb.71cbin.1	94.74	2.013	60
Sample_LCC13.mb.53cbin.1	82.49	2.000	306
Sample_LCC13.mb.3	97.31	1.342	88
Sample_LCC13.mb.39cbin.1	93.99	1.497	81
Sample_LCC13.mb.36cbin.1	99.05	0.000	96
Sample_LCC13.mb.74	84.29	1.006	153
Sample_LCC13.Cluster5527	99.46	0.537	64
Sample_LCC13.mb.25	87.73	0.377	40
Sample_LCC13.mb.60	92.85	2.040	149
Sample_LCC13.mb.42cbin.1	95.23	0.000	231
Sample_LCC13.mb.69	95.19	2.964	49
Sample_LCC13.mb.96	84.22	0.671	95
Sample_LCC13.mb.93	86.32	0.648	207
Sample_LCC13.Cluster8065cbin.1	91.58	2.079	320
Sample_LCC13.mb.99cbin.1	96.42	1.923	113
Sample_LCC13.mb.30	95.57	0.384	28
Sample_LCC13.mb.75	98.24	0.584	59
Sample_LCC13.mb.47	93.49	0.512	92
Sample_LCC13.Cluster2164cbin.1	99.78	0.431	113
Sample_LCC13.mb.70	96.32	1.858	45
Sample_LCC13.mb.91cbin.1	83.42	2.125	687
Sample_LCC14.Cluster1275	93.95	0.832	14
Sample_LCC14.Cluster2550	99.18	0.000	22
Sample_LCC14.Cluster964	92.61	0.000	30
Sample_LCC14.mb.40	96.64	0.000	53
Sample_LCC14.mb.3	98.65	0.671	32

Sample_LCC14.mb.37	97.31	0.671	53
Sample_LCC14.mb.50cbin.1	89.06	4.746	598
Sample_LCC14.Cluster677cbin.1	83.12	0.345	398
Sample_LCC14.Cluster315cbin.1	94.55	0.680	224
Sample_LCC14.mb.58cbin.1	96.19	1.946	287
Sample_LCC14.Cluster994	99.18	0.000	61
Sample_LCC14.mb.10	83.10	1.700	67
Sample_LCC14.mb.23cbin.1	98.80	0.000	108
Sample_LCC14.mb.42cbin.1	98.95	0.000	32
Sample_LCC14.mb.83	99.22	0.000	9
Sample_LCC14.mb.62cbin.1	91.15	0.907	59
Sample_LCC14.Cluster234	99.27	0.480	22
Sample_LCC14.mb.48cbin.1	94.73	0.000	62
Sample_LCC14.mb.81cbin.1	95.30	1.665	371
Sample_LCC14.Cluster2264cbin.1	83.47	0.373	67
Sample_LCC14.mb.14cbin.1	91.33	1.898	566
Sample_LCC14.Cluster2175	98.51	0.185	25
Sample_LCC14.mb.64cbin.1	95.57	0.128	121
Sample_LCC14.Cluster2329cbin.1	93.13	0.000	23
Sample_LCC14.mb.27	96.61	2.364	211
Sample_LCC14.mb.72cbin.1	96.12	4.131	380
Sample_LCC15.mb.27cbin.1	96.84	0.177	168
Sample_LCC15.mb.14cbin.1	85.25	2.764	431
Sample_LCC15.Cluster403cbin.1	94.83	2.325	205
Sample_LCC15.mb.9	98.40	0.104	122
Sample_LCC16.mb.28	80.31	0.671	227
Sample_LCC16.Cluster1073cbin.1	96.41	1.323	39
Sample_LCC16.mb.15	87.26	1.916	337
Sample_LCC16.mb.34cbin.1	85.26	0.000	84
Sample_LCC16.mb.29cbin.1	97.31	0.000	18
Sample_LCC16.Cluster2701cbin.1	99.32	0.671	23
Sample_LCC16.mb.5cbin.1	94.39	0.961	36
Sample_LCC16.mb.1cbin.1	98.41	2.040	236
Sample_LCC16.Cluster75cbin.1	97.27	0.606	43
Sample_LCC16.mb.25	93.28	1.342	34
Sample_LCC16.mb.32	95.83	1.759	43
Sample_LCC16.Cluster2570	97.10	1.449	71
Sample_LCC16.Cluster2870cbin.1	91.77	1.611	102
Sample_LCC16.mb.30	88.31	1.486	154
Sample_LCC17.Cluster16446	93.82	0.000	21
Sample_LCC17.Cluster16367	91.57	4.494	13
Sample_LCC17.Cluster16722	92.41	1.123	13
Sample_LCC17.Cluster15858	89.33	2.777	136
Sample_LCC17.Cluster1862cbin.1	84.35	0.161	308
Sample_LCC17.Cluster3097cbin.1	93.70	0.932	65
Sample_LCC17.Cluster15226cbin.1	98.65	0.000	11
Sample_LCC17.Cluster4190cbin.1	98.38	0.000	44
Sample_LCC17.Cluster2315cbin.1	94.17	2.348	88
Sample_LCC17.Cluster180	83.68	1.342	131
Sample_LCC17.Cluster8838	97.84	0.000	41
Sample_LCC17.Cluster12879	89.93	0.000	16
Sample_LCC17.Cluster3989cbin.1	96.64	1.879	28
Sample_LCC17.Cluster15123	87.91	0.000	46
Sample_LCC17.Cluster15926	99.20	0.000	68
Sample_LCC17.Cluster8364	94.32	0.709	53
Sample_LCC17.mb.12cbin.1	87.39	0.671	44
Sample_LCC17.Cluster317	98.26	0.016	131
Sample_LCC17.Cluster4955cbin.1	98.55	0.961	31

Sample_LCC17.Cluster4788cbin.1	93.95	0.335	66
Sample_LCC17.mb.131cbin.1	94.07	2.013	83
Sample_LCC17.mb.36cbin.1	96.97	4.697	20
Sample_LCC17.Cluster44cbin.1	94.79	2.366	207
Sample_LCC17.mb.119	92.68	2.027	199
Sample_LCC17.Cluster9633cbin.1	99.03	0.961	39
Sample_LCC17.mb.74	86.66	0.000	21
Sample_LCC17.Cluster13321cbin.1	90.66	1.207	87
Sample_LCC17.Cluster750cbin.1	94.71	0.000	42
Sample_LCC17.Cluster16120cbin.1	99.68	1.582	96
Sample_LCC17.mb.52cbin.1	80.26	2.684	328
Sample_LCC17.mb.25cbin.1	93.40	4.260	235
Sample_LCC17.mb.53	89.11	3.243	179
Sample_LCC17.Cluster13753cbin.1	99.05	0.000	75
Sample_LCC17.mb.59cbin.1	95.30	3.477	28
Sample_LCC17.mb.22	90.73	1.057	265
Sample_LCC17.mb.120	92.61	0.671	49
Sample_LCC17.Cluster11558cbin.1	95.60	0.000	347
Sample_LCC17.mb.78	91.15	1.006	104
Sample_LCC17.mb.26	90.00	0.125	39
Sample_LCC17.mb.68	92.95	0.000	61
Sample_LCC17.Cluster14361cbin.1	87.55	1.089	533
Sample_LCC17.mb.86	87.13	0.671	268
Sample_LCC17.mb.67cbin.1	94.99	1.360	234
Sample_LCC17.mb.70	90.60	0.335	17
Sample_LCC17.mb.8cbin.1	93.23	1.449	63
Sample_LCC17.mb.88	94.63	0.000	56
Sample_LCC17.mb.95cbin.1	91.98	4.621	261
Sample_LCC17.mb.92cbin.1	93.91	1.342	25
Sample_LCC17.mb.94cbin.1	93.85	0.894	211
Sample_LCC17.mb.112cbin.1	95.20	2.614	163
Sample_LCC17.mb.82	96.05	0.097	143
Sample_LCC17.mb.73	96.93	0.631	37
Sample_LCC19.mb.44	93.54	0.000	50
Sample_LCC19.mb.64	91.57	3.370	42
Sample_LCC19.Cluster45cbin.1	90.60	0.734	18
Sample_LCC19.Cluster2436cbin.1	94.83	1.265	40
Sample_LCC19.mb.43	93.67	1.265	116
Sample_LCC19.Cluster744cbin.1	90.08	1.682	29
Sample_LCC19.Cluster1101	94.96	0.000	8
Sample_LCC19.mb.35	90.58	1.360	219
Sample_LCC19.mb.48cbin.1	89.07	0.572	103
Sample_LCC19.Cluster24cbin.1	81.54	1.766	315
Sample_LCC19.mb.19cbin.1	99.32	2.684	22
Sample_LCC19.Cluster3872cbin.1	88.35	0.000	54
Sample_LCC19.mb.17cbin.1	99.84	0.113	36
Sample_LCC19.Cluster5834cbin.1	99.32	0.503	51
Sample_LCC19.mb.90cbin.1	98.76	0.000	124
Sample_LCC19.mb.45	95.96	2.320	191
Sample_LCC19.mb.85cbin.1	90.25	1.276	41
Sample_LCC19.Cluster1741	82.07	0.000	54
Sample_LCC19.mb.49	88.34	0.929	82
Sample_LCC19.Cluster3286cbin.1	95.90	2.552	344
Sample_LCC19.mb.79	88.60	1.127	129
Sample_LCC19.mb.53cbin.1	97.54	4.616	193
Sample_LCC19.Cluster1812cbin.1	80.26	1.230	472
Sample_LCC19.mb.31cbin.1	96.94	1.067	46
Sample_LCC19.Cluster32	96.39	0.766	74

Sample_LCC20.Cluster10333	85.95	2.247	13
Sample_LCC20.mb.3	92.69	2.247	19
Sample_LCC20.Cluster8196cbin.1	97.31	0.134	23
Sample_LCC20.Cluster247cbin.1	97.31	0.671	45
Sample_LCC20.Cluster427cbin.1	92.39	1.006	100
Sample_LCC20.mb.57	93.47	0.537	216
Sample_LCC20.mb.21	99.32	0.000	26
Sample_LCC20.mb.43cbin.1	85.88	2.460	158
Sample_LCC20.Cluster3708	93.28	0.000	20
Sample_LCC20.mb.60	99.30	2.284	105
Sample_LCC20.mb.44	88.92	2.852	121
Sample_LCC20.Cluster2912cbin.1	95.91	0.000	29
Sample_LCC20.Cluster3319cbin.1	98.69	1.796	202
Sample_LCC20.Cluster689cbin.1	97.59	1.013	46
Sample_LCC20.Cluster5995	97.09	0.041	21
Sample_LCC20.mb.63	86.46	0.000	225
Sample_LCC20.mb.51	85.01	0.000	42
Sample_LCC20.mb.22	99.51	0.961	29
Sample_LCC20.mb.29cbin.1	97.98	0.335	16
Sample_LCC20.mb.75	93.40	0.000	198
Sample_LCC20.mb.32cbin.1	98.03	0.800	143
Sample_LCC20.mb.18	93.99	0.961	62
Sample_LCC20.mb.36cbin.1	95.67	1.531	146
Sample_LCC20.mb.56	95.57	1.360	221
Sample_LCC20.mb.92cbin.1	89.93	2.999	70
Sample_LCC20.Cluster5529	97.35	0.000	18
Sample_LCC20.Cluster5535	96.41	1.612	282
Sample_LCC20.mb.34cbin.1	96.60	1.376	225
Sample_LCC20.mb.62	96.04	4.429	204
Sample_LCC20.mb.94cbin.1	97.47	2.365	345
Sample_LCC20.Cluster3175	98.47	0.989	43
Sample_LCC20.Cluster625	96.07	0.106	43
Sample_LCC21.Cluster13810cbin.1	87.35	2.247	152
Sample_LCC21.Cluster163	88.04	2.016	160
Sample_LCC21.mb.33cbin.1	94.75	1.075	72
Sample_LCC21.mb.50cbin.1	85.04	0.854	179
Sample_LCC21.mb.31	88.01	3.355	314
Sample_LCC21.Cluster4626	89.25	0.074	45
Sample_LCC21.mb.43	93.28	0.000	47
Sample_LCC21.Cluster10978cbin.1	99.51	2.797	51
Sample_LCC21.Cluster4492cbin.1	87.67	0.747	247
Sample_LCC21.Cluster5920	96.21	0.898	93
Sample_LCC21.mb.104	94.63	0.000	38
Sample_LCC21.Cluster11322	99.32	2.013	20
Sample_LCC21.mb.92cbin.1	92.59	1.342	34
Sample_LCC21.Cluster5138cbin.1	96.93	1.677	24
Sample_LCC21.mb.79cbin.1	84.31	4.026	32
Sample_LCC21.mb.57cbin.1	90.18	3.571	224
Sample_LCC21.mb.107cbin.1	98.65	0.000	18
Sample_LCC21.mb.46	97.09	0.000	27
Sample_LCC21.mb.70	84.09	4.308	216
Sample_LCC21.mb.75	98.99	0.000	76
Sample_LCC21.Cluster8966cbin.1	98.63	0.000	42
Sample_LCC21.Cluster1186cbin.1	98.31	3.605	42
Sample_LCC21.Cluster13247cbin.1	100.00	0.632	79
Sample_LCC21.Cluster9689cbin.1	98.50	3.960	455
Sample_LCC21.mb.99cbin.1	89.65	2.040	460
Sample_LCC21.Cluster1714cbin.1	92.50	2.307	556

Sample_LCC21.mb.71cbin.1	99.58	1.184	162
Sample_LCC22.Cluster11576	85.33	0.000	33
Sample_LCC22.Cluster2424	96.77	0.097	35
Sample_LCC22.mb.22cbin.1	91.75	1.677	135
Sample_LCC22.mb.27	88.88	0.854	31
Sample_LCC22.mb.18cbin.1	99.30	1.165	83
Sample_LCC22.Cluster385	91.27	0.335	52
Sample_LCC22.mb.11cbin.1	96.64	0.000	72
Sample_LCC22.mb.40cbin.1	95.61	2.348	137
Sample_LCC22.Cluster128	99.19	1.612	51
Sample_LCC22.mb.35	91.96	0.671	65
Sample_LCC22.Cluster6998	95.97	0.000	9
Sample_LCC22.Cluster3715	99.40	0.898	41
Sample_LCC22.mb.47	89.81	0.000	198
Sample_LCC22.Cluster9158	94.63	0.335	27
Sample_LCC22.mb.16	88.81	3.911	110
Sample_LCC22.mb.46cbin.1	95.97	0.000	130
Sample_LCC22.mb.36	88.40	2.342	65
Sample_LCC22.Cluster278	97.95	0.000	27
Sample_LCC22.mb.2cbin.1	86.12	3.152	438
Sample_LCC22.Cluster8027cbin.1	98.63	0.000	46
Sample_LCC22.mb.19	96.61	0.000	46
Sample_LCC22.mb.37	92.61	0.894	21
Sample_LCC22.mb.76	93.95	0.671	30
Sample_LCC22.Cluster6899cbin.1	80.24	2.065	79
Sample_LCC22.mb.34cbin.1	95.86	2.364	137
Sample_LCC22.mb.62cbin.1	95.43	2.644	94
Sample_LCC22.mb.58	97.37	1.250	83
Sample_LCC22.mb.81cbin.1	82.80	2.154	238
Sample_LCC22.mb.31cbin.1	88.06	0.371	87
Sample_LCC22.mb.29cbin.1	98.73	4.236	380
Sample_LCC22.Cluster8294cbin.1	89.04	2.389	180
Sample_LCC22.mb.87	99.32	0.000	26
Sample_LCC22.mb.38	90.25	2.820	78
Sample_LCC22.mb.50	94.45	0.769	139
Sample_LCC22.mb.5cbin.1	94.71	1.971	276
Sample_LCC22.mb.92cbin.1	93.08	0.588	193
Sample_LCC22.mb.90cbin.1	99.59	0.037	56
Sample_LCC23.mb.89cbin.1	98.24	4.784	137
Sample_LCC23.mb.18cbin.1	87.19	4.865	294
Sample_LCC23.Cluster156	95.67	0.806	28
Sample_LCC23.mb.47	88.92	1.342	34
Sample_LCC23.Cluster3cbin.1	100.00	0.806	56
Sample_LCC23.Cluster976cbin.1	92.29	0.740	56
Sample_LCC23.Cluster311cbin.1	97.95	0.000	114
Sample_LCC23.Cluster8057	96.64	1.006	19
Sample_LCC23.mb.17	82.16	2.599	107
Sample_LCC23.Cluster9143	98.65	0.335	14
Sample_LCC23.mb.93cbin.1	93.95	0.000	35
Sample_LCC23.mb.26	93.92	2.237	126
Sample_LCC23.mb.55	98.61	0.961	59
Sample_LCC23.Cluster44cbin.1	93.78	0.621	97
Sample_LCC23.mb.19	88.52	2.021	337
Sample_LCC23.mb.83cbin.1	96.55	1.497	116
Sample_LCC23.Cluster7957cbin.1	94.62	1.582	66
Sample_LCC23.mb.54cbin.1	89.43	0.754	42
Sample_LCC23.mb.57	93.47	3.450	45
Sample_LCC23.mb.14	87.00	2.375	153

Sample_LCC23.mb.72cbin.1	85.07	0.377	52
Sample_LCC23.mb.98	98.65	0.000	24
Sample_LCC23.mb.87cbin.1	85.84	2.267	186
Sample_LCC23.mb.6	98.55	0.000	83
Sample_LCC23.mb.23cbin.1	92.30	0.897	92
Sample_LCC23.Cluster310cbin.1	96.44	1.268	339
Sample_LCC24.Cluster5757	87.72	0.732	372
Sample_LCC24.Cluster3364cbin.1	93.09	2.572	242
Sample_LCC24.Cluster82cbin.1	94.63	2.446	154
Sample_LCC24.Cluster946	90.60	0.000	111
Sample_LCC24.mb.18	92.74	0.806	64
Sample_LCC24.mb.53cbin.1	92.51	3.817	259
Sample_LCC24.Cluster172cbin.1	90.96	1.548	193
Sample_LCC24.Cluster78cbin.1	92.21	1.136	121
Sample_LCC24.mb.31	89.55	0.793	366
Sample_LCC24.mb.15	95.96	0.961	52
Sample_LCC24.mb.21	97.22	1.776	148
Sample_LCC24.Cluster6435cbin.1	99.47	0.000	29
Sample_LCC24.mb.23cbin.1	97.98	0.671	16
Sample_LCC24.Cluster44	98.13	0.621	79
Sample_LCC24.mb.4	93.08	2.684	165
Sample_LCC24.mb.50cbin.1	98.50	0.000	43
Sample_LCC24.Cluster2026cbin.1	91.50	0.062	75
Sample_LCC24.mb.26	96.79	2.163	36
Sample_LCC24.Cluster4973cbin.1	93.69	3.922	300
Sample_LCC24.mb.42	88.04	3.954	190
Sample_LCC24.mb.40cbin.1	99.27	1.378	57
Sample_LCC24.mb.60	88.99	4.272	296
Sample_LCC24.Cluster1831	91.41	0.384	159
Sample_LCC25.mb.22cbin.1	97.78	2.215	194
Sample_LCC25.mb.1	80.22	2.109	344
Sample_LCC25.Cluster6741cbin.1	86.38	1.408	345
Sample_LCC25.mb.2	98.92	0.806	111
Sample_LCC25.mb.31cbin.1	86.43	1.836	405
Sample_LCC25.mb.35	91.83	0.722	57
Sample_LCC25.Cluster2894	99.38	1.497	20
Sample_LCC25.Cluster5887	86.91	1.677	171
Sample_LCC25.mb.12	99.32	2.684	23
Sample_LCC25.mb.3cbin.1	92.75	1.709	293
Sample_LCC25.mb.73	97.59	1.442	25
Sample_LCC25.Cluster1411cbin.1	96.00	1.604	135
Sample_LCC25.mb.37cbin.1	98.06	0.644	109
Sample_LCC25.Cluster4290	85.99	0.362	46
Sample_LCC25.Cluster6913	98.87	1.123	82
Sample_LCC25.mb.5cbin.1	100.00	0.000	19
Sample_LCC25.Cluster2423cbin.1	86.47	1.492	53
Sample_LCC25.Cluster2907cbin.1	98.79	1.932	122
Sample_LCC25.mb.59cbin.1	100.00	0.632	68
Sample_LCC25.Cluster976cbin.1	93.67	2.631	161
Sample_LCC25.mb.21cbin.1	99.92	0.562	117
Sample_LPA01.Cluster5570cbin.1	87.81	1.956	311
Sample_LPA01.Cluster9393	98.65	0.000	13
Sample_LPA01.Cluster3463cbin.1	97.98	0.000	49
Sample_LPA01.mb.8	81.03	1.724	102
Sample_LPA01.mb.54	89.70	1.342	52
Sample_LPA01.Cluster1315cbin.1	98.10	0.021	83
Sample_LPA01.Cluster658cbin.1	92.30	0.961	27
Sample_LPA01.Cluster90cbin.1	98.65	0.020	39

Sample_LPA01.mb.57cbin.1	89.96	2.307	253
Sample_LPA01.Cluster9888	87.91	0.335	48
Sample_LPA01.mb.13	88.74	2.517	145
Sample_LPA01.mb.27cbin.1	96.25	0.680	240
Sample_LPA01.mb.87cbin.1	95.10	2.564	109
Sample_LPA01.mb.61cbin.1	87.67	2.551	200
Sample_LPA01.mb.21cbin.1	96.12	1.250	150
Sample_LPA01.mb.78cbin.1	96.72	1.898	106
Sample_LPA01.Cluster9248cbin.1	94.63	1.342	23
Sample_LPA01.Cluster10199cbin.1	100.00	0.632	80
Sample_LPA01.mb.74	99.19	1.075	87
Sample_LPA01.mb.34	97.27	2.040	162
Sample_LPA01.mb.67	95.65	1.449	59
Sample_LPA01.mb.15cbin.1	94.72	4.106	48
Sample_LPA01.mb.16	93.88	1.690	54
Sample_LPA01.Cluster1542cbin.1	95.52	0.464	140
Sample_LPA01.mb.23	92.45	0.377	30
Sample_LPA01.mb.20cbin.1	99.29	0.105	107
Sample_LPA01.mb.81cbin.1	97.94	0.000	30
Sample_LPA01.Cluster3577cbin.1	96.99	0.000	85
Sample_LPA01.mb.101	95.45	0.576	143
Sample_LPA01.mb.90	91.02	0.512	33
Sample_LPA02.Cluster15863	92.69	2.808	21
Sample_LPA02.Cluster6186	84.11	2.404	397
Sample_LPA02.Cluster7901	92.17	0.000	25
Sample_LPA02.Cluster4739cbin.1	94.39	2.531	265
Sample_LPA02.Cluster184cbin.1	94.29	0.671	79
Sample_LPA02.Cluster3382	97.31	0.000	11
Sample_LPA02.Cluster5710	96.98	0.898	25
Sample_LPA02.mb.63	93.53	0.000	6
Sample_LPA02.Cluster2508cbin.1	99.54	0.454	24
Sample_LPA02.mb.101	92.22	1.602	261
Sample_LPA02.mb.103cbin.1	95.94	0.370	63
Sample_LPA02.mb.116	89.28	1.454	154
Sample_LPA02.mb.104cbin.1	97.27	1.230	41
Sample_LPA02.mb.36	87.69	1.342	46
Sample_LPA02.mb.129	96.42	2.348	123
Sample_LPA02.mb.57	88.44	3.264	91
Sample_LPA02.mb.97	87.50	0.000	33
Sample_LPA02.mb.17	95.97	0.671	26
Sample_LPA02.mb.16	95.30	0.671	26
Sample_LPA02.mb.45cbin.1	99.53	0.245	33
Sample_LPA02.mb.39	96.61	2.415	97
Sample_LPA02.mb.44	98.79	2.227	115
Sample_LPA02.mb.88	89.70	3.225	198
Sample_LPA02.mb.52	86.03	3.081	115
Sample_LPA02.mb.28	85.82	0.000	60
Sample_LPA02.mb.81	94.29	1.342	154
Sample_LPA02.mb.83	97.09	0.000	33
Sample_LPA02.mb.59	97.46	0.194	68
Sample_LPA02.mb.91	99.32	1.006	33
Sample_LPA02.mb.80cbin.1	89.98	4.170	301
Sample_LPA02.mb.92	89.87	1.898	150
Sample_LPA02.Cluster2225cbin.1	97.75	0.782	276
Sample_LPA02.mb.50cbin.1	96.34	0.769	102
Sample_LPA03.mb.38	91.29	1.123	24
Sample_LPA03.mb.44	92.69	3.370	21
Sample_LPA03.mb.48	91.57	2.407	21

Sample_LPA03.mb.24	98.38	1.148	80
Sample_LPA03.mb.52	94.38	3.566	42
Sample_LPA03.mb.36	99.19	0.806	33
Sample_LPA03.mb.21	93.93	1.342	32
Sample_LPA03.mb.15	92.63	2.797	113
Sample_LPA03.mb.7	93.82	2.407	34
Sample_LPA03.Cluster989cbin.1	86.35	2.684	247
Sample_LPA03.Cluster907cbin.1	98.38	2.248	61
Sample_LPA03.mb.55cbin.1	97.36	0.000	28
Sample_LPA03.mb.66	92.74	1.677	208
Sample_LPA03.mb.42cbin.1	92.28	2.348	38
Sample_LPA03.Cluster245cbin.1	86.74	1.342	98
Sample_LPA03.mb.30	90.12	1.476	322
Sample_LPA03.mb.23cbin.1	84.53	4.535	629
Sample_LPA03.mb.67	85.79	3.523	47
Sample_LPA03.Cluster2348	92.70	0.000	25
Sample_LPA03.Cluster1473	97.95	0.000	17
Sample_LPA03.mb.10cbin.1	97.98	0.335	34
Sample_LPA03.mb.57cbin.1	99.32	0.671	65
Sample_LPA03.mb.53	93.19	1.360	123
Sample_LPA03.mb.40	99.90	1.136	20
Sample_LPA03.mb.17cbin.1	93.18	0.555	400
Sample_LPA03.Cluster5054cbin.1	91.28	4.216	593
Sample_LPA03.mb.50cbin.1	98.99	0.621	188
Sample_LPA04.Cluster13204	93.93	0.111	129
Sample_LPA04.Cluster7577	91.23	1.398	12
Sample_LPA04.mb.3	98.38	0.000	44
Sample_LPA04.Cluster724	96.77	0.000	23
Sample_LPA04.mb.54cbin.1	91.57	4.494	37
Sample_LPA04.Cluster12181cbin.1	84.27	0.671	332
Sample_LPA04.mb.23	91.21	0.840	109
Sample_LPA04.mb.52cbin.1	87.33	2.688	16
Sample_LPA04.Cluster612cbin.1	88.56	1.510	66
Sample_LPA04.mb.58	93.06	3.020	199
Sample_LPA04.mb.16	85.47	1.380	325
Sample_LPA04.mb.19	90.15	1.833	118
Sample_LPA04.mb.76	99.19	1.075	47
Sample_LPA04.mb.79cbin.1	80.70	0.000	16
Sample_LPA04.mb.51	95.95	0.000	98
Sample_LPA04.mb.75	92.05	1.677	173
Sample_LPA04.Cluster5076	95.98	1.347	77
Sample_LPA04.mb.63	81.36	1.812	286
Sample_LPA04.Cluster1103cbin.1	100.00	0.000	41
Sample_LPA04.mb.59	86.24	2.379	157
Sample_LPA04.mb.37	94.31	0.625	93
Sample_LPA04.Cluster3136	97.95	1.360	29
Sample_LPA04.mb.72	84.30	2.852	180
Sample_LPA04.mb.57	98.38	1.075	87
Sample_LPA04.Cluster12331	92.93	0.020	68
Sample_LPA04.mb.30	97.35	0.335	41
Sample_LPA04.mb.80	95.63	2.013	457
Sample_LPA04.mb.42cbin.1	98.63	1.183	194
Sample_LPA04.mb.87	86.82	0.671	95
Sample_LPA04.mb.70	92.08	1.342	230
Sample_LPA04.Cluster1856	90.38	1.442	32
Sample_LPA04.Cluster9609	97.73	0.000	16
Sample_LPA04.mb.92cbin.1	98.40	1.702	135
Sample_LPA04.Cluster8097cbin.1	99.41	0.487	178

Sample_LPA04.mb.95cbin.1	87.73	2.122	200
Sample_LPA04.mb.5cbin.1	90.92	2.307	365
Sample_LPA04.Cluster7322cbin.1	97.06	0.150	59
Sample_LPA04.mb.2	98.77	0.093	35
Sample_LPA05.mb.13	90.44	4.494	47
Sample_LPA05.mb.17	96.77	0.000	58
Sample_LPA05.Cluster12739cbin.1	88.45	4.362	375
Sample_LPA05.Cluster2994cbin.1	93.77	3.500	335
Sample_LPA05.Cluster147cbin.1	86.46	4.070	491
Sample_LPA05.Cluster1752cbin.1	86.31	2.908	265
Sample_LPA05.mb.3	99.30	1.165	113
Sample_LPA05.Cluster11436cbin.1	94.27	2.095	232
Sample_LPA05.mb.44cbin.1	95.58	3.691	227
Sample_LPA05.mb.16	90.54	2.533	297
Sample_LPA05.mb.38cbin.1	84.56	1.565	267
Sample_LPA05.mb.22	97.06	1.612	149
Sample_LPA05.Cluster10884	99.32	0.671	26
Sample_LPA05.mb.46	97.58	1.706	42
Sample_LPA05.Cluster625cbin.1	100.00	0.681	19
Sample_LPA05.Cluster223cbin.1	81.45	1.490	435
Sample_LPA05.Cluster8593	99.32	0.000	19
Sample_LPA05.mb.62cbin.1	97.31	0.671	141
Sample_LPA05.mb.50cbin.1	94.28	1.476	410
Sample_LPA05.mb.55	95.46	0.680	319
Sample_LPA05.mb.2	98.06	0.483	55
Sample_LPA05.mb.45cbin.1	89.35	2.270	168
Sample_LPA05.mb.59cbin.1	99.53	0.115	86
Sample_LPA05.mb.25	94.33	0.000	46
Sample_LPA05.mb.14cbin.1	96.30	1.115	169
Sample_LPA05.Cluster3925cbin.1	96.50	1.807	207
Sample_LPA05.mb.84	99.34	0.747	27
Sample_LPA08.mb.35cbin.1	81.03	0.000	105
Sample_LPA08.mb.19	93.73	2.684	134
Sample_LPA08.Cluster126cbin.1	95.98	0.000	150
Sample_LPA08.Cluster13cbin.1	88.81	2.796	148
Sample_LPA08.Cluster103	95.97	0.671	47
Sample_LPA08.mb.41	90.93	0.559	94
Sample_LPA08.mb.34cbin.1	90.93	0.000	27
Sample_LPA08.mb.43	92.61	2.348	56
Sample_LPA08.mb.65cbin.1	92.11	1.140	236
Sample_LPA08.Cluster1168cbin.1	94.15	0.961	21
Sample_LPA08.Cluster449	97.31	0.000	8
Sample_LPA08.Cluster3810cbin.1	99.98	1.497	27
Sample_LPA08.mb.30cbin.1	99.32	1.006	17
Sample_LPA08.Cluster818cbin.1	96.40	0.000	36
Sample_LPA08.mb.23cbin.1	96.71	0.545	111
Sample_LPA08.Cluster111cbin.1	97.11	0.000	36
Sample_LPA08.mb.63	94.07	1.739	141
Sample_LPA08.mb.67	90.70	0.000	117
Sample_LPA08.mb.17	97.26	0.000	44
Sample_LPA08.mb.37	99.05	0.000	82
Sample_LPA08.Cluster2626cbin.1	80.12	1.509	469
Sample_LPA08.mb.47	97.55	0.588	144
Sample_LPA08.Cluster24cbin.1	98.22	0.000	314
Sample_LPA08.mb.49	92.64	0.000	39
Sample_LPA08.Cluster5160cbin.1	99.03	4.347	82
Sample_LPA08.Cluster2236cbin.1	97.66	0.292	66
Sample_LPA08.Cluster548cbin.1	99.20	0.738	191

Sample_LPA08.Cluster1301	95.38	0.000	51
Sample_LPB01.Cluster213cbin.1	90.60	0.671	28
Sample_LPB01.mb.30cbin.1	88.23	4.087	308
Sample_LPB01.mb.21cbin.1	99.32	3.125	128
Sample_LPB01.mb.41	91.75	0.000	224
Sample_LPB01.Cluster1684cbin.1	90.44	0.100	110
Sample_LPB01.mb.63cbin.1	92.64	2.340	296
Sample_LPB01.mb.84	98.65	0.000	7
Sample_LPB01.Cluster3819cbin.1	97.10	2.705	68
Sample_LPB01.mb.27	93.75	1.442	24
Sample_LPB01.Cluster1675	96.60	0.000	21
Sample_LPB01.Cluster5666	85.50	2.367	25
Sample_LPB01.mb.22	94.63	0.000	24
Sample_LPB01.mb.59cbin.1	93.28	1.006	64
Sample_LPB01.mb.61cbin.1	94.55	4.761	240
Sample_LPB01.Cluster9682	92.95	4.026	25
Sample_LPB01.Cluster1317cbin.1	90.79	4.086	176
Sample_LPB01.Cluster7622cbin.1	97.10	0.724	86
Sample_LPB01.Cluster10035	97.46	0.632	89
Sample_LPB01.mb.18	96.79	0.000	28
Sample_LPB01.Cluster4014cbin.1	96.48	0.537	374
Sample_LPB01.Cluster1905cbin.1	93.95	0.000	114
Sample_LPB01.mb.95	95.51	2.358	199
Sample_LPB01.mb.42	92.29	4.377	226
Sample_LPB01.mb.94cbin.1	94.39	0.000	23
Sample_LPB01.Cluster1739cbin.1	97.56	0.792	188
Sample_LPB01.mb.75	97.45	3.365	42
Sample_LPB02.Cluster13993	98.65	0.000	12
Sample_LPB02.Cluster7087	92.17	0.000	27
Sample_LPB02.Cluster1263cbin.1	90.53	1.342	258
Sample_LPB02.Cluster10625cbin.1	96.64	0.000	44
Sample_LPB02.mb.18cbin.1	88.48	0.000	15
Sample_LPB02.Cluster215cbin.1	82.82	3.061	372
Sample_LPB02.Cluster3912cbin.1	97.03	0.370	54
Sample_LPB02.Cluster10803cbin.1	91.98	1.677	23
Sample_LPB02.Cluster440	94.13	2.800	26
Sample_LPB02.Cluster5269cbin.1	86.32	2.815	253
Sample_LPB02.Cluster12684	96.17	0.000	33
Sample_LPB02.Cluster4672	90.32	0.289	59
Sample_LPB02.mb.116	97.31	0.000	12
Sample_LPB02.Cluster13006cbin.1	97.27	3.243	67
Sample_LPB02.Cluster5645cbin.1	88.80	0.000	54
Sample_LPB02.mb.117	89.55	3.970	157
Sample_LPB02.mb.32	96.25	0.671	121
Sample_LPB02.mb.28cbin.1	97.67	1.898	38
Sample_LPB02.mb.111	95.07	0.000	25
Sample_LPB02.Cluster9005cbin.1	93.58	1.702	86
Sample_LPB02.mb.126	98.07	0.961	54
Sample_LPB02.mb.129	99.32	1.006	38
Sample_LPB02.mb.56	88.79	1.026	26
Sample_LPB02.Cluster4541cbin.1	93.27	0.806	419
Sample_LPB02.mb.48	80.11	4.919	284
Sample_LPB02.mb.134	94.63	0.000	26
Sample_LPB02.mb.33cbin.1	95.80	0.299	33
Sample_LPB02.mb.47	98.65	0.335	38
Sample_LPB02.mb.67	93.95	2.953	134
Sample_LPB02.Cluster5495	96.00	0.524	78
Sample_LPB02.mb.89	84.22	1.647	69

Sample_LPB02.mb.86	96.08	0.000	90
Sample_LPB02.mb.26cbin.1	91.25	0.543	131
Sample_LPB02.mb.96cbin.1	96.42	0.000	162
Sample_LPB02.mb.57cbin.1	89.24	0.000	45
Sample_LPB02.mb.49	95.97	2.013	41
Sample_LPB02.mb.51	88.60	4.113	206
Sample_LPB02.mb.4	88.51	0.000	78
Sample_LPB02.mb.68	98.55	2.403	49
Sample_LPB02.mb.62	90.83	2.485	235
Sample_LPB02.mb.83	93.50	3.508	269
Sample_LPB03.mb.17	87.07	1.123	11
Sample_LPB03.mb.18	93.82	1.284	35
Sample_LPB03.mb.24	87.93	2.407	21
Sample_LPB03.Cluster8614cbin.1	80.74	2.887	329
Sample_LPB03.Cluster557cbin.1	98.38	4.032	37
Sample_LPB03.mb.38	91.93	1.881	91
Sample_LPB03.Cluster949cbin.1	98.60	0.466	96
Sample_LPB03.mb.4	83.17	0.671	88
Sample_LPB03.Cluster155	87.61	1.677	189
Sample_LPB03.mb.13	90.60	0.671	48
Sample_LPB03.mb.71cbin.1	92.41	1.123	23
Sample_LPB03.mb.61	91.66	1.638	131
Sample_LPB03.mb.53cbin.1	87.25	2.796	269
Sample_LPB03.Cluster637cbin.1	85.65	4.697	41
Sample_LPB03.Cluster4384cbin.1	82.23	2.705	376
Sample_LPB03.mb.39	99.19	0.115	26
Sample_LPB03.mb.74	94.38	3.370	41
Sample_LPB03.mb.48	98.65	0.671	98
Sample_LPB03.mb.27	100.00	0.806	23
Sample_LPB03.Cluster2870	95.70	0.000	21
Sample_LPB03.mb.79	92.59	1.342	32
Sample_LPB03.Cluster2773cbin.1	96.60	0.000	24
Sample_LPB03.mb.60	93.12	3.796	262
Sample_LPB03.Cluster1336	97.95	0.000	31
Sample_LPB03.mb.28	98.65	1.738	115
Sample_LPB03.Cluster466	98.66	2.543	25
Sample_LPB03.mb.46	88.12	0.894	145
Sample_LPB03.mb.75	91.15	0.357	134
Sample_LPB03.mb.83	82.36	4.530	243
Sample_LPB03.Cluster375	81.49	0.507	52
Sample_LPB03.mb.82cbin.1	84.66	2.416	235
Sample_LPB03.Cluster632	99.90	0.909	15
Sample_LPB03.mb.93	96.77	0.000	42
Sample_LPB03.mb.40	87.99	3.210	300
Sample_LPB03.mb.31	95.35	2.486	93
Sample_LPB03.mb.55cbin.1	96.91	0.335	49
Sample_LPB03.mb.86	93.28	0.894	335
Sample_LPB03.mb.78	94.23	1.201	10
Sample_LPB03.mb.50	98.11	1.103	60
Sample_LPB03.mb.45cbin.1	93.64	0.788	345
Sample_LPB03.mb.47	99.25	0.929	99
Sample_LPB03.Cluster4744cbin.1	99.38	0.808	228
Sample_LPB04.Cluster13416cbin.1	89.88	4.654	105
Sample_LPB04.Cluster2924cbin.1	96.43	0.806	206
Sample_LPB04.mb.12	95.74	0.671	29
Sample_LPB04.Cluster395	87.24	0.671	58
Sample_LPB04.mb.54	85.59	1.612	332
Sample_LPB04.mb.25	99.26	0.000	34

Sample_LPB04.mb.41	93.06	0.000	33
Sample_LPB04.Cluster591cbin.1	82.66	1.520	352
Sample_LPB04.Cluster636	91.10	4.026	63
Sample_LPB04.mb.60	99.19	1.075	49
Sample_LPB04.mb.49	82.71	0.671	109
Sample_LPB04.mb.27	94.29	0.671	19
Sample_LPB04.Cluster6113	96.64	0.000	23
Sample_LPB04.mb.36cbin.1	95.04	4.113	314
Sample_LPB04.mb.56cbin.1	87.55	4.697	33
Sample_LPB04.Cluster4480	93.62	0.000	8
Sample_LPB04.Cluster8893	99.51	0.480	14
Sample_LPB04.Cluster4cbin.1	80.73	1.020	503
Sample_LPB04.mb.32cbin.1	98.65	1.006	19
Sample_LPB04.Cluster13352	100.00	0.000	21
Sample_LPB04.Cluster2894cbin.1	91.60	2.344	242
Sample_LPB04.Cluster24	97.21	1.873	148
Sample_LPB04.Cluster12970cbin.1	98.32	0.000	139
Sample_LPB04.Cluster2445	97.95	1.360	34
Sample_LPB04.mb.47	80.57	0.671	398
Sample_LPB04.mb.76cbin.1	99.30	1.864	99
Sample_LPB04.mb.33	93.78	1.522	152
Sample_LPB04.mb.6cbin.1	81.65	0.000	41
Sample_LPB04.mb.53	92.56	2.419	51
Sample_LPB04.mb.61	92.39	0.625	104
Sample_LPB04.Cluster10566	99.62	0.000	15
Sample_LPB04.mb.29cbin.1	94.15	1.454	171
Sample_LPB04.mb.67	94.44	2.514	131
Sample_LPB04.Cluster7411cbin.1	83.06	0.806	680
Sample_LPB04.mb.68	95.75	2.173	156
Sample_LPB04.mb.69cbin.1	90.97	1.814	55
Sample_LPB04.mb.88	84.35	3.306	425
Sample_LPB04.mb.73	86.54	4.474	275
Sample_LPB04.Cluster7566	84.87	1.602	148
Sample_LPB04.mb.90	97.43	0.360	22
Sample_LPB04.mb.64	98.77	3.541	36
Sample_LPB04.mb.18cbin.1	95.31	1.730	222
Sample_LPB04.mb.51cbin.1	83.70	0.314	81
Sample_LPB04.mb.9cbin.1	98.65	0.000	21
Sample_LPB04.mb.91cbin.1	98.65	0.000	45
Sample_LPB04.mb.7	97.74	2.334	211
Sample_LPB04.mb.100cbin.1	90.27	4.696	230
Sample_LPB05.Cluster2433cbin.1	85.02	0.000	250
Sample_LPB05.Cluster13939	96.72	0.111	19
Sample_LPB05.Cluster4858	92.37	0.671	23
Sample_LPB05.Cluster4851cbin.1	91.59	0.000	198
Sample_LPB05.mb.12cbin.1	92.69	4.494	15
Sample_LPB05.mb.112	96.77	1.075	63
Sample_LPB05.Cluster7796cbin.1	95.14	3.006	265
Sample_LPB05.Cluster6861	91.21	0.675	11
Sample_LPB05.Cluster12286cbin.1	97.15	1.265	189
Sample_LPB05.mb.3	96.50	0.000	93
Sample_LPB05.mb.54cbin.1	94.89	0.000	136
Sample_LPB05.mb.47	97.76	0.000	71
Sample_LPB05.Cluster11235	99.32	0.671	23
Sample_LPB05.mb.50	90.79	2.714	281
Sample_LPB05.Cluster7261	99.98	1.497	22
Sample_LPB05.mb.109cbin.1	87.91	4.853	236
Sample_LPB05.mb.16cbin.1	90.60	3.355	42

Sample_LPB05.Cluster3159	97.59	0.961	20
Sample_LPB05.mb.100	88.92	0.000	130
Sample_LPB05.mb.66	91.57	0.561	27
Sample_LPB05.mb.37cbin.1	93.95	0.335	140
Sample_LPB05.mb.17	95.25	1.265	101
Sample_LPB05.Cluster3708cbin.1	98.65	0.000	24
Sample_LPB05.Cluster9022	95.97	2.013	20
Sample_LPB05.Cluster10054	99.32	0.000	18
Sample_LPB05.Cluster4298cbin.1	97.95	0.000	28
Sample_LPB05.mb.117	97.85	2.745	78
Sample_LPB05.mb.119	94.63	0.671	69
Sample_LPB05.Cluster5602	97.70	4.310	80
Sample_LPB05.mb.29cbin.1	96.92	2.512	68
Sample_LPB05.Cluster11890cbin.1	97.73	1.886	197
Sample_LPB05.mb.69	87.24	0.671	40
Sample_LPB05.mb.62	93.99	0.000	40
Sample_LPB05.mb.26	97.31	1.342	67
Sample_LPB05.mb.96cbin.1	93.06	0.000	47
Sample_LPB05.mb.71	88.36	1.174	45
Sample_LPB05.mb.65	88.63	1.209	134
Sample_LPB05.mb.7	84.25	2.300	342
Sample_LPB05.mb.82	94.29	0.335	70
Sample_LPB05.mb.84	85.41	1.682	82
Sample_LPB05.Cluster6812cbin.1	99.10	3.831	63
Sample_LPB05.mb.75	98.65	0.671	52
Sample_LPB05.mb.93cbin.1	98.40	2.937	128
Sample_LPB05.mb.94	97.86	1.205	230
Sample_LPB05.mb.64cbin.1	87.94	2.820	418
Sample_LPB05.mb.95	87.12	4.368	268
Sample_LPB05.mb.48cbin.1	99.37	0.314	95
Sample_LPB08.Cluster5246cbin.1	97.09	0.671	62
Sample_LPB08.mb.71	92.13	1.845	21
Sample_LPB08.Cluster4938	95.30	3.579	141
Sample_LPB08.mb.16	98.65	0.000	52
Sample_LPB08.mb.20	90.31	2.427	157
Sample_LPB08.mb.57	90.93	0.671	23
Sample_LPB08.Cluster730	92.22	0.961	21
Sample_LPB08.mb.49	86.62	3.533	348
Sample_LPB08.Cluster4192	86.84	1.006	16
Sample_LPB08.mb.28cbin.1	93.62	0.671	96
Sample_LPB08.Cluster14cbin.1	94.49	1.381	69
Sample_LPB08.mb.33	92.75	2.898	60
Sample_LPB08.mb.67	93.68	2.242	152
Sample_LPB08.mb.13cbin.1	99.98	1.497	26
Sample_LPB08.mb.9cbin.1	89.93	1.342	34
Sample_LPB08.mb.12	96.40	0.598	89
Sample_LPB08.mb.3	96.15	0.000	35
Sample_LPB08.mb.10cbin.1	88.09	4.219	145
Sample_LPB08.Cluster93	100.00	1.818	26
Sample_LPB08.Cluster6413cbin.1	89.60	0.671	370
Sample_LPB08.mb.41cbin.1	80.75	0.000	43
Sample_LPB08.Cluster4247	99.51	0.000	61
Sample_LPB08.mb.6	93.25	2.399	518
Sample_LPB08.mb.25	99.24	0.125	90
Sample_LPB08.Cluster4577cbin.1	99.41	0.000	82
Sample_LPB08.mb.15cbin.1	99.64	0.105	82
Sample_LPB08.mb.36cbin.1	96.57	0.000	265
Sample_LPB08.Cluster3074cbin.1	97.12	1.189	58

Sample_LPB08.mb.60cbin.1	98.84	0.384	50
Sample_LPB08.mb.8	85.24	1.610	47
Sample_LPC01.Cluster3588	92.28	0.000	37
Sample_LPC01.Cluster12	88.74	0.671	209
Sample_LPC01.mb.111	98.65	0.000	38
Sample_LPC01.Cluster2751	97.67	0.632	36
Sample_LPC01.mb.112	97.79	0.000	31
Sample_LPC01.mb.119	97.48	0.000	102
Sample_LPC01.mb.116	86.60	1.510	208
Sample_LPC01.Cluster1033cbin.1	91.82	0.961	23
Sample_LPC01.mb.68	80.71	0.892	88
Sample_LPC01.mb.30cbin.1	86.93	1.235	368
Sample_LPC01.mb.2cbin.1	94.63	4.697	51
Sample_LPC01.Cluster396cbin.1	87.27	0.850	191
Sample_LPC01.Cluster1744	97.27	0.671	25
Sample_LPC01.mb.109cbin.1	95.56	0.021	68
Sample_LPC01.Cluster11550cbin.1	90.83	0.167	186
Sample_LPC01.Cluster5424	96.57	0.000	23
Sample_LPC01.mb.28cbin.1	95.56	2.215	121
Sample_LPC01.mb.124cbin.1	95.06	2.721	237
Sample_LPC01.Cluster6136	95.97	3.140	69
Sample_LPC01.mb.105cbin.1	94.15	0.966	42
Sample_LPC01.mb.90	91.27	1.342	53
Sample_LPC01.Cluster10977cbin.1	99.36	1.265	116
Sample_LPC01.mb.118	97.86	0.020	28
Sample_LPC01.mb.96	98.43	1.342	63
Sample_LPC01.mb.110cbin.1	88.67	0.105	214
Sample_LPC01.mb.61	94.30	2.061	155
Sample_LPC01.mb.45	89.93	0.000	31
Sample_LPC01.mb.41	97.55	1.774	72
Sample_LPC01.mb.76cbin.1	94.71	2.830	262
Sample_LPC01.mb.89cbin.1	92.61	0.335	69
Sample_LPC01.mb.91	89.30	3.381	169
Sample_LPC01.mb.128cbin.1	93.15	2.611	82
Sample_LPC01.mb.67cbin.1	86.21	1.143	396
Sample_LPC01.mb.34cbin.1	96.80	0.557	94
Sample_LPC01.mb.27cbin.1	97.56	0.769	100
Sample_LPC02.Cluster15787	90.66	0.000	29
Sample_LPC02.Cluster15690	98.66	0.111	9
Sample_LPC02.Cluster14689	98.65	0.671	11
Sample_LPC02.Cluster9279cbin.1	96.73	2.483	130
Sample_LPC02.Cluster130cbin.1	93.45	1.610	59
Sample_LPC02.mb.123	91.58	1.342	35
Sample_LPC02.Cluster3115	98.32	0.671	27
Sample_LPC02.Cluster3628	93.28	0.000	10
Sample_LPC02.Cluster8220cbin.1	91.94	1.006	29
Sample_LPC02.mb.24	92.74	0.342	96
Sample_LPC02.Cluster3411cbin.1	93.04	0.186	238
Sample_LPC02.Cluster4862	97.27	0.335	20
Sample_LPC02.Cluster12100	98.65	0.000	28
Sample_LPC02.Cluster2482	97.95	0.680	23
Sample_LPC02.mb.52	89.32	0.000	30
Sample_LPC02.Cluster596cbin.1	86.80	2.534	211
Sample_LPC02.mb.122	96.76	1.481	52
Sample_LPC02.mb.23	90.93	0.671	59
Sample_LPC02.Cluster9367cbin.1	94.40	0.000	27
Sample_LPC02.mb.17	92.11	0.335	66
Sample_LPC02.mb.47	87.01	1.923	305

Sample_LPC02.mb.21	85.52	3.355	231
Sample_LPC02.mb.12	100.00	0.000	15
Sample_LPC02.mb.125	99.19	0.961	26
Sample_LPC02.mb.109	96.42	0.671	86
Sample_LPC02.mb.15	89.26	0.335	34
Sample_LPC02.mb.76	86.34	0.335	251
Sample_LPC02.mb.70cbin.1	92.17	0.000	30
Sample_LPC02.mb.60	95.07	0.901	172
Sample_LPC02.Cluster5884	92.23	0.000	86
Sample_LPC02.mb.69cbin.1	89.76	1.898	129
Sample_LPC02.mb.127	93.34	1.091	257
Sample_LPC02.mb.80cbin.1	94.74	2.013	112
Sample_LPC02.mb.7cbin.1	91.61	2.908	148
Sample_LPC02.mb.58	89.66	2.708	121
Sample_LPC02.Cluster8316	95.00	1.282	52
Sample_LPC02.mb.87	100.00	0.480	33
Sample_LPC02.mb.90cbin.1	99.11	0.360	43
Sample_LPC02.mb.51	96.02	0.000	122
Sample_LPC02.mb.79	97.75	2.641	193
Sample_LPC02.mb.99	96.43	0.512	54
Sample_LPC03.Cluster13638	98.65	0.000	8
Sample_LPC03.Cluster353cbin.1	96.50	1.515	88
Sample_LPC03.Cluster11360cbin.1	95.30	0.671	29
Sample_LPC03.mb.122	97.94	0.000	58
Sample_LPC03.Cluster8563	85.45	0.671	10
Sample_LPC03.Cluster8725	92.17	0.671	21
Sample_LPC03.Cluster12660cbin.1	98.65	3.020	149
Sample_LPC03.Cluster211cbin.1	87.24	1.342	115
Sample_LPC03.Cluster213cbin.1	84.33	2.404	91
Sample_LPC03.Cluster1592cbin.1	93.59	4.219	146
Sample_LPC03.Cluster1220cbin.1	89.05	4.026	107
Sample_LPC03.mb.128cbin.1	84.61	0.335	44
Sample_LPC03.mb.3	97.84	0.000	44
Sample_LPC03.mb.35	100.00	0.111	42
Sample_LPC03.Cluster9996cbin.1	80.99	3.511	530
Sample_LPC03.mb.29cbin.1	86.26	2.348	206
Sample_LPC03.Cluster5010cbin.1	99.03	1.442	14
Sample_LPC03.Cluster2175	97.95	0.680	23
Sample_LPC03.mb.94	80.34	0.000	140
Sample_LPC03.Cluster1576cbin.1	98.93	0.000	54
Sample_LPC03.mb.33cbin.1	83.06	0.335	178
Sample_LPC03.mb.19	95.96	0.953	173
Sample_LPC03.mb.124	97.31	0.000	14
Sample_LPC03.mb.111cbin.1	85.23	2.684	38
Sample_LPC03.Cluster11cbin.1	86.70	0.698	465
Sample_LPC03.mb.40	90.60	0.671	148
Sample_LPC03.Cluster653	88.54	1.439	14
Sample_LPC03.mb.108	96.28	0.240	57
Sample_LPC03.mb.51cbin.1	81.02	1.510	121
Sample_LPC03.Cluster4989cbin.1	94.25	0.675	44
Sample_LPC03.mb.56	92.36	4.642	373
Sample_LPC03.mb.126	97.98	1.342	20
Sample_LPC03.mb.117cbin.1	98.86	0.377	34
Sample_LPC03.mb.50cbin.1	97.31	1.342	90
Sample_LPC03.mb.28	86.23	2.093	47
Sample_LPC03.mb.24cbin.1	84.15	0.880	56
Sample_LPC03.mb.9cbin.1	96.42	1.342	67
Sample_LPC03.mb.71	95.19	1.923	41

Sample_LPC03.mb.90cbin.1	90.22	1.425	193
Sample_LPC03.mb.86cbin.1	94.25	0.000	62
Sample_LPC03.mb.11	91.03	3.738	173
Sample_LPC03.mb.77	83.54	1.243	100
Sample_LPC04.Cluster12914	91.33	0.111	145
Sample_LPC04.Cluster148	85.86	1.612	24
Sample_LPC04.Cluster8345	94.38	1.398	4
Sample_LPC04.Cluster6011cbin.1	92.93	2.237	24
Sample_LPC04.mb.111	89.51	0.000	61
Sample_LPC04.mb.26	90.96	1.565	230
Sample_LPC04.mb.12	89.70	0.000	29
Sample_LPC04.mb.101cbin.1	86.45	1.526	133
Sample_LPC04.mb.106	90.51	1.236	60
Sample_LPC04.mb.30	96.90	1.209	355
Sample_LPC04.mb.104	88.99	2.074	176
Sample_LPC04.Cluster1226cbin.1	89.26	0.838	90
Sample_LPC04.mb.57	91.29	1.123	36
Sample_LPC04.mb.67	94.94	2.407	21
Sample_LPC04.Cluster6453	91.96	0.898	75
Sample_LPC04.mb.70	90.44	2.247	31
Sample_LPC04.mb.22	97.58	0.806	61
Sample_LPC04.mb.51cbin.1	93.88	0.699	54
Sample_LPC04.Cluster1402cbin.1	100.00	0.000	38
Sample_LPC04.Cluster10775	98.65	1.006	20
Sample_LPC04.mb.47	85.32	4.215	279
Sample_LPC04.Cluster12998	100.00	0.000	22
Sample_LPC04.mb.55	97.79	0.000	60
Sample_LPC04.mb.43cbin.1	87.24	2.768	95
Sample_LPC04.Cluster10936cbin.1	99.32	0.000	20
Sample_LPC04.Cluster554cbin.1	96.94	2.071	243
Sample_LPC04.mb.6	94.59	1.342	169
Sample_LPC04.mb.5	93.59	0.671	199
Sample_LPC04.Cluster2788	97.95	1.360	34
Sample_LPC04.mb.65cbin.1	96.42	0.335	106
Sample_LPC04.mb.20	93.73	0.671	227
Sample_LPC04.mb.58	86.57	0.671	40
Sample_LPC04.mb.85	97.58	1.075	45
Sample_LPC04.mb.32	98.63	1.677	37
Sample_LPC04.mb.89	81.45	0.806	43
Sample_LPC04.mb.40	90.86	1.006	265
Sample_LPC04.mb.19cbin.1	95.30	0.000	38
Sample_LPC04.mb.99	90.45	0.000	224
Sample_LPC04.mb.90	96.40	0.268	79
Sample_LPC04.Cluster1843cbin.1	98.79	0.507	40
Sample_LPC04.mb.74	93.71	2.702	134
Sample_LPC04.mb.8	81.18	4.250	150
Sample_LPC04.Cluster6674cbin.1	91.95	1.075	482
Sample_LPC04.mb.95	95.97	0.671	25
Sample_LPC04.Cluster9196	99.24	0.000	25
Sample_LPC04.mb.91cbin.1	94.25	3.624	43
Sample_LPC04.mb.105	91.40	0.833	327
Sample_LPC04.mb.80cbin.1	99.10	0.335	35
Sample_LPC04.mb.87	95.75	0.496	59
Sample_LPC04.mb.28cbin.1	93.46	0.576	199
Sample_LPC04.mb.69	95.90	0.000	105
Sample_LPC04.mb.54	95.70	1.774	115
Sample_LPC05.Cluster7047	97.31	0.000	23
Sample_LPC05.mb.119cbin.1	88.41	3.137	188

Sample_LPC05.mb.14cbin.1	99.32	1.342	72
Sample_LPC05.Cluster11595cbin.1	96.73	0.724	29
Sample_LPC05.Cluster12425	95.97	0.335	38
Sample_LPC05.Cluster11892cbin.1	99.98	2.095	22
Sample_LPC05.mb.16cbin.1	99.77	0.671	64
Sample_LPC05.Cluster10737	95.30	0.000	22
Sample_LPC05.Cluster8509	95.97	0.244	19
Sample_LPC05.Cluster141cbin.1	97.73	2.348	143
Sample_LPC05.mb.69cbin.1	97.84	0.000	54
Sample_LPC05.mb.102	96.63	0.961	35
Sample_LPC05.Cluster9730	87.32	0.025	81
Sample_LPC05.mb.100	96.64	0.335	17
Sample_LPC05.Cluster12574cbin.1	94.63	0.000	43
Sample_LPC05.Cluster9183	97.76	0.000	26
Sample_LPC05.mb.113cbin.1	98.55	1.682	92
Sample_LPC05.mb.24	94.21	0.680	192
Sample_LPC05.Cluster12851cbin.1	97.73	0.000	90
Sample_LPC05.Cluster7412cbin.1	96.83	3.164	80
Sample_LPC05.mb.117	87.14	1.342	279
Sample_LPC05.Cluster13262cbin.1	87.26	2.777	637
Sample_LPC05.mb.27cbin.1	93.28	1.342	67
Sample_LPC05.mb.52	88.88	3.716	316
Sample_LPC05.mb.2	91.36	2.223	243
Sample_LPC05.Cluster4101cbin.1	88.16	3.810	130
Sample_LPC05.Cluster726cbin.1	100.00	0.000	140
Sample_LPC05.mb.76cbin.1	85.51	0.000	43
Sample_LPC05.mb.49	93.22	4.459	173
Sample_LPC05.mb.95cbin.1	93.73	2.348	40
Sample_LPC05.mb.85	87.97	1.985	366
Sample_LPC05.mb.112	96.98	0.000	91
Sample_LPC05.mb.78	85.11	2.168	90
Sample_LPC05.mb.20	91.94	0.700	85
Sample_LPC05.Cluster10950cbin.1	95.84	2.391	227
Sample_LPC05.mb.25cbin.1	97.76	0.371	107
Sample_LPC08.mb.20cbin.1	81.89	0.000	243
Sample_LPC08.mb.5	92.13	1.845	20
Sample_LPC08.mb.17	94.50	0.000	187
Sample_LPC08.mb.54cbin.1	95.74	0.671	63
Sample_LPC08.mb.13	87.58	0.000	21
Sample_LPC08.mb.55cbin.1	98.65	2.013	111
Sample_LPC08.mb.15	92.51	1.549	95
Sample_LPC08.mb.41cbin.1	92.46	0.480	20
Sample_LPC08.mb.10	99.51	1.449	75
Sample_LPC08.Cluster2113cbin.1	99.98	1.497	25
Sample_LPC08.Cluster5547	84.69	1.094	257
Sample_LPC08.Cluster147cbin.1	97.00	0.149	199
Sample_LPC08.Cluster4552cbin.1	99.32	1.006	17
Sample_LPC08.mb.26	98.11	0.000	106
Sample_LPC08.Cluster227	98.63	0.000	19
Sample_LPC08.mb.31cbin.1	98.07	0.000	36
Sample_LPC08.mb.42	90.92	1.183	570
Sample_LPC08.mb.44cbin.1	88.49	0.000	38
Sample_LPC08.Cluster4945cbin.1	99.51	0.483	93
Sample_LPC08.Cluster5239cbin.1	87.14	0.806	520
Sample_LPC08.mb.1cbin.1	98.49	0.514	173
Sample_LPC08.mb.33cbin.1	98.50	1.265	236
Sample_LPC08.mb.49	94.93	0.584	64
Sample_LPC08.Cluster2875cbin.1	96.83	2.998	48

Sample_LPC08.Cluster1848cbin.1	98.84	0.000	53
Sample_SCA55.Cluster12046	91.11	0.111	122
Sample_SCA55.mb.104cbin.1	82.52	3.225	119
Sample_SCA55.Cluster2874	90.61	0.000	29
Sample_SCA55.Cluster7572cbin.1	94.91	0.223	196
Sample_SCA55.Cluster4326cbin.1	87.05	0.000	49
Sample_SCA55.Cluster3804cbin.1	95.67	2.215	249
Sample_SCA55.Cluster3130cbin.1	94.60	2.142	50
Sample_SCA55.Cluster2493	96.97	0.000	11
Sample_SCA55.mb.44	97.60	0.000	15
Sample_SCA55.Cluster10485	98.50	1.042	107
Sample_SCA55.mb.27	84.88	1.698	271
Sample_SCA55.mb.23	84.00	0.000	39
Sample_SCA55.Cluster11573cbin.1	98.79	0.294	52
Sample_SCA55.Cluster6699	97.09	0.000	38
Sample_SCA55.Cluster790	99.43	0.077	21
Sample_SCA55.Cluster12012	90.64	0.000	21
Sample_SCA55.mb.7cbin.1	93.95	0.000	22
Sample_SCA55.mb.4cbin.1	93.70	0.000	71
Sample_SCA55.Cluster8267	98.95	0.483	61
Sample_SCA55.mb.52cbin.1	96.77	2.419	78
Sample_SCA55.mb.97	89.74	0.854	7
Sample_SCA55.mb.38	88.18	3.140	157
Sample_SCA55.mb.21cbin.1	85.14	1.911	311
Sample_SCA55.mb.85	86.53	0.671	104
Sample_SCA55.mb.45cbin.1	98.63	4.026	37
Sample_SCA55.mb.60	91.89	2.721	89
Sample_SCA55.mb.87	98.65	0.335	48
Sample_SCA55.mb.110cbin.1	98.06	3.582	179
Sample_SCA55.mb.95	95.10	2.040	112
Sample_SCA55.mb.99cbin.1	96.41	2.098	44
Sample_SCA58.Cluster16595	87.35	1.123	165
Sample_SCA58.Cluster16706cbin.1	94.94	2.407	29
Sample_SCA58.Cluster1102	88.70	1.075	39
Sample_SCA58.Cluster4228cbin.1	95.39	1.342	64
Sample_SCA58.mb.102	87.13	0.000	38
Sample_SCA58.Cluster4693	100.00	0.115	8
Sample_SCA58.Cluster15781	91.04	1.841	107
Sample_SCA58.mb.118cbin.1	92.26	4.026	47
Sample_SCA58.Cluster605	91.94	1.649	72
Sample_SCA58.mb.25cbin.1	92.33	0.806	12
Sample_SCA58.Cluster1069cbin.1	86.25	2.494	341
Sample_SCA58.Cluster6503cbin.1	95.07	0.000	64
Sample_SCA58.mb.121	84.66	4.697	140
Sample_SCA58.Cluster13327	99.32	1.006	19
Sample_SCA58.Cluster10143	87.56	2.013	23
Sample_SCA58.Cluster1691	97.95	0.000	27
Sample_SCA58.mb.20	80.16	2.593	261
Sample_SCA58.Cluster10382cbin.1	97.31	0.000	68
Sample_SCA58.mb.5	95.96	1.148	169
Sample_SCA58.Cluster864	96.25	0.000	146
Sample_SCA58.mb.129cbin.1	85.85	1.363	63
Sample_SCA58.mb.31cbin.1	81.60	2.430	368
Sample_SCA58.mb.35	99.30	1.515	93
Sample_SCA58.mb.4cbin.1	88.81	0.000	64
Sample_SCA58.mb.107	96.63	0.961	40
Sample_SCA58.mb.80	92.41	2.247	29

Sample_SCA58.mb.43	81.06	1.149	87
Sample_SCA58.mb.15cbin.1	90.86	1.442	33
Sample_SCA58.mb.120cbin.1	96.60	0.671	29
Sample_SCA58.mb.46	91.61	1.006	272
Sample_SCA58.Cluster11257	97.73	0.000	21
Sample_SCA58.mb.103	91.88	0.480	23
Sample_SCA58.mb.77	100.00	0.000	56
Sample_SCA58.mb.27	90.02	2.040	185
Sample_SCA58.mb.63cbin.1	91.27	4.194	48
Sample_SCA58.mb.51	81.17	1.490	405
Sample_SCA58.Cluster8627cbin.1	97.21	1.612	276
Sample_SCA58.mb.123	96.29	0.487	162
Sample_SCA58.mb.55cbin.1	99.19	3.225	145
Sample_SCA58.Cluster8567	89.23	0.897	39
Sample_SCA58.Cluster8833	98.84	0.000	50
Sample_SCA58.mb.91cbin.1	92.56	2.348	156
Sample_SCA58.mb.56cbin.1	100.00	2.176	47
Sample_SCA58.mb.117cbin.1	85.08	4.584	424
Sample_SCA58.mb.86	98.07	0.961	21
Sample_SCA58.mb.38	88.97	2.230	146
Sample_SCA58.Cluster8815	93.69	4.902	152
Sample_SCA58.mb.135	98.09	0.870	123
Sample_SCA59.Cluster5865cbin.1	88.23	1.342	28
Sample_SCA59.Cluster17127	97.60	0.800	41
Sample_SCA59.mb.106	81.17	2.247	17
Sample_SCA59.mb.124	91.57	1.284	32
Sample_SCA59.Cluster179	98.32	1.342	35
Sample_SCA59.Cluster9717	99.19	0.115	16
Sample_SCA59.Cluster17197cbin.1	100.00	0.000	21
Sample_SCA59.Cluster10905	95.30	0.000	20
Sample_SCA59.Cluster12937cbin.1	99.32	0.671	25
Sample_SCA59.Cluster4667cbin.1	93.82	0.021	79
Sample_SCA59.Cluster12108	92.49	0.523	204
Sample_SCA59.Cluster3650cbin.1	97.27	0.671	29
Sample_SCA59.Cluster3610cbin.1	81.66	0.200	194
Sample_SCA59.Cluster227	95.91	0.340	218
Sample_SCA59.mb.120cbin.1	82.36	4.570	212
Sample_SCA59.mb.133cbin.1	91.94	3.020	32
Sample_SCA59.mb.26	99.19	1.612	41
Sample_SCA59.mb.15	96.64	1.342	61
Sample_SCA59.Cluster9650cbin.1	89.93	0.671	86
Sample_SCA59.Cluster6328cbin.1	93.54	3.810	342
Sample_SCA59.mb.50	99.19	2.284	135
Sample_SCA59.mb.102	97.31	0.000	103
Sample_SCA59.mb.24	84.61	0.000	196
Sample_SCA59.mb.30cbin.1	98.65	3.803	166
Sample_SCA59.mb.121	91.54	1.759	31
Sample_SCA59.mb.18	96.95	0.256	85
Sample_SCA59.mb.65	98.38	1.075	96
Sample_SCA59.mb.117cbin.1	95.62	0.681	196
Sample_SCA59.mb.51	85.33	0.671	48
Sample_SCA59.mb.48cbin.1	91.35	1.565	123
Sample_SCA59.mb.115	94.75	4.093	175
Sample_SCA59.mb.46	92.19	0.126	196
Sample_SCA59.Cluster7336	97.31	1.075	93
Sample_SCA59.mb.126	91.13	1.754	106
Sample_SCA59.mb.81	84.09	3.691	108
Sample_SCA59.mb.36	98.65	1.677	23

Sample_SCA59.mb.130	100.00	0.000	74
Sample_SCA59.Cluster8336	99.23	0.384	48
Sample_SCA59.Cluster5138cbin.1	97.55	0.987	400
Sample_SCA59.mb.69cbin.1	93.43	3.629	322
Sample_SCA59.mb.52	95.30	1.342	169
Sample_SCA59.mb.88	89.42	0.961	75
Sample_SCA59.mb.86	100.00	0.000	8
Sample_SCA59.mb.59cbin.1	90.91	0.788	461
Sample_SCA59.mb.90cbin.1	91.78	0.929	283
Sample_SCA60.Cluster15187	93.82	0.000	179
Sample_SCA60.mb.102	95.30	0.671	48
Sample_SCA60.mb.17cbin.1	95.80	1.864	87
Sample_SCA60.mb.3	84.89	0.671	90
Sample_SCA60.Cluster9736cbin.1	98.43	0.671	67
Sample_SCA60.Cluster5985cbin.1	98.31	1.265	56
Sample_SCA60.Cluster2624cbin.1	96.63	1.923	29
Sample_SCA60.Cluster131	93.62	1.342	115
Sample_SCA60.Cluster874	100.00	0.806	20
Sample_SCA60.Cluster2331cbin.1	98.10	0.000	79
Sample_SCA60.mb.22	97.98	1.677	98
Sample_SCA60.Cluster560cbin.1	98.39	2.210	222
Sample_SCA60.Cluster7540cbin.1	80.95	2.534	343
Sample_SCA60.Cluster2720	97.95	0.000	23
Sample_SCA60.Cluster11954cbin.1	97.31	1.006	35
Sample_SCA60.mb.84	94.94	3.531	38
Sample_SCA60.mb.24	91.75	2.531	347
Sample_SCA60.Cluster11269cbin.1	82.35	2.237	433
Sample_SCA60.Cluster9931	95.97	0.000	25
Sample_SCA60.mb.25	95.19	0.480	49
Sample_SCA60.mb.13cbin.1	95.97	0.000	48
Sample_SCA60.mb.52cbin.1	80.98	2.049	347
Sample_SCA60.mb.104cbin.1	94.23	0.961	112
Sample_SCA60.Cluster10224cbin.1	99.51	2.495	63
Sample_SCA60.mb.18	97.31	0.000	20
Sample_SCA60.mb.81cbin.1	84.61	0.854	36
Sample_SCA60.mb.96cbin.1	91.93	1.981	91
Sample_SCA60.mb.86	92.38	0.671	235
Sample_SCA60.Cluster10911	92.98	1.265	97
Sample_SCA60.mb.67cbin.1	93.91	2.364	76
Sample_SCA60.mb.77	95.30	0.671	90
Sample_SCA60.Cluster5689cbin.1	90.24	2.416	78
Sample_SCA60.mb.87	85.90	4.362	102
Sample_SCA60.mb.91	95.04	0.675	71
Sample_SCA60.mb.83cbin.1	81.66	3.020	95
Sample_SCA60.mb.2cbin.1	98.86	0.651	31
Sample_SCA60.mb.9cbin.1	95.97	1.342	54
Sample_SCA60.mb.57cbin.1	97.98	0.000	86
Sample_SCA60.Cluster3577cbin.1	88.74	2.317	558
Sample_SCA60.mb.90cbin.1	93.63	3.045	92
Sample_SCA60.mb.1	88.19	2.255	182
Sample_SCA60.mb.48cbin.1	98.92	0.537	153
Sample_SCA60.mb.92cbin.1	95.23	2.177	115
Sample_SCA60.Cluster205cbin.1	96.31	0.725	274
Sample_SCA61.mb.12	96.77	1.075	94
Sample_SCA61.mb.105	95.80	1.165	86
Sample_SCA61.mb.25	93.40	2.572	254
Sample_SCA61.Cluster3845cbin.1	80.19	0.000	31
Sample_SCA61.mb.2	81.29	0.134	33

Sample_SCA61.mb.27	94.07	1.118	63
Sample_SCA61.Cluster430	99.51	0.016	32
Sample_SCA61.mb.26	98.65	0.000	52
Sample_SCA61.Cluster6157cbin.1	99.32	0.671	17
Sample_SCA61.Cluster26cbin.1	82.60	4.657	298
Sample_SCA61.Cluster7291cbin.1	97.98	0.000	20
Sample_SCA61.mb.47	91.94	3.355	106
Sample_SCA61.Cluster572	96.87	0.000	34
Sample_SCA61.Cluster8403cbin.1	97.53	0.000	82
Sample_SCA61.Cluster6202cbin.1	90.71	1.572	303
Sample_SCA61.Cluster4540	90.89	1.222	231
Sample_SCA61.mb.16	97.46	0.000	175
Sample_SCA61.mb.52cbin.1	91.94	0.000	28
Sample_SCA61.mb.73	89.70	2.013	118
Sample_SCA61.mb.23cbin.1	97.80	0.000	21
Sample_SCA61.mb.48	82.77	1.369	271
Sample_SCA61.Cluster3533cbin.1	94.07	0.555	463
Sample_SCA61.mb.63	97.95	0.000	20
Sample_SCA61.mb.95cbin.1	91.35	2.531	45
Sample_SCA61.mb.66cbin.1	92.14	0.000	24
Sample_SCA61.mb.80cbin.1	100.00	0.480	31
Sample_SCA61.Cluster3102cbin.1	91.15	2.168	63
Sample_SCA61.Cluster3246	97.88	0.576	60
Sample_SCA61.Cluster5366cbin.1	94.99	0.616	382
Sample_SCA61.Cluster3521cbin.1	95.64	0.743	43
Sample_SCA61.mb.96cbin.1	92.17	0.480	141
Sample_SCA64.Cluster2709	81.52	0.671	30
Sample_SCA64.Cluster428cbin.1	94.68	1.515	77
Sample_SCA64.mb.101	88.24	0.000	157
Sample_SCA64.mb.68cbin.1	96.55	3.605	113
Sample_SCA64.Cluster4036	98.99	0.000	40
Sample_SCA64.mb.25	91.27	1.510	65
Sample_SCA64.mb.36cbin.1	98.09	0.994	138
Sample_SCA64.mb.102	95.95	0.000	74
Sample_SCA64.mb.33	89.93	0.335	44
Sample_SCA64.Cluster5527cbin.1	99.32	0.671	23
Sample_SCA64.Cluster1692cbin.1	99.90	0.000	93
Sample_SCA64.mb.51	82.55	0.000	86
Sample_SCA64.mb.13cbin.1	96.98	1.682	161
Sample_SCA64.mb.1cbin.1	86.58	0.909	218
Sample_SCA64.mb.43cbin.1	94.06	1.478	196
Sample_SCA64.mb.10	90.22	1.778	34
Sample_SCA64.Cluster336	97.95	0.000	35
Sample_SCA64.Cluster4286cbin.1	97.98	0.335	63
Sample_SCA64.Cluster981	98.75	0.625	27
Sample_SCA64.Cluster6262cbin.1	98.20	0.000	94
Sample_SCA64.mb.4cbin.1	98.37	2.412	69
Sample_SCA64.Cluster4525	98.87	0.000	24
Sample_SCA64.mb.91	93.58	2.237	117
Sample_SCA64.mb.48	92.62	0.880	226
Sample_SCA64.mb.99	97.76	0.335	80
Sample_SCA64.mb.79cbin.1	92.79	0.921	289
Sample_SCA64.Cluster3798cbin.1	98.79	1.610	144
Sample_SCA64.mb.65cbin.1	98.79	2.898	69
Sample_SCA64.mb.81	99.51	3.525	31
Sample_SCA64.mb.75cbin.1	98.61	0.576	39
Sample_SCA64.mb.60	93.58	0.961	85
Sample_SCA64.Cluster2169cbin.1	93.63	1.319	172

Sample_SCA64.mb.9	85.60	0.510	126
Sample_SCA64.Cluster2443	95.71	0.269	147
Sample_SCA64.mb.93cbin.1	97.16	1.886	97
Sample_SCA64.Cluster3337	98.65	0.512	36
Sample_SCA64.mb.74cbin.1	81.98	0.646	74
Sample_SCA64.mb.77	85.82	0.371	80
Sample_SCA64.mb.44	87.41	2.267	92
Sample_SCA65.Cluster3577cbin.1	97.84	0.000	36
Sample_SCA65.mb.10	97.42	1.470	86
Sample_SCA65.mb.19	93.95	1.342	84
Sample_SCA65.Cluster164cbin.1	93.28	0.671	54
Sample_SCA65.mb.27cbin.1	84.35	2.284	181
Sample_SCA65.Cluster3800	89.13	2.531	35
Sample_SCA65.mb.13	91.94	0.083	75
Sample_SCA65.mb.7	91.38	2.516	324
Sample_SCA65.Cluster1800cbin.1	96.71	0.961	26
Sample_SCA65.Cluster3067cbin.1	90.99	1.696	282
Sample_SCA65.mb.24cbin.1	92.61	0.000	45
Sample_SCA65.mb.79	93.62	2.013	86
Sample_SCA65.Cluster1344	97.95	0.680	22
Sample_SCA65.mb.14cbin.1	89.26	0.000	32
Sample_SCA65.mb.16	94.52	0.000	50
Sample_SCA65.mb.62	93.95	0.000	21
Sample_SCA65.mb.61	98.04	1.442	35
Sample_SCA65.mb.82	94.23	1.602	155
Sample_SCA65.mb.43	93.95	0.000	29
Sample_SCA65.mb.71cbin.1	93.28	1.860	34
Sample_SCA65.mb.56cbin.1	88.57	4.143	233
Sample_SCA65.mb.92	97.31	0.000	11
Sample_SCA65.mb.54cbin.1	97.98	1.342	54
Sample_SCA65.mb.96	95.96	0.268	38
Sample_SCA65.Cluster4304cbin.1	94.20	2.053	98
Sample_SCA65.Cluster4074cbin.1	93.90	1.881	437
Sample_SCA65.Cluster3562cbin.1	83.07	4.460	41
Sample_SCA65.mb.66cbin.1	93.10	0.751	168
Sample_SCA65.mb.37cbin.1	97.62	0.576	54
Sample_SCA65.mb.25	88.17	0.631	63
Sample_SCA66.mb.53	91.99	3.020	108
Sample_SCA66.Cluster55cbin.1	92.61	1.698	133
Sample_SCA66.Cluster1328cbin.1	89.26	0.000	70
Sample_SCA66.mb.82	91.57	2.247	55
Sample_SCA66.Cluster3505cbin.1	99.18	0.077	54
Sample_SCA66.mb.64cbin.1	98.65	0.000	37
Sample_SCA66.mb.24cbin.1	97.15	1.956	172
Sample_SCA66.mb.49cbin.1	94.42	2.995	216
Sample_SCA66.Cluster1226	96.15	0.961	23
Sample_SCA66.Cluster241cbin.1	94.63	2.628	157
Sample_SCA66.Cluster4855cbin.1	95.97	0.671	18
Sample_SCA66.Cluster7665	99.32	0.335	15
Sample_SCA66.Cluster283	97.95	0.000	22
Sample_SCA66.mb.83cbin.1	96.50	1.165	85
Sample_SCA66.Cluster963	97.72	0.568	25
Sample_SCA66.mb.39cbin.1	95.67	1.497	168
Sample_SCA66.mb.22cbin.1	97.26	0.000	61
Sample_SCA66.mb.40	95.89	1.449	83
Sample_SCA66.Cluster1132cbin.1	100.00	0.000	44
Sample_SCA66.mb.7	97.98	0.000	66
Sample_SCA66.Cluster5969	97.73	0.000	15

Sample_SCA66.mb.57	94.78	4.840	130
Sample_SCA66.mb.59cbin.1	99.13	0.338	182
Sample_SCA66.mb.80cbin.1	99.46	2.692	35
Sample_SCA66.mb.35	89.37	4.710	72
Sample_SCA66.mb.71	92.54	3.846	86
Sample_SCA66.Cluster6953cbin.1	97.58	0.000	82
Sample_SCA66.mb.84	99.03	3.381	95
Sample_SCA66.mb.44	95.18	1.410	125
Sample_SCA66.Cluster4395cbin.1	99.61	0.769	87
Sample_SCA66.mb.92cbin.1	90.76	2.145	112
Sample_SCA66.mb.62cbin.1	87.53	3.159	154
Sample_SCA66.mb.88cbin.1	88.39	1.146	250
Sample_SCA67.mb.128cbin.1	96.54	1.101	198
Sample_SCA67.mb.121	96.77	0.806	47
Sample_SCA67.mb.106	90.88	0.671	44
Sample_SCA67.mb.59	88.00	0.000	54
Sample_SCA67.mb.103	95.07	0.223	180
Sample_SCA67.mb.111cbin.1	83.47	1.006	192
Sample_SCA67.Cluster5052	85.55	0.000	56
Sample_SCA67.mb.29cbin.1	84.29	3.313	288
Sample_SCA67.mb.112cbin.1	87.91	1.311	56
Sample_SCA67.Cluster3344cbin.1	98.07	2.403	31
Sample_SCA67.Cluster15592cbin.1	88.65	2.465	407
Sample_SCA67.mb.68cbin.1	93.38	0.762	201
Sample_SCA67.mb.52cbin.1	93.28	4.161	48
Sample_SCA67.mb.124cbin.1	89.37	4.228	284
Sample_SCA67.mb.21	83.42	1.442	340
Sample_SCA67.mb.90	97.76	0.000	57
Sample_SCA67.Cluster9603cbin.1	91.73	4.610	191
Sample_SCA67.mb.11cbin.1	91.54	2.245	109
Sample_SCA67.mb.13	95.46	0.062	133
Sample_SCA67.mb.32	83.01	0.000	336
Sample_SCA67.mb.78	95.63	0.469	116
Sample_SCA67.mb.42cbin.1	97.31	1.677	31
Sample_SCA67.mb.63	92.28	1.230	163
Sample_SCA67.mb.88	91.94	0.939	51
Sample_SCA67.mb.1	98.86	0.000	33
Sample_SCA67.mb.96	90.93	0.000	44
Sample_SCA67.mb.41	99.03	1.449	55
Sample_SCA67.mb.6cbin.1	99.32	0.000	20
Sample_SCA67.mb.91	98.65	0.469	41
Sample_SCA67.mb.97	97.09	0.000	31
Sample_SCA67.mb.92cbin.1	93.26	4.887	275
Sample_SCA67.mb.81	97.31	1.789	60
Sample_SCA68.Cluster11483	85.95	1.123	14
Sample_SCA68.Cluster11308	98.66	0.111	35
Sample_SCA68.Cluster1343cbin.1	93.70	0.932	58
Sample_SCA68.Cluster215cbin.1	91.49	0.000	64
Sample_SCA68.Cluster53	82.88	1.006	43
Sample_SCA68.mb.76	85.95	4.494	61
Sample_SCA68.mb.69	92.93	1.342	24
Sample_SCA68.mb.34cbin.1	81.19	0.592	265
Sample_SCA68.mb.74cbin.1	96.77	0.000	68
Sample_SCA68.mb.17	88.92	1.006	63
Sample_SCA68.mb.31	81.65	2.237	79
Sample_SCA68.mb.39	92.93	0.335	94
Sample_SCA68.mb.49	95.74	1.342	47
Sample_SCA68.Cluster224cbin.1	89.93	0.671	32

Sample_SCA68.mb.27	93.95	0.335	177
Sample_SCA68.Cluster615cbin.1	92.39	2.034	149
Sample_SCA68.mb.62	94.25	2.027	120
Sample_SCA68.Cluster1382cbin.1	97.31	0.503	17
Sample_SCA68.mb.30cbin.1	99.36	0.632	106
Sample_SCA68.mb.45	92.72	0.000	148
Sample_SCA68.Cluster2827cbin.1	97.27	1.342	22
Sample_SCA68.Cluster10198	98.94	0.000	25
Sample_SCA68.mb.79	87.91	4.781	45
Sample_SCA68.mb.64cbin.1	98.55	2.884	80
Sample_SCA68.mb.38	97.98	1.342	56
Sample_SCA68.Cluster8245	99.51	0.000	51
Sample_SCA68.mb.6	98.02	1.201	63
Sample_SCA68.mb.65cbin.1	98.65	2.181	93
Sample_SCA68.mb.81	97.09	0.000	32
Sample_SCA68.Cluster5605cbin.1	98.14	1.610	63
Sample_SCA68.mb.72cbin.1	98.12	0.877	305
Sample_SCA68.mb.51cbin.1	93.27	2.133	262
Sample_SCA69.Cluster5198cbin.1	93.26	4.362	22
Sample_SCA69.Cluster249cbin.1	87.80	1.759	54
Sample_SCA69.Cluster361cbin.1	83.67	4.926	294
Sample_SCA69.mb.20	97.90	0.932	162
Sample_SCA69.Cluster65cbin.1	89.11	0.000	27
Sample_SCA69.Cluster3856	91.94	0.000	19
Sample_SCA69.Cluster1774cbin.1	92.86	1.677	144
Sample_SCA69.Cluster851	82.57	1.682	419
Sample_SCA69.Cluster974cbin.1	91.66	1.612	105
Sample_SCA69.mb.34	86.89	1.265	175
Sample_SCA69.Cluster6cbin.1	85.98	1.808	324
Sample_SCA69.Cluster12637	100.00	0.000	23
Sample_SCA69.Cluster2119	93.76	0.598	34
Sample_SCA69.Cluster12106	83.93	1.570	254
Sample_SCA69.Cluster1131cbin.1	94.37	0.833	33
Sample_SCA69.mb.40cbin.1	92.12	2.348	96
Sample_SCA69.mb.26cbin.1	90.60	1.006	83
Sample_SCA69.Cluster171cbin.1	92.49	1.020	345
Sample_SCA69.Cluster347	86.39	2.947	63
Sample_SCA69.Cluster8586cbin.1	98.65	0.000	23
Sample_SCA69.mb.42cbin.1	93.28	3.020	59
Sample_SCA69.mb.24	94.25	1.677	29
Sample_SCA69.mb.21cbin.1	98.56	3.333	35
Sample_SCA69.mb.22	96.31	4.423	57
Sample_SCA69.Cluster8764	96.69	0.966	166
Sample_SCA69.Cluster6685cbin.1	98.04	1.351	99
Sample_SCA69.mb.54	91.94	1.016	41
Sample_SCA69.mb.31	98.73	1.265	108
Sample_SCA69.mb.78	97.98	0.806	44
Sample_SCA69.mb.100cbin.1	91.55	2.229	279
Sample_SCA69.mb.46	92.31	0.998	75
Sample_SCA69.mb.64cbin.1	90.09	1.351	305
Sample_SCA69.mb.73	89.26	0.223	58
Sample_SCA69.mb.66	80.40	3.061	451
Sample_SCA69.mb.55cbin.1	82.02	0.552	248
Sample_SCA69.Cluster7739cbin.1	97.31	0.000	114
Sample_SCA69.Cluster7332	93.56	0.961	115
Sample_SCA69.Cluster7186cbin.1	98.14	0.210	60
Sample_SCA69.mb.52	99.36	1.665	118
Sample_SCA69.mb.75cbin.1	95.04	4.339	118

Sample_SCA69.mb.86	94.65	1.362	106
Sample_SCA69.mb.87	97.10	0.680	133
Sample_SCA69.Cluster1375cbin.1	93.48	0.997	207
Sample_SCA69.mb.72	87.16	0.377	38
Sample_SCA69.mb.76	93.23	0.187	70
Sample_SCA69.Cluster810	93.00	4.682	68
Sample_SCA70.Cluster4533cbin.1	95.96	0.000	75
Sample_SCA70.Cluster1750cbin.1	88.53	4.423	125
Sample_SCA70.Cluster362cbin.1	90.60	4.194	96
Sample_SCA70.mb.109cbin.1	89.09	0.806	284
Sample_SCA70.Cluster572cbin.1	98.43	0.000	143
Sample_SCA70.Cluster4600cbin.1	97.98	0.671	52
Sample_SCA70.Cluster10417cbin.1	96.45	2.995	53
Sample_SCA70.Cluster4264cbin.1	91.99	0.138	183
Sample_SCA70.mb.14	93.24	1.342	92
Sample_SCA70.mb.132cbin.1	99.32	0.671	20
Sample_SCA70.mb.103cbin.1	88.59	2.839	142
Sample_SCA70.Cluster4846	95.70	0.000	29
Sample_SCA70.Cluster7775	100.00	0.898	44
Sample_SCA70.Cluster8429cbin.1	95.30	0.167	71
Sample_SCA70.Cluster5559cbin.1	97.33	0.000	36
Sample_SCA70.mb.112	92.06	0.634	96
Sample_SCA70.mb.116	87.69	0.000	87
Sample_SCA70.Cluster2459cbin.1	97.79	0.000	63
Sample_SCA70.Cluster11952cbin.1	98.22	0.966	137
Sample_SCA70.Cluster10895cbin.1	95.60	2.646	147
Sample_SCA70.mb.37cbin.1	89.16	4.026	103
Sample_SCA70.Cluster4626cbin.1	96.08	0.880	209
Sample_SCA70.mb.34	85.46	2.745	265
Sample_SCA70.mb.115	80.33	0.952	304
Sample_SCA70.mb.43cbin.1	95.22	0.000	90
Sample_SCA70.mb.87cbin.1	95.80	3.263	254
Sample_SCA70.mb.28	93.03	1.014	87
Sample_SCA70.Cluster8153cbin.1	81.65	1.612	653
Sample_SCA70.mb.47	80.02	0.339	155
Sample_SCA70.mb.18	96.30	0.000	46
Sample_SCA70.mb.36cbin.1	87.19	3.763	454
Sample_SCA70.mb.40cbin.1	95.42	3.401	299
Sample_SCA70.mb.70cbin.1	98.56	2.415	88
Sample_SCA70.mb.82cbin.1	100.00	0.000	34
Sample_SCA70.mb.69cbin.1	88.36	1.351	117
Sample_SCA70.mb.83cbin.1	98.76	0.000	44
Sample_SCA70.mb.90	96.55	1.006	45
Sample_SCA70.mb.84cbin.1	94.35	2.013	100
Sample_SCA70.mb.7cbin.1	95.97	1.677	55
Sample_SCA70.mb.86cbin.1	96.41	0.377	54
Sample_SCA70.Cluster527cbin.1	97.95	1.265	326
Sample_SCA70.mb.65cbin.1	96.35	3.101	246
Sample_SCB55.Cluster15779	100.00	0.111	27
Sample_SCB55.Cluster6784cbin.1	92.74	1.612	49
Sample_SCB55.mb.132cbin.1	98.27	2.664	32
Sample_SCB55.Cluster5577cbin.1	80.05	1.342	200
Sample_SCB55.mb.26	93.10	2.821	300
Sample_SCB55.Cluster142	81.13	1.006	170
Sample_SCB55.Cluster15984	97.60	0.000	21
Sample_SCB55.mb.128	89.74	0.854	7
Sample_SCB55.mb.123	96.64	0.000	20
Sample_SCB55.Cluster15282	97.16	0.042	132

Sample_SCB55.Cluster5831	92.95	0.000	36
Sample_SCB55.mb.2	87.91	0.671	184
Sample_SCB55.mb.111	89.66	0.894	87
Sample_SCB55.mb.62	92.40	0.699	10
Sample_SCB55.mb.20	90.26	2.348	44
Sample_SCB55.Cluster1195	99.43	0.077	20
Sample_SCB55.Cluster13242	98.79	0.294	50
Sample_SCB55.mb.39	93.28	1.342	45
Sample_SCB55.Cluster9473cbin.1	97.09	0.000	41
Sample_SCB55.Cluster13869cbin.1	98.63	0.671	47
Sample_SCB55.mb.57	95.30	0.671	49
Sample_SCB55.mb.49cbin.1	93.52	1.666	48
Sample_SCB55.mb.52cbin.1	85.17	3.075	265
Sample_SCB55.mb.25	97.31	0.738	16
Sample_SCB55.mb.96	93.35	0.699	280
Sample_SCB55.mb.1cbin.1	89.65	3.522	162
Sample_SCB55.mb.107cbin.1	96.06	1.545	167
Sample_SCB55.mb.58	92.50	4.283	193
Sample_SCB55.mb.71	88.72	2.013	272
Sample_SCB55.mb.7	90.64	0.000	24
Sample_SCB55.mb.94cbin.1	95.03	1.677	60
Sample_SCB55.mb.97	97.18	1.342	56
Sample_SCB55.mb.83	95.84	0.943	170
Sample_SCB55.mb.61cbin.1	93.23	3.562	319
Sample_SCB55.mb.17cbin.1	97.60	3.487	48
Sample_SCB58.Cluster2238cbin.1	91.10	1.235	264
Sample_SCB58.Cluster251cbin.1	89.68	0.000	36
Sample_SCB58.Cluster7841	92.33	0.806	8
Sample_SCB58.Cluster17584cbin.1	94.18	4.270	59
Sample_SCB58.Cluster323cbin.1	82.40	0.732	289
Sample_SCB58.mb.102	84.21	1.754	291
Sample_SCB58.Cluster16941cbin.1	91.04	2.519	112
Sample_SCB58.Cluster4731	100.00	0.115	13
Sample_SCB58.Cluster533	89.93	1.342	65
Sample_SCB58.Cluster14393cbin.1	81.62	1.979	332
Sample_SCB58.mb.100	86.96	3.189	245
Sample_SCB58.Cluster14600cbin.1	99.32	1.677	17
Sample_SCB58.mb.21	86.79	2.247	31
Sample_SCB58.Cluster11721cbin.1	97.09	0.000	27
Sample_SCB58.Cluster5432cbin.1	91.93	0.021	65
Sample_SCB58.mb.10	81.87	2.318	40
Sample_SCB58.Cluster1280	97.95	0.000	29
Sample_SCB58.mb.103cbin.1	95.14	4.416	222
Sample_SCB58.mb.135	98.38	0.403	45
Sample_SCB58.mb.126cbin.1	98.60	2.331	117
Sample_SCB58.Cluster3866cbin.1	100.00	0.480	40
Sample_SCB58.mb.43	91.57	2.247	33
Sample_SCB58.mb.15	87.80	0.000	40
Sample_SCB58.Cluster6185	96.60	0.000	18
Sample_SCB58.Cluster11768	80.27	3.559	100
Sample_SCB58.Cluster725	98.04	0.921	21
Sample_SCB58.mb.132cbin.1	94.40	3.382	249
Sample_SCB58.mb.76	87.07	3.531	51
Sample_SCB58.mb.116	91.90	0.907	247
Sample_SCB58.mb.32cbin.1	93.26	2.572	127
Sample_SCB58.mb.59	84.38	1.901	141
Sample_SCB58.mb.9	89.24	0.806	68

Sample_SCB58.mb.34cbin.1	95.80	3.020	150
Sample_SCB58.mb.39cbin.1	96.15	0.961	22
Sample_SCB58.mb.87cbin.1	97.98	2.684	244
Sample_SCB58.mb.31	96.63	0.000	39
Sample_SCB58.mb.35	96.41	0.000	94
Sample_SCB58.Cluster11034cbin.1	91.88	1.127	146
Sample_SCB58.mb.7	91.27	1.342	76
Sample_SCB58.Cluster9840cbin.1	99.46	0.000	109
Sample_SCB58.mb.49	88.12	0.000	82
Sample_SCB58.mb.20	98.31	0.961	30
Sample_SCB58.mb.66	96.42	0.061	113
Sample_SCB58.mb.46	92.93	2.348	180
Sample_SCB58.mb.48	98.65	0.302	61
Sample_SCB58.Cluster6891cbin.1	93.98	1.433	438
Sample_SCB58.Cluster9211	99.23	0.897	42
Sample_SCB58.mb.111	93.97	0.384	56
Sample_SCB58.mb.96cbin.1	89.78	1.881	339
Sample_SCB58.mb.97cbin.1	98.49	0.000	23
Sample_SCB58.mb.60	97.47	1.100	136
Sample_SCB59.Cluster161cbin.1	90.96	2.852	160
Sample_SCB59.Cluster480	98.09	0.671	35
Sample_SCB59.Cluster11926cbin.1	97.31	0.000	20
Sample_SCB59.mb.142	94.29	4.026	136
Sample_SCB59.mb.28	85.79	0.894	258
Sample_SCB59.Cluster12411	99.32	2.684	25
Sample_SCB59.mb.135cbin.1	96.80	0.800	154
Sample_SCB59.mb.124cbin.1	94.71	4.921	144
Sample_SCB59.mb.43cbin.1	93.26	2.013	36
Sample_SCB59.Cluster4224cbin.1	97.27	4.250	30
Sample_SCB59.mb.49	93.75	1.075	83
Sample_SCB59.mb.17cbin.1	99.19	0.115	14
Sample_SCB59.mb.25	93.79	2.348	113
Sample_SCB59.mb.107	97.11	0.000	13
Sample_SCB59.mb.16cbin.1	91.17	2.410	269
Sample_SCB59.mb.81	84.26	3.370	21
Sample_SCB59.mb.127	95.30	3.091	272
Sample_SCB59.mb.109	93.41	0.335	77
Sample_SCB59.mb.118	100.00	0.000	21
Sample_SCB59.mb.4cbin.1	88.60	0.000	78
Sample_SCB59.mb.113cbin.1	97.45	0.000	173
Sample_SCB59.mb.51	89.00	0.223	113
Sample_SCB59.Cluster32cbin.1	96.74	0.515	349
Sample_SCB59.mb.101cbin.1	99.43	0.772	115
Sample_SCB59.mb.18	94.23	0.961	60
Sample_SCB59.mb.133cbin.1	89.24	4.161	308
Sample_SCB59.mb.94cbin.1	100.00	0.806	82
Sample_SCB59.mb.63cbin.1	92.39	0.894	135
Sample_SCB59.mb.62cbin.1	94.74	0.671	117
Sample_SCB59.mb.44	89.93	1.677	135
Sample_SCB59.Cluster9671cbin.1	96.77	0.000	99
Sample_SCB59.mb.40	93.59	1.759	42
Sample_SCB59.Cluster7227	97.30	1.538	52
Sample_SCB59.mb.73cbin.1	93.67	0.021	83
Sample_SCB59.mb.141cbin.1	87.86	1.858	299
Sample_SCB59.mb.97	95.19	0.000	7
Sample_SCB59.mb.68	97.16	0.000	76
Sample_SCB59.mb.9cbin.1	99.03	0.897	255
Sample_SCB60.Cluster14735cbin.1	96.62	2.247	55

Sample_SCB60.Cluster14408	89.74	1.424	40
Sample_SCB60.Cluster2146cbin.1	88.25	1.342	74
Sample_SCB60.mb.15	94.94	2.407	39
Sample_SCB60.mb.102cbin.1	88.03	0.854	30
Sample_SCB60.mb.10	95.09	1.510	88
Sample_SCB60.Cluster242	100.00	0.806	33
Sample_SCB60.Cluster11239	87.91	0.000	16
Sample_SCB60.mb.135	95.30	0.000	79
Sample_SCB60.Cluster3500cbin.1	94.11	0.907	218
Sample_SCB60.Cluster10627cbin.1	89.36	1.096	336
Sample_SCB60.Cluster1947	97.46	0.000	73
Sample_SCB60.Cluster10659	97.31	0.335	34
Sample_SCB60.Cluster350cbin.1	86.98	1.250	275
Sample_SCB60.Cluster568cbin.1	93.95	2.684	157
Sample_SCB60.Cluster2276	97.95	0.000	23
Sample_SCB60.mb.30cbin.1	93.24	1.864	79
Sample_SCB60.mb.17	93.95	0.894	83
Sample_SCB60.mb.100cbin.1	91.56	1.677	237
Sample_SCB60.mb.76	85.95	2.247	33
Sample_SCB60.mb.103cbin.1	94.63	0.000	61
Sample_SCB60.mb.18cbin.1	94.63	0.000	64
Sample_SCB60.mb.36	93.24	2.364	83
Sample_SCB60.mb.129	91.68	0.000	106
Sample_SCB60.mb.111cbin.1	99.51	0.641	124
Sample_SCB60.mb.24	87.24	0.000	11
Sample_SCB60.mb.57	90.91	3.020	76
Sample_SCB60.mb.72	86.57	1.235	217
Sample_SCB60.mb.67	80.53	0.000	50
Sample_SCB60.mb.32	97.59	1.442	34
Sample_SCB60.Cluster8880	95.09	0.000	29
Sample_SCB60.mb.75	93.43	2.341	308
Sample_SCB60.mb.49cbin.1	99.32	1.782	24
Sample_SCB60.Cluster9407cbin.1	96.56	4.729	391
Sample_SCB60.mb.12cbin.1	98.38	0.000	160
Sample_SCB60.mb.82	95.03	0.961	168
Sample_SCB60.mb.74cbin.1	95.30	2.684	73
Sample_SCB60.mb.81	89.65	1.304	61
Sample_SCB60.Cluster1292cbin.1	91.83	2.905	95
Sample_SCB61.Cluster2650	96.77	0.000	56
Sample_SCB61.mb.103	80.70	0.000	34
Sample_SCB61.Cluster8139	99.32	1.342	15
Sample_SCB61.mb.67cbin.1	82.75	0.000	217
Sample_SCB61.mb.27	93.95	1.006	42
Sample_SCB61.mb.34	88.97	2.331	383
Sample_SCB61.mb.40cbin.1	89.59	0.894	332
Sample_SCB61.mb.47	96.75	4.026	143
Sample_SCB61.Cluster1101cbin.1	86.05	0.961	23
Sample_SCB61.mb.114cbin.1	97.04	4.746	44
Sample_SCB61.Cluster6209	97.98	0.000	28
Sample_SCB61.Cluster5839	99.32	0.671	19
Sample_SCB61.Cluster112	97.95	0.000	26
Sample_SCB61.mb.45	93.15	0.000	181
Sample_SCB61.mb.82	91.94	0.000	74
Sample_SCB61.Cluster523	96.87	0.000	34
Sample_SCB61.mb.50cbin.1	88.40	1.449	43
Sample_SCB61.Cluster4479	98.65	0.000	21
Sample_SCB61.mb.79	83.94	1.677	330
Sample_SCB61.mb.109cbin.1	95.88	2.531	130

Sample_SCB61.mb.44cbin.1	88.67	4.761	111
Sample_SCB61.mb.31cbin.1	92.39	3.485	61
Sample_SCB61.mb.76cbin.1	85.70	4.081	167
Sample_SCB61.mb.88	90.86	0.000	30
Sample_SCB61.mb.3	81.33	0.716	526
Sample_SCB61.mb.83	90.08	2.066	79
Sample_SCB61.Cluster4578cbin.1	95.25	2.215	195
Sample_SCB61.mb.91cbin.1	99.51	0.000	25
Sample_SCB61.Cluster3478cbin.1	89.03	1.858	44
Sample_SCB61.mb.94	95.84	0.000	25
Sample_SCB61.mb.99cbin.1	91.06	3.461	119
Sample_SCB61.mb.90	98.65	0.576	30
Sample_SCB61.mb.28cbin.1	99.21	0.365	83
Sample_SCB64.Cluster3052	81.85	1.342	31
Sample_SCB64.mb.29cbin.1	82.45	0.000	119
Sample_SCB64.Cluster4934cbin.1	92.58	0.335	254
Sample_SCB64.Cluster7568	94.63	3.020	71
Sample_SCB64.Cluster4325	98.32	1.342	43
Sample_SCB64.Cluster7301cbin.1	88.97	4.749	200
Sample_SCB64.mb.4	90.38	1.923	259
Sample_SCB64.Cluster5490cbin.1	84.00	1.140	197
Sample_SCB64.Cluster3178cbin.1	100.00	0.000	95
Sample_SCB64.Cluster328cbin.1	90.14	0.480	38
Sample_SCB64.Cluster1273	98.75	0.625	25
Sample_SCB64.mb.31	85.63	1.510	178
Sample_SCB64.mb.124cbin.1	98.65	2.848	112
Sample_SCB64.Cluster4422cbin.1	97.58	3.864	78
Sample_SCB64.mb.16	98.16	2.016	171
Sample_SCB64.Cluster345	97.95	0.000	38
Sample_SCB64.Cluster7227	99.32	0.671	24
Sample_SCB64.mb.54cbin.1	92.55	2.237	121
Sample_SCB64.mb.108cbin.1	86.17	3.225	34
Sample_SCB64.mb.105cbin.1	96.64	0.671	35
Sample_SCB64.mb.34	97.95	1.360	211
Sample_SCB64.Cluster9343cbin.1	95.30	3.355	28
Sample_SCB64.mb.18cbin.1	91.81	4.545	33
Sample_SCB64.mb.67	80.57	1.207	298
Sample_SCB64.mb.55	94.71	0.961	37
Sample_SCB64.Cluster4386cbin.1	96.34	2.985	78
Sample_SCB64.mb.28	97.23	0.591	430
Sample_SCB64.Cluster4283cbin.1	98.46	0.512	37
Sample_SCB64.mb.8cbin.1	94.25	2.203	183
Sample_SCB64.mb.57cbin.1	89.39	3.401	101
Sample_SCB64.mb.92	95.30	0.000	19
Sample_SCB64.mb.89	86.38	0.283	123
Sample_SCB64.mb.69cbin.1	97.99	0.724	115
Sample_SCB64.mb.95cbin.1	85.15	1.054	299
Sample_SCB64.mb.122	85.59	1.119	76
Sample_SCB64.mb.46cbin.1	96.92	0.000	87
Sample_SCB64.mb.58cbin.1	85.73	3.667	157
Sample_SCB64.mb.72	88.10	0.371	42
Sample_SCB64.mb.81cbin.1	97.82	1.545	185
Sample_SCB65.Cluster2978	97.84	0.000	37
Sample_SCB65.mb.19	95.25	0.537	204
Sample_SCB65.mb.43cbin.1	82.29	1.677	320
Sample_SCB65.mb.57	85.04	4.697	366
Sample_SCB65.mb.27cbin.1	98.52	0.518	84
Sample_SCB65.Cluster3240	92.63	0.632	33

Sample_SCB65.Cluster2325cbin.1	87.26	2.794	323
Sample_SCB65.mb.34	83.11	0.988	391
Sample_SCB65.mb.24cbin.1	95.97	0.000	59
Sample_SCB65.Cluster6701	97.31	2.348	25
Sample_SCB65.Cluster7656cbin.1	97.31	0.671	18
Sample_SCB65.Cluster123	97.95	0.680	28
Sample_SCB65.mb.70cbin.1	91.27	3.811	152
Sample_SCB65.mb.54	85.20	1.372	277
Sample_SCB65.Cluster4782cbin.1	98.65	0.692	33
Sample_SCB65.Cluster98cbin.1	98.23	3.846	43
Sample_SCB65.mb.10	93.95	0.000	42
Sample_SCB65.mb.3	91.82	3.265	151
Sample_SCB65.mb.66	100.00	0.268	47
Sample_SCB65.mb.79	95.63	2.852	100
Sample_SCB65.mb.84	88.78	1.682	102
Sample_SCB65.Cluster4891cbin.1	95.00	1.811	66
Sample_SCB65.mb.68cbin.1	86.15	0.000	53
Sample_SCB65.mb.72cbin.1	89.88	3.571	229
Sample_SCB65.mb.9cbin.1	90.41	0.000	57
Sample_SCB65.Cluster4119cbin.1	93.81	0.017	506
Sample_SCB65.mb.77cbin.1	96.83	0.105	108
Sample_SCB65.mb.28cbin.1	95.92	1.503	199
Sample_SCB65.mb.20	97.50	0.705	46
Sample_SCB65.mb.37	91.89	4.288	127
Sample_SCB65.mb.4cbin.1	89.30	1.346	658
Sample_SCB65.mb.74cbin.1	95.01	0.185	42
Sample_SCB65.mb.65cbin.1	95.81	1.499	59
Sample_SCB66.Cluster9757cbin.1	82.02	0.000	37
Sample_SCB66.Cluster3087	92.93	1.342	23
Sample_SCB66.Cluster9832cbin.1	92.69	2.247	29
Sample_SCB66.mb.113	92.13	1.284	9
Sample_SCB66.Cluster314cbin.1	97.31	0.894	66
Sample_SCB66.Cluster326cbin.1	93.28	1.342	67
Sample_SCB66.Cluster6cbin.1	88.46	1.342	351
Sample_SCB66.Cluster2065	92.61	1.230	18
Sample_SCB66.Cluster3667cbin.1	99.18	0.000	33
Sample_SCB66.Cluster3687cbin.1	95.56	1.265	22
Sample_SCB66.Cluster2786cbin.1	92.55	1.671	267
Sample_SCB66.Cluster7029	99.32	2.348	15
Sample_SCB66.Cluster332	97.95	0.000	19
Sample_SCB66.Cluster1226	93.01	0.795	21
Sample_SCB66.Cluster7847cbin.1	95.97	0.671	18
Sample_SCB66.mb.23cbin.1	95.46	1.265	124
Sample_SCB66.mb.36cbin.1	99.59	0.115	33
Sample_SCB66.mb.65	98.38	0.000	86
Sample_SCB66.mb.5cbin.1	94.40	0.932	77
Sample_SCB66.Cluster6263	99.47	0.261	74
Sample_SCB66.Cluster5296cbin.1	93.25	0.120	92
Sample_SCB66.Cluster5056	97.26	0.000	32
Sample_SCB66.Cluster1256cbin.1	93.17	3.766	264
Sample_SCB66.mb.107cbin.1	95.27	3.093	137
Sample_SCB66.mb.84	98.65	0.000	22
Sample_SCB66.Cluster9252cbin.1	94.33	2.500	100
Sample_SCB66.Cluster1136cbin.1	100.00	0.000	45
Sample_SCB66.Cluster3927cbin.1	91.36	0.806	424
Sample_SCB66.mb.59	94.71	1.923	25
Sample_SCB66.mb.51cbin.1	95.35	1.700	268
Sample_SCB66.mb.85	90.81	1.006	128

Sample_SCB66.Cluster5699	99.62	0.000	15
Sample_SCB66.mb.95cbin.1	81.21	1.877	393
Sample_SCB66.mb.40	99.03	1.250	30
Sample_SCB66.mb.80	93.23	3.381	153
Sample_SCB66.mb.89cbin.1	94.63	2.013	115
Sample_SCB66.Cluster4043	99.61	3.230	71
Sample_SCB66.mb.96	95.10	1.036	79
Sample_SCB66.mb.62	94.03	2.056	115
Sample_SCB66.mb.87	97.98	0.000	81
Sample_SCB66.mb.76	94.23	0.909	49
Sample_SCB66.mb.30	96.41	3.396	120
Sample_SCB67.Cluster3713cbin.1	98.23	1.209	106
Sample_SCB67.Cluster1428	95.97	0.000	22
Sample_SCB67.mb.101	97.42	0.027	68
Sample_SCB67.Cluster12262	98.99	0.000	53
Sample_SCB67.Cluster6508	80.98	0.000	50
Sample_SCB67.Cluster13396cbin.1	94.19	0.740	97
Sample_SCB67.Cluster10940cbin.1	97.10	1.992	62
Sample_SCB67.Cluster3518	93.99	0.961	24
Sample_SCB67.Cluster19cbin.1	87.47	0.310	346
Sample_SCB67.mb.121	92.61	1.957	70
Sample_SCB67.Cluster6180cbin.1	95.85	0.185	138
Sample_SCB67.Cluster2274cbin.1	99.03	0.721	87
Sample_SCB67.Cluster15509	98.97	0.183	24
Sample_SCB67.mb.24cbin.1	86.57	0.335	14
Sample_SCB67.Cluster6745cbin.1	98.10	0.021	81
Sample_SCB67.mb.123cbin.1	97.31	0.671	85
Sample_SCB67.mb.59cbin.1	90.66	0.000	46
Sample_SCB67.mb.142	97.42	0.488	57
Sample_SCB67.Cluster17310cbin.1	93.02	0.805	410
Sample_SCB67.mb.26	86.46	1.962	374
Sample_SCB67.mb.61	89.11	2.822	174
Sample_SCB67.mb.10	95.97	0.000	20
Sample_SCB67.Cluster153cbin.1	97.65	2.013	148
Sample_SCB67.mb.12	84.92	0.738	101
Sample_SCB67.mb.138	96.21	1.778	142
Sample_SCB67.mb.34	87.91	1.006	38
Sample_SCB67.mb.45	85.57	0.000	135
Sample_SCB67.mb.58cbin.1	87.24	0.134	38
Sample_SCB67.mb.85	83.28	3.691	91
Sample_SCB67.mb.113	99.24	0.000	16
Sample_SCB67.mb.7cbin.1	80.01	2.705	287
Sample_SCB67.mb.65cbin.1	94.79	4.697	243
Sample_SCB67.Cluster7159	88.56	1.602	302
Sample_SCB67.mb.44	92.33	0.000	48
Sample_SCB67.mb.76cbin.1	98.05	0.000	56
Sample_SCB67.mb.117	80.84	1.474	532
Sample_SCB67.mb.64cbin.1	93.73	3.578	190
Sample_SCB67.mb.80	98.65	0.335	44
Sample_SCB67.mb.95cbin.1	83.89	0.335	16
Sample_SCB67.mb.81	97.09	0.000	31
Sample_SCB67.mb.87	88.35	0.062	69
Sample_SCB68.mb.52	86.66	1.454	34
Sample_SCB68.Cluster1534cbin.1	89.51	0.932	51
Sample_SCB68.mb.42	94.15	2.089	208
Sample_SCB68.mb.29	97.45	0.000	80
Sample_SCB68.mb.8	91.57	1.123	14
Sample_SCB68.mb.62cbin.1	88.70	3.230	157

Sample_SCB68.mb.16cbin.1	95.22	2.348	100
Sample_SCB68.mb.72	97.20	1.165	70
Sample_SCB68.Cluster1151cbin.1	87.75	0.000	50
Sample_SCB68.Cluster863cbin.1	88.59	0.335	54
Sample_SCB68.Cluster1667cbin.1	85.46	2.013	255
Sample_SCB68.mb.60cbin.1	95.58	0.671	67
Sample_SCB68.mb.49	82.29	1.677	311
Sample_SCB68.Cluster2701	88.25	0.000	13
Sample_SCB68.mb.10	96.87	1.682	36
Sample_SCB68.Cluster8682cbin.1	95.97	0.335	41
Sample_SCB68.Cluster2582cbin.1	98.73	0.632	96
Sample_SCB68.Cluster3722cbin.1	92.23	2.684	19
Sample_SCB68.mb.77	82.28	2.348	502
Sample_SCB68.Cluster290	91.82	0.993	50
Sample_SCB68.mb.47	99.32	0.000	25
Sample_SCB68.Cluster8783cbin.1	98.94	0.000	39
Sample_SCB68.mb.51	98.38	0.045	66
Sample_SCB68.mb.68	97.09	0.894	32
Sample_SCB68.Cluster8615cbin.1	98.83	0.584	77
Sample_SCB68.Cluster3863cbin.1	80.46	0.576	367
Sample_SCB68.mb.2	93.98	1.524	201
Sample_SCB68.mb.33	93.68	1.486	80
Sample_SCB68.mb.27cbin.1	97.97	0.710	208
Sample_SCB69.Cluster6357	91.25	1.677	21
Sample_SCB69.Cluster69	89.51	3.629	25
Sample_SCB69.mb.12	97.01	0.000	87
Sample_SCB69.mb.110	83.89	0.447	34
Sample_SCB69.mb.123cbin.1	93.35	2.215	143
Sample_SCB69.mb.46	86.30	0.000	261
Sample_SCB69.mb.127	87.91	0.671	33
Sample_SCB69.mb.32	85.36	0.671	63
Sample_SCB69.mb.108cbin.1	91.94	4.697	194
Sample_SCB69.Cluster13611	100.00	0.000	23
Sample_SCB69.Cluster2393	99.38	0.598	30
Sample_SCB69.mb.10	89.39	0.528	108
Sample_SCB69.mb.28	97.84	2.419	98
Sample_SCB69.Cluster5203	98.89	0.000	67
Sample_SCB69.mb.113	87.36	0.460	30
Sample_SCB69.Cluster12889	99.47	2.094	168
Sample_SCB69.Cluster10744cbin.1	97.36	0.671	263
Sample_SCB69.Cluster9183	98.65	0.000	21
Sample_SCB69.mb.25cbin.1	95.26	0.894	28
Sample_SCB69.mb.111cbin.1	94.18	0.883	93
Sample_SCB69.Cluster1157cbin.1	89.71	1.300	25
Sample_SCB69.Cluster10340cbin.1	98.55	0.000	83
Sample_SCB69.mb.116	91.78	1.077	57
Sample_SCB69.Cluster13349cbin.1	86.53	3.059	539
Sample_SCB69.mb.31	96.25	0.680	104
Sample_SCB69.mb.78	80.42	0.000	33
Sample_SCB69.mb.87cbin.1	98.38	0.806	44
Sample_SCB69.mb.15	93.20	0.377	34
Sample_SCB69.Cluster6631cbin.1	95.16	1.075	144
Sample_SCB69.mb.122cbin.1	91.81	2.534	181
Sample_SCB69.mb.66cbin.1	90.60	2.852	38
Sample_SCB69.mb.72	94.63	0.092	173
Sample_SCB69.mb.80cbin.1	91.89	2.702	405
Sample_SCB69.mb.82cbin.1	89.26	4.209	70
Sample_SCB69.mb.54	94.25	1.013	66

Sample_SCB69.mb.97	85.44	3.914	48
Sample_SCB69.mb.91cbin.1	92.61	1.525	25
Sample_SCB69.mb.47	99.20	2.373	88
Sample_SCB69.mb.101	93.40	2.097	142
Sample_SCB69.mb.89cbin.1	94.56	0.961	316
Sample_SCB69.mb.94	87.98	3.401	389
Sample_SCB69.mb.99cbin.1	98.73	1.265	101
Sample_SCB69.mb.74	93.55	4.567	275
Sample_SCB69.mb.95cbin.1	91.23	0.496	93
Sample_SCB69.mb.30	98.39	0.833	82
Sample_SCB69.Cluster2719cbin.1	95.97	3.396	219
Sample_SCB69.mb.96	94.28	1.972	233
Sample_SCB69.mb.41cbin.1	95.99	1.115	64
Sample_SCB69.mb.39	97.36	0.222	100
Sample_SCB70.Cluster16715	89.32	1.284	15
Sample_SCB70.Cluster16251	93.53	4.494	35
Sample_SCB70.Cluster403cbin.1	98.38	0.000	46
Sample_SCB70.Cluster14535	86.24	0.000	24
Sample_SCB70.mb.28	91.01	0.000	32
Sample_SCB70.mb.12	89.51	0.000	43
Sample_SCB70.Cluster394	86.91	3.355	38
Sample_SCB70.Cluster73cbin.1	86.01	0.223	59
Sample_SCB70.Cluster3672	94.63	0.671	42
Sample_SCB70.Cluster14944	90.85	1.314	174
Sample_SCB70.Cluster16059cbin.1	86.40	1.333	17
Sample_SCB70.mb.114	81.60	1.418	286
Sample_SCB70.Cluster6337cbin.1	100.00	0.898	42
Sample_SCB70.mb.54	91.66	4.032	320
Sample_SCB70.Cluster8cbin.1	82.63	1.972	381
Sample_SCB70.mb.74	99.19	1.075	57
Sample_SCB70.Cluster4044	93.76	0.000	28
Sample_SCB70.mb.16	88.28	1.478	272
Sample_SCB70.mb.10	95.57	0.680	233
Sample_SCB70.mb.37	85.96	3.020	106
Sample_SCB70.Cluster8507cbin.1	97.09	0.000	39
Sample_SCB70.mb.112	98.38	2.560	89
Sample_SCB70.mb.17cbin.1	94.02	0.939	141
Sample_SCB70.mb.14	98.38	3.961	53
Sample_SCB70.mb.58	90.48	4.113	371
Sample_SCB70.mb.27cbin.1	98.65	0.000	38
Sample_SCB70.mb.52cbin.1	83.79	4.697	87
Sample_SCB70.mb.44	97.58	2.688	171
Sample_SCB70.mb.63	90.39	0.634	93
Sample_SCB70.mb.77cbin.1	89.70	2.013	114
Sample_SCB70.mb.34cbin.1	97.18	0.335	37
Sample_SCB70.mb.29	94.66	0.579	58
Sample_SCB70.mb.8	86.74	4.026	49
Sample_SCB70.mb.96	90.60	0.671	30
Sample_SCB70.mb.78cbin.1	97.42	0.000	59
Sample_SCC55.mb.2	89.74	0.854	7
Sample_SCC55.Cluster5346	96.10	0.838	155
Sample_SCC55.Cluster1346cbin.1	95.97	0.671	112
Sample_SCC55.Cluster2360	92.05	0.335	62
Sample_SCC55.mb.25	86.01	2.684	195
Sample_SCC55.Cluster2032cbin.1	94.60	1.666	57
Sample_SCC55.Cluster37	92.47	1.612	81
Sample_SCC55.Cluster1341	87.65	0.185	64
Sample_SCC55.Cluster4708cbin.1	98.99	0.000	41

Sample_SCC55.Cluster1689	92.72	0.632	52
Sample_SCC55.mb.72cbin.1	97.60	0.000	45
Sample_SCC55.mb.33cbin.1	87.06	0.671	271
Sample_SCC55.Cluster3849	85.44	2.534	278
Sample_SCC55.Cluster7696cbin.1	93.27	2.931	216
Sample_SCC55.mb.24cbin.1	92.91	3.571	195
Sample_SCC55.mb.19cbin.1	97.31	1.006	64
Sample_SCC55.Cluster435	99.43	0.077	21
Sample_SCC55.mb.28	98.65	1.342	24
Sample_SCC55.mb.75	98.11	1.415	197
Sample_SCC55.mb.3cbin.1	90.72	1.373	202
Sample_SCC55.mb.66	95.94	0.000	12
Sample_SCC55.mb.29	85.90	0.000	50
Sample_SCC55.Cluster7720cbin.1	95.41	1.690	86
Sample_SCC55.mb.55	83.77	0.994	131
Sample_SCC55.mb.9	95.88	0.294	49
Sample_SCC55.mb.41	93.48	4.577	99
Sample_SCC55.mb.71	97.44	0.000	30
Sample_SCC55.Cluster680cbin.1	96.41	1.354	42
Sample_SCC58.mb.103	88.20	2.247	35
Sample_SCC58.mb.108cbin.1	87.92	1.123	50
Sample_SCC58.mb.31	93.82	2.407	33
Sample_SCC58.Cluster37cbin.1	87.63	3.411	181
Sample_SCC58.Cluster161	85.40	3.691	192
Sample_SCC58.Cluster4392	100.00	0.115	10
Sample_SCC58.mb.17cbin.1	93.96	0.671	153
Sample_SCC58.mb.37cbin.1	98.38	1.075	89
Sample_SCC58.mb.26cbin.1	88.70	1.881	45
Sample_SCC58.Cluster13902cbin.1	91.60	2.670	93
Sample_SCC58.mb.19	96.30	2.572	239
Sample_SCC58.mb.20	97.90	0.932	93
Sample_SCC58.mb.68	81.33	1.333	45
Sample_SCC58.Cluster2946	96.15	0.961	20
Sample_SCC58.Cluster11902cbin.1	99.32	1.006	17
Sample_SCC58.mb.32cbin.1	93.95	3.355	130
Sample_SCC58.mb.83	91.57	3.370	28
Sample_SCC58.mb.109	81.73	0.000	23
Sample_SCC58.mb.66	92.26	1.342	35
Sample_SCC58.mb.63	95.33	3.225	325
Sample_SCC58.Cluster5765cbin.1	96.60	0.671	24
Sample_SCC58.mb.41cbin.1	96.94	4.957	293
Sample_SCC58.mb.74	95.44	0.806	169
Sample_SCC58.Cluster849	97.95	0.000	30
Sample_SCC58.mb.95cbin.1	88.84	0.806	61
Sample_SCC58.mb.80	86.12	0.000	43
Sample_SCC58.mb.106	95.63	0.894	59
Sample_SCC58.mb.79cbin.1	93.17	3.467	137
Sample_SCC58.mb.72	91.56	2.648	208
Sample_SCC58.Cluster10136	99.62	0.000	23
Sample_SCC58.mb.34	97.10	2.769	63
Sample_SCC58.mb.71	97.27	0.306	69
Sample_SCC58.mb.87cbin.1	95.62	3.817	121
Sample_SCC58.mb.78cbin.1	90.03	2.187	287
Sample_SCC58.mb.90	95.96	1.360	238
Sample_SCC58.Cluster4938cbin.1	96.75	0.993	359
Sample_SCC58.Cluster7703cbin.1	98.43	0.096	133
Sample_SCC58.mb.93cbin.1	89.70	0.000	27

Sample_SCC58.mb.101	94.24	0.375	165
Sample_SCC58.Cluster176cbin.1	89.17	0.949	553
Sample_SCC59.mb.27cbin.1	83.23	1.411	288
Sample_SCC59.mb.36	95.56	0.268	86
Sample_SCC59.Cluster7017cbin.1	93.54	0.115	38
Sample_SCC59.mb.12cbin.1	97.65	3.355	205
Sample_SCC59.mb.84	81.03	1.724	208
Sample_SCC59.mb.9	92.41	2.247	21
Sample_SCC59.Cluster16cbin.1	91.51	2.684	262
Sample_SCC59.mb.70cbin.1	97.60	0.800	71
Sample_SCC59.Cluster8562	94.63	0.000	16
Sample_SCC59.mb.93	89.91	1.342	35
Sample_SCC59.mb.34	93.01	3.635	108
Sample_SCC59.Cluster3093cbin.1	97.27	2.684	23
Sample_SCC59.mb.48	96.31	0.000	58
Sample_SCC59.Cluster9725	99.32	0.671	22
Sample_SCC59.mb.24	100.00	0.000	20
Sample_SCC59.mb.47cbin.1	81.09	2.348	377
Sample_SCC59.mb.33	97.59	2.403	38
Sample_SCC59.mb.87	84.89	1.875	287
Sample_SCC59.mb.90	91.94	1.342	22
Sample_SCC59.Cluster6670cbin.1	96.77	0.335	189
Sample_SCC59.mb.10	94.73	1.988	129
Sample_SCC59.Cluster4295cbin.1	92.53	2.664	222
Sample_SCC59.mb.71cbin.1	100.00	0.000	160
Sample_SCC59.Cluster6696cbin.1	97.50	1.282	272
Sample_SCC60.Cluster16100	94.94	2.407	31
Sample_SCC60.Cluster16043cbin.1	89.32	2.247	80
Sample_SCC60.Cluster13861	97.75	0.000	61
Sample_SCC60.mb.14	92.40	1.398	42
Sample_SCC60.mb.32	97.33	0.000	85
Sample_SCC60.mb.12	85.63	1.062	285
Sample_SCC60.Cluster214cbin.1	92.13	0.806	220
Sample_SCC60.mb.31	92.89	2.521	185
Sample_SCC60.mb.56	96.37	2.419	108
Sample_SCC60.mb.10	89.96	2.852	206
Sample_SCC60.Cluster15816	100.00	0.061	15
Sample_SCC60.Cluster12433	98.65	0.000	21
Sample_SCC60.mb.24cbin.1	95.30	0.083	59
Sample_SCC60.mb.65	97.90	1.165	88
Sample_SCC60.mb.43	82.54	3.645	427
Sample_SCC60.Cluster11285cbin.1	97.31	0.335	39
Sample_SCC60.Cluster12028	99.32	0.000	23
Sample_SCC60.Cluster236	97.95	0.000	25
Sample_SCC60.mb.57	87.27	2.034	284
Sample_SCC60.mb.28	92.09	1.003	172
Sample_SCC60.mb.21	92.28	0.020	176
Sample_SCC60.mb.51	93.91	4.093	114
Sample_SCC60.mb.35cbin.1	87.58	1.342	56
Sample_SCC60.mb.2	94.71	0.480	99
Sample_SCC60.mb.76	93.73	0.000	34
Sample_SCC60.mb.60	85.18	1.342	106
Sample_SCC60.mb.48cbin.1	93.28	4.474	152
Sample_SCC60.mb.45cbin.1	97.46	0.000	82
Sample_SCC60.mb.64cbin.1	96.59	0.680	219
Sample_SCC60.mb.84cbin.1	98.43	3.355	110
Sample_SCC60.Cluster9124cbin.1	95.84	0.000	31
Sample_SCC60.mb.67	95.59	0.671	31

Sample_SCC60.mb.87	93.28	0.000	41
Sample_SCC60.mb.99	93.89	0.000	42
Sample_SCC60.Cluster5145cbin.1	97.84	0.000	224
Sample_SCC60.mb.13	91.73	4.746	177
Sample_SCC60.mb.9	94.59	1.351	83
Sample_SCC60.mb.75cbin.1	88.43	2.144	170
Sample_SCC60.mb.86cbin.1	97.59	1.442	31
Sample_SCC60.mb.81	91.05	1.342	102
Sample_SCC60.Cluster6558cbin.1	93.00	4.770	144
Sample_SCC60.mb.93	97.42	1.449	37
Sample_SCC61.mb.33	95.69	0.000	58
Sample_SCC61.Cluster4039	93.93	0.335	52
Sample_SCC61.mb.94	82.45	2.631	139
Sample_SCC61.mb.29cbin.1	95.97	3.020	191
Sample_SCC61.Cluster213cbin.1	85.99	0.160	305
Sample_SCC61.mb.79cbin.1	91.27	0.671	11
Sample_SCC61.mb.12cbin.1	81.73	4.478	268
Sample_SCC61.mb.42	92.30	0.632	210
Sample_SCC61.mb.53cbin.1	97.67	0.949	43
Sample_SCC61.Cluster175	100.00	0.480	29
Sample_SCC61.Cluster3441cbin.1	87.42	1.481	140
Sample_SCC61.mb.13	84.78	0.510	122
Sample_SCC61.Cluster307cbin.1	97.95	0.000	27
Sample_SCC61.Cluster4758cbin.1	99.32	0.671	19
Sample_SCC61.Cluster3659	98.65	0.000	17
Sample_SCC61.Cluster620	96.87	0.000	34
Sample_SCC61.mb.32	95.25	3.164	105
Sample_SCC61.mb.68	87.16	0.000	81
Sample_SCC61.mb.69cbin.1	97.10	1.449	52
Sample_SCC61.mb.23cbin.1	91.47	4.081	122
Sample_SCC61.mb.78cbin.1	97.18	0.000	66
Sample_SCC61.mb.92cbin.1	81.07	2.844	293
Sample_SCC61.Cluster6030cbin.1	98.65	1.342	81
Sample_SCC61.Cluster4061cbin.1	91.54	3.880	185
Sample_SCC61.Cluster3270cbin.1	91.45	0.604	383
Sample_SCC61.mb.85cbin.1	94.71	0.000	202
Sample_SCC61.mb.41cbin.1	89.76	1.177	213
Sample_SCC61.Cluster2650cbin.1	95.64	3.846	154
Sample_SCC61.Cluster3036cbin.1	98.65	0.576	34
Sample_SCC61.Cluster1752	97.69	0.682	138
Sample_SCC64.mb.26	92.93	1.342	31
Sample_SCC64.mb.22	96.32	1.165	72
Sample_SCC64.Cluster3622cbin.1	96.97	0.000	53
Sample_SCC64.mb.52cbin.1	86.55	3.137	256
Sample_SCC64.mb.25	90.38	0.230	312
Sample_SCC64.Cluster662cbin.1	85.24	0.632	335
Sample_SCC64.Cluster61cbin.1	96.70	0.912	102
Sample_SCC64.Cluster5107	97.31	0.671	25
Sample_SCC64.Cluster7354cbin.1	93.08	0.471	117
Sample_SCC64.Cluster276cbin.1	92.61	0.292	66
Sample_SCC64.mb.13cbin.1	92.78	1.442	83
Sample_SCC64.mb.11	97.23	0.000	36
Sample_SCC64.Cluster820	98.75	0.625	26
Sample_SCC64.Cluster6936cbin.1	93.24	0.000	19
Sample_SCC64.mb.63	93.27	0.671	123
Sample_SCC64.mb.18	97.44	0.000	73
Sample_SCC64.mb.79	99.51	1.442	34
Sample_SCC64.mb.67	99.51	4.106	74

Sample_SCC64.Cluster2663	80.29	0.000	40
Sample_SCC64.Cluster7245	100.00	0.632	67
Sample_SCC64.mb.43cbin.1	97.03	1.700	88
Sample_SCC64.mb.54	96.68	4.739	39
Sample_SCC64.mb.45	97.06	0.105	244
Sample_SCC64.mb.24	91.66	0.901	94
Sample_SCC64.mb.38	98.86	1.509	27
Sample_SCC64.mb.47cbin.1	99.27	0.030	126
Sample_SCC64.Cluster2242	97.69	0.000	69
Sample_SCC64.Cluster3650cbin.1	91.03	1.971	150
Sample_SCC64.Cluster3045	96.15	0.512	38
Sample_SCC64.mb.46cbin.1	92.82	2.137	155
Sample_SCC64.mb.29	90.55	1.115	56
Sample_SCC65.Cluster2480cbin.1	97.84	0.000	35
Sample_SCC65.mb.51cbin.1	98.27	4.075	36
Sample_SCC65.Cluster781cbin.1	91.64	2.684	344
Sample_SCC65.mb.22	97.31	1.677	68
Sample_SCC65.mb.32	97.58	0.518	74
Sample_SCC65.Cluster2641	91.98	2.531	35
Sample_SCC65.mb.7cbin.1	85.82	1.486	320
Sample_SCC65.Cluster5070	96.94	2.190	31
Sample_SCC65.mb.69cbin.1	83.32	1.586	353
Sample_SCC65.Cluster520	97.75	0.961	26
Sample_SCC65.mb.2	83.99	0.907	394
Sample_SCC65.Cluster5719	96.30	0.335	25
Sample_SCC65.mb.70cbin.1	91.27	0.000	38
Sample_SCC65.mb.58cbin.1	85.26	4.026	372
Sample_SCC65.Cluster13cbin.1	100.00	1.075	84
Sample_SCC65.mb.33cbin.1	99.32	0.000	27
Sample_SCC65.Cluster6213cbin.1	95.30	0.335	57
Sample_SCC65.mb.45	94.72	1.285	47
Sample_SCC65.mb.77cbin.1	96.04	1.554	132
Sample_SCC65.mb.28	100.00	0.105	139
Sample_SCC65.mb.53cbin.1	81.70	0.483	665
Sample_SCC65.mb.42cbin.1	91.60	0.939	171
Sample_SCC65.Cluster3329cbin.1	95.91	0.000	46
Sample_SCC65.mb.16	98.77	0.705	161
Sample_SCC65.mb.52	93.68	0.631	63
Sample_SCC66.Cluster3790	95.85	0.806	21
Sample_SCC66.Cluster1441cbin.1	98.60	1.515	89
Sample_SCC66.mb.15	92.69	2.247	20
Sample_SCC66.Cluster294cbin.1	93.28	3.355	130
Sample_SCC66.Cluster2799cbin.1	82.56	1.497	259
Sample_SCC66.Cluster8cbin.1	91.42	2.146	260
Sample_SCC66.mb.118	95.97	0.000	49
Sample_SCC66.Cluster6670cbin.1	91.46	1.932	218
Sample_SCC66.mb.6cbin.1	92.56	1.612	223
Sample_SCC66.Cluster438	97.95	0.000	19
Sample_SCC66.Cluster8261cbin.1	99.32	0.335	21
Sample_SCC66.mb.57cbin.1	80.79	1.282	213
Sample_SCC66.Cluster8780	93.26	0.805	120
Sample_SCC66.mb.33	93.26	0.961	28
Sample_SCC66.mb.54	99.18	0.000	31
Sample_SCC66.Cluster5669cbin.1	80.12	4.975	364
Sample_SCC66.Cluster1357	92.54	0.000	39
Sample_SCC66.Cluster1223cbin.1	93.87	0.480	27
Sample_SCC66.mb.32cbin.1	95.52	3.061	286
Sample_SCC66.mb.90cbin.1	90.60	0.000	122

Sample_SCC66.mb.114cbin.1	98.65	1.342	90
Sample_SCC66.mb.87	97.25	1.054	145
Sample_SCC66.mb.109cbin.1	99.31	0.000	60
Sample_SCC66.mb.107cbin.1	95.67	1.265	198
Sample_SCC66.Cluster5209	98.92	0.000	132
Sample_SCC66.mb.100	91.25	2.788	118
Sample_SCC66.mb.97cbin.1	97.31	0.000	20
Sample_SCC66.mb.27	90.43	0.833	43
Sample_SCC66.mb.2cbin.1	97.81	1.368	168
Sample_SCC66.mb.88cbin.1	97.98	1.342	21
Sample_SCC66.Cluster4739cbin.1	99.61	0.384	92
Sample_SCC66.mb.82cbin.1	98.84	2.572	127
Sample_SCC66.mb.86	91.48	2.884	79
Sample_SCC66.mb.7	99.62	0.000	15
Sample_SCC67.Cluster4189cbin.1	93.00	0.699	103
Sample_SCC67.mb.12	93.33	0.000	39
Sample_SCC67.Cluster3149cbin.1	80.15	0.000	145
Sample_SCC67.Cluster11226cbin.1	92.28	0.000	55
Sample_SCC67.mb.14	97.31	1.006	23
Sample_SCC67.mb.114	95.99	2.348	170
Sample_SCC67.mb.126	89.54	0.488	80
Sample_SCC67.mb.69	83.92	3.225	290
Sample_SCC67.mb.28	85.77	2.348	92
Sample_SCC67.Cluster15193cbin.1	99.32	0.209	26
Sample_SCC67.Cluster11439	97.09	0.000	20
Sample_SCC67.mb.55cbin.1	88.57	0.245	236
Sample_SCC67.Cluster15443	98.65	0.335	48
Sample_SCC67.mb.105cbin.1	97.03	0.370	67
Sample_SCC67.Cluster8360cbin.1	98.05	0.000	63
Sample_SCC67.Cluster45cbin.1	93.86	2.500	332
Sample_SCC67.mb.35	88.75	1.342	51
Sample_SCC67.Cluster8834cbin.1	91.56	1.443	250
Sample_SCC67.mb.26cbin.1	87.61	0.160	402
Sample_SCC67.Cluster15804cbin.1	99.72	1.137	107
Sample_SCC67.Cluster11250	85.37	2.189	498
Sample_SCC67.mb.18	92.29	2.040	241
Sample_SCC67.mb.5cbin.1	90.60	3.304	67
Sample_SCC67.mb.62	86.55	4.854	350
Sample_SCC67.mb.101cbin.1	99.32	0.671	24
Sample_SCC67.Cluster12373cbin.1	99.38	1.159	143
Sample_SCC67.mb.2	91.85	0.194	104
Sample_SCC67.mb.45	87.18	0.671	239
Sample_SCC67.mb.75	94.63	0.000	36
Sample_SCC67.mb.52cbin.1	94.70	1.454	176
Sample_SCC67.mb.103	97.31	0.671	217
Sample_SCC67.mb.73cbin.1	95.01	1.845	202
Sample_SCC67.mb.97cbin.1	89.73	1.677	70
Sample_SCC67.mb.131	99.62	0.577	59
Sample_SCC67.mb.90cbin.1	94.24	1.419	228
Sample_SCC67.mb.63	80.55	2.534	366
Sample_SCC67.mb.95cbin.1	98.07	1.923	36
Sample_SCC67.mb.77cbin.1	93.65	3.366	207
Sample_SCC67.mb.74cbin.1	98.63	1.369	79
Sample_SCC68.mb.39	88.76	4.032	251
Sample_SCC68.mb.17	91.60	0.932	71
Sample_SCC68.Cluster3923	93.26	1.677	27
Sample_SCC68.mb.58	91.57	1.123	17
Sample_SCC68.mb.23cbin.1	80.73	0.671	230

Sample_SCC68.mb.22cbin.1	83.65	2.819	512
Sample_SCC68.Cluster829cbin.1	90.93	4.530	64
Sample_SCC68.mb.38	93.56	0.335	125
Sample_SCC68.Cluster193cbin.1	81.73	0.671	56
Sample_SCC68.mb.33	83.49	1.571	292
Sample_SCC68.mb.18	85.90	0.000	115
Sample_SCC68.Cluster4067cbin.1	96.66	0.380	138
Sample_SCC68.mb.41	96.14	0.000	81
Sample_SCC68.mb.61	93.95	0.671	48
Sample_SCC68.mb.72cbin.1	100.00	0.111	60
Sample_SCC68.Cluster6989cbin.1	80.47	0.457	367
Sample_SCC68.mb.45cbin.1	89.59	3.355	179
Sample_SCC68.mb.50	94.59	2.027	78
Sample_SCC68.mb.19cbin.1	98.55	0.619	43
Sample_SCC68.mb.28cbin.1	98.73	0.632	107
Sample_SCC68.mb.35	96.64	1.454	175
Sample_SCC68.Cluster9019cbin.1	84.81	2.516	463
Sample_SCC68.mb.68cbin.1	83.22	3.355	59
Sample_SCC68.mb.1	97.09	0.020	28
Sample_SCC68.mb.44cbin.1	96.60	1.006	27
Sample_SCC68.Cluster9947cbin.1	98.94	0.000	27
Sample_SCC68.mb.59cbin.1	94.63	1.342	147
Sample_SCC68.mb.66	96.86	0.335	59
Sample_SCC68.mb.60	96.15	0.961	52
Sample_SCC68.mb.20	99.51	0.966	96
Sample_SCC68.mb.10	92.93	1.115	81
Sample_SCC68.mb.49	98.49	2.464	184
Sample_SCC68.mb.24cbin.1	96.67	1.144	257
Sample_SCC69.mb.31	97.58	0.806	45
Sample_SCC69.mb.56cbin.1	99.19	1.075	138
Sample_SCC69.mb.37	99.30	0.932	87
Sample_SCC69.Cluster248cbin.1	86.40	0.671	39
Sample_SCC69.Cluster2319cbin.1	88.92	1.447	39
Sample_SCC69.mb.28cbin.1	99.19	1.612	31
Sample_SCC69.mb.15cbin.1	92.73	2.531	431
Sample_SCC69.mb.29	92.42	0.000	42
Sample_SCC69.Cluster3524cbin.1	93.95	0.671	41
Sample_SCC69.mb.34cbin.1	84.36	1.363	405
Sample_SCC69.mb.57	84.44	0.080	45
Sample_SCC69.mb.30cbin.1	86.57	3.859	40
Sample_SCC69.mb.32	84.16	1.363	320
Sample_SCC69.mb.22cbin.1	98.38	2.419	90
Sample_SCC69.Cluster3324cbin.1	99.30	1.265	103
Sample_SCC69.Cluster10971	100.00	0.000	25
Sample_SCC69.Cluster10644	86.71	2.770	283
Sample_SCC69.mb.67	89.91	1.677	21
Sample_SCC69.Cluster525cbin.1	90.45	0.681	24
Sample_SCC69.Cluster1876	99.38	0.598	32
Sample_SCC69.mb.25cbin.1	94.72	0.709	278
Sample_SCC69.mb.62	87.69	0.000	51
Sample_SCC69.mb.46cbin.1	87.79	0.184	261
Sample_SCC69.Cluster9042cbin.1	97.82	0.000	88
Sample_SCC69.mb.35	97.84	0.230	108
Sample_SCC69.mb.68cbin.1	88.92	2.013	27
Sample_SCC69.mb.55	94.54	0.460	25
Sample_SCC69.Cluster6823cbin.1	97.63	1.388	121
Sample_SCC69.mb.21	99.62	0.377	33
Sample_SCC69.Cluster5299cbin.1	89.24	3.763	520

Sample_SCC69.mb.58cbin.1	80.89	2.777	98
Sample_SCC69.mb.70cbin.1	90.62	4.697	21
Sample_SCC69.mb.52cbin.1	97.75	4.996	272
Sample_SCC69.mb.60	98.73	0.632	70
Sample_SCC69.mb.9	93.26	1.923	97
Sample_SCC69.mb.75	98.65	0.671	27
Sample_SCC69.mb.83	87.55	3.196	306
Sample_SCC69.mb.69	97.25	1.559	182
Sample_SCC69.mb.43cbin.1	90.56	1.995	162
Sample_SCC69.mb.72cbin.1	93.30	0.000	47
Sample_SCC69.mb.81cbin.1	93.42	0.601	112
Sample_SCC69.Cluster668cbin.1	96.34	2.976	127
Sample_SCC69.mb.7cbin.1	97.70	4.717	299
Sample_SCC70.mb.18	93.82	1.284	20
Sample_SCC70.Cluster7894	91.93	0.699	3
Sample_SCC70.mb.33	88.48	1.404	38
Sample_SCC70.Cluster12265cbin.1	98.65	0.000	25
Sample_SCC70.mb.26	93.54	0.000	55
Sample_SCC70.Cluster1298cbin.1	92.28	0.000	88
Sample_SCC70.mb.49	96.62	1.123	11
Sample_SCC70.Cluster4194cbin.1	80.54	1.486	454
Sample_SCC70.mb.60	87.64	0.000	31
Sample_SCC70.mb.15cbin.1	86.01	0.671	83
Sample_SCC70.mb.110cbin.1	89.81	1.342	74
Sample_SCC70.Cluster6984	90.86	0.966	62
Sample_SCC70.Cluster6586	100.00	0.898	40
Sample_SCC70.mb.28	93.17	0.634	97
Sample_SCC70.Cluster12184	96.24	0.335	34
Sample_SCC70.mb.69cbin.1	87.45	1.075	240
Sample_SCC70.mb.48	82.48	1.006	177
Sample_SCC70.Cluster8175cbin.1	98.24	0.185	111
Sample_SCC70.mb.16	95.30	0.335	59
Sample_SCC70.mb.58	95.10	4.778	230
Sample_SCC70.Cluster4154	97.04	0.000	32
Sample_SCC70.Cluster9642cbin.1	99.32	0.000	43
Sample_SCC70.mb.105	100.00	0.000	25
Sample_SCC70.Cluster12153cbin.1	97.09	0.000	42
Sample_SCC70.mb.34	81.42	0.791	60
Sample_SCC70.mb.20cbin.1	95.97	2.834	69
Sample_SCC70.Cluster6180	84.98	2.082	199
Sample_SCC70.Cluster11577cbin.1	98.87	0.724	159
Sample_SCC70.mb.78cbin.1	98.65	0.671	17
Sample_SCC70.mb.91cbin.1	85.52	1.075	211
Sample_SCC70.mb.102	94.63	0.671	169
Sample_SCC70.Cluster6980cbin.1	96.99	3.437	179
Sample_SCC70.mb.50	90.90	1.360	337
Sample_SCC70.mb.71	84.64	1.067	80
Sample_SCC70.mb.109cbin.1	97.54	0.000	53
Sample_SCC70.mb.52cbin.1	80.59	1.183	385
Sample_SCC70.mb.77	93.07	4.530	190
Sample_SCC70.mb.85	98.12	0.000	106
Sample_SCC70.mb.40	84.13	0.566	343
Sample_SCC70.mb.97	88.68	4.718	81
Sample_SCC70.mb.76cbin.1	98.55	0.000	65
Sample_SCC70.mb.81	97.06	3.043	105
Sample_SCC70.mb.93cbin.1	97.31	0.671	71
Sample_SCC70.mb.88	95.97	0.000	23
Sample_SCC70.mb.92	94.66	1.858	120

Sample_SPA03.Cluster14037cbin.1	92.69	3.370	16
Sample_SPA03.Cluster11746cbin.1	99.32	0.000	21
Sample_SPA03.mb.22	89.88	0.000	32
Sample_SPA03.Cluster260cbin.1	91.27	1.342	59
Sample_SPA03.Cluster4871cbin.1	91.23	2.215	43
Sample_SPA03.mb.116	96.97	0.671	129
Sample_SPA03.mb.39	97.84	0.097	50
Sample_SPA03.Cluster8013cbin.1	99.10	2.684	138
Sample_SPA03.Cluster1587	87.98	0.480	39
Sample_SPA03.mb.23cbin.1	99.30	3.962	90
Sample_SPA03.Cluster3083cbin.1	95.82	1.445	139
Sample_SPA03.mb.18	97.15	1.845	154
Sample_SPA03.Cluster3223cbin.1	86.87	1.301	41
Sample_SPA03.Cluster35	97.98	1.552	103
Sample_SPA03.mb.35	95.63	0.000	174
Sample_SPA03.Cluster8475cbin.1	99.32	0.000	22
Sample_SPA03.mb.118	90.05	1.006	129
Sample_SPA03.mb.119cbin.1	100.00	2.403	46
Sample_SPA03.mb.24	92.30	1.190	170
Sample_SPA03.mb.2	94.40	1.006	133
Sample_SPA03.mb.38	92.05	4.347	262
Sample_SPA03.Cluster453cbin.1	92.52	2.691	362
Sample_SPA03.mb.66cbin.1	97.98	0.335	120
Sample_SPA03.mb.11	98.02	0.480	23
Sample_SPA03.mb.50cbin.1	91.47	2.348	187
Sample_SPA03.mb.19	98.65	2.013	25
Sample_SPA03.Cluster6910cbin.1	99.62	0.000	12
Sample_SPA03.mb.62cbin.1	88.79	3.612	74
Sample_SPA03.mb.27	89.62	1.363	79
Sample_SPA03.Cluster6052cbin.1	95.69	2.916	260
Sample_SPA03.mb.93	94.94	1.845	144
Sample_SPA03.mb.51cbin.1	93.02	4.761	256
Sample_SPA03.mb.54	93.62	4.421	90
Sample_SPA03.mb.69	90.20	3.947	125
Sample_SPA03.mb.92cbin.1	98.99	0.000	45
Sample_SPA03.mb.7	97.31	0.000	10
Sample_SPA03.Cluster6964cbin.1	94.53	3.392	100
Sample_SPA03.Cluster5354	97.17	2.884	76
Sample_SPA03.mb.64	83.30	0.955	470
Sample_SPA03.mb.77	93.75	0.000	34
Sample_SPA03.Cluster5960	98.39	0.000	60
Sample_SPA03.mb.98	97.94	4.026	89
Sample_SPA03.mb.90	96.05	0.000	55
Sample_SPA03.mb.40	91.75	0.375	147
Sample_SPA03.mb.47cbin.1	99.10	1.953	120
Sample_SPA04.Cluster15261cbin.1	82.05	1.754	157
Sample_SPA04.mb.17	89.04	2.247	25
Sample_SPA04.Cluster368cbin.1	96.64	1.531	70
Sample_SPA04.Cluster7492cbin.1	98.65	1.677	125
Sample_SPA04.mb.14cbin.1	90.58	1.342	29
Sample_SPA04.Cluster1305cbin.1	93.89	0.961	93
Sample_SPA04.Cluster6010cbin.1	92.99	1.932	106
Sample_SPA04.Cluster1798cbin.1	90.00	0.625	87
Sample_SPA04.Cluster5326	96.40	1.097	33
Sample_SPA04.mb.42	90.66	1.333	26
Sample_SPA04.Cluster12575	97.98	0.671	20
Sample_SPA04.Cluster88	98.32	1.342	49
Sample_SPA04.Cluster13556	98.53	0.671	42

Sample_SPA04.Cluster13447cbin.1	99.36	0.656	46
Sample_SPA04.mb.102	94.47	1.682	87
Sample_SPA04.Cluster14222	80.09	1.305	410
Sample_SPA04.mb.119cbin.1	88.82	4.697	200
Sample_SPA04.mb.46	88.12	0.949	310
Sample_SPA04.mb.115cbin.1	97.16	0.000	77
Sample_SPA04.mb.79cbin.1	97.17	0.806	39
Sample_SPA04.mb.31	98.38	0.000	74
Sample_SPA04.mb.89	89.51	0.894	226
Sample_SPA04.mb.71cbin.1	92.61	2.125	66
Sample_SPA04.mb.61	87.42	0.000	132
Sample_SPA04.mb.29	88.69	2.224	180
Sample_SPA04.mb.5	93.60	3.061	132
Sample_SPA04.mb.2	92.80	3.605	34
Sample_SPA04.mb.85	100.00	0.115	16
Sample_SPA04.mb.73cbin.1	95.83	2.684	110
Sample_SPA04.mb.26	89.47	0.610	58
Sample_SPA04.mb.88cbin.1	94.19	0.632	139
Sample_SPA04.mb.57	96.93	0.000	18
Sample_SPA04.mb.53cbin.1	98.38	2.403	48
Sample_SPA04.mb.94	84.49	4.641	115
Sample_SPA04.mb.43cbin.1	92.27	4.504	359
Sample_SPA04.mb.75	87.90	1.708	130
Sample_SPA04.mb.64cbin.1	97.43	1.666	165
Sample_SPA06.Cluster13396	88.00	0.000	20
Sample_SPA06.Cluster2511cbin.1	91.25	1.748	51
Sample_SPA06.Cluster6148	100.00	0.000	24
Sample_SPA06.Cluster317cbin.1	95.13	2.013	79
Sample_SPA06.Cluster781	97.20	0.932	74
Sample_SPA06.Cluster4378cbin.1	97.31	0.000	39
Sample_SPA06.Cluster178cbin.1	96.30	0.671	43
Sample_SPA06.Cluster476cbin.1	92.61	3.020	64
Sample_SPA06.Cluster442	98.38	0.806	28
Sample_SPA06.Cluster1690cbin.1	95.63	0.000	12
Sample_SPA06.Cluster528cbin.1	89.93	2.348	82
Sample_SPA06.mb.117	93.93	1.342	20
Sample_SPA06.Cluster2162	97.27	0.000	24
Sample_SPA06.Cluster346cbin.1	97.83	0.000	87
Sample_SPA06.Cluster465cbin.1	100.00	0.268	34
Sample_SPA06.mb.20	90.66	1.333	41
Sample_SPA06.Cluster341cbin.1	96.59	0.226	62
Sample_SPA06.mb.26	92.69	3.370	17
Sample_SPA06.Cluster12015	93.95	0.000	25
Sample_SPA06.mb.119	84.94	2.013	169
Sample_SPA06.mb.114cbin.1	94.83	1.265	48
Sample_SPA06.Cluster5570cbin.1	85.70	0.257	247
Sample_SPA06.Cluster625	97.95	0.000	23
Sample_SPA06.mb.133	94.48	1.698	154
Sample_SPA06.Cluster6549cbin.1	98.34	0.000	47
Sample_SPA06.Cluster7016	94.52	0.000	26
Sample_SPA06.mb.134cbin.1	89.26	0.939	32
Sample_SPA06.Cluster12358	99.36	1.898	114
Sample_SPA06.mb.15	92.89	1.525	262
Sample_SPA06.mb.70	92.23	1.531	265
Sample_SPA06.mb.88cbin.1	83.62	1.724	285
Sample_SPA06.mb.136cbin.1	99.53	0.308	89
Sample_SPA06.mb.3cbin.1	90.60	1.006	34
Sample_SPA06.mb.92	98.65	1.342	37

Sample_SPA06.mb.27	92.11	0.000	63
Sample_SPA06.mb.5	97.02	1.223	31
Sample_SPA06.mb.35	91.48	1.006	229
Sample_SPA06.Cluster5434	99.19	0.192	34
Sample_SPA06.Cluster4291cbin.1	97.88	0.576	51
Sample_SPA06.Cluster4973cbin.1	97.75	0.584	144
Sample_SPA06.mb.93cbin.1	100.00	0.115	15
Sample_SPA06.mb.95cbin.1	98.43	2.013	74
Sample_SPA06.mb.77cbin.1	98.65	0.000	19
Sample_SPA06.mb.7cbin.1	96.59	1.530	236
Sample_SPA06.mb.72cbin.1	93.96	2.464	67
Sample_SPA06.Cluster4990cbin.1	81.33	1.412	639
Sample_SPA06.mb.99cbin.1	99.53	0.307	24
Sample_SPA06.mb.59cbin.1	98.87	2.320	178
Sample_SPA07.Cluster12085	93.82	2.407	16
Sample_SPA07.Cluster12056	87.92	2.247	20
Sample_SPA07.Cluster7043	96.42	0.000	47
Sample_SPA07.Cluster958cbin.1	93.00	1.165	77
Sample_SPA07.Cluster3082cbin.1	97.19	0.671	92
Sample_SPA07.Cluster544cbin.1	89.93	1.006	57
Sample_SPA07.mb.69cbin.1	83.96	1.612	202
Sample_SPA07.Cluster99cbin.1	93.02	0.000	26
Sample_SPA07.Cluster356cbin.1	93.28	2.684	106
Sample_SPA07.mb.4	99.32	2.013	70
Sample_SPA07.mb.50	89.32	0.671	62
Sample_SPA07.mb.46cbin.1	95.20	1.230	205
Sample_SPA07.Cluster10244	90.62	0.951	82
Sample_SPA07.mb.9	85.95	1.123	31
Sample_SPA07.Cluster6053cbin.1	89.26	0.335	20
Sample_SPA07.mb.54	90.49	1.600	131
Sample_SPA07.mb.30cbin.1	97.31	0.000	62
Sample_SPA07.mb.32cbin.1	85.97	0.268	376
Sample_SPA07.mb.7cbin.1	97.98	1.006	57
Sample_SPA07.mb.37	99.03	0.961	38
Sample_SPA07.mb.43cbin.1	90.82	2.173	59
Sample_SPA07.Cluster10547cbin.1	96.64	0.020	104
Sample_SPA07.mb.81cbin.1	90.84	4.261	206
Sample_SPA07.mb.31	94.01	1.842	229
Sample_SPA07.mb.62	99.05	0.000	130
Sample_SPA07.mb.67	100.00	0.480	35
Sample_SPA07.Cluster8392cbin.1	95.00	2.456	116
Sample_SPA07.mb.99cbin.1	87.63	0.960	230
Sample_SPA07.mb.20cbin.1	97.48	0.559	75
Sample_SPA07.mb.57	89.66	0.000	41
Sample_SPA07.mb.82	89.11	0.966	51
Sample_SPA07.mb.92	97.98	0.000	20
Sample_SPA07.Cluster5915cbin.1	99.61	0.984	79
Sample_SPA07.mb.38cbin.1	92.20	1.595	227
Sample_SPA07.mb.98cbin.1	99.51	0.120	93
Sample_SPA08.Cluster4973	92.93	1.342	24
Sample_SPA08.mb.10	91.57	1.685	14
Sample_SPA08.mb.105	88.20	2.247	27
Sample_SPA08.mb.123cbin.1	80.70	0.000	101
Sample_SPA08.mb.108	87.65	0.671	48
Sample_SPA08.Cluster744cbin.1	94.48	2.651	198
Sample_SPA08.Cluster60cbin.1	96.97	0.894	110
Sample_SPA08.Cluster216cbin.1	95.44	2.034	194
Sample_SPA08.Cluster8154	99.19	0.806	8

Sample_SPA08.mb.101cbin.1	89.61	2.539	85
Sample_SPA08.mb.28cbin.1	97.75	0.000	92
Sample_SPA08.mb.42	88.20	1.685	54
Sample_SPA08.Cluster707cbin.1	88.51	3.253	235
Sample_SPA08.Cluster10169	98.65	0.671	18
Sample_SPA08.Cluster6153	100.00	0.480	10
Sample_SPA08.Cluster5908	99.98	1.497	20
Sample_SPA08.Cluster6101cbin.1	97.58	2.898	100
Sample_SPA08.mb.37	80.93	0.671	140
Sample_SPA08.Cluster1878	98.75	0.625	39
Sample_SPA08.mb.119	99.19	0.806	90
Sample_SPA08.mb.52	98.38	0.806	44
Sample_SPA08.Cluster7637	97.09	0.000	23
Sample_SPA08.mb.115	89.76	0.510	160
Sample_SPA08.Cluster12217cbin.1	98.65	0.122	70
Sample_SPA08.Cluster5941cbin.1	98.30	0.675	107
Sample_SPA08.mb.72	100.00	0.111	25
Sample_SPA08.mb.29	89.93	1.342	99
Sample_SPA08.mb.13	81.29	1.530	30
Sample_SPA08.mb.124	86.61	4.407	245
Sample_SPA08.mb.40	91.93	0.061	93
Sample_SPA08.mb.47cbin.1	87.05	2.590	335
Sample_SPA08.mb.120	90.18	0.584	72
Sample_SPA08.mb.65cbin.1	95.97	0.000	39
Sample_SPA08.mb.17	97.31	0.000	15
Sample_SPA08.mb.56	85.55	2.516	209
Sample_SPA08.Cluster6551cbin.1	97.31	3.225	300
Sample_SPA08.mb.75cbin.1	95.74	0.671	127
Sample_SPA08.mb.83cbin.1	97.09	2.818	73
Sample_SPA08.mb.70cbin.1	87.24	0.671	180
Sample_SPA08.mb.68	90.60	1.565	127
Sample_SPA08.mb.74	96.64	0.000	18
Sample_SPA08.mb.14cbin.1	94.73	1.570	67
Sample_SPA08.mb.66	95.57	0.000	43
Sample_SPA08.mb.64cbin.1	90.16	3.463	158
Sample_SPA08.mb.86	100.00	0.000	64
Sample_SPA08.mb.4cbin.1	92.10	3.683	85
Sample_SPA08.mb.90cbin.1	100.00	1.145	31
Sample_SPA08.mb.125	98.51	1.486	41
Sample_SPA08.mb.97cbin.1	97.83	4.342	31
Sample_SPA08.mb.80cbin.1	95.06	2.150	333
Sample_SPA09.mb.27cbin.1	80.70	3.508	57
Sample_SPA09.mb.2cbin.1	81.03	0.000	87
Sample_SPA09.Cluster5439cbin.1	90.73	0.671	334
Sample_SPA09.mb.40cbin.1	88.94	3.020	270
Sample_SPA09.mb.10cbin.1	88.59	0.000	23
Sample_SPA09.mb.90	84.59	2.013	290
Sample_SPA09.Cluster6961	85.90	0.000	42
Sample_SPA09.Cluster404cbin.1	96.05	0.680	229
Sample_SPA09.mb.93cbin.1	87.15	2.013	387
Sample_SPA09.mb.63cbin.1	90.71	1.898	203
Sample_SPA09.mb.91	98.18	1.286	102
Sample_SPA09.Cluster1823	94.54	0.681	15
Sample_SPA09.mb.58	90.70	3.164	130
Sample_SPA09.mb.48	91.03	1.754	99
Sample_SPA09.mb.38	96.62	0.153	76
Sample_SPA09.mb.80cbin.1	92.30	2.777	79
Sample_SPA09.mb.67	95.00	1.449	139

Sample_SPA09.Cluster10012cbin.1	96.88	3.692	72
Sample_SPA09.mb.15cbin.1	95.16	2.914	69
Sample_SPA09.mb.39	93.75	2.430	25
Sample_SPA09.Cluster6494cbin.1	97.73	0.000	26
Sample_SPA09.mb.89	94.81	4.697	51
Sample_SPA09.mb.99cbin.1	94.23	0.961	28
Sample_SPA09.mb.20	87.46	0.000	39
Sample_SPA09.mb.28	93.71	2.971	66
Sample_SPA09.mb.88cbin.1	97.46	0.105	55
Sample_SPA09.mb.82cbin.1	96.79	2.884	288
Sample_SPA09.Cluster99cbin.1	95.49	1.580	171
Sample_SPA10.mb.16cbin.1	98.94	1.265	35
Sample_SPA10.mb.26	98.65	0.671	58
Sample_SPA10.mb.36cbin.1	85.22	0.680	347
Sample_SPA10.mb.42	97.46	0.000	68
Sample_SPA10.mb.19	98.65	0.335	44
Sample_SPA10.mb.53cbin.1	98.32	4.718	497
Sample_SPA10.mb.20cbin.1	90.78	0.170	82
Sample_SPA10.mb.29cbin.1	93.25	1.207	30
Sample_SPA10.mb.61	97.98	0.335	39
Sample_SPA10.Cluster4817	97.98	0.000	18
Sample_SPA10.mb.48	92.49	1.988	147
Sample_SPA10.mb.34	90.78	0.000	201
Sample_SPA10.mb.31cbin.1	96.28	0.632	35
Sample_SPA10.mb.55	86.93	1.169	59
Sample_SPA10.Cluster1134cbin.1	96.83	0.105	55
Sample_SPA10.mb.51cbin.1	97.66	0.000	49
Sample_SPA10.Cluster25cbin.1	96.90	1.209	302
Sample_SPA10.mb.15cbin.1	97.06	2.602	47
Sample_SPA10.mb.18cbin.1	93.36	0.681	54
Sample_SPA10.mb.17cbin.1	99.42	0.192	37
Sample_SPA10.Cluster1582cbin.1	92.37	2.081	253
Sample_SPA10.Cluster2898	97.74	2.819	42
Sample_SPA11.Cluster4973cbin.1	94.05	2.684	358
Sample_SPA11.Cluster127cbin.1	83.48	2.460	175
Sample_SPA11.Cluster6814cbin.1	96.62	0.000	31
Sample_SPA11.Cluster5732	99.19	0.115	15
Sample_SPA11.Cluster231cbin.1	91.72	3.691	64
Sample_SPA11.Cluster440cbin.1	94.62	0.000	43
Sample_SPA11.Cluster6967cbin.1	93.24	0.632	36
Sample_SPA11.Cluster79cbin.1	90.66	2.684	68
Sample_SPA11.Cluster12075cbin.1	97.30	0.134	208
Sample_SPA11.Cluster73cbin.1	98.32	3.087	40
Sample_SPA11.Cluster5881cbin.1	94.68	1.449	73
Sample_SPA11.Cluster3236	97.27	0.000	17
Sample_SPA11.Cluster5039	99.40	0.898	45
Sample_SPA11.Cluster1285	98.75	0.625	26
Sample_SPA11.mb.118	82.55	0.000	139
Sample_SPA11.Cluster2524cbin.1	98.65	1.006	42
Sample_SPA11.Cluster10740	87.68	0.636	71
Sample_SPA11.mb.55cbin.1	89.31	3.761	145
Sample_SPA11.mb.39cbin.1	96.77	1.545	143
Sample_SPA11.Cluster9675	97.98	1.006	17
Sample_SPA11.mb.12cbin.1	84.62	1.898	349
Sample_SPA11.mb.16cbin.1	82.31	1.700	77
Sample_SPA11.mb.14	87.52	2.267	156
Sample_SPA11.Cluster8660	88.04	0.402	72
Sample_SPA11.mb.101cbin.1	95.95	0.671	98

Sample_SPA11.mb.78cbin.1	98.60	3.263	102
Sample_SPA11.mb.18cbin.1	94.48	0.000	102
Sample_SPA11.Cluster4412cbin.1	97.84	0.779	205
Sample_SPA11.Cluster4833cbin.1	96.59	2.338	109
Sample_SPA11.mb.21	99.32	0.335	25
Sample_SPA11.mb.111cbin.1	96.49	0.953	63
Sample_SPA11.Cluster1799cbin.1	89.74	0.192	490
Sample_SPA11.mb.41	95.19	0.961	29
Sample_SPA11.Cluster5091	99.23	0.000	46
Sample_SPA11.mb.43	90.88	2.040	250
Sample_SPA11.mb.3	85.69	0.949	314
Sample_SPA11.mb.37	91.94	0.000	20
Sample_SPA11.mb.38cbin.1	97.26	0.000	34
Sample_SPA11.mb.93	94.14	2.215	198
Sample_SPA11.mb.85	91.10	1.433	103
Sample_SPA11.mb.58cbin.1	81.21	4.988	343
Sample_SPA11.mb.62	90.25	4.305	113
Sample_SPA11.mb.36	99.62	0.000	17
Sample_SPA11.mb.9cbin.1	100.00	0.480	26
Sample_SPA11.mb.51cbin.1	90.70	4.480	377
Sample_SPA11.mb.74cbin.1	90.74	1.119	69
Sample_SPA11.mb.50	88.82	2.416	201
Sample_SPA11.mb.92	94.10	0.827	193
Sample_SPA12.Cluster24708cbin.1	92.41	2.247	29
Sample_SPA12.Cluster11878	92.90	2.633	311
Sample_SPA12.Cluster259	80.69	2.237	111
Sample_SPA12.mb.127	100.00	1.444	58
Sample_SPA12.Cluster10362	96.30	0.000	38
Sample_SPA12.mb.124cbin.1	88.84	3.579	235
Sample_SPA12.Cluster18234cbin.1	96.64	0.000	49
Sample_SPA12.Cluster17354cbin.1	89.39	2.531	397
Sample_SPA12.mb.101	83.66	1.342	277
Sample_SPA12.Cluster23502cbin.1	99.05	0.471	128
Sample_SPA12.mb.117	94.83	2.013	124
Sample_SPA12.Cluster5511cbin.1	81.04	0.679	161
Sample_SPA12.Cluster20418cbin.1	93.95	1.342	31
Sample_SPA12.mb.27	98.38	0.806	48
Sample_SPA12.mb.21cbin.1	93.59	0.115	214
Sample_SPA12.mb.147	99.36	3.375	136
Sample_SPA12.Cluster5034	97.95	0.000	17
Sample_SPA12.mb.109cbin.1	92.61	0.671	150
Sample_SPA12.mb.22cbin.1	86.95	4.593	153
Sample_SPA12.mb.68cbin.1	92.17	0.671	35
Sample_SPA12.mb.73	80.02	1.677	32
Sample_SPA12.mb.52cbin.1	92.98	2.320	208
Sample_SPA12.mb.31	85.02	2.607	256
Sample_SPA12.mb.14cbin.1	80.13	2.348	381
Sample_SPA12.mb.89	92.37	4.918	440
Sample_SPA12.mb.34cbin.1	99.27	0.632	87
Sample_SPA12.mb.95	87.89	0.805	237
Sample_SPA12.mb.50	90.32	1.245	279
Sample_SPA12.mb.36cbin.1	88.11	3.396	32
Sample_SPA12.mb.80	94.65	3.020	245
Sample_SPA12.mb.90	88.01	1.465	40
Sample_SPA12.mb.59	91.19	3.131	202
Sample_SPA12.mb.86	83.45	2.233	141
Sample_SPA12.mb.96	95.80	0.898	92
Sample_SPA12.mb.82	97.51	0.621	131

Sample_SPA12.mb.94	95.57	0.000	41
Sample_SPA12.mb.9cbin.1	97.27	0.000	22
Sample_SPA12.mb.37	94.05	0.384	320
Sample_SPA12.Cluster1195cbin.1	99.24	3.527	65
Sample_SPA12.mb.65	98.06	2.958	113
Sample_SPA13.Cluster985cbin.1	97.17	0.806	133
Sample_SPA13.Cluster7268cbin.1	98.65	0.335	15
Sample_SPA13.Cluster230	87.24	2.684	34
Sample_SPA13.mb.38	95.74	0.671	53
Sample_SPA13.mb.12	95.97	0.000	60
Sample_SPA13.mb.61cbin.1	81.14	2.586	702
Sample_SPA13.Cluster160	98.38	0.806	38
Sample_SPA13.mb.46	99.19	0.115	32
Sample_SPA13.mb.33	95.30	1.610	217
Sample_SPA13.Cluster5892	96.64	1.006	16
Sample_SPA13.mb.19cbin.1	95.55	2.095	184
Sample_SPA13.Cluster518	91.50	0.681	49
Sample_SPA13.Cluster288	99.54	3.598	32
Sample_SPA13.Cluster281	100.00	0.076	18
Sample_SPA13.Cluster824	98.75	0.625	34
Sample_SPA13.mb.47cbin.1	81.02	0.000	296
Sample_SPA13.mb.62cbin.1	92.61	0.671	44
Sample_SPA13.Cluster4841	99.51	1.449	77
Sample_SPA13.mb.3	97.93	0.136	121
Sample_SPA13.Cluster428cbin.1	99.31	0.371	47
Sample_SPA13.mb.74cbin.1	98.65	2.013	109
Sample_SPA13.mb.6	97.11	0.000	26
Sample_SPA13.mb.29	100.00	0.000	58
Sample_SPA13.mb.36	96.04	0.161	237
Sample_SPA13.Cluster2414cbin.1	96.52	1.610	51
Sample_SPA13.mb.49	88.27	4.766	445
Sample_SPA13.mb.24	98.39	1.160	107
Sample_SPA13.Cluster3020	99.61	0.512	57
Sample_SPA13.mb.50	99.62	0.000	19
Sample_SPA13.Cluster2319cbin.1	95.34	0.371	57
Sample_SPA13.mb.67cbin.1	97.49	3.481	93
Sample_SPA13.mb.65	84.14	2.083	238
Sample_SPA13.mb.86	98.30	1.518	35
Sample_SPA13.mb.69	98.83	0.000	73
Sample_SPA13.mb.64cbin.1	99.42	3.782	60
Sample_SPA15.Cluster10137	91.73	0.806	26
Sample_SPA15.mb.22	97.17	0.806	49
Sample_SPA15.Cluster344	81.20	0.671	27
Sample_SPA15.Cluster227cbin.1	90.17	2.071	208
Sample_SPA15.mb.23	81.33	3.020	124
Sample_SPA15.Cluster15391cbin.1	86.37	3.164	363
Sample_SPA15.mb.4	91.92	3.355	27
Sample_SPA15.mb.29	89.87	0.000	175
Sample_SPA15.mb.102cbin.1	88.81	1.027	237
Sample_SPA15.Cluster523	85.90	0.805	20
Sample_SPA15.mb.24cbin.1	89.26	3.355	54
Sample_SPA15.mb.38	94.09	1.342	131
Sample_SPA15.mb.28	86.74	1.342	59
Sample_SPA15.mb.59cbin.1	94.82	1.344	194
Sample_SPA15.Cluster3881	96.37	0.671	17
Sample_SPA15.mb.35	87.70	1.738	149
Sample_SPA15.Cluster7678cbin.1	98.80	2.245	72
Sample_SPA15.mb.58	92.05	3.691	92

Sample_SPA15.mb.54	87.11	2.705	137
Sample_SPA15.mb.65	88.63	1.342	42
Sample_SPA15.mb.81	85.79	3.601	226
Sample_SPA15.mb.91cbin.1	91.26	1.111	155
Sample_SPA15.mb.63	91.94	0.000	87
Sample_SPA15.mb.55	90.93	3.243	310
Sample_SPA15.mb.66	97.27	0.000	24
Sample_SPA15.mb.71	98.65	0.000	60
Sample_SPA15.mb.88	96.64	0.223	26
Sample_SPA15.Cluster8224cbin.1	96.39	0.743	236
Sample_SPA15.Cluster10540cbin.1	97.39	1.486	102
Sample_SPA15.mb.13cbin.1	93.14	1.762	652
Sample_SPA15.Cluster1062cbin.1	92.71	0.307	44
Sample_SPA15.mb.69cbin.1	97.86	0.508	69
Sample_SPA16.Cluster3423cbin.1	80.90	0.632	144
Sample_SPA16.Cluster10324cbin.1	91.64	2.594	65
Sample_SPA16.Cluster9401cbin.1	81.01	1.898	93
Sample_SPA16.Cluster3843	99.18	0.000	28
Sample_SPA16.Cluster2004cbin.1	97.77	0.000	82
Sample_SPA16.mb.2cbin.1	91.60	1.841	86
Sample_SPA16.mb.1	98.65	2.016	71
Sample_SPA16.mb.117cbin.1	84.83	3.117	221
Sample_SPA16.mb.59	97.81	0.671	167
Sample_SPA16.Cluster967cbin.1	90.62	0.000	102
Sample_SPA16.mb.38	86.07	3.888	278
Sample_SPA16.mb.46	85.88	1.898	207
Sample_SPA16.mb.65cbin.1	99.32	4.194	212
Sample_SPA16.mb.42	85.69	1.461	59
Sample_SPA16.Cluster6977cbin.1	93.23	0.241	80
Sample_SPA16.Cluster9846cbin.1	95.54	2.547	55
Sample_SPA16.mb.77	90.66	2.531	170
Sample_SPA16.Cluster3151cbin.1	94.48	0.000	85
Sample_SPA16.mb.39cbin.1	95.11	0.000	51
Sample_SPA16.mb.54	95.11	0.000	32
Sample_SPA16.mb.88	88.31	1.006	217
Sample_SPA16.mb.48	94.35	0.967	60
Sample_SPA16.mb.14cbin.1	92.89	3.734	307
Sample_SPA16.mb.9	90.72	2.210	191
Sample_SPA16.mb.69	99.90	0.454	23
Sample_SPA16.mb.99cbin.1	98.48	1.741	217
Sample_SPA17.Cluster7053cbin.1	82.75	0.000	337
Sample_SPA17.mb.52	92.61	0.000	19
Sample_SPA17.Cluster449	92.61	0.671	40
Sample_SPA17.mb.95cbin.1	81.37	1.724	49
Sample_SPA17.Cluster1797	96.46	0.000	59
Sample_SPA17.mb.44	96.51	2.004	126
Sample_SPA17.mb.17	90.50	1.265	263
Sample_SPA17.mb.79	85.90	2.348	205
Sample_SPA17.Cluster1537	98.80	1.497	20
Sample_SPA17.mb.41cbin.1	99.03	0.961	40
Sample_SPA17.mb.3cbin.1	100.00	0.806	44
Sample_SPA17.mb.39cbin.1	89.77	0.431	50
Sample_SPA17.mb.78cbin.1	99.36	0.000	41
Sample_SPA17.mb.36cbin.1	85.84	0.000	57
Sample_SPA17.mb.24	83.20	0.000	61
Sample_SPA17.Cluster883cbin.1	88.79	3.231	85
Sample_SPA17.mb.33cbin.1	82.72	1.577	530
Sample_SPA17.mb.76	82.55	2.534	73

Sample_SPA17.Cluster6320	96.88	0.000	18
Sample_SPA17.Cluster4831cbin.1	90.38	0.447	187
Sample_SPA17.mb.75cbin.1	96.87	0.000	55
Sample_SPA17.mb.98	89.44	0.000	60
Sample_SPA17.mb.8cbin.1	99.47	0.000	19
Sample_SPA17.Cluster6508	100.00	2.247	72
Sample_SPA17.mb.114	94.92	2.657	136
Sample_SPA17.mb.88cbin.1	87.34	0.738	150
Sample_SPA17.mb.70	81.22	1.489	143
Sample_SPA17.mb.16cbin.1	96.53	0.192	49
Sample_SPA17.mb.108cbin.1	92.16	0.000	91
Sample_SPA17.Cluster2740	99.42	0.384	33
Sample_SPA17.mb.97cbin.1	80.59	0.539	31
Sample_SPA20.mb.23	94.63	1.677	173
Sample_SPA20.mb.12cbin.1	98.65	1.565	117
Sample_SPA20.mb.36	99.32	1.230	69
Sample_SPA20.Cluster2573	99.18	0.543	19
Sample_SPA20.mb.5	88.00	1.078	80
Sample_SPA20.mb.32cbin.1	97.17	3.225	90
Sample_SPA20.Cluster1446cbin.1	95.59	0.000	83
Sample_SPA20.mb.39cbin.1	86.95	0.894	239
Sample_SPA20.mb.61cbin.1	92.72	1.265	274
Sample_SPA20.Cluster130	99.53	0.230	16
Sample_SPA20.mb.9	96.02	0.000	44
Sample_SPA20.mb.6cbin.1	91.94	1.677	26
Sample_SPA20.Cluster6883cbin.1	99.47	0.261	36
Sample_SPA20.Cluster4938cbin.1	80.40	0.292	46
Sample_SPA20.mb.24cbin.1	94.93	0.105	92
Sample_SPA20.mb.43	93.92	0.949	71
Sample_SPA20.Cluster2412cbin.1	96.62	0.621	103
Sample_SPA21.Cluster2186cbin.1	95.69	0.097	139
Sample_SPA21.mb.117	93.25	1.284	5
Sample_SPA21.Cluster11127cbin.1	87.70	2.237	294
Sample_SPA21.Cluster18	83.42	1.230	176
Sample_SPA21.mb.25	92.41	0.000	19
Sample_SPA21.mb.113cbin.1	92.06	1.515	90
Sample_SPA21.Cluster4618cbin.1	97.20	0.000	78
Sample_SPA21.mb.103	91.05	0.000	51
Sample_SPA21.Cluster6505cbin.1	97.09	0.671	83
Sample_SPA21.Cluster7151cbin.1	90.93	0.000	46
Sample_SPA21.mb.65	82.45	0.000	272
Sample_SPA21.Cluster10395cbin.1	92.61	0.335	39
Sample_SPA21.mb.110	90.82	1.464	62
Sample_SPA21.Cluster6611	94.29	3.355	25
Sample_SPA21.mb.116cbin.1	97.99	0.961	54
Sample_SPA21.mb.26	88.96	2.993	97
Sample_SPA21.Cluster125cbin.1	98.65	1.342	63
Sample_SPA21.Cluster7969	95.95	3.635	76
Sample_SPA21.mb.70cbin.1	88.54	4.865	117
Sample_SPA21.Cluster9049cbin.1	98.65	1.384	44
Sample_SPA21.mb.75cbin.1	91.94	3.376	76
Sample_SPA21.mb.118cbin.1	88.40	0.000	89
Sample_SPA21.mb.11	90.78	0.527	259
Sample_SPA21.mb.37cbin.1	100.00	0.480	44
Sample_SPA21.mb.13cbin.1	94.93	0.000	74
Sample_SPA21.mb.121	83.21	1.722	320
Sample_SPA21.mb.53	97.16	0.000	79
Sample_SPA21.mb.124	96.42	0.483	52

Sample_SPA21.mb.61	92.87	1.016	64
Sample_SPA21.mb.76	100.00	0.000	122
Sample_SPA21.mb.84	96.41	0.881	184
Sample_SPA21.mb.39cbin.1	92.34	0.000	45
Sample_SPA21.mb.79	94.90	1.886	313
Sample_SPA21.mb.2	95.66	1.883	91
Sample_SPA21.mb.114cbin.1	96.12	0.371	51
Sample_SPA21.mb.41cbin.1	94.80	1.923	136
Sample_SPA21.mb.60	97.94	1.923	47
Sample_SPA22.Cluster19998	94.94	2.407	26
Sample_SPA22.Cluster19730	91.01	0.561	11
Sample_SPA22.Cluster13759	98.87	1.123	33
Sample_SPA22.Cluster19925	98.66	0.111	7
Sample_SPA22.Cluster10053	92.17	0.671	17
Sample_SPA22.Cluster10703cbin.1	96.35	1.364	112
Sample_SPA22.Cluster19293cbin.1	89.74	1.139	28
Sample_SPA22.mb.10cbin.1	84.61	4.464	82
Sample_SPA22.Cluster18334cbin.1	83.12	3.113	290
Sample_SPA22.mb.108cbin.1	98.79	3.494	100
Sample_SPA22.mb.21	85.95	2.247	23
Sample_SPA22.Cluster358cbin.1	84.56	3.691	91
Sample_SPA22.mb.37	96.77	0.000	87
Sample_SPA22.mb.28cbin.1	85.12	0.268	60
Sample_SPA22.Cluster926cbin.1	87.36	1.342	36
Sample_SPA22.Cluster221cbin.1	86.40	2.040	315
Sample_SPA22.mb.14cbin.1	83.78	4.091	208
Sample_SPA22.Cluster14389cbin.1	91.82	0.041	51
Sample_SPA22.mb.8	93.82	2.247	10
Sample_SPA22.Cluster14174cbin.1	91.63	2.769	229
Sample_SPA22.Cluster8559	98.89	0.898	31
Sample_SPA22.mb.4	87.02	0.671	47
Sample_SPA22.mb.120	92.70	2.380	137
Sample_SPA22.mb.32cbin.1	96.12	4.838	197
Sample_SPA22.mb.36	97.87	0.000	62
Sample_SPA22.mb.91	80.16	1.748	49
Sample_SPA22.mb.2cbin.1	93.26	1.121	38
Sample_SPA22.Cluster1203cbin.1	97.51	0.000	191
Sample_SPA22.mb.77	95.97	0.671	44
Sample_SPA22.mb.122	97.09	0.000	28
Sample_SPA22.mb.58	88.05	1.342	35
Sample_SPA22.mb.96	85.18	1.510	218
Sample_SPA22.mb.53	91.44	3.368	245
Sample_SPA22.mb.82	89.93	1.189	38
Sample_SPA22.Cluster14732cbin.1	93.75	1.890	170
Sample_SPA22.mb.84	91.19	2.621	288
Sample_SPA22.mb.93	97.27	0.335	28
Sample_SPA22.mb.98cbin.1	89.18	0.961	102
Sample_SPA22.mb.41	99.62	0.000	38
Sample_SPA22.mb.110	92.89	2.803	157
Sample_SPA25.Cluster129cbin.1	90.36	3.322	195
Sample_SPA25.Cluster3919cbin.1	93.14	0.806	43
Sample_SPA25.Cluster11307	88.88	0.854	18
Sample_SPA25.Cluster5997	99.19	0.115	22
Sample_SPA25.Cluster1390cbin.1	86.95	2.818	357
Sample_SPA25.Cluster3488	91.94	0.000	18
Sample_SPA25.mb.57cbin.1	84.83	0.000	144
Sample_SPA25.Cluster557	93.54	0.806	42
Sample_SPA25.mb.13cbin.1	85.78	4.026	273

Sample_SPA25.mb.30cbin.1	89.59	1.342	45
Sample_SPA25.mb.42	89.00	4.194	202
Sample_SPA25.mb.68	99.30	1.864	87
Sample_SPA25.Cluster9308	95.03	0.335	32
Sample_SPA25.Cluster26cbin.1	86.56	0.134	300
Sample_SPA25.Cluster3311cbin.1	95.26	2.013	26
Sample_SPA25.Cluster1972	97.95	0.000	21
Sample_SPA25.Cluster5883	97.92	1.198	79
Sample_SPA25.mb.7cbin.1	89.26	1.006	99
Sample_SPA25.mb.41	94.55	0.480	128
Sample_SPA25.mb.45cbin.1	87.62	0.671	306
Sample_SPA25.mb.23cbin.1	99.42	3.117	169
Sample_SPA25.mb.71	97.58	0.879	118
Sample_SPA25.mb.28	96.10	0.000	58
Sample_SPA25.mb.86	91.58	0.032	20
Sample_SPA25.mb.92cbin.1	98.79	2.644	49
Sample_SPA25.mb.46cbin.1	98.22	0.000	203
Sample_SPA25.Cluster4646cbin.1	93.99	1.765	58
Sample_SPA25.mb.81	94.04	0.483	36
Sample_SPA25.mb.6cbin.1	99.27	0.523	24
Sample_SPA25.mb.99	95.97	0.335	67
Sample_SPA25.mb.95	98.49	1.132	26
Sample_SPA26.Cluster15006	92.69	1.123	21
Sample_SPA26.mb.104	92.41	2.247	32
Sample_SPA26.Cluster14380cbin.1	89.74	1.424	80
Sample_SPA26.Cluster6968cbin.1	85.19	0.671	30
Sample_SPA26.mb.36	92.41	2.247	18
Sample_SPA26.Cluster48cbin.1	93.95	2.852	185
Sample_SPA26.mb.17	93.26	1.342	34
Sample_SPA26.Cluster2066	97.14	0.000	28
Sample_SPA26.Cluster2008cbin.1	80.58	1.589	338
Sample_SPA26.mb.133	92.61	2.684	45
Sample_SPA26.Cluster4054cbin.1	92.47	1.115	74
Sample_SPA26.mb.20cbin.1	89.37	0.000	51
Sample_SPA26.Cluster5890cbin.1	97.39	1.250	106
Sample_SPA26.Cluster4627cbin.1	98.73	0.632	106
Sample_SPA26.Cluster1527cbin.1	96.20	0.625	202
Sample_SPA26.mb.130cbin.1	95.13	2.684	271
Sample_SPA26.Cluster12075	99.32	0.000	25
Sample_SPA26.mb.30	96.30	0.000	81
Sample_SPA26.Cluster751	100.00	0.909	20
Sample_SPA26.mb.27	100.00	1.612	41
Sample_SPA26.Cluster9645	97.09	0.000	21
Sample_SPA26.mb.13cbin.1	95.59	2.684	27
Sample_SPA26.mb.79	98.16	4.704	148
Sample_SPA26.mb.50	88.59	4.026	114
Sample_SPA26.mb.95	82.11	0.671	378
Sample_SPA26.Cluster986	90.00	2.596	31
Sample_SPA26.mb.23cbin.1	81.32	0.000	32
Sample_SPA26.Cluster10164cbin.1	99.41	0.076	77
Sample_SPA26.mb.45	97.31	0.000	11
Sample_SPA26.mb.15	98.80	0.000	28
Sample_SPA26.mb.57cbin.1	97.31	0.020	119
Sample_SPA26.mb.37	94.63	3.125	220
Sample_SPA26.mb.54	90.62	0.000	37
Sample_SPA26.mb.83	95.08	1.442	58
Sample_SPA26.mb.78	92.61	0.000	27
Sample_SPA26.mb.80cbin.1	98.65	0.335	46

Sample_SPA26.mb.89cbin.1	90.73	2.854	307
Sample_SPA26.mb.74cbin.1	97.16	1.132	219
Sample_SPA26.mb.59	96.18	0.836	116
Sample_SPA26.mb.90	93.54	1.075	191
Sample_SPA26.Cluster3042cbin.1	97.67	0.808	79
Sample_SPA27.Cluster2470cbin.1	94.96	0.000	88
Sample_SPA27.Cluster1062cbin.1	96.41	0.632	44
Sample_SPA27.mb.21cbin.1	89.42	3.062	181
Sample_SPA27.mb.27cbin.1	80.52	1.360	368
Sample_SPA27.Cluster283	98.23	0.961	26
Sample_SPA27.Cluster79cbin.1	80.33	0.454	240
Sample_SPA27.Cluster925cbin.1	94.40	2.944	230
Sample_SPA27.Cluster1827cbin.1	96.20	0.632	64
Sample_SPA27.Cluster213cbin.1	96.44	0.000	88
Sample_SPA27.Cluster2653cbin.1	99.51	0.483	83
Sample_SPA27.Cluster135cbin.1	97.53	0.000	101
Sample_SPA27.mb.37	90.09	3.197	378
Sample_SPA27.mb.19	97.46	0.116	145
Sample_SPA27.Cluster2782cbin.1	92.75	4.106	110
Sample_SPA27.Cluster943	96.16	4.228	73
Sample_SPA27.mb.1	93.84	0.000	61
Sample_SPA27.mb.26	97.36	4.957	233
Sample_SPA27.Cluster2785cbin.1	86.21	0.877	163
Sample_SPA27.mb.25	95.47	0.769	123
Sample_SPA27.Cluster632cbin.1	91.39	0.117	65
Sample_SPA28.Cluster10584	92.13	1.845	16
Sample_SPA28.Cluster9686cbin.1	89.09	1.342	216
Sample_SPA28.mb.87	81.89	2.586	165
Sample_SPA28.mb.17cbin.1	81.67	1.968	230
Sample_SPA28.mb.61	91.11	3.146	428
Sample_SPA28.mb.27	95.07	1.342	107
Sample_SPA28.mb.19	95.55	1.342	128
Sample_SPA28.mb.57	98.65	0.671	35
Sample_SPA28.Cluster5010	95.41	0.000	50
Sample_SPA28.mb.77	88.48	1.677	309
Sample_SPA28.Cluster35cbin.1	81.00	2.267	371
Sample_SPA28.Cluster9763cbin.1	91.64	0.707	74
Sample_SPA28.mb.51	93.01	3.626	72
Sample_SPA28.Cluster3006cbin.1	98.88	0.000	29
Sample_SPA28.Cluster5286	88.59	0.671	14
Sample_SPA28.mb.52	96.64	0.000	111
Sample_SPA28.mb.20cbin.1	96.83	0.021	95
Sample_SPA28.mb.24cbin.1	96.64	0.000	52
Sample_SPA28.Cluster8696cbin.1	98.65	1.006	39
Sample_SPA28.Cluster117	94.82	1.145	173
Sample_SPA28.Cluster5599	84.96	3.021	379
Sample_SPA28.mb.83cbin.1	94.36	2.900	247
Sample_SPA28.Cluster6267cbin.1	95.83	2.516	55
Sample_SPA28.Cluster9539	98.65	0.167	17
Sample_SPA28.mb.21cbin.1	98.10	1.045	56
Sample_SPA28.mb.70cbin.1	97.27	0.850	239
Sample_SPA28.mb.64	99.32	0.000	21
Sample_SPA28.mb.69	94.63	0.000	53
Sample_SPA28.mb.8cbin.1	93.73	0.632	114
Sample_SPA28.mb.37	86.28	3.035	322
Sample_SPA28.mb.73cbin.1	87.29	0.765	582
Sample_SPA30.Cluster12070	93.25	1.284	2
Sample_SPA30.mb.13	97.98	0.000	19

Sample_SPA30.mb.27	97.31	0.335	64
Sample_SPA30.Cluster444cbin.1	89.93	4.026	52
Sample_SPA30.mb.100	92.61	0.041	21
Sample_SPA30.Cluster87cbin.1	81.52	2.364	88
Sample_SPA30.Cluster1140cbin.1	96.97	0.671	95
Sample_SPA30.Cluster372cbin.1	90.60	2.729	87
Sample_SPA30.mb.41	97.90	1.165	75
Sample_SPA30.mb.22	84.20	1.006	38
Sample_SPA30.Cluster5439	99.40	0.998	21
Sample_SPA30.Cluster218cbin.1	94.31	4.919	106
Sample_SPA30.Cluster6069	99.98	1.497	18
Sample_SPA30.Cluster10669cbin.1	86.83	1.901	420
Sample_SPA30.mb.25	92.54	0.000	83
Sample_SPA30.Cluster371cbin.1	97.95	0.000	23
Sample_SPA30.mb.102cbin.1	94.58	1.845	50
Sample_SPA30.Cluster5550cbin.1	99.32	1.689	88
Sample_SPA30.Cluster7309cbin.1	96.57	0.000	59
Sample_SPA30.Cluster3188cbin.1	97.46	1.242	289
Sample_SPA30.Cluster11505cbin.1	95.56	0.632	75
Sample_SPA30.Cluster4953	96.93	2.230	67
Sample_SPA30.mb.63cbin.1	96.64	0.335	38
Sample_SPA30.mb.80	83.17	0.671	287
Sample_SPA30.mb.50	93.98	1.610	119
Sample_SPA30.Cluster6094	98.07	0.384	41
Sample_SPA30.mb.14cbin.1	95.89	1.127	116
Sample_SPA31.Cluster8294cbin.1	88.20	3.370	54
Sample_SPA31.mb.47	93.33	4.000	34
Sample_SPA31.mb.15	94.75	1.075	197
Sample_SPA31.mb.57cbin.1	96.37	2.150	134
Sample_SPA31.Cluster1487cbin.1	83.50	2.125	390
Sample_SPA31.mb.11cbin.1	87.58	0.671	65
Sample_SPA31.mb.74	93.60	2.013	59
Sample_SPA31.Cluster403cbin.1	97.07	0.806	81
Sample_SPA31.Cluster110	100.00	0.000	104
Sample_SPA31.mb.69	89.59	0.223	65
Sample_SPA31.Cluster5253cbin.1	96.40	1.006	36
Sample_SPA31.Cluster2324cbin.1	93.24	2.147	93
Sample_SPA31.mb.40	89.66	1.442	63
Sample_SPA31.mb.45cbin.1	91.94	1.342	182
Sample_SPA31.Cluster4489cbin.1	95.30	2.013	74
Sample_SPA31.mb.62	94.29	0.671	118
Sample_SPA31.Cluster5882cbin.1	97.09	1.342	23
Sample_SPA31.Cluster7476	98.29	0.261	97
Sample_SPA31.Cluster6198	96.57	0.000	34
Sample_SPA31.mb.79cbin.1	91.45	1.342	243
Sample_SPA31.mb.83	87.98	0.000	15
Sample_SPA31.mb.2cbin.1	98.83	0.584	81
Sample_SPA31.mb.68	95.43	3.125	32
Sample_SPA31.mb.7cbin.1	96.22	4.716	151
Sample_SPA31.mb.60cbin.1	98.73	1.898	247
Sample_SPA31.Cluster5757	98.88	0.743	59
Sample_SPA31.mb.67	95.18	0.384	90
Sample_SPA31.mb.82cbin.1	97.88	1.993	73
Sample_SPA31.mb.56cbin.1	95.20	0.737	191
Sample_SPA32.mb.10	93.54	0.000	48
Sample_SPA32.Cluster8317cbin.1	98.65	1.342	10
Sample_SPA32.Cluster6554	84.56	0.000	41
Sample_SPA32.Cluster5430cbin.1	98.65	0.671	62

Sample_SPA32.mb.41cbin.1	84.86	1.677	328
Sample_SPA32.Cluster831cbin.1	97.31	0.000	49
Sample_SPA32.Cluster3859	83.69	0.483	18
Sample_SPA32.Cluster1745	96.83	0.000	45
Sample_SPA32.mb.13	90.66	1.139	80
Sample_SPA32.mb.59cbin.1	84.22	0.671	134
Sample_SPA32.mb.79cbin.1	88.70	2.578	253
Sample_SPA32.Cluster317cbin.1	99.54	1.969	75
Sample_SPA32.mb.58cbin.1	91.44	1.956	230
Sample_SPA32.mb.80	93.95	0.000	61
Sample_SPA32.mb.53	96.13	1.932	73
Sample_SPA32.mb.2cbin.1	96.12	2.348	191
Sample_SPA32.mb.21	86.45	4.430	66
Sample_SPA32.mb.29cbin.1	97.31	2.147	119
Sample_SPA32.mb.57	99.03	0.000	24
Sample_SPA32.mb.67cbin.1	95.46	0.000	226
Sample_SPA32.Cluster37cbin.1	94.55	1.612	344
Sample_SPA32.mb.35	93.17	3.639	114
Sample_SPA32.mb.99cbin.1	96.31	0.961	25
Sample_SPA32.mb.75	92.08	2.272	263
Sample_SPA32.mb.92	94.64	0.483	327
Sample_SPA32.mb.55cbin.1	98.46	0.690	49
Sample_SPA34.Cluster14210	92.69	2.247	38
Sample_SPA34.Cluster14283cbin.1	81.46	3.370	38
Sample_SPA34.Cluster13844	88.88	0.854	20
Sample_SPA34.Cluster13142cbin.1	95.19	1.677	190
Sample_SPA34.Cluster236cbin.1	93.28	2.460	82
Sample_SPA34.Cluster5308cbin.1	91.56	2.292	215
Sample_SPA34.Cluster10977cbin.1	92.40	3.006	236
Sample_SPA34.mb.100	85.23	0.894	45
Sample_SPA34.Cluster2863	88.70	0.480	22
Sample_SPA34.mb.104	92.63	0.671	67
Sample_SPA34.Cluster3950cbin.1	97.31	0.000	21
Sample_SPA34.mb.10cbin.1	95.74	1.342	46
Sample_SPA34.mb.27cbin.1	94.18	0.000	97
Sample_SPA34.Cluster1738cbin.1	96.87	0.625	180
Sample_SPA34.mb.47	85.62	3.146	239
Sample_SPA34.Cluster2567	97.95	0.680	29
Sample_SPA34.mb.57cbin.1	96.10	2.083	156
Sample_SPA34.Cluster8839	97.98	0.671	18
Sample_SPA34.mb.106cbin.1	86.84	0.692	333
Sample_SPA34.Cluster10225	93.96	0.075	35
Sample_SPA34.mb.105cbin.1	96.64	0.335	29
Sample_SPA34.mb.113	89.93	0.000	16
Sample_SPA34.mb.40	89.93	0.671	50
Sample_SPA34.mb.21	95.22	0.000	182
Sample_SPA34.Cluster17cbin.1	96.82	1.612	355
Sample_SPA34.mb.55	80.73	0.533	240
Sample_SPA34.mb.62	86.56	3.747	134
Sample_SPA34.mb.102	92.78	0.016	20
Sample_SPA34.mb.93	82.43	0.000	43
Sample_SPA34.mb.53	89.22	0.000	140
Sample_SPA34.mb.94cbin.1	97.20	1.515	126
Sample_SPA34.mb.83	88.27	1.363	150
Sample_SPA34.mb.72cbin.1	90.60	3.355	99
Sample_SPA34.Cluster4722cbin.1	95.51	2.564	364
Sample_SPA34.Cluster6895cbin.1	98.21	0.495	62
Sample_SPA34.mb.79	87.97	1.342	197

Sample_SPA34.Cluster8898cbin.1	95.25	0.939	158
Sample_SPA34.mb.90cbin.1	95.84	1.449	115
Sample_SPA34.mb.92	99.32	0.671	50
Sample_SPA34.mb.97	93.28	1.342	165
Sample_SPA34.mb.56cbin.1	99.46	0.537	110
Sample_SPA34.mb.61cbin.1	86.91	1.022	86
Sample_SPA34.Cluster1818	99.10	0.112	28
Sample_SPA35.Cluster18259	92.41	3.370	34
Sample_SPA35.Cluster17834cbin.1	81.91	2.331	19
Sample_SPA35.Cluster14601	97.76	1.230	44
Sample_SPA35.Cluster14221cbin.1	94.79	2.055	142
Sample_SPA35.mb.100cbin.1	93.54	2.471	219
Sample_SPA35.Cluster9708	89.46	0.671	8
Sample_SPA35.mb.113	94.81	0.635	154
Sample_SPA35.Cluster432	81.94	1.565	102
Sample_SPA35.mb.10	91.94	0.000	37
Sample_SPA35.mb.32	88.23	0.940	119
Sample_SPA35.mb.22	96.77	0.000	33
Sample_SPA35.Cluster16310cbin.1	92.38	3.001	289
Sample_SPA35.mb.103cbin.1	86.69	2.223	212
Sample_SPA35.mb.47	90.45	0.000	22
Sample_SPA35.Cluster13092	97.09	0.000	24
Sample_SPA35.Cluster1826cbin.1	96.96	0.000	57
Sample_SPA35.mb.66	83.42	0.000	123
Sample_SPA35.mb.46	92.76	1.881	443
Sample_SPA35.Cluster4290cbin.1	92.91	3.935	180
Sample_SPA35.mb.50	83.28	1.398	410
Sample_SPA35.Cluster3665	97.27	0.000	24
Sample_SPA35.mb.65cbin.1	86.17	0.894	334
Sample_SPA35.mb.71cbin.1	87.06	2.797	29
Sample_SPA35.mb.102cbin.1	96.64	0.000	57
Sample_SPA35.mb.30	96.77	1.612	56
Sample_SPA35.mb.40cbin.1	94.26	3.355	138
Sample_SPA35.mb.49	88.49	3.355	96
Sample_SPA35.mb.58	85.06	0.838	204
Sample_SPA35.mb.23	100.00	0.000	28
Sample_SPA35.mb.3cbin.1	81.43	4.854	95
Sample_SPA35.mb.25	94.07	3.579	184
Sample_SPA35.mb.75cbin.1	85.77	2.109	325
Sample_SPA35.mb.114cbin.1	98.65	1.090	133
Sample_SPA35.mb.59	96.64	0.805	46
Sample_SPA35.mb.73	83.94	1.530	356
Sample_SPA35.mb.60cbin.1	99.54	0.000	14
Sample_SPA35.mb.87	89.70	4.474	279
Sample_SPA35.mb.93	95.04	0.781	95
Sample_SPA35.mb.98cbin.1	88.44	4.706	477
Sample_SPA35.Cluster1466	98.80	0.074	30
Sample_SPA36.Cluster12603	98.66	0.111	11
Sample_SPA36.mb.2	94.35	1.075	67
Sample_SPA36.Cluster108	85.23	0.671	21
Sample_SPA36.mb.40	88.69	0.774	128
Sample_SPA36.Cluster1221cbin.1	88.28	2.348	80
Sample_SPA36.mb.19cbin.1	80.27	1.740	401
Sample_SPA36.Cluster1524cbin.1	88.28	1.102	147
Sample_SPA36.Cluster1295cbin.1	93.73	1.342	87
Sample_SPA36.Cluster5207cbin.1	92.91	1.026	107
Sample_SPA36.mb.61	86.62	0.769	384
Sample_SPA36.mb.20	87.43	4.935	154

Sample_SPA36.Cluster6225cbin.1	94.63	0.335	79
Sample_SPA36.mb.13cbin.1	89.26	0.000	34
Sample_SPA36.Cluster8708cbin.1	98.99	0.000	69
Sample_SPA36.Cluster10796	98.65	0.000	21
Sample_SPA36.Cluster5539cbin.1	83.15	1.449	168
Sample_SPA36.Cluster12513	100.00	0.000	19
Sample_SPA36.Cluster3422	96.37	0.671	30
Sample_SPA36.mb.44cbin.1	98.55	2.013	79
Sample_SPA36.Cluster9710	99.32	0.671	19
Sample_SPA36.mb.62	85.72	2.430	438
Sample_SPA36.Cluster41	100.00	0.000	24
Sample_SPA36.mb.31	99.32	0.000	20
Sample_SPA36.mb.28cbin.1	93.91	0.000	18
Sample_SPA36.mb.18cbin.1	93.28	2.013	109
Sample_SPA36.mb.27	97.95	0.680	38
Sample_SPA36.mb.36	97.31	0.000	22
Sample_SPA36.Cluster10253cbin.1	98.64	1.689	87
Sample_SPA36.Cluster1302cbin.1	84.10	3.528	250
Sample_SPA36.mb.43	95.26	2.891	115
Sample_SPA36.mb.7cbin.1	87.03	2.257	291
Sample_SPA36.mb.68	97.60	1.097	54
Sample_SPA36.mb.83cbin.1	86.07	0.632	57
Sample_SPA36.mb.81	97.50	0.512	92
Sample_SPA37.Cluster17704	87.07	2.808	30
Sample_SPA37.Cluster17805	92.41	1.123	19
Sample_SPA37.mb.113	88.48	0.000	18
Sample_SPA37.mb.2	95.29	0.000	38
Sample_SPA37.mb.41	88.20	1.284	38
Sample_SPA37.mb.25	93.93	2.684	31
Sample_SPA37.Cluster340cbin.1	82.77	2.617	295
Sample_SPA37.mb.32	92.17	0.671	23
Sample_SPA37.Cluster3961	93.75	2.804	35
Sample_SPA37.Cluster15419cbin.1	97.58	0.335	40
Sample_SPA37.mb.78cbin.1	94.42	3.091	188
Sample_SPA37.Cluster6548	97.04	0.000	26
Sample_SPA37.mb.16cbin.1	94.32	0.000	81
Sample_SPA37.Cluster144cbin.1	80.30	4.370	318
Sample_SPA37.Cluster11952cbin.1	83.87	0.768	406
Sample_SPA37.Cluster18	93.48	1.303	316
Sample_SPA37.mb.61	94.74	0.671	82
Sample_SPA37.mb.69	99.32	2.055	145
Sample_SPA37.mb.1	94.97	2.445	270
Sample_SPA37.Cluster6932cbin.1	99.34	0.949	53
Sample_SPA37.mb.40	97.98	1.006	37
Sample_SPA37.mb.30	93.99	0.480	37
Sample_SPA37.mb.108cbin.1	97.07	1.754	88
Sample_SPA37.mb.8	97.09	1.923	227
Sample_SPA37.Cluster8131cbin.1	83.51	2.939	832
Sample_SPA37.mb.60cbin.1	99.35	2.898	135
Sample_SPA37.mb.72	99.11	0.591	271
Sample_SPA37.mb.75	98.86	1.509	42
Sample_SPA37.Cluster15692cbin.1	96.92	3.719	94
Sample_SPA37.mb.11cbin.1	93.24	4.679	486
Sample_SPA37.mb.94	98.12	0.349	76
Sample_SPA38.Cluster1699	90.82	1.342	90
Sample_SPA38.Cluster605	88.59	2.348	42
Sample_SPA38.Cluster9283cbin.1	92.72	0.969	224
Sample_SPA38.Cluster9cbin.1	88.47	3.041	265

Sample_SPA38.Cluster11102	99.32	1.006	15
Sample_SPA38.Cluster7150	99.98	1.497	25
Sample_SPA38.Cluster647cbin.1	96.59	0.000	248
Sample_SPA38.Cluster3464	100.00	0.000	23
Sample_SPA38.mb.113	97.58	2.016	86
Sample_SPA38.Cluster14202cbin.1	90.01	2.215	162
Sample_SPA38.mb.101cbin.1	93.62	0.000	56
Sample_SPA38.mb.26cbin.1	93.77	0.632	183
Sample_SPA38.mb.133	93.75	0.961	35
Sample_SPA38.Cluster11496cbin.1	92.87	3.128	367
Sample_SPA38.mb.134	94.63	0.000	20
Sample_SPA38.Cluster8789cbin.1	98.79	0.579	171
Sample_SPA38.mb.3cbin.1	86.80	1.342	58
Sample_SPA38.mb.57cbin.1	93.17	1.324	143
Sample_SPA38.mb.88	95.69	0.000	56
Sample_SPA38.Cluster5590cbin.1	98.74	0.371	51
Sample_SPA38.mb.38	98.07	0.961	34
Sample_SPA38.mb.71cbin.1	91.71	3.355	75
Sample_SPA38.mb.103cbin.1	97.66	2.317	137
Sample_SPA38.mb.66	87.39	1.587	192
Sample_SPA38.mb.70	85.99	1.677	238
Sample_SPA38.mb.40cbin.1	80.11	0.062	58
Sample_SPA38.mb.87cbin.1	88.59	2.684	146
Sample_SPA38.mb.31cbin.1	99.24	0.000	19
Sample_SPA38.mb.111cbin.1	99.23	0.512	73
Sample_SPA38.mb.91	95.82	3.834	79
Sample_SPA38.mb.74cbin.1	96.70	0.000	34
Sample_SPA38.mb.97cbin.1	100.00	0.513	55
Sample_SPA38.mb.124cbin.1	98.69	0.769	129
Sample_SPA38.mb.80cbin.1	82.36	1.240	190
Sample_SPA38.mb.41cbin.1	88.59	0.929	43
Sample_SPA38.mb.64	88.76	1.132	177
Sample_SPA39.mb.115	92.41	2.247	27
Sample_SPA39.Cluster11734cbin.1	91.05	2.189	49
Sample_SPA39.Cluster251cbin.1	91.94	0.000	34
Sample_SPA39.mb.108	87.99	0.066	205
Sample_SPA39.Cluster4992	93.88	2.974	28
Sample_SPA39.Cluster569cbin.1	97.31	0.671	45
Sample_SPA39.mb.134	96.50	1.165	73
Sample_SPA39.Cluster11372cbin.1	97.30	0.707	102
Sample_SPA39.mb.135	90.59	2.702	199
Sample_SPA39.Cluster1834cbin.1	95.67	0.961	44
Sample_SPA39.Cluster8588cbin.1	99.78	4.540	122
Sample_SPA39.mb.12	90.86	1.728	72
Sample_SPA39.Cluster3838	96.93	0.000	11
Sample_SPA39.Cluster11266	97.42	1.151	134
Sample_SPA39.mb.30cbin.1	96.77	0.000	25
Sample_SPA39.Cluster6116cbin.1	96.64	4.697	17
Sample_SPA39.Cluster65cbin.1	94.25	0.833	79
Sample_SPA39.mb.102cbin.1	98.73	3.186	93
Sample_SPA39.mb.29	92.56	0.675	110
Sample_SPA39.Cluster366	95.65	0.155	31
Sample_SPA39.mb.100cbin.1	90.05	2.995	110
Sample_SPA39.mb.27	98.38	0.953	21
Sample_SPA39.Cluster2121	100.00	0.000	37
Sample_SPA39.mb.119	92.53	0.240	30
Sample_SPA39.mb.58cbin.1	97.31	3.914	222
Sample_SPA39.mb.33	86.51	4.905	470

Sample_SPA39.mb.128cbin.1	94.60	0.606	44
Sample_SPA39.Cluster8650cbin.1	99.03	0.000	99
Sample_SPA39.mb.136	98.65	0.000	21
Sample_SPA39.mb.46cbin.1	94.63	0.000	141
Sample_SPA39.mb.113	98.52	2.483	136
Sample_SPA39.mb.47	92.49	0.671	43
Sample_SPA39.mb.64	95.15	0.671	220
Sample_SPA39.mb.6	95.30	1.698	56
Sample_SPA39.mb.49cbin.1	97.03	0.680	228
Sample_SPA39.mb.122cbin.1	86.55	0.555	501
Sample_SPA39.Cluster4019	98.41	1.740	110
Sample_SPA39.mb.40	98.63	0.000	40
Sample_SPA39.Cluster6486cbin.1	98.84	0.000	48
Sample_SPA39.mb.68	81.37	0.000	31
Sample_SPA39.mb.81	80.53	1.530	66
Sample_SPA39.Cluster6261	97.24	3.082	104
Sample_SPA39.mb.89cbin.1	99.24	0.000	43
Sample_SPA39.mb.80cbin.1	99.36	0.632	52
Sample_SPA39.mb.99cbin.1	89.67	0.480	37
Sample_SPA39.mb.96	91.82	2.593	82
Sample_SPA39.mb.34cbin.1	98.65	0.128	145
Sample_SPA41.Cluster6557cbin.1	97.55	4.838	18
Sample_SPA41.Cluster455cbin.1	98.38	1.075	50
Sample_SPA41.Cluster5568	92.59	1.677	21
Sample_SPA41.mb.21	83.78	0.000	114
Sample_SPA41.mb.11	95.29	0.745	210
Sample_SPA41.mb.25cbin.1	97.98	0.000	49
Sample_SPA41.Cluster9759	97.90	0.000	24
Sample_SPA41.Cluster11858	93.60	0.800	152
Sample_SPA41.Cluster519	80.53	3.691	35
Sample_SPA41.mb.12	86.03	0.671	185
Sample_SPA41.Cluster10261cbin.1	92.58	0.842	80
Sample_SPA41.Cluster6650cbin.1	99.18	0.000	23
Sample_SPA41.Cluster395cbin.1	88.03	3.691	332
Sample_SPA41.Cluster11436	100.00	0.000	41
Sample_SPA41.Cluster135cbin.1	89.59	3.389	288
Sample_SPA41.Cluster8883cbin.1	92.02	2.267	210
Sample_SPA41.mb.28	98.55	1.545	36
Sample_SPA41.mb.64	84.22	0.671	434
Sample_SPA41.Cluster10322cbin.1	97.64	0.000	118
Sample_SPA41.mb.40	84.47	1.190	161
Sample_SPA41.mb.52	82.55	2.930	155
Sample_SPA41.Cluster9423	99.58	0.070	23
Sample_SPA41.Cluster9689	99.32	0.335	31
Sample_SPA41.Cluster334cbin.1	89.54	0.000	26
Sample_SPA41.mb.41	80.74	1.723	286
Sample_SPA41.mb.110cbin.1	97.30	3.797	101
Sample_SPA41.mb.50cbin.1	93.00	0.671	108
Sample_SPA41.mb.88	85.79	0.158	74
Sample_SPA41.mb.62cbin.1	90.93	2.684	129
Sample_SPA41.mb.51cbin.1	94.49	0.671	79
Sample_SPA41.mb.55cbin.1	93.89	2.013	312
Sample_SPA41.mb.7cbin.1	92.28	4.758	57
Sample_SPA41.mb.92cbin.1	89.32	1.228	295
Sample_SPA41.mb.71	97.07	0.877	74
Sample_SPA41.Cluster5952	98.87	0.626	112
Sample_SPA41.Cluster7247	99.25	0.000	35
Sample_SPA41.Cluster4243cbin.1	97.92	1.336	153

Sample_SPA42.mb.113	88.31	0.268	120
Sample_SPA42.mb.44	89.50	2.150	348
Sample_SPA42.Cluster102cbin.1	92.37	3.187	112
Sample_SPA42.Cluster500	93.28	3.187	133
Sample_SPA42.Cluster5135cbin.1	93.95	0.000	53
Sample_SPA42.Cluster16495cbin.1	93.66	1.650	142
Sample_SPA42.mb.23	89.07	1.654	287
Sample_SPA42.Cluster5583cbin.1	96.65	0.000	48
Sample_SPA42.mb.32cbin.1	93.91	3.603	83
Sample_SPA42.Cluster10090	97.09	0.000	21
Sample_SPA42.mb.75cbin.1	93.95	2.016	46
Sample_SPA42.mb.107cbin.1	99.32	1.006	52
Sample_SPA42.mb.72cbin.1	97.84	0.000	68
Sample_SPA42.mb.29cbin.1	84.30	3.392	311
Sample_SPA42.Cluster634	99.55	1.407	28
Sample_SPA42.Cluster9771cbin.1	84.68	3.216	371
Sample_SPA42.mb.82cbin.1	89.26	1.250	49
Sample_SPA42.mb.53cbin.1	95.30	0.000	78
Sample_SPA42.mb.4cbin.1	84.82	3.962	305
Sample_SPA42.mb.33cbin.1	98.75	1.242	81
Sample_SPA42.mb.64cbin.1	93.64	1.174	186
Sample_SPA42.mb.73cbin.1	96.15	0.000	82
Sample_SPA42.mb.74	96.63	3.125	27
Sample_SPA42.Cluster10643cbin.1	93.03	2.736	285
Sample_SPA42.mb.88cbin.1	97.91	0.576	59
Sample_SPA42.mb.85	97.64	0.000	80
Sample_SPA42.mb.7	99.62	0.000	16
Sample_SPA42.mb.78cbin.1	82.52	1.720	569
Sample_SPA42.mb.77	83.28	2.661	364
Sample_SPA45.Cluster4144	87.54	0.000	15
Sample_SPA45.Cluster5872	96.30	0.000	66
Sample_SPA45.Cluster669cbin.1	93.95	2.348	97
Sample_SPA45.mb.122cbin.1	89.00	2.531	112
Sample_SPA45.mb.18cbin.1	96.94	1.677	117
Sample_SPA45.Cluster12792cbin.1	90.37	0.000	413
Sample_SPA45.mb.22cbin.1	86.68	0.223	46
Sample_SPA45.Cluster7259cbin.1	93.73	0.000	75
Sample_SPA45.Cluster9319	99.32	1.006	20
Sample_SPA45.mb.105cbin.1	95.49	1.698	284
Sample_SPA45.mb.26	99.19	0.604	34
Sample_SPA45.Cluster5932cbin.1	98.98	1.745	94
Sample_SPA45.Cluster10018cbin.1	91.47	0.724	266
Sample_SPA45.mb.45cbin.1	88.25	0.000	19
Sample_SPA45.Cluster8060cbin.1	84.95	1.026	295
Sample_SPA45.mb.117cbin.1	91.52	1.169	146
Sample_SPA45.mb.67	99.30	0.932	79
Sample_SPA45.mb.56	95.35	2.215	168
Sample_SPA45.mb.101	92.98	1.169	54
Sample_SPA45.mb.65	96.64	0.335	79
Sample_SPA45.mb.23cbin.1	88.39	4.529	220
Sample_SPA45.mb.86	97.20	2.684	106
Sample_SPA45.mb.70	81.86	3.888	424
Sample_SPA45.mb.59cbin.1	87.80	1.542	244
Sample_SPA45.mb.8cbin.1	97.31	1.006	38
Sample_SPA45.mb.48cbin.1	94.63	0.671	24
Sample_SPA45.mb.71cbin.1	95.56	1.898	101
Sample_SPA45.Cluster3337cbin.1	95.44	3.481	558
Sample_SPA45.mb.37	93.11	4.975	43

Sample_SPA45.Cluster5564	96.10	0.384	93
Sample_SPA45.mb.73cbin.1	98.92	0.806	46
Sample_SPA45.mb.93	94.60	1.825	64
Sample_SPA45.mb.104cbin.1	97.45	1.486	115
Sample_SPA45.mb.85cbin.1	98.20	1.197	85
Sample_SPA45.mb.82cbin.1	98.80	0.800	174
Sample_SPA45.mb.72	95.34	1.789	84
Sample_SPA45.mb.90	95.91	0.226	72
Sample_SPA45.mb.98cbin.1	95.43	0.549	213
Sample_SPA45.mb.91	98.11	0.000	91
Sample_SPA45.mb.87	85.27	1.165	346
Sample_SPA45.mb.61	93.38	0.913	299
Sample_SPA45.mb.5cbin.1	89.23	0.576	97
Sample_SPA45.mb.95cbin.1	99.59	0.165	73
Sample_SPA47.Cluster11721	90.44	3.932	19
Sample_SPA47.Cluster4099	96.62	0.000	5
Sample_SPA47.Cluster10668cbin.1	95.97	4.250	309
Sample_SPA47.Cluster8596cbin.1	95.74	0.671	73
Sample_SPA47.Cluster8125cbin.1	95.60	0.000	28
Sample_SPA47.Cluster10912cbin.1	89.75	0.842	110
Sample_SPA47.Cluster5842cbin.1	91.58	1.677	229
Sample_SPA47.Cluster54cbin.1	94.21	2.013	269
Sample_SPA47.Cluster5816	89.82	0.798	12
Sample_SPA47.Cluster344cbin.1	92.61	3.020	111
Sample_SPA47.Cluster1713cbin.1	97.31	1.132	10
Sample_SPA47.Cluster251cbin.1	95.05	2.176	104
Sample_SPA47.mb.31	93.54	0.806	37
Sample_SPA47.mb.119cbin.1	94.89	0.115	150
Sample_SPA47.Cluster268	96.25	1.250	19
Sample_SPA47.Cluster8012	95.30	1.006	20
Sample_SPA47.mb.102	90.28	0.806	101
Sample_SPA47.mb.103cbin.1	97.96	0.000	40
Sample_SPA47.mb.117	94.63	1.342	38
Sample_SPA47.Cluster903	98.75	0.625	22
Sample_SPA47.Cluster980	95.00	3.712	18
Sample_SPA47.Cluster10cbin.1	92.61	2.860	168
Sample_SPA47.mb.112cbin.1	94.72	1.530	249
Sample_SPA47.Cluster310cbin.1	95.74	0.480	44
Sample_SPA47.mb.16	91.94	0.671	51
Sample_SPA47.mb.30cbin.1	88.25	0.000	27
Sample_SPA47.mb.42	98.32	0.671	108
Sample_SPA47.Cluster89cbin.1	93.62	2.227	168
Sample_SPA47.mb.36cbin.1	89.19	1.342	77
Sample_SPA47.Cluster6035	95.47	0.000	33
Sample_SPA47.mb.29	91.27	1.342	275
Sample_SPA47.mb.49cbin.1	82.20	2.531	254
Sample_SPA47.mb.39cbin.1	91.69	1.442	30
Sample_SPA47.Cluster6029	98.92	0.000	51
Sample_SPA47.mb.34	83.41	0.284	330
Sample_SPA47.mb.11	91.25	1.492	140
Sample_SPA47.mb.84cbin.1	96.15	4.801	278
Sample_SPA47.mb.68	92.93	1.265	238
Sample_SPA47.Cluster4528	99.61	0.576	79
Sample_SPA47.mb.87cbin.1	92.28	0.671	140
Sample_SPA47.mb.54cbin.1	100.00	2.163	36
Sample_SPA47.Cluster7793cbin.1	97.60	0.769	227
Sample_SPA47.mb.27cbin.1	95.90	1.461	64
Sample_SPA47.mb.74	88.90	3.846	49

Sample_SPA47.mb.64	98.65	0.447	21
Sample_SPA47.mb.86	89.26	0.000	33
Sample_SPA47.mb.43	97.39	2.287	295
Sample_SPA47.mb.6	99.46	0.000	53
Sample_SPB03.Cluster3771	91.39	0.097	48
Sample_SPB03.Cluster911cbin.1	99.30	2.913	84
Sample_SPB03.mb.105	96.64	0.000	36
Sample_SPB03.mb.3	98.65	0.000	17
Sample_SPB03.Cluster3812cbin.1	93.62	0.000	36
Sample_SPB03.mb.42	97.31	1.342	53
Sample_SPB03.mb.65cbin.1	92.93	1.342	29
Sample_SPB03.mb.4	97.76	0.671	99
Sample_SPB03.mb.50cbin.1	86.44	3.914	305
Sample_SPB03.mb.74cbin.1	81.03	0.862	155
Sample_SPB03.Cluster36cbin.1	89.65	2.822	368
Sample_SPB03.mb.56	93.73	0.671	73
Sample_SPB03.Cluster323	94.52	0.481	133
Sample_SPB03.mb.48	85.21	0.503	182
Sample_SPB03.Cluster9294	99.32	1.342	21
Sample_SPB03.mb.47	91.94	0.000	68
Sample_SPB03.mb.64cbin.1	83.14	2.005	346
Sample_SPB03.mb.5	97.31	0.671	11
Sample_SPB03.mb.22	98.38	2.726	57
Sample_SPB03.mb.81	90.01	1.342	306
Sample_SPB03.mb.28	89.03	2.852	234
Sample_SPB03.mb.7	98.99	0.000	40
Sample_SPB03.mb.2cbin.1	93.92	0.185	51
Sample_SPB03.mb.89	95.14	2.465	69
Sample_SPB03.mb.92	89.18	1.923	40
Sample_SPB03.mb.55	99.24	0.000	11
Sample_SPB03.mb.67	98.95	1.690	78
Sample_SPB03.mb.79cbin.1	93.68	0.689	369
Sample_SPB03.mb.60cbin.1	94.90	0.705	273
Sample_SPB03.mb.78	89.60	3.794	302
Sample_SPB03.mb.100cbin.1	98.89	0.303	135
Sample_SPB03.mb.35cbin.1	97.90	0.136	57
Sample_SPB04.mb.16	90.16	4.494	179
Sample_SPB04.mb.37	93.41	2.664	32
Sample_SPB04.Cluster2864cbin.1	95.97	0.000	32
Sample_SPB04.mb.53cbin.1	91.70	1.196	254
Sample_SPB04.mb.15	83.53	3.225	419
Sample_SPB04.Cluster3664cbin.1	98.14	0.000	60
Sample_SPB04.Cluster4111	99.40	0.898	76
Sample_SPB04.mb.61cbin.1	97.04	0.632	120
Sample_SPB04.mb.24	82.59	4.570	143
Sample_SPB04.Cluster4530cbin.1	89.77	0.119	126
Sample_SPB04.Cluster383cbin.1	95.91	0.000	26
Sample_SPB04.Cluster1539cbin.1	88.58	0.433	213
Sample_SPB04.mb.69cbin.1	95.86	3.401	291
Sample_SPB04.Cluster9349cbin.1	98.65	0.335	47
Sample_SPB04.mb.44	94.63	0.671	31
Sample_SPB04.mb.56cbin.1	96.93	0.000	14
Sample_SPB04.mb.7cbin.1	95.71	4.182	135
Sample_SPB04.Cluster2090cbin.1	98.22	0.000	166
Sample_SPB04.mb.60cbin.1	98.00	4.911	121
Sample_SPB04.mb.67cbin.1	87.92	4.335	380
Sample_SPB04.Cluster4564cbin.1	99.23	1.293	134
Sample_SPB04.mb.32cbin.1	96.22	1.153	283

Sample_SPB04.Cluster3304cbin.1	99.19	0.358	47
Sample_SPB06.Cluster12077	80.16	2.447	24
Sample_SPB06.mb.101cbin.1	91.57	2.247	32
Sample_SPB06.mb.109	89.33	1.333	35
Sample_SPB06.Cluster305cbin.1	81.72	4.362	257
Sample_SPB06.Cluster460cbin.1	93.28	2.348	69
Sample_SPB06.Cluster5391cbin.1	94.51	2.589	44
Sample_SPB06.Cluster11221	84.56	0.000	38
Sample_SPB06.mb.121	89.93	0.000	78
Sample_SPB06.Cluster5816	97.98	0.335	17
Sample_SPB06.Cluster10276	83.54	0.305	286
Sample_SPB06.Cluster11862	82.27	0.632	30
Sample_SPB06.Cluster230	100.00	1.777	27
Sample_SPB06.mb.22cbin.1	99.30	0.932	81
Sample_SPB06.Cluster6548	99.32	1.006	16
Sample_SPB06.Cluster483	97.95	0.000	24
Sample_SPB06.mb.124cbin.1	93.95	0.671	154
Sample_SPB06.Cluster79cbin.1	100.00	0.268	49
Sample_SPB06.Cluster6857	90.09	3.547	33
Sample_SPB06.Cluster11209	98.65	0.671	23
Sample_SPB06.mb.100	98.65	0.000	31
Sample_SPB06.mb.30cbin.1	81.67	0.793	273
Sample_SPB06.mb.27	95.13	1.006	155
Sample_SPB06.mb.139	94.40	0.681	142
Sample_SPB06.Cluster8017	95.74	3.635	74
Sample_SPB06.mb.115cbin.1	97.83	0.000	96
Sample_SPB06.mb.132cbin.1	96.94	0.185	75
Sample_SPB06.mb.126	97.31	0.671	49
Sample_SPB06.mb.66	93.93	1.342	20
Sample_SPB06.mb.55	96.94	0.793	37
Sample_SPB06.mb.20	85.19	2.210	104
Sample_SPB06.mb.33cbin.1	86.24	1.136	85
Sample_SPB06.mb.48	93.28	2.013	78
Sample_SPB06.mb.46cbin.1	97.27	0.000	24
Sample_SPB06.Cluster5860cbin.1	99.19	0.576	42
Sample_SPB06.Cluster6553cbin.1	91.67	1.503	64
Sample_SPB06.mb.74cbin.1	85.67	1.197	145
Sample_SPB06.mb.81	80.94	1.342	207
Sample_SPB06.Cluster7281cbin.1	97.88	0.576	38
Sample_SPB06.mb.97	96.42	1.372	84
Sample_SPB06.mb.83cbin.1	98.38	0.806	24
Sample_SPB06.mb.44	98.65	0.000	77
Sample_SPB06.mb.54cbin.1	87.45	4.628	363
Sample_SPB06.mb.93	93.90	0.000	62
Sample_SPB06.mb.65	81.45	2.215	282
Sample_SPB06.Cluster6571cbin.1	99.46	0.537	103
Sample_SPB06.mb.96cbin.1	87.87	0.909	298
Sample_SPB06.mb.89cbin.1	97.46	0.105	110
Sample_SPB06.mb.88cbin.1	94.03	3.283	146
Sample_SPB06.mb.25	86.51	2.106	81
Sample_SPB07.Cluster10588cbin.1	92.69	2.407	27
Sample_SPB07.Cluster10626	91.57	1.123	13
Sample_SPB07.Cluster3324cbin.1	92.47	0.000	191
Sample_SPB07.mb.19	92.41	4.494	22
Sample_SPB07.Cluster127cbin.1	87.91	0.000	52
Sample_SPB07.Cluster2229cbin.1	93.06	0.000	130
Sample_SPB07.mb.109	86.57	2.013	211
Sample_SPB07.mb.105cbin.1	90.71	4.697	254

Sample_SPB07.mb.44	87.44	2.013	83
Sample_SPB07.mb.47	92.55	1.677	178
Sample_SPB07.Cluster1982	100.00	0.000	33
Sample_SPB07.Cluster1478	98.75	0.625	29
Sample_SPB07.Cluster5497cbin.1	93.56	0.350	72
Sample_SPB07.Cluster153cbin.1	92.85	0.000	230
Sample_SPB07.mb.87cbin.1	82.45	0.000	114
Sample_SPB07.mb.104	93.92	0.986	200
Sample_SPB07.Cluster1836	100.00	0.227	11
Sample_SPB07.Cluster283	99.55	0.000	34
Sample_SPB07.Cluster8450	96.64	1.677	40
Sample_SPB07.mb.31cbin.1	87.81	0.000	81
Sample_SPB07.mb.42	80.19	1.449	72
Sample_SPB07.Cluster3014cbin.1	96.95	0.754	79
Sample_SPB07.mb.2	83.22	0.000	19
Sample_SPB07.mb.50	88.63	1.814	235
Sample_SPB07.Cluster7656	91.18	0.483	72
Sample_SPB07.mb.38cbin.1	99.19	0.000	112
Sample_SPB07.mb.30	85.89	0.000	27
Sample_SPB07.mb.66	98.03	0.223	177
Sample_SPB07.mb.4cbin.1	99.51	0.961	35
Sample_SPB07.mb.86	91.60	1.697	79
Sample_SPB07.mb.14cbin.1	94.63	0.701	52
Sample_SPB07.mb.48	87.21	4.202	297
Sample_SPB07.mb.94cbin.1	82.82	0.443	291
Sample_SPB07.mb.49	93.61	3.599	147
Sample_SPB07.Cluster6051cbin.1	99.46	0.000	75
Sample_SPB07.Cluster4507cbin.1	99.25	0.000	36
Sample_SPB07.Cluster4015cbin.1	84.92	4.973	725
Sample_SPB07.Cluster5231	99.23	0.961	52
Sample_SPB07.mb.72	91.28	0.724	46
Sample_SPB07.mb.75	83.11	2.726	191
Sample_SPB07.mb.7cbin.1	92.61	3.855	82
Sample_SPB07.mb.89	89.89	2.611	82
Sample_SPB08.Cluster2354cbin.1	81.37	1.724	404
Sample_SPB08.Cluster4881	92.26	1.342	23
Sample_SPB08.Cluster340cbin.1	87.13	1.342	49
Sample_SPB08.Cluster151	89.26	0.335	26
Sample_SPB08.mb.31	93.33	4.666	57
Sample_SPB08.Cluster3815cbin.1	95.97	0.000	181
Sample_SPB08.Cluster8127	99.19	0.806	8
Sample_SPB08.Cluster4330cbin.1	98.08	1.497	90
Sample_SPB08.Cluster5760	88.29	0.000	16
Sample_SPB08.mb.46	94.85	0.000	98
Sample_SPB08.mb.5	98.38	3.225	125
Sample_SPB08.mb.13cbin.1	82.99	0.000	44
Sample_SPB08.Cluster2943cbin.1	97.31	0.000	13
Sample_SPB08.mb.25cbin.1	81.20	0.776	63
Sample_SPB08.Cluster6125	100.00	0.480	11
Sample_SPB08.mb.53	98.60	1.165	102
Sample_SPB08.mb.38	92.03	1.062	259
Sample_SPB08.mb.63	89.26	0.000	30
Sample_SPB08.mb.55	84.00	0.671	39
Sample_SPB08.Cluster7895	87.69	0.000	19
Sample_SPB08.Cluster6032cbin.1	95.30	1.510	232
Sample_SPB08.mb.116	93.28	1.067	106
Sample_SPB08.mb.114	99.03	1.730	63
Sample_SPB08.Cluster8080	99.32	2.348	22

Sample_SPB08.mb.50	91.19	2.380	237
Sample_SPB08.mb.7	85.90	1.342	77
Sample_SPB08.mb.58cbin.1	80.50	1.696	103
Sample_SPB08.mb.70cbin.1	89.20	2.584	360
Sample_SPB08.mb.101cbin.1	96.56	0.671	50
Sample_SPB08.mb.65	99.10	3.355	76
Sample_SPB08.mb.59cbin.1	87.03	4.988	127
Sample_SPB08.mb.86	85.23	0.671	35
Sample_SPB08.mb.26cbin.1	98.38	0.806	194
Sample_SPB08.Cluster8661cbin.1	91.21	1.125	262
Sample_SPB08.Cluster4205cbin.1	95.96	1.503	330
Sample_SPB08.mb.62	88.81	4.807	34
Sample_SPB08.mb.69	97.10	0.483	45
Sample_SPB08.mb.74cbin.1	95.63	0.447	75
Sample_SPB08.mb.79	96.79	2.163	132
Sample_SPB08.mb.87cbin.1	95.29	0.092	57
Sample_SPB09.Cluster19cbin.1	85.23	0.866	210
Sample_SPB09.mb.30	98.60	1.165	89
Sample_SPB09.mb.19	95.41	0.671	82
Sample_SPB09.Cluster7564cbin.1	98.10	1.582	95
Sample_SPB09.mb.5cbin.1	84.56	0.503	336
Sample_SPB09.mb.20cbin.1	92.61	0.000	23
Sample_SPB09.Cluster332cbin.1	96.25	3.582	237
Sample_SPB09.mb.50cbin.1	95.47	2.348	174
Sample_SPB09.mb.2	97.98	0.000	11
Sample_SPB09.Cluster116	99.55	0.000	45
Sample_SPB09.Cluster5574	89.04	0.000	29
Sample_SPB09.Cluster1064	96.22	0.000	44
Sample_SPB09.mb.85cbin.1	90.92	3.850	257
Sample_SPB09.mb.27	99.32	0.000	31
Sample_SPB09.Cluster245cbin.1	84.08	1.586	38
Sample_SPB09.Cluster8333cbin.1	98.65	0.000	87
Sample_SPB09.mb.88cbin.1	92.61	2.327	108
Sample_SPB09.mb.67	97.10	2.584	61
Sample_SPB09.mb.61	95.30	3.846	29
Sample_SPB09.mb.32	99.03	0.483	49
Sample_SPB09.mb.64	93.59	0.352	262
Sample_SPB09.mb.54cbin.1	100.00	0.105	63
Sample_SPB09.mb.56	99.24	0.000	19
Sample_SPB09.Cluster3968	98.92	0.017	228
Sample_SPB09.mb.24cbin.1	85.68	2.205	45
Sample_SPB09.mb.82cbin.1	90.19	1.691	246
Sample_SPB09.mb.46cbin.1	95.64	2.403	263
Sample_SPB09.mb.8cbin.1	97.73	0.632	124
Sample_SPB10.Cluster7361cbin.1	83.66	0.671	25
Sample_SPB10.Cluster4204cbin.1	95.37	0.782	173
Sample_SPB10.Cluster2637cbin.1	97.67	1.898	33
Sample_SPB10.mb.63	90.60	0.335	10
Sample_SPB10.Cluster1613cbin.1	91.13	0.021	62
Sample_SPB10.mb.73	88.93	1.403	208
Sample_SPB10.Cluster7560	95.28	0.000	56
Sample_SPB10.Cluster5073cbin.1	84.55	2.715	296
Sample_SPB10.mb.60cbin.1	85.02	0.632	82
Sample_SPB10.mb.9	92.61	0.671	26
Sample_SPB10.Cluster192	98.40	0.000	62
Sample_SPB10.Cluster6869cbin.1	97.98	0.000	30
Sample_SPB10.Cluster6006	98.63	0.000	37
Sample_SPB10.mb.71cbin.1	97.98	1.971	88

Sample_SPB10.mb.58	95.63	0.335	40
Sample_SPB10.mb.10cbin.1	97.36	1.360	102
Sample_SPB10.Cluster4063	99.51	0.241	70
Sample_SPB10.mb.70cbin.1	92.97	1.020	287
Sample_SPB10.Cluster12cbin.1	89.71	0.787	480
Sample_SPB10.mb.36cbin.1	98.32	0.469	84
Sample_SPB10.mb.6cbin.1	98.60	1.140	101
Sample_SPB10.mb.29	94.15	4.210	107
Sample_SPB10.mb.77	99.21	1.207	61
Sample_SPB10.mb.54cbin.1	96.51	0.105	287
Sample_SPB10.Cluster2695cbin.1	92.53	0.123	51
Sample_SPB10.mb.53	97.41	0.185	43
Sample_SPB10.Cluster4934cbin.1	92.83	0.791	142
Sample_SPB10.mb.28	97.74	0.208	44
Sample_SPB10.mb.67cbin.1	90.69	3.642	699
Sample_SPB11.Cluster5576	98.63	1.342	28
Sample_SPB11.Cluster2626	97.04	0.949	33
Sample_SPB11.mb.24	91.24	1.403	295
Sample_SPB11.Cluster14	97.19	1.027	210
Sample_SPB11.Cluster1613cbin.1	97.31	0.000	152
Sample_SPB11.mb.54	98.60	1.165	191
Sample_SPB11.Cluster96cbin.1	95.41	4.647	283
Sample_SPB11.mb.58	89.77	1.282	298
Sample_SPB11.Cluster2830	92.21	0.898	39
Sample_SPB11.Cluster7987cbin.1	97.30	0.134	194
Sample_SPB11.Cluster12cbin.1	84.59	2.856	289
Sample_SPB11.Cluster6266	99.32	1.006	16
Sample_SPB11.Cluster1895	97.27	0.000	16
Sample_SPB11.mb.1	96.37	1.449	59
Sample_SPB11.Cluster4339	92.61	0.335	19
Sample_SPB11.mb.4	94.19	0.316	218
Sample_SPB11.mb.3	89.18	0.961	23
Sample_SPB11.Cluster4404	83.22	0.000	19
Sample_SPB11.Cluster746	98.75	0.625	25
Sample_SPB11.mb.63cbin.1	91.27	0.939	66
Sample_SPB11.mb.80cbin.1	97.31	0.921	132
Sample_SPB11.mb.39	82.43	2.559	254
Sample_SPB11.mb.66	100.00	0.806	30
Sample_SPB11.mb.10	86.95	0.483	45
Sample_SPB11.Cluster7487cbin.1	98.63	1.405	86
Sample_SPB11.mb.53cbin.1	89.47	0.584	69
Sample_SPB11.mb.13cbin.1	97.35	0.000	74
Sample_SPB11.mb.47	87.04	1.007	121
Sample_SPB11.Cluster2040cbin.1	96.67	2.135	86
Sample_SPB11.mb.73cbin.1	98.63	0.000	29
Sample_SPB11.Cluster3214cbin.1	98.14	0.929	47
Sample_SPB11.mb.74	84.28	1.809	157
Sample_SPB11.mb.8cbin.1	94.84	4.622	308
Sample_SPB11.Cluster1227cbin.1	95.48	3.609	356
Sample_SPB12.Cluster19582	91.57	1.123	14
Sample_SPB12.Cluster18884	89.74	0.854	7
Sample_SPB12.Cluster6377cbin.1	95.96	1.169	57
Sample_SPB12.Cluster3162cbin.1	93.00	0.000	96
Sample_SPB12.mb.124	87.91	0.335	231
Sample_SPB12.mb.127	91.30	0.466	356
Sample_SPB12.Cluster4807cbin.1	88.10	1.851	166
Sample_SPB12.mb.131	97.20	3.131	241
Sample_SPB12.mb.15	97.98	0.000	13

Sample_SPB12.Cluster13513cbin.1	97.31	0.894	65
Sample_SPB12.mb.138	98.32	0.671	59
Sample_SPB12.Cluster1418	97.95	0.000	22
Sample_SPB12.Cluster18261	99.47	0.000	36
Sample_SPB12.Cluster11654cbin.1	90.57	1.183	173
Sample_SPB12.mb.34	90.55	0.000	22
Sample_SPB12.Cluster197	99.37	0.621	62
Sample_SPB12.mb.101cbin.1	97.27	0.000	20
Sample_SPB12.mb.4	97.98	1.342	47
Sample_SPB12.Cluster9121cbin.1	94.47	2.657	200
Sample_SPB12.mb.132cbin.1	89.09	0.984	99
Sample_SPB12.mb.33	82.14	2.521	241
Sample_SPB12.mb.94	93.33	3.030	36
Sample_SPB12.mb.121	98.22	0.000	39
Sample_SPB12.mb.10cbin.1	85.99	2.824	332
Sample_SPB12.mb.128cbin.1	88.86	0.754	32
Sample_SPB12.mb.43cbin.1	95.46	2.339	53
Sample_SPB12.mb.29cbin.1	94.01	1.497	110
Sample_SPB12.mb.35	83.09	1.077	362
Sample_SPB12.mb.23	97.31	0.000	25
Sample_SPB12.mb.21	97.98	0.000	22
Sample_SPB12.mb.51cbin.1	84.56	0.335	19
Sample_SPB12.mb.37	98.65	1.342	46
Sample_SPB12.mb.47	95.98	0.966	52
Sample_SPB12.mb.52	97.98	0.000	28
Sample_SPB12.mb.99cbin.1	90.60	0.000	82
Sample_SPB12.Cluster6415cbin.1	96.60	1.765	214
Sample_SPB12.mb.90cbin.1	99.02	0.791	105
Sample_SPB12.mb.87cbin.1	98.63	0.373	50
Sample_SPB12.Cluster1202cbin.1	98.17	1.240	44
Sample_SPB13.Cluster2582	83.10	0.862	52
Sample_SPB13.Cluster286	88.59	0.671	30
Sample_SPB13.Cluster219cbin.1	96.64	0.530	161
Sample_SPB13.mb.28	95.30	0.000	139
Sample_SPB13.Cluster413	96.02	1.020	46
Sample_SPB13.mb.13	96.63	0.000	23
Sample_SPB13.Cluster3974cbin.1	84.57	1.689	369
Sample_SPB13.mb.43cbin.1	91.94	0.000	46
Sample_SPB13.Cluster4375cbin.1	96.61	1.932	82
Sample_SPB13.Cluster6039	99.32	3.020	19
Sample_SPB13.Cluster3169	96.38	1.497	21
Sample_SPB13.mb.32	97.54	0.961	38
Sample_SPB13.Cluster17cbin.1	91.74	1.156	210
Sample_SPB13.Cluster418	99.54	0.984	25
Sample_SPB13.Cluster946	98.75	0.625	33
Sample_SPB13.mb.1	94.44	4.803	53
Sample_SPB13.Cluster8cbin.1	95.97	2.013	239
Sample_SPB13.mb.81	89.84	0.671	196
Sample_SPB13.mb.83	88.39	0.000	277
Sample_SPB13.Cluster3792cbin.1	93.02	2.363	90
Sample_SPB13.mb.69cbin.1	96.64	2.390	44
Sample_SPB13.mb.38cbin.1	89.87	4.372	149
Sample_SPB13.mb.66cbin.1	92.69	0.789	341
Sample_SPB13.mb.4cbin.1	100.00	0.632	75
Sample_SPB13.Cluster2682cbin.1	97.16	3.841	49
Sample_SPB13.Cluster2803cbin.1	93.77	0.000	90
Sample_SPB13.mb.59	99.62	0.000	16
Sample_SPB13.mb.67cbin.1	97.07	0.000	71

Sample_SPB13.Cluster6576cbin.1	94.68	4.926	229
Sample_SPB13.mb.74	95.86	1.442	31
Sample_SPB13.mb.31cbin.1	96.39	4.397	86
Sample_SPB13.mb.63	98.88	0.000	36
Sample_SPB15.Cluster11681	92.69	2.969	25
Sample_SPB15.mb.5cbin.1	92.41	2.247	41
Sample_SPB15.Cluster5cbin.1	92.40	4.020	163
Sample_SPB15.mb.18	83.64	1.744	249
Sample_SPB15.mb.44cbin.1	96.77	3.293	118
Sample_SPB15.Cluster10967	99.32	0.671	14
Sample_SPB15.Cluster34	80.19	0.671	162
Sample_SPB15.mb.67	84.64	0.671	222
Sample_SPB15.Cluster362cbin.1	84.56	4.026	34
Sample_SPB15.mb.65	89.82	0.061	191
Sample_SPB15.mb.22cbin.1	84.76	1.342	169
Sample_SPB15.mb.48	87.58	1.906	139
Sample_SPB15.mb.25	89.84	1.006	231
Sample_SPB15.mb.35	91.66	0.873	131
Sample_SPB15.Cluster4864cbin.1	95.30	2.013	24
Sample_SPB15.mb.33cbin.1	83.08	4.697	314
Sample_SPB15.mb.50cbin.1	81.43	2.684	30
Sample_SPB15.mb.64	87.24	1.342	50
Sample_SPB15.Cluster1660cbin.1	92.77	0.335	105
Sample_SPB15.mb.3	93.89	2.813	230
Sample_SPB15.mb.53	97.31	1.006	95
Sample_SPB15.mb.60cbin.1	88.25	4.317	42
Sample_SPB15.mb.58cbin.1	97.77	1.497	170
Sample_SPB15.mb.73cbin.1	95.30	1.342	85
Sample_SPB15.mb.84cbin.1	97.27	4.921	17
Sample_SPB15.mb.37	99.66	0.361	57
Sample_SPB16.mb.47	96.49	3.508	37
Sample_SPB16.mb.43	82.75	0.000	173
Sample_SPB16.mb.23cbin.1	81.64	4.029	382
Sample_SPB16.Cluster7479cbin.1	82.23	2.351	363
Sample_SPB16.mb.66	93.73	1.296	171
Sample_SPB16.mb.58	98.49	0.241	20
Sample_SPB16.Cluster975cbin.1	98.12	0.000	113
Sample_SPB16.mb.40cbin.1	81.26	1.342	275
Sample_SPB16.Cluster7012	98.65	1.677	15
Sample_SPB16.mb.30cbin.1	80.65	3.020	268
Sample_SPB16.mb.53	95.05	0.671	30
Sample_SPB16.Cluster273	99.55	0.000	26
Sample_SPB16.mb.74cbin.1	91.55	4.857	300
Sample_SPB16.Cluster8301	99.47	0.000	24
Sample_SPB16.mb.37cbin.1	86.79	2.899	305
Sample_SPB16.mb.19cbin.1	93.95	1.677	32
Sample_SPB16.Cluster7212cbin.1	97.38	2.531	152
Sample_SPB16.Cluster5972cbin.1	99.51	0.785	89
Sample_SPB16.mb.4cbin.1	92.16	2.670	264
Sample_SPB16.mb.5	99.03	1.787	99
Sample_SPB16.Cluster3018cbin.1	97.93	1.865	95
Sample_SPB16.Cluster6185cbin.1	86.39	1.752	487
Sample_SPB17.mb.23	89.23	0.322	91
Sample_SPB17.Cluster126cbin.1	99.54	0.772	40
Sample_SPB17.mb.15cbin.1	93.08	1.781	111
Sample_SPB17.Cluster17cbin.1	98.63	0.510	123
Sample_SPB17.mb.18	94.75	0.593	149
Sample_SPB17.mb.5cbin.1	98.42	0.000	50

Sample_SPB17.mb.39cbin.1	97.69	0.192	57
Sample_SPB17.Cluster866	97.40	0.384	36
Sample_SPB17.mb.46cbin.1	98.87	1.127	98
Sample_SPB20.mb.3	98.50	0.000	25
Sample_SPB20.mb.5	89.32	1.123	36
Sample_SPB20.mb.48	91.10	2.013	154
Sample_SPB20.mb.8	87.07	1.123	21
Sample_SPB20.Cluster4644cbin.1	94.90	1.140	168
Sample_SPB20.Cluster2764cbin.1	95.59	0.000	82
Sample_SPB20.mb.54cbin.1	92.82	0.843	183
Sample_SPB20.Cluster56cbin.1	90.21	1.125	214
Sample_SPB20.mb.90	84.11	2.852	390
Sample_SPB20.mb.22	99.03	1.469	36
Sample_SPB20.Cluster1848cbin.1	97.95	0.000	42
Sample_SPB20.Cluster9206	99.32	1.006	17
Sample_SPB20.mb.24cbin.1	89.43	3.301	240
Sample_SPB20.mb.93	84.03	0.000	59
Sample_SPB20.Cluster379	100.00	0.115	15
Sample_SPB20.mb.64	93.66	2.729	129
Sample_SPB20.mb.2	95.30	1.342	67
Sample_SPB20.mb.67	91.53	3.225	153
Sample_SPB20.mb.92	97.76	0.020	58
Sample_SPB20.mb.107	94.91	0.671	125
Sample_SPB20.mb.46cbin.1	83.46	0.671	370
Sample_SPB20.mb.77	89.19	1.814	171
Sample_SPB20.mb.53	84.80	1.190	134
Sample_SPB20.Cluster7098cbin.1	83.57	2.596	103
Sample_SPB20.mb.94cbin.1	97.98	0.167	41
Sample_SPB20.mb.33	93.15	1.854	85
Sample_SPB20.mb.87	93.02	0.000	100
Sample_SPB20.mb.62cbin.1	87.13	1.388	242
Sample_SPB20.mb.45cbin.1	99.21	4.477	60
Sample_SPB20.mb.44	95.44	0.388	25
Sample_SPB20.mb.36	97.38	0.961	36
Sample_SPB20.mb.63	91.52	0.894	103
Sample_SPB20.mb.16	89.61	2.549	95
Sample_SPB20.mb.72	83.91	2.046	89
Sample_SPB20.mb.60	85.67	1.115	84
Sample_SPB20.Cluster4561cbin.1	96.11	0.000	82
Sample_SPB20.mb.95	83.16	0.000	145
Sample_SPB20.Cluster566	98.80	0.682	34
Sample_SPB21.Cluster4534	97.84	1.173	42
Sample_SPB21.mb.107cbin.1	93.03	0.111	175
Sample_SPB21.Cluster14434	88.88	0.284	33
Sample_SPB21.mb.14	92.93	1.342	22
Sample_SPB21.mb.1cbin.1	97.20	1.864	91
Sample_SPB21.Cluster7341cbin.1	89.59	0.000	61
Sample_SPB21.Cluster9839cbin.1	97.76	2.013	100
Sample_SPB21.mb.22	99.32	2.013	80
Sample_SPB21.mb.43	85.61	0.671	241
Sample_SPB21.mb.103cbin.1	98.32	0.000	51
Sample_SPB21.mb.95	94.82	4.780	58
Sample_SPB21.mb.29cbin.1	95.04	3.164	166
Sample_SPB21.mb.48	98.65	1.740	32
Sample_SPB21.mb.38	89.60	2.190	205
Sample_SPB21.mb.101	97.59	0.961	29
Sample_SPB21.mb.45cbin.1	92.79	2.069	163
Sample_SPB21.Cluster10842cbin.1	99.32	1.006	33

Sample_SPB21.mb.55	91.27	2.572	58
Sample_SPB21.Cluster10257cbin.1	96.49	0.000	95
Sample_SPB21.Cluster563cbin.1	100.00	0.000	41
Sample_SPB21.mb.64	92.78	2.852	230
Sample_SPB21.mb.85	98.65	0.000	20
Sample_SPB21.mb.42	93.96	1.696	174
Sample_SPB21.mb.20	96.97	0.671	57
Sample_SPB21.mb.52	96.59	0.680	218
Sample_SPB21.Cluster9992	93.55	2.608	92
Sample_SPB21.mb.44	95.30	0.000	23
Sample_SPB21.Cluster8593cbin.1	98.49	0.000	30
Sample_SPB21.mb.33	92.74	0.806	126
Sample_SPB21.mb.96	90.82	0.671	23
Sample_SPB21.mb.99	91.83	1.053	62
Sample_SPB21.mb.57cbin.1	95.97	4.175	81
Sample_SPB21.Cluster8466	99.32	0.000	222
Sample_SPB21.mb.78cbin.1	100.00	0.480	53
Sample_SPB21.mb.40cbin.1	89.28	2.487	68
Sample_SPB21.mb.86	97.31	0.000	23
Sample_SPB21.Cluster6847cbin.1	90.70	4.584	59
Sample_SPB21.Cluster6428	96.94	0.384	137
Sample_SPB21.mb.70	92.18	1.679	125
Sample_SPB21.mb.83cbin.1	98.51	0.000	65
Sample_SPB22.Cluster12841cbin.1	93.82	1.123	13
Sample_SPB22.Cluster12951	98.66	0.111	10
Sample_SPB22.mb.103	93.82	2.407	92
Sample_SPB22.Cluster12921cbin.1	80.16	2.447	30
Sample_SPB22.Cluster5512cbin.1	88.23	1.342	28
Sample_SPB22.Cluster7098cbin.1	85.07	2.516	192
Sample_SPB22.Cluster9389	98.87	1.123	34
Sample_SPB22.mb.2cbin.1	91.37	4.231	66
Sample_SPB22.mb.38	97.58	0.000	58
Sample_SPB22.mb.11	92.05	0.000	76
Sample_SPB22.Cluster3071	87.50	2.403	33
Sample_SPB22.Cluster4341cbin.1	85.19	2.013	37
Sample_SPB22.mb.53	88.47	0.671	43
Sample_SPB22.mb.88	92.13	0.561	19
Sample_SPB22.Cluster10662cbin.1	98.65	1.006	27
Sample_SPB22.mb.15cbin.1	87.98	4.560	702
Sample_SPB22.mb.30cbin.1	92.58	2.013	269
Sample_SPB22.Cluster7923	89.82	0.000	24
Sample_SPB22.Cluster542	98.66	0.000	49
Sample_SPB22.mb.86	96.42	0.000	54
Sample_SPB22.mb.28	97.10	4.927	61
Sample_SPB22.mb.34	87.27	3.467	302
Sample_SPB22.mb.47	94.29	0.671	198
Sample_SPB22.mb.83	98.60	1.165	89
Sample_SPB22.Cluster1106cbin.1	98.75	2.484	329
Sample_SPB22.mb.74	88.25	0.000	37
Sample_SPB22.Cluster888	97.95	0.680	29
Sample_SPB22.mb.24	95.07	0.000	31
Sample_SPB22.mb.100	93.28	0.671	42
Sample_SPB22.mb.57	90.62	4.807	56
Sample_SPB22.mb.92	94.54	0.944	19
Sample_SPB22.Cluster11789cbin.1	92.01	3.315	573
Sample_SPB22.mb.42	85.32	0.591	544
Sample_SPB22.mb.80	99.03	0.480	20
Sample_SPB22.Cluster5885	94.65	0.192	118

Sample_SPB22.mb.72	95.90	0.584	49
Sample_SPB22.mb.55	99.24	0.000	41
Sample_SPB22.mb.36	85.70	0.929	81
Sample_SPB22.Cluster5410cbin.1	96.76	0.384	219
Sample_SPB22.mb.73	91.45	0.469	125
Sample_SPB25.Cluster14413	92.69	2.407	59
Sample_SPB25.Cluster6862	92.40	0.699	8
Sample_SPB25.Cluster2209cbin.1	91.93	4.327	209
Sample_SPB25.Cluster557	97.58	0.806	21
Sample_SPB25.mb.14	91.78	0.335	221
Sample_SPB25.Cluster13262cbin.1	99.05	0.000	114
Sample_SPB25.mb.51	84.34	0.806	350
Sample_SPB25.mb.12	98.38	0.000	13
Sample_SPB25.mb.20cbin.1	89.59	1.342	84
Sample_SPB25.Cluster6772	97.31	0.671	98
Sample_SPB25.mb.52	95.52	1.165	103
Sample_SPB25.mb.26	99.32	0.671	39
Sample_SPB25.mb.18cbin.1	82.63	1.972	387
Sample_SPB25.Cluster13419cbin.1	95.84	2.112	258
Sample_SPB25.mb.32	100.00	1.006	92
Sample_SPB25.mb.48cbin.1	97.31	0.671	61
Sample_SPB25.Cluster11630cbin.1	99.61	0.000	29
Sample_SPB25.mb.38	100.00	0.000	30
Sample_SPB25.mb.67	91.99	2.913	214
Sample_SPB25.mb.24cbin.1	82.22	4.219	319
Sample_SPB25.Cluster677	94.50	0.977	48
Sample_SPB25.Cluster228cbin.1	93.76	2.777	50
Sample_SPB25.mb.65	88.14	0.335	253
Sample_SPB25.mb.74	80.44	2.066	118
Sample_SPB25.mb.91cbin.1	93.95	3.041	164
Sample_SPB25.mb.31	99.11	0.499	55
Sample_SPB25.mb.92	96.09	0.236	66
Sample_SPB25.Cluster6849cbin.1	95.98	0.867	63
Sample_SPB25.mb.83cbin.1	97.76	0.000	43
Sample_SPB25.mb.82cbin.1	93.45	0.971	141
Sample_SPB25.mb.81	95.32	1.478	95
Sample_SPB25.mb.77	98.12	1.466	82
Sample_SPB25.Cluster7636cbin.1	98.72	1.761	114
Sample_SPB25.Cluster6850cbin.1	98.97	3.390	135
Sample_SPB25.mb.68	100.00	1.148	34
Sample_SPB25.mb.62cbin.1	94.64	4.603	355
Sample_SPB25.mb.95cbin.1	98.37	1.108	162
Sample_SPB26.Cluster13102	92.41	1.123	16
Sample_SPB26.Cluster128cbin.1	93.95	0.692	42
Sample_SPB26.Cluster1230cbin.1	98.79	0.806	18
Sample_SPB26.Cluster1477cbin.1	86.68	0.000	43
Sample_SPB26.mb.27cbin.1	89.16	2.121	149
Sample_SPB26.Cluster12694	98.40	0.800	92
Sample_SPB26.Cluster7424	94.59	1.351	27
Sample_SPB26.mb.30	98.38	2.419	40
Sample_SPB26.mb.117	91.65	1.342	38
Sample_SPB26.Cluster11079	99.32	1.006	15
Sample_SPB26.Cluster4030	97.04	0.671	26
Sample_SPB26.Cluster787	99.06	0.545	37
Sample_SPB26.Cluster948cbin.1	98.65	1.612	36
Sample_SPB26.Cluster503cbin.1	99.55	4.277	30
Sample_SPB26.Cluster1166cbin.1	97.95	1.360	35
Sample_SPB26.mb.103	87.25	0.000	35

Sample_SPB26.mb.65	95.80	1.165	94
Sample_SPB26.mb.37	93.91	2.702	97
Sample_SPB26.Cluster11740cbin.1	99.02	0.000	73
Sample_SPB26.mb.73cbin.1	89.18	0.000	237
Sample_SPB26.Cluster96	99.11	0.591	59
Sample_SPB26.mb.12	97.27	1.708	24
Sample_SPB26.Cluster10643cbin.1	90.00	0.000	24
Sample_SPB26.mb.112	83.27	0.377	119
Sample_SPB26.mb.16	95.28	0.943	73
Sample_SPB26.mb.7	98.15	0.806	83
Sample_SPB26.Cluster7918cbin.1	98.92	0.537	103
Sample_SPB26.mb.88	87.74	3.378	568
Sample_SPB26.mb.34cbin.1	99.51	0.000	52
Sample_SPB26.mb.33	86.74	1.753	168
Sample_SPB26.mb.92	89.26	4.086	56
Sample_SPB26.mb.23	88.51	4.601	88
Sample_SPB26.mb.77	94.35	0.909	70
Sample_SPB26.Cluster9006cbin.1	95.52	1.165	262
Sample_SPB26.mb.91	98.65	0.000	63
Sample_SPB26.mb.69	85.60	1.570	215
Sample_SPB26.Cluster1013cbin.1	97.07	2.440	212
Sample_SPB27.Cluster2043cbin.1	93.93	1.342	24
Sample_SPB27.Cluster3466cbin.1	83.80	0.061	270
Sample_SPB27.mb.25cbin.1	99.30	1.165	70
Sample_SPB27.mb.24cbin.1	97.98	1.342	105
Sample_SPB27.mb.40cbin.1	81.45	1.426	350
Sample_SPB27.mb.27cbin.1	91.27	2.013	38
Sample_SPB27.Cluster2426cbin.1	99.18	1.086	86
Sample_SPB27.mb.19cbin.1	96.64	0.671	116
Sample_SPB27.Cluster1597cbin.1	91.77	0.021	60
Sample_SPB27.Cluster210cbin.1	93.65	2.312	219
Sample_SPB27.Cluster57cbin.1	92.51	0.000	73
Sample_SPB27.Cluster6184	95.70	0.335	38
Sample_SPB27.mb.21	94.71	0.961	33
Sample_SPB27.Cluster1327	97.04	0.000	20
Sample_SPB27.mb.42	84.98	4.761	69
Sample_SPB27.mb.22	83.22	0.000	22
Sample_SPB27.mb.45	86.68	1.740	271
Sample_SPB27.mb.26cbin.1	97.69	0.268	25
Sample_SPB27.Cluster5753cbin.1	97.58	3.131	45
Sample_SPB27.mb.56	91.26	1.995	394
Sample_SPB27.mb.77cbin.1	89.83	0.000	285
Sample_SPB27.mb.34cbin.1	87.97	0.000	70
Sample_SPB27.mb.66cbin.1	97.66	2.807	44
Sample_SPB27.mb.70	92.77	0.675	227
Sample_SPB27.Cluster2757cbin.1	83.45	1.164	593
Sample_SPB27.mb.52cbin.1	95.41	1.690	89
Sample_SPB27.mb.29	98.42	0.000	33
Sample_SPB27.mb.38	93.17	0.000	49
Sample_SPB27.mb.88	92.75	0.000	32
Sample_SPB27.mb.63	98.83	0.480	30
Sample_SPB27.Cluster2220	99.34	1.582	48
Sample_SPB27.mb.10	96.31	0.000	36
Sample_SPB27.Cluster4319cbin.1	99.61	1.666	87
Sample_SPB27.mb.48cbin.1	97.54	0.929	80
Sample_SPB27.mb.54	95.77	1.550	90
Sample_SPB28.mb.43	91.61	1.677	277
Sample_SPB28.Cluster2207cbin.1	85.78	1.317	324

Sample_SPB28.Cluster212	99.19	0.806	40
Sample_SPB28.mb.30cbin.1	90.82	3.380	227
Sample_SPB28.Cluster6615cbin.1	93.33	3.645	295
Sample_SPB28.Cluster7542	99.32	0.671	30
Sample_SPB28.Cluster6082	98.65	0.000	20
Sample_SPB28.mb.19	94.20	2.403	67
Sample_SPB28.Cluster9014cbin.1	98.65	1.342	47
Sample_SPB28.Cluster5597cbin.1	94.38	2.076	211
Sample_SPB28.mb.47	97.61	0.680	222
Sample_SPB28.mb.70cbin.1	94.69	0.335	97
Sample_SPB28.mb.57	94.89	2.551	92
Sample_SPB28.mb.78cbin.1	97.30	0.842	89
Sample_SPB28.mb.35	88.49	2.215	108
Sample_SPB28.Cluster4850cbin.1	92.02	1.177	369
Sample_SPB28.mb.56	97.31	0.838	127
Sample_SPB28.mb.13	99.31	0.738	115
Sample_SPB28.mb.51cbin.1	83.98	0.632	63
Sample_SPB28.mb.71cbin.1	98.53	4.412	55
Sample_SPB28.mb.89cbin.1	96.53	2.878	117
Sample_SPB28.mb.65	98.63	0.265	67
Sample_SPB28.Cluster4528cbin.1	96.47	3.076	354
Sample_SPB28.mb.32cbin.1	89.69	0.555	481
Sample_SPB28.Cluster35	95.63	0.359	93
Sample_SPB30.Cluster3079cbin.1	82.36	1.724	543
Sample_SPB30.Cluster4134	92.93	1.342	20
Sample_SPB30.Cluster814cbin.1	99.30	1.165	77
Sample_SPB30.Cluster9210	94.96	0.000	35
Sample_SPB30.Cluster3409cbin.1	89.24	0.021	60
Sample_SPB30.Cluster2181	93.42	0.961	23
Sample_SPB30.mb.19	83.87	0.000	27
Sample_SPB30.mb.68	82.63	0.559	389
Sample_SPB30.Cluster3620	96.60	0.335	12
Sample_SPB30.mb.27cbin.1	92.61	1.972	120
Sample_SPB30.Cluster1481cbin.1	92.45	3.746	151
Sample_SPB30.Cluster10678	96.54	0.000	71
Sample_SPB30.Cluster422cbin.1	99.11	0.000	24
Sample_SPB30.mb.22	95.91	0.000	15
Sample_SPB30.mb.35	93.04	1.901	252
Sample_SPB30.mb.34cbin.1	93.42	2.040	248
Sample_SPB30.mb.47cbin.1	98.65	0.943	76
Sample_SPB30.mb.70	83.19	2.908	189
Sample_SPB30.mb.5cbin.1	95.97	0.000	143
Sample_SPB30.mb.43	93.31	1.133	250
Sample_SPB30.mb.29	97.95	0.000	19
Sample_SPB30.mb.80	93.73	0.279	145
Sample_SPB30.mb.31	95.18	1.463	175
Sample_SPB30.mb.26	96.68	0.000	50
Sample_SPB30.mb.57cbin.1	87.98	0.480	31
Sample_SPB30.mb.58cbin.1	98.02	1.875	24
Sample_SPB30.mb.41cbin.1	95.89	1.534	131
Sample_SPB30.Cluster8968	95.69	0.842	138
Sample_SPB30.Cluster4912cbin.1	99.46	0.806	116
Sample_SPB30.mb.74	90.86	0.000	13
Sample_SPB30.mb.46cbin.1	94.27	0.480	21
Sample_SPB30.mb.81cbin.1	94.18	0.000	37
Sample_SPB30.mb.92	92.95	2.745	95
Sample_SPB30.mb.72	100.00	0.636	33
Sample_SPB30.mb.93	85.75	2.531	387

Sample_SPB30.mb.9	99.32	0.000	23
Sample_SPB30.mb.94cbin.1	92.28	0.671	175
Sample_SPB30.mb.53	91.53	0.093	39
Sample_SPB30.mb.10cbin.1	92.91	2.695	275
Sample_SPB31.Cluster13260	89.32	2.969	64
Sample_SPB31.Cluster3560	92.93	1.342	29
Sample_SPB31.mb.50cbin.1	83.12	4.310	155
Sample_SPB31.Cluster2308cbin.1	87.13	0.061	250
Sample_SPB31.mb.21	92.17	0.671	20
Sample_SPB31.Cluster89	91.27	0.671	56
Sample_SPB31.mb.4	95.39	0.000	42
Sample_SPB31.Cluster4714	98.20	0.898	36
Sample_SPB31.mb.19	85.01	1.250	75
Sample_SPB31.mb.46	97.65	0.335	89
Sample_SPB31.mb.72cbin.1	96.45	1.221	167
Sample_SPB31.mb.1	86.57	0.335	17
Sample_SPB31.mb.76	93.14	0.000	44
Sample_SPB31.Cluster10043cbin.1	88.31	1.719	345
Sample_SPB31.mb.15	96.29	1.739	129
Sample_SPB31.Cluster6381	97.09	1.342	22
Sample_SPB31.Cluster13048	99.47	0.000	33
Sample_SPB31.Cluster291	99.11	0.000	35
Sample_SPB31.mb.63	87.15	1.275	265
Sample_SPB31.mb.40	98.02	1.230	93
Sample_SPB31.mb.27cbin.1	92.81	3.752	234
Sample_SPB31.mb.86	89.82	0.581	68
Sample_SPB31.mb.64	95.86	1.403	211
Sample_SPB31.mb.25	88.33	1.783	105
Sample_SPB31.Cluster8738	98.06	0.483	78
Sample_SPB31.mb.107cbin.1	86.25	0.186	112
Sample_SPB31.mb.42	96.57	0.000	89
Sample_SPB31.mb.90	88.01	0.185	216
Sample_SPB31.mb.85cbin.1	93.85	1.845	226
Sample_SPB31.mb.93	89.70	1.260	135
Sample_SPB31.mb.23	99.19	4.042	93
Sample_SPB31.Cluster5530	97.19	0.384	61
Sample_SPB31.mb.78	99.24	0.000	20
Sample_SPB31.mb.41	97.91	1.253	193
Sample_SPB31.Cluster35cbin.1	97.51	0.553	84
Sample_SPB32.mb.100	93.54	0.000	43
Sample_SPB32.Cluster6265cbin.1	99.32	1.342	12
Sample_SPB32.mb.13cbin.1	85.90	0.671	27
Sample_SPB32.mb.11	93.73	0.671	65
Sample_SPB32.Cluster3095cbin.1	93.14	2.531	224
Sample_SPB32.Cluster2891cbin.1	98.42	0.289	24
Sample_SPB32.Cluster4348cbin.1	99.32	0.000	18
Sample_SPB32.Cluster11cbin.1	88.65	2.210	97
Sample_SPB32.Cluster142	93.75	0.000	137
Sample_SPB32.mb.95cbin.1	81.06	1.898	194
Sample_SPB32.Cluster110	99.55	0.000	25
Sample_SPB32.mb.18	94.58	1.932	77
Sample_SPB32.Cluster161cbin.1	99.03	0.000	23
Sample_SPB32.mb.53cbin.1	85.92	4.648	187
Sample_SPB32.Cluster2789	91.45	0.632	82
Sample_SPB32.Cluster5551cbin.1	94.33	0.000	77
Sample_SPB32.mb.47	95.56	0.632	158
Sample_SPB32.Cluster4022cbin.1	82.36	3.308	306
Sample_SPB32.mb.5	85.47	0.675	68

Sample_SPB32.mb.104cbin.1	85.33	1.008	145
Sample_SPB32.Cluster3715	94.63	0.000	38
Sample_SPB32.mb.39	87.52	1.169	203
Sample_SPB32.mb.93cbin.1	95.59	0.961	24
Sample_SPB32.mb.29	96.77	0.000	70
Sample_SPB32.mb.57cbin.1	92.40	1.265	50
Sample_SPB32.mb.70cbin.1	97.45	0.636	35
Sample_SPB32.mb.64	92.05	2.214	48
Sample_SPB32.Cluster3691cbin.1	92.67	4.719	323
Sample_SPB32.Cluster1224cbin.1	97.30	1.282	227
Sample_SPB32.mb.86cbin.1	95.73	2.219	122
Sample_SPB32.mb.96cbin.1	99.51	2.161	117
Sample_SPB32.Cluster1850cbin.1	94.00	1.115	43
Sample_SPB32.mb.107cbin.1	98.46	0.576	38
Sample_SPB32.mb.78cbin.1	82.75	1.320	298
Sample_SPB34.Cluster8235	82.45	0.000	44
Sample_SPB34.Cluster2119cbin.1	90.78	0.000	68
Sample_SPB34.Cluster219cbin.1	94.18	3.355	286
Sample_SPB34.Cluster8435	81.87	0.000	21
Sample_SPB34.Cluster11cbin.1	81.63	3.451	337
Sample_SPB34.Cluster1214cbin.1	97.31	0.711	58
Sample_SPB34.Cluster5423cbin.1	90.15	0.335	187
Sample_SPB34.mb.10	95.19	1.442	62
Sample_SPB34.mb.63	93.95	1.454	131
Sample_SPB34.mb.103cbin.1	87.87	0.000	46
Sample_SPB34.Cluster1265cbin.1	98.75	1.250	60
Sample_SPB34.Cluster6727cbin.1	98.65	2.684	43
Sample_SPB34.mb.43	90.45	0.806	72
Sample_SPB34.Cluster9258	84.36	0.061	289
Sample_SPB34.Cluster6494cbin.1	97.82	0.000	86
Sample_SPB34.mb.58cbin.1	97.31	3.020	90
Sample_SPB34.Cluster6796cbin.1	97.10	0.724	150
Sample_SPB34.mb.71	98.65	1.342	50
Sample_SPB34.Cluster2430cbin.1	97.66	3.735	406
Sample_SPB34.mb.105	93.56	4.554	322
Sample_SPB34.Cluster11702	98.87	1.123	54
Sample_SPB34.mb.107	80.59	0.621	97
Sample_SPB34.mb.8cbin.1	84.10	1.677	331
Sample_SPB34.mb.90	87.24	0.000	99
Sample_SPB34.Cluster9277cbin.1	98.38	0.000	92
Sample_SPB34.mb.96cbin.1	95.57	0.743	73
Sample_SPB34.mb.52	97.58	1.132	158
Sample_SPB34.mb.80	95.75	3.605	144
Sample_SPB34.mb.60	96.21	1.207	93
Sample_SPB34.mb.6	92.46	4.388	253
Sample_SPB34.mb.2cbin.1	91.26	1.198	418
Sample_SPB34.mb.78cbin.1	95.51	2.826	98
Sample_SPB34.mb.91	98.05	1.169	131
Sample_SPB34.mb.72cbin.1	93.84	3.076	144
Sample_SPB35.Cluster13733	84.99	3.370	38
Sample_SPB35.Cluster13575	92.41	2.247	23
Sample_SPB35.Cluster2519	95.69	0.000	139
Sample_SPB35.Cluster10159	95.97	2.684	24
Sample_SPB35.mb.29	84.26	3.745	72
Sample_SPB35.Cluster9089	98.65	0.000	30
Sample_SPB35.mb.118cbin.1	91.70	1.398	10
Sample_SPB35.mb.24cbin.1	93.88	3.561	179
Sample_SPB35.mb.112	83.89	0.000	17

Sample_SPB35.Cluster5949cbin.1	87.69	2.013	55
Sample_SPB35.Cluster902cbin.1	87.69	2.908	241
Sample_SPB35.mb.107	86.07	1.342	51
Sample_SPB35.mb.47	93.82	1.123	45
Sample_SPB35.Cluster47cbin.1	95.16	1.008	51
Sample_SPB35.Cluster6306	94.10	2.294	66
Sample_SPB35.Cluster932cbin.1	82.87	3.216	830
Sample_SPB35.mb.114cbin.1	96.42	3.355	84
Sample_SPB35.Cluster11811cbin.1	92.60	0.000	216
Sample_SPB35.Cluster134	97.27	0.000	49
Sample_SPB35.Cluster2001	98.75	0.625	43
Sample_SPB35.Cluster9269	97.09	0.671	24
Sample_SPB35.mb.85cbin.1	91.57	1.123	19
Sample_SPB35.Cluster495cbin.1	99.54	0.075	27
Sample_SPB35.mb.106	88.70	4.308	276
Sample_SPB35.mb.17cbin.1	92.95	4.423	98
Sample_SPB35.Cluster9599	96.64	0.000	48
Sample_SPB35.mb.110	97.40	1.594	49
Sample_SPB35.mb.100	100.00	0.000	16
Sample_SPB35.mb.54cbin.1	92.33	1.454	262
Sample_SPB35.mb.30	97.65	1.342	111
Sample_SPB35.mb.83	82.36	0.000	242
Sample_SPB35.mb.25cbin.1	96.09	0.335	122
Sample_SPB35.mb.8cbin.1	90.55	2.097	27
Sample_SPB35.mb.76cbin.1	97.76	0.335	29
Sample_SPB35.mb.45cbin.1	81.15	1.342	61
Sample_SPB35.mb.95	83.83	0.713	33
Sample_SPB35.mb.57cbin.1	81.87	2.013	80
Sample_SPB35.mb.49cbin.1	97.09	0.671	132
Sample_SPB35.mb.67cbin.1	91.27	1.006	57
Sample_SPB35.mb.41cbin.1	97.02	3.416	241
Sample_SPB35.mb.82cbin.1	92.91	1.230	112
Sample_SPB35.mb.66cbin.1	93.44	0.000	293
Sample_SPB35.Cluster428	98.80	0.074	29
Sample_SPB36.Cluster48cbin.1	85.36	1.342	302
Sample_SPB36.mb.102	82.41	0.000	171
Sample_SPB36.mb.51	91.57	0.561	21
Sample_SPB36.mb.39	89.20	0.335	210
Sample_SPB36.mb.4	88.88	0.854	23
Sample_SPB36.mb.57cbin.1	95.80	1.165	132
Sample_SPB36.mb.74cbin.1	95.96	0.806	45
Sample_SPB36.Cluster4543cbin.1	95.88	1.265	191
Sample_SPB36.Cluster3595	92.78	0.480	20
Sample_SPB36.Cluster4053cbin.1	95.97	2.013	55
Sample_SPB36.mb.50	86.91	1.342	47
Sample_SPB36.Cluster3243cbin.1	90.47	0.680	25
Sample_SPB36.Cluster136cbin.1	93.87	0.157	56
Sample_SPB36.mb.58	88.22	3.299	119
Sample_SPB36.mb.15	97.27	1.342	25
Sample_SPB36.Cluster977	99.55	0.000	27
Sample_SPB36.mb.76	88.82	1.342	111
Sample_SPB36.mb.23cbin.1	96.39	3.189	45
Sample_SPB36.mb.85	98.22	0.838	131
Sample_SPB36.mb.37	97.31	0.000	119
Sample_SPB36.mb.38	98.06	0.060	44
Sample_SPB36.mb.67	90.96	3.265	158
Sample_SPB36.mb.9cbin.1	98.65	0.335	38
Sample_SPB36.mb.106	92.93	1.050	38

Sample_SPB36.mb.5	88.05	4.062	99
Sample_SPB36.mb.63cbin.1	93.60	3.404	174
Sample_SPB36.Cluster9234cbin.1	99.31	0.646	73
Sample_SPB37.Cluster5612cbin.1	81.09	0.000	99
Sample_SPB37.mb.131	81.55	0.000	36
Sample_SPB37.Cluster444cbin.1	92.61	2.013	31
Sample_SPB37.mb.18	94.40	0.000	39
Sample_SPB37.mb.10	86.57	0.671	70
Sample_SPB37.mb.109	94.63	0.083	25
Sample_SPB37.Cluster11531	97.30	0.707	97
Sample_SPB37.Cluster219cbin.1	91.39	1.478	63
Sample_SPB37.mb.107	89.26	0.793	54
Sample_SPB37.Cluster3269cbin.1	93.75	0.961	44
Sample_SPB37.Cluster10324	97.98	0.671	17
Sample_SPB37.mb.103cbin.1	96.42	2.684	177
Sample_SPB37.mb.32	94.87	1.515	266
Sample_SPB37.Cluster4525	96.93	0.000	13
Sample_SPB37.Cluster285cbin.1	87.11	0.296	37
Sample_SPB37.Cluster57cbin.1	97.44	1.183	142
Sample_SPB37.Cluster7811	88.59	0.671	13
Sample_SPB37.mb.141cbin.1	99.18	0.000	34
Sample_SPB37.mb.142	94.44	0.000	204
Sample_SPB37.Cluster1449cbin.1	90.41	0.454	72
Sample_SPB37.Cluster11556cbin.1	99.52	0.472	27
Sample_SPB37.Cluster399cbin.1	97.17	0.998	100
Sample_SPB37.mb.127	90.61	1.360	241
Sample_SPB37.mb.105	94.46	0.223	88
Sample_SPB37.mb.112cbin.1	99.47	0.000	35
Sample_SPB37.mb.29cbin.1	92.40	0.632	62
Sample_SPB37.mb.102	89.46	1.169	206
Sample_SPB37.mb.30cbin.1	96.13	0.757	53
Sample_SPB37.mb.17cbin.1	92.25	1.869	208
Sample_SPB37.mb.63	83.22	0.000	36
Sample_SPB37.mb.44cbin.1	84.17	0.020	176
Sample_SPB37.mb.13cbin.1	90.43	0.000	25
Sample_SPB37.mb.76cbin.1	95.40	1.265	168
Sample_SPB37.mb.40cbin.1	91.28	0.241	258
Sample_SPB37.Cluster6256cbin.1	98.73	3.069	204
Sample_SPB37.mb.82cbin.1	94.59	0.675	69
Sample_SPB37.mb.73cbin.1	88.43	0.680	134
Sample_SPB37.mb.39	95.65	1.242	158
Sample_SPB37.mb.52	93.78	0.961	30
Sample_SPB37.mb.90cbin.1	80.78	1.913	50
Sample_SPB37.mb.8cbin.1	98.13	0.000	30
Sample_SPB37.mb.69cbin.1	96.42	4.033	123
Sample_SPB37.mb.75cbin.1	90.93	2.460	280
Sample_SPB37.Cluster6931cbin.1	96.59	0.128	107
Sample_SPB37.mb.62cbin.1	96.80	0.598	121
Sample_SPB37.mb.42	96.24	1.959	135
Sample_SPB37.mb.4cbin.1	99.61	0.000	50
Sample_SPB38.Cluster33cbin.1	90.93	4.942	174
Sample_SPB38.mb.29cbin.1	89.36	2.147	194
Sample_SPB38.Cluster9738cbin.1	93.53	1.785	113
Sample_SPB38.Cluster404	99.19	1.612	30
Sample_SPB38.mb.32cbin.1	91.54	0.671	254
Sample_SPB38.mb.4	95.56	2.531	249
Sample_SPB38.Cluster1235cbin.1	95.18	0.000	66
Sample_SPB38.Cluster8110	99.32	1.677	13

Sample_SPB38.Cluster6151cbin.1	91.82	2.534	247
Sample_SPB38.Cluster2495	100.00	0.480	23
Sample_SPB38.mb.5	89.39	2.531	237
Sample_SPB38.mb.26cbin.1	97.98	0.335	29
Sample_SPB38.mb.38cbin.1	90.15	1.808	298
Sample_SPB38.Cluster51cbin.1	94.36	0.345	518
Sample_SPB38.mb.78	93.02	1.906	227
Sample_SPB38.Cluster7246cbin.1	99.11	0.724	62
Sample_SPB38.mb.81	99.19	0.195	20
Sample_SPB38.mb.67cbin.1	92.54	0.769	91
Sample_SPB38.Cluster5633	93.42	0.373	63
Sample_SPB38.mb.60	93.28	0.000	51
Sample_SPB38.mb.83	82.21	0.000	122
Sample_SPB38.Cluster6964	99.24	2.019	21
Sample_SPB38.mb.10cbin.1	93.78	1.282	50
Sample_SPB38.mb.41cbin.1	97.95	0.000	58
Sample_SPB38.mb.7	97.98	0.000	35
Sample_SPB38.mb.75	99.54	0.961	59
Sample_SPB38.mb.33cbin.1	90.37	4.042	334
Sample_SPB38.mb.51	98.41	1.265	139
Sample_SPB38.mb.34	96.49	0.576	275
Sample_SPB38.mb.93cbin.1	96.18	2.649	133
Sample_SPB38.mb.92cbin.1	96.45	2.969	184
Sample_SPB38.mb.79	95.91	1.132	174
Sample_SPB38.mb.91cbin.1	98.07	1.282	71
Sample_SPB39.Cluster13847cbin.1	92.02	2.808	25
Sample_SPB39.mb.20cbin.1	88.20	1.845	28
Sample_SPB39.Cluster7601	92.17	0.671	17
Sample_SPB39.mb.27	94.73	1.398	149
Sample_SPB39.Cluster12504	84.43	0.707	223
Sample_SPB39.mb.15cbin.1	92.72	0.223	213
Sample_SPB39.mb.102	95.91	0.377	97
Sample_SPB39.mb.64	81.43	0.915	179
Sample_SPB39.Cluster2146cbin.1	94.65	1.789	140
Sample_SPB39.mb.73	99.32	1.342	46
Sample_SPB39.Cluster119	96.27	0.621	62
Sample_SPB39.mb.72	93.52	0.671	232
Sample_SPB39.Cluster8360	99.32	0.000	17
Sample_SPB39.Cluster2005cbin.1	96.98	0.880	34
Sample_SPB39.Cluster7507cbin.1	98.65	0.000	22
Sample_SPB39.Cluster7097cbin.1	87.83	2.927	320
Sample_SPB39.mb.50	96.27	0.000	14
Sample_SPB39.mb.19	88.49	4.852	429
Sample_SPB39.mb.100	82.15	3.044	362
Sample_SPB39.mb.69	97.58	1.612	150
Sample_SPB39.Cluster5922cbin.1	93.22	1.207	55
Sample_SPB39.mb.65	96.15	1.939	40
Sample_SPB39.mb.75cbin.1	87.30	2.879	277
Sample_SPB39.mb.71cbin.1	98.65	0.671	76
Sample_SPB39.mb.70	94.31	2.419	126
Sample_SPB39.mb.94cbin.1	94.51	2.426	189
Sample_SPB39.Cluster5967cbin.1	94.19	1.075	368
Sample_SPB39.Cluster3079cbin.1	87.68	3.205	538
Sample_SPB39.mb.4cbin.1	98.06	0.966	84
Sample_SPB39.mb.18cbin.1	91.65	1.672	142
Sample_SPB39.mb.9cbin.1	90.66	0.021	76
Sample_SPB39.mb.6	97.73	0.377	63
Sample_SPB39.mb.87	98.06	1.207	42

Sample_SPB39.Cluster9689cbin.1	94.15	1.474	534
Sample_SPB39.mb.104cbin.1	93.92	3.007	254
Sample_SPB39.mb.97	97.98	0.000	40
Sample_SPB39.mb.78	88.84	0.000	49
Sample_SPB41.Cluster6743	92.40	0.699	6
Sample_SPB41.Cluster10038cbin.1	98.65	0.692	25
Sample_SPB41.mb.20	98.38	1.075	47
Sample_SPB41.Cluster1256	83.38	0.000	33
Sample_SPB41.Cluster220cbin.1	89.90	4.865	211
Sample_SPB41.Cluster3956cbin.1	92.69	1.349	84
Sample_SPB41.mb.16	81.97	4.138	419
Sample_SPB41.mb.100cbin.1	82.55	1.677	98
Sample_SPB41.mb.12	89.26	0.671	42
Sample_SPB41.Cluster11656	100.00	0.000	45
Sample_SPB41.Cluster6965	99.18	0.000	20
Sample_SPB41.mb.71	92.41	2.247	26
Sample_SPB41.Cluster6251	99.40	0.898	32
Sample_SPB41.Cluster1709cbin.1	97.83	0.961	22
Sample_SPB41.Cluster1542	97.95	0.000	24
Sample_SPB41.mb.55	80.88	3.020	289
Sample_SPB41.mb.63	98.60	2.564	135
Sample_SPB41.Cluster500cbin.1	98.07	0.480	64
Sample_SPB41.mb.81	93.26	1.342	48
Sample_SPB41.Cluster706	100.00	2.592	20
Sample_SPB41.mb.27	96.25	2.366	202
Sample_SPB41.mb.19	91.24	1.901	276
Sample_SPB41.mb.84	90.10	0.020	56
Sample_SPB41.mb.30cbin.1	93.17	0.671	31
Sample_SPB41.Cluster335cbin.1	97.98	2.348	46
Sample_SPB41.mb.54	98.38	1.478	85
Sample_SPB41.mb.65cbin.1	86.35	0.671	46
Sample_SPB41.mb.25	98.63	0.000	76
Sample_SPB41.mb.62cbin.1	97.11	1.442	62
Sample_SPB41.Cluster6618cbin.1	98.38	0.000	220
Sample_SPB41.mb.7	99.45	0.070	24
Sample_SPB41.mb.88cbin.1	98.42	0.000	120
Sample_SPB41.mb.67cbin.1	99.21	0.199	121
Sample_SPB41.Cluster7206	97.99	3.097	48
Sample_SPB41.mb.51cbin.1	91.89	2.005	206
Sample_SPB41.mb.96	99.46	0.000	91
Sample_SPB41.Cluster756cbin.1	98.62	0.394	217
Sample_SPB42.Cluster18534	92.41	2.247	13
Sample_SPB42.Cluster10349	86.49	0.806	24
Sample_SPB42.mb.127	89.33	0.000	38
Sample_SPB42.mb.104cbin.1	92.26	1.342	21
Sample_SPB42.mb.11cbin.1	95.43	3.413	149
Sample_SPB42.mb.106	90.79	1.515	394
Sample_SPB42.Cluster10307	93.95	0.000	36
Sample_SPB42.Cluster17197cbin.1	98.29	3.264	134
Sample_SPB42.Cluster188cbin.1	93.87	3.378	135
Sample_SPB42.Cluster89	87.58	2.393	101
Sample_SPB42.Cluster656cbin.1	90.71	1.118	182
Sample_SPB42.Cluster15277cbin.1	87.24	0.111	41
Sample_SPB42.Cluster65cbin.1	89.65	4.362	172
Sample_SPB42.mb.10	88.58	0.838	137
Sample_SPB42.mb.32cbin.1	97.31	1.075	113
Sample_SPB42.Cluster10714	95.07	0.000	21
Sample_SPB42.Cluster582	100.00	0.790	21

Sample_SPB42.mb.53	93.90	0.000	161
Sample_SPB42.Cluster15132cbin.1	98.06	0.000	85
Sample_SPB42.mb.140	92.02	1.006	103
Sample_SPB42.Cluster877	97.95	0.000	39
Sample_SPB42.mb.20	87.12	2.013	138
Sample_SPB42.Cluster75cbin.1	96.89	1.242	150
Sample_SPB42.mb.41	90.60	2.593	148
Sample_SPB42.mb.15	94.71	0.961	24
Sample_SPB42.Cluster8875cbin.1	98.19	0.289	137
Sample_SPB42.mb.72cbin.1	86.32	1.006	258
Sample_SPB42.mb.129	96.87	0.961	38
Sample_SPB42.mb.116	99.62	0.000	20
Sample_SPB42.mb.69cbin.1	89.53	2.534	322
Sample_SPB42.mb.94cbin.1	89.81	1.006	226
Sample_SPB42.mb.95cbin.1	96.60	0.782	52
Sample_SPB42.mb.75cbin.1	94.77	2.034	202
Sample_SPB42.mb.91cbin.1	89.04	3.236	174
Sample_SPB42.Cluster2849cbin.1	98.63	0.766	35
Sample_SPB42.mb.19cbin.1	93.52	1.141	357
Sample_SPB45.Cluster12822	94.94	1.284	21
Sample_SPB45.Cluster12958cbin.1	91.57	2.247	23
Sample_SPB45.Cluster12862	86.79	0.000	24
Sample_SPB45.Cluster1279cbin.1	88.50	3.091	216
Sample_SPB45.Cluster3400cbin.1	98.38	0.000	47
Sample_SPB45.mb.22cbin.1	85.08	1.612	40
Sample_SPB45.Cluster2056	98.60	0.139	23
Sample_SPB45.mb.26cbin.1	92.95	1.866	40
Sample_SPB45.mb.16	95.80	1.165	76
Sample_SPB45.mb.45	80.55	2.016	479
Sample_SPB45.Cluster10521cbin.1	95.83	0.000	201
Sample_SPB45.mb.11	89.66	2.348	105
Sample_SPB45.mb.19	96.86	2.705	284
Sample_SPB45.Cluster4014cbin.1	94.60	1.349	66
Sample_SPB45.mb.39cbin.1	88.97	2.013	221
Sample_SPB45.Cluster7580cbin.1	98.32	0.000	60
Sample_SPB45.Cluster6181	97.87	0.731	47
Sample_SPB45.mb.107cbin.1	85.34	0.000	36
Sample_SPB45.mb.62	90.16	4.494	56
Sample_SPB45.Cluster5347cbin.1	91.40	1.616	301
Sample_SPB45.mb.48cbin.1	89.93	1.733	54
Sample_SPB45.mb.25	81.98	1.342	91
Sample_SPB45.Cluster3720cbin.1	90.33	1.342	19
Sample_SPB45.mb.104cbin.1	89.06	3.027	378
Sample_SPB45.Cluster12776	100.00	0.000	13
Sample_SPB45.mb.5	80.82	2.684	248
Sample_SPB45.Cluster2337	97.95	0.000	30
Sample_SPB45.Cluster508	99.55	0.000	27
Sample_SPB45.Cluster6995cbin.1	94.63	2.684	92
Sample_SPB45.mb.20cbin.1	99.32	1.006	28
Sample_SPB45.mb.76cbin.1	91.05	0.671	34
Sample_SPB45.Cluster25cbin.1	92.68	1.140	211
Sample_SPB45.mb.93	96.37	3.714	57
Sample_SPB45.mb.80cbin.1	91.27	2.034	79
Sample_SPB45.mb.75cbin.1	99.10	0.020	151
Sample_SPB45.mb.60	92.25	0.000	28
Sample_SPB45.mb.98	90.60	1.342	44
Sample_SPB45.mb.81	93.03	0.696	189
Sample_SPB45.mb.82	97.58	0.806	49

Sample_SPB45.mb.78	84.74	0.000	26
Sample_SPB45.mb.54	97.73	0.578	158
Sample_SPB45.Cluster6357	99.61	0.576	83
Sample_SPB45.mb.95cbin.1	88.70	1.802	342
Sample_SPB45.mb.55cbin.1	85.87	0.557	109
Sample_SPB45.mb.41cbin.1	97.27	0.705	336
Sample_SPB45.mb.49	94.87	0.000	305
Sample_SPB45.mb.87cbin.1	97.24	1.301	59
Sample_SPB47.Cluster12751cbin.1	86.79	3.370	64
Sample_SPB47.Cluster3739	96.62	0.000	8
Sample_SPB47.Cluster2178cbin.1	85.71	0.000	32
Sample_SPB47.Cluster12	82.53	1.174	222
Sample_SPB47.Cluster22cbin.1	83.27	1.565	340
Sample_SPB47.Cluster1652cbin.1	93.95	0.671	152
Sample_SPB47.Cluster1233cbin.1	97.31	0.671	86
Sample_SPB47.Cluster2232cbin.1	92.59	0.805	85
Sample_SPB47.Cluster2856	96.64	0.000	23
Sample_SPB47.mb.36	92.41	1.123	37
Sample_SPB47.Cluster48cbin.1	97.31	1.275	44
Sample_SPB47.Cluster1049cbin.1	89.31	1.442	30
Sample_SPB47.Cluster1765	91.94	0.000	8
Sample_SPB47.Cluster366cbin.1	93.53	1.020	213
Sample_SPB47.Cluster5597	99.40	1.297	22
Sample_SPB47.Cluster29cbin.1	95.41	0.591	155
Sample_SPB47.mb.11	96.64	2.013	83
Sample_SPB47.Cluster1190cbin.1	97.60	2.500	20
Sample_SPB47.Cluster1024	98.75	0.625	21
Sample_SPB47.mb.112	91.94	2.617	123
Sample_SPB47.mb.25cbin.1	91.06	1.898	216
Sample_SPB47.Cluster12280cbin.1	82.91	0.632	56
Sample_SPB47.Cluster8455cbin.1	99.32	1.677	21
Sample_SPB47.Cluster7105cbin.1	98.65	0.000	23
Sample_SPB47.Cluster10640cbin.1	96.47	1.194	213
Sample_SPB47.mb.44	83.87	2.908	242
Sample_SPB47.Cluster10470	98.11	0.754	91
Sample_SPB47.Cluster8504	99.51	0.000	71
Sample_SPB47.mb.86	94.35	0.806	40
Sample_SPB47.mb.122	93.78	4.775	167
Sample_SPB47.mb.14	97.50	0.625	55
Sample_SPB47.mb.43cbin.1	87.42	0.848	333
Sample_SPB47.mb.41	89.11	0.000	77
Sample_SPB47.mb.30cbin.1	83.46	1.186	254
Sample_SPB47.mb.37	92.66	0.961	179
Sample_SPB47.mb.125	88.88	0.701	225
Sample_SPB47.mb.99	92.61	1.342	112
Sample_SPB47.mb.57	88.87	3.117	64
Sample_SPB47.Cluster7084cbin.1	98.92	0.537	63
Sample_SPB47.Cluster4651	98.84	0.961	78
Sample_SPB47.mb.79cbin.1	92.06	2.277	284
Sample_SPB47.mb.27	88.26	0.373	162
Sample_SPB47.mb.26cbin.1	93.21	4.956	103
Sample_SPB47.mb.49	82.99	0.371	448
Sample_SPB47.mb.88cbin.1	90.88	0.692	231
Sample_SPB47.mb.93	100.00	2.884	33
Sample_SPB47.mb.97cbin.1	81.26	0.849	44
Sample_SPB47.mb.98	98.49	0.000	51
Sample_SPB47.mb.84cbin.1	98.38	0.000	160
Sample_SPB47.mb.61cbin.1	99.03	2.330	281

Sample_SPC03.Cluster14085	92.69	3.370	13
Sample_SPC03.Cluster3141cbin.1	97.84	0.097	58
Sample_SPC03.Cluster11152	98.65	4.362	21
Sample_SPC03.mb.117	93.95	0.806	45
Sample_SPC03.Cluster257	84.89	1.342	84
Sample_SPC03.Cluster9900	96.64	2.684	20
Sample_SPC03.Cluster260cbin.1	85.87	1.845	248
Sample_SPC03.Cluster7720	97.98	0.671	24
Sample_SPC03.mb.24cbin.1	86.93	4.113	275
Sample_SPC03.mb.49	96.52	0.000	13
Sample_SPC03.mb.92cbin.1	82.30	2.678	255
Sample_SPC03.mb.22cbin.1	89.45	3.496	326
Sample_SPC03.Cluster10177	95.60	0.000	23
Sample_SPC03.mb.48	82.39	3.635	89
Sample_SPC03.Cluster5710cbin.1	91.61	0.671	59
Sample_SPC03.mb.103	92.20	3.784	239
Sample_SPC03.mb.104cbin.1	99.03	2.403	52
Sample_SPC03.mb.6cbin.1	98.60	0.932	90
Sample_SPC03.mb.106	97.83	0.000	47
Sample_SPC03.Cluster8613cbin.1	98.63	0.000	40
Sample_SPC03.mb.35cbin.1	95.95	3.068	235
Sample_SPC03.mb.44	94.29	0.680	95
Sample_SPC03.mb.113cbin.1	92.32	0.185	53
Sample_SPC03.mb.97cbin.1	86.57	4.997	93
Sample_SPC03.mb.57	95.97	0.671	67
Sample_SPC03.mb.2cbin.1	98.30	2.001	67
Sample_SPC03.mb.86cbin.1	98.99	0.000	47
Sample_SPC03.mb.114	96.61	4.227	100
Sample_SPC03.mb.12	93.66	3.190	175
Sample_SPC03.mb.46	97.64	0.754	288
Sample_SPC03.Cluster708	96.93	0.372	51
Sample_SPC04.Cluster214cbin.1	92.78	0.671	80
Sample_SPC04.Cluster376cbin.1	88.59	2.348	54
Sample_SPC04.mb.6	98.38	1.612	41
Sample_SPC04.Cluster1117cbin.1	91.94	3.020	41
Sample_SPC04.mb.13	100.00	0.115	17
Sample_SPC04.mb.76	90.66	1.333	27
Sample_SPC04.mb.80	91.29	2.247	30
Sample_SPC04.Cluster5731cbin.1	88.68	2.908	343
Sample_SPC04.mb.54cbin.1	81.76	1.677	320
Sample_SPC04.mb.34	81.73	1.686	253
Sample_SPC04.mb.78cbin.1	93.26	1.342	28
Sample_SPC04.Cluster5253	96.40	1.097	31
Sample_SPC04.Cluster120cbin.1	81.76	1.074	269
Sample_SPC04.Cluster907cbin.1	94.37	0.625	136
Sample_SPC04.mb.15cbin.1	96.45	0.435	227
Sample_SPC04.mb.29cbin.1	99.51	0.961	32
Sample_SPC04.Cluster11760cbin.1	97.98	0.000	42
Sample_SPC04.Cluster8782	99.32	0.335	18
Sample_SPC04.mb.83cbin.1	98.60	2.913	116
Sample_SPC04.mb.22cbin.1	91.69	1.250	175
Sample_SPC04.mb.47	98.65	0.671	14
Sample_SPC04.mb.59cbin.1	95.23	3.231	281
Sample_SPC04.Cluster5803cbin.1	88.00	4.065	374
Sample_SPC04.mb.85	85.65	1.906	91
Sample_SPC04.Cluster3414cbin.1	95.47	1.242	300
Sample_SPC04.mb.65cbin.1	82.74	1.972	336
Sample_SPC04.mb.93	80.36	1.562	422

Sample_SPC04.mb.82cbin.1	87.87	1.952	16
Sample_SPC04.mb.87	84.85	0.000	10
Sample_SPC04.mb.81	97.76	3.848	190
Sample_SPC04.mb.89	96.83	0.671	63
Sample_SPC04.mb.62cbin.1	95.16	0.073	26
Sample_SPC04.mb.69cbin.1	96.79	0.705	163
Sample_SPC04.mb.88cbin.1	99.42	0.192	127
Sample_SPC04.mb.92cbin.1	99.96	0.037	68
Sample_SPC06.Cluster3248cbin.1	84.67	0.806	20
Sample_SPC06.Cluster1387cbin.1	98.11	1.075	75
Sample_SPC06.mb.44cbin.1	94.94	1.123	6
Sample_SPC06.Cluster694	96.50	0.699	69
Sample_SPC06.Cluster5415	97.98	0.000	19
Sample_SPC06.Cluster328cbin.1	92.61	0.671	119
Sample_SPC06.mb.48	92.28	1.342	62
Sample_SPC06.Cluster1832cbin.1	94.63	1.342	12
Sample_SPC06.mb.55	100.00	0.115	60
Sample_SPC06.mb.71cbin.1	93.95	0.671	42
Sample_SPC06.Cluster1121	88.54	0.671	25
Sample_SPC06.Cluster5959cbin.1	97.98	1.677	31
Sample_SPC06.mb.21cbin.1	91.38	0.632	325
Sample_SPC06.mb.13	98.38	1.677	17
Sample_SPC06.mb.8	96.15	1.048	55
Sample_SPC06.mb.74	91.27	0.671	28
Sample_SPC06.mb.78cbin.1	88.36	2.013	59
Sample_SPC06.mb.35	90.40	2.684	230
Sample_SPC06.Cluster1414	100.00	0.227	15
Sample_SPC06.Cluster5280cbin.1	99.41	0.584	90
Sample_SPC06.mb.86	86.35	0.000	71
Sample_SPC06.mb.81	99.51	3.639	121
Sample_SPC06.mb.72cbin.1	96.59	3.741	241
Sample_SPC06.mb.59cbin.1	96.59	0.000	137
Sample_SPC06.mb.65	95.30	0.671	91
Sample_SPC06.mb.58cbin.1	93.66	3.196	272
Sample_SPC06.mb.68cbin.1	99.51	0.240	85
Sample_SPC06.mb.27	99.22	0.584	65
Sample_SPC06.Cluster4605	99.19	0.192	34
Sample_SPC06.Cluster1874cbin.1	93.91	1.185	483
Sample_SPC06.mb.38	90.39	0.123	223
Sample_SPC07.Cluster12284	82.45	0.000	138
Sample_SPC07.mb.117cbin.1	94.73	4.784	19
Sample_SPC07.Cluster12882	99.32	1.342	36
Sample_SPC07.Cluster7505cbin.1	88.60	3.416	315
Sample_SPC07.Cluster187cbin.1	91.94	1.342	25
Sample_SPC07.Cluster5885cbin.1	98.94	0.632	86
Sample_SPC07.Cluster8839cbin.1	85.97	4.291	552
Sample_SPC07.Cluster82cbin.1	90.93	2.745	131
Sample_SPC07.Cluster907	92.17	0.894	49
Sample_SPC07.Cluster598cbin.1	91.03	3.544	105
Sample_SPC07.Cluster923cbin.1	98.09	3.733	76
Sample_SPC07.Cluster7094	99.40	0.598	17
Sample_SPC07.Cluster4915cbin.1	97.46	0.021	84
Sample_SPC07.mb.107	94.12	0.766	154
Sample_SPC07.mb.12	80.53	0.000	157
Sample_SPC07.Cluster3423	100.00	0.000	20
Sample_SPC07.mb.4	92.41	4.494	14
Sample_SPC07.Cluster120	98.84	1.075	50
Sample_SPC07.Cluster2242	98.75	0.625	25

Sample_SPC07.Cluster12260	99.47	0.000	13
Sample_SPC07.mb.120	84.54	2.415	70
Sample_SPC07.mb.32cbin.1	95.89	0.000	21
Sample_SPC07.Cluster11122	97.98	0.000	40
Sample_SPC07.mb.108	98.65	1.006	14
Sample_SPC07.Cluster8152cbin.1	95.30	2.684	19
Sample_SPC07.mb.18	93.88	0.000	116
Sample_SPC07.mb.41	97.20	1.864	78
Sample_SPC07.mb.118cbin.1	95.97	1.342	70
Sample_SPC07.Cluster12891	99.36	0.632	62
Sample_SPC07.mb.25	88.81	2.040	192
Sample_SPC07.Cluster8463	98.86	0.000	24
Sample_SPC07.mb.93	92.59	1.342	35
Sample_SPC07.mb.30	97.27	0.961	36
Sample_SPC07.Cluster5320cbin.1	97.31	0.000	112
Sample_SPC07.mb.42	98.38	0.806	36
Sample_SPC07.mb.82cbin.1	88.25	1.405	57
Sample_SPC07.mb.85	82.88	0.000	45
Sample_SPC07.mb.8	86.66	0.000	38
Sample_SPC07.Cluster8650cbin.1	98.38	0.000	129
Sample_SPC07.mb.23	97.07	0.000	74
Sample_SPC07.mb.63	91.27	0.671	110
Sample_SPC07.mb.88cbin.1	85.57	2.705	186
Sample_SPC07.Cluster6991	99.61	0.961	84
Sample_SPC07.mb.75	91.83	0.894	208
Sample_SPC07.mb.47	94.13	4.591	115
Sample_SPC07.mb.96	95.05	0.881	37
Sample_SPC07.mb.78cbin.1	93.16	3.426	69
Sample_SPC07.mb.71	91.72	0.309	100
Sample_SPC07.mb.95	98.31	0.480	21
Sample_SPC08.Cluster17317	81.46	4.494	21
Sample_SPC08.Cluster17466	93.82	1.284	28
Sample_SPC08.Cluster160cbin.1	91.59	0.671	45
Sample_SPC08.Cluster3332cbin.1	98.38	0.364	60
Sample_SPC08.Cluster1267	81.65	0.000	30
Sample_SPC08.mb.118	91.57	1.685	14
Sample_SPC08.Cluster351cbin.1	87.08	4.823	40
Sample_SPC08.mb.10	95.80	1.165	100
Sample_SPC08.Cluster5062cbin.1	95.63	0.000	18
Sample_SPC08.Cluster315cbin.1	80.76	0.961	316
Sample_SPC08.Cluster219cbin.1	93.47	1.496	192
Sample_SPC08.Cluster14570cbin.1	97.31	0.671	62
Sample_SPC08.Cluster4847cbin.1	96.64	4.697	17
Sample_SPC08.mb.29	98.66	4.111	23
Sample_SPC08.Cluster7170	99.40	0.598	11
Sample_SPC08.mb.110	93.62	0.223	76
Sample_SPC08.Cluster7553	100.00	0.480	11
Sample_SPC08.Cluster15847	93.26	0.000	24
Sample_SPC08.Cluster588cbin.1	95.50	1.250	151
Sample_SPC08.Cluster6706cbin.1	99.98	1.497	20
Sample_SPC08.mb.113	90.64	3.355	299
Sample_SPC08.Cluster509	99.55	0.000	45
Sample_SPC08.mb.11	97.74	2.028	47
Sample_SPC08.mb.66	91.57	1.685	46
Sample_SPC08.mb.54	89.93	1.333	59
Sample_SPC08.Cluster9250	97.09	0.000	27
Sample_SPC08.Cluster15302cbin.1	95.65	2.173	99
Sample_SPC08.mb.26	89.93	4.697	46

Sample_SPC08.Cluster9757	99.32	0.335	26
Sample_SPC08.mb.124	83.47	3.993	290
Sample_SPC08.mb.127	84.21	2.834	269
Sample_SPC08.Cluster9827cbin.1	98.64	0.675	85
Sample_SPC08.mb.93	84.83	3.370	25
Sample_SPC08.Cluster5156cbin.1	80.20	2.153	157
Sample_SPC08.mb.56	80.48	0.838	305
Sample_SPC08.mb.91	98.38	0.806	44
Sample_SPC08.mb.58	89.20	0.671	218
Sample_SPC08.mb.49	93.91	0.675	36
Sample_SPC08.mb.18cbin.1	94.83	1.442	71
Sample_SPC08.mb.79	84.55	0.000	259
Sample_SPC08.Cluster7435cbin.1	96.21	1.124	343
Sample_SPC08.mb.4	91.15	2.013	245
Sample_SPC08.mb.76	89.31	4.026	81
Sample_SPC08.mb.51	97.59	3.044	51
Sample_SPC08.mb.55	97.58	0.806	127
Sample_SPC08.Cluster7176cbin.1	95.03	2.323	56
Sample_SPC08.mb.61	93.95	1.169	196
Sample_SPC08.mb.9	97.34	1.932	49
Sample_SPC08.mb.41	96.36	3.343	386
Sample_SPC09.Cluster31cbin.1	86.55	3.691	223
Sample_SPC09.Cluster1936cbin.1	99.32	0.671	76
Sample_SPC09.Cluster90	86.04	0.671	49
Sample_SPC09.Cluster8082cbin.1	98.16	3.639	62
Sample_SPC09.Cluster2849cbin.1	96.83	0.021	90
Sample_SPC09.mb.12	94.72	0.000	167
Sample_SPC09.mb.33	96.81	1.598	57
Sample_SPC09.mb.16	86.05	0.680	64
Sample_SPC09.Cluster5456	99.98	1.696	18
Sample_SPC09.mb.29	99.32	0.671	18
Sample_SPC09.mb.69	83.54	2.215	414
Sample_SPC09.mb.67cbin.1	95.97	0.335	55
Sample_SPC09.Cluster2177cbin.1	87.91	1.424	354
Sample_SPC09.Cluster5059cbin.1	97.69	4.960	66
Sample_SPC09.mb.21	99.32	0.671	18
Sample_SPC09.Cluster6794cbin.1	93.95	1.342	221
Sample_SPC09.Cluster6170	98.06	0.000	59
Sample_SPC09.Cluster6340cbin.1	98.49	0.000	17
Sample_SPC09.mb.87cbin.1	95.67	2.403	38
Sample_SPC09.Cluster5921cbin.1	92.98	0.501	277
Sample_SPC09.mb.82	95.16	0.959	55
Sample_SPC09.Cluster4566cbin.1	98.52	0.669	29
Sample_SPC09.mb.83	98.10	0.650	40
Sample_SPC09.mb.73cbin.1	99.23	3.846	214
Sample_SPC10.Cluster2969	91.25	0.671	40
Sample_SPC10.mb.10	80.53	0.489	16
Sample_SPC10.Cluster27	90.60	0.000	11
Sample_SPC10.Cluster2394cbin.1	97.33	0.632	99
Sample_SPC10.Cluster1785	94.75	1.902	103
Sample_SPC10.mb.20	89.22	0.421	211
Sample_SPC10.mb.26	93.03	0.632	56
Sample_SPC10.Cluster4267cbin.1	97.98	0.335	35
Sample_SPC10.mb.11	88.25	4.910	162
Sample_SPC10.Cluster5577cbin.1	90.57	0.000	32
Sample_SPC10.mb.23cbin.1	96.87	0.000	13
Sample_SPC10.mb.12cbin.1	94.63	0.134	143
Sample_SPC10.mb.49cbin.1	91.44	0.680	69

Sample_SPC10.Cluster4683cbin.1	99.51	2.415	75
Sample_SPC10.mb.46cbin.1	94.49	0.850	91
Sample_SPC10.mb.73	92.40	4.761	66
Sample_SPC10.mb.2	90.25	4.225	398
Sample_SPC10.mb.34	93.46	3.216	205
Sample_SPC10.mb.59cbin.1	91.71	0.140	187
Sample_SPC10.Cluster2004cbin.1	96.75	1.115	40
Sample_SPC10.mb.37	96.83	1.028	152
Sample_SPC10.mb.29cbin.1	89.52	0.495	50
Sample_SPC10.mb.1cbin.1	96.47	0.750	311
Sample_SPC10.Cluster3694cbin.1	90.35	2.535	219
Sample_SPC10.mb.62cbin.1	98.37	0.187	44
Sample_SPC10.Cluster140	98.80	0.334	34
Sample_SPC11.Cluster4048	95.96	0.000	17
Sample_SPC11.Cluster2715cbin.1	84.94	1.006	187
Sample_SPC11.Cluster288	84.89	1.342	28
Sample_SPC11.Cluster5831	98.48	0.000	33
Sample_SPC11.Cluster4863	99.19	0.115	13
Sample_SPC11.Cluster134cbin.1	90.80	1.775	251
Sample_SPC11.Cluster10732cbin.1	97.30	0.134	114
Sample_SPC11.Cluster160	97.04	2.419	35
Sample_SPC11.Cluster294cbin.1	85.10	1.360	161
Sample_SPC11.Cluster4327cbin.1	99.40	1.497	43
Sample_SPC11.Cluster1525cbin.1	89.83	1.342	218
Sample_SPC11.Cluster6659cbin.1	82.29	3.704	403
Sample_SPC11.Cluster2946	97.27	4.026	16
Sample_SPC11.Cluster83cbin.1	96.30	1.342	58
Sample_SPC11.mb.104	91.11	1.733	277
Sample_SPC11.Cluster1285	98.75	0.625	24
Sample_SPC11.Cluster1447cbin.1	97.11	0.176	60
Sample_SPC11.mb.119	95.78	2.531	121
Sample_SPC11.mb.133cbin.1	92.31	2.684	78
Sample_SPC11.Cluster8333	98.65	1.006	18
Sample_SPC11.Cluster6575	96.07	1.570	39
Sample_SPC11.Cluster10755cbin.1	98.51	0.671	86
Sample_SPC11.mb.53	92.41	1.685	23
Sample_SPC11.Cluster6755cbin.1	95.20	0.000	24
Sample_SPC11.Cluster10067cbin.1	98.53	2.348	54
Sample_SPC11.mb.105	85.89	2.005	120
Sample_SPC11.mb.41	91.90	1.342	178
Sample_SPC11.mb.116cbin.1	99.33	0.000	34
Sample_SPC11.Cluster6184	98.86	0.000	17
Sample_SPC11.mb.40cbin.1	87.33	0.543	207
Sample_SPC11.mb.10	97.22	0.483	55
Sample_SPC11.mb.114	98.65	0.000	21
Sample_SPC11.mb.24	93.26	0.961	30
Sample_SPC11.mb.76	88.88	0.854	26
Sample_SPC11.Cluster2437cbin.1	89.96	4.270	211
Sample_SPC11.mb.136	97.91	0.949	130
Sample_SPC11.mb.2	98.63	0.909	26
Sample_SPC11.mb.51	99.32	3.355	204
Sample_SPC11.mb.46	89.55	2.294	192
Sample_SPC11.mb.64	96.42	0.000	69
Sample_SPC11.mb.3cbin.1	89.83	0.680	241
Sample_SPC11.Cluster4045cbin.1	98.84	1.153	78
Sample_SPC11.mb.75	94.84	2.013	202
Sample_SPC11.mb.70cbin.1	91.94	0.000	36
Sample_SPC11.mb.82cbin.1	89.85	1.342	107

Sample_SPC11.mb.47	98.55	0.480	43
Sample_SPC11.mb.56	82.70	1.415	434
Sample_SPC11.mb.110	89.38	2.518	154
Sample_SPC11.mb.6	100.00	0.000	30
Sample_SPC11.mb.44	95.13	4.291	158
Sample_SPC11.mb.66	95.30	1.006	93
Sample_SPC11.mb.95cbin.1	89.97	0.961	219
Sample_SPC11.mb.37cbin.1	93.83	0.173	414
Sample_SPC11.mb.99	90.54	0.373	62
Sample_SPC11.mb.73cbin.1	96.22	1.119	90
Sample_SPC11.mb.86cbin.1	96.60	4.213	82
Sample_SPC11.mb.94	97.31	0.000	164
Sample_SPC12.mb.1	85.34	3.571	114
Sample_SPC12.Cluster15328cbin.1	82.88	4.870	440
Sample_SPC12.Cluster5908cbin.1	96.48	1.282	173
Sample_SPC12.Cluster17469	86.23	1.600	242
Sample_SPC12.mb.103	87.92	2.247	27
Sample_SPC12.Cluster480	86.48	2.364	55
Sample_SPC12.mb.126	97.58	0.806	44
Sample_SPC12.Cluster2893cbin.1	96.63	0.000	23
Sample_SPC12.Cluster6801cbin.1	95.97	0.000	29
Sample_SPC12.Cluster13456	99.32	0.335	25
Sample_SPC12.Cluster2319	89.38	1.342	21
Sample_SPC12.Cluster14444cbin.1	97.31	1.677	49
Sample_SPC12.Cluster13847cbin.1	85.62	4.362	299
Sample_SPC12.Cluster11106cbin.1	91.02	3.490	312
Sample_SPC12.mb.36	91.57	0.160	32
Sample_SPC12.mb.138	81.89	3.691	359
Sample_SPC12.mb.131	82.55	0.671	391
Sample_SPC12.mb.107	94.67	0.680	240
Sample_SPC12.mb.117cbin.1	80.20	0.000	18
Sample_SPC12.Cluster12531cbin.1	92.98	2.046	77
Sample_SPC12.mb.32	98.65	0.000	14
Sample_SPC12.mb.114	99.29	0.480	165
Sample_SPC12.mb.122cbin.1	100.00	0.000	19
Sample_SPC12.mb.113cbin.1	93.27	0.584	108
Sample_SPC12.mb.43	97.71	0.020	168
Sample_SPC12.mb.82cbin.1	90.66	4.111	49
Sample_SPC12.mb.133cbin.1	97.57	1.409	111
Sample_SPC12.mb.20	96.15	0.961	33
Sample_SPC12.mb.56cbin.1	97.17	1.568	116
Sample_SPC12.mb.73	98.60	1.864	122
Sample_SPC12.mb.132	94.79	2.323	160
Sample_SPC12.mb.22	94.63	1.342	71
Sample_SPC12.mb.86	82.55	2.348	72
Sample_SPC12.mb.61	98.29	0.632	103
Sample_SPC12.mb.8	99.10	3.355	76
Sample_SPC12.Cluster9084cbin.1	91.74	0.769	527
Sample_SPC12.mb.72cbin.1	92.03	2.092	225
Sample_SPC12.mb.53	90.31	0.769	70
Sample_SPC12.mb.81	97.70	1.201	83
Sample_SPC12.mb.137	90.96	1.672	236
Sample_SPC12.mb.23	97.58	2.850	147
Sample_SPC12.mb.60cbin.1	97.78	3.711	115
Sample_SPC12.mb.54	93.58	0.800	29
Sample_SPC12.mb.4cbin.1	88.32	0.466	49
Sample_SPC12.mb.91cbin.1	99.46	1.075	231
Sample_SPC13.Cluster1460cbin.1	97.17	0.806	143

Sample_SPC13.Cluster7173	82.37	0.671	275
Sample_SPC13.Cluster8354	94.63	0.894	70
Sample_SPC13.Cluster7899cbin.1	98.65	0.335	15
Sample_SPC13.Cluster37cbin.1	82.98	0.680	433
Sample_SPC13.Cluster265cbin.1	96.44	0.000	30
Sample_SPC13.Cluster3818cbin.1	99.98	1.497	23
Sample_SPC13.mb.37cbin.1	85.90	0.134	39
Sample_SPC13.Cluster6624	99.32	1.006	18
Sample_SPC13.Cluster4518cbin.1	97.98	0.000	34
Sample_SPC13.Cluster1373	98.75	0.625	33
Sample_SPC13.mb.3	97.59	0.000	25
Sample_SPC13.mb.103	98.40	0.000	132
Sample_SPC13.mb.14cbin.1	89.97	0.076	15
Sample_SPC13.mb.47	91.27	1.342	35
Sample_SPC13.Cluster5690cbin.1	99.51	0.483	99
Sample_SPC13.mb.29	97.71	1.121	59
Sample_SPC13.mb.52cbin.1	92.61	1.891	76
Sample_SPC13.mb.43	95.96	1.612	123
Sample_SPC13.mb.55	97.65	0.713	93
Sample_SPC13.mb.49	97.98	0.671	141
Sample_SPC13.mb.88	80.15	1.008	312
Sample_SPC13.Cluster8560cbin.1	100.00	0.632	68
Sample_SPC13.mb.59	96.75	1.898	132
Sample_SPC13.mb.72	97.98	0.384	114
Sample_SPC13.mb.24cbin.1	95.01	0.480	48
Sample_SPC13.mb.20	92.98	0.000	48
Sample_SPC13.mb.15	99.62	0.000	16
Sample_SPC13.Cluster3275cbin.1	92.80	3.965	50
Sample_SPC13.mb.57	97.11	0.000	41
Sample_SPC13.mb.9	97.57	2.393	204
Sample_SPC13.mb.53	99.21	1.759	57
Sample_SPC13.Cluster5341cbin.1	99.61	0.512	87
Sample_SPC13.mb.48cbin.1	93.43	2.210	79
Sample_SPC13.Cluster3765cbin.1	96.72	0.743	107
Sample_SPC13.mb.79	95.11	0.961	36
Sample_SPC13.mb.66cbin.1	82.15	0.632	353
Sample_SPC13.mb.83	97.31	0.335	44
Sample_SPC13.Cluster2319cbin.1	99.42	2.051	136
Sample_SPC13.mb.76	98.65	0.167	72
Sample_SPC13.mb.8	97.22	0.000	266
Sample_SPC13.mb.56	91.62	0.746	89
Sample_SPC13.mb.54	99.40	0.000	27
Sample_SPC13.mb.77	97.46	0.652	209
Sample_SPC13.mb.84	94.20	0.185	92
Sample_SPC13.Cluster411	98.56	0.533	31
Sample_SPC13.mb.70cbin.1	95.71	4.146	152
Sample_SPC15.mb.26cbin.1	92.13	0.806	73
Sample_SPC15.Cluster10481	99.32	1.118	18
Sample_SPC15.Cluster5266	93.29	0.894	223
Sample_SPC15.Cluster5521cbin.1	90.84	0.000	23
Sample_SPC15.mb.22	93.95	0.671	52
Sample_SPC15.Cluster4826cbin.1	91.72	2.624	179
Sample_SPC15.Cluster2952	96.22	0.898	55
Sample_SPC15.Cluster6cbin.1	88.50	2.173	290
Sample_SPC15.Cluster5604cbin.1	85.91	0.732	259
Sample_SPC15.mb.51	90.92	0.000	120
Sample_SPC15.Cluster1367	98.63	0.000	54
Sample_SPC15.Cluster11881	90.57	0.261	30

Sample_SPC15.mb.31	92.30	0.657	251
Sample_SPC15.mb.30	95.41	2.028	129
Sample_SPC15.mb.66	80.70	0.000	61
Sample_SPC15.mb.17	99.36	1.896	113
Sample_SPC15.mb.59	84.56	0.000	49
Sample_SPC15.mb.40	85.92	2.323	305
Sample_SPC15.mb.72cbin.1	91.94	1.342	25
Sample_SPC15.mb.55	83.20	0.754	34
Sample_SPC15.mb.80cbin.1	94.14	0.740	86
Sample_SPC15.mb.48	97.97	0.284	156
Sample_SPC15.mb.83	92.57	0.671	55
Sample_SPC15.mb.25cbin.1	95.12	0.701	118
Sample_SPC15.Cluster2024cbin.1	99.07	0.361	51
Sample_SPC16.mb.2	86.20	4.026	395
Sample_SPC16.Cluster4036cbin.1	96.41	1.265	148
Sample_SPC16.mb.29cbin.1	82.38	3.243	253
Sample_SPC16.Cluster8896	94.47	0.707	64
Sample_SPC16.Cluster3365	99.98	0.898	31
Sample_SPC16.Cluster799cbin.1	98.12	0.000	104
Sample_SPC16.mb.25cbin.1	95.40	2.560	216
Sample_SPC16.Cluster10140	99.47	0.000	24
Sample_SPC16.mb.37	95.53	2.589	157
Sample_SPC16.Cluster7549cbin.1	95.97	0.671	40
Sample_SPC16.Cluster5477cbin.1	82.09	1.326	127
Sample_SPC16.mb.27cbin.1	97.58	4.589	57
Sample_SPC16.Cluster7014cbin.1	96.77	1.751	125
Sample_SPC16.mb.71	96.01	0.000	51
Sample_SPC16.Cluster3708	98.07	0.000	52
Sample_SPC16.mb.62	87.98	2.761	72
Sample_SPC17.mb.37	97.98	0.000	30
Sample_SPC17.Cluster1353	96.46	0.000	57
Sample_SPC17.Cluster1055	99.36	0.000	33
Sample_SPC17.Cluster306cbin.1	97.31	0.356	151
Sample_SPC17.mb.41	99.03	0.961	36
Sample_SPC17.Cluster1142cbin.1	98.96	2.095	86
Sample_SPC17.Cluster2779cbin.1	92.95	1.342	360
Sample_SPC17.mb.60	95.27	0.717	82
Sample_SPC17.mb.1cbin.1	95.32	0.097	35
Sample_SPC17.Cluster1240	94.35	1.154	152
Sample_SPC17.Cluster3452cbin.1	97.77	1.868	216
Sample_SPC17.mb.28cbin.1	89.64	2.933	142
Sample_SPC17.Cluster48cbin.1	86.31	0.322	103
Sample_SPC17.mb.64cbin.1	91.72	0.944	109
Sample_SPC17.Cluster66cbin.1	97.98	3.231	87
Sample_SPC17.mb.51cbin.1	92.11	0.805	179
Sample_SPC17.mb.43cbin.1	82.27	0.738	155
Sample_SPC17.mb.74cbin.1	93.28	0.680	67
Sample_SPC17.Cluster2305	89.57	4.744	116
Sample_SPC17.Cluster887cbin.1	93.32	0.371	255
Sample_SPC17.Cluster2479cbin.1	92.39	1.169	67
Sample_SPC17.Cluster2998cbin.1	98.83	2.923	102
Sample_SPC17.mb.2cbin.1	93.21	0.941	51
Sample_SPC17.mb.45cbin.1	91.11	1.153	458
Sample_SPC17.Cluster1300cbin.1	99.61	0.897	56
Sample_SPC17.Cluster1839cbin.1	89.00	1.127	75
Sample_SPC17.mb.73cbin.1	99.37	0.037	64
Sample_SPC20.Cluster11157	85.91	1.123	265
Sample_SPC20.Cluster6091cbin.1	92.74	2.419	64

Sample_SPC20.mb.26cbin.1	90.66	1.333	66
Sample_SPC20.mb.23	93.54	0.000	101
Sample_SPC20.Cluster8391cbin.1	98.99	1.677	200
Sample_SPC20.mb.90	81.03	0.000	117
Sample_SPC20.Cluster2624cbin.1	93.28	0.000	37
Sample_SPC20.mb.93	93.26	1.342	29
Sample_SPC20.Cluster6543	97.09	0.000	24
Sample_SPC20.mb.82	91.18	2.742	200
Sample_SPC20.mb.24cbin.1	89.93	1.006	10
Sample_SPC20.Cluster8953cbin.1	99.32	1.342	37
Sample_SPC20.mb.41	93.07	2.512	109
Sample_SPC20.mb.60cbin.1	96.28	3.044	166
Sample_SPC20.Cluster7734	81.52	4.106	28
Sample_SPC20.Cluster7449cbin.1	94.60	0.483	90
Sample_SPC20.Cluster4172cbin.1	93.67	0.738	131
Sample_SPC20.mb.94	95.65	2.531	159
Sample_SPC20.mb.3	96.31	1.228	77
Sample_SPC20.mb.20	98.50	1.482	52
Sample_SPC20.mb.66	97.29	1.342	67
Sample_SPC20.mb.59	93.91	0.671	50
Sample_SPC20.mb.69	84.50	3.653	85
Sample_SPC20.mb.18	99.24	0.377	26
Sample_SPC20.mb.30	93.94	2.230	75
Sample_SPC20.mb.61	90.93	1.461	126
Sample_SPC20.Cluster9809cbin.1	88.12	1.794	555
Sample_SPC20.mb.65	89.13	1.127	160
Sample_SPC21.mb.16cbin.1	80.34	0.000	62
Sample_SPC21.Cluster3681	97.84	1.173	42
Sample_SPC21.Cluster5073	91.25	1.342	21
Sample_SPC21.mb.30	87.80	1.510	124
Sample_SPC21.mb.29	93.47	1.118	140
Sample_SPC21.mb.23cbin.1	94.96	2.502	93
Sample_SPC21.mb.39	95.74	0.671	80
Sample_SPC21.mb.10	92.41	1.053	59
Sample_SPC21.mb.35	92.15	0.000	223
Sample_SPC21.mb.33cbin.1	92.40	1.476	160
Sample_SPC21.mb.73cbin.1	88.88	0.284	42
Sample_SPC21.mb.24cbin.1	98.65	1.677	47
Sample_SPC21.mb.84cbin.1	98.43	0.000	75
Sample_SPC21.mb.6	93.84	0.134	179
Sample_SPC21.mb.88	96.50	1.165	74
Sample_SPC21.mb.77	94.63	0.335	38
Sample_SPC21.Cluster9797cbin.1	99.32	1.006	28
Sample_SPC21.Cluster11874cbin.1	93.95	0.020	52
Sample_SPC21.mb.18	98.65	0.671	23
Sample_SPC21.mb.78cbin.1	98.32	0.000	106
Sample_SPC21.mb.62	92.63	1.360	227
Sample_SPC21.mb.70cbin.1	92.46	0.961	23
Sample_SPC21.mb.45cbin.1	93.56	1.559	98
Sample_SPC21.mb.8cbin.1	89.59	2.348	172
Sample_SPC21.Cluster7727	96.60	0.000	27
Sample_SPC21.mb.72	95.30	0.000	20
Sample_SPC21.Cluster4981cbin.1	89.62	2.674	69
Sample_SPC21.mb.91	100.00	0.480	41
Sample_SPC21.Cluster6746	98.38	1.075	108
Sample_SPC21.mb.87cbin.1	99.03	0.000	45
Sample_SPC21.Cluster7572cbin.1	97.91	0.185	76
Sample_SPC21.mb.60	90.79	2.817	174

Sample_SPC21.mb.93	90.86	0.826	412
Sample_SPC21.Cluster6987cbin.1	89.94	2.948	290
Sample_SPC21.Cluster6056	97.88	0.576	70
Sample_SPC21.mb.98	95.20	0.806	122
Sample_SPC21.mb.1	90.44	0.836	60
Sample_SPC22.Cluster17904	91.57	2.247	12
Sample_SPC22.Cluster7620cbin.1	96.55	1.880	30
Sample_SPC22.Cluster16607	89.03	0.000	19
Sample_SPC22.Cluster7396	97.84	0.000	39
Sample_SPC22.mb.117	80.33	0.000	9
Sample_SPC22.Cluster481	100.00	1.411	52
Sample_SPC22.Cluster8892cbin.1	92.26	1.342	26
Sample_SPC22.Cluster110cbin.1	88.32	2.969	123
Sample_SPC22.Cluster184	98.32	0.671	45
Sample_SPC22.mb.107	100.00	0.115	13
Sample_SPC22.mb.21	85.51	0.862	54
Sample_SPC22.Cluster9571cbin.1	89.45	0.632	38
Sample_SPC22.mb.41	85.67	1.123	26
Sample_SPC22.Cluster15262	82.94	0.894	338
Sample_SPC22.mb.12	99.01	0.838	64
Sample_SPC22.Cluster17294	98.95	0.000	24
Sample_SPC22.Cluster17647cbin.1	95.42	1.408	214
Sample_SPC22.Cluster1719cbin.1	97.95	0.680	35
Sample_SPC22.mb.140cbin.1	95.41	1.342	59
Sample_SPC22.mb.130cbin.1	89.07	0.000	232
Sample_SPC22.mb.33	98.60	1.668	89
Sample_SPC22.mb.103	98.65	2.348	156
Sample_SPC22.mb.31	94.29	0.671	105
Sample_SPC22.mb.113	98.68	1.843	50
Sample_SPC22.Cluster1939cbin.1	96.81	1.477	36
Sample_SPC22.Cluster14493cbin.1	97.48	3.836	299
Sample_SPC22.mb.46	83.98	4.884	199
Sample_SPC22.mb.78	98.38	0.000	58
Sample_SPC22.mb.143cbin.1	93.95	1.342	32
Sample_SPC22.mb.16	99.10	0.671	24
Sample_SPC22.mb.62	89.64	1.489	283
Sample_SPC22.mb.38	96.63	0.000	55
Sample_SPC22.mb.60cbin.1	83.90	4.255	352
Sample_SPC22.mb.65cbin.1	99.32	2.348	140
Sample_SPC22.Cluster7098cbin.1	97.99	1.760	184
Sample_SPC22.mb.126	99.24	0.217	42
Sample_SPC22.mb.54	98.07	4.326	45
Sample_SPC22.mb.39cbin.1	88.25	0.887	513
Sample_SPC22.mb.30	85.81	0.480	24
Sample_SPC22.mb.98	87.52	1.398	62
Sample_SPC22.mb.43	96.96	4.310	289
Sample_SPC22.mb.66	90.26	2.782	208
Sample_SPC22.mb.127	94.95	4.384	105
Sample_SPC22.mb.125cbin.1	96.04	0.898	148
Sample_SPC22.mb.45cbin.1	94.17	3.333	353
Sample_SPC22.mb.86cbin.1	99.62	0.000	36
Sample_SPC22.mb.68cbin.1	95.76	1.486	150
Sample_SPC22.mb.81	90.22	0.563	119
Sample_SPC22.mb.87cbin.1	99.28	3.841	80
Sample_SPC25.Cluster24cbin.1	91.44	1.510	163
Sample_SPC25.Cluster19206cbin.1	99.05	0.943	117
Sample_SPC25.mb.5	80.17	0.000	117
Sample_SPC25.Cluster5384cbin.1	97.31	0.671	31

Sample_SPC25.Cluster3917	90.80	0.000	74
Sample_SPC25.mb.10	82.55	0.335	28
Sample_SPC25.Cluster14599cbin.1	95.97	1.901	44
Sample_SPC25.Cluster2875	96.07	0.000	53
Sample_SPC25.Cluster13107cbin.1	99.32	0.000	24
Sample_SPC25.Cluster6801	98.20	0.898	52
Sample_SPC25.Cluster11604cbin.1	98.65	2.684	26
Sample_SPC25.mb.27	91.94	0.894	196
Sample_SPC25.mb.67	92.00	1.333	12
Sample_SPC25.Cluster33	98.69	0.909	77
Sample_SPC25.Cluster4444cbin.1	84.71	0.090	35
Sample_SPC25.mb.107cbin.1	82.49	2.796	290
Sample_SPC25.mb.23cbin.1	93.83	0.000	141
Sample_SPC25.Cluster17392cbin.1	98.32	3.803	107
Sample_SPC25.mb.118	84.46	2.040	393
Sample_SPC25.Cluster13379cbin.1	99.27	1.288	102
Sample_SPC25.mb.117	91.25	0.000	76
Sample_SPC25.mb.110cbin.1	97.27	1.342	31
Sample_SPC25.mb.102cbin.1	97.98	1.073	21
Sample_SPC25.mb.56	97.78	1.700	154
Sample_SPC25.mb.103cbin.1	97.09	3.355	42
Sample_SPC25.mb.66	87.91	3.020	190
Sample_SPC25.Cluster12091cbin.1	96.45	3.118	350
Sample_SPC25.mb.47	99.75	0.480	20
Sample_SPC25.mb.59	91.66	0.000	27
Sample_SPC25.mb.87	82.90	1.677	107
Sample_SPC25.mb.82cbin.1	97.31	0.000	155
Sample_SPC25.mb.98	86.22	2.107	315
Sample_SPC25.mb.85	90.24	2.898	81
Sample_SPC25.mb.92cbin.1	97.43	3.301	326
Sample_SPC25.mb.14cbin.1	90.21	1.346	487
Sample_SPC25.mb.4cbin.1	98.80	0.322	37
Sample_SPC26.Cluster285cbin.1	91.92	0.061	54
Sample_SPC26.mb.126	86.23	0.671	242
Sample_SPC26.mb.12	88.88	0.854	37
Sample_SPC26.Cluster664cbin.1	83.66	3.020	55
Sample_SPC26.Cluster5521	91.94	0.000	26
Sample_SPC26.mb.114	95.13	1.342	61
Sample_SPC26.Cluster4938cbin.1	89.08	0.000	71
Sample_SPC26.mb.21	96.73	1.165	109
Sample_SPC26.mb.105	83.95	1.174	215
Sample_SPC26.Cluster4153cbin.1	97.31	0.000	21
Sample_SPC26.Cluster446cbin.1	80.90	0.000	24
Sample_SPC26.Cluster8877	95.97	0.335	14
Sample_SPC26.Cluster522cbin.1	88.22	0.185	62
Sample_SPC26.Cluster3107	98.20	0.000	35
Sample_SPC26.Cluster20cbin.1	96.27	0.621	135
Sample_SPC26.mb.41	93.73	0.335	58
Sample_SPC26.mb.118cbin.1	98.65	0.671	42
Sample_SPC26.mb.38	82.14	0.000	298
Sample_SPC26.mb.128	99.03	0.000	27
Sample_SPC26.mb.24cbin.1	98.14	0.000	47
Sample_SPC26.Cluster8628	94.73	0.292	56
Sample_SPC26.mb.29	98.79	0.000	19
Sample_SPC26.mb.20cbin.1	98.65	0.671	22
Sample_SPC26.mb.48	90.60	1.403	51
Sample_SPC26.mb.28cbin.1	95.26	2.104	26
Sample_SPC26.Cluster2762cbin.1	88.85	2.664	378

Sample_SPC26.mb.109cbin.1	94.05	1.075	457
Sample_SPC26.mb.15	98.49	0.000	26
Sample_SPC26.Cluster7097	95.81	1.538	199
Sample_SPC26.mb.71cbin.1	96.64	1.565	87
Sample_SPC26.mb.35	87.14	0.507	29
Sample_SPC26.mb.51	87.73	1.369	238
Sample_SPC26.mb.16cbin.1	92.17	3.931	304
Sample_SPC26.mb.62cbin.1	94.43	2.308	108
Sample_SPC26.mb.82	87.91	1.097	94
Sample_SPC26.mb.53cbin.1	87.02	0.641	353
Sample_SPC26.mb.6cbin.1	99.27	0.241	144
Sample_SPC26.mb.34	95.57	0.769	98
Sample_SPC26.mb.50	94.99	0.495	78
Sample_SPC27.Cluster3329cbin.1	93.93	1.342	24
Sample_SPC27.Cluster9815	98.66	0.111	39
Sample_SPC27.Cluster546	92.02	1.165	61
Sample_SPC27.Cluster858cbin.1	97.58	2.217	121
Sample_SPC27.Cluster6911	99.16	0.000	31
Sample_SPC27.Cluster228cbin.1	90.57	0.671	34
Sample_SPC27.Cluster4231	95.97	0.671	36
Sample_SPC27.Cluster103cbin.1	97.98	1.384	104
Sample_SPC27.mb.52	81.03	0.000	33
Sample_SPC27.Cluster120cbin.1	97.45	0.000	96
Sample_SPC27.Cluster5420cbin.1	96.64	0.000	22
Sample_SPC27.mb.107cbin.1	99.04	0.000	88
Sample_SPC27.Cluster3062cbin.1	95.93	0.671	18
Sample_SPC27.mb.117	95.56	1.582	131
Sample_SPC27.mb.109cbin.1	86.57	1.677	97
Sample_SPC27.Cluster2068cbin.1	97.04	0.000	19
Sample_SPC27.Cluster7512	94.63	0.335	37
Sample_SPC27.Cluster5238cbin.1	97.58	0.949	139
Sample_SPC27.Cluster323cbin.1	99.55	0.444	24
Sample_SPC27.mb.100cbin.1	92.17	1.700	68
Sample_SPC27.mb.20cbin.1	96.06	4.328	462
Sample_SPC27.Cluster66cbin.1	99.07	0.691	27
Sample_SPC27.mb.121cbin.1	89.24	0.021	74
Sample_SPC27.mb.37	94.93	0.000	61
Sample_SPC27.Cluster1521cbin.1	95.84	0.480	38
Sample_SPC27.Cluster9403cbin.1	95.95	0.826	296
Sample_SPC27.mb.41	93.23	0.740	33
Sample_SPC27.mb.120	87.43	4.535	461
Sample_SPC27.mb.54cbin.1	83.55	0.000	74
Sample_SPC27.mb.79cbin.1	97.04	2.373	95
Sample_SPC27.mb.28	98.30	1.368	41
Sample_SPC27.mb.71	81.97	0.961	29
Sample_SPC27.mb.1cbin.1	87.34	0.000	70
Sample_SPC27.Cluster3474	95.70	1.672	66
Sample_SPC27.mb.30	98.23	0.000	34
Sample_SPC27.Cluster3889cbin.1	98.46	1.089	87
Sample_SPC27.mb.93	94.30	0.000	59
Sample_SPC27.mb.81	92.72	0.671	74
Sample_SPC27.mb.85	96.84	1.324	85
Sample_SPC27.mb.123	92.89	0.000	38
Sample_SPC27.mb.90	98.51	0.671	65
Sample_SPC27.mb.69cbin.1	98.51	0.766	107
Sample_SPC28.Cluster11270	92.13	2.969	15
Sample_SPC28.mb.2	89.32	1.123	50
Sample_SPC28.Cluster11306	80.02	2.348	22

Sample_SPC28.Cluster483cbin.1	99.30	1.934	78
Sample_SPC28.mb.20	95.97	0.000	52
Sample_SPC28.Cluster10398	95.41	0.707	74
Sample_SPC28.mb.22	93.84	1.342	209
Sample_SPC28.Cluster3456cbin.1	96.83	0.021	88
Sample_SPC28.Cluster3153cbin.1	97.40	0.000	32
Sample_SPC28.Cluster8cbin.1	82.51	1.682	417
Sample_SPC28.Cluster753cbin.1	92.93	0.469	122
Sample_SPC28.Cluster5150	96.64	0.671	15
Sample_SPC28.Cluster9cbin.1	81.99	0.614	290
Sample_SPC28.Cluster5697cbin.1	89.93	1.342	31
Sample_SPC28.Cluster6428	98.65	0.671	31
Sample_SPC28.mb.24	99.32	0.671	26
Sample_SPC28.Cluster4642cbin.1	81.89	1.396	423
Sample_SPC28.Cluster965	98.63	0.227	37
Sample_SPC28.Cluster8548cbin.1	97.94	0.510	187
Sample_SPC28.mb.72cbin.1	97.98	0.000	49
Sample_SPC28.mb.92	97.98	0.671	41
Sample_SPC28.mb.67	95.19	1.682	122
Sample_SPC28.mb.33	95.16	0.000	106
Sample_SPC28.mb.40	93.63	1.980	192
Sample_SPC28.mb.9cbin.1	90.59	3.797	296
Sample_SPC28.mb.13	99.36	0.105	88
Sample_SPC28.mb.62cbin.1	98.65	0.000	17
Sample_SPC28.Cluster5040cbin.1	93.48	3.225	411
Sample_SPC28.mb.77	99.03	1.449	71
Sample_SPC28.mb.7cbin.1	97.10	2.548	73
Sample_SPC28.mb.89cbin.1	98.65	0.167	107
Sample_SPC28.mb.43cbin.1	91.49	2.540	214
Sample_SPC30.Cluster7389cbin.1	80.03	4.250	394
Sample_SPC30.Cluster1051	87.91	0.000	37
Sample_SPC30.Cluster3246	96.41	1.107	32
Sample_SPC30.mb.31	83.55	2.013	255
Sample_SPC30.Cluster236cbin.1	100.00	0.806	32
Sample_SPC30.mb.108cbin.1	90.22	3.683	191
Sample_SPC30.mb.100cbin.1	95.97	0.000	141
Sample_SPC30.mb.35	97.09	0.671	97
Sample_SPC30.Cluster4993cbin.1	93.58	2.994	293
Sample_SPC30.mb.24cbin.1	97.65	0.671	152
Sample_SPC30.Cluster7999cbin.1	98.65	0.000	33
Sample_SPC30.Cluster5293cbin.1	91.78	0.000	60
Sample_SPC30.mb.1	98.83	0.000	26
Sample_SPC30.mb.43cbin.1	94.46	0.671	89
Sample_SPC30.Cluster7400cbin.1	86.76	0.671	330
Sample_SPC30.Cluster5295cbin.1	96.95	1.576	87
Sample_SPC30.mb.48	80.06	0.949	130
Sample_SPC30.Cluster9176cbin.1	99.47	0.261	30
Sample_SPC30.Cluster5217cbin.1	98.62	3.692	57
Sample_SPC30.mb.87	82.75	0.000	103
Sample_SPC30.mb.13	96.15	0.561	34
Sample_SPC30.mb.73	96.78	1.165	78
Sample_SPC30.Cluster8831cbin.1	99.51	0.483	112
Sample_SPC30.mb.11cbin.1	95.97	1.446	27
Sample_SPC30.mb.41	94.21	2.267	239
Sample_SPC30.Cluster3797cbin.1	88.87	4.206	497
Sample_SPC30.mb.78	92.40	1.582	177
Sample_SPC30.mb.62	83.52	0.552	225
Sample_SPC30.mb.34	97.72	2.727	26

Sample_SPC30.mb.99	89.57	1.811	194
Sample_SPC30.Cluster6113cbin.1	99.36	0.949	100
Sample_SPC30.mb.85	91.98	1.201	25
Sample_SPC30.Cluster3074	99.20	0.076	108
Sample_SPC30.mb.71cbin.1	95.24	1.118	103
Sample_SPC30.mb.56cbin.1	95.99	1.285	197
Sample_SPC30.mb.82	98.65	0.335	39
Sample_SPC30.mb.75	99.32	0.000	21
Sample_SPC30.mb.52	85.92	0.000	556
Sample_SPC30.mb.68cbin.1	93.67	0.632	68
Sample_SPC30.mb.80cbin.1	89.57	1.119	108
Sample_SPC30.mb.74cbin.1	97.58	2.956	248
Sample_SPC31.Cluster13517	93.82	2.969	40
Sample_SPC31.mb.19	92.41	3.370	33
Sample_SPC31.Cluster5528cbin.1	93.93	1.342	30
Sample_SPC31.Cluster4761cbin.1	94.72	0.000	43
Sample_SPC31.Cluster7149	92.17	0.671	35
Sample_SPC31.mb.49	93.33	0.000	60
Sample_SPC31.Cluster557cbin.1	80.64	0.000	128
Sample_SPC31.Cluster7073cbin.1	88.22	0.111	348
Sample_SPC31.Cluster23cbin.1	82.46	3.040	656
Sample_SPC31.mb.36	85.79	1.845	172
Sample_SPC31.mb.64cbin.1	95.56	2.688	57
Sample_SPC31.mb.43cbin.1	95.07	3.147	138
Sample_SPC31.Cluster1	100.00	0.806	25
Sample_SPC31.mb.80cbin.1	94.73	2.551	257
Sample_SPC31.mb.33	94.05	3.266	146
Sample_SPC31.Cluster851cbin.1	91.94	1.908	128
Sample_SPC31.mb.5	80.18	1.342	208
Sample_SPC31.mb.76	80.16	2.097	43
Sample_SPC31.Cluster9043cbin.1	97.31	1.342	20
Sample_SPC31.Cluster3927	95.70	0.335	21
Sample_SPC31.Cluster456	99.11	0.000	46
Sample_SPC31.mb.100cbin.1	97.27	0.671	24
Sample_SPC31.mb.35cbin.1	85.61	4.720	351
Sample_SPC31.mb.60cbin.1	84.62	3.747	104
Sample_SPC31.mb.18cbin.1	95.74	0.061	27
Sample_SPC31.mb.65	93.17	0.671	90
Sample_SPC31.mb.42	95.50	3.020	130
Sample_SPC31.mb.58	83.33	0.480	148
Sample_SPC31.mb.83	88.59	0.671	25
Sample_SPC31.mb.39	96.61	1.449	45
Sample_SPC31.mb.9	84.06	1.459	57
Sample_SPC31.mb.103	99.24	0.000	20
Sample_SPC31.Cluster9271cbin.1	90.28	3.752	583
Sample_SPC31.mb.90	87.28	2.900	79
Sample_SPC31.mb.104	92.59	1.440	75
Sample_SPC31.mb.12cbin.1	98.75	2.884	183
Sample_SPC31.Cluster6989cbin.1	87.26	1.314	290
Sample_SPC31.mb.68cbin.1	100.00	0.089	41
Sample_SPC31.Cluster446	96.77	0.524	100
Sample_SPC32.mb.18cbin.1	96.77	0.000	48
Sample_SPC32.Cluster5908	83.19	0.020	245
Sample_SPC32.mb.23	97.65	0.000	32
Sample_SPC32.mb.51	85.23	0.335	30
Sample_SPC32.Cluster3302cbin.1	80.11	0.000	153
Sample_SPC32.mb.48cbin.1	95.30	0.000	52
Sample_SPC32.Cluster784	93.95	0.000	28

Sample_SPC32.Cluster14	98.65	0.671	63
Sample_SPC32.mb.3cbin.1	99.10	0.671	66
Sample_SPC32.Cluster4295	98.55	1.223	31
Sample_SPC32.mb.33cbin.1	97.67	1.265	58
Sample_SPC32.mb.68cbin.1	94.83	4.026	290
Sample_SPC32.mb.9cbin.1	96.83	3.263	325
Sample_SPC32.Cluster38	91.25	0.518	199
Sample_SPC32.Cluster5660cbin.1	99.32	0.000	21
Sample_SPC32.mb.34cbin.1	98.55	1.442	34
Sample_SPC32.Cluster329	96.87	0.000	35
Sample_SPC32.Cluster2799cbin.1	95.02	0.584	149
Sample_SPC32.Cluster6603cbin.1	98.94	1.056	207
Sample_SPC32.Cluster5317cbin.1	98.65	1.342	60
Sample_SPC32.mb.72cbin.1	91.82	0.480	30
Sample_SPC32.mb.75	97.98	0.000	18
Sample_SPC32.mb.35cbin.1	99.03	2.294	57
Sample_SPC32.Cluster1330cbin.1	96.44	2.307	237
Sample_SPC32.Cluster2341	98.65	0.576	34
Sample_SPC32.mb.43cbin.1	92.23	0.000	37
Sample_SPC32.mb.16cbin.1	91.13	1.461	103
Sample_SPC32.mb.55cbin.1	91.95	1.159	370
Sample_SPC34.Cluster218cbin.1	94.35	2.419	160
Sample_SPC34.Cluster6412cbin.1	93.93	1.342	23
Sample_SPC34.Cluster13592	88.88	0.854	18
Sample_SPC34.mb.16	83.62	3.448	55
Sample_SPC34.Cluster180	82.69	0.000	76
Sample_SPC34.Cluster784cbin.1	88.59	0.671	41
Sample_SPC34.mb.43	92.00	1.333	22
Sample_SPC34.mb.24cbin.1	97.90	1.165	118
Sample_SPC34.Cluster6988cbin.1	93.85	0.093	192
Sample_SPC34.mb.39	87.91	0.000	15
Sample_SPC34.Cluster3041	93.26	1.923	21
Sample_SPC34.mb.32	88.25	0.692	128
Sample_SPC34.Cluster11066cbin.1	99.32	1.006	22
Sample_SPC34.mb.102	97.58	0.806	44
Sample_SPC34.Cluster2616	97.95	0.680	26
Sample_SPC34.Cluster400	99.00	0.000	49
Sample_SPC34.mb.84cbin.1	97.58	0.806	44
Sample_SPC34.mb.28	95.48	0.496	178
Sample_SPC34.Cluster10388cbin.1	97.94	0.000	57
Sample_SPC34.mb.30	99.36	0.632	110
Sample_SPC34.mb.36	90.93	0.000	64
Sample_SPC34.Cluster8564cbin.1	95.34	2.001	71
Sample_SPC34.mb.21cbin.1	81.14	1.020	70
Sample_SPC34.mb.26cbin.1	92.82	0.671	159
Sample_SPC34.mb.61cbin.1	93.28	2.684	123
Sample_SPC34.mb.68	87.21	2.684	212
Sample_SPC34.mb.65	96.62	1.898	147
Sample_SPC34.mb.78cbin.1	95.16	0.000	36
Sample_SPC34.mb.76	96.87	0.603	64
Sample_SPC34.mb.87	99.51	0.000	29
Sample_SPC34.mb.74cbin.1	99.51	0.785	85
Sample_SPC34.mb.29	92.31	1.239	60
Sample_SPC34.mb.75	81.30	0.880	473
Sample_SPC34.mb.95cbin.1	93.02	4.567	172
Sample_SPC34.Cluster6455cbin.1	84.60	1.465	645
Sample_SPC34.mb.57	94.56	2.018	163
Sample_SPC35.Cluster4392cbin.1	97.84	0.097	41

Sample_SPC35.Cluster13613	80.16	2.797	25
Sample_SPC35.Cluster4020cbin.1	98.38	0.806	44
Sample_SPC35.Cluster6132	95.43	1.398	27
Sample_SPC35.mb.109cbin.1	91.01	2.808	16
Sample_SPC35.Cluster12386cbin.1	95.97	0.671	24
Sample_SPC35.Cluster8829	97.76	1.006	46
Sample_SPC35.mb.113cbin.1	89.16	0.699	35
Sample_SPC35.Cluster548cbin.1	87.73	4.423	107
Sample_SPC35.Cluster9504	96.64	0.000	55
Sample_SPC35.Cluster12742cbin.1	96.69	2.493	243
Sample_SPC35.Cluster8080cbin.1	98.99	0.396	42
Sample_SPC35.Cluster6587	95.45	1.594	104
Sample_SPC35.mb.29	88.59	1.454	180
Sample_SPC35.mb.31	97.60	0.000	32
Sample_SPC35.Cluster6966cbin.1	86.73	3.416	355
Sample_SPC35.mb.77cbin.1	95.69	3.360	176
Sample_SPC35.mb.10	94.35	1.612	118
Sample_SPC35.mb.64	84.70	0.671	201
Sample_SPC35.Cluster4cbin.1	92.57	2.484	295
Sample_SPC35.mb.80	81.21	3.030	16
Sample_SPC35.mb.92	91.01	1.123	24
Sample_SPC35.mb.74	80.02	4.068	22
Sample_SPC35.mb.41cbin.1	86.35	1.342	41
Sample_SPC35.mb.48cbin.1	98.62	2.056	200
Sample_SPC35.mb.63	91.47	3.467	88
Sample_SPC35.mb.13cbin.1	92.91	0.000	210
Sample_SPC35.mb.73cbin.1	97.20	2.013	197
Sample_SPC35.Cluster1269	97.35	0.480	25
Sample_SPC35.mb.112cbin.1	94.66	0.311	33
Sample_SPC35.mb.4cbin.1	93.23	1.280	27
Sample_SPC35.mb.101cbin.1	95.79	0.000	19
Sample_SPC35.mb.71	94.59	0.675	22
Sample_SPC35.mb.102	93.36	1.774	75
Sample_SPC35.mb.38cbin.1	98.75	0.937	52
Sample_SPC35.mb.65cbin.1	97.14	0.000	61
Sample_SPC35.Cluster7717	97.31	0.000	155
Sample_SPC35.mb.43cbin.1	89.59	4.138	282
Sample_SPC35.Cluster8961cbin.1	98.24	1.539	137
Sample_SPC35.mb.9	93.28	0.000	121
Sample_SPC35.mb.46cbin.1	96.88	0.943	63
Sample_SPC35.mb.88	100.00	0.000	19
Sample_SPC35.mb.115	95.28	0.000	86
Sample_SPC35.Cluster1343	98.80	0.074	30
Sample_SPC36.Cluster3901cbin.1	91.25	1.342	29
Sample_SPC36.Cluster9499	83.97	2.034	311
Sample_SPC36.mb.61	98.38	1.881	65
Sample_SPC36.mb.4	88.86	1.490	456
Sample_SPC36.Cluster635cbin.1	86.59	1.342	288
Sample_SPC36.Cluster303	80.39	2.013	54
Sample_SPC36.Cluster790cbin.1	95.97	1.342	51
Sample_SPC36.Cluster1804	85.90	0.671	96
Sample_SPC36.mb.63cbin.1	87.90	1.027	362
Sample_SPC36.mb.75	81.92	3.327	152
Sample_SPC36.Cluster5498cbin.1	98.02	2.115	178
Sample_SPC36.mb.35	93.63	2.947	165
Sample_SPC36.Cluster8252	99.32	0.671	19
Sample_SPC36.Cluster3019	96.37	0.000	28
Sample_SPC36.mb.62cbin.1	99.25	0.462	54

Sample_SPC36.Cluster1077cbin.1	96.36	0.454	25
Sample_SPC36.Cluster7181	99.32	0.000	32
Sample_SPC36.mb.20	99.32	0.671	21
Sample_SPC36.Cluster9568	98.65	0.335	19
Sample_SPC36.mb.58	97.94	0.000	105
Sample_SPC36.mb.60cbin.1	97.29	1.351	85
Sample_SPC36.mb.71	99.21	2.355	46
Sample_SPC36.mb.87cbin.1	100.00	0.632	53
Sample_SPC36.mb.80cbin.1	85.84	0.802	76
Sample_SPC37.Cluster13187	81.62	1.123	18
Sample_SPC37.Cluster13221	92.41	1.123	14
Sample_SPC37.Cluster13116	100.00	0.111	25
Sample_SPC37.Cluster4582cbin.1	98.38	0.806	35
Sample_SPC37.mb.1cbin.1	87.00	3.091	228
Sample_SPC37.Cluster7281	89.48	0.671	25
Sample_SPC37.Cluster100cbin.1	80.25	2.621	236
Sample_SPC37.mb.39cbin.1	87.29	3.655	347
Sample_SPC37.mb.42cbin.1	95.39	0.335	68
Sample_SPC37.Cluster450cbin.1	82.46	0.894	380
Sample_SPC37.mb.10	89.73	1.901	179
Sample_SPC37.mb.31	96.61	4.809	175
Sample_SPC37.mb.34	90.38	3.355	160
Sample_SPC37.Cluster4758	97.04	0.000	27
Sample_SPC37.mb.20	93.73	2.013	227
Sample_SPC37.mb.38	93.14	1.164	273
Sample_SPC37.Cluster9270cbin.1	97.09	0.000	27
Sample_SPC37.mb.21	91.05	0.000	69
Sample_SPC37.mb.57cbin.1	98.73	1.534	105
Sample_SPC37.Cluster9106	99.32	0.000	18
Sample_SPC37.mb.93	93.93	2.013	35
Sample_SPC37.mb.68cbin.1	82.99	0.671	91
Sample_SPC37.Cluster2120cbin.1	98.11	1.761	63
Sample_SPC37.Cluster7965cbin.1	80.84	2.651	380
Sample_SPC37.mb.61	84.29	1.342	232
Sample_SPC37.mb.60cbin.1	96.64	1.677	198
Sample_SPC37.mb.67	95.34	0.921	113
Sample_SPC37.mb.64	83.00	3.968	323
Sample_SPC37.mb.59	98.71	3.461	39
Sample_SPC37.Cluster9006cbin.1	87.48	4.584	465
Sample_SPC37.mb.91cbin.1	95.56	0.021	82
Sample_SPC37.mb.44	91.94	2.540	71
Sample_SPC37.mb.78	96.92	0.801	67
Sample_SPC37.mb.71	98.81	4.536	423
Sample_SPC37.mb.15cbin.1	93.14	1.870	477
Sample_SPC37.mb.25	95.94	0.563	162
Sample_SPC37.mb.9cbin.1	98.47	0.676	103
Sample_SPC37.mb.8	98.86	0.000	68
Sample_SPC37.mb.72	95.96	1.908	102
Sample_SPC37.mb.16cbin.1	99.45	2.370	275
Sample_SPC37.mb.63cbin.1	92.44	4.651	140
Sample_SPC38.Cluster9960cbin.1	96.30	2.852	275
Sample_SPC38.Cluster169	80.53	1.128	46
Sample_SPC38.mb.34	97.98	2.684	30
Sample_SPC38.mb.95	92.69	0.000	56
Sample_SPC38.mb.23	91.61	2.908	97
Sample_SPC38.mb.29	95.30	2.181	115
Sample_SPC38.mb.38cbin.1	90.54	1.744	264
Sample_SPC38.mb.36	94.90	1.128	147

Sample_SPC38.mb.81cbin.1	99.30	1.864	94
Sample_SPC38.Cluster3558	94.63	0.671	19
Sample_SPC38.Cluster8932	96.24	0.335	30
Sample_SPC38.mb.30	91.70	1.700	203
Sample_SPC38.mb.82	96.20	2.848	105
Sample_SPC38.mb.47	89.42	0.000	33
Sample_SPC38.Cluster128cbin.1	96.59	0.680	117
Sample_SPC38.mb.50	97.98	0.000	31
Sample_SPC38.mb.78	93.26	0.961	59
Sample_SPC38.mb.63	99.98	1.497	29
Sample_SPC38.Cluster5767cbin.1	95.56	2.531	93
Sample_SPC38.mb.12	93.96	2.512	27
Sample_SPC38.mb.51	99.32	0.000	22
Sample_SPC38.mb.43	91.43	2.201	304
Sample_SPC38.mb.39	93.03	0.791	112
Sample_SPC38.mb.16	97.15	0.000	58
Sample_SPC38.mb.11cbin.1	100.00	1.265	71
Sample_SPC38.mb.99cbin.1	95.36	0.000	46
Sample_SPC38.mb.69	91.69	1.132	15
Sample_SPC38.mb.9	97.94	0.000	42
Sample_SPC38.mb.68cbin.1	92.78	2.375	248
Sample_SPC38.mb.17	93.67	1.132	212
Sample_SPC38.mb.65cbin.1	85.22	4.093	81
Sample_SPC38.mb.42	97.29	2.106	64
Sample_SPC38.mb.89	96.02	0.384	72
Sample_SPC39.mb.28cbin.1	82.55	0.000	55
Sample_SPC39.mb.54	83.10	0.862	226
Sample_SPC39.Cluster11745cbin.1	95.41	1.230	179
Sample_SPC39.mb.37cbin.1	94.63	0.671	162
Sample_SPC39.Cluster7153cbin.1	98.94	2.163	35
Sample_SPC39.Cluster776cbin.1	95.27	0.675	61
Sample_SPC39.mb.15	82.88	0.000	132
Sample_SPC39.mb.11cbin.1	99.18	0.000	33
Sample_SPC39.Cluster5880cbin.1	98.73	0.021	91
Sample_SPC39.Cluster1686cbin.1	99.51	0.961	51
Sample_SPC39.mb.61cbin.1	93.95	1.927	173
Sample_SPC39.mb.52	90.60	0.671	56
Sample_SPC39.mb.33	87.50	2.043	234
Sample_SPC39.Cluster5759	97.98	0.671	14
Sample_SPC39.mb.71cbin.1	97.90	1.165	105
Sample_SPC39.mb.63cbin.1	90.81	0.223	46
Sample_SPC39.mb.102cbin.1	95.30	2.013	96
Sample_SPC39.Cluster256	97.33	0.000	57
Sample_SPC39.mb.27	98.15	3.131	128
Sample_SPC39.mb.56cbin.1	96.36	0.707	97
Sample_SPC39.Cluster11623cbin.1	99.47	3.141	52
Sample_SPC39.mb.21	98.65	0.335	41
Sample_SPC39.mb.5cbin.1	94.55	1.247	187
Sample_SPC39.Cluster9674cbin.1	97.98	0.920	71
Sample_SPC39.Cluster2073	99.65	0.057	38
Sample_SPC39.mb.78cbin.1	86.60	0.949	179
Sample_SPC39.Cluster9455cbin.1	99.51	4.290	78
Sample_SPC39.mb.74	97.26	0.000	35
Sample_SPC39.mb.104	87.32	1.323	354
Sample_SPC39.mb.79cbin.1	98.64	1.979	42
Sample_SPC39.Cluster5123	95.38	0.000	73
Sample_SPC39.mb.97	95.77	1.265	75
Sample_SPC41.Cluster13347	89.32	1.284	24

Sample_SPC41.Cluster13293cbin.1	91.57	2.487	28
Sample_SPC41.mb.18cbin.1	85.59	1.008	164
Sample_SPC41.Cluster6548	92.40	0.699	11
Sample_SPC41.mb.23	90.21	2.089	84
Sample_SPC41.mb.101cbin.1	84.21	1.006	245
Sample_SPC41.mb.20cbin.1	91.53	1.881	116
Sample_SPC41.mb.1	98.60	1.398	105
Sample_SPC41.mb.102	97.98	0.000	26
Sample_SPC41.mb.5	84.87	0.000	61
Sample_SPC41.mb.11	93.84	0.671	111
Sample_SPC41.mb.40	92.39	0.000	20
Sample_SPC41.mb.33	94.72	0.000	40
Sample_SPC41.mb.16cbin.1	99.32	1.677	50
Sample_SPC41.mb.69	97.71	1.612	135
Sample_SPC41.mb.26	90.60	4.026	43
Sample_SPC41.mb.68	90.58	3.494	294
Sample_SPC41.mb.87	90.32	0.000	23
Sample_SPC41.Cluster12539	95.81	0.800	192
Sample_SPC41.mb.83	91.27	0.671	42
Sample_SPC41.mb.100	94.47	2.572	188
Sample_SPC41.mb.44	88.78	3.205	266
Sample_SPC41.mb.28	96.64	0.000	122
Sample_SPC41.Cluster8115	97.09	0.000	22
Sample_SPC41.mb.91	93.06	4.295	341
Sample_SPC41.mb.95cbin.1	91.27	2.684	164
Sample_SPC41.mb.80cbin.1	99.90	0.000	70
Sample_SPC41.mb.94	95.70	1.006	183
Sample_SPC41.mb.85	97.31	0.134	51
Sample_SPC41.mb.98cbin.1	97.27	2.237	92
Sample_SPC41.mb.99cbin.1	98.11	0.903	358
Sample_SPC42.Cluster17162cbin.1	87.07	2.808	62
Sample_SPC42.mb.101cbin.1	94.35	0.000	34
Sample_SPC42.Cluster10367cbin.1	98.65	0.335	50
Sample_SPC42.mb.114	98.65	1.342	122
Sample_SPC42.Cluster7237	97.31	0.671	25
Sample_SPC42.Cluster76cbin.1	98.32	1.342	39
Sample_SPC42.mb.45	85.21	2.908	285
Sample_SPC42.mb.29cbin.1	97.41	2.750	36
Sample_SPC42.mb.41	92.55	0.671	25
Sample_SPC42.Cluster7381	94.27	1.449	38
Sample_SPC42.mb.67	82.93	0.000	257
Sample_SPC42.mb.68	98.65	0.000	39
Sample_SPC42.mb.112	84.64	1.863	308
Sample_SPC42.Cluster8949cbin.1	98.65	0.976	19
Sample_SPC42.Cluster13963cbin.1	99.34	2.123	112
Sample_SPC42.Cluster1138cbin.1	99.45	0.681	24
Sample_SPC42.Cluster10175	99.51	0.060	94
Sample_SPC42.mb.77	92.79	0.671	196
Sample_SPC42.mb.110	91.10	2.432	245
Sample_SPC42.mb.25	99.32	0.671	38
Sample_SPC42.mb.56	92.77	0.185	70
Sample_SPC42.mb.115cbin.1	95.28	4.026	209
Sample_SPC42.mb.73cbin.1	91.94	1.363	50
Sample_SPC42.mb.62cbin.1	93.10	0.000	76
Sample_SPC42.mb.54	93.54	1.686	101
Sample_SPC42.mb.7cbin.1	91.88	0.743	144
Sample_SPC42.mb.60cbin.1	92.11	3.586	133
Sample_SPC42.mb.28	94.67	0.724	63

Sample_SPC42.mb.83cbin.1	89.90	0.961	29
Sample_SPC42.mb.78cbin.1	94.42	3.252	117
Sample_SPC42.mb.81	98.11	0.000	87
Sample_SPC45.Cluster1174cbin.1	98.27	4.545	49
Sample_SPC45.Cluster1324cbin.1	99.30	1.165	79
Sample_SPC45.mb.21cbin.1	94.77	0.000	29
Sample_SPC45.mb.104	82.66	0.671	21
Sample_SPC45.mb.110	88.90	1.342	22
Sample_SPC45.mb.11	92.85	2.516	126
Sample_SPC45.mb.48cbin.1	97.17	1.075	55
Sample_SPC45.Cluster13128cbin.1	97.31	0.671	105
Sample_SPC45.Cluster2583	88.54	1.923	32
Sample_SPC45.Cluster7266	98.20	0.898	46
Sample_SPC45.mb.28cbin.1	85.01	0.000	45
Sample_SPC45.mb.46cbin.1	86.26	3.020	55
Sample_SPC45.mb.18	85.31	0.000	122
Sample_SPC45.Cluster4017cbin.1	82.07	0.377	85
Sample_SPC45.mb.7	98.93	0.806	54
Sample_SPC45.Cluster1974	97.95	0.226	49
Sample_SPC45.mb.1	95.13	4.474	126
Sample_SPC45.mb.65	93.49	1.342	160
Sample_SPC45.mb.67	94.05	1.398	92
Sample_SPC45.mb.91	90.65	0.806	121
Sample_SPC45.mb.25	95.30	2.013	113
Sample_SPC45.mb.22	95.08	2.334	66
Sample_SPC45.mb.77	87.96	2.300	151
Sample_SPC45.mb.49	96.93	0.000	14
Sample_SPC45.Cluster11681cbin.1	98.92	0.000	86
Sample_SPC45.mb.84	89.04	1.510	264
Sample_SPC45.mb.73cbin.1	94.77	0.335	39
Sample_SPC45.mb.2cbin.1	95.51	2.848	210
Sample_SPC45.mb.64	98.65	3.914	23
Sample_SPC45.mb.56cbin.1	94.44	0.671	181
Sample_SPC45.mb.99cbin.1	98.94	1.006	40
Sample_SPC45.mb.87	90.70	0.000	53
Sample_SPC45.mb.89	92.28	3.623	184
Sample_SPC45.Cluster6907cbin.1	98.21	4.213	69
Sample_SPC45.mb.98	85.40	2.237	256
Sample_SPC45.mb.17	98.84	0.000	51
Sample_SPC45.mb.88cbin.1	98.42	3.397	25
Sample_SPC45.mb.72cbin.1	87.40	0.961	508
Sample_SPC45.Cluster911cbin.1	98.94	0.404	190
Sample_SPC47.Cluster2599cbin.1	92.98	1.398	223
Sample_SPC47.Cluster3860cbin.1	91.38	0.284	167
Sample_SPC47.mb.11	93.42	4.250	212
Sample_SPC47.Cluster374cbin.1	93.95	2.034	102
Sample_SPC47.mb.31	87.24	0.000	55
Sample_SPC47.Cluster1897	97.31	0.000	11
Sample_SPC47.mb.10cbin.1	88.72	0.194	216
Sample_SPC47.mb.5	86.19	1.021	313
Sample_SPC47.mb.57cbin.1	92.69	0.632	164
Sample_SPC47.Cluster1045	98.75	0.625	25
Sample_SPC47.mb.43	93.05	4.873	222
Sample_SPC47.mb.29cbin.1	87.47	2.348	46
Sample_SPC47.mb.101cbin.1	97.27	2.045	15
Sample_SPC47.mb.21	97.31	0.000	49
Sample_SPC47.mb.61	84.64	1.121	312
Sample_SPC47.Cluster11991cbin.1	91.13	1.265	49

Sample_SPC47.mb.39cbin.1	97.12	3.725	174
Sample_SPC47.Cluster170cbin.1	91.23	0.630	194
Sample_SPC47.mb.73cbin.1	92.51	1.476	176
Sample_SPC47.Cluster9834cbin.1	99.51	3.910	70
Sample_SPC47.mb.77cbin.1	90.38	1.923	33
Sample_SPC47.mb.52	83.56	1.424	331
Sample_SPC47.mb.96cbin.1	91.27	1.635	84
Sample_SPC47.Cluster3686cbin.1	81.06	0.446	44
Sample_SPC47.mb.12	98.11	0.000	58
Sample_SPC47.mb.25	89.42	1.486	78
Sample_SPC47.Cluster6069cbin.1	99.46	0.017	69
Sample_SPC47.mb.66	92.42	1.242	76
Sample_SPC47.mb.18cbin.1	86.29	0.107	582
Sample_SPC47.Cluster3793	99.61	0.576	98
Sample_SPC47.mb.63	95.66	0.557	99
Sample_SPC47.Cluster5874cbin.1	96.98	0.384	310
Sample_SPC47.mb.84	97.65	4.932	289
Sample_SPC47.mb.76cbin.1	98.83	0.635	66
Sample_SPC47.mb.74	98.33	0.516	217
Sample_YPA1.mb.4cbin.1	97.85	2.019	153
Sample_YPA1.mb.7	98.56	0.680	45
Sample_YPA1.Cluster3	80.87	0.057	96
Sample_YPA1.Cluster770cbin.1	97.90	1.197	91
Sample_YPA1.mb.17	93.67	0.000	112
Sample_YPA1.Cluster2cbin.1	97.98	0.000	163
Sample_YPA1.Cluster14cbin.1	96.76	0.000	94
Sample_YPA1.Cluster736cbin.1	94.15	0.097	69
Sample_YPA1.Cluster346	91.44	0.123	41
Sample_YPA1.mb.5	88.97	0.000	591
Sample_YPA1.Cluster642cbin.1	97.33	0.188	51
Sample_YPA2.Cluster7617	81.54	2.237	192
Sample_YPA2.Cluster111	83.13	4.194	269
Sample_YPA2.Cluster444cbin.1	96.64	1.698	71
Sample_YPA2.mb.21	93.33	1.132	118
Sample_YPA2.mb.71	93.33	1.333	50
Sample_YPA2.Cluster967cbin.1	94.49	3.397	144
Sample_YPA2.mb.17cbin.1	92.29	3.059	166
Sample_YPA2.mb.22	83.32	1.336	299
Sample_YPA2.mb.31	95.63	0.000	137
Sample_YPA2.mb.69cbin.1	98.60	2.564	112
Sample_YPA2.mb.6	98.65	0.671	25
Sample_YPA2.mb.55	86.65	3.955	449
Sample_YPA2.mb.7	88.25	1.812	318
Sample_YPA2.Cluster964cbin.1	97.75	0.961	69
Sample_YPA2.Cluster10058	98.65	0.000	18
Sample_YPA2.Cluster9010cbin.1	97.98	1.006	14
Sample_YPA2.mb.61	93.98	1.265	115
Sample_YPA2.mb.26	96.25	0.185	63
Sample_YPA2.mb.23cbin.1	97.05	1.247	225
Sample_YPA2.mb.45cbin.1	98.60	2.544	170
Sample_YPA2.Cluster755	98.11	0.000	64
Sample_YPA2.Cluster6851cbin.1	91.09	0.000	39
Sample_YPA2.mb.15cbin.1	95.45	1.136	186
Sample_YPA2.Cluster7305	85.22	0.628	376
Sample_YPA2.mb.5	89.97	0.000	39
Sample_YPA2.Cluster8664cbin.1	98.65	0.183	47
Sample_YPA2.mb.18cbin.1	88.60	2.867	89
Sample_YPA2.mb.75	98.43	0.335	85

Sample_YPA2.mb.72cbin.1	98.71	0.573	75
Sample_YPA2.mb.37	99.42	0.000	44
Sample_YPA3.mb.22cbin.1	98.27	3.275	80
Sample_YPA3.Cluster4908	98.32	0.671	52
Sample_YPA3.mb.55	86.36	3.896	475
Sample_YPA3.mb.40cbin.1	82.94	2.740	418
Sample_YPA3.mb.54cbin.1	98.38	0.115	84
Sample_YPA3.mb.50	96.75	0.000	169
Sample_YPA3.mb.48cbin.1	90.60	3.691	64
Sample_YPA3.Cluster38cbin.1	98.32	0.805	42
Sample_YPA3.mb.61cbin.1	92.95	4.423	153
Sample_YPA3.Cluster4466	80.20	0.000	13
Sample_YPA3.Cluster2815cbin.1	90.78	0.000	41
Sample_YPA3.mb.66cbin.1	86.57	1.487	94
Sample_YPA3.mb.4	98.65	0.000	15
Sample_YPA3.Cluster806cbin.1	99.51	0.000	56
Sample_YPA3.Cluster5432	99.32	1.006	20
Sample_YPA3.mb.5cbin.1	99.03	0.961	33
Sample_YPA3.mb.52cbin.1	95.93	0.000	21
Sample_YPA3.mb.19cbin.1	94.63	0.671	85
Sample_YPA3.Cluster1349	96.64	4.047	95
Sample_YPA3.mb.68	96.00	3.061	74
Sample_YPA3.mb.58	93.98	1.257	169
Sample_YPA3.mb.46	98.44	0.805	265
Sample_YPA3.Cluster4437cbin.1	98.88	0.371	35
Sample_YPA3.Cluster3753	99.42	0.512	41
Sample_YPA3.mb.7	95.39	3.247	84
Sample_YPA4.mb.11cbin.1	95.97	1.398	134
Sample_YPA4.Cluster5859	97.98	2.237	46
Sample_YPA4.mb.19	98.43	0.000	101
Sample_YPA4.mb.30cbin.1	96.77	0.806	148
Sample_YPA4.Cluster452cbin.1	92.61	1.342	32
Sample_YPA4.mb.1	87.36	3.825	207
Sample_YPA4.Cluster139	90.60	2.745	47
Sample_YPA4.mb.22cbin.1	91.80	3.217	344
Sample_YPA4.mb.26	92.61	0.000	39
Sample_YPA4.Cluster45cbin.1	98.32	0.671	40
Sample_YPA4.mb.47	97.65	3.376	255
Sample_YPA4.Cluster5769cbin.1	88.60	0.021	45
Sample_YPA4.Cluster3321cbin.1	83.70	1.442	417
Sample_YPA4.Cluster7833	98.65	0.000	17
Sample_YPA4.Cluster3723cbin.1	96.66	0.231	42
Sample_YPA4.Cluster7082	99.32	1.006	20
Sample_YPA4.mb.50	94.21	3.018	166
Sample_YPA4.mb.52	90.86	0.961	26
Sample_YPA4.mb.46cbin.1	86.15	2.792	302
Sample_YPA4.mb.58	90.56	3.424	386
Sample_YPA4.mb.65cbin.1	94.63	1.342	91
Sample_YPA4.Cluster7499	98.65	0.000	74
Sample_YPA4.mb.70	91.90	0.000	20
Sample_YPA4.mb.43cbin.1	99.44	2.721	151
Sample_YPA4.mb.9cbin.1	94.63	0.671	82
Sample_YPA4.mb.63cbin.1	99.36	3.494	89
Sample_YPA4.mb.15cbin.1	99.25	0.185	40
Sample_YPA4.mb.64cbin.1	98.65	0.512	59
Sample_YPA5.Cluster28cbin.1	90.04	2.348	120
Sample_YPA5.Cluster1676cbin.1	84.25	0.740	219
Sample_YPA5.mb.19cbin.1	87.58	0.894	88

Sample_YPA5.mb.14	89.26	1.677	57
Sample_YPA5.mb.16	93.28	0.000	113
Sample_YPA5.Cluster7017	81.87	0.000	18
Sample_YPA5.mb.22cbin.1	98.99	0.000	44
Sample_YPA5.Cluster3216	98.02	1.250	37
Sample_YPA5.Cluster1060	98.75	0.625	22
Sample_YPA5.Cluster19	99.28	1.216	70
Sample_YPA5.mb.52cbin.1	90.93	1.251	49
Sample_YPA5.mb.23	98.07	0.961	145
Sample_YPA5.mb.69	96.64	1.342	58
Sample_YPA5.Cluster577cbin.1	89.11	0.641	50
Sample_YPA5.mb.39	97.48	2.028	101
Sample_YPA5.Cluster4724	98.55	0.000	56
Sample_YPA5.mb.29	94.63	0.155	180
Sample_YPA5.mb.13cbin.1	97.16	0.377	54
Sample_YPA5.mb.47cbin.1	99.36	0.632	106
Sample_YPA5.mb.8	95.65	0.801	40
Sample_YPA5.mb.7	99.32	0.305	20
Sample_YPA5.mb.81	87.25	3.071	49
Sample_YPA5.Cluster1993cbin.1	93.46	0.433	156
Sample_YPA5.mb.28	94.37	0.310	96
Sample_YPA5.mb.43	98.83	0.000	57
Sample_YPA5.mb.38cbin.1	95.98	4.195	289
Sample_YPA5.mb.68	93.71	0.724	42
Sample_YPA5.mb.30cbin.1	96.40	1.679	164
Sample_YPA5.mb.74cbin.1	96.64	1.887	251
Sample_YPA5.Cluster2191cbin.1	97.63	2.500	156
Sample_YPA5.Cluster3451cbin.1	98.65	0.384	64
Sample_YPA5.mb.40	92.74	1.654	51
Sample_YPA6.Cluster5179cbin.1	91.93	0.026	59
Sample_YPA6.mb.53	92.41	1.123	29
Sample_YPA6.Cluster7899	92.17	0.671	20
Sample_YPA6.Cluster81cbin.1	84.56	2.940	87
Sample_YPA6.mb.10	91.94	2.034	132
Sample_YPA6.mb.42	98.65	2.684	55
Sample_YPA6.Cluster3578cbin.1	98.65	0.671	28
Sample_YPA6.mb.63	88.06	2.348	214
Sample_YPA6.mb.13	97.27	0.000	21
Sample_YPA6.mb.47	95.01	3.174	375
Sample_YPA6.mb.60	96.61	1.690	60
Sample_YPA6.mb.43	93.77	0.000	150
Sample_YPA6.mb.28	88.58	2.125	420
Sample_YPA6.Cluster1155	99.03	0.480	21
Sample_YPA6.mb.61	98.73	2.415	52
Sample_YPA6.mb.27cbin.1	95.88	1.371	321
Sample_YPA6.mb.12cbin.1	98.92	1.612	143
Sample_YPA6.mb.34	95.00	0.867	33
Sample_YPA6.mb.24	96.92	0.000	47
Sample_YPA6.mb.58	93.36	4.022	130
Sample_YPA7.mb.29	93.82	2.969	51
Sample_YPA7.mb.45	88.20	1.123	77
Sample_YPA7.mb.19	80.02	1.677	19
Sample_YPA7.mb.18	95.30	2.237	167
Sample_YPA7.mb.32cbin.1	93.06	0.671	163
Sample_YPA7.Cluster910cbin.1	93.51	0.123	95
Sample_YPA7.Cluster251cbin.1	92.52	1.442	258
Sample_YPA7.mb.42	82.06	2.531	361
Sample_YPA7.mb.69	85.87	0.244	280

Sample_YPA7.Cluster3867cbin.1	97.98	0.335	16
Sample_YPA7.Cluster3122	96.98	2.295	16
Sample_YPA7.mb.16	93.16	1.360	254
Sample_YPA7.mb.24	99.32	1.006	66
Sample_YPA7.mb.31cbin.1	94.24	3.231	105
Sample_YPA7.Cluster628	97.95	0.000	38
Sample_YPA7.mb.26cbin.1	93.95	1.342	77
Sample_YPA7.mb.35	89.88	0.000	81
Sample_YPA7.mb.1	96.13	1.690	42
Sample_YPA7.mb.60cbin.1	97.27	0.356	155
Sample_YPA7.Cluster176cbin.1	98.31	0.480	22
Sample_YPA7.mb.9	90.60	1.006	35
Sample_YPA7.Cluster2391cbin.1	89.38	0.891	48
Sample_YPA7.mb.7	90.58	1.381	193
Sample_YPA7.mb.54	96.68	4.697	27
Sample_YPA7.mb.80	95.59	0.335	25
Sample_YPA7.mb.85	99.32	0.427	27
Sample_YPA7.mb.63cbin.1	99.32	2.083	97
Sample_YPA7.mb.81	99.11	0.030	63
Sample_YPA7.Cluster3591cbin.1	99.61	1.923	63
Sample_YPA7.Cluster7303cbin.1	93.26	1.034	566
Sample_YPA7.Cluster2095	99.03	0.384	88
Sample_YPA7.mb.64	80.42	4.951	307
Sample_YPA7.mb.76	88.39	0.929	97
Sample_YPA7.mb.22cbin.1	95.98	2.464	151
Sample_YPA7.mb.67	98.06	2.777	163
Sample_YPA7.mb.62	97.87	0.805	56
Sample_YPB1.Cluster1591	88.59	0.000	40
Sample_YPB1.mb.28cbin.1	83.55	0.793	38
Sample_YPB1.Cluster327cbin.1	92.61	0.000	24
Sample_YPB1.Cluster1720	99.18	0.000	18
Sample_YPB1.Cluster198cbin.1	98.41	0.680	228
Sample_YPB1.Cluster782cbin.1	96.83	0.021	74
Sample_YPB1.Cluster708	94.63	0.000	10
Sample_YPB1.Cluster3867	99.32	0.671	19
Sample_YPB1.mb.77	100.00	2.013	306
Sample_YPB1.Cluster425cbin.1	85.68	0.454	17
Sample_YPB1.mb.6cbin.1	98.38	0.806	35
Sample_YPB1.mb.16	90.58	0.340	81
Sample_YPB1.mb.21	99.53	0.460	28
Sample_YPB1.mb.39	99.11	0.000	30
Sample_YPB1.Cluster447cbin.1	98.31	0.000	73
Sample_YPB1.mb.23	92.85	0.552	58
Sample_YPB1.mb.59cbin.1	95.37	2.721	116
Sample_YPB1.mb.38	96.23	0.000	31
Sample_YPB1.mb.8cbin.1	96.87	0.000	36
Sample_YPB1.mb.17cbin.1	96.45	1.756	108
Sample_YPB1.Cluster1122cbin.1	99.09	0.724	45
Sample_YPB1.mb.36	94.36	1.424	146
Sample_YPB1.mb.49cbin.1	99.27	2.415	63
Sample_YPB1.Cluster2259cbin.1	94.42	0.537	448
Sample_YPB1.Cluster1036cbin.1	93.68	0.991	82
Sample_YPB1.mb.32	96.60	1.132	228
Sample_YPB1.mb.44cbin.1	97.73	1.537	98
Sample_YPB2.Cluster12916	84.55	1.123	14
Sample_YPB2.Cluster12477	100.00	0.111	20
Sample_YPB2.Cluster11555	98.65	0.000	8
Sample_YPB2.Cluster5718cbin.1	95.01	3.041	208

Sample_YPB2.mb.48	92.69	3.932	100
Sample_YPB2.mb.21	85.89	2.516	315
Sample_YPB2.Cluster87	85.90	0.671	75
Sample_YPB2.Cluster2520	82.01	0.000	15
Sample_YPB2.mb.15	87.29	3.020	101
Sample_YPB2.mb.24cbin.1	88.92	0.671	56
Sample_YPB2.Cluster9399cbin.1	99.03	1.223	28
Sample_YPB2.mb.32cbin.1	98.94	0.632	45
Sample_YPB2.Cluster1278	98.75	0.625	30
Sample_YPB2.mb.4cbin.1	94.63	3.327	646
Sample_YPB2.mb.5cbin.1	96.43	2.214	92
Sample_YPB2.Cluster1528cbin.1	98.52	1.442	76
Sample_YPB2.mb.93cbin.1	92.93	1.342	28
Sample_YPB2.Cluster9659	99.32	1.006	14
Sample_YPB2.mb.56	98.99	0.000	40
Sample_YPB2.mb.1	95.90	2.631	101
Sample_YPB2.mb.6	90.75	2.403	218
Sample_YPB2.mb.58cbin.1	95.87	1.360	269
Sample_YPB2.mb.73	95.96	1.209	115
Sample_YPB2.mb.27cbin.1	96.63	4.347	106
Sample_YPB2.mb.74	95.56	0.000	122
Sample_YPB2.mb.83	95.89	2.173	42
Sample_YPB2.mb.67cbin.1	100.00	0.227	17
Sample_YPB2.mb.69	82.27	1.107	44
Sample_YPB2.mb.72	94.82	4.057	182
Sample_YPB2.Cluster3860	97.69	0.000	107
Sample_YPB2.mb.75cbin.1	97.12	4.375	250
Sample_YPB2.Cluster947	99.67	0.773	33
Sample_YPB3.Cluster3030cbin.1	88.57	3.187	368
Sample_YPB3.mb.31	80.87	0.671	352
Sample_YPB3.mb.59	97.58	2.688	148
Sample_YPB3.mb.47cbin.1	94.07	0.000	159
Sample_YPB3.Cluster1cbin.1	85.48	0.685	19
Sample_YPB3.Cluster1244	90.60	0.000	23
Sample_YPB3.Cluster2914	99.18	0.000	23
Sample_YPB3.mb.39cbin.1	98.31	1.898	121
Sample_YPB3.mb.8	90.71	3.976	278
Sample_YPB3.mb.20	93.26	0.844	244
Sample_YPB3.Cluster4065	91.27	1.006	14
Sample_YPB3.mb.26	95.50	0.680	246
Sample_YPB3.Cluster145cbin.1	96.60	1.006	51
Sample_YPB3.Cluster33cbin.1	85.94	1.152	56
Sample_YPB3.mb.40	83.61	1.489	284
Sample_YPB3.Cluster1000	98.78	0.598	24
Sample_YPB3.Cluster867cbin.1	95.80	0.598	84
Sample_YPB3.Cluster1114cbin.1	90.00	1.250	18
Sample_YPB3.Cluster198	94.51	2.163	157
Sample_YPB3.Cluster2149cbin.1	90.54	0.619	61
Sample_YPB3.Cluster2564	95.91	0.024	36
Sample_YPB4.Cluster6185	93.25	1.284	12
Sample_YPB4.mb.26cbin.1	87.63	1.174	353
Sample_YPB4.Cluster2405	94.63	0.000	25
Sample_YPB4.Cluster2143	98.65	0.671	35
Sample_YPB4.Cluster3798	93.67	1.582	50
Sample_YPB4.mb.5cbin.1	95.88	3.797	206
Sample_YPB4.Cluster1733	88.31	0.000	24
Sample_YPB4.mb.68	97.67	1.707	66
Sample_YPB4.mb.8cbin.1	95.30	0.000	28

Sample_YPB4.Cluster16	92.51	4.081	39
Sample_YPB4.Cluster60cbin.1	97.73	0.816	230
Sample_YPB4.Cluster342	96.46	0.000	52
Sample_YPB4.mb.62	83.84	2.923	149
Sample_YPB4.mb.43cbin.1	85.70	1.434	407
Sample_YPB4.Cluster4709cbin.1	97.98	0.000	21
Sample_YPB4.mb.19cbin.1	95.19	0.598	30
Sample_YPB4.Cluster3580	98.65	1.006	12
Sample_YPB4.mb.30cbin.1	89.22	0.000	78
Sample_YPB4.mb.34	99.22	0.000	10
Sample_YPB4.Cluster5996	99.47	0.000	27
Sample_YPB4.mb.29cbin.1	93.95	2.268	174
Sample_YPB4.Cluster170	100.00	0.681	12
Sample_YPB4.mb.60cbin.1	99.94	0.460	58
Sample_YPB4.mb.55	87.93	2.898	275
Sample_YPB4.mb.51cbin.1	93.58	0.619	62
Sample_YPB4.Cluster1428	97.76	2.602	34
Sample_YPB4.mb.13	96.73	4.967	398
Sample_YPB4.mb.11cbin.1	94.54	3.822	256
Sample_YPB5.Cluster8314cbin.1	88.00	1.333	152
Sample_YPB5.Cluster5620cbin.1	83.75	3.803	357
Sample_YPB5.Cluster3185cbin.1	88.46	0.020	154
Sample_YPB5.mb.25cbin.1	82.48	0.134	315
Sample_YPB5.mb.30cbin.1	80.77	0.815	72
Sample_YPB5.mb.42	94.63	0.671	31
Sample_YPB5.Cluster5686	94.92	0.801	37
Sample_YPB5.mb.19cbin.1	98.65	0.671	55
Sample_YPB5.mb.24cbin.1	97.98	0.859	85
Sample_YPB5.Cluster488cbin.1	97.37	0.806	110
Sample_YPB5.Cluster5204cbin.1	95.30	0.335	26
Sample_YPB5.mb.65cbin.1	92.87	0.073	192
Sample_YPB5.Cluster2663cbin.1	86.24	2.565	352
Sample_YPB5.Cluster1628cbin.1	98.33	0.185	47
Sample_YPB5.Cluster5889cbin.1	98.65	1.118	41
Sample_YPB5.mb.36cbin.1	88.08	1.901	235
Sample_YPB5.mb.75	93.93	1.342	32
Sample_YPB5.mb.56	97.42	1.342	95
Sample_YPB5.Cluster1199cbin.1	99.90	0.632	103
Sample_YPB5.Cluster4488cbin.1	97.58	2.415	86
Sample_YPB5.mb.23	91.08	1.984	269
Sample_YPB5.mb.57	89.26	1.405	32
Sample_YPB5.Cluster400cbin.1	96.56	0.625	99
Sample_YPB5.mb.5	98.07	0.961	35
Sample_YPB5.Cluster1691cbin.1	94.90	0.377	50
Sample_YPB5.mb.54cbin.1	98.99	0.000	42
Sample_YPB5.mb.63cbin.1	95.56	3.797	196
Sample_YPB5.Cluster7289cbin.1	98.65	0.000	49
Sample_YPB5.mb.38	84.05	0.483	46
Sample_YPB5.mb.46	93.41	0.223	130
Sample_YPB5.mb.33	90.53	0.483	206
Sample_YPB5.mb.74	94.63	0.000	18
Sample_YPB5.mb.47	84.70	0.373	191
Sample_YPB5.mb.78	96.64	0.671	115
Sample_YPB5.Cluster6949cbin.1	92.90	1.281	198
Sample_YPB5.Cluster2441cbin.1	95.19	0.743	91
Sample_YPB5.mb.9	99.36	0.000	65
Sample_YPB5.mb.40	95.16	2.416	44
Sample_YPB6.Cluster2046cbin.1	100.00	3.667	58

Sample_YPB6.mb.27	88.92	1.165	437
Sample_YPB6.mb.42cbin.1	96.64	0.000	130
Sample_YPB6.mb.23cbin.1	88.71	4.861	283
Sample_YPB6.mb.37cbin.1	98.32	0.000	68
Sample_YPB6.mb.30cbin.1	82.28	2.040	417
Sample_YPB6.mb.47cbin.1	92.83	1.975	60
Sample_YPB6.mb.2	98.01	0.000	85
Sample_YPB6.Cluster3534	99.32	0.671	14
Sample_YPB6.mb.12	85.12	3.200	705
Sample_YPB6.mb.13cbin.1	96.76	0.105	212
Sample_YPB6.Cluster3334cbin.1	97.26	0.839	74
Sample_YPB6.mb.24	80.35	0.495	55
Sample_YPB6.Cluster1198cbin.1	99.15	1.582	125
Sample_YPB6.Cluster1316cbin.1	98.70	0.000	42
Sample_YPB6.Cluster2752cbin.1	95.44	4.125	115
Sample_YPB6.mb.51	95.26	1.437	139
Sample_YPB7.Cluster12975	87.92	0.561	39
Sample_YPB7.Cluster12994	94.66	2.666	22
Sample_YPB7.Cluster1523cbin.1	82.07	1.075	241
Sample_YPB7.mb.11	96.77	0.000	47
Sample_YPB7.Cluster6653	91.49	0.671	40
Sample_YPB7.Cluster613	93.28	1.342	49
Sample_YPB7.mb.12	88.42	4.484	197
Sample_YPB7.Cluster244cbin.1	91.77	4.276	296
Sample_YPB7.Cluster3668cbin.1	94.63	4.138	55
Sample_YPB7.mb.33	89.55	2.957	287
Sample_YPB7.Cluster5070cbin.1	96.60	0.671	23
Sample_YPB7.mb.34	91.97	0.134	137
Sample_YPB7.mb.39	92.97	1.360	341
Sample_YPB7.mb.28	94.15	1.461	58
Sample_YPB7.mb.69	93.95	0.335	37
Sample_YPB7.mb.7cbin.1	95.06	1.587	253
Sample_YPB7.mb.29	93.31	0.632	291
Sample_YPB7.Cluster1361	99.03	0.961	20
Sample_YPB7.mb.23	98.86	0.000	109
Sample_YPB7.mb.76	96.92	1.870	78
Sample_YPB7.Cluster10119cbin.1	83.35	0.384	329
Sample_YPB7.mb.50cbin.1	97.84	0.537	146
Sample_YPB7.mb.40cbin.1	94.81	1.425	189
Sample_YPB7.mb.20	93.35	3.358	139
Sample_YPB7.mb.67	97.88	0.000	46
Sample_YPB7.Cluster8264cbin.1	99.63	0.753	84
Sample_YPB7.mb.65cbin.1	99.63	0.181	54
Sample_YPC1.Cluster3916	93.25	1.284	2
Sample_YPC1.Cluster2324cbin.1	87.18	0.949	42
Sample_YPC1.mb.55	97.50	1.700	129
Sample_YPC1.Cluster838	93.28	0.000	78
Sample_YPC1.mb.52cbin.1	90.93	0.000	13
Sample_YPC1.Cluster22cbin.1	91.94	2.684	27
Sample_YPC1.Cluster992cbin.1	98.94	1.265	34
Sample_YPC1.mb.58	89.93	0.000	84
Sample_YPC1.mb.45	96.77	0.806	15
Sample_YPC1.Cluster62cbin.1	88.43	0.000	33
Sample_YPC1.mb.63cbin.1	91.29	0.632	56
Sample_YPC1.Cluster479cbin.1	98.10	0.000	57
Sample_YPC1.Cluster214cbin.1	97.97	0.340	58
Sample_YPC1.Cluster1720	99.32	1.006	18
Sample_YPC1.mb.51	97.19	0.641	109

Sample_YPC1.mb.19cbin.1	88.80	0.681	42
Sample_YPC1.Cluster77	96.87	0.000	10
Sample_YPC1.Cluster10cbin.1	99.36	0.742	65
Sample_YPC1.Cluster1799	96.57	0.000	29
Sample_YPC1.mb.33	99.57	2.345	57
Sample_YPC1.mb.32	86.88	1.707	171
Sample_YPC1.Cluster660cbin.1	97.46	0.000	80
Sample_YPC1.mb.49	99.32	0.134	145
Sample_YPC1.mb.13	99.61	0.000	42
Sample_YPC1.mb.4cbin.1	95.53	0.123	64
Sample_YPC2.Cluster12313	92.69	4.494	22
Sample_YPC2.Cluster11916	97.33	0.111	42
Sample_YPC2.Cluster2866cbin.1	95.96	0.806	37
Sample_YPC2.Cluster11967cbin.1	88.81	0.000	11
Sample_YPC2.mb.2	90.49	0.671	145
Sample_YPC2.Cluster314	87.71	1.258	88
Sample_YPC2.mb.4	84.34	1.597	190
Sample_YPC2.mb.72	88.20	1.685	121
Sample_YPC2.mb.58	99.30	1.165	143
Sample_YPC2.mb.28	90.32	0.000	69
Sample_YPC2.mb.45	91.27	1.525	48
Sample_YPC2.mb.42cbin.1	97.04	1.956	45
Sample_YPC2.mb.11	92.61	0.000	10
Sample_YPC2.Cluster623cbin.1	97.73	0.680	241
Sample_YPC2.mb.21	93.91	2.403	78
Sample_YPC2.mb.49cbin.1	93.73	1.565	286
Sample_YPC2.Cluster2726cbin.1	97.27	1.006	34
Sample_YPC2.Cluster9181	99.32	1.677	15
Sample_YPC2.mb.10	95.23	0.000	28
Sample_YPC2.mb.66	93.28	0.041	25
Sample_YPC2.Cluster1309	95.09	0.000	54
Sample_YPC2.Cluster752cbin.1	94.82	0.946	145
Sample_YPC2.mb.39cbin.1	98.18	2.894	132
Sample_YPC2.mb.77	96.64	1.610	57
Sample_YPC2.mb.25cbin.1	92.61	2.125	288
Sample_YPC2.mb.9cbin.1	86.12	0.671	111
Sample_YPC2.mb.31	95.52	2.572	230
Sample_YPC2.Cluster11303cbin.1	99.51	2.173	86
Sample_YPC2.Cluster10628	95.84	0.000	74
Sample_YPC2.mb.54cbin.1	91.09	0.000	40
Sample_YPC2.Cluster6818cbin.1	99.62	0.000	19
Sample_YPC2.mb.59cbin.1	92.12	0.632	98
Sample_YPC2.Cluster5220cbin.1	95.76	0.000	32
Sample_YPC2.Cluster7809cbin.1	97.49	4.306	85
Sample_YPC2.Cluster5530	99.23	0.000	41
Sample_YPC3.mb.11	82.05	2.963	449
Sample_YPC3.Cluster277	99.19	0.806	18
Sample_YPC3.mb.16	98.43	0.000	88
Sample_YPC3.Cluster3714cbin.1	94.51	1.054	51
Sample_YPC3.Cluster3877	99.18	0.000	24
Sample_YPC3.mb.1	97.29	1.698	259
Sample_YPC3.Cluster4271cbin.1	97.46	1.265	56
Sample_YPC3.Cluster65	89.11	0.000	36
Sample_YPC3.Cluster333cbin.1	94.55	0.680	226
Sample_YPC3.Cluster3516	99.38	1.497	44
Sample_YPC3.Cluster1980cbin.1	96.51	0.000	52
Sample_YPC3.Cluster5558	98.65	1.006	11
Sample_YPC3.Cluster1901cbin.1	96.38	0.170	67

Sample_YPC3.mb.47cbin.1	96.05	0.748	190
Sample_YPC3.mb.3	99.22	0.000	11
Sample_YPC3.mb.37cbin.1	89.10	2.404	285
Sample_YPC3.Cluster19	99.53	0.153	37
Sample_YPC3.Cluster1253	100.00	0.227	14
Sample_YPC3.mb.19	93.50	1.469	27
Sample_YPC3.mb.50	98.63	0.000	26
Sample_YPC3.mb.45cbin.1	97.34	4.823	85
Sample_YPC3.mb.23	91.53	3.375	305
Sample_YPC3.Cluster3672cbin.1	99.61	1.858	59
Sample_YPC3.mb.13cbin.1	98.14	0.836	44
Sample_YPC4.Cluster7056	97.31	0.732	53
Sample_YPC4.Cluster2418cbin.1	97.98	0.230	99
Sample_YPC4.Cluster3903cbin.1	84.11	3.164	399
Sample_YPC4.Cluster699	85.90	1.447	31
Sample_YPC4.Cluster795cbin.1	95.74	1.006	127
Sample_YPC4.Cluster448cbin.1	88.59	1.677	51
Sample_YPC4.mb.52	88.40	3.752	246
Sample_YPC4.mb.71cbin.1	85.82	1.565	313
Sample_YPC4.mb.12cbin.1	94.95	0.000	80
Sample_YPC4.Cluster4622cbin.1	98.88	0.231	47
Sample_YPC4.mb.27	91.90	0.000	17
Sample_YPC4.Cluster8779	96.97	0.000	18
Sample_YPC4.Cluster8162	94.63	1.677	23
Sample_YPC4.mb.65cbin.1	83.89	2.013	83
Sample_YPC4.mb.30	100.00	0.961	64
Sample_YPC4.mb.7cbin.1	94.96	4.138	51
Sample_YPC4.mb.15cbin.1	94.42	0.671	66
Sample_YPC4.Cluster8636cbin.1	98.65	1.006	150
Sample_YPC4.mb.36cbin.1	94.57	2.585	164
Sample_YPC4.mb.80	90.86	0.961	25
Sample_YPC4.mb.35	88.39	1.864	578
Sample_YPC4.mb.47cbin.1	94.73	1.754	68
Sample_YPC4.mb.74cbin.1	97.09	2.210	138
Sample_YPC4.Cluster4564cbin.1	97.66	3.411	227
Sample_YPC4.mb.83cbin.1	97.39	1.486	46
Sample_YPC4.mb.9cbin.1	99.42	0.512	43
Sample_YPC5.Cluster10878	91.57	1.123	9
Sample_YPC5.Cluster1749cbin.1	98.38	0.000	53
Sample_YPC5.Cluster3557	92.93	1.342	26
Sample_YPC5.mb.27cbin.1	98.65	0.000	12
Sample_YPC5.Cluster10250cbin.1	99.32	1.677	44
Sample_YPC5.Cluster149cbin.1	95.30	0.000	42
Sample_YPC5.mb.56cbin.1	97.90	1.165	94
Sample_YPC5.Cluster480cbin.1	92.61	2.013	49
Sample_YPC5.Cluster11cbin.1	84.89	1.342	46
Sample_YPC5.mb.31	90.77	1.174	104
Sample_YPC5.mb.24	89.93	2.684	227
Sample_YPC5.Cluster5474cbin.1	98.99	0.000	50
Sample_YPC5.Cluster3768cbin.1	98.02	1.250	44
Sample_YPC5.mb.38cbin.1	96.25	1.360	215
Sample_YPC5.Cluster3cbin.1	89.73	0.898	292
Sample_YPC5.Cluster12cbin.1	93.22	1.216	269
Sample_YPC5.mb.3	99.51	4.214	74
Sample_YPC5.mb.53	98.65	0.335	47
Sample_YPC5.mb.36	98.65	0.964	34
Sample_YPC5.mb.85	91.27	0.671	54
Sample_YPC5.mb.51cbin.1	100.00	0.632	109

Sample_YPC5.mb.80	89.56	2.013	220
Sample_YPC5.mb.37cbin.1	97.31	0.671	47
Sample_YPC5.mb.25cbin.1	98.64	0.966	108
Sample_YPC5.mb.69	99.03	0.747	38
Sample_YPC5.Cluster7905cbin.1	97.66	0.974	49
Sample_YPC5.mb.79cbin.1	97.27	0.671	28
Sample_YPC5.mb.48	90.09	1.741	148
Sample_YPC5.mb.84cbin.1	99.32	1.006	40
Sample_YPC5.Cluster5166cbin.1	98.92	0.000	140
Sample_YPC5.mb.62	86.74	0.000	104
Sample_YPC5.Cluster2626	90.54	1.185	202
Sample_YPC5.mb.73cbin.1	94.30	0.949	77
Sample_YPC5.Cluster4418cbin.1	94.63	3.469	71
Sample_YPC5.mb.70	99.03	0.384	57
Sample_YPC6.Cluster7961	93.62	0.000	34
Sample_YPC6.Cluster8978cbin.1	95.80	2.013	64
Sample_YPC6.mb.26	86.57	2.013	38
Sample_YPC6.Cluster445	86.57	2.318	68
Sample_YPC6.Cluster10275	85.53	0.094	274
Sample_YPC6.Cluster206	86.46	1.363	233
Sample_YPC6.mb.44cbin.1	80.31	1.062	101
Sample_YPC6.mb.66	93.30	2.237	179
Sample_YPC6.mb.19cbin.1	89.86	1.251	264
Sample_YPC6.mb.12	89.58	0.782	214
Sample_YPC6.mb.69	85.23	0.167	110
Sample_YPC6.mb.5	100.00	1.612	41
Sample_YPC6.mb.21	88.74	2.742	352
Sample_YPC6.Cluster4134cbin.1	87.47	2.013	22
Sample_YPC6.mb.15	93.96	1.488	220
Sample_YPC6.mb.56	98.87	0.721	157
Sample_YPC6.mb.71	96.69	0.598	69
Sample_YPC6.mb.67	98.53	0.584	75
Sample_YPC6.Cluster781	98.31	0.667	27
Sample_YPC6.mb.57cbin.1	91.08	0.738	145
Sample_YPC6.mb.68cbin.1	97.84	0.000	149
Sample_YPC6.mb.41	91.40	2.168	140
Sample_YPC6.mb.42cbin.1	94.36	3.289	150
Sample_YPC6.mb.50	99.42	0.000	57
Sample_YPC6.mb.9	98.34	2.425	70
Sample_YPC6.Cluster2250cbin.1	99.96	0.597	70
Sample_YPC7.mb.15	83.40	1.573	331
Sample_YPC7.mb.10	95.99	1.342	41
Sample_YPC7.mb.23	91.27	0.000	45
Sample_YPC7.Cluster3550cbin.1	98.32	0.671	36
Sample_YPC7.mb.63	96.29	1.817	92
Sample_YPC7.Cluster1606cbin.1	94.63	2.684	89
Sample_YPC7.Cluster3409cbin.1	94.93	0.021	68
Sample_YPC7.Cluster8892	98.65	0.000	20
Sample_YPC7.Cluster3787cbin.1	93.91	0.000	19
Sample_YPC7.mb.3	80.20	0.671	16
Sample_YPC7.mb.81	99.10	1.342	132
Sample_YPC7.Cluster7783cbin.1	82.86	2.818	458
Sample_YPC7.mb.34cbin.1	99.03	0.961	31
Sample_YPC7.mb.60cbin.1	98.07	0.000	156
Sample_YPC7.mb.42	94.63	0.671	70
Sample_YPC7.mb.78cbin.1	95.18	0.046	44
Sample_YPC7.Cluster4349cbin.1	80.11	2.021	599
Sample_YPC7.Cluster5553cbin.1	91.03	2.877	230

Sample_YPC7.mb.6cbin.1	98.24	0.877	71
Sample_YPC7.mb.18cbin.1	95.90	0.464	43
Sample_YPC7.Cluster5024	94.80	0.897	56
Sample_YPC7.mb.27	98.10	0.593	91
Sample_YPC7.mb.40	98.99	0.000	38

MAG ID	GC content (%)	Checkm lineage	N50 length (bp)	Genome size (Mbp)
Sample_LCA09.Cluster12306	26.00	Bacteria	307090	1297850
Sample_LCA09.Cluster1389cbin.1	53.10	Clostridia	26776	1949356
Sample_LCA09.Cluster103	61.90	Clostridiales	49600	1588306
Sample_LCA09.Cluster29cbin.1	61.10	Clostridiales	8134	1657156
Sample_LCA09.Cluster9849cbin.1	37.30	Clostridiales	9043	2292989
Sample_LCA09.Cluster3712cbin.1	48.10	Clostridiales	80166	2883383
Sample_LCA09.Cluster3578	45.80	Clostridiales	128157	2234929
Sample_LCA09.mb.109	32.40	Clostridiales	44742	2847374
Sample_LCA09.mb.101cbin.1	60.50	Clostridiales	63120	2399601
Sample_LCA09.Cluster1791	53.80	Clostridiales	211691	1840564
Sample_LCA09.mb.39cbin.1	54.00	Clostridia	7362	1499545
Sample_LCA09.mb.16cbin.1	38.20	Clostridiales	50808	3483555
Sample_LCA09.Cluster770cbin.1	60.00	Bacteroidetes	10290	2578749
Sample_LCA09.Cluster11946cbin.1	32.50	Lactobacillales	129011	1843270
Sample_LCA09.mb.10	56.30	Clostridiales	16026	2354496
Sample_LCA09.Cluster9301	40.90	Clostridiales	236790	2054985
Sample_LCA09.mb.2cbin.1	56.50	Clostridiales	52298	2667750
Sample_LCA09.Cluster11194cbin.1	37.30	Clostridiales	100999	2756574
Sample_LCA09.Cluster961	56.30	Bifidobacteriaceae	170022	2019947
Sample_LCA09.mb.53cbin.1	49.70	Clostridiales	85895	2240957
Sample_LCA09.mb.127	60.60	Clostridiales	45516	2199270
Sample_LCA09.Cluster74cbin.1	59.60	Bifidobacteriaceae	97278	2687162
Sample_LCA09.mb.73	38.30	Clostridiales	22462	2645003
Sample_LCA09.mb.103	43.40	Lachnospiraceae	89187	2914268
Sample_LCA09.mb.15cbin.1	51.30	Clostridiales	199093	2301910
Sample_LCA09.mb.45	49.00	Lachnospiraceae	12270	2725822
Sample_LCA09.mb.75	36.30	Clostridiales	43467	2501757
Sample_LCA09.mb.17cbin.1	42.70	Clostridiales	131371	2671046
Sample_LCA09.mb.106cbin.1	57.70	Bacteroidetes	168890	3199532
Sample_LCA09.mb.66	49.30	Clostridiales	19751	2250645
Sample_LCA09.mb.79	56.80	Clostridiales	60652	2448695
Sample_LCA09.mb.82cbin.1	57.00	Clostridiales	68863	1850825
Sample_LCA09.Cluster3917	44.70	Bacteroidales	145171	3832313
Sample_LCA09.mb.31cbin.1	40.50	Clostridiales	107751	2708740
Sample_LCA09.mb.99	62.30	Clostridiales	25167	1865030
Sample_LCA09.Cluster1997	45.30	Bacteroidales	96728	3986714
Sample_LCA09.mb.64	63.10	Bifidobacteriaceae	19209	2027732
Sample_LCA09.Cluster2032	42.50	Bacteria	26851	4304393
Sample_LCA09.Cluster6035cbin.1	46.40	Bacteroidales	100989	4169903
Sample_LCA09.Cluster9790cbin.1	42.00	Bacteroidales	70195	4494863
Sample_LCA09.mb.86cbin.1	44.20	Clostridiales	50440	3382098
Sample_LCA09.mb.63	48.40	Proteobacteria	16344	2629823
Sample_LCA09.mb.98cbin.1	41.00	Lachnospiraceae	74690	2961883
Sample_LCA10.mb.27cbin.1	60.30	Bacteria	11544	3762679
Sample_LCA10.Cluster26cbin.1	60.40	Clostridiales	8375	1986601
Sample_LCA10.Cluster330cbin.1	58.80	Clostridiales	25952	2158955
Sample_LCA10.Cluster54	62.60	Actinobacteria	268425	2307108
Sample_LCA10.mb.10	59.30	Clostridiales	128017	2184818
Sample_LCA10.Cluster1853cbin.1	48.20	Clostridiales	60223	2958549
Sample_LCA10.Cluster2071cbin.1	46.60	Lactobacillales	195396	2090005
Sample_LCA10.Cluster140	60.80	Clostridiales	182857	2755946
Sample_LCA10.mb.18	55.60	Clostridiales	91054	3241888
Sample_LCA10.mb.32cbin.1	48.50	Firmicutes	22954	1966836
Sample_LCA10.mb.1	56.50	Clostridiales	14201	2474162

Sample_LCA10.Cluster1516cbin.1	51.30	Clostridiales	239100	2100557
Sample_LCA10.mb.60cbin.1	59.70	Actinobacteria	93219	2118795
Sample_LCA10.Cluster160cbin.1	59.20	Clostridiales	14284	2829854
Sample_LCA10.mb.51cbin.1	54.30	Bacteroidetes	130663	2081513
Sample_LCA10.mb.52	60.90	Clostridiales	12936	2175482
Sample_LCA10.mb.47	55.20	Bacteroidetes	108862	2583108
Sample_LCA10.Cluster63	62.90	Bifidobacteriaceae	138410	2104755
Sample_LCA10.mb.55cbin.1	59.60	Bifidobacteriaceae	160525	2317362
Sample_LCA10.Cluster332	58.20	Bacteroidetes	221031	3115781
Sample_LCA10.mb.39cbin.1	46.80	Lachnospiraceae	78204	7052311
Sample_LCA10.mb.37cbin.1	48.60	Proteobacteria	19360	2447060
Sample_LCA10.mb.44cbin.1	41.60	Lachnospiraceae	70078	2627420
Sample_LCA10.Cluster399	58.70	Bacteroidetes	229350	3231323
Sample_LCA10.Cluster2087	43.40	Bacteria	22154	4010613
Sample_LCA10.Cluster1830cbin.1	44.30	Bacteria	24887	3703031
Sample_LCA10.Cluster1398cbin.1	49.00	Clostridiales	101968	7280753
Sample_LCA10.mb.48	42.50	Bacteroides	49674	3621940
Sample_LCA10.Cluster2295cbin.1	44.90	Bacteroidales	129300	4846684
Sample_LCA10.mb.67	50.80	Enterobacteriaceae	108120	4434875
Sample_LCA11.mb.110cbin.1	49.60	Clostridia	118605	1816000
Sample_LCA11.Cluster5845	48.80	Bacteria	229795	2013212
Sample_LCA11.Cluster295	60.60	Clostridiales	73213	1847372
Sample_LCA11.mb.27	47.20	Clostridiales	32885	1903985
Sample_LCA11.mb.4	26.70	Bacteria	36824	1212042
Sample_LCA11.Cluster7780cbin.1	47.70	Selenomonadales	13236	1960234
Sample_LCA11.Cluster4752	51.60	Clostridiales	187499	1952555
Sample_LCA11.Cluster10191	40.60	Clostridiales	162611	2041317
Sample_LCA11.Cluster7430	45.90	Clostridiales	83788	1877021
Sample_LCA11.Cluster11756cbin.1	37.80	Clostridiales	157933	2041018
Sample_LCA11.Cluster2198	55.60	Bacteria	131844	2686888
Sample_LCA11.Cluster12733cbin.1	28.90	Bacteria	4800	1889081
Sample_LCA11.mb.24cbin.1	59.90	Clostridiales	53096	2237570
Sample_LCA11.mb.113	58.20	Bacteroidetes	188168	2636747
Sample_LCA11.mb.22	53.90	Clostridiales	8688	1783508
Sample_LCA11.mb.74	26.10	Bacteria	55004	1301627
Sample_LCA11.Cluster9799cbin.1	41.00	Lachnospiraceae	14826	2519852
Sample_LCA11.Cluster13155	30.80	Bacteria	81472	3612470
Sample_LCA11.mb.37	56.60	Clostridiales	13899	2171228
Sample_LCA11.mb.39cbin.1	58.90	Clostridiales	16363	2281390
Sample_LCA11.mb.60cbin.1	61.80	Clostridiales	12354	1551588
Sample_LCA11.mb.88	27.10	Clostridiales	154033	1727017
Sample_LCA11.Cluster6942cbin.1	43.10	Bacteria	11162	3800019
Sample_LCA11.Cluster9181cbin.1	48.30	Clostridiales	19990	6081383
Sample_LCA11.mb.96	52.90	Clostridiales	68512	2107163
Sample_LCA11.mb.44	44.30	Clostridiales	48598	2655388
Sample_LCA11.Cluster6646	45.50	Bacteroidales	124524	3896096
Sample_LCA11.mb.62cbin.1	29.00	Bacteria	17887	2340432
Sample_LCA11.mb.89cbin.1	48.70	Clostridiales	16146	2719951
Sample_LCA11.mb.85cbin.1	54.90	Clostridiales	68949	2354366
Sample_LCA11.Cluster9182cbin.1	44.90	Bacteroidales	77288	4729796
Sample_LCA11.mb.29cbin.1	46.20	Bacteroidales	89078	3342386
Sample_LCA11.mb.105cbin.1	42.10	Bacteroidales	117272	4499374
Sample_LCA11.mb.81cbin.1	46.70	Lachnospiraceae	149024	7356807
Sample_LCA11.mb.55	46.70	Bacteroidales	36464	3937491
Sample_LCA11.Cluster4813cbin.1	50.50	Enterobacteriaceae	145958	4707519
Sample_LCA11.mb.75	58.10	Enterobacteriaceae	25175	4717513
Sample_LCA12.Cluster17512	28.60	Bacteria	17065	1275895
Sample_LCA12.Cluster10145	41.80	Clostridiales	164661	2286271

Sample_LCA12.Cluster16605	33.20	Clostridiales	82171	2366103
Sample_LCA12.Cluster6897cbin.1	48.10	Clostridia	241705	2336834
Sample_LCA12.mb.139cbin.1	27.50	Bacteria	112841	1163787
Sample_LCA12.mb.111	58.20	Clostridiales	47864	2895916
Sample_LCA12.mb.136	52.80	Clostridia	83412	2179673
Sample_LCA12.Cluster4404cbin.1	48.10	Bacteroidales	88177	2475525
Sample_LCA12.mb.3	26.30	Bacteria	191038	1382881
Sample_LCA12.Cluster12909	42.10	Clostridiales	82411	1652494
Sample_LCA12.Cluster3695cbin.1	54.20	Bacteroidetes	90701	1937696
Sample_LCA12.Cluster4266cbin.1	49.10	Bacteroidales	139519	3062166
Sample_LCA12.mb.116	59.00	Clostridiales	31554	1955358
Sample_LCA12.Cluster10369	44.60	Clostridiales	199730	2177719
Sample_LCA12.Cluster3601	53.90	Clostridiales	176663	1833872
Sample_LCA12.Cluster10cbin.1	62.90	Proteobacteria	7693	1950421
Sample_LCA12.Cluster4880cbin.1	51.30	Clostridiales	121630	2075392
Sample_LCA12.Cluster9314	45.00	Clostridiales	83352	2530445
Sample_LCA12.Cluster10866	44.40	Clostridiales	151352	2654799
Sample_LCA12.Cluster15	63.70	Deltaproteobacteria	49480	2628224
Sample_LCA12.Cluster717	55.70	Bacteria	43054	2690980
Sample_LCA12.mb.49	45.90	Clostridia	33744	1762286
Sample_LCA12.mb.22	62.60	Clostridiales	18334	1736297
Sample_LCA12.mb.112	61.20	Bacteroidetes	155090	2358159
Sample_LCA12.Cluster13856cbin.1	38.20	Lactobacillales	5940	2513520
Sample_LCA12.mb.124cbin.1	56.40	Clostridiales	11248	2530689
Sample_LCA12.mb.129cbin.1	52.80	Clostridiales	157064	2239211
Sample_LCA12.mb.58cbin.1	50.70	Bacteria	59013	1907398
Sample_LCA12.mb.110	41.70	Lachnospiraceae	59306	2524670
Sample_LCA12.Cluster8154cbin.1	45.10	Prevotella	60385	3526736
Sample_LCA12.mb.43	57.50	Clostridiales	36247	2217132
Sample_LCA12.mb.52cbin.1	57.00	Clostridiales	31326	2228072
Sample_LCA12.mb.4	41.00	Clostridiales	21378	2004160
Sample_LCA12.Cluster7146cbin.1	43.20	Bacteria	17760	3932565
Sample_LCA12.mb.88	48.10	Clostridiales	148678	1795438
Sample_LCA12.mb.83	62.20	Clostridiales	12735	1492049
Sample_LCA12.mb.67	62.50	Clostridiales	73397	1831950
Sample_LCA12.mb.25cbin.1	42.40	Lachnospiraceae	57085	2039443
Sample_LCA12.mb.95	59.80	Clostridiales	100173	2285778
Sample_LCA12.mb.85	40.70	Lachnospiraceae	93215	2695109
Sample_LCA12.mb.65cbin.1	59.70	Actinobacteria	59812	2280874
Sample_LCA12.mb.34	46.00	Bacteroidales	58721	2945807
Sample_LCA12.mb.46	59.10	Bacteroidetes	76230	2913989
Sample_LCA12.mb.6	36.40	Clostridiales	88264	2206289
Sample_LCA12.mb.78	60.70	Clostridiales	36425	2253940
Sample_LCA12.mb.79cbin.1	55.70	Bacteroidales	141886	3592439
Sample_LCA12.mb.143	42.00	Bacteroidales	18801	4398418
Sample_LCA12.mb.76	41.40	Clostridiales	60697	2529245
Sample_LCA12.mb.97	57.30	Bacteroidetes	95382	2505396
Sample_LCA12.mb.69	43.30	Lachnospiraceae	97628	2699250
Sample_LCA12.Cluster3297cbin.1	51.00	Enterobacteriaceae	27119	4520069
Sample_LCA12.mb.42cbin.1	44.90	Bacteroidales	50340	4541938
Sample_LCA12.mb.47cbin.1	46.40	Bacteroidales	69697	4458931
Sample_LCA12.Cluster256cbin.1	57.80	Enterobacteriaceae	75288	5000218
Sample_LCA13.Cluster6321cbin.1	50.80	Bacteria	76624	1848137
Sample_LCA13.Cluster450cbin.1	60.60	Clostridiales	52053	1664001
Sample_LCA13.Cluster63cbin.1	61.10	Clostridiales	74700	2025824
Sample_LCA13.mb.105	41.40	Clostridiales	12863	3264066
Sample_LCA13.mb.14cbin.1	59.20	Clostridiales	9266	1860171
Sample_LCA13.Cluster97cbin.1	55.40	Clostridiales	27797	2466539

Sample_LCA13.Cluster5880	43.40	Selenomonadales	44376	2070363
Sample_LCA13.Cluster4024	53.70	Clostridiales	377152	1815509
Sample_LCA13.mb.118	56.50	Clostridiales	30440	2235774
Sample_LCA13.mb.110cbin.1	49.10	Clostridiales	8210	2326675
Sample_LCA13.mb.27	58.70	Clostridiales	18009	2210827
Sample_LCA13.Cluster11949	40.60	Clostridiales	130176	2189581
Sample_LCA13.Cluster5089cbin.1	51.90	Clostridiales	146045	2324999
Sample_LCA13.Cluster9916cbin.1	38.90	Clostridiales	74183	2025650
Sample_LCA13.Cluster28	63.40	Deltaproteobacteria	59484	2610584
Sample_LCA13.mb.66	52.50	Clostridiales	92526	2011949
Sample_LCA13.mb.53	58.00	Clostridiales	7968	1793227
Sample_LCA13.mb.19	36.80	Clostridiales	20277	1936163
Sample_LCA13.Cluster10737	43.00	Clostridiales	133120	2609339
Sample_LCA13.Cluster57	59.90	Bifidobacteriaceae	96684	2453599
Sample_LCA13.mb.4	60.90	Bacteroidetes	7343	2241181
Sample_LCA13.mb.22cbin.1	60.20	Bacteroidetes	31545	2564429
Sample_LCA13.mb.36	31.10	Euryarchaeota	29988	1702861
Sample_LCA13.mb.61	59.70	Clostridiales	95224	2300053
Sample_LCA13.Cluster3740cbin.1	46.80	Lachnospiraceae	46022	6960560
Sample_LCA13.Cluster9471	41.50	Lachnospiraceae	24830	2802619
Sample_LCA13.mb.74	61.00	Clostridiales	45853	1797423
Sample_LCA13.mb.107	59.00	Clostridiales	117217	3466009
Sample_LCA13.mb.116	42.00	Lachnospiraceae	87674	2373425
Sample_LCA13.mb.86	49.90	Clostridiales	79376	2312046
Sample_LCA13.mb.63	56.10	Clostridiales	13662	2502366
Sample_LCA13.mb.109	43.50	Bacteroidales	108213	3084042
Sample_LCA13.mb.65	59.40	Clostridiales	9461	2651022
Sample_LCA13.mb.78	55.00	Bacteroidetes	42977	2194853
Sample_LCA13.mb.90cbin.1	60.90	Clostridiales	21720	2102709
Sample_LCA13.Cluster8570	45.20	Bacteroidales	232237	4559494
Sample_LCA13.Cluster12558cbin.1	42.40	Bacteroides	80510	4701938
Sample_LCA13.mb.102cbin.1	42.20	Bacteroidales	46364	4744595
Sample_LCA13.mb.44	45.60	Bacteroidales	42712	3728042
Sample_LCA13.mb.9cbin.1	43.50	Bacteria	113950	4256297
Sample_LCA13.mb.67	46.40	Bacteroidales	307301	4149752
Sample_LCA13.Cluster1780cbin.1	58.10	Enterobacteriaceae	23795	4815174
Sample_LCA13.mb.100cbin.1	50.50	Enterobacteriaceae	38536	4683559
Sample_LCA14.Cluster1346	51.80	Clostridia	315366	2420397
Sample_LCA14.Cluster2622	46.80	Lactobacillales	150159	1954058
Sample_LCA14.Cluster1004	52.00	Clostridiales	120115	2467938
Sample_LCA14.Cluster815cbin.1	60.20	Clostridiales	56310	2744251
Sample_LCA14.mb.51	61.00	Clostridiales	144139	2625416
Sample_LCA14.mb.61cbin.1	52.90	Clostridiales	74728	2674257
Sample_LCA14.Cluster4930	32.50	Lactobacillales	64809	1868251
Sample_LCA14.Cluster1036cbin.1	52.70	Lactobacillales	48310	1806169
Sample_LCA14.Cluster351cbin.1	53.50	Selenomonadales	57206	2369171
Sample_LCA14.Cluster5	60.60	Bifidobacteriaceae	47737	2361672
Sample_LCA14.mb.67	56.30	Clostridiales	14644	2285865
Sample_LCA14.mb.22	34.50	Lactobacillus	466302	1854500
Sample_LCA14.Cluster964cbin.1	60.30	Deltaproteobacteria	7328	3454812
Sample_LCA14.Cluster308cbin.1	59.60	Clostridiales	131226	2998515
Sample_LCA14.mb.2	28.50	Clostridiales	35100	2901726
Sample_LCA14.mb.26	42.20	Bacteroidales	49411	3199134
Sample_LCA14.Cluster362	58.30	Bacteroidetes	344837	3208383
Sample_LCA14.mb.14	41.80	Lachnospiraceae	80509	2414167
Sample_LCA14.Cluster1967cbin.1	48.50	Bacteroidales	204138	3636460
Sample_LCA14.mb.15cbin.1	46.40	Bacteroidales	247651	4180831
Sample_LCA14.mb.65cbin.1	45.10	Bacteroidales	32695	4623266

Sample_LCA14.mb.12	51.20	Enterobacteriaceae	9508	3946154
Sample_LCA14.mb.60	49.60	Clostridiales	356694	5833480
Sample_LCA15.mb.47	46.90	Bacteria	48229	1735257
Sample_LCA15.mb.39cbin.1	33.70	Clostridiales	12093	1986017
Sample_LCA15.mb.46	34.20	Clostridiales	118508	2394224
Sample_LCA15.Cluster1357cbin.1	47.80	Clostridiales	84848	2938475
Sample_LCA15.mb.24	48.80	Clostridiales	56135	2615047
Sample_LCA15.mb.53cbin.1	60.80	Clostridiales	7004	2119043
Sample_LCA15.mb.12	53.80	Clostridiales	515830	1893267
Sample_LCA15.Cluster1569	43.60	Selenomonadales	60270	2282753
Sample_LCA15.mb.20cbin.1	56.40	Clostridiales	12350	2516885
Sample_LCA15.Cluster3639	41.00	Clostridiales	220948	2061917
Sample_LCA15.Cluster2898cbin.1	44.10	Clostridiales	9655	2928115
Sample_LCA15.Cluster41cbin.1	60.10	Bifidobacteriaceae	8655	2001023
Sample_LCA15.mb.1	59.60	Clostridiales	26698	1929194
Sample_LCA15.mb.45cbin.1	57.10	Bifidobacteriaceae	118203	2108691
Sample_LCA15.Cluster5216cbin.1	36.00	Clostridiales	170919	2513618
Sample_LCA15.mb.18cbin.1	44.90	Clostridiales	38201	2667273
Sample_LCA15.Cluster3291	41.30	Lachnospiraceae	90085	2786023
Sample_LCA15.mb.48	41.40	Clostridiales	48047	2947049
Sample_LCA15.mb.69	38.50	Lactobacillales	41938	2398456
Sample_LCA15.mb.59	42.80	Lachnospiraceae	99764	3115405
Sample_LCA15.Cluster1253cbin.1	46.10	Bacteroidales	82134	3466384
Sample_LCA15.mb.33	46.60	Bacteroidales	44479	4161622
Sample_LCA15.Cluster753cbin.1	41.90	Bacteroidales	42908	4693259
Sample_LCA15.mb.61cbin.1	50.60	Enterobacteriaceae	154695	4550922
Sample_LCA15.Cluster35	56.20	Enterobacteriaceae	199776	5279330
Sample_LCA16.Cluster2110	50.60	Bacteria	55100	1961387
Sample_LCA16.mb.26	49.50	Clostridiales	73213	2002805
Sample_LCA16.mb.32cbin.1	58.00	Clostridiales	36613	3080636
Sample_LCA16.Cluster1219	52.20	Bacteria	16887	4205078
Sample_LCA16.Cluster252cbin.1	59.90	Bacteria	102925	5033228
Sample_LCA16.mb.13	47.00	Lachnospiraceae	70409	2618940
Sample_LCA16.Cluster6403cbin.1	41.00	Clostridiales	11162	1899318
Sample_LCA16.mb.56	49.60	Clostridiales	36811	2243930
Sample_LCA16.Cluster7169	37.60	Clostridiales	156298	2032362
Sample_LCA16.mb.14cbin.1	44.80	Clostridiales	11448	2314447
Sample_LCA16.mb.29	59.50	Bifidobacteriaceae	37758	2054274
Sample_LCA16.mb.61	53.40	Clostridiales	141702	1909501
Sample_LCA16.mb.75cbin.1	45.40	Bacteroidales	97628	3422065
Sample_LCA16.mb.6	41.10	Clostridiales	206911	2642502
Sample_LCA16.Cluster2784cbin.1	43.40	Bacteria	5384	3277998
Sample_LCA16.Cluster4602	43.40	Bacteroidales	148215	3081283
Sample_LCA16.Cluster3983cbin.1	48.10	Bacteroidales	229250	3433431
Sample_LCA16.Cluster7377	37.50	Lactobacillales	177811	2720356
Sample_LCA16.mb.9cbin.1	41.20	Lachnospiraceae	68635	2946017
Sample_LCA16.Cluster3119cbin.1	42.50	Bacteria	14575	4066686
Sample_LCA16.mb.12cbin.1	45.20	Bacteroidales	8208	3882001
Sample_LCA16.Cluster777cbin.1	50.70	Enterobacteriaceae	80004	4667295
Sample_LCA16.mb.1cbin.1	57.40	Enterobacteriaceae	184092	5228175
Sample_LCA17.mb.41cbin.1	46.00	Bacteria	31069	1736000
Sample_LCA17.mb.49	49.70	Clostridiales	8391	1634387
Sample_LCA17.Cluster8456cbin.1	41.40	Clostridiales	43180	2612981
Sample_LCA17.Cluster550cbin.1	60.10	Clostridiales	54511	2524012
Sample_LCA17.Cluster3336cbin.1	50.30	Bacteroidales	15100	2478899
Sample_LCA17.Cluster2742cbin.1	48.30	Firmicutes	32847	1646286
Sample_LCA17.Cluster8642cbin.1	40.30	Clostridiales	145770	1591401
Sample_LCA17.mb.88	26.60	Bacteria	89492	1501098

Sample_LCA17.mb.70	58.40	Clostridiales	45025	2773052
Sample_LCA17.mb.62cbin.1	59.60	Clostridiales	6924	1606809
Sample_LCA17.mb.56cbin.1	61.90	Clostridiales	12644	1920023
Sample_LCA17.Cluster10970cbin.1	27.40	Clostridiales	7331	2590572
Sample_LCA17.mb.60cbin.1	60.50	Clostridiales	82074	1876779
Sample_LCA17.mb.46	58.60	Bacteroidetes	6406	2382627
Sample_LCA17.mb.96	53.00	Clostridia	81079	2075418
Sample_LCA17.mb.22cbin.1	60.00	Bifidobacteriaceae	16880	1967919
Sample_LCA17.mb.38cbin.1	60.10	Bacteroidetes	89590	2501919
Sample_LCA17.Cluster49cbin.1	56.00	Proteobacteria	5471	2084612
Sample_LCA17.mb.54cbin.1	46.00	Lachnospiraceae	75552	3073746
Sample_LCA17.mb.58	46.30	Bacteroidetes	40711	2330341
Sample_LCA17.Cluster8124cbin.1	40.90	Lachnospiraceae	54813	3279331
Sample_LCA17.Cluster8958cbin.1	38.30	Bacteroidales	28195	3398350
Sample_LCA17.mb.61	50.30	Bacteroidales	103715	2194329
Sample_LCA17.mb.24	41.30	Bacteroidales	90369	2957054
Sample_LCA17.mb.76cbin.1	56.30	Bacteroidales	61169	3396025
Sample_LCA17.mb.79cbin.1	36.50	Clostridiales	9829	1751122
Sample_LCA17.mb.73	58.00	Clostridiales	63919	2505567
Sample_LCA17.mb.97	60.20	Actinobacteria	8692	1847576
Sample_LCA17.mb.80	38.50	Clostridiales	81777	2140376
Sample_LCA17.mb.94	54.70	Bacteroidetes	79177	2083594
Sample_LCA17.mb.9	51.40	Clostridiales	187481	2183630
Sample_LCA17.mb.50cbin.1	43.50	Bacteria	38380	3935825
Sample_LCA17.mb.74cbin.1	47.50	Bacteroidales	35908	3718630
Sample_LCA17.mb.3cbin.1	45.20	Bacteroidales	25852	4212481
Sample_LCA17.mb.40	45.50	Bacteroidales	25959	4249297
Sample_LCA17.Cluster534cbin.1	51.60	Enterobacteriaceae	5787	3394643
Sample_LCA19.Cluster6802cbin.1	26.40	Bacteria	8623	1011539
Sample_LCA19.Cluster1769cbin.1	46.30	Bacteria	46448	1864065
Sample_LCA19.Cluster1748	47.60	Clostridiales	99248	2562977
Sample_LCA19.mb.77cbin.1	61.60	Clostridiales	102253	1955215
Sample_LCA19.Cluster1073	53.60	Clostridiales	615950	1817354
Sample_LCA19.Cluster674cbin.1	54.30	Bacteroidetes	117549	2152854
Sample_LCA19.mb.41cbin.1	60.60	Bacteroidetes	7226	2267390
Sample_LCA19.mb.50cbin.1	63.70	Actinobacteria	7941	2279656
Sample_LCA19.Cluster4112	41.00	Clostridiales	195885	2060029
Sample_LCA19.Cluster201cbin.1	56.20	Clostridiales	14025	2518399
Sample_LCA19.Cluster314cbin.1	56.20	Proteobacteria	10121	1852691
Sample_LCA19.mb.60	48.90	Clostridiales	59921	2569957
Sample_LCA19.mb.88	59.80	Clostridiales	21622	1971250
Sample_LCA19.Cluster6064	35.90	Clostridiales	199977	2507362
Sample_LCA19.mb.39cbin.1	57.20	Bifidobacteriaceae	116936	2161893
Sample_LCA19.Cluster4036cbin.1	40.80	Lachnospiraceae	79906	3052510
Sample_LCA19.mb.75cbin.1	59.00	Clostridiales	39040	2233593
Sample_LCA19.mb.85	42.70	Clostridiales	37165	2571650
Sample_LCA19.mb.47	42.10	Bacteroidales	64312	3339053
Sample_LCA19.mb.58cbin.1	44.50	Clostridiales	29315	3063021
Sample_LCA19.mb.48	38.30	Lactobacillales	24981	2602845
Sample_LCA19.Cluster1508	46.50	Bacteroidales	87048	3054431
Sample_LCA19.mb.52	42.00	Bacteroides	31066	4546916
Sample_LCA19.mb.23cbin.1	45.20	Bacteroidales	13810	4237696
Sample_LCA19.mb.36	56.10	Enterobacteriaceae	209812	5468709
Sample_LCA20.mb.10cbin.1	29.30	Bacteria	130412	1123297
Sample_LCA20.mb.30	26.00	Bacteria	85357	1395082
Sample_LCA20.mb.57	26.40	Bacteria	127272	1205898
Sample_LCA20.Cluster427cbin.1	58.30	Clostridiales	48223	2809631
Sample_LCA20.mb.34cbin.1	46.10	Clostridiales	85333	2160390

Sample_LCA20.mb.12	37.50	Clostridiales	174249	2340351
Sample_LCA20.Cluster408	58.50	Clostridiales	26731	1612848
Sample_LCA20.mb.104	61.70	Clostridiales	34543	1909025
Sample_LCA20.Cluster1109cbin.1	54.90	Clostridiales	37053	2361746
Sample_LCA20.mb.59	44.60	Clostridiales	13291	2406005
Sample_LCA20.Cluster2172cbin.1	50.00	Clostridiales	122574	2325557
Sample_LCA20.Cluster6765	40.60	Clostridiales	135599	1918207
Sample_LCA20.Cluster8336cbin.1	37.20	Clostridiales	121907	1832603
Sample_LCA20.Cluster5171	44.50	Clostridiales	152858	1852628
Sample_LCA20.mb.1cbin.1	56.10	Clostridiales	15886	2464978
Sample_LCA20.mb.6cbin.1	61.70	Clostridiales	196102	1961348
Sample_LCA20.mb.36	46.60	Clostridiales	12439	1999408
Sample_LCA20.Cluster6794cbin.1	40.50	Clostridiales	183998	2177345
Sample_LCA20.Cluster2547	51.60	Clostridiales	179445	2319810
Sample_LCA20.mb.43	54.90	Bacteroidetes	123145	2206642
Sample_LCA20.mb.44cbin.1	48.90	Bacteroidales	167034	3169219
Sample_LCA20.Cluster133cbin.1	59.30	Bacteroidetes	69064	2850377
Sample_LCA20.mb.13	37.60	Clostridiales	114021	2492697
Sample_LCA20.mb.7	50.90	Prevotella	67928	2318866
Sample_LCA20.mb.75cbin.1	31.10	Euryarchaeota	58487	1759703
Sample_LCA20.mb.98	48.50	Lachnospiraceae	9930	3242367
Sample_LCA20.mb.102	43.40	Bacteroidales	259581	3164479
Sample_LCA20.mb.56	47.80	Bacteroidales	25620	3134597
Sample_LCA20.mb.66cbin.1	42.70	Bacteria	7395	3766753
Sample_LCA20.Cluster4859cbin.1	45.90	Bacteroidales	17462	4320175
Sample_LCA20.Cluster2539cbin.1	50.80	Enterobacteriaceae	147476	4515762
Sample_LCA20.mb.55	57.70	Enterobacteriaceae	229737	5042511
Sample_LCA21.mb.26cbin.1	53.80	Clostridia	30625	1677810
Sample_LCA21.mb.108	59.60	Clostridiales	7456	1728628
Sample_LCA21.Cluster3243cbin.1	50.50	Bacteroidales	66438	2208584
Sample_LCA21.Cluster461cbin.1	58.40	Clostridiales	42765	2597859
Sample_LCA21.mb.35	61.20	Clostridiales	62278	1789857
Sample_LCA21.mb.10	49.30	Clostridiales	18874	2324659
Sample_LCA21.Cluster4740	49.00	Clostridiales	68609	2624558
Sample_LCA21.Cluster3612cbin.1	48.40	Firmicutes	32127	1839914
Sample_LCA21.Cluster4672cbin.1	43.80	Selenomonadales	57914	2197014
Sample_LCA21.mb.79	49.60	Clostridiales	92394	1945536
Sample_LCA21.mb.12	40.80	Clostridiales	69515	2041587
Sample_LCA21.mb.51	41.20	Lachnospiraceae	109996	2507014
Sample_LCA21.mb.107	44.60	Clostridiales	101660	2187399
Sample_LCA21.mb.13	43.10	Clostridiales	43020	3157133
Sample_LCA21.mb.88cbin.1	33.30	Clostridiales	44913	2039849
Sample_LCA21.Cluster4089cbin.1	49.80	Lachnospiraceae	80397	2716028
Sample_LCA21.mb.71cbin.1	45.20	Clostridiales	7085	2139055
Sample_LCA21.mb.61cbin.1	51.50	Clostridiales	148149	2109813
Sample_LCA21.mb.93	34.10	Bacteria	30984	1857694
Sample_LCA21.mb.103cbin.1	31.50	Firmicutes	57810	2223166
Sample_LCA21.mb.59cbin.1	44.50	Clostridiales	37630	2611635
Sample_LCA21.mb.98	61.70	Clostridiales	81345	2344361
Sample_LCA21.mb.21	48.40	Proteobacteria	63977	2619985
Sample_LCA21.mb.9	44.50	Lachnospiraceae	129588	3050140
Sample_LCA21.mb.17cbin.1	46.10	Bacteroidales	38780	3362423
Sample_LCA21.mb.66cbin.1	58.40	Bacteroidetes	114712	3168142
Sample_LCA21.mb.95cbin.1	40.90	Lachnospiraceae	12854	2407295
Sample_LCA21.mb.90	62.40	Deltaproteobacteria	30767	3715508
Sample_LCA21.mb.99cbin.1	41.20	Lachnospiraceae	97675	2855975
Sample_LCA21.mb.112cbin.1	45.30	Bacteroidales	50425	4587977
Sample_LCA21.mb.69	45.60	Bacteroidales	30137	3720874

Sample_LCA21.mb.67cbin.1	47.30	Bacteroidales	16274	3905429
Sample_LCA21.Cluster5531cbin.1	50.70	Enterobacteriaceae	31254	4857403
Sample_LCA22.Cluster2200	50.80	Bacteria	99385	1838011
Sample_LCA22.mb.26	26.80	Bacteria	22864	1303836
Sample_LCA22.Cluster2096cbin.1	48.60	Clostridiales	18132	2923885
Sample_LCA22.Cluster321	58.30	Clostridiales	66639	2325620
Sample_LCA22.mb.21	58.70	Clostridiales	29407	2017010
Sample_LCA22.Cluster251	59.50	Actinobacteria	111982	2249989
Sample_LCA22.mb.29	61.10	Clostridiales	75294	2088623
Sample_LCA22.Cluster3338	47.30	Selenomonadales	84326	2122412
Sample_LCA22.Cluster5574	41.50	Clostridiales	351088	1777278
Sample_LCA22.mb.79	32.80	Bacteria	88065	1940528
Sample_LCA22.Cluster227	55.40	Bacteria	189963	2722398
Sample_LCA22.mb.74	41.30	Clostridiales	5721	2481202
Sample_LCA22.mb.93	49.40	Clostridia	20541	1843339
Sample_LCA22.Cluster274cbin.1	56.70	Bifidobacteriaceae	39951	1996484
Sample_LCA22.mb.73	61.10	Clostridiales	105350	1676789
Sample_LCA22.Cluster119cbin.1	61.60	Proteobacteria	42240	2479948
Sample_LCA22.mb.58cbin.1	49.80	Clostridiales	23370	2231879
Sample_LCA22.mb.20cbin.1	36.10	Clostridiales	127923	2056400
Sample_LCA22.mb.92	58.10	Clostridiales	14369	3040378
Sample_LCA22.mb.45	46.80	Lachnospiraceae	56476	2695178
Sample_LCA22.mb.36cbin.1	38.50	Clostridiales	71575	2229222
Sample_LCA22.mb.81cbin.1	54.90	Bacteroidetes	34363	1951292
Sample_LCA22.mb.44	43.60	Lachnospiraceae	97263	2714393
Sample_LCA22.Cluster5426cbin.1	42.10	Bacteroidales	63074	3439543
Sample_LCA22.Cluster8517cbin.1	40.70	Lachnospiraceae	77574	3259047
Sample_LCA22.mb.77	41.30	Clostridiales	264913	2432238
Sample_LCA22.Cluster3734cbin.1	42.90	Bacteria	28244	4229709
Sample_LCA22.mb.51	41.70	Clostridiales	17364	3093716
Sample_LCA22.mb.33cbin.1	45.10	Bacteroidales	89660	4245767
Sample_LCA22.mb.22cbin.1	44.40	Bacteroidales	44520	4896396
Sample_LCA22.mb.91cbin.1	41.90	Bacteroidales	32217	3964990
Sample_LCA22.mb.15cbin.1	50.60	Enterobacteriaceae	172256	4912829
Sample_LCA23.mb.29cbin.1	47.10	Clostridiales	8500	2323921
Sample_LCA23.mb.11cbin.1	60.30	Clostridiales	38639	1887666
Sample_LCA23.Cluster2542	51.50	Bacteroidales	74542	2091827
Sample_LCA23.Cluster263	59.80	Actinobacteria	135893	2158730
Sample_LCA23.mb.33cbin.1	41.30	Lachnospiraceae	17399	2363288
Sample_LCA23.Cluster3080cbin.1	47.40	Selenomonadales	27805	2068676
Sample_LCA23.mb.28	49.30	Clostridiales	25696	2415462
Sample_LCA23.mb.54	58.70	Clostridiales	66717	1984022
Sample_LCA23.Cluster406	55.90	Bacteria	13657	2383098
Sample_LCA23.Cluster8070	40.80	Clostridiales	143216	2089678
Sample_LCA23.mb.59	61.30	Clostridiales	11404	2589026
Sample_LCA23.Cluster213cbin.1	54.30	Selenomonadales	7917	1796248
Sample_LCA23.Cluster5816cbin.1	44.30	Clostridiales	153618	2732737
Sample_LCA23.mb.53	54.50	Bacteroidetes	109550	2132464
Sample_LCA23.Cluster5538cbin.1	44.70	Clostridiales	74103	3103907
Sample_LCA23.Cluster8409cbin.1	41.30	Lachnospiraceae	55650	3115096
Sample_LCA23.mb.67cbin.1	38.10	Clostridiales	180426	2029457
Sample_LCA23.mb.68	59.90	Bacteroidetes	21195	2725323
Sample_LCA23.Cluster3820cbin.1	43.10	Lachnospiraceae	35901	3106836
Sample_LCA23.Cluster4195cbin.1	43.00	Bacteria	5943	3248282
Sample_LCA23.mb.86	51.70	Clostridiales	10738	1917687
Sample_LCA23.mb.4cbin.1	63.10	Proteobacteria	47402	2609997
Sample_LCA23.mb.70cbin.1	50.00	Bacteroidales	64787	2430984
Sample_LCA23.mb.30	40.70	Lachnospiraceae	74307	3540843

Sample_LCA23.mb.84	43.80	Lachnospiraceae	101065	2789227
Sample_LCA23.mb.76cbin.1	56.90	Clostridiales	13853	2221188
Sample_LCA23.Cluster6963cbin.1	45.10	Bacteroidales	20576	4282279
Sample_LCA23.mb.74cbin.1	48.60	Bacteroidales	21205	3495794
Sample_LCA23.mb.40cbin.1	45.40	Bacteroidales	83842	4151708
Sample_LCA23.mb.85cbin.1	50.80	Enterobacteriaceae	39745	4761826
Sample_LCA24.mb.24	57.80	Bacteria	31735	2182854
Sample_LCA24.Cluster446cbin.1	51.30	Clostridiales	9882	2191261
Sample_LCA24.mb.16	60.00	Actinobacteria	28139	2163928
Sample_LCA24.Cluster4405cbin.1	34.80	Lactobacillus	5853	1478843
Sample_LCA24.Cluster1988cbin.1	44.70	Clostridiales	187577	2038269
Sample_LCA24.mb.42cbin.1	48.40	Firmicutes	27661	2104943
Sample_LCA24.mb.25	60.60	Bacteroidetes	29931	2415045
Sample_LCA24.mb.38cbin.1	56.70	Bifidobacteriaceae	27343	1837304
Sample_LCA24.Cluster4798	32.50	Lactobacillales	141595	1866162
Sample_LCA24.mb.40	60.00	Bifidobacteriaceae	154633	2195294
Sample_LCA24.Cluster11	62.70	Proteobacteria	37047	2265850
Sample_LCA24.mb.55	54.00	Selenomonadales	78560	2226660
Sample_LCA24.mb.9	63.30	Bifidobacteriaceae	24565	2152688
Sample_LCA24.mb.19	59.40	Bacteroidetes	153647	2761017
Sample_LCA24.mb.20	46.20	Bacteroidales	31201	2839222
Sample_LCA24.Cluster1580cbin.1	46.60	Bacteroidales	97433	2850581
Sample_LCA24.mb.33cbin.1	41.10	Lachnospiraceae	97677	3112422
Sample_LCA24.mb.7	46.40	Bacteroidales	111975	3577876
Sample_LCA25.mb.50	38.10	Clostridiales	258770	1850740
Sample_LCA25.mb.8	49.50	Clostridiales	8517	2139410
Sample_LCA25.mb.20	40.90	Clostridiales	189416	2094720
Sample_LCA25.Cluster2783	43.30	Selenomonadales	133146	2487308
Sample_LCA25.mb.15	60.10	Bacteroidetes	24675	2618771
Sample_LCA25.mb.7	54.90	Bacteroidetes	134900	2059803
Sample_LCA25.Cluster326cbin.1	58.00	Clostridiales	33428	2018387
Sample_LCA25.mb.19cbin.1	43.70	Bacteroidales	5946	2240942
Sample_LCA25.mb.35cbin.1	60.30	Bifidobacteriaceae	27386	2349596
Sample_LCA25.Cluster1744cbin.1	45.80	Bacteroidales	78136	2793951
Sample_LCA25.Cluster3706cbin.1	40.70	Lachnospiraceae	61081	3123884
Sample_LCA25.Cluster6469	29.00	Bacteria	72623	2595685
Sample_LCA25.mb.6cbin.1	45.20	Prevotella	33414	3152665
Sample_LCA25.Cluster2442cbin.1	41.20	Lachnospiraceae	56801	3598065
Sample_LCA25.mb.18cbin.1	31.10	Firmicutes	55154	2507571
Sample_LCA25.mb.26	42.70	Lachnospiraceae	90930	3565096
Sample_LCA25.Cluster1321cbin.1	45.30	Bacteroidales	6276	3624736
Sample_LCA25.Cluster192	51.00	Enterobacteriaceae	74263	4390086
Sample_LCB09.Cluster10320	26.00	Bacteria	307090	1297737
Sample_LCB09.mb.33	53.60	Clostridia	37615	1775449
Sample_LCB09.Cluster9274cbin.1	36.60	Clostridiales	8646	2163829
Sample_LCB09.mb.34	49.50	Clostridiales	94043	1925495
Sample_LCB09.mb.22	32.40	Clostridiales	44233	2587276
Sample_LCB09.mb.57	42.60	Bacteria	70105	5626329
Sample_LCB09.Cluster2908cbin.1	45.60	Clostridiales	132839	2530943
Sample_LCB09.mb.19cbin.1	59.30	Clostridiales	14461	1811227
Sample_LCB09.Cluster66cbin.1	60.80	Clostridiales	43751	2103093
Sample_LCB09.mb.46cbin.1	48.80	Lachnospiraceae	13957	2828857
Sample_LCB09.Cluster1827	51.40	Clostridiales	192362	1976424
Sample_LCB09.mb.58cbin.1	56.60	Clostridiales	71450	2374753
Sample_LCB09.Cluster7974	40.90	Clostridiales	189620	2050523
Sample_LCB09.mb.49cbin.1	48.10	Clostridiales	26410	3042865
Sample_LCB09.Cluster9335	32.70	Lactobacillales	6420	1577210
Sample_LCB09.mb.78	53.00	Clostridia	58824	2012740

Sample_LCB09.mb.65cbin.1	56.10	Clostridiales	55327	2462172
Sample_LCB09.Cluster862cbin.1	56.30	Bifidobacteriaceae	182411	1951049
Sample_LCB09.mb.31cbin.1	55.90	Bacteria	12655	2523314
Sample_LCB09.mb.70	62.30	Clostridiales	30052	1811966
Sample_LCB09.mb.42cbin.1	60.10	Bacteroidetes	29908	2682553
Sample_LCB09.mb.69cbin.1	60.90	Clostridiales	54550	2250639
Sample_LCB09.mb.18	37.50	Clostridiales	74231	2657586
Sample_LCB09.mb.90	38.40	Clostridiales	8437	2116082
Sample_LCB09.mb.2	42.10	Lachnospiraceae	9529	1997489
Sample_LCB09.mb.54	43.40	Lachnospiraceae	103337	3026272
Sample_LCB09.Cluster6670cbin.1	41.10	Lachnospiraceae	72932	2937043
Sample_LCB09.mb.6	56.00	Clostridiales	16026	2519081
Sample_LCB09.Cluster345cbin.1	58.80	Bacteroidetes	178167	3015535
Sample_LCB09.mb.67	60.00	Bifidobacteriaceae	39226	2153638
Sample_LCB09.mb.82cbin.1	53.70	Clostridiales	173633	1865409
Sample_LCB09.Cluster3531	44.60	Bacteroidales	141285	3709233
Sample_LCB09.mb.73cbin.1	42.60	Clostridiales	141995	2725376
Sample_LCB09.Cluster3297cbin.1	46.40	Bacteroidales	95333	4136645
Sample_LCB09.mb.50cbin.1	45.40	Bacteroidales	97398	3997817
Sample_LCB09.mb.51cbin.1	42.00	Bacteroidales	71779	4617739
Sample_LCB10.Cluster10459	26.00	Bacteria	307090	1298260
Sample_LCB10.mb.100	47.20	Clostridiales	53662	2127013
Sample_LCB10.Cluster43cbin.1	62.30	Clostridiales	5531	1400468
Sample_LCB10.mb.113	38.20	Clostridiales	35584	2464144
Sample_LCB10.mb.30	52.90	Clostridia	71323	2104461
Sample_LCB10.mb.20	32.30	Clostridiales	110739	2543044
Sample_LCB10.Cluster2571cbin.1	46.40	Clostridiales	37217	2202117
Sample_LCB10.mb.104cbin.1	56.30	Clostridiales	58104	2689000
Sample_LCB10.Cluster2169cbin.1	48.50	Firmicutes	28032	1789506
Sample_LCB10.Cluster49	63.00	Bifidobacteriaceae	99063	2163667
Sample_LCB10.mb.71	49.50	Clostridiales	27490	1893512
Sample_LCB10.mb.8	55.30	Bacteria	10392	3304093
Sample_LCB10.mb.58	42.30	Lachnospiraceae	12221	1807304
Sample_LCB10.Cluster7878	40.80	Clostridiales	236790	2026761
Sample_LCB10.Cluster362	55.60	Bacteria	126621	2617114
Sample_LCB10.mb.120cbin.1	45.40	Firmicutes	54195	1722237
Sample_LCB10.mb.106	56.50	Clostridiales	15321	2322218
Sample_LCB10.Cluster2330cbin.1	51.20	Clostridiales	205067	2276375
Sample_LCB10.Cluster10213	32.50	Lactobacillales	128914	1837067
Sample_LCB10.mb.11	53.70	Clostridiales	328209	1884200
Sample_LCB10.mb.19cbin.1	61.00	Clostridiales	15722	2146847
Sample_LCB10.Cluster1270cbin.1	56.20	Bifidobacteriaceae	110243	2017484
Sample_LCB10.mb.31cbin.1	44.00	Selenomonadales	10333	2145461
Sample_LCB10.mb.67	48.20	Clostridiales	59365	2950446
Sample_LCB10.mb.63cbin.1	60.70	Clostridiales	58114	2393225
Sample_LCB10.Cluster7401cbin.1	37.50	Clostridiales	82932	2577894
Sample_LCB10.mb.89	53.70	Clostridia	35989	1767338
Sample_LCB10.mb.52	59.20	Clostridiales	15607	2092853
Sample_LCB10.Cluster2199cbin.1	49.70	Lachnospiraceae	27878	2827088
Sample_LCB10.mb.40cbin.1	59.70	Bacteroidetes	38488	2806601
Sample_LCB10.Cluster5231cbin.1	40.90	Lachnospiraceae	68758	3006117
Sample_LCB10.mb.82cbin.1	56.40	Clostridiales	66421	2714390
Sample_LCB10.mb.44cbin.1	49.80	Lactobacillales	44896	2078042
Sample_LCB10.mb.116cbin.1	48.80	Proteobacteria	17046	2558575
Sample_LCB10.mb.81	60.70	Clostridiales	23636	1957311
Sample_LCB10.Cluster3344	44.60	Bacteroidales	151809	3746935
Sample_LCB10.Cluster5766cbin.1	42.30	Bacteria	9772	4137586
Sample_LCB10.Cluster2061	45.30	Bacteroidales	93516	4000781

Sample_LCB10.mb.93	44.30	Clostridiales	74576	2428757
Sample_LCB10.mb.97cbin.1	42.80	Clostridiales	142866	2671497
Sample_LCB10.mb.99	58.70	Bacteroidetes	204738	3024655
Sample_LCB10.mb.75	46.70	Bacteroidales	101862	3819916
Sample_LCB10.mb.61cbin.1	41.80	Bacteroidales	71779	4652857
Sample_LCB11.mb.22cbin.1	26.10	Bacteria	17703	1242595
Sample_LCB11.Cluster4735	49.50	Clostridiales	91341	1948685
Sample_LCB11.mb.57cbin.1	62.00	Bacteria	10247	1743991
Sample_LCB11.Cluster68	61.10	Clostridiales	18052	1606199
Sample_LCB11.mb.30	58.20	Clostridia	38810	2921045
Sample_LCB11.Cluster4219cbin.1	48.80	Clostridiales	12411	2690878
Sample_LCB11.Cluster626cbin.1	59.70	Clostridiales	42128	2216460
Sample_LCB11.mb.51	47.20	Clostridiales	10772	1851486
Sample_LCB11.mb.34	61.50	Clostridiales	53113	1631854
Sample_LCB11.mb.62	53.20	Clostridia	68512	2053361
Sample_LCB11.mb.36cbin.1	56.90	Clostridiales	63765	1922675
Sample_LCB11.mb.55	48.70	Bacteria	116918	2088177
Sample_LCB11.mb.45	58.40	Clostridiales	59772	1788916
Sample_LCB11.mb.47	38.10	Clostridiales	59098	3365754
Sample_LCB11.Cluster9145cbin.1	37.90	Clostridiales	149552	1983936
Sample_LCB11.Cluster802	59.70	Bifidobacteriaceae	179302	2114414
Sample_LCB11.Cluster1742	55.60	Bacteria	128893	2679939
Sample_LCB11.Cluster7542cbin.1	44.20	Clostridiales	64046	2739724
Sample_LCB11.mb.52	56.10	Clostridiales	95040	2513893
Sample_LCB11.mb.19cbin.1	45.30	Clostridiales	65268	2414135
Sample_LCB11.mb.35cbin.1	40.50	Clostridiales	162494	2128193
Sample_LCB11.Cluster7794cbin.1	41.60	Lachnospiraceae	7349	2461158
Sample_LCB11.mb.77cbin.1	62.10	Clostridiales	14512	2036481
Sample_LCB11.mb.63	57.90	Bacteroidetes	133506	2790653
Sample_LCB11.mb.17	46.80	Bacteroidales	27325	3708846
Sample_LCB11.Cluster6984cbin.1	44.90	Bacteroidales	14388	4377683
Sample_LCB11.Cluster5870cbin.1	45.10	Bacteroidales	107840	4232888
Sample_LCB11.mb.32	42.00	Bacteroidales	113553	4210973
Sample_LCB11.mb.29	57.90	Enterobacteriaceae	93910	4940037
Sample_LCB12.Cluster2504cbin.1	52.20	Clostridiales	76446	2091320
Sample_LCB12.Cluster6052cbin.1	48.30	Clostridia	158541	2297352
Sample_LCB12.mb.110	26.30	Bacteria	131102	1407850
Sample_LCB12.Cluster14970cbin.1	41.30	Clostridiales	176224	2500852
Sample_LCB12.Cluster30	62.30	Clostridiales	13011	1311691
Sample_LCB12.mb.1	53.10	Clostridia	71650	2012453
Sample_LCB12.mb.30	51.60	Bacteria	10582	1567984
Sample_LCB12.mb.42	27.70	Bacteria	131488	1084035
Sample_LCB12.mb.16	49.60	Clostridiales	71437	1950006
Sample_LCB12.mb.113cbin.1	33.30	Clostridiales	119398	2533994
Sample_LCB12.mb.116cbin.1	57.40	Clostridiales	32131	2220605
Sample_LCB12.Cluster8572cbin.1	45.00	Clostridiales	83352	2614980
Sample_LCB12.Cluster347cbin.1	56.00	Bacteroidales	108844	3440613
Sample_LCB12.mb.125cbin.1	60.30	Clostridiales	87352	2002063
Sample_LCB12.Cluster6	63.90	Deltaproteobacteria	16570	2468008
Sample_LCB12.mb.13cbin.1	48.30	Bacteroidales	59154	2310386
Sample_LCB12.Cluster3428cbin.1	52.80	Clostridiales	120267	2357917
Sample_LCB12.Cluster725	56.00	Bacteria	41801	2572337
Sample_LCB12.Cluster10251cbin.1	43.50	Lachnospiraceae	96861	2744737
Sample_LCB12.Cluster11873cbin.1	45.10	Prevotella	56411	3628262
Sample_LCB12.mb.124	36.50	Clostridiales	40762	2103985
Sample_LCB12.mb.105cbin.1	43.80	Clostridiales	315184	3011416
Sample_LCB12.Cluster13525cbin.1	38.10	Lactobacillales	6983	2488958
Sample_LCB12.mb.91	48.50	Clostridiales	39048	1735007

Sample_LCB12.mb.37	42.10	Clostridiales	36863	1841485
Sample_LCB12.mb.101	59.30	Bacteroidetes	16201	2861244
Sample_LCB12.mb.90	60.00	Clostridiales	79945	2021180
Sample_LCB12.mb.45	60.30	Bacteroidetes	39132	2515045
Sample_LCB12.mb.8	58.10	Clostridiales	53790	1906019
Sample_LCB12.mb.55cbin.1	55.90	Clostridiales	11991	2530225
Sample_LCB12.mb.54	41.40	Clostridiales	46499	2579403
Sample_LCB12.mb.75	59.70	Actinobacteria	70866	2250432
Sample_LCB12.mb.77	53.70	Clostridiales	279343	1735675
Sample_LCB12.mb.92	40.90	Lachnospiraceae	103068	2455204
Sample_LCB12.Cluster6103	46.70	Bacteroidales	62543	3863695
Sample_LCB12.mb.32	42.00	Lachnospiraceae	58958	2320027
Sample_LCB12.mb.93	41.50	Clostridiales	7972	1546095
Sample_LCB12.mb.82	60.90	Clostridiales	23934	2168548
Sample_LCB12.mb.80cbin.1	57.60	Bacteroidetes	11938	2310799
Sample_LCB12.mb.7cbin.1	44.60	Clostridiales	139814	2138707
Sample_LCB12.mb.94cbin.1	48.70	Firmicutes	32848	1982379
Sample_LCB12.mb.95	46.90	Clostridiales	11316	1983263
Sample_LCB12.mb.86cbin.1	60.90	Bacteroidetes	155152	2427678
Sample_LCB12.mb.56cbin.1	40.20	Lachnospiraceae	70071	3412427
Sample_LCB12.mb.25	41.90	Bacteroidales	26493	4680058
Sample_LCB12.mb.74	45.50	Bacteroidales	18820	4027950
Sample_LCB12.mb.136cbin.1	50.70	Enterobacteriaceae	26256	4457077
Sample_LCB12.mb.33cbin.1	58.10	Enterobacteriaceae	24234	4854239
Sample_LCB13.Cluster4317cbin.1	50.70	Bacteria	58980	1888374
Sample_LCB13.Cluster6252cbin.1	49.50	Clostridiales	90647	1990980
Sample_LCB13.mb.110	53.60	Clostridia	15763	1710994
Sample_LCB13.mb.12	47.20	Clostridiales	13541	1855355
Sample_LCB13.Cluster228	60.70	Clostridiales	57989	1696363
Sample_LCB13.Cluster308cbin.1	61.30	Clostridiales	54375	1933443
Sample_LCB13.Cluster7562cbin.1	45.00	Clostridiales	14917	2400484
Sample_LCB13.mb.18cbin.1	59.70	Clostridiales	7615	1646051
Sample_LCB13.Cluster12208	38.60	Clostridiales	82967	1789517
Sample_LCB13.Cluster567cbin.1	60.00	Actinobacteria	70038	2210634
Sample_LCB13.Cluster3631	53.70	Clostridiales	337983	1824115
Sample_LCB13.Cluster11305cbin.1	40.70	Clostridiales	183566	2017719
Sample_LCB13.mb.70	26.70	Bacteria	64180	1181145
Sample_LCB13.Cluster799cbin.1	60.60	Clostridiales	45819	2294248
Sample_LCB13.mb.111cbin.1	56.70	Bacteroidales	14956	3106676
Sample_LCB13.Cluster219cbin.1	60.30	Bacteroidetes	42814	2619010
Sample_LCB13.mb.112	31.00	Euryarchaeota	57131	1732112
Sample_LCB13.mb.41	60.80	Clostridiales	55708	1816751
Sample_LCB13.mb.105cbin.1	43.50	Selenomonadales	54179	2509414
Sample_LCB13.mb.46	57.70	Clostridiales	7580	2024998
Sample_LCB13.Cluster10074cbin.1	38.50	Bacteroidales	5868	2782614
Sample_LCB13.mb.27	55.00	Bacteroidetes	34087	2150200
Sample_LCB13.mb.68	59.60	Clostridiales	101035	2315623
Sample_LCB13.mb.72	62.00	Clostridiales	7962	1547102
Sample_LCB13.mb.71	57.30	Clostridiales	30489	2002002
Sample_LCB13.mb.34cbin.1	63.80	Deltaproteobacteria	31232	2525313
Sample_LCB13.mb.15	41.90	Lachnospiraceae	75294	2360135
Sample_LCB13.mb.31	41.80	Lachnospiraceae	95148	2709216
Sample_LCB13.mb.19	43.50	Bacteroidales	141035	2902859
Sample_LCB13.mb.83	60.70	Clostridiales	75324	2302065
Sample_LCB13.Cluster7062	43.10	Bacteria	108883	4129107
Sample_LCB13.mb.98	41.20	Clostridiales	56421	3776850
Sample_LCB13.mb.28	44.50	Bacteroidales	62383	3163495
Sample_LCB13.mb.69	41.00	Lachnospiraceae	19834	2469929

Sample_LCB13.mb.80	59.30	Clostridiales	98360	3295648
Sample_LCB13.mb.9cbin.1	42.60	Clostridiales	100011	2860547
Sample_LCB13.mb.36cbin.1	46.30	Bacteroidales	262056	4259680
Sample_LCB13.mb.43	45.20	Bacteroidales	217776	4361685
Sample_LCB13.mb.60	45.60	Bacteroidales	44700	3815209
Sample_LCB13.Cluster5307cbin.1	50.40	Enterobacteriaceae	43643	4826282
Sample_LCB13.Cluster5606cbin.1	57.70	Enterobacteriaceae	44262	5079241
Sample_LCB14.mb.6	51.80	Clostridia	315384	2412630
Sample_LCB14.Cluster696	51.70	Clostridiales	118493	2267074
Sample_LCB14.mb.43	52.90	Clostridiales	72329	2617531
Sample_LCB14.Cluster2207	46.80	Lactobacillales	177687	1927263
Sample_LCB14.mb.45	59.60	Clostridiales	12617	2815818
Sample_LCB14.Cluster11cbin.1	60.80	Clostridiales	109607	2815778
Sample_LCB14.Cluster1090cbin.1	43.90	Selenomonadales	6458	2013519
Sample_LCB14.mb.4cbin.1	56.10	Clostridiales	15682	2487239
Sample_LCB14.mb.63cbin.1	59.40	Clostridiales	109885	3388768
Sample_LCB14.Cluster704cbin.1	52.70	Lactobacillales	47368	1791918
Sample_LCB14.mb.5	34.50	Lactobacillus	509541	1861641
Sample_LCB14.Cluster16cbin.1	58.10	Clostridiales	20583	1976410
Sample_LCB14.Cluster238cbin.1	53.50	Selenomonadales	51780	2354398
Sample_LCB14.mb.46cbin.1	32.50	Lactobacillales	96019	1917722
Sample_LCB14.mb.55	60.60	Deltaproteobacteria	5707	2960029
Sample_LCB14.mb.65	59.50	Clostridiales	100212	2902613
Sample_LCB14.Cluster4494	28.40	Clostridiales	187541	2831525
Sample_LCB14.mb.52cbin.1	62.20	Clostridiales	13506	3326801
Sample_LCB14.Cluster247	58.40	Bacteroidetes	225227	3257202
Sample_LCB14.mb.36	42.10	Bacteroidales	49411	3314977
Sample_LCB14.mb.21cbin.1	41.60	Lachnospiraceae	80703	2618513
Sample_LCB14.Cluster1916cbin.1	44.20	Bacteroidales	81857	3453759
Sample_LCB14.Cluster1757	48.40	Bacteroidales	258263	3574511
Sample_LCB14.mb.3	46.60	Bacteroidales	574066	3709064
Sample_LCB14.Cluster2653cbin.1	42.10	Bacteroides	56444	4028634
Sample_LCB14.Cluster2742cbin.1	45.10	Bacteroidales	50010	4689736
Sample_LCB14.mb.50	49.90	Clostridiales	307763	5401245
Sample_LCB14.mb.25cbin.1	55.80	Enterobacteriaceae	47018	4360100
Sample_LCB14.mb.37	50.90	Enterobacteriaceae	20080	4414609
Sample_LCB15.mb.1	47.00	Bacteria	53881	1685149
Sample_LCB15.mb.32	33.90	Clostridiales	6875	2735238
Sample_LCB15.mb.3cbin.1	54.80	Bacteroidetes	159168	1897191
Sample_LCB15.mb.28	60.60	Bacteroidetes	11623	2336809
Sample_LCB15.mb.48cbin.1	56.70	Clostridiales	8995	2325982
Sample_LCB15.mb.50cbin.1	41.00	Clostridiales	27598	1926388
Sample_LCB15.mb.24cbin.1	59.70	Clostridiales	30207	2003998
Sample_LCB15.mb.20cbin.1	56.70	Bifidobacteriaceae	24743	2034516
Sample_LCB15.mb.23cbin.1	50.00	Proteobacteria	7803	1650611
Sample_LCB15.mb.18cbin.1	36.20	Clostridiales	14450	2325985
Sample_LCB15.mb.43	41.50	Lachnospiraceae	100999	2481902
Sample_LCB15.Cluster42cbin.1	57.60	Clostridiales	32968	2331672
Sample_LCB15.mb.33	41.60	Lachnospiraceae	17990	3566843
Sample_LCB15.Cluster1020	46.10	Bacteroidales	81797	3390329
Sample_LCB15.mb.31	46.90	Bacteroidales	29301	3863960
Sample_LCB15.mb.49cbin.1	41.40	Bacteroidales	38985	4655238
Sample_LCB15.mb.17cbin.1	50.60	Enterobacteriaceae	141132	4248244
Sample_LCB15.mb.34cbin.1	56.10	Enterobacteriaceae	199617	5336353
Sample_LCB16.mb.50cbin.1	47.80	Bacteria	6346	1366974
Sample_LCB16.mb.28	49.50	Clostridiales	10731	2134180
Sample_LCB16.mb.41cbin.1	63.90	Actinobacteria	7457	2230848
Sample_LCB16.Cluster692cbin.1	54.40	Bacteroidetes	122659	2148788

Sample_LCB16.Cluster819	43.50	Selenomonadales	46875	2278652
Sample_LCB16.Cluster2885	40.90	Clostridiales	166491	2029087
Sample_LCB16.mb.54	60.60	Clostridiales	50263	2181584
Sample_LCB16.Cluster43	60.00	Bifidobacteriaceae	11036	1981749
Sample_LCB16.mb.57	44.30	Clostridiales	10573	2769812
Sample_LCB16.mb.66cbin.1	56.30	Clostridiales	15170	2543339
Sample_LCB16.mb.14	43.20	Lachnospiraceae	89121	2921827
Sample_LCB16.Cluster4450cbin.1	35.90	Clostridiales	203446	2641093
Sample_LCB16.Cluster4274cbin.1	41.10	Lachnospiraceae	68533	2941270
Sample_LCB16.mb.64cbin.1	58.60	Clostridiales	32196	2273536
Sample_LCB16.mb.5cbin.1	42.10	Bacteroidales	13123	3531065
Sample_LCB16.mb.26cbin.1	56.10	Enterobacteriaceae	224240	5506077
Sample_LCB17.Cluster2017	52.00	Bacteria	7148	1493616
Sample_LCB17.Cluster12880cbin.1	36.50	Clostridiales	198573	1846484
Sample_LCB17.Cluster11076cbin.1	38.20	Clostridiales	13376	2745120
Sample_LCB17.mb.100	45.70	Bacteria	74524	1819040
Sample_LCB17.Cluster58	62.20	Clostridiales	11594	1398403
Sample_LCB17.Cluster228	60.80	Clostridiales	74224	1652783
Sample_LCB17.Cluster2810	50.00	Clostridiales	120465	1935426
Sample_LCB17.Cluster5688	46.60	Clostridiales	52015	1748830
Sample_LCB17.mb.10cbin.1	41.70	Clostridiales	166076	2412036
Sample_LCB17.Cluster10309cbin.1	40.50	Clostridiales	151377	1609210
Sample_LCB17.Cluster3559cbin.1	48.70	Firmicutes	32691	1880144
Sample_LCB17.Cluster3277cbin.1	54.30	Bacteroidetes	106339	2180675
Sample_LCB17.Cluster245cbin.1	56.10	Bacteroidales	142495	3440062
Sample_LCB17.mb.11	57.30	Clostridiales	49038	1777147
Sample_LCB17.Cluster13887cbin.1	27.30	Clostridiales	6429	2374355
Sample_LCB17.mb.4	26.60	Bacteria	190494	1457285
Sample_LCB17.Cluster4	63.90	Deltaproteobacteria	13890	2336486
Sample_LCB17.mb.23	37.60	Clostridiales	35192	2047466
Sample_LCB17.mb.27	34.20	Clostridiales	97278	1972067
Sample_LCB17.Cluster5194cbin.1	44.20	Lachnospiraceae	14498	2455558
Sample_LCB17.Cluster14164cbin.1	31.50	Firmicutes	48101	1696314
Sample_LCB17.Cluster4975	45.10	Prevotella	54494	3265146
Sample_LCB17.mb.128	33.80	Clostridiales	59042	1913082
Sample_LCB17.Cluster8299	41.00	Lachnospiraceae	73906	2715894
Sample_LCB17.mb.127	59.60	Bacteroidetes	81606	2642774
Sample_LCB17.mb.126	41.20	Lachnospiraceae	27025	2264068
Sample_LCB17.mb.26	46.30	Bacteroidetes	168034	2386924
Sample_LCB17.mb.131cbin.1	59.60	Bifidobacteriaceae	237625	2063847
Sample_LCB17.Cluster686cbin.1	55.70	Proteobacteria	14602	2644598
Sample_LCB17.mb.67	62.60	Clostridiales	11434	1689052
Sample_LCB17.mb.108	41.30	Bacteroidales	97607	2656792
Sample_LCB17.Cluster12249cbin.1	43.20	Bacteria	50486	4073586
Sample_LCB17.mb.65	45.00	Clostridiales	40875	2444722
Sample_LCB17.mb.106	38.40	Bacteroidales	65148	3314784
Sample_LCB17.mb.24cbin.1	50.40	Bacteroidales	94351	2222837
Sample_LCB17.mb.94cbin.1	58.10	Clostridiales	34322	2978638
Sample_LCB17.mb.83cbin.1	61.70	Clostridiales	25383	2029816
Sample_LCB17.mb.59cbin.1	46.30	Lachnospiraceae	56853	3143189
Sample_LCB17.Cluster5491cbin.1	45.70	Bacteroidales	38582	4022039
Sample_LCB17.mb.21	44.10	Bacteroidales	114987	3164305
Sample_LCB17.mb.88	60.20	Actinobacteria	13940	1982659
Sample_LCB17.mb.71	51.40	Clostridiales	119170	2137253
Sample_LCB17.mb.73	56.10	Clostridiales	14892	2658060
Sample_LCB17.mb.80cbin.1	57.80	Clostridiales	37653	2647039
Sample_LCB17.mb.86cbin.1	38.60	Clostridiales	79815	2166949
Sample_LCB17.mb.84	35.90	Clostridiales	107543	2625302

Sample_LCB17.mb.98	41.10	Clostridiales	157966	2508540
Sample_LCB17.mb.118cbin.1	51.10	Enterobacteriaceae	8689	4059145
Sample_LCB17.mb.81cbin.1	46.10	Bacteroidales	46867	2968979
Sample_LCB17.mb.62	45.50	Bacteroidales	16133	4198527
Sample_LCB19.mb.11cbin.1	46.40	Bacteria	62001	1832259
Sample_LCB19.Cluster50cbin.1	61.70	Clostridiales	164109	1923642
Sample_LCB19.mb.17cbin.1	57.70	Clostridiales	10463	2130822
Sample_LCB19.Cluster1720	47.90	Clostridiales	99248	2936243
Sample_LCB19.mb.91	33.30	Clostridiales	51023	2208546
Sample_LCB19.Cluster2356	45.80	Selenomonadales	137490	1914417
Sample_LCB19.Cluster833	53.90	Clostridiales	616053	1773560
Sample_LCB19.mb.47cbin.1	48.90	Clostridiales	66190	2456082
Sample_LCB19.Cluster1786	43.40	Selenomonadales	84617	2336315
Sample_LCB19.Cluster23cbin.1	60.40	Bacteroidetes	9135	2378820
Sample_LCB19.Cluster270cbin.1	56.00	Proteobacteria	15408	2010738
Sample_LCB19.Cluster4285cbin.1	40.90	Clostridiales	185466	2102036
Sample_LCB19.mb.44cbin.1	52.20	Clostridiales	10206	1800466
Sample_LCB19.mb.49	56.50	Bifidobacteriaceae	74997	1677283
Sample_LCB19.Cluster3608	42.70	Clostridiales	119580	2608355
Sample_LCB19.mb.65	59.80	Clostridiales	34141	1699243
Sample_LCB19.mb.58	44.10	Clostridiales	20793	3156284
Sample_LCB19.Cluster2409cbin.1	43.70	Bacteroidales	8873	2332719
Sample_LCB19.mb.34	43.70	Lachnospiraceae	101032	2829983
Sample_LCB19.mb.87	56.60	Clostridiales	15626	2320415
Sample_LCB19.mb.38	26.90	Clostridiales	130845	2441050
Sample_LCB19.mb.67	45.40	Clostridiales	46544	2463242
Sample_LCB19.mb.75cbin.1	36.00	Clostridiales	186246	2515815
Sample_LCB19.Cluster4209cbin.1	39.70	Lachnospiraceae	90239	3191376
Sample_LCB19.Cluster3027cbin.1	42.10	Bacteroidales	63697	3349357
Sample_LCB19.mb.41	41.90	Lachnospiraceae	13289	2204236
Sample_LCB19.mb.1	42.50	Bacteroides	49334	4034019
Sample_LCB19.mb.61cbin.1	42.30	Lachnospiraceae	93657	3991174
Sample_LCB19.Cluster959cbin.1	45.10	Bacteroidales	52648	4618596
Sample_LCB19.mb.5	56.10	Enterobacteriaceae	177856	5416561
Sample_LCB20.Cluster10120	26.40	Bacteria	134571	1179179
Sample_LCB20.Cluster9862	29.30	Bacteria	223391	1134637
Sample_LCB20.mb.6	26.00	Bacteria	120217	1362353
Sample_LCB20.mb.46	61.20	Clostridiales	5328	1696126
Sample_LCB20.Cluster531cbin.1	56.20	Clostridiales	43608	2575652
Sample_LCB20.mb.31cbin.1	61.80	Clostridiales	66616	1535673
Sample_LCB20.mb.55cbin.1	58.10	Clostridiales	45421	3068008
Sample_LCB20.mb.32	55.00	Clostridiales	20214	2399220
Sample_LCB20.mb.17	58.80	Clostridiales	23781	2472777
Sample_LCB20.Cluster3757cbin.1	44.20	Selenomonadales	8517	1946320
Sample_LCB20.mb.52cbin.1	57.30	Clostridiales	91411	1886604
Sample_LCB20.mb.37	37.00	Clostridiales	6091	1416682
Sample_LCB20.mb.78	60.70	Clostridiales	16523	1753659
Sample_LCB20.Cluster7747	40.50	Clostridiales	142399	1948185
Sample_LCB20.mb.10cbin.1	44.60	Clostridiales	152324	1950640
Sample_LCB20.Cluster552cbin.1	50.90	Prevotella	68717	2440384
Sample_LCB20.mb.64	42.00	Clostridiales	6686	2253593
Sample_LCB20.mb.20	60.20	Bacteroidetes	39879	2485528
Sample_LCB20.Cluster3414	51.30	Clostridiales	141281	2259110
Sample_LCB20.mb.16	43.10	Clostridiales	40580	2571986
Sample_LCB20.mb.68cbin.1	55.80	Clostridiales	128792	2489224
Sample_LCB20.mb.79cbin.1	50.00	Clostridiales	130137	2280730
Sample_LCB20.mb.19	58.90	Bacteroidetes	112417	2660610
Sample_LCB20.mb.65	54.80	Bacteroidetes	123145	2190388

Sample_LCB20.mb.54cbin.1	59.40	Bacteroidetes	55103	2876977
Sample_LCB20.Cluster5370cbin.1	43.40	Bacteroidales	259499	3163922
Sample_LCB20.Cluster5551cbin.1	42.70	Bacteria	72926	4039252
Sample_LCB20.Cluster6220cbin.1	44.90	Bacteroidales	11114	4431612
Sample_LCB20.mb.30	45.80	Bacteroidales	130825	4231455
Sample_LCB20.mb.23cbin.1	42.10	Bacteroidales	38405	4251758
Sample_LCB20.mb.83	47.10	Bacteroidales	27260	3825602
Sample_LCB20.Cluster2174	50.80	Enterobacteriaceae	161312	4295585
Sample_LCB20.mb.3	57.60	Enterobacteriaceae	225948	4627972
Sample_LCB21.Cluster12270cbin.1	26.70	Bacteria	10680	1141058
Sample_LCB21.Cluster33cbin.1	59.70	Actinobacteria	5777	1557059
Sample_LCB21.mb.108	49.50	Clostridiales	96599	1996032
Sample_LCB21.Cluster348	61.10	Clostridiales	55049	1589597
Sample_LCB21.Cluster3886cbin.1	48.40	Firmicutes	11853	1589466
Sample_LCB21.mb.18cbin.1	50.20	Bacteroidales	74863	2492854
Sample_LCB21.Cluster3188cbin.1	53.80	Clostridiales	260816	1851801
Sample_LCB21.Cluster771cbin.1	57.50	Clostridiales	40857	2924079
Sample_LCB21.mb.95cbin.1	53.70	Clostridia	35872	1738217
Sample_LCB21.Cluster9179	40.90	Clostridiales	189060	2016579
Sample_LCB21.Cluster6899cbin.1	45.00	Clostridiales	14677	2360197
Sample_LCB21.mb.56cbin.1	56.60	Clostridiales	18125	2473721
Sample_LCB21.Cluster7124cbin.1	43.10	Clostridiales	34664	3338860
Sample_LCB21.mb.76	34.00	Bacteria	31023	1814203
Sample_LCB21.mb.98cbin.1	57.00	Clostridiales	59461	2288093
Sample_LCB21.mb.77	41.10	Lachnospiraceae	111145	2820725
Sample_LCB21.mb.50cbin.1	51.20	Clostridiales	148149	2331023
Sample_LCB21.mb.70	48.90	Clostridiales	54659	2762195
Sample_LCB21.Cluster4525cbin.1	48.40	Proteobacteria	54778	2587753
Sample_LCB21.mb.102cbin.1	58.30	Bacteroidetes	148457	3173790
Sample_LCB21.mb.36cbin.1	40.30	Lachnospiraceae	26215	2980818
Sample_LCB21.Cluster8836cbin.1	39.90	Lachnospiraceae	36900	3764945
Sample_LCB21.Cluster84cbin.1	59.80	Clostridiales	44941	4347472
Sample_LCB21.mb.9cbin.1	43.80	Selenomonadales	43526	2290274
Sample_LCB21.mb.91	44.50	Clostridiales	144056	2241599
Sample_LCB21.mb.96	62.50	Deltaproteobacteria	39632	3578815
Sample_LCB21.mb.53cbin.1	42.00	Clostridiales	22644	3178796
Sample_LCB21.Cluster7184cbin.1	45.20	Bacteroidales	47818	4522414
Sample_LCB21.mb.68	46.80	Bacteroidales	33998	3980741
Sample_LCB21.mb.87	45.80	Bacteroidales	32092	3680485
Sample_LCB21.mb.66	41.70	Bacteroidales	35188	4634548
Sample_LCB21.Cluster4135cbin.1	50.70	Enterobacteriaceae	93850	4859998
Sample_LCB22.Cluster2302cbin.1	50.80	Bacteria	89434	1870211
Sample_LCB22.mb.44	59.00	Bacteria	29580	2310456
Sample_LCB22.Cluster11885	32.80	Bacteria	135835	1706399
Sample_LCB22.mb.4cbin.1	49.60	Clostridiales	94580	1918936
Sample_LCB22.Cluster494cbin.1	56.00	Clostridiales	30229	2919499
Sample_LCB22.mb.92	26.70	Bacteria	43962	1314793
Sample_LCB22.Cluster190cbin.1	59.80	Actinobacteria	19797	2035613
Sample_LCB22.mb.15	58.20	Clostridiales	29195	2199584
Sample_LCB22.mb.7	58.10	Clostridiales	40379	3108703
Sample_LCB22.Cluster4801cbin.1	46.70	Lachnospiraceae	57313	2710050
Sample_LCB22.Cluster3595	47.30	Selenomonadales	84326	2132392
Sample_LCB22.mb.50cbin.1	60.00	Clostridiales	101268	2195657
Sample_LCB22.Cluster6899	41.50	Clostridiales	475347	1748462
Sample_LCB22.mb.16	57.90	Clostridiales	56343	2882242
Sample_LCB22.Cluster127	55.40	Bacteria	318695	2729157
Sample_LCB22.mb.31	59.20	Clostridiales	11489	2095898
Sample_LCB22.Cluster9445	38.60	Clostridiales	142468	2152762

Sample_LCB22.mb.10	41.30	Clostridiales	239689	2549019
Sample_LCB22.mb.64cbin.1	54.90	Bacteroidetes	32940	1902889
Sample_LCB22.Cluster10629cbin.1	36.80	Clostridiales	10287	2878026
Sample_LCB22.Cluster7041cbin.1	41.30	Lachnospiraceae	69121	2900621
Sample_LCB22.mb.24	36.00	Clostridiales	185114	2480657
Sample_LCB22.Cluster5886	42.20	Bacteroidales	66368	3647458
Sample_LCB22.mb.74	61.60	Proteobacteria	39535	2481450
Sample_LCB22.mb.58	41.80	Lachnospiraceae	54199	2729860
Sample_LCB22.mb.71cbin.1	44.90	Prevotella	34048	3415741
Sample_LCB22.Cluster2133cbin.1	41.60	Bacteroidales	38369	3846829
Sample_LCB22.Cluster4267	45.30	Bacteroidales	73136	4032948
Sample_LCB22.mb.63cbin.1	48.70	Bacteroidales	25935	3457769
Sample_LCB22.mb.13cbin.1	44.70	Bacteroidales	46754	4871693
Sample_LCB22.mb.91cbin.1	42.80	Bacteria	50071	4299039
Sample_LCB22.mb.46cbin.1	50.50	Enterobacteriaceae	172334	4925115
Sample_LCB23.Cluster8702	38.20	Clostridiales	143330	2022625
Sample_LCB23.Cluster351cbin.1	58.10	Clostridiales	70927	2074890
Sample_LCB23.Cluster597cbin.1	55.70	Clostridiales	57418	2921785
Sample_LCB23.Cluster4176cbin.1	47.60	Selenomonadales	25954	1949061
Sample_LCB23.Cluster153	59.70	Actinobacteria	120885	2171913
Sample_LCB23.Cluster1202cbin.1	51.60	Clostridiales	23368	2008073
Sample_LCB23.mb.15cbin.1	51.60	Bacteroidales	73070	2269875
Sample_LCB23.Cluster750cbin.1	54.40	Bacteroidetes	76463	2218040
Sample_LCB23.Cluster5199	44.40	Clostridiales	159896	2496872
Sample_LCB23.Cluster540	55.60	Bacteria	43031	2672904
Sample_LCB23.Cluster10099cbin.1	37.70	Clostridiales	10097	2093825
Sample_LCB23.Cluster7723	40.80	Clostridiales	125240	2069378
Sample_LCB23.Cluster10438	28.20	Bacteria	31318	2432101
Sample_LCB23.mb.5cbin.1	49.10	Clostridiales	34982	2575318
Sample_LCB23.Cluster37	63.10	Proteobacteria	47329	2586047
Sample_LCB23.mb.87cbin.1	61.90	Clostridiales	7334	2142530
Sample_LCB23.mb.35	60.20	Clostridiales	38753	2373319
Sample_LCB23.Cluster527	53.80	Selenomonadales	41110	2096302
Sample_LCB23.Cluster6112cbin.1	49.80	Bacteroidales	65029	2379354
Sample_LCB23.Cluster647cbin.1	55.30	Bacteroidales	58120	2691326
Sample_LCB23.mb.72	43.80	Lachnospiraceae	101291	2632317
Sample_LCB23.mb.67cbin.1	44.60	Clostridiales	86271	3021678
Sample_LCB23.mb.73cbin.1	41.30	Lachnospiraceae	53658	3078908
Sample_LCB23.mb.90cbin.1	41.70	Lachnospiraceae	74821	2536536
Sample_LCB23.Cluster2610	45.40	Bacteroidales	92210	4133510
Sample_LCB23.Cluster3712cbin.1	41.60	Bacteroidales	36346	5213647
Sample_LCB23.mb.20cbin.1	50.90	Enterobacteriaceae	43085	4645024
Sample_LCB24.Cluster810	51.90	Clostridiales	16165	1810097
Sample_LCB24.Cluster528cbin.1	54.60	Bacteroidetes	26990	2051829
Sample_LCB24.mb.60cbin.1	61.00	Clostridiales	27756	2112230
Sample_LCB24.Cluster1115cbin.1	48.30	Firmicutes	38337	1928170
Sample_LCB24.Cluster162cbin.1	56.40	Clostridiales	14335	2439074
Sample_LCB24.Cluster253cbin.1	60.50	Bifidobacteriaceae	156276	2222386
Sample_LCB24.Cluster3220cbin.1	44.30	Clostridiales	194490	2201226
Sample_LCB24.mb.56	59.90	Actinobacteria	40657	2136311
Sample_LCB24.Cluster7479	32.50	Lactobacillales	133187	1863016
Sample_LCB24.Cluster52	62.40	Proteobacteria	52753	2404002
Sample_LCB24.Cluster4368cbin.1	41.20	Lachnospiraceae	86249	2962799
Sample_LCB24.mb.61	52.80	Lactobacillales	55907	1802948
Sample_LCB24.mb.70cbin.1	62.90	Bifidobacteriaceae	71337	2107120
Sample_LCB24.Cluster4504cbin.1	43.10	Bacteria	7521	3466620
Sample_LCB24.Cluster5212cbin.1	38.40	Bacteroidales	36250	3431763
Sample_LCB24.mb.77	34.70	Lactobacillus	43056	1848855

Sample_LCB24.mb.81cbin.1	53.90	Selenomonadales	75654	2201240
Sample_LCB24.mb.50	41.80	Lachnospiraceae	16956	2274217
Sample_LCB24.mb.7	58.70	Bifidobacteriaceae	34908	2364407
Sample_LCB24.mb.71cbin.1	45.10	Bacteroidales	37520	4506993
Sample_LCB24.mb.17cbin.1	51.00	Enterobacteriaceae	15823	4326959
Sample_LCB25.mb.71cbin.1	48.50	Clostridiales	10115	2602752
Sample_LCB25.Cluster768cbin.1	56.80	Clostridiales	6846	2086526
Sample_LCB25.Cluster4305cbin.1	38.20	Clostridiales	7463	1639153
Sample_LCB25.mb.21cbin.1	49.00	Clostridiales	29982	2670304
Sample_LCB25.Cluster468	54.60	Bacteroidetes	84968	2023823
Sample_LCB25.Cluster2594	43.40	Selenomonadales	149110	2518368
Sample_LCB25.Cluster6031cbin.1	27.20	Clostridiales	9016	2235391
Sample_LCB25.Cluster2cbin.1	57.40	Clostridiales	24645	2511172
Sample_LCB25.mb.17	61.80	Proteobacteria	13948	2276644
Sample_LCB25.mb.59	60.10	Clostridiales	55393	2547815
Sample_LCB25.mb.25	36.00	Clostridiales	80029	2421683
Sample_LCB25.mb.72	40.60	Clostridiales	167673	2046761
Sample_LCB25.Cluster14	60.10	Bifidobacteriaceae	302703	2419512
Sample_LCB25.mb.65	59.30	Bacteroidetes	158360	2793500
Sample_LCB25.mb.70cbin.1	45.10	Prevotella	32221	3278494
Sample_LCB25.mb.20cbin.1	45.20	Bacteroidales	71427	3146907
Sample_LCB25.mb.22	41.30	Lachnospiraceae	54140	3093038
Sample_LCB25.Cluster6084	29.10	Bacteria	85458	2599744
Sample_LCB25.mb.45	43.30	Bacteroidales	47296	2946997
Sample_LCB25.mb.58cbin.1	40.50	Lachnospiraceae	44847	3448588
Sample_LCB25.mb.2cbin.1	31.20	Firmicutes	53025	2589349
Sample_LCB25.mb.10	42.70	Lachnospiraceae	93071	3595386
Sample_LCB25.Cluster4572cbin.1	43.20	Bacteria	60765	4068629
Sample_LCB25.Cluster1584cbin.1	45.60	Bacteroidales	29692	4096718
Sample_LCB25.Cluster779cbin.1	50.80	Enterobacteriaceae	79427	4537440
Sample_LCC09.Cluster2953cbin.1	52.80	Clostridia	149944	1708442
Sample_LCC09.Cluster1771cbin.1	52.90	Clostridia	19705	2023090
Sample_LCC09.Cluster10253cbin.1	43.40	Clostridiales	13997	2725867
Sample_LCC09.mb.103cbin.1	52.40	Clostridiales	76589	2041163
Sample_LCC09.Cluster1325cbin.1	57.90	Clostridiales	27002	2117310
Sample_LCC09.Cluster32cbin.1	58.80	Clostridiales	18316	2319374
Sample_LCC09.mb.108cbin.1	56.50	Clostridiales	60233	2545679
Sample_LCC09.Cluster1576cbin.1	56.30	Clostridiales	15284	2304370
Sample_LCC09.Cluster1143cbin.1	58.70	Clostridiales	17954	2232474
Sample_LCC09.mb.110cbin.1	47.90	Clostridiales	75738	3035994
Sample_LCC09.mb.125	49.50	Clostridiales	24943	2436596
Sample_LCC09.Cluster11861	32.50	Lactobacillales	122318	1838793
Sample_LCC09.Cluster2611	50.50	Lactobacillales	38711	1858651
Sample_LCC09.mb.106cbin.1	53.60	Clostridiales	170296	1883584
Sample_LCC09.Cluster7300cbin.1	43.90	Clostridiales	20624	3467720
Sample_LCC09.Cluster9141	40.90	Clostridiales	190699	2057738
Sample_LCC09.mb.29cbin.1	46.30	Clostridiales	46258	1912186
Sample_LCC09.mb.59	49.40	Clostridiales	86241	1952900
Sample_LCC09.Cluster8951cbin.1	37.60	Clostridiales	11641	2498171
Sample_LCC09.mb.18	44.50	Clostridiales	16420	2444452
Sample_LCC09.mb.46cbin.1	61.00	Clostridiales	72226	1905653
Sample_LCC09.mb.128	43.70	Lachnospiraceae	103337	2717194
Sample_LCC09.mb.137cbin.1	56.40	Bifidobacteriaceae	100393	2117585
Sample_LCC09.Cluster898	58.70	Bacteroidetes	130202	2917634
Sample_LCC09.mb.49cbin.1	60.80	Clostridiales	80860	2216927
Sample_LCC09.mb.1cbin.1	42.80	Clostridiales	141262	2642818
Sample_LCC09.mb.41cbin.1	60.50	Clostridiales	51372	2251658
Sample_LCC09.Cluster5114cbin.1	44.30	Bacteroidales	141285	3502765

Sample_LCC09.mb.96	34.20	Clostridiales	15202	2216342
Sample_LCC09.mb.79	57.60	Clostridiales	7757	2384848
Sample_LCC09.Cluster2591cbin.1	45.50	Bacteroidales	102023	3918491
Sample_LCC09.mb.93	37.00	Clostridiales	44049	2566091
Sample_LCC09.mb.6	43.50	Selenomonadales	13507	2058110
Sample_LCC09.mb.45	58.60	Bifidobacteriaceae	48811	2210990
Sample_LCC09.mb.27cbin.1	48.30	Proteobacteria	45833	2814194
Sample_LCC09.Cluster6301cbin.1	42.30	Bacteria	100817	4481218
Sample_LCC09.mb.60cbin.1	62.80	Bifidobacteriaceae	110749	2055075
Sample_LCC09.mb.20	46.80	Bacteroidales	93408	3322610
Sample_LCC09.mb.56cbin.1	40.50	Clostridiales	101806	2727638
Sample_LCC09.mb.104	43.10	Bacteroidales	177623	4897423
Sample_LCC09.mb.61cbin.1	42.20	Lachnospiraceae	67224	2561740
Sample_LCC09.mb.64cbin.1	40.80	Lachnospiraceae	78145	3198017
Sample_LCC09.mb.77	41.70	Lachnospiraceae	39143	2294816
Sample_LCC10.Cluster8948	26.00	Bacteria	307090	1298271
Sample_LCC10.mb.16	47.10	Clostridiales	18565	1960328
Sample_LCC10.Cluster705cbin.1	58.50	Clostridiales	6794	1843577
Sample_LCC10.Cluster634cbin.1	56.20	Clostridiales	59766	2332847
Sample_LCC10.mb.70	52.70	Clostridia	81906	2165451
Sample_LCC10.mb.101cbin.1	56.50	Clostridiales	53376	2758012
Sample_LCC10.Cluster2822cbin.1	46.30	Clostridiales	10167	2074225
Sample_LCC10.mb.75	49.40	Clostridiales	86195	1946110
Sample_LCC10.mb.60cbin.1	38.10	Clostridiales	22967	3264625
Sample_LCC10.mb.46	60.90	Clostridiales	59020	2260586
Sample_LCC10.Cluster99	58.90	Clostridiales	15103	1920760
Sample_LCC10.Cluster1836cbin.1	48.60	Firmicutes	32227	1675142
Sample_LCC10.Cluster742	58.10	Bifidobacteriaceae	107423	2034984
Sample_LCC10.mb.65cbin.1	48.50	Clostridiales	14820	2752416
Sample_LCC10.mb.102	60.80	Clostridiales	20572	2280872
Sample_LCC10.Cluster6772cbin.1	40.90	Clostridiales	175133	2058909
Sample_LCC10.Cluster620cbin.1	59.50	Bacteroidetes	62337	2773754
Sample_LCC10.mb.14cbin.1	44.10	Lachnospiraceae	88699	2227666
Sample_LCC10.mb.47	56.50	Clostridiales	7656	2251120
Sample_LCC10.Cluster272	55.60	Bacteria	118198	2613506
Sample_LCC10.mb.93	53.60	Clostridiales	27879	1781387
Sample_LCC10.Cluster8756cbin.1	32.40	Lactobacillales	129010	1844264
Sample_LCC10.Cluster1767cbin.1	50.30	Lactobacillales	22395	1893472
Sample_LCC10.Cluster6632cbin.1	37.50	Clostridiales	7899	2490530
Sample_LCC10.mb.63cbin.1	51.50	Clostridiales	200348	2418680
Sample_LCC10.mb.29	58.80	Bacteroidetes	225759	2797902
Sample_LCC10.mb.82cbin.1	53.70	Clostridiales	280656	1882672
Sample_LCC10.mb.84cbin.1	40.70	Lachnospiraceae	65232	3130578
Sample_LCC10.Cluster5474cbin.1	44.80	Bacteroidales	139091	3847821
Sample_LCC10.mb.89cbin.1	42.30	Clostridiales	142711	2785753
Sample_LCC10.Cluster3676cbin.1	46.50	Bacteroidales	100989	4045239
Sample_LCC10.mb.83	48.60	Proteobacteria	35680	2663607
Sample_LCC10.Cluster7124cbin.1	42.00	Bacteroidales	71753	4656423
Sample_LCC10.mb.50cbin.1	42.30	Bacteria	27788	4370880
Sample_LCC10.mb.38cbin.1	51.50	Enterobacteriaceae	6992	3558113
Sample_LCC10.mb.95	45.50	Bacteroidales	97398	3451248
Sample_LCC10.mb.98cbin.1	42.60	Bacteroides	57840	5821047
Sample_LCC11.Cluster6383	49.60	Clostridiales	82763	1914889
Sample_LCC11.Cluster3915cbin.1	52.90	Clostridia	58693	2076813
Sample_LCC11.mb.16cbin.1	62.40	Clostridiales	9236	1605131
Sample_LCC11.mb.47cbin.1	49.10	Bacteria	8791	1849463
Sample_LCC11.mb.41cbin.1	57.20	Clostridia	30153	3432942
Sample_LCC11.mb.11	56.90	Clostridiales	71322	1805150

Sample_LCC11.mb.64	58.40	Clostridiales	7337	2597497
Sample_LCC11.mb.33	62.60	Clostridiales	24173	2078513
Sample_LCC11.mb.52	60.80	Clostridiales	74942	1860481
Sample_LCC11.Cluster11053	37.80	Clostridiales	90195	1934951
Sample_LCC11.Cluster25cbin.1	63.60	Actinobacteria	26723	2745402
Sample_LCC11.Cluster10094cbin.1	40.40	Clostridiales	162611	2120593
Sample_LCC11.Cluster10060cbin.1	40.90	Clostridiales	66410	2519054
Sample_LCC11.mb.62cbin.1	56.60	Clostridiales	19941	2312076
Sample_LCC11.Cluster5200	51.20	Clostridiales	126921	2192541
Sample_LCC11.Cluster2495	55.60	Bacteria	128893	2694365
Sample_LCC11.mb.70	61.30	Clostridiales	38135	1995624
Sample_LCC11.mb.29	58.50	Bacteroidetes	20355	2670779
Sample_LCC11.mb.9cbin.1	62.20	Clostridiales	10362	1551102
Sample_LCC11.Cluster937	59.80	Bifidobacteriaceae	186628	2095967
Sample_LCC11.Cluster8812	43.90	Clostridiales	202453	2904802
Sample_LCC11.Cluster8336cbin.1	41.50	Lachnospiraceae	7609	2895768
Sample_LCC11.mb.80	55.70	Clostridiales	11667	3430531
Sample_LCC11.mb.54cbin.1	41.70	Lachnospiraceae	88545	2538937
Sample_LCC11.Cluster6780	45.50	Bacteroidales	107689	3877327
Sample_LCC11.mb.2	46.60	Bacteroidales	26615	3954806
Sample_LCC11.mb.10	42.00	Bacteroidales	106765	4201251
Sample_LCC12.Cluster3883	50.70	Bacteria	44470	1857580
Sample_LCC12.Cluster3277	53.30	Clostridia	84701	1814979
Sample_LCC12.mb.114	26.40	Bacteria	77177	1265566
Sample_LCC12.Cluster5768	48.70	Clostridia	251872	2050333
Sample_LCC12.mb.20	27.60	Bacteria	112929	1119309
Sample_LCC12.Cluster12140	41.90	Clostridiales	179624	2165979
Sample_LCC12.mb.104	60.00	Clostridia	6765	2342244
Sample_LCC12.Cluster15287cbin.1	33.30	Clostridiales	109779	2638858
Sample_LCC12.mb.117	48.40	Clostridiales	25315	1769813
Sample_LCC12.Cluster59cbin.1	61.70	Clostridiales	11587	1703107
Sample_LCC12.Cluster836cbin.1	59.20	Clostridiales	39696	1920425
Sample_LCC12.Cluster8334cbin.1	45.10	Clostridiales	80905	2588384
Sample_LCC12.Cluster1827	55.30	Bacteroidetes	49349	1837110
Sample_LCC12.mb.103cbin.1	56.70	Clostridiales	83732	2028878
Sample_LCC12.Cluster3878cbin.1	48.70	Firmicutes	32623	1944133
Sample_LCC12.mb.34cbin.1	57.10	Clostridiales	13842	1763225
Sample_LCC12.Cluster3959	52.50	Clostridiales	228685	2020904
Sample_LCC12.mb.111	59.60	Actinobacteria	57170	2204036
Sample_LCC12.mb.91cbin.1	57.10	Bacteria	35449	2584759
Sample_LCC12.mb.13	56.90	Bacteroidales	9972	2991357
Sample_LCC12.mb.108cbin.1	49.30	Bacteroidales	37523	2942970
Sample_LCC12.mb.58	62.50	Clostridiales	49127	1814967
Sample_LCC12.mb.16cbin.1	53.90	Clostridiales	177579	1908687
Sample_LCC12.mb.119	41.00	Clostridiales	16890	1932824
Sample_LCC12.mb.62cbin.1	60.50	Clostridiales	64906	1814409
Sample_LCC12.mb.19cbin.1	61.50	Bacteroidetes	101450	2346464
Sample_LCC12.mb.42	56.30	Bacteria	7581	2217725
Sample_LCC12.mb.24	44.40	Clostridiales	194373	2590262
Sample_LCC12.Cluster4565cbin.1	45.20	Bacteroidales	12850	2889434
Sample_LCC12.mb.29	41.80	Lachnospiraceae	47095	2604868
Sample_LCC12.Cluster9948cbin.1	44.80	Prevotella	55804	3743259
Sample_LCC12.mb.99	56.60	Clostridiales	36580	2533175
Sample_LCC12.mb.49cbin.1	44.50	Clostridiales	67809	2173377
Sample_LCC12.mb.6	43.70	Lachnospiraceae	96861	2475338
Sample_LCC12.mb.69	56.50	Clostridiales	12815	2441273
Sample_LCC12.mb.72	60.30	Clostridiales	21851	2536755
Sample_LCC12.mb.77	42.30	Clostridiales	56713	1819656

Sample_LCC12.mb.47cbin.1	42.20	Lachnospiraceae	42621	2331428
Sample_LCC12.mb.81	60.40	Bacteroidetes	29530	2629684
Sample_LCC12.mb.22	41.90	Bacteroidales	24006	4743012
Sample_LCC12.mb.35cbin.1	46.50	Bacteroidales	96580	4557669
Sample_LCC12.Cluster134cbin.1	57.90	Enterobacteriaceae	32226	4987753
Sample_LCC12.mb.68cbin.1	50.50	Enterobacteriaceae	26105	4603325
Sample_LCC13.Cluster3327cbin.1	50.60	Bacteria	62632	1902653
Sample_LCC13.mb.15	42.20	Bacteria	56166	4516768
Sample_LCC13.mb.10cbin.1	50.10	Clostridiales	90647	2080467
Sample_LCC13.Cluster148cbin.1	60.90	Clostridiales	22345	1747370
Sample_LCC13.Cluster203cbin.1	61.90	Clostridiales	48714	1747872
Sample_LCC13.mb.24	58.10	Clostridiales	43532	2989030
Sample_LCC13.mb.102cbin.1	60.40	Clostridiales	42688	1914889
Sample_LCC13.mb.107cbin.1	62.10	Clostridiales	40462	1851732
Sample_LCC13.mb.108cbin.1	57.20	Clostridiales	86306	1876651
Sample_LCC13.mb.2	61.20	Clostridiales	66447	2172081
Sample_LCC13.Cluster521cbin.1	59.90	Actinobacteria	58094	2237006
Sample_LCC13.mb.22	45.00	Clostridiales	9864	2219420
Sample_LCC13.Cluster1228cbin.1	54.80	Bacteroidetes	42952	2139611
Sample_LCC13.Cluster2671	53.60	Clostridiales	268357	1917306
Sample_LCC13.mb.29	57.20	Clostridiales	39290	1897324
Sample_LCC13.Cluster13355	31.00	Euryarchaeota	129264	1769319
Sample_LCC13.Cluster9468cbin.1	38.90	Clostridiales	60241	1897139
Sample_LCC13.mb.20cbin.1	43.30	Lachnospiraceae	51169	2852337
Sample_LCC13.Cluster283cbin.1	52.00	Clostridiales	151068	2213166
Sample_LCC13.mb.18	58.90	Bacteroidetes	102352	2283966
Sample_LCC13.Cluster6965cbin.1	42.70	Lachnospiraceae	7730	2036268
Sample_LCC13.Cluster9914	40.60	Clostridiales	129680	2112503
Sample_LCC13.mb.1cbin.1	56.60	Clostridiales	8873	2234672
Sample_LCC13.Cluster19cbin.1	63.30	Deltaproteobacteria	76963	2640469
Sample_LCC13.Cluster477cbin.1	59.90	Bifidobacteriaceae	95754	2484549
Sample_LCC13.Cluster8306	43.00	Clostridiales	137539	2663979
Sample_LCC13.mb.71cbin.1	52.60	Clostridiales	60406	2242386
Sample_LCC13.mb.53cbin.1	59.20	Clostridiales	6661	1771870
Sample_LCC13.mb.3	59.50	Clostridiales	61726	3281891
Sample_LCC13.mb.39cbin.1	43.40	Selenomonadales	45833	2287967
Sample_LCC13.mb.36cbin.1	29.10	Bacteria	47344	2674602
Sample_LCC13.mb.74	56.70	Clostridiales	19595	2154466
Sample_LCC13.Cluster5527	43.20	Bacteria	108883	4032520
Sample_LCC13.mb.25	43.30	Bacteroidales	140191	2853314
Sample_LCC13.mb.60	60.40	Clostridiales	22747	2459586
Sample_LCC13.mb.42cbin.1	57.80	Clostridiales	27373	3442718
Sample_LCC13.mb.69	59.60	Bacteroidetes	77820	2654863
Sample_LCC13.mb.96	59.80	Clostridiales	44257	2270287
Sample_LCC13.mb.93	57.00	Bacteroidales	21169	2984610
Sample_LCC13.Cluster8065cbin.1	42.00	Bacteroidales	21454	4638298
Sample_LCC13.mb.99cbin.1	60.30	Bacteroidetes	33135	2554829
Sample_LCC13.mb.30	45.20	Bacteroidales	188570	4206746
Sample_LCC13.mb.75	41.90	Lachnospiraceae	68745	2384796
Sample_LCC13.mb.47	45.60	Bacteroidales	63462	3557288
Sample_LCC13.Cluster2164cbin.1	50.50	Enterobacteriaceae	77846	4845843
Sample_LCC13.mb.70	46.30	Bacteroidales	231698	4251920
Sample_LCC13.mb.91cbin.1	58.40	Enterobacteriaceae	6769	3965664
Sample_LCC14.Cluster1275	51.90	Clostridia	379506	2403182
Sample_LCC14.Cluster2550	46.90	Lactobacillales	177687	1867135
Sample_LCC14.Cluster964	52.30	Clostridiales	108958	2272042
Sample_LCC14.mb.40	53.10	Clostridiales	72073	2520225
Sample_LCC14.mb.3	60.40	Clostridiales	138871	2880441

Sample_LCC14.mb.37	61.30	Clostridiales	75898	2610633
Sample_LCC14.mb.50cbin.1	49.50	Clostridiales	8806	3972058
Sample_LCC14.Cluster677cbin.1	60.70	Bifidobacteriaceae	5291	1912199
Sample_LCC14.Cluster315cbin.1	56.10	Clostridiales	15820	2547486
Sample_LCC14.mb.58cbin.1	43.80	Selenomonadales	9735	2178779
Sample_LCC14.Cluster994	52.70	Lactobacillales	45338	1800233
Sample_LCC14.mb.10	60.60	Clostridiales	45880	1786743
Sample_LCC14.mb.23cbin.1	53.80	Selenomonadales	36362	2336749
Sample_LCC14.mb.42cbin.1	32.60	Lactobacillales	122067	1921624
Sample_LCC14.mb.83	34.50	Lactobacillus	509809	1861909
Sample_LCC14.mb.62cbin.1	59.40	Clostridiales	92214	3029175
Sample_LCC14.Cluster234	58.20	Bacteroidetes	257562	3304664
Sample_LCC14.mb.48cbin.1	41.80	Lachnospiraceae	79622	2448517
Sample_LCC14.mb.81cbin.1	62.50	Clostridiales	12772	3273092
Sample_LCC14.Cluster2264cbin.1	44.40	Bacteroidales	89503	3109750
Sample_LCC14.mb.14cbin.1	49.40	Clostridiales	15899	6180614
Sample_LCC14.Cluster2175	48.40	Bacteroidales	260184	3594561
Sample_LCC14.mb.64cbin.1	44.90	Bacteroidales	58400	4713793
Sample_LCC14.Cluster2329cbin.1	49.70	Clostridiales	339032	5604247
Sample_LCC14.mb.27	55.60	Enterobacteriaceae	40935	4551345
Sample_LCC14.mb.72cbin.1	50.90	Enterobacteriaceae	17216	4450384
Sample_LCC15.mb.27cbin.1	55.10	Prevotella	20191	2356845
Sample_LCC15.mb.14cbin.1	45.10	Lachnospiraceae	5629	2078075
Sample_LCC15.Cluster403cbin.1	56.80	Bifidobacteriaceae	15503	2029177
Sample_LCC15.mb.9	51.00	Enterobacteriaceae	46438	4359194
Sample_LCC16.mb.28	63.10	Clostridiales	8834	1562527
Sample_LCC16.Cluster1073cbin.1	47.70	Clostridiales	97705	3025014
Sample_LCC16.mb.15	43.50	Selenomonadales	7882	2122874
Sample_LCC16.mb.34cbin.1	52.10	Clostridiales	36765	1716264
Sample_LCC16.mb.29cbin.1	53.60	Clostridiales	437506	1935031
Sample_LCC16.Cluster2701cbin.1	40.70	Clostridiales	135169	2106244
Sample_LCC16.mb.5cbin.1	54.50	Bacteroidetes	109598	2133792
Sample_LCC16.mb.1cbin.1	56.20	Clostridiales	15502	2578466
Sample_LCC16.Cluster75cbin.1	57.00	Bifidobacteriaceae	109280	2161851
Sample_LCC16.mb.25	35.90	Clostridiales	142743	2446902
Sample_LCC16.mb.32	43.50	Lachnospiraceae	112269	2827473
Sample_LCC16.Cluster2570	41.20	Lachnospiraceae	69073	2865196
Sample_LCC16.Cluster2870cbin.1	40.90	Clostridiales	58941	3767556
Sample_LCC16.mb.30	46.70	Bacteroidales	39437	4007980
Sample_LCC17.Cluster16446	26.60	Bacteria	58879	1048374
Sample_LCC17.Cluster16367	26.50	Bacteria	175020	1208669
Sample_LCC17.Cluster16722	25.10	Bacteria	198093	1236943
Sample_LCC17.Cluster15858	28.60	Bacteria	11747	1272042
Sample_LCC17.Cluster1862cbin.1	51.20	Bacteria	6019	1627514
Sample_LCC17.Cluster3097cbin.1	53.60	Clostridiales	46692	1791276
Sample_LCC17.Cluster15226cbin.1	36.50	Clostridiales	232298	1881253
Sample_LCC17.Cluster4190cbin.1	53.00	Clostridia	81799	2019079
Sample_LCC17.Cluster2315cbin.1	58.20	Clostridiales	52586	2849393
Sample_LCC17.Cluster180	62.10	Clostridiales	15554	1432343
Sample_LCC17.Cluster8838	45.70	Bacteria	62019	1764064
Sample_LCC17.Cluster12879	40.60	Clostridiales	124750	1595916
Sample_LCC17.Cluster3989cbin.1	49.00	Clostridiales	114012	2477461
Sample_LCC17.Cluster15123	33.60	Clostridiales	74472	1874865
Sample_LCC17.Cluster15926	31.20	Euryarchaeota	39467	1697872
Sample_LCC17.Cluster8364	45.70	Clostridiales	48930	1899680
Sample_LCC17.mb.12cbin.1	48.70	Clostridiales	76958	2428149
Sample_LCC17.Cluster317	60.10	Bacteroidetes	30243	2688269
Sample_LCC17.Cluster4955cbin.1	54.60	Bacteroidetes	103045	2092421

Sample_LCC17.Cluster4788cbin.1	38.60	Clostridiales	49273	2157108
Sample_LCC17.mb.131cbin.1	56.10	Clostridiales	52438	2552052
Sample_LCC17.mb.36cbin.1	41.30	Clostridiales	223083	2530806
Sample_LCC17.Cluster44cbin.1	63.90	Deltaproteobacteria	15002	2340089
Sample_LCC17.mb.119	57.70	Bacteria	15778	2306101
Sample_LCC17.Cluster9633cbin.1	46.40	Bacteroidetes	104640	2344611
Sample_LCC17.mb.74	26.40	Bacteria	104037	1275189
Sample_LCC17.Cluster13321cbin.1	41.30	Lachnospiraceae	64960	2910914
Sample_LCC17.Cluster750cbin.1	59.60	Bacteroidetes	88011	2668258
Sample_LCC17.Cluster16120cbin.1	31.30	Firmicutes	36380	2339585
Sample_LCC17.mb.52cbin.1	57.80	Clostridiales	6534	1747967
Sample_LCC17.mb.25cbin.1	60.30	Actinobacteria	10289	1918454
Sample_LCC17.mb.53	62.80	Clostridiales	13188	1746050
Sample_LCC17.Cluster13753cbin.1	38.40	Bacteroidales	65491	3358006
Sample_LCC17.mb.59cbin.1	37.70	Clostridiales	118891	2257500
Sample_LCC17.mb.22	51.10	Bacteroidetes	10137	2185682
Sample_LCC17.mb.120	57.70	Clostridiales	81530	2455685
Sample_LCC17.Cluster11558cbin.1	43.30	Bacteria	16316	3881562
Sample_LCC17.mb.78	58.20	Clostridiales	25619	2109503
Sample_LCC17.mb.26	50.30	Bacteroidales	106953	2299214
Sample_LCC17.mb.68	44.80	Clostridiales	62997	2591845
Sample_LCC17.Cluster14361cbin.1	45.30	Bacteroidales	8151	3647373
Sample_LCC17.mb.86	46.20	Clostridiales	9993	1937078
Sample_LCC17.mb.67cbin.1	56.40	Clostridiales	15693	2470625
Sample_LCC17.mb.70	41.20	Clostridiales	212707	2463723
Sample_LCC17.mb.8cbin.1	46.30	Lachnospiraceae	64768	2895875
Sample_LCC17.mb.88	45.10	Clostridiales	78905	1994029
Sample_LCC17.mb.95cbin.1	59.90	Bifidobacteriaceae	8997	1893531
Sample_LCC17.mb.92cbin.1	51.40	Clostridiales	122182	2134157
Sample_LCC17.mb.94cbin.1	43.20	Clostridiales	17859	2616266
Sample_LCC17.mb.112cbin.1	46.80	Bacteroidales	45366	4139891
Sample_LCC17.mb.82	41.90	Lachnospiraceae	24386	2241933
Sample_LCC17.mb.73	44.20	Bacteroidales	150978	3572399
Sample_LCC19.mb.44	47.00	Bacteria	53591	1679839
Sample_LCC19.mb.64	26.10	Bacteria	43110	1179770
Sample_LCC19.Cluster45cbin.1	61.30	Clostridiales	136849	2049424
Sample_LCC19.Cluster2436cbin.1	47.70	Clostridiales	83733	3006261
Sample_LCC19.mb.43	49.00	Clostridiales	30570	2498564
Sample_LCC19.Cluster744cbin.1	54.70	Bacteroidetes	155375	1936191
Sample_LCC19.Cluster1101	53.70	Clostridiales	569342	1805402
Sample_LCC19.mb.35	56.60	Clostridiales	13588	2259941
Sample_LCC19.mb.48cbin.1	60.10	Clostridiales	37733	1914861
Sample_LCC19.Cluster24cbin.1	60.20	Bifidobacteriaceae	6619	1849755
Sample_LCC19.mb.19cbin.1	41.00	Clostridiales	185157	2113602
Sample_LCC19.Cluster3872cbin.1	42.90	Clostridiales	82751	2386317
Sample_LCC19.mb.17cbin.1	56.80	Bifidobacteriaceae	154094	2154471
Sample_LCC19.Cluster5834cbin.1	35.90	Clostridiales	89126	2617018
Sample_LCC19.mb.90cbin.1	63.70	Actinobacteria	32329	2592483
Sample_LCC19.mb.45	26.60	Clostridiales	20483	2775106
Sample_LCC19.mb.85cbin.1	43.90	Lachnospiraceae	98577	2479340
Sample_LCC19.Cluster1741	46.70	Bacteroidales	127847	2806294
Sample_LCC19.mb.49	42.30	Bacteroidales	63606	3260087
Sample_LCC19.Cluster3286cbin.1	41.70	Lachnospiraceae	14866	3501988
Sample_LCC19.mb.79	45.10	Clostridiales	32467	2705470
Sample_LCC19.mb.53cbin.1	38.10	Lactobacillales	36245	2873056
Sample_LCC19.Cluster1812cbin.1	44.90	Bacteroidales	10445	3820169
Sample_LCC19.mb.31cbin.1	50.70	Enterobacteriaceae	151464	4702608
Sample_LCC19.Cluster32	56.20	Enterobacteriaceae	146518	5556779

Sample_LCC20.Cluster10333	26.40	Bacteria	127272	1080291
Sample_LCC20.mb.3	26.00	Bacteria	121358	1361878
Sample_LCC20.Cluster8196cbin.1	37.50	Clostridiales	141749	2062084
Sample_LCC20.Cluster247cbin.1	60.90	Clostridiales	87342	1922826
Sample_LCC20.Cluster427cbin.1	58.30	Clostridiales	39455	2379266
Sample_LCC20.mb.57	53.00	Clostridia	12188	1959606
Sample_LCC20.mb.21	41.80	Clostridiales	160795	2620320
Sample_LCC20.mb.43cbin.1	61.00	Clostridiales	15626	1638402
Sample_LCC20.Cluster3708	49.90	Clostridiales	146561	2307960
Sample_LCC20.mb.60	58.00	Clostridiales	45421	3008712
Sample_LCC20.mb.44	62.00	Clostridiales	24618	1954018
Sample_LCC20.Cluster2912cbin.1	49.00	Bacteroidales	180829	3007968
Sample_LCC20.Cluster3319cbin.1	44.00	Selenomonadales	13906	2207150
Sample_LCC20.Cluster689cbin.1	50.90	Prevotella	69771	2385043
Sample_LCC20.Cluster5995	40.60	Clostridiales	187098	1966520
Sample_LCC20.mb.63	51.10	Clostridiales	8841	1533973
Sample_LCC20.mb.51	57.30	Clostridiales	69682	1878435
Sample_LCC20.mb.22	54.80	Bacteroidetes	123145	2207280
Sample_LCC20.mb.29cbin.1	44.60	Clostridiales	152822	1908374
Sample_LCC20.mb.75	54.80	Clostridiales	17690	2514980
Sample_LCC20.mb.32cbin.1	31.20	Euryarchaeota	16951	1688181
Sample_LCC20.mb.18	59.40	Bacteroidetes	76372	2745648
Sample_LCC20.mb.36cbin.1	37.70	Clostridiales	37853	2395095
Sample_LCC20.mb.56	56.40	Clostridiales	15518	2436870
Sample_LCC20.mb.92cbin.1	55.70	Clostridiales	122130	2724602
Sample_LCC20.Cluster5529	43.40	Bacteroidales	265362	3076903
Sample_LCC20.Cluster5535	43.00	Bacteria	19180	3771591
Sample_LCC20.mb.34cbin.1	42.30	Bacteroidales	36561	4448720
Sample_LCC20.mb.62	46.50	Bacteroidales	35039	4241460
Sample_LCC20.mb.94cbin.1	45.60	Bacteroidales	20645	4552612
Sample_LCC20.Cluster3175	50.90	Enterobacteriaceae	142896	4413562
Sample_LCC20.Cluster625	57.70	Enterobacteriaceae	196991	4923684
Sample_LCC21.Cluster13810cbin.1	26.70	Bacteria	10369	1151845
Sample_LCC21.Cluster163	59.60	Actinobacteria	14043	1539610
Sample_LCC21.mb.33cbin.1	53.80	Clostridia	35991	1839666
Sample_LCC21.mb.50cbin.1	33.00	Bacteria	17057	1930349
Sample_LCC21.mb.31	59.60	Clostridiales	6639	1757709
Sample_LCC21.Cluster4626	50.50	Bacteroidales	69920	2259911
Sample_LCC21.mb.43	60.70	Clostridiales	67669	1858345
Sample_LCC21.Cluster10978cbin.1	41.10	Lachnospiraceae	111009	2835344
Sample_LCC21.Cluster4492cbin.1	48.80	Firmicutes	10169	1897352
Sample_LCC21.Cluster5920	44.00	Selenomonadales	35151	2084991
Sample_LCC21.mb.104	54.00	Clostridiales	74075	1708356
Sample_LCC21.Cluster11322	40.90	Clostridiales	189245	2034202
Sample_LCC21.mb.92cbin.1	49.50	Clostridiales	96854	2029867
Sample_LCC21.Cluster5138cbin.1	51.40	Clostridiales	123414	2187660
Sample_LCC21.mb.79cbin.1	57.30	Clostridiales	88002	1788525
Sample_LCC21.mb.57cbin.1	60.30	Actinobacteria	13633	2098748
Sample_LCC21.mb.107cbin.1	43.50	Clostridiales	180933	2886613
Sample_LCC21.mb.46	44.50	Clostridiales	119676	2246451
Sample_LCC21.mb.70	59.90	Clostridiales	11518	1968969
Sample_LCC21.mb.75	45.10	Clostridiales	55660	2518407
Sample_LCC21.Cluster8966cbin.1	42.70	Clostridiales	116436	2689955
Sample_LCC21.Cluster1186cbin.1	58.00	Bacteroidetes	110916	3261971
Sample_LCC21.Cluster13247cbin.1	31.20	Firmicutes	60672	2427478
Sample_LCC21.Cluster9689cbin.1	40.10	Lachnospiraceae	13451	3914383
Sample_LCC21.mb.99cbin.1	56.00	Clostridiales	5739	2323878
Sample_LCC21.Cluster1714cbin.1	45.60	Bacteroidales	8082	3657932

Sample_LCC21.mb.71cbin.1	50.80	Enterobacteriaceae	44369	4542110
Sample_LCC22.Cluster11576	26.70	Bacteria	118031	1185786
Sample_LCC22.Cluster2424	50.80	Bacteria	106097	1843502
Sample_LCC22.mb.22cbin.1	49.50	Clostridiales	18045	1867852
Sample_LCC22.mb.27	32.80	Bacteria	87603	1948482
Sample_LCC22.mb.18cbin.1	58.10	Clostridiales	50036	3097295
Sample_LCC22.Cluster385	58.30	Clostridiales	65266	2353634
Sample_LCC22.mb.11cbin.1	58.20	Clostridiales	53065	2171871
Sample_LCC22.mb.40cbin.1	57.20	Clostridiales	24196	2273061
Sample_LCC22.Cluster128	59.60	Actinobacteria	49431	2072281
Sample_LCC22.mb.35	61.40	Clostridiales	54210	2073283
Sample_LCC22.Cluster6998	41.50	Clostridiales	479723	1750722
Sample_LCC22.Cluster3715	47.30	Selenomonadales	84326	2132938
Sample_LCC22.mb.47	50.10	Clostridiales	15348	1971609
Sample_LCC22.Cluster9158	39.00	Clostridiales	102045	1862840
Sample_LCC22.mb.16	58.90	Clostridiales	32692	2145395
Sample_LCC22.mb.46cbin.1	53.30	Clostridiales	32704	2572860
Sample_LCC22.mb.36	46.60	Lachnospiraceae	58846	2711758
Sample_LCC22.Cluster278	55.40	Bacteria	317815	2726366
Sample_LCC22.mb.2cbin.1	37.60	Clostridiales	5830	2216621
Sample_LCC22.Cluster8027cbin.1	42.90	Clostridiales	94339	2672527
Sample_LCC22.mb.19	41.80	Lachnospiraceae	96833	2531047
Sample_LCC22.mb.37	36.00	Clostridiales	194952	2399557
Sample_LCC22.mb.76	60.90	Clostridiales	105022	1902040
Sample_LCC22.Cluster6899cbin.1	42.10	Bacteroidales	61728	3075818
Sample_LCC22.mb.34cbin.1	45.40	Prevotella	33602	3275529
Sample_LCC22.mb.62cbin.1	55.10	Bacteroidetes	32940	1922060
Sample_LCC22.mb.58	61.60	Proteobacteria	42060	2493468
Sample_LCC22.mb.81cbin.1	61.30	Clostridiales	11293	1900389
Sample_LCC22.mb.31cbin.1	46.40	Bacteroidales	62589	3070835
Sample_LCC22.mb.29cbin.1	40.30	Clostridiales	20546	4547770
Sample_LCC22.Cluster8294cbin.1	41.90	Bacteroidales	33602	3990865
Sample_LCC22.mb.87	41.20	Clostridiales	215984	2690050
Sample_LCC22.mb.38	45.10	Bacteroidales	79685	3772930
Sample_LCC22.mb.50	45.40	Bacteroidales	52515	4067759
Sample_LCC22.mb.5cbin.1	43.00	Bacteria	22390	4058288
Sample_LCC22.mb.92cbin.1	48.90	Bacteroidales	27608	3286438
Sample_LCC22.mb.90cbin.1	50.60	Enterobacteriaceae	172256	4944108
Sample_LCC23.mb.89cbin.1	41.20	Bacteria	51479	3052442
Sample_LCC23.mb.18cbin.1	58.90	Clostridiales	11895	2544324
Sample_LCC23.Cluster156	59.80	Actinobacteria	134039	2094335
Sample_LCC23.mb.47	60.00	Clostridiales	85839	1911335
Sample_LCC23.Cluster3cbin.1	63.20	Actinobacteria	76747	2660295
Sample_LCC23.Cluster976cbin.1	51.10	Bacteroidales	57891	2573602
Sample_LCC23.Cluster311cbin.1	55.60	Bacteria	40136	2708885
Sample_LCC23.Cluster8057	40.50	Clostridiales	147176	2069494
Sample_LCC23.mb.17	47.00	Prevotella	27616	2106376
Sample_LCC23.Cluster9143	38.20	Clostridiales	190128	2024067
Sample_LCC23.mb.93cbin.1	58.60	Clostridiales	68618	1970759
Sample_LCC23.mb.26	51.50	Clostridiales	22069	2089028
Sample_LCC23.mb.55	54.60	Bacteroidetes	59879	2121625
Sample_LCC23.Cluster44cbin.1	63.10	Proteobacteria	37821	2599865
Sample_LCC23.mb.19	28.20	Bacteria	8439	2162299
Sample_LCC23.mb.83cbin.1	47.30	Selenomonadales	29044	2090088
Sample_LCC23.Cluster7957cbin.1	44.70	Clostridiales	86965	3030546
Sample_LCC23.mb.54cbin.1	49.90	Bacteroidales	78812	2259886
Sample_LCC23.mb.57	43.70	Lachnospiraceae	97976	2727078
Sample_LCC23.mb.14	43.30	Lachnospiraceae	32294	2788320

Sample_LCC23.mb.72cbin.1	55.20	Bacteroidales	70848	2473125
Sample_LCC23.mb.98	44.30	Clostridiales	155471	2705418
Sample_LCC23.mb.87cbin.1	57.80	Clostridiales	29156	2502504
Sample_LCC23.mb.6	41.60	Lachnospiraceae	71322	3045259
Sample_LCC23.mb.23cbin.1	45.40	Bacteroidales	78399	3883496
Sample_LCC23.Cluster310cbin.1	51.10	Enterobacteriaceae	18982	4248554
Sample_LCC24.Cluster5757	34.30	Clostridiales	6695	2031526
Sample_LCC24.Cluster3364cbin.1	42.10	Clostridiales	14729	2608979
Sample_LCC24.Cluster82cbin.1	59.20	Clostridiales	19635	1906918
Sample_LCC24.Cluster946	51.50	Clostridiales	23973	1953178
Sample_LCC24.mb.18	60.00	Actinobacteria	47004	2076100
Sample_LCC24.mb.53cbin.1	48.80	Clostridiales	15215	2630259
Sample_LCC24.Cluster172cbin.1	53.60	Lactobacillales	10325	1544350
Sample_LCC24.Cluster78cbin.1	56.50	Bifidobacteriaceae	27436	1863987
Sample_LCC24.mb.31	56.50	Clostridiales	7434	2276301
Sample_LCC24.mb.15	60.20	Bacteroidetes	75610	2453016
Sample_LCC24.mb.21	34.80	Lactobacillus	18104	1798401
Sample_LCC24.Cluster6435cbin.1	32.60	Lactobacillales	133279	1871564
Sample_LCC24.mb.23cbin.1	44.30	Clostridiales	259354	2188687
Sample_LCC24.Cluster44	62.40	Proteobacteria	58827	2395041
Sample_LCC24.mb.4	63.20	Bifidobacteriaceae	17867	2073397
Sample_LCC24.mb.50cbin.1	54.20	Selenomonadales	81225	2070432
Sample_LCC24.Cluster2026cbin.1	46.00	Bacteroidales	60409	2969450
Sample_LCC24.mb.26	59.10	Bacteroidetes	120531	2731636
Sample_LCC24.Cluster4973cbin.1	41.20	Clostridiales	19016	3448049
Sample_LCC24.mb.42	58.70	Bifidobacteriaceae	17002	2295669
Sample_LCC24.mb.40cbin.1	41.30	Lachnospiraceae	111412	2989175
Sample_LCC24.mb.60	31.30	Firmicutes	10523	2124901
Sample_LCC24.Cluster1831	45.60	Bacteroidales	35351	3663298
Sample_LCC25.mb.22cbin.1	48.30	Clostridiales	24901	2964171
Sample_LCC25.mb.1	49.40	Clostridiales	8070	2309806
Sample_LCC25.Cluster6741cbin.1	27.70	Clostridiales	5091	1574839
Sample_LCC25.mb.2	59.90	Actinobacteria	28476	2098744
Sample_LCC25.mb.31cbin.1	56.70	Clostridiales	6080	2106863
Sample_LCC25.mb.35	60.70	Clostridiales	64611	2191771
Sample_LCC25.Cluster2894	43.40	Selenomonadales	250365	2379498
Sample_LCC25.Cluster5887	36.00	Clostridiales	16077	2068474
Sample_LCC25.mb.12	40.90	Clostridiales	164767	2289660
Sample_LCC25.mb.3cbin.1	59.80	Bacteroidetes	12225	2720599
Sample_LCC25.mb.73	54.80	Bacteroidetes	136011	2020029
Sample_LCC25.Cluster1411cbin.1	45.50	Prevotella	34339	3120550
Sample_LCC25.mb.37cbin.1	41.00	Lachnospiraceae	72746	2914305
Sample_LCC25.Cluster4290	42.80	Lachnospiraceae	98857	2996292
Sample_LCC25.Cluster6913	29.10	Bacteria	59993	2596339
Sample_LCC25.mb.5cbin.1	60.10	Bifidobacteriaceae	236859	2425739
Sample_LCC25.Cluster2423cbin.1	44.50	Bacteroidales	97389	3094687
Sample_LCC25.Cluster2907cbin.1	41.20	Lachnospiraceae	58350	3532062
Sample_LCC25.mb.59cbin.1	31.10	Firmicutes	56810	2530287
Sample_LCC25.Cluster976cbin.1	42.10	Bacteroidales	41036	3909322
Sample_LCC25.mb.21cbin.1	50.80	Enterobacteriaceae	70697	4625324
Sample_LPA01.Cluster5570cbin.1	41.30	Clostridiales	13000	2969978
Sample_LPA01.Cluster9393	36.40	Clostridiales	157196	1897292
Sample_LPA01.Cluster3463cbin.1	45.90	Clostridiales	77571	2193194
Sample_LPA01.mb.8	60.10	Bacteria	40000	2593780
Sample_LPA01.mb.54	33.30	Clostridiales	72776	1986479
Sample_LPA01.Cluster1315cbin.1	48.50	Firmicutes	32405	1938690
Sample_LPA01.Cluster658cbin.1	54.70	Bacteroidetes	104484	2029274
Sample_LPA01.Cluster90cbin.1	60.00	Clostridiales	139335	2984495

Sample_LPA01.mb.57cbin.1	59.40	Clostridiales	9209	1832901
Sample_LPA01.Cluster9888	33.70	Clostridiales	60132	1777250
Sample_LPA01.mb.13	60.40	Clostridiales	23002	2282811
Sample_LPA01.mb.27cbin.1	56.40	Clostridiales	14740	2428931
Sample_LPA01.mb.87cbin.1	58.40	Clostridiales	39234	2809633
Sample_LPA01.mb.61cbin.1	49.40	Clostridiales	18803	2376057
Sample_LPA01.mb.21cbin.1	57.70	Proteobacteria	18169	2196229
Sample_LPA01.mb.78cbin.1	48.40	Clostridiales	42391	2729227
Sample_LPA01.Cluster9248cbin.1	35.90	Clostridiales	187298	2539375
Sample_LPA01.Cluster10199cbin.1	31.30	Firmicutes	51736	2359315
Sample_LPA01.mb.74	60.10	Actinobacteria	35205	2028582
Sample_LPA01.mb.34	57.90	Bacteria	28065	2950458
Sample_LPA01.mb.67	47.00	Lachnospiraceae	81242	2987414
Sample_LPA01.mb.15cbin.1	43.20	Lachnospiraceae	123820	2920672
Sample_LPA01.mb.16	41.50	Lachnospiraceae	73199	2717299
Sample_LPA01.Cluster1542cbin.1	48.80	Bacteroidales	40992	3306394
Sample_LPA01.mb.23	43.30	Bacteroidales	183514	2936682
Sample_LPA01.mb.20cbin.1	48.50	Proteobacteria	37817	2639660
Sample_LPA01.mb.81cbin.1	42.90	Clostridiales	112447	2655099
Sample_LPA01.Cluster3577cbin.1	45.10	Bacteroidales	88478	4472020
Sample_LPA01.mb.101	45.50	Bacteroidales	38822	3746891
Sample_LPA01.mb.90	58.80	Bacteroidetes	137942	2937546
Sample_LPA02.Cluster15863	24.80	Bacteria	96892	1347461
Sample_LPA02.Cluster6186	43.80	Clostridiales	7660	2368573
Sample_LPA02.Cluster7901	45.30	Clostridiales	129966	1833521
Sample_LPA02.Cluster4739cbin.1	48.80	Clostridiales	14183	2613055
Sample_LPA02.Cluster184cbin.1	58.70	Clostridiales	40366	2184193
Sample_LPA02.Cluster3382	53.50	Clostridiales	249481	1941610
Sample_LPA02.Cluster5710	43.80	Selenomonadales	129540	2274173
Sample_LPA02.mb.63	26.70	Bacteria	257512	1150263
Sample_LPA02.Cluster2508cbin.1	56.40	Bifidobacteriaceae	154544	2157522
Sample_LPA02.mb.101	59.00	Bacteroidetes	10969	2238003
Sample_LPA02.mb.103cbin.1	49.70	Bacteroidales	58010	2652478
Sample_LPA02.mb.116	46.60	Clostridiales	23095	2129579
Sample_LPA02.mb.104cbin.1	51.00	Clostridiales	132835	2333458
Sample_LPA02.mb.36	57.10	Clostridiales	63172	1915877
Sample_LPA02.mb.129	38.90	Clostridiales	28032	2003373
Sample_LPA02.mb.57	62.00	Clostridiales	28707	1968940
Sample_LPA02.mb.97	49.90	Clostridia	75375	1555585
Sample_LPA02.mb.17	41.90	Clostridiales	149679	2376669
Sample_LPA02.mb.16	44.00	Clostridiales	188721	2768835
Sample_LPA02.mb.45cbin.1	62.60	Bifidobacteriaceae	100689	2116845
Sample_LPA02.mb.39	46.70	Lachnospiraceae	50641	3274027
Sample_LPA02.mb.44	54.90	Bacteroidetes	30565	2359717
Sample_LPA02.mb.88	60.10	Actinobacteria	13846	1992417
Sample_LPA02.mb.52	46.20	Prevotella	42920	2896029
Sample_LPA02.mb.28	45.90	Bacteroidales	74642	2764086
Sample_LPA02.mb.81	44.80	Clostridiales	24087	2441709
Sample_LPA02.mb.83	44.60	Clostridiales	130086	2280757
Sample_LPA02.mb.59	41.80	Lachnospiraceae	60478	2416341
Sample_LPA02.mb.91	40.70	Clostridiales	74399	2090852
Sample_LPA02.mb.80cbin.1	50.20	Lachnospiraceae	10895	2615955
Sample_LPA02.mb.92	42.00	Clostridiales	34055	2848046
Sample_LPA02.Cluster2225cbin.1	50.70	Enterobacteriaceae	26032	4644976
Sample_LPA02.mb.50cbin.1	44.90	Bacteroidales	78110	4871522
Sample_LPA03.mb.38	25.20	Bacteria	154057	1294474
Sample_LPA03.mb.44	26.00	Bacteria	76863	1249024
Sample_LPA03.mb.48	26.40	Bacteria	145734	1229322

Sample_LPA03.mb.24	53.60	Clostridia	36084	1889418
Sample_LPA03.mb.52	25.80	Bacteria	52101	1374625
Sample_LPA03.mb.36	52.70	Clostridia	112149	2112493
Sample_LPA03.mb.21	49.40	Clostridiales	74708	2003078
Sample_LPA03.mb.15	58.50	Clostridiales	39651	2487693
Sample_LPA03.mb.7	24.30	Bacteria	48086	1036057
Sample_LPA03.Cluster989cbin.1	53.70	Clostridiales	11394	2058283
Sample_LPA03.Cluster907cbin.1	57.10	Clostridia	124555	4064562
Sample_LPA03.mb.55cbin.1	54.90	Clostridia	213734	2686883
Sample_LPA03.mb.66	46.70	Clostridiales	12503	1851554
Sample_LPA03.mb.42cbin.1	62.10	Clostridiales	84679	1862625
Sample_LPA03.Cluster245cbin.1	58.60	Clostridiales	42274	2341336
Sample_LPA03.mb.30	42.90	Clostridiales	12969	3000735
Sample_LPA03.mb.23cbin.1	40.10	Clostridiales	6440	3442770
Sample_LPA03.mb.67	57.30	Clostridiales	74193	1911328
Sample_LPA03.Cluster2348	51.80	Clostridiales	134557	2134921
Sample_LPA03.Cluster1473	55.60	Bacteria	225702	2693238
Sample_LPA03.mb.10cbin.1	38.60	Clostridiales	120194	2212691
Sample_LPA03.mb.57cbin.1	41.00	Clostridiales	46457	1961275
Sample_LPA03.mb.53	58.70	Clostridiales	36380	2969992
Sample_LPA03.mb.40	58.50	Bifidobacteriaceae	165613	2462598
Sample_LPA03.mb.17cbin.1	43.20	Bacteria	13862	3977834
Sample_LPA03.Cluster5054cbin.1	39.00	Lachnospiraceae	9057	3768190
Sample_LPA03.mb.50cbin.1	57.60	Enterobacteriaceae	52788	5241037
Sample_LPA04.Cluster13204	28.60	Bacteria	14481	1352163
Sample_LPA04.Cluster7577	47.20	Clostridiales	165011	1560380
Sample_LPA04.mb.3	52.80	Clostridia	68479	2152907
Sample_LPA04.Cluster724	55.10	Clostridia	201332	2660834
Sample_LPA04.mb.54cbin.1	26.10	Bacteria	51241	1197535
Sample_LPA04.Cluster12181cbin.1	36.50	Clostridiales	7972	2107251
Sample_LPA04.mb.23	69.20	Bacteria	30586	2206631
Sample_LPA04.mb.52cbin.1	62.10	Clostridia	248839	1872101
Sample_LPA04.Cluster612cbin.1	60.60	Clostridiales	41835	1806812
Sample_LPA04.mb.58	51.00	Clostridiales	14409	2071798
Sample_LPA04.mb.16	58.40	Bacteria	8273	2112271
Sample_LPA04.mb.19	62.40	Clostridiales	39894	2559405
Sample_LPA04.mb.76	53.50	Clostridia	64897	1854336
Sample_LPA04.mb.79cbin.1	43.00	Clostridiales	112144	1259910
Sample_LPA04.mb.51	56.70	Clostridiales	38022	2496059
Sample_LPA04.mb.75	47.10	Clostridiales	16449	1919620
Sample_LPA04.Cluster5076	43.60	Selenomonadales	47138	2103651
Sample_LPA04.mb.63	62.30	Clostridiales	6387	1564697
Sample_LPA04.Cluster1103cbin.1	58.70	Bacteria	99354	2650774
Sample_LPA04.mb.59	59.10	Clostridiales	21974	2175211
Sample_LPA04.mb.37	48.50	Proteobacteria	28338	1635647
Sample_LPA04.Cluster3136	55.50	Bacteria	127223	2795287
Sample_LPA04.mb.72	59.80	Clostridiales	16879	2285023
Sample_LPA04.mb.57	60.20	Actinobacteria	40251	2111972
Sample_LPA04.Cluster12331	37.40	Clostridiales	67519	2426442
Sample_LPA04.mb.30	44.10	Clostridiales	92327	2738535
Sample_LPA04.mb.80	38.00	Clostridiales	10014	3421146
Sample_LPA04.mb.42cbin.1	63.80	Deltaproteobacteria	19844	2560682
Sample_LPA04.mb.87	53.50	Clostridiales	31512	1928492
Sample_LPA04.mb.70	36.80	Clostridiales	10412	1741051
Sample_LPA04.Cluster1856	58.30	Bacteroidetes	141099	3096996
Sample_LPA04.Cluster9609	43.40	Bacteroidales	289886	3040453
Sample_LPA04.mb.92cbin.1	31.10	Euryarchaeota	17368	1743199
Sample_LPA04.Cluster8097cbin.1	42.00	Lachnospiraceae	50020	3757709

Sample_LPA04.mb.95cbin.1	47.20	Bacteroidales	33481	3617873
Sample_LPA04.mb.5cbin.1	45.70	Bacteroidales	15230	3828141
Sample_LPA04.Cluster7322cbin.1	45.20	Bacteroidales	115884	4612647
Sample_LPA04.mb.2	50.60	Enterobacteriaceae	183654	4544448
Sample_LPA05.mb.13	26.00	Bacteria	35872	1263127
Sample_LPA05.mb.17	46.50	Bacteria	41777	1768719
Sample_LPA05.Cluster12739cbin.1	37.40	Clostridiales	7707	2193651
Sample_LPA05.Cluster2994cbin.1	48.70	Clostridiales	10998	2662497
Sample_LPA05.Cluster147cbin.1	58.90	Clostridiales	6013	2536983
Sample_LPA05.Cluster1752cbin.1	61.40	Clostridiales	12820	2143222
Sample_LPA05.mb.3	58.00	Clostridiales	42700	3150750
Sample_LPA05.Cluster11436cbin.1	43.50	Selenomonadales	12506	2157678
Sample_LPA05.mb.44cbin.1	56.80	Clostridiales	16653	2544614
Sample_LPA05.mb.16	48.80	Bacteria	10248	2332959
Sample_LPA05.mb.38cbin.1	53.00	Clostridiales	9827	1953010
Sample_LPA05.mb.22	60.10	Actinobacteria	19095	2064272
Sample_LPA05.Cluster10884	40.90	Clostridiales	120053	2150544
Sample_LPA05.mb.46	40.90	Lachnospiraceae	122978	2636133
Sample_LPA05.Cluster625cbin.1	56.40	Bifidobacteriaceae	313284	2199216
Sample_LPA05.Cluster223cbin.1	55.90	Proteobacteria	4691	1871583
Sample_LPA05.Cluster8593	44.00	Clostridiales	213452	2884193
Sample_LPA05.mb.62cbin.1	61.50	Clostridiales	30715	2545910
Sample_LPA05.mb.50cbin.1	61.60	Clostridiales	10262	3191661
Sample_LPA05.mb.55	56.50	Clostridiales	9330	2360873
Sample_LPA05.mb.2	41.60	Lachnospiraceae	71660	2682609
Sample_LPA05.mb.45cbin.1	50.60	Lachnospiraceae	19335	2430045
Sample_LPA05.mb.59cbin.1	60.00	Bifidobacteriaceae	47893	2316093
Sample_LPA05.mb.25	43.30	Bacteroidales	118359	2850349
Sample_LPA05.mb.14cbin.1	48.80	Bacteroidales	34270	3451840
Sample_LPA05.Cluster3925cbin.1	45.30	Bacteroidales	29398	3933288
Sample_LPA05.mb.84	58.50	Bacteroidetes	230911	3119840
Sample_LPA08.mb.35cbin.1	42.10	Bacteria	87281	4611181
Sample_LPA08.mb.19	33.60	Clostridiales	23524	2132107
Sample_LPA08.Cluster126cbin.1	55.10	Clostridiales	27518	2386730
Sample_LPA08.Cluster13cbin.1	60.90	Clostridiales	16530	1735306
Sample_LPA08.Cluster103	57.90	Clostridiales	69946	2127697
Sample_LPA08.mb.41	61.40	Clostridiales	31914	1915701
Sample_LPA08.mb.34cbin.1	61.60	Clostridiales	144098	2082147
Sample_LPA08.mb.43	60.80	Clostridiales	79066	2389130
Sample_LPA08.mb.65cbin.1	38.00	Clostridiales	29257	3589409
Sample_LPA08.Cluster1168cbin.1	54.20	Bacteroidetes	161273	2106750
Sample_LPA08.Cluster449	53.70	Clostridiales	260894	1860583
Sample_LPA08.Cluster3810cbin.1	43.40	Selenomonadales	148610	2505766
Sample_LPA08.mb.30cbin.1	40.60	Clostridiales	237346	2110332
Sample_LPA08.Cluster818cbin.1	54.20	Selenomonadales	86732	2160488
Sample_LPA08.mb.23cbin.1	56.50	Bifidobacteriaceae	35984	2019408
Sample_LPA08.Cluster111cbin.1	59.00	Bacteroidetes	133533	2906612
Sample_LPA08.mb.63	46.40	Lachnospiraceae	34416	2923094
Sample_LPA08.mb.67	60.70	Clostridiales	26397	2219831
Sample_LPA08.mb.17	42.80	Clostridiales	95012	2649601
Sample_LPA08.mb.37	29.00	Bacteria	48443	2678454
Sample_LPA08.Cluster2626cbin.1	43.20	Bacteroidales	5902	2440315
Sample_LPA08.mb.47	32.60	Lactobacillales	19866	1948170
Sample_LPA08.Cluster24cbin.1	60.10	Deltaproteobacteria	18482	3822488
Sample_LPA08.mb.49	50.20	Bacteroidales	97930	2472130
Sample_LPA08.Cluster5160cbin.1	40.80	Lachnospiraceae	67726	3170039
Sample_LPA08.Cluster2236cbin.1	41.80	Lachnospiraceae	63558	2516711
Sample_LPA08.Cluster548cbin.1	48.20	Proteobacteria	20450	2730176

Sample_LPA08.Cluster1301	45.20	Bacteroidales	136547	4072813
Sample_LPB01.Cluster213cbin.1	59.80	Clostridiales	150223	2018897
Sample_LPB01.mb.30cbin.1	59.40	Clostridiales	6545	1729568
Sample_LPB01.mb.21cbin.1	33.60	Clostridiales	150184	3703948
Sample_LPB01.mb.41	61.50	Clostridiales	16364	2411693
Sample_LPB01.Cluster1684cbin.1	48.40	Firmicutes	20994	1677897
Sample_LPB01.mb.63cbin.1	48.70	Clostridiales	10938	2463713
Sample_LPB01.mb.84	36.30	Clostridiales	472273	1886670
Sample_LPB01.Cluster3819cbin.1	46.70	Lachnospiraceae	76514	3413028
Sample_LPB01.mb.27	54.60	Bacteroidetes	114740	2004787
Sample_LPB01.Cluster1675	51.00	Clostridiales	150109	2293771
Sample_LPB01.Cluster5666	43.40	Lachnospiraceae	153570	2375497
Sample_LPB01.mb.22	41.20	Clostridiales	150727	2495592
Sample_LPB01.mb.59cbin.1	33.70	Clostridiales	52493	2031509
Sample_LPB01.mb.61cbin.1	56.50	Clostridiales	13253	2403089
Sample_LPB01.Cluster9682	36.10	Clostridiales	131250	2601528
Sample_LPB01.Cluster1317cbin.1	58.90	Bacteroidetes	27997	2940279
Sample_LPB01.Cluster7622cbin.1	41.10	Lachnospiraceae	79104	3062554
Sample_LPB01.Cluster10035	31.30	Firmicutes	45980	2283257
Sample_LPB01.mb.18	43.30	Bacteroidales	181281	2949958
Sample_LPB01.Cluster4014cbin.1	43.00	Bacteria	14453	3792122
Sample_LPB01.Cluster1905cbin.1	48.60	Bacteroidales	46540	3335621
Sample_LPB01.mb.95	29.60	Bacteria	17982	2455533
Sample_LPB01.mb.42	41.60	Clostridiales	24756	3264891
Sample_LPB01.mb.94cbin.1	43.40	Clostridiales	128931	2516644
Sample_LPB01.Cluster1739cbin.1	45.00	Bacteroidales	39382	4113223
Sample_LPB01.mb.75	59.10	Bacteroidetes	128162	3149001
Sample_LPB02.Cluster13993	36.50	Clostridiales	200166	1770897
Sample_LPB02.Cluster7087	45.30	Clostridiales	97171	1826822
Sample_LPB02.Cluster1263cbin.1	58.60	Clostridiales	13984	2662996
Sample_LPB02.Cluster10625cbin.1	45.50	Clostridiales	159676	2173839
Sample_LPB02.mb.18cbin.1	26.70	Bacteria	93037	1031817
Sample_LPB02.Cluster215cbin.1	56.00	Bacteria	7243	2214538
Sample_LPB02.Cluster3912cbin.1	49.70	Bacteroidales	75106	2632284
Sample_LPB02.Cluster10803cbin.1	41.90	Clostridiales	168898	2334603
Sample_LPB02.Cluster440	60.50	Bifidobacteriaceae	116262	1878911
Sample_LPB02.Cluster5269cbin.1	44.80	Prevotella	16907	2906901
Sample_LPB02.Cluster12684	38.40	Clostridiales	103826	2686419
Sample_LPB02.Cluster4672	49.50	Lachnospiraceae	65399	2794955
Sample_LPB02.mb.116	53.70	Clostridiales	210324	1885226
Sample_LPB02.Cluster13006cbin.1	36.10	Clostridiales	111806	3270796
Sample_LPB02.Cluster5645cbin.1	46.10	Bacteroidales	91464	2804211
Sample_LPB02.mb.117	59.50	Clostridiales	22537	2401460
Sample_LPB02.mb.32	58.90	Clostridiales	21801	2009811
Sample_LPB02.mb.28cbin.1	48.30	Clostridiales	105148	2830124
Sample_LPB02.mb.111	44.60	Clostridiales	153646	2226335
Sample_LPB02.Cluster9005cbin.1	41.70	Lachnospiraceae	63777	2505950
Sample_LPB02.mb.126	54.40	Bacteroidetes	57870	2193890
Sample_LPB02.mb.129	40.60	Clostridiales	126288	2141543
Sample_LPB02.mb.56	41.80	Lachnospiraceae	146663	2014493
Sample_LPB02.Cluster4541cbin.1	43.20	Bacteria	10999	3590476
Sample_LPB02.mb.48	60.30	Actinobacteria	8304	1911110
Sample_LPB02.mb.134	35.90	Clostridiales	184037	2286831
Sample_LPB02.mb.33cbin.1	44.10	Selenomonadales	129540	2103317
Sample_LPB02.mb.47	38.80	Clostridiales	73586	1902667
Sample_LPB02.mb.67	46.30	Clostridiales	36887	2581119
Sample_LPB02.Cluster5495	45.30	Bacteroidales	85080	3824745
Sample_LPB02.mb.89	62.30	Clostridiales	42563	1818318

Sample_LPB02.mb.86	56.70	Clostridiales	41486	2469025
Sample_LPB02.mb.26cbin.1	38.50	Lachnospiraceae	33031	2563012
Sample_LPB02.mb.96cbin.1	58.30	Clostridiales	16021	1926500
Sample_LPB02.mb.57cbin.1	50.40	Bacteroidales	76969	2359929
Sample_LPB02.mb.49	36.30	Clostridiales	134981	2523467
Sample_LPB02.mb.51	49.40	Proteobacteria	16871	2122919
Sample_LPB02.mb.4	45.50	Clostridiales	60921	2980394
Sample_LPB02.mb.68	59.10	Bacteroidetes	85560	2988255
Sample_LPB02.mb.62	42.10	Lachnospiraceae	13177	2147424
Sample_LPB02.mb.83	42.00	Bacteroidales	29527	4324194
Sample_LPB03.mb.17	27.20	Bacteria	260662	994960
Sample_LPB03.mb.18	24.30	Bacteria	53883	989306
Sample_LPB03.mb.24	26.50	Bacteria	94410	1184989
Sample_LPB03.Cluster8614cbin.1	37.00	Clostridiales	6532	1801825
Sample_LPB03.Cluster557cbin.1	54.80	Clostridia	182971	2746766
Sample_LPB03.mb.38	53.70	Clostridia	28044	1713524
Sample_LPB03.Cluster949cbin.1	58.20	Clostridiales	46155	2864803
Sample_LPB03.mb.4	46.70	Clostridiales	24126	1522689
Sample_LPB03.Cluster155	59.40	Clostridiales	14887	1970493
Sample_LPB03.mb.13	60.40	Clostridiales	64814	1783019
Sample_LPB03.mb.71cbin.1	25.20	Bacteria	154057	1295902
Sample_LPB03.mb.61	49.20	Clostridia	13040	1313559
Sample_LPB03.mb.53cbin.1	53.70	Clostridiales	10059	1963987
Sample_LPB03.Cluster637cbin.1	56.70	Clostridiales	84316	1974218
Sample_LPB03.Cluster4384cbin.1	46.60	Lachnospiraceae	6678	2106854
Sample_LPB03.mb.39	54.10	Actinobacteria	104900	1623313
Sample_LPB03.mb.74	25.70	Bacteria	52101	1393834
Sample_LPB03.mb.48	38.30	Clostridiales	66725	3090723
Sample_LPB03.mb.27	59.80	Actinobacteria	183497	2321320
Sample_LPB03.Cluster2870	52.80	Clostridiales	184996	2197660
Sample_LPB03.mb.79	49.40	Clostridiales	71288	1910180
Sample_LPB03.Cluster2773cbin.1	51.80	Clostridiales	169646	2230137
Sample_LPB03.mb.60	56.70	Clostridiales	13106	2470761
Sample_LPB03.Cluster1336	55.60	Bacteria	177370	2689521
Sample_LPB03.mb.28	40.80	Clostridiales	29770	2002164
Sample_LPB03.Cluster466	60.40	Bifidobacteriaceae	137551	1921717
Sample_LPB03.mb.46	47.90	Clostridiales	16811	1673532
Sample_LPB03.mb.75	58.70	Clostridiales	17845	1607493
Sample_LPB03.mb.83	62.20	Clostridiales	6876	1460615
Sample_LPB03.Cluster375	58.40	Bacteroidetes	101082	2574556
Sample_LPB03.mb.82cbin.1	60.80	Clostridiales	13898	2100794
Sample_LPB03.Cluster632	58.60	Bifidobacteriaceae	224860	2502552
Sample_LPB03.mb.93	62.20	Actinobacteria	103701	2159130
Sample_LPB03.mb.40	43.30	Clostridiales	11498	2585384
Sample_LPB03.mb.31	28.50	Clostridiales	58038	2945165
Sample_LPB03.mb.55cbin.1	38.70	Clostridiales	78312	2171925
Sample_LPB03.mb.86	42.60	Clostridiales	12873	3105003
Sample_LPB03.mb.78	56.90	Bacteroidetes	334938	2361632
Sample_LPB03.mb.50	46.10	Bacteroidales	79731	2976634
Sample_LPB03.mb.45cbin.1	62.00	Deltaproteobacteria	12284	3101838
Sample_LPB03.mb.47	46.40	Bacteroidales	80512	4385315
Sample_LPB03.Cluster4744cbin.1	50.60	Enterobacteriaceae	30818	4539006
Sample_LPB04.Cluster13416cbin.1	25.90	Bacteria	21940	1329976
Sample_LPB04.Cluster2924cbin.1	48.30	Clostridia	12156	1821501
Sample_LPB04.mb.12	50.30	Clostridiales	131334	2226051
Sample_LPB04.Cluster395	60.80	Clostridiales	44206	1596034
Sample_LPB04.mb.54	55.50	Clostridia	8000	2118392
Sample_LPB04.mb.25	49.00	Bacteria	93283	1983051

Sample_LPB04.mb.41	47.20	Clostridiales	96371	2092913
Sample_LPB04.Cluster591cbin.1	58.30	Bacteria	7274	2069239
Sample_LPB04.Cluster636	59.20	Clostridiales	61302	2148831
Sample_LPB04.mb.60	53.50	Clostridia	55400	1738267
Sample_LPB04.mb.49	57.40	Clostridiales	24414	1801143
Sample_LPB04.mb.27	61.90	Clostridiales	155666	1863672
Sample_LPB04.Cluster6113	49.80	Clostridiales	144604	2311880
Sample_LPB04.mb.36cbin.1	48.50	Clostridiales	11819	2718357
Sample_LPB04.mb.56cbin.1	59.80	Clostridiales	104028	2304411
Sample_LPB04.Cluster4480	54.00	Clostridiales	280090	1713568
Sample_LPB04.Cluster8893	46.10	Bacteroidetes	243367	2390750
Sample_LPB04.Cluster4cbin.1	59.10	Clostridiales	6284	2600074
Sample_LPB04.mb.32cbin.1	41.10	Clostridiales	167972	1943464
Sample_LPB04.Cluster13352	31.00	Euryarchaeota	111896	1791139
Sample_LPB04.Cluster2894cbin.1	56.70	Bifidobacteriaceae	10094	1856455
Sample_LPB04.Cluster24	63.60	Deltaproteobacteria	32031	2544565
Sample_LPB04.Cluster12970cbin.1	36.10	Clostridiales	28759	2449644
Sample_LPB04.Cluster2445	55.80	Bacteria	143811	3045963
Sample_LPB04.mb.47	59.90	Clostridiales	5541	1990245
Sample_LPB04.mb.76cbin.1	58.10	Clostridiales	48359	3004329
Sample_LPB04.mb.33	59.90	Bacteroidetes	30823	3082185
Sample_LPB04.mb.6cbin.1	56.70	Clostridiales	69057	1876378
Sample_LPB04.mb.53	59.80	Bacteroidetes	102517	2642070
Sample_LPB04.mb.61	48.30	Proteobacteria	23562	1702949
Sample_LPB04.Cluster10566	43.40	Bacteroidales	320281	3117883
Sample_LPB04.mb.29cbin.1	41.90	Lachnospiraceae	23880	2349458
Sample_LPB04.mb.67	43.40	Selenomonadales	23236	2141453
Sample_LPB04.Cluster7411cbin.1	43.10	Bacteria	5537	3298152
Sample_LPB04.mb.68	46.90	Lachnospiraceae	30862	3112220
Sample_LPB04.mb.69cbin.1	57.80	Clostridiales	90197	2429695
Sample_LPB04.mb.88	54.70	Clostridiales	7111	2478779
Sample_LPB04.mb.73	45.90	Clostridiales	11437	2241358
Sample_LPB04.Cluster7566	44.80	Bacteroidales	39992	3672806
Sample_LPB04.mb.90	58.60	Bacteroidetes	244471	2480442
Sample_LPB04.mb.64	58.90	Bacteroidetes	156777	3091334
Sample_LPB04.mb.18cbin.1	45.10	Bacteroidales	31935	4506684
Sample_LPB04.mb.51cbin.1	41.80	Bacteroidales	84555	3900788
Sample_LPB04.mb.9cbin.1	44.10	Clostridiales	167913	2752062
Sample_LPB04.mb.91cbin.1	37.10	Clostridiales	140942	2876587
Sample_LPB04.mb.7	41.30	Lachnospiraceae	32183	3910171
Sample_LPB04.mb.100cbin.1	41.50	Bacteroides	46635	5504692
Sample_LPB05.Cluster2433cbin.1	51.70	Bacteria	8080	1611574
Sample_LPB05.Cluster13939	28.40	Bacteria	184930	1673723
Sample_LPB05.Cluster4858	49.40	Clostridiales	97842	1887422
Sample_LPB05.Cluster4851cbin.1	48.10	Clostridiales	12084	1887940
Sample_LPB05.mb.12cbin.1	26.00	Bacteria	216521	1272105
Sample_LPB05.mb.112	53.60	Clostridia	35739	1728126
Sample_LPB05.Cluster7796cbin.1	48.60	Clostridiales	14898	2730401
Sample_LPB05.Cluster6861	48.60	Bacteria	309211	2226192
Sample_LPB05.Cluster12286cbin.1	39.80	Clostridiales	30077	3099658
Sample_LPB05.mb.3	56.00	Bacteria	33055	1934074
Sample_LPB05.mb.54cbin.1	48.10	Clostridia	24170	1901555
Sample_LPB05.mb.47	52.40	Clostridiales	43244	2140419
Sample_LPB05.Cluster11235	40.80	Clostridiales	140320	2112585
Sample_LPB05.mb.50	62.70	Clostridia	13267	2732861
Sample_LPB05.Cluster7261	43.40	Selenomonadales	141683	2363134
Sample_LPB05.mb.109cbin.1	61.80	Clostridiales	15984	2641611
Sample_LPB05.mb.16cbin.1	59.80	Clostridiales	116134	2488103

Sample_LPB05.Cluster3159	54.60	Bacteroidetes	202443	2871580
Sample_LPB05.mb.100	48.20	Clostridiales	18532	1707969
Sample_LPB05.mb.66	27.20	Bacteria	73915	1312827
Sample_LPB05.mb.37cbin.1	61.90	Clostridiales	19553	1715007
Sample_LPB05.mb.17	40.90	Clostridiales	56610	3370528
Sample_LPB05.Cluster3708cbin.1	53.20	Clostridiales	205757	2327367
Sample_LPB05.Cluster9022	44.50	Clostridiales	119142	1946581
Sample_LPB05.Cluster10054	43.90	Clostridiales	195621	2885436
Sample_LPB05.Cluster4298cbin.1	55.40	Bacteria	127223	2941868
Sample_LPB05.mb.117	56.10	Clostridiales	56957	2456228
Sample_LPB05.mb.119	46.10	Clostridiales	49258	2372012
Sample_LPB05.Cluster5602	46.90	Lachnospiraceae	135744	7039417
Sample_LPB05.mb.29cbin.1	43.70	Lachnospiraceae	101148	3362319
Sample_LPB05.Cluster11890cbin.1	38.40	Bacteroidales	28718	3461885
Sample_LPB05.mb.69	57.00	Clostridiales	76757	1760191
Sample_LPB05.mb.62	58.30	Bacteroidetes	83279	2389378
Sample_LPB05.mb.26	59.50	Clostridiales	78720	3317226
Sample_LPB05.mb.96cbin.1	47.00	Clostridiales	80107	2051555
Sample_LPB05.mb.71	57.00	Clostridiales	92204	2028880
Sample_LPB05.mb.65	60.20	Actinobacteria	22587	2000534
Sample_LPB05.mb.7	59.50	Clostridiales	8650	2240845
Sample_LPB05.mb.82	59.80	Clostridiales	78100	2758924
Sample_LPB05.mb.84	60.20	Bacteroidetes	38972	2198983
Sample_LPB05.Cluster6812cbin.1	43.30	Bacteroidales	133894	5193983
Sample_LPB05.mb.75	37.20	Clostridiales	85301	2722213
Sample_LPB05.mb.93cbin.1	60.10	Bifidobacteriaceae	35434	2597161
Sample_LPB05.mb.94	58.80	Deltaproteobacteria	19589	2837737
Sample_LPB05.mb.64cbin.1	45.00	Bacteroidales	15194	4409073
Sample_LPB05.mb.95	47.50	Bacteroidales	20625	3747194
Sample_LPB05.mb.48cbin.1	50.70	Enterobacteriaceae	92108	4605195
Sample_LPB08.Cluster5246cbin.1	43.00	Clostridiales	77340	2889446
Sample_LPB08.mb.71	27.00	Bacteria	130680	1446091
Sample_LPB08.Cluster4938	37.40	Clostridiales	41853	3546580
Sample_LPB08.mb.16	36.00	Clostridiales	132194	2791785
Sample_LPB08.mb.20	61.60	Clostridiales	17630	2003260
Sample_LPB08.mb.57	61.90	Clostridiales	109944	1945895
Sample_LPB08.Cluster730	54.90	Bacteroidetes	173095	1958317
Sample_LPB08.mb.49	49.00	Clostridiales	8275	2366805
Sample_LPB08.Cluster4192	40.20	Clostridiales	203025	1778621
Sample_LPB08.mb.28cbin.1	54.00	Clostridiales	28691	1785531
Sample_LPB08.Cluster14cbin.1	60.00	Clostridiales	58976	2464120
Sample_LPB08.mb.33	46.50	Lachnospiraceae	70791	2942927
Sample_LPB08.mb.67	56.40	Clostridiales	36750	2887877
Sample_LPB08.mb.13cbin.1	43.40	Selenomonadales	147433	2408502
Sample_LPB08.mb.9cbin.1	60.00	Clostridiales	106360	1987138
Sample_LPB08.mb.12	54.60	Selenomonadales	35609	2084988
Sample_LPB08.mb.3	59.50	Bacteroidetes	133533	2703834
Sample_LPB08.mb.10cbin.1	45.30	Clostridiales	32089	2462954
Sample_LPB08.Cluster93	56.60	Bifidobacteriaceae	144920	2170002
Sample_LPB08.Cluster6413cbin.1	36.90	Clostridiales	9750	2787275
Sample_LPB08.mb.41cbin.1	50.30	Bacteroidales	65418	2252881
Sample_LPB08.Cluster4247	41.10	Lachnospiraceae	87170	3021040
Sample_LPB08.mb.6	60.10	Deltaproteobacteria	7831	3464686
Sample_LPB08.mb.25	43.40	Bacteroidales	68164	3147337
Sample_LPB08.Cluster4577cbin.1	41.40	Lachnospiraceae	61570	2757817
Sample_LPB08.mb.15cbin.1	48.20	Proteobacteria	60017	2778187
Sample_LPB08.mb.36cbin.1	43.10	Bacteria	21249	3877064
Sample_LPB08.Cluster3074cbin.1	46.40	Bacteroidales	134106	4263653

Sample_LPB08.mb.60cbin.1	45.20	Bacteroidales	136777	4269106
Sample_LPB08.mb.8	43.40	Bacteroidales	154995	4359428
Sample_LPC01.Cluster3588	46.50	Clostridiales	91864	1865526
Sample_LPC01.Cluster12	62.20	Clostridiales	13509	2018496
Sample_LPC01.mb.111	33.20	Clostridiales	103446	2242505
Sample_LPC01.Cluster2751	48.20	Clostridiales	105234	2879660
Sample_LPC01.mb.112	36.30	Clostridiales	121113	2352134
Sample_LPC01.mb.119	36.60	Clostridiales	43180	3033591
Sample_LPC01.mb.116	62.30	Clostridiales	10153	1719645
Sample_LPC01.Cluster1033cbin.1	54.90	Bacteroidetes	116622	1957388
Sample_LPC01.mb.68	60.80	Bacteria	36920	2131197
Sample_LPC01.mb.30cbin.1	58.40	Clostridiales	9074	2622509
Sample_LPC01.mb.2cbin.1	38.50	Clostridiales	96411	2486992
Sample_LPC01.Cluster396cbin.1	59.60	Clostridiales	16217	2212442
Sample_LPC01.Cluster1744	51.00	Clostridiales	125334	2294923
Sample_LPC01.mb.109cbin.1	48.40	Firmicutes	35224	1805613
Sample_LPC01.Cluster11550cbin.1	37.50	Clostridiales	19655	2468875
Sample_LPC01.Cluster5424	43.00	Clostridiales	146736	2458363
Sample_LPC01.mb.28cbin.1	40.70	Clostridiales	58192	3766307
Sample_LPC01.mb.124cbin.1	56.40	Clostridiales	13518	2392010
Sample_LPC01.Cluster6136	41.30	Lachnospiraceae	66181	2768218
Sample_LPC01.mb.105cbin.1	43.40	Lachnospiraceae	129470	2721360
Sample_LPC01.mb.90	46.10	Clostridiales	81988	2623352
Sample_LPC01.Cluster10977cbin.1	31.30	Firmicutes	31189	2311906
Sample_LPC01.mb.118	35.90	Clostridiales	132079	2502654
Sample_LPC01.mb.96	41.00	Clostridiales	75155	2708005
Sample_LPC01.mb.110cbin.1	49.30	Proteobacteria	13146	2054116
Sample_LPC01.mb.61	48.90	Clostridiales	27311	2760457
Sample_LPC01.mb.45	44.60	Clostridiales	118121	2426041
Sample_LPC01.mb.41	28.70	Clostridiales	67469	2600389
Sample_LPC01.mb.76cbin.1	29.90	Bacteria	12113	2335934
Sample_LPC01.mb.89cbin.1	33.70	Clostridiales	50652	2060372
Sample_LPC01.mb.91	46.80	Lachnospiraceae	31294	3149126
Sample_LPC01.mb.128cbin.1	44.00	Bacteroidales	63155	3697040
Sample_LPC01.mb.67cbin.1	43.20	Bacteroidales	8254	2637959
Sample_LPC01.mb.34cbin.1	48.80	Bacteroidales	54883	3327236
Sample_LPC01.mb.27cbin.1	45.10	Bacteroidales	69927	4131436
Sample_LPC02.Cluster15787	26.00	Bacteria	55967	1204271
Sample_LPC02.Cluster15690	28.50	Bacteria	469394	1664354
Sample_LPC02.Cluster14689	36.50	Clostridiales	208149	1779574
Sample_LPC02.Cluster9279cbin.1	43.60	Clostridiales	39485	2798499
Sample_LPC02.Cluster130cbin.1	59.00	Clostridiales	47346	2189756
Sample_LPC02.mb.123	49.40	Clostridiales	74762	1866814
Sample_LPC02.Cluster3115	51.90	Clostridiales	136971	2106991
Sample_LPC02.Cluster3628	53.90	Clostridiales	231882	1793178
Sample_LPC02.Cluster8220cbin.1	46.50	Clostridiales	84242	1716444
Sample_LPC02.mb.24	59.80	Clostridia	38419	2470373
Sample_LPC02.Cluster3411cbin.1	55.50	Bacteroidetes	14714	2539433
Sample_LPC02.Cluster4862	51.30	Clostridiales	160000	2149710
Sample_LPC02.Cluster12100	41.80	Clostridiales	161110	2426220
Sample_LPC02.Cluster2482	55.50	Bacteria	206762	2786344
Sample_LPC02.mb.52	26.70	Bacteria	68153	1170883
Sample_LPC02.Cluster596cbin.1	60.10	Bifidobacteriaceae	13944	2016131
Sample_LPC02.mb.122	41.00	Lachnospiraceae	88066	2588338
Sample_LPC02.mb.23	53.30	Clostridiales	47144	1967808
Sample_LPC02.Cluster9367cbin.1	44.60	Clostridiales	131968	2241202
Sample_LPC02.mb.17	56.90	Clostridiales	58979	2185085
Sample_LPC02.mb.47	58.70	Clostridiales	11212	2563510

Sample_LPC02.mb.21	59.50	Clostridiales	12724	2128521
Sample_LPC02.mb.12	58.10	Bacteroidetes	405279	2628208
Sample_LPC02.mb.125	54.90	Bacteroidetes	105497	2107243
Sample_LPC02.mb.109	44.50	Clostridiales	59999	2580952
Sample_LPC02.mb.15	38.90	Clostridiales	74987	1758713
Sample_LPC02.mb.76	48.10	Clostridiales	8902	1756585
Sample_LPC02.mb.70cbin.1	45.20	Clostridiales	129559	1992037
Sample_LPC02.mb.60	56.90	Clostridiales	17854	2283408
Sample_LPC02.Cluster5884	46.20	Bacteroidales	58673	3177351
Sample_LPC02.mb.69cbin.1	48.70	Clostridiales	32470	2585714
Sample_LPC02.mb.127	42.10	Lachnospiraceae	10689	2197620
Sample_LPC02.mb.80cbin.1	56.10	Clostridiales	33559	2403694
Sample_LPC02.mb.7cbin.1	59.40	Clostridiales	17016	1896763
Sample_LPC02.mb.58	59.50	Clostridiales	32561	2274890
Sample_LPC02.Cluster8316	45.40	Bacteroidales	131161	3963386
Sample_LPC02.mb.87	59.50	Bacteroidetes	138188	2882102
Sample_LPC02.mb.90cbin.1	59.50	Bacteroidetes	95685	2855469
Sample_LPC02.mb.51	43.30	Bacteria	42498	3858901
Sample_LPC02.mb.79	42.00	Bacteroidales	57925	4949969
Sample_LPC02.mb.99	45.00	Bacteroidales	147779	4688929
Sample_LPC03.Cluster13638	36.50	Clostridiales	249695	1765818
Sample_LPC03.Cluster353cbin.1	58.20	Clostridiales	50640	2793093
Sample_LPC03.Cluster11360cbin.1	41.50	Clostridiales	268502	2556661
Sample_LPC03.mb.122	30.60	Bacteria	50159	1677790
Sample_LPC03.Cluster8563	46.10	Clostridiales	168841	1697679
Sample_LPC03.Cluster8725	45.20	Clostridiales	120321	1670357
Sample_LPC03.Cluster12660cbin.1	38.20	Clostridiales	43769	3224200
Sample_LPC03.Cluster211cbin.1	60.70	Clostridiales	22907	1821209
Sample_LPC03.Cluster213cbin.1	60.50	Clostridiales	27023	1724599
Sample_LPC03.Cluster1592cbin.1	59.20	Clostridiales	19326	1922065
Sample_LPC03.Cluster1220cbin.1	56.00	Clostridiales	40526	2380193
Sample_LPC03.mb.128cbin.1	59.40	Clostridiales	123000	2132574
Sample_LPC03.mb.3	50.90	Bacteria	69521	1913878
Sample_LPC03.mb.35	29.30	Bacteria	76537	1576098
Sample_LPC03.Cluster9996cbin.1	41.50	Lachnospiraceae	6237	2837607
Sample_LPC03.mb.29cbin.1	52.50	Clostridiales	17275	2254167
Sample_LPC03.Cluster5010cbin.1	56.90	Bacteroidetes	268193	2578641
Sample_LPC03.Cluster2175	55.60	Bacteria	219767	2687061
Sample_LPC03.mb.94	58.50	Bacteria	15361	1521673
Sample_LPC03.Cluster1576cbin.1	52.90	Bacteroidales	69771	2383028
Sample_LPC03.mb.33cbin.1	57.40	Clostridiales	14201	1714474
Sample_LPC03.mb.19	60.20	Actinobacteria	17710	2065793
Sample_LPC03.mb.124	53.70	Clostridiales	280357	1905784
Sample_LPC03.mb.111cbin.1	39.30	Clostridiales	76937	2074530
Sample_LPC03.Cluster11cbin.1	62.20	Deltaproteobacteria	7470	2839403
Sample_LPC03.mb.40	50.80	Clostridiales	19602	2003714
Sample_LPC03.Cluster653	58.50	Bifidobacteriaceae	235399	2339744
Sample_LPC03.mb.108	59.60	Bacteroidetes	79318	2789054
Sample_LPC03.mb.51cbin.1	61.10	Clostridiales	24432	1963355
Sample_LPC03.Cluster4989cbin.1	51.20	Prevotella	118556	3165168
Sample_LPC03.mb.56	57.50	Clostridiales	6974	2187211
Sample_LPC03.mb.126	42.90	Clostridiales	251881	3013259
Sample_LPC03.mb.117cbin.1	53.80	Bacteroidales	106409	2499121
Sample_LPC03.mb.50cbin.1	53.80	Clostridiales	53782	2724723
Sample_LPC03.mb.28	43.50	Lachnospiraceae	98410	2639414
Sample_LPC03.mb.24cbin.1	46.40	Bacteroidales	85554	2692642
Sample_LPC03.mb.9cbin.1	48.70	Clostridiales	74330	2607915
Sample_LPC03.mb.71	54.70	Bacteroidetes	91933	2166226

Sample_LPC03.mb.90cbin.1	46.60	Lachnospiraceae	17705	2380566
Sample_LPC03.mb.86cbin.1	49.20	Bacteroidales	77724	2748000
Sample_LPC03.mb.11	47.10	Bacteroidales	30659	3749363
Sample_LPC03.mb.77	44.20	Bacteroidales	72053	3613345
Sample_LPC04.Cluster12914	28.50	Bacteria	12471	1339672
Sample_LPC04.Cluster148	62.50	Clostridia	142253	1958621
Sample_LPC04.Cluster8345	46.60	Clostridiales	388913	1550073
Sample_LPC04.Cluster6011cbin.1	49.70	Clostridiales	105009	2017835
Sample_LPC04.mb.111	63.50	Clostridia	61468	2214337
Sample_LPC04.mb.26	52.80	Clostridiales	9776	1810323
Sample_LPC04.mb.12	47.20	Clostridiales	96363	1889792
Sample_LPC04.mb.101cbin.1	47.90	Bacteria	25324	2204451
Sample_LPC04.mb.106	56.80	Clostridia	106553	3865528
Sample_LPC04.mb.30	59.20	Clostridia	11946	3192611
Sample_LPC04.mb.104	60.50	Clostridiales	14655	1885127
Sample_LPC04.Cluster1226cbin.1	57.30	Clostridiales	40687	2130079
Sample_LPC04.mb.57	26.40	Bacteria	70401	1317807
Sample_LPC04.mb.67	24.30	Bacteria	87056	1074055
Sample_LPC04.Cluster6453	43.90	Selenomonadales	47682	2007454
Sample_LPC04.mb.70	27.80	Bacteria	54413	1108397
Sample_LPC04.mb.22	60.00	Actinobacteria	74853	2081531
Sample_LPC04.mb.51cbin.1	42.30	Clostridiales	98777	2688142
Sample_LPC04.Cluster1402cbin.1	58.50	Bacteria	137420	2727673
Sample_LPC04.Cluster10775	41.00	Clostridiales	143148	1943651
Sample_LPC04.mb.47	59.30	Clostridiales	9849	2140369
Sample_LPC04.Cluster12998	31.00	Euryarchaeota	129944	1788453
Sample_LPC04.mb.55	49.10	Bacteria	54248	1936916
Sample_LPC04.mb.43cbin.1	62.30	Clostridiales	51010	2649566
Sample_LPC04.Cluster10936cbin.1	41.30	Clostridiales	214549	2599204
Sample_LPC04.Cluster554cbin.1	63.80	Deltaproteobacteria	14734	2545423
Sample_LPC04.mb.6	36.50	Clostridiales	21568	2387696
Sample_LPC04.mb.5	45.00	Clostridiales	16419	2451777
Sample_LPC04.Cluster2788	55.80	Bacteria	141886	3045756
Sample_LPC04.mb.65cbin.1	53.00	Clostridiales	34165	2401899
Sample_LPC04.mb.20	40.80	Clostridiales	15163	2431694
Sample_LPC04.mb.58	53.50	Clostridiales	63392	1922755
Sample_LPC04.mb.85	53.50	Clostridia	65817	1808529
Sample_LPC04.mb.32	58.40	Clostridiales	105606	2762812
Sample_LPC04.mb.89	53.30	Clostridia	50455	1856082
Sample_LPC04.mb.40	35.90	Clostridiales	11286	2286431
Sample_LPC04.mb.19cbin.1	37.10	Clostridiales	141239	2808388
Sample_LPC04.mb.99	59.40	Clostridia	12753	2142352
Sample_LPC04.mb.90	61.30	Clostridia	58839	2640543
Sample_LPC04.Cluster1843cbin.1	58.70	Bacteroidetes	158506	3101095
Sample_LPC04.mb.74	41.10	Bacteria	27874	2538102
Sample_LPC04.mb.8	61.10	Clostridiales	12958	1451444
Sample_LPC04.Cluster6674cbin.1	43.30	Bacteria	10303	3683228
Sample_LPC04.mb.95	37.70	Clostridiales	162211	2215743
Sample_LPC04.Cluster9196	43.40	Bacteroidales	156128	3105997
Sample_LPC04.mb.91cbin.1	59.60	Clostridiales	75485	2522894
Sample_LPC04.mb.105	45.60	Bacteroidales	16391	3767371
Sample_LPC04.mb.80cbin.1	36.60	Clostridiales	157351	2175220
Sample_LPC04.mb.87	59.80	Bacteroidetes	74714	2719790
Sample_LPC04.mb.28cbin.1	44.90	Bacteroidales	35915	4250869
Sample_LPC04.mb.69	43.10	Lachnospiraceae	59328	2944414
Sample_LPC04.mb.54	42.20	Bacteroides	54479	4741183
Sample_LPC05.Cluster7047	47.90	Clostridiales	121675	1996937
Sample_LPC05.mb.119cbin.1	62.10	Clostridiales	11886	1633152

Sample_LPC05.mb.14cbin.1	34.20	Clostridiales	110029	2682933
Sample_LPC05.Cluster11595cbin.1	45.10	Lachnospiraceae	129368	2411287
Sample_LPC05.Cluster12425	39.90	Clostridiales	84174	2008240
Sample_LPC05.Cluster11892cbin.1	43.30	Selenomonadales	279830	2448052
Sample_LPC05.mb.16cbin.1	43.60	Clostridiales	75146	2994676
Sample_LPC05.Cluster10737	42.90	Clostridiales	172424	2564362
Sample_LPC05.Cluster8509	44.40	Clostridiales	119490	1908004
Sample_LPC05.Cluster141cbin.1	56.90	Bifidobacteriaceae	26313	2057099
Sample_LPC05.mb.69cbin.1	50.50	Bacteria	60352	2032967
Sample_LPC05.mb.102	54.90	Bacteroidetes	71830	2005011
Sample_LPC05.Cluster9730	43.40	Lachnospiraceae	38703	1925337
Sample_LPC05.mb.100	36.60	Clostridiales	160203	2037844
Sample_LPC05.Cluster12574cbin.1	40.50	Clostridiales	164386	2770295
Sample_LPC05.Cluster9183	43.70	Clostridiales	171888	2276256
Sample_LPC05.mb.113cbin.1	60.00	Bacteroidetes	44871	2692171
Sample_LPC05.mb.24	56.40	Clostridiales	18506	2279983
Sample_LPC05.Cluster12851cbin.1	38.40	Bacteroidales	52576	3271340
Sample_LPC05.Cluster7412cbin.1	45.30	Clostridiales	72109	3321657
Sample_LPC05.mb.117	37.30	Clostridiales	11363	2431294
Sample_LPC05.Cluster13262cbin.1	43.00	Bacteria	5933	3318207
Sample_LPC05.mb.27cbin.1	46.20	Clostridiales	48990	2258674
Sample_LPC05.mb.52	48.50	Bacteria	9637	2276767
Sample_LPC05.mb.2	60.10	Clostridiales	15862	2874870
Sample_LPC05.Cluster4101cbin.1	45.50	Bacteroidales	41200	3318434
Sample_LPC05.Cluster726cbin.1	55.30	Proteobacteria	30433	2796483
Sample_LPC05.mb.76cbin.1	56.80	Clostridiales	89972	2021457
Sample_LPC05.mb.49	47.30	Lachnospiraceae	30795	3030655
Sample_LPC05.mb.95cbin.1	57.60	Clostridiales	95294	2352355
Sample_LPC05.mb.85	41.00	Clostridiales	13225	3301678
Sample_LPC05.mb.112	43.30	Bacteroidales	52990	2957174
Sample_LPC05.mb.78	58.90	Clostridiales	36611	1862129
Sample_LPC05.mb.20	41.60	Lachnospiraceae	68227	3237038
Sample_LPC05.Cluster10950cbin.1	42.10	Bacteroidales	30751	4356595
Sample_LPC05.mb.25cbin.1	47.90	Bacteroidales	70863	3779230
Sample_LPC08.mb.20cbin.1	42.10	Bacteria	35799	4937448
Sample_LPC08.mb.5	27.00	Bacteria	140182	1463455
Sample_LPC08.mb.17	36.40	Clostridiales	19673	2369877
Sample_LPC08.mb.54cbin.1	42.90	Clostridiales	104230	2847270
Sample_LPC08.mb.13	62.00	Clostridiales	144677	1924056
Sample_LPC08.mb.55cbin.1	37.80	Clostridiales	47548	3251847
Sample_LPC08.mb.15	60.40	Clostridiales	34313	2332145
Sample_LPC08.mb.41cbin.1	54.80	Bacteroidetes	172816	1971210
Sample_LPC08.mb.10	46.30	Lachnospiraceae	68255	3187118
Sample_LPC08.Cluster2113cbin.1	43.40	Selenomonadales	291829	2417134
Sample_LPC08.Cluster5547	32.70	Lactobacillales	8024	1622014
Sample_LPC08.Cluster147cbin.1	54.50	Selenomonadales	14756	2097697
Sample_LPC08.Cluster4552cbin.1	40.50	Clostridiales	237540	2071415
Sample_LPC08.mb.26	29.20	Bacteria	34809	2460301
Sample_LPC08.Cluster227	56.90	Bifidobacteriaceae	198329	2114232
Sample_LPC08.mb.31cbin.1	59.00	Bacteroidetes	130127	2917074
Sample_LPC08.mb.42	59.70	Deltaproteobacteria	7115	3407048
Sample_LPC08.mb.44cbin.1	50.30	Bacteroidales	68770	2273695
Sample_LPC08.Cluster4945cbin.1	40.90	Lachnospiraceae	69974	3208459
Sample_LPC08.Cluster5239cbin.1	43.00	Bacteria	8018	3488129
Sample_LPC08.mb.1cbin.1	43.50	Bacteroidales	26103	3079525
Sample_LPC08.mb.33cbin.1	48.10	Proteobacteria	14417	2668888
Sample_LPC08.mb.49	41.60	Lachnospiraceae	59403	2496096
Sample_LPC08.Cluster2875cbin.1	46.50	Bacteroidales	152237	4173609

Sample_LPC08.Cluster1848cbin.1	45.30	Bacteroidales	133555	4238796
Sample_SCA55.Cluster12046	29.60	Bacteria	16761	1231757
Sample_SCA55.mb.104cbin.1	47.20	Clostridia	31167	1453225
Sample_SCA55.Cluster2874	51.70	Clostridiales	94168	1944123
Sample_SCA55.Cluster7572cbin.1	42.10	Clostridiales	22220	2543190
Sample_SCA55.Cluster4326cbin.1	46.50	Clostridiales	93141	1837155
Sample_SCA55.Cluster3804cbin.1	48.90	Clostridiales	16021	2607740
Sample_SCA55.Cluster3130cbin.1	51.60	Bacteroidetes	110335	3340115
Sample_SCA55.Cluster2493	53.70	Clostridiales	211704	1842578
Sample_SCA55.mb.44	27.90	Euryarchaeota	169592	1776570
Sample_SCA55.Cluster10485	37.70	Lactobacillales	27657	1677449
Sample_SCA55.mb.27	33.70	Bacteria	8011	1770439
Sample_SCA55.mb.23	45.10	Clostridiales	90715	2460962
Sample_SCA55.Cluster11573cbin.1	36.80	Gammaproteobacteria	87029	2283824
Sample_SCA55.Cluster6699	44.60	Clostridiales	90507	2201472
Sample_SCA55.Cluster790	58.20	Veillonellaceae	330188	2485262
Sample_SCA55.Cluster12012	36.00	Clostridiales	159492	2465552
Sample_SCA55.mb.7cbin.1	36.50	Clostridiales	149174	1758312
Sample_SCA55.mb.4cbin.1	51.40	Bacteroidales	46292	2484072
Sample_SCA55.Cluster8267	41.70	Lachnospiraceae	70058	2811347
Sample_SCA55.mb.52cbin.1	59.80	Actinobacteria	51547	2120103
Sample_SCA55.mb.97	32.10	Bacteria	371778	1929365
Sample_SCA55.mb.38	44.50	Lachnospiraceae	19984	2124603
Sample_SCA55.mb.21cbin.1	50.60	Lachnospiraceae	9759	2423601
Sample_SCA55.mb.85	52.90	Clostridiales	31717	2138071
Sample_SCA55.mb.45cbin.1	37.20	Clostridiales	129317	2707381
Sample_SCA55.mb.60	59.80	Clostridiales	28744	2003200
Sample_SCA55.mb.87	38.70	Clostridiales	69113	2065371
Sample_SCA55.mb.110cbin.1	42.10	Lachnospiraceae	71526	3953623
Sample_SCA55.mb.95	58.70	Clostridiales	40506	2340306
Sample_SCA55.mb.99cbin.1	56.00	Enterobacteriaceae	217734	5272826
Sample_SCA58.Cluster16595	25.50	Bacteria	8002	1018072
Sample_SCA58.Cluster16706cbin.1	24.20	Bacteria	70716	989478
Sample_SCA58.Cluster1102	58.60	Clostridia	76492	2355909
Sample_SCA58.Cluster4228cbin.1	49.70	Clostridiales	66821	2085773
Sample_SCA58.mb.102	48.60	Clostridiales	88622	1752485
Sample_SCA58.Cluster4693	54.20	Actinobacteria	328091	1719666
Sample_SCA58.Cluster15781	34.20	Bacilli	48882	2673778
Sample_SCA58.mb.118cbin.1	49.70	Clostridiales	71452	1973563
Sample_SCA58.Cluster605	59.30	Clostridiales	48003	2073175
Sample_SCA58.mb.25cbin.1	48.70	Clostridia	372070	1521699
Sample_SCA58.Cluster1069cbin.1	56.60	Clostridiales	8389	2271980
Sample_SCA58.Cluster6503cbin.1	47.90	Clostridiales	47294	1809777
Sample_SCA58.mb.121	62.30	Clostridiales	13339	1457741
Sample_SCA58.Cluster13327	40.90	Clostridiales	177199	1973467
Sample_SCA58.Cluster10143	44.60	Clostridiales	146897	2132865
Sample_SCA58.Cluster1691	55.70	Bacteria	175223	2785556
Sample_SCA58.mb.20	62.00	Clostridiales	9289	1872844
Sample_SCA58.Cluster10382cbin.1	44.20	Clostridiales	72323	2687952
Sample_SCA58.mb.5	53.90	Clostridia	13609	1631094
Sample_SCA58.Cluster864	57.80	Proteobacteria	23432	2121184
Sample_SCA58.mb.129cbin.1	57.70	Clostridiales	63327	2390901
Sample_SCA58.mb.31cbin.1	49.50	Clostridiales	5620	1851150
Sample_SCA58.mb.35	58.10	Clostridiales	54639	3080501
Sample_SCA58.mb.4cbin.1	60.00	Clostridiales	103246	2063676
Sample_SCA58.mb.107	59.90	Bacteroidetes	110989	2678183
Sample_SCA58.mb.80	26.70	Bacteria	128480	1314474

Sample_SCA58.mb.43	62.40	Clostridiales	26529	1437038
Sample_SCA58.mb.15cbin.1	60.10	Bacteroidetes	146915	2439125
Sample_SCA58.mb.120cbin.1	50.90	Clostridiales	157222	2418217
Sample_SCA58.mb.46	55.00	Clostridiales	11816	2478546
Sample_SCA58.Cluster11257	43.30	Bacteroidales	158408	3104451
Sample_SCA58.mb.103	58.90	Bacteroidetes	173389	2877663
Sample_SCA58.mb.77	60.70	Actinobacteria	47400	1818609
Sample_SCA58.mb.27	60.90	Clostridiales	16990	2198391
Sample_SCA58.mb.63cbin.1	60.20	Clostridiales	71061	1922006
Sample_SCA58.mb.51	64.10	Actinobacteria	5674	2012404
Sample_SCA58.Cluster8627cbin.1	43.20	Bacteria	20003	3936390
Sample_SCA58.mb.123	41.90	Lachnospiraceae	21888	2258887
Sample_SCA58.mb.55cbin.1	59.90	Actinobacteria	26765	2305986
Sample_SCA58.Cluster8567	45.20	Bacteroidales	143688	4078969
Sample_SCA58.Cluster8833	45.40	Bacteroidales	110540	3987042
Sample_SCA58.mb.91cbin.1	61.60	Clostridiales	26169	2542084
Sample_SCA58.mb.56cbin.1	60.30	Bifidobacteriaceae	135928	2322483
Sample_SCA58.mb.117cbin.1	45.30	Bacteroidales	12479	3727943
Sample_SCA58.mb.86	54.80	Bacteroidetes	149642	2057189
Sample_SCA58.mb.38	41.40	Bacteroidales	45271	3615902
Sample_SCA58.Cluster8815	41.60	Bacteroidales	52066	4772262
Sample_SCA58.mb.135	56.00	Enterobacteriaceae	67703	4752581
Sample_SCA59.Cluster5865cbin.1	49.30	Clostridiales	72765	1801329
Sample_SCA59.Cluster17127	27.20	Euryarchaeota	65770	1748288
Sample_SCA59.mb.106	26.80	Bacteria	87015	1121148
Sample_SCA59.mb.124	26.50	Bacteria	99599	1255816
Sample_SCA59.Cluster179	58.50	Clostridiales	121478	2390478
Sample_SCA59.Cluster9717	44.90	Actinobacteria	216632	1530700
Sample_SCA59.Cluster17197cbin.1	31.10	Euryarchaeota	218229	1735250
Sample_SCA59.Cluster10905	42.90	Clostridiales	157632	1937266
Sample_SCA59.Cluster12937cbin.1	40.50	Clostridiales	253712	2171978
Sample_SCA59.Cluster4667cbin.1	48.60	Firmicutes	32914	1876784
Sample_SCA59.Cluster12108	32.60	Lactobacillales	11041	1783607
Sample_SCA59.Cluster3650cbin.1	51.80	Clostridiales	172626	2294004
Sample_SCA59.Cluster3610cbin.1	51.00	Bacteroidales	14894	2050645
Sample_SCA59.Cluster227	58.60	Bacteria	16514	2840744
Sample_SCA59.mb.120cbin.1	42.00	Lachnospiraceae	12554	1895705
Sample_SCA59.mb.133cbin.1	57.30	Clostridiales	74964	1877178
Sample_SCA59.mb.26	59.30	Actinobacteria	64644	1697135
Sample_SCA59.mb.15	43.80	Clostridiales	65052	2779570
Sample_SCA59.Cluster9650cbin.1	46.00	Clostridiales	41752	2146343
Sample_SCA59.Cluster6328cbin.1	45.20	Prevotella	13407	3263620
Sample_SCA59.mb.50	53.90	Clostridia	16010	1706121
Sample_SCA59.mb.102	44.40	Clostridiales	39338	2557205
Sample_SCA59.mb.24	52.20	Clostridiales	13045	1787312
Sample_SCA59.mb.30cbin.1	37.10	Clostridiales	28101	2773123
Sample_SCA59.mb.121	43.70	Lachnospiraceae	111204	2618473
Sample_SCA59.mb.18	58.70	Bacteroidetes	44772	2455094
Sample_SCA59.mb.65	58.80	Clostridia	44358	2845867
Sample_SCA59.mb.117cbin.1	58.80	Bifidobacteriaceae	17194	2223854
Sample_SCA59.mb.51	61.10	Clostridiales	52648	1676631
Sample_SCA59.mb.48cbin.1	57.90	Clostridiales	26616	2080317
Sample_SCA59.mb.115	41.70	Lachnospiraceae	26031	2676184
Sample_SCA59.mb.46	49.50	Clostridiales	17487	2339438
Sample_SCA59.Cluster7336	43.20	Bacteria	67155	4084862
Sample_SCA59.mb.126	42.10	Lachnospiraceae	29753	2162322
Sample_SCA59.mb.81	62.50	Clostridiales	20585	1625358
Sample_SCA59.mb.36	41.30	Clostridiales	233217	2569866

Sample_SCA59.mb.130	43.80	Bacteria	82837	4241656
Sample_SCA59.Cluster8336	45.30	Bacteroidales	136143	4061519
Sample_SCA59.Cluster5138cbin.1	45.10	Bacteroidales	15147	4321504
Sample_SCA59.mb.69cbin.1	63.20	Actinobacteria	13837	2853971
Sample_SCA59.mb.52	36.10	Clostridiales	23610	2425888
Sample_SCA59.mb.88	60.10	Bacteroidetes	48483	2327408
Sample_SCA59.mb.86	54.90	Bacteroidetes	517187	2813988
Sample_SCA59.mb.59cbin.1	62.50	Deltaproteobacteria	6920	2763734
Sample_SCA59.mb.90cbin.1	47.30	Bacteroidales	20237	3751082
Sample_SCA60.Cluster15187	29.40	Bacteria	10625	1467804
Sample_SCA60.mb.102	52.90	Clostridiales	74824	2427178
Sample_SCA60.mb.17cbin.1	57.90	Clostridiales	49975	3022995
Sample_SCA60.mb.3	56.00	Clostridiales	33199	1763922
Sample_SCA60.Cluster9736cbin.1	43.10	Clostridiales	70140	3114210
Sample_SCA60.Cluster5985cbin.1	47.90	Clostridiales	83559	3054085
Sample_SCA60.Cluster2624cbin.1	54.50	Bacteroidetes	104344	2031884
Sample_SCA60.Cluster131	51.80	Clostridiales	30729	1972160
Sample_SCA60.Cluster874	59.80	Actinobacteria	146638	2187569
Sample_SCA60.Cluster2331cbin.1	52.20	Firmicutes	56886	2448494
Sample_SCA60.mb.22	55.60	Clostridiales	40214	2448469
Sample_SCA60.Cluster560cbin.1	56.20	Clostridiales	16553	2546179
Sample_SCA60.Cluster7540cbin.1	43.20	Lachnospiraceae	5957	1742322
Sample_SCA60.Cluster2720	55.60	Bacteria	183414	2685368
Sample_SCA60.Cluster11954cbin.1	39.80	Clostridiales	100993	2280493
Sample_SCA60.mb.84	24.20	Bacteria	53856	1027488
Sample_SCA60.mb.24	40.70	Clostridiales	15896	3797038
Sample_SCA60.Cluster11269cbin.1	41.00	Clostridiales	7050	2404270
Sample_SCA60.Cluster9931	40.80	Clostridiales	121776	1944680
Sample_SCA60.mb.25	60.40	Bacteroidetes	83793	2348060
Sample_SCA60.mb.13cbin.1	45.40	Clostridiales	68944	1994476
Sample_SCA60.mb.52cbin.1	58.60	Clostridiales	5212	1593586
Sample_SCA60.mb.104cbin.1	60.30	Bacteroidetes	33989	2509637
Sample_SCA60.Cluster10224cbin.1	41.40	Lachnospiraceae	88211	2968389
Sample_SCA60.mb.18	41.70	Clostridiales	182190	2470232
Sample_SCA60.mb.81cbin.1	32.80	Bacteria	73567	1918465
Sample_SCA60.mb.96cbin.1	47.60	Clostridiales	25955	1478531
Sample_SCA60.mb.86	36.40	Clostridiales	11038	1826438
Sample_SCA60.Cluster10911	41.60	Clostridiales	34677	2907350
Sample_SCA60.mb.67cbin.1	59.40	Bacteria	75588	4109085
Sample_SCA60.mb.77	49.60	Clostridiales	45623	2213397
Sample_SCA60.Cluster5689cbin.1	46.00	Bacteroidales	81892	3546359
Sample_SCA60.mb.87	62.60	Clostridiales	31903	1932816
Sample_SCA60.mb.91	57.20	Bacteria	56226	2528946
Sample_SCA60.mb.83cbin.1	45.60	Clostridiales	25631	1635989
Sample_SCA60.mb.2cbin.1	43.50	Bacteroidales	162200	3014229
Sample_SCA60.mb.9cbin.1	47.20	Clostridiales	86766	2560773
Sample_SCA60.mb.57cbin.1	37.30	Clostridiales	50742	2781560
Sample_SCA60.Cluster3577cbin.1	45.20	Bacteroidales	9516	4074368
Sample_SCA60.mb.90cbin.1	47.10	Lachnospiraceae	58069	3227368
Sample_SCA60.mb.1	41.80	Bacteroidales	37702	3884369
Sample_SCA60.mb.48cbin.1	43.30	Bacteria	49006	4030832
Sample_SCA60.mb.92cbin.1	26.90	Clostridiales	51178	2742095
Sample_SCA60.Cluster205cbin.1	51.00	Enterobacteriaceae	24480	4684343
Sample_SCA61.mb.12	47.20	Bacteria	25914	1746227
Sample_SCA61.mb.105	58.20	Clostridiales	59067	2975325
Sample_SCA61.mb.25	38.30	Clostridiales	13589	2354516
Sample_SCA61.Cluster3845cbin.1	46.70	Lachnospiraceae	102028	2135180
Sample_SCA61.mb.2	61.00	Clostridiales	76314	1902541

Sample_SCA61.mb.27	56.60	Clostridiales	61120	2467029
Sample_SCA61.Cluster430	58.40	Bacteroidetes	118446	2549279
Sample_SCA61.mb.26	58.10	Clostridiales	66387	2297952
Sample_SCA61.Cluster6157cbin.1	40.90	Clostridiales	177443	2026691
Sample_SCA61.Cluster26cbin.1	59.90	Clostridiales	6973	1816355
Sample_SCA61.Cluster7291cbin.1	38.00	Clostridiales	178746	1879982
Sample_SCA61.mb.47	57.90	Clostridiales	33546	2085524
Sample_SCA61.Cluster572	57.30	Proteobacteria	109781	2367611
Sample_SCA61.Cluster8403cbin.1	27.10	Clostridiales	50263	2662540
Sample_SCA61.Cluster6202cbin.1	37.50	Clostridiales	10575	2454611
Sample_SCA61.Cluster4540	42.30	Lachnospiraceae	15482	2513537
Sample_SCA61.mb.16	44.10	Clostridiales	28321	3076604
Sample_SCA61.mb.52cbin.1	52.00	Clostridiales	110974	2369266
Sample_SCA61.mb.73	62.60	Clostridiales	25411	1753002
Sample_SCA61.mb.23cbin.1	44.10	Clostridiales	216190	2758359
Sample_SCA61.mb.48	43.50	Clostridiales	9752	2028303
Sample_SCA61.Cluster3533cbin.1	43.40	Bacteria	10711	3729143
Sample_SCA61.mb.63	55.30	Bacteria	319131	2677454
Sample_SCA61.mb.95cbin.1	48.10	Clostridiales	102844	2736216
Sample_SCA61.mb.66cbin.1	59.90	Bacteroidetes	148939	2569387
Sample_SCA61.mb.80cbin.1	60.00	Bacteroidetes	235593	2690281
Sample_SCA61.Cluster3102cbin.1	44.40	Bacteroidales	100346	3512294
Sample_SCA61.Cluster3246	45.00	Bacteroidales	95051	4434214
Sample_SCA61.Cluster5366cbin.1	41.80	Bacteroidales	17329	4759122
Sample_SCA61.Cluster3521cbin.1	46.50	Bacteroidales	172786	4329041
Sample_SCA61.mb.96cbin.1	45.70	Bacteroidales	45437	3708770
Sample_SCA64.Cluster2709	49.30	Clostridiales	73245	1764700
Sample_SCA64.Cluster428cbin.1	58.10	Clostridiales	61960	2929514
Sample_SCA64.mb.101	62.00	Clostridiales	14740	1619671
Sample_SCA64.mb.68cbin.1	60.50	Bacteria	30930	1841965
Sample_SCA64.Cluster4036	44.90	Clostridiales	125368	2570789
Sample_SCA64.mb.25	60.20	Clostridiales	53933	1912619
Sample_SCA64.mb.36cbin.1	45.10	Actinobacteria	18159	1888193
Sample_SCA64.mb.102	61.50	Clostridiales	49433	2418596
Sample_SCA64.mb.33	56.40	Clostridiales	101972	2344695
Sample_SCA64.Cluster5527cbin.1	40.80	Clostridiales	150336	2077667
Sample_SCA64.Cluster1692cbin.1	51.90	Firmicutes	50036	2548179
Sample_SCA64.mb.51	51.30	Clostridiales	38320	2114775
Sample_SCA64.mb.13cbin.1	55.60	Bacteroidetes	24491	2579437
Sample_SCA64.mb.1cbin.1	59.90	Bifidobacteriaceae	11929	1792802
Sample_SCA64.mb.43cbin.1	60.10	Actinobacteria	13084	1943158
Sample_SCA64.mb.10	59.30	Bacteroidetes	130105	2741646
Sample_SCA64.Cluster336	58.10	Bacteria	185474	3119347
Sample_SCA64.Cluster4286cbin.1	43.40	Clostridiales	154624	2999207
Sample_SCA64.Cluster981	55.50	Proteobacteria	161344	2279582
Sample_SCA64.Cluster6262cbin.1	37.20	Clostridiales	55885	2804004
Sample_SCA64.mb.4cbin.1	37.70	Lactobacillales	38835	1768873
Sample_SCA64.Cluster4525	43.40	Bacteroidales	164047	3024422
Sample_SCA64.mb.91	57.70	Clostridiales	25995	2117734
Sample_SCA64.mb.48	55.70	Bacteroidales	13363	2420626
Sample_SCA64.mb.99	57.00	Clostridiales	44808	2385873
Sample_SCA64.mb.79cbin.1	63.80	Actinobacteria	11070	2335269
Sample_SCA64.Cluster3798cbin.1	40.90	Lachnospiraceae	51561	3736133
Sample_SCA64.mb.65cbin.1	46.20	Lachnospiraceae	65937	3551097
Sample_SCA64.mb.81	54.50	Bacteroidetes	100805	2154870
Sample_SCA64.mb.75cbin.1	59.90	Bifidobacteriaceae	99760	2212755
Sample_SCA64.mb.60	59.20	Bacteroidetes	53905	2750262
Sample_SCA64.Cluster2169cbin.1	46.80	Bacteroidales	33976	3879170

Sample_SCA64.mb.9	57.90	Clostridiales	23811	1885899
Sample_SCA64.Cluster2443	45.60	Bacteroidales	42467	3884223
Sample_SCA64.mb.93cbin.1	29.90	Bacteria	46159	2524755
Sample_SCA64.Cluster3337	45.00	Bacteroidales	226536	4579543
Sample_SCA64.mb.74cbin.1	44.50	Bacteroidales	66312	2915184
Sample_SCA64.mb.77	49.00	Bacteroidales	79719	3240195
Sample_SCA64.mb.44	41.50	Bacteroidales	149274	4930952
Sample_SCA65.Cluster3577cbin.1	49.40	Bacteria	64523	1775109
Sample_SCA65.mb.10	52.60	Bacteria	42222	2051640
Sample_SCA65.mb.19	38.00	Clostridiales	56150	2648006
Sample_SCA65.Cluster164cbin.1	60.40	Clostridiales	57863	2003443
Sample_SCA65.mb.27cbin.1	54.70	Actinobacteria	9970	1413915
Sample_SCA65.Cluster3800	48.40	Clostridiales	108602	2806003
Sample_SCA65.mb.13	60.90	Clostridiales	55192	2337303
Sample_SCA65.mb.7	55.60	Clostridiales	8313	2157357
Sample_SCA65.Cluster1800cbin.1	55.00	Bacteroidetes	126040	1946780
Sample_SCA65.Cluster3067cbin.1	43.90	Selenomonadales	9179	2060210
Sample_SCA65.mb.24cbin.1	50.80	Clostridiales	127698	2825884
Sample_SCA65.mb.79	62.10	Clostridiales	38055	2095662
Sample_SCA65.Cluster1344	55.40	Bacteria	167612	2814531
Sample_SCA65.mb.14cbin.1	44.50	Clostridiales	114117	2445604
Sample_SCA65.mb.16	42.90	Clostridiales	101097	2574371
Sample_SCA65.mb.62	41.20	Clostridiales	167138	2411000
Sample_SCA65.mb.61	59.20	Bacteroidetes	124193	2883125
Sample_SCA65.mb.82	59.30	Bacteroidetes	19701	2145927
Sample_SCA65.mb.43	40.50	Clostridiales	155868	2469088
Sample_SCA65.mb.71cbin.1	38.60	Clostridiales	99896	2089761
Sample_SCA65.mb.56cbin.1	39.30	Streptococcus	13834	2160731
Sample_SCA65.mb.92	53.50	Clostridiales	503395	1931896
Sample_SCA65.mb.54cbin.1	37.50	Clostridiales	99831	2754805
Sample_SCA65.mb.96	63.10	Actinobacteria	165972	2972303
Sample_SCA65.Cluster4304cbin.1	42.20	Lachnospiraceae	100903	3631692
Sample_SCA65.Cluster4074cbin.1	42.20	Bacteria	12896	4101322
Sample_SCA65.Cluster3562cbin.1	46.80	Bacteroidales	141755	3985892
Sample_SCA65.mb.66cbin.1	42.20	Bacteroidales	43280	4155413
Sample_SCA65.mb.37cbin.1	45.00	Bacteroidales	143452	4709717
Sample_SCA65.mb.25	43.30	Bacteroidales	175554	5761772
Sample_SCA66.mb.53	49.40	Clostridiales	28092	1918911
Sample_SCA66.Cluster55cbin.1	60.30	Clostridiales	21180	1948679
Sample_SCA66.Cluster1328cbin.1	52.70	Clostridiales	49438	2132576
Sample_SCA66.mb.82	26.10	Bacteria	40615	1330018
Sample_SCA66.Cluster3505cbin.1	46.80	Lactobacillales	72412	1989369
Sample_SCA66.mb.64cbin.1	36.40	Clostridiales	102123	1947956
Sample_SCA66.mb.24cbin.1	48.30	Clostridiales	24598	2849433
Sample_SCA66.mb.49cbin.1	59.40	Clostridiales	11408	1883488
Sample_SCA66.Cluster1226	54.80	Bacteroidetes	142280	1987720
Sample_SCA66.Cluster241cbin.1	57.30	Clostridiales	42438	3221308
Sample_SCA66.Cluster4855cbin.1	44.00	Clostridiales	159303	2045865
Sample_SCA66.Cluster7665	40.60	Clostridiales	278928	2121962
Sample_SCA66.Cluster283	55.60	Bacteria	204220	2717404
Sample_SCA66.mb.83cbin.1	58.20	Clostridiales	50519	2986846
Sample_SCA66.Cluster963	56.60	Bifidobacteriaceae	106606	2060479
Sample_SCA66.mb.39cbin.1	60.20	Bifidobacteriaceae	26990	2585226
Sample_SCA66.mb.22cbin.1	42.70	Clostridiales	86126	2857585
Sample_SCA66.mb.40	43.50	Lachnospiraceae	76801	2933026
Sample_SCA66.Cluster1132cbin.1	55.10	Proteobacteria	114877	2865709
Sample_SCA66.mb.7	44.30	Clostridiales	59211	2652717
Sample_SCA66.Cluster5969	43.70	Bacteroidales	336681	3214694

Sample_SCA66.mb.57	26.80	Clostridiales	35551	2725497
Sample_SCA66.mb.59cbin.1	40.90	Lachnospiraceae	39272	3186003
Sample_SCA66.mb.80cbin.1	59.70	Bacteroidetes	144124	2818074
Sample_SCA66.mb.35	43.00	Lachnospiraceae	71208	3181730
Sample_SCA66.mb.71	59.50	Bacteroidetes	50026	2576988
Sample_SCA66.Cluster6953cbin.1	43.10	Bacteria	79454	3930120
Sample_SCA66.mb.84	46.70	Lachnospiraceae	54321	3331815
Sample_SCA66.mb.44	59.20	Bacteroidetes	41898	3117763
Sample_SCA66.Cluster4395cbin.1	45.00	Bacteroidales	122841	4455315
Sample_SCA66.mb.92cbin.1	40.70	Bacteroidales	38666	3013469
Sample_SCA66.mb.62cbin.1	41.20	Bacteroidales	68215	4463504
Sample_SCA66.mb.88cbin.1	46.30	Bacteroidales	19788	3653648
Sample_SCA67.mb.128cbin.1	53.90	Clostridia	9749	1610926
Sample_SCA67.mb.121	52.90	Clostridia	71950	2109906
Sample_SCA67.mb.106	58.30	Clostridiales	71319	2008215
Sample_SCA67.mb.59	26.30	Bacteria	46036	1308293
Sample_SCA67.mb.103	56.10	Clostridiales	18817	2258235
Sample_SCA67.mb.111cbin.1	51.80	Clostridiales	14163	2095038
Sample_SCA67.Cluster5052	51.40	Bacteroidales	51966	2232864
Sample_SCA67.mb.29cbin.1	57.90	Clostridiales	8750	1987804
Sample_SCA67.mb.112cbin.1	56.10	Clostridiales	109534	2536605
Sample_SCA67.Cluster3344cbin.1	54.30	Bacteroidetes	94333	2300561
Sample_SCA67.Cluster15592cbin.1	37.70	Clostridiales	6952	2334456
Sample_SCA67.mb.68cbin.1	49.30	Bacteria	11599	1850013
Sample_SCA67.mb.52cbin.1	46.40	Clostridiales	78442	1889798
Sample_SCA67.mb.124cbin.1	45.30	Prevotella	17065	3189394
Sample_SCA67.mb.21	56.10	Bacteroidetes	8089	2226230
Sample_SCA67.mb.90	52.50	Clostridiales	62630	2111722
Sample_SCA67.Cluster9603cbin.1	43.70	Lachnospiraceae	22600	2769579
Sample_SCA67.mb.11cbin.1	38.60	Streptococcus	26915	1883337
Sample_SCA67.mb.13	53.50	Clostridiales	31284	2269000
Sample_SCA67.mb.32	43.00	Clostridiales	7672	2116838
Sample_SCA67.mb.78	57.10	Clostridiales	30750	2426355
Sample_SCA67.mb.42cbin.1	41.70	Clostridiales	132890	2453547
Sample_SCA67.mb.63	48.20	Bacteroidales	23882	2679156
Sample_SCA67.mb.88	62.20	Clostridiales	81393	2012059
Sample_SCA67.mb.1	43.30	Bacteroidales	172551	3161982
Sample_SCA67.mb.96	45.00	Clostridiales	102694	2461233
Sample_SCA67.mb.41	41.70	Lachnospiraceae	70959	2632662
Sample_SCA67.mb.6cbin.1	43.00	Clostridiales	242039	2783838
Sample_SCA67.mb.91	38.80	Clostridiales	72450	2051872
Sample_SCA67.mb.97	44.60	Clostridiales	124712	2218220
Sample_SCA67.mb.92cbin.1	60.70	Bacteroidetes	13203	2575480
Sample_SCA67.mb.81	59.40	Clostridiales	76693	3391301
Sample_SCA68.Cluster11483	26.50	Bacteria	115859	1306973
Sample_SCA68.Cluster11308	29.40	Bacteria	56400	1449726
Sample_SCA68.Cluster1343cbin.1	53.50	Clostridiales	53340	1819356
Sample_SCA68.Cluster215cbin.1	59.60	Clostridiales	79211	2201846
Sample_SCA68.Cluster53	61.80	Clostridiales	74005	1514659
Sample_SCA68.mb.76	26.80	Bacteria	26642	1196872
Sample_SCA68.mb.69	49.60	Clostridiales	118471	2065927
Sample_SCA68.mb.34cbin.1	47.40	Lactobacillales	5520	1321831
Sample_SCA68.mb.74cbin.1	53.20	Clostridia	43978	1945729
Sample_SCA68.mb.17	59.80	Clostridiales	65574	2144320
Sample_SCA68.mb.31	58.00	Clostridiales	46161	2062357
Sample_SCA68.mb.39	54.40	Clostridiales	36421	2060664
Sample_SCA68.mb.49	58.30	Clostridiales	70825	2151807
Sample_SCA68.Cluster224cbin.1	59.50	Clostridiales	124943	2365505

Sample_SCA68.mb.27	52.10	Clostridiales	23683	2430819
Sample_SCA68.Cluster615cbin.1	55.90	Clostridiales	48253	3393815
Sample_SCA68.mb.62	61.00	Bacteria	55825	4614005
Sample_SCA68.Cluster1382cbin.1	53.40	Clostridiales	176255	1956176
Sample_SCA68.mb.30cbin.1	51.00	Firmicutes	37272	2238454
Sample_SCA68.mb.45	48.00	Clostridiales	15953	1819048
Sample_SCA68.Cluster2827cbin.1	51.70	Clostridiales	133374	2234202
Sample_SCA68.Cluster10198	38.10	Lactobacillus	113554	1849765
Sample_SCA68.mb.79	61.10	Clostridiales	82633	2036093
Sample_SCA68.mb.64cbin.1	59.90	Bacteroidetes	51890	2616204
Sample_SCA68.mb.38	37.30	Clostridiales	75793	2737066
Sample_SCA68.Cluster8245	41.40	Lachnospiraceae	90472	2888966
Sample_SCA68.mb.6	58.70	Bacteroidetes	71521	3059692
Sample_SCA68.mb.65cbin.1	38.20	Clostridiales	81153	2543328
Sample_SCA68.mb.81	44.40	Clostridiales	142843	2275996
Sample_SCA68.Cluster5605cbin.1	46.40	Bacteroidales	136755	4453274
Sample_SCA68.mb.72cbin.1	41.80	Bacteroidales	36833	5065285
Sample_SCA68.mb.51cbin.1	51.00	Enterobacteriaceae	25132	4354148
Sample_SCA69.Cluster5198cbin.1	49.60	Clostridiales	117748	1979438
Sample_SCA69.Cluster249cbin.1	59.00	Clostridiales	115998	2207553
Sample_SCA69.Cluster361cbin.1	58.00	Clostridiales	6565	1597231
Sample_SCA69.mb.20	58.30	Clostridiales	30138	2972581
Sample_SCA69.Cluster65cbin.1	62.30	Actinobacteria	157814	2284433
Sample_SCA69.Cluster3856	52.30	Clostridiales	151556	2318349
Sample_SCA69.Cluster1774cbin.1	58.20	Clostridiales	30684	2778700
Sample_SCA69.Cluster851	56.20	Bacteroidetes	5079	2002486
Sample_SCA69.Cluster974cbin.1	60.00	Actinobacteria	30447	2202258
Sample_SCA69.mb.34	41.40	Clostridiales	24922	2968125
Sample_SCA69.Cluster6cbin.1	64.30	Deltaproteobacteria	8389	2179547
Sample_SCA69.Cluster12637	30.90	Euryarchaeota	106061	1788490
Sample_SCA69.Cluster2119	56.50	Selenomonadales	74679	2058580
Sample_SCA69.Cluster12106	32.80	Lactobacillales	7968	1616494
Sample_SCA69.Cluster1131cbin.1	58.40	Bifidobacteriaceae	155728	2436576
Sample_SCA69.mb.40cbin.1	62.10	Clostridiales	40695	1955481
Sample_SCA69.mb.26cbin.1	56.00	Clostridiales	84571	2617104
Sample_SCA69.Cluster171cbin.1	58.90	Clostridiales	13354	3017823
Sample_SCA69.Cluster347	59.50	Clostridiales	51976	2029345
Sample_SCA69.Cluster8586cbin.1	44.50	Clostridiales	158480	2616692
Sample_SCA69.mb.42cbin.1	60.30	Clostridiales	76641	2519145
Sample_SCA69.mb.24	51.60	Clostridiales	124023	2107515
Sample_SCA69.mb.21cbin.1	59.20	Bifidobacteriaceae	101672	2291651
Sample_SCA69.mb.22	54.60	Bacteroidetes	74130	2315655
Sample_SCA69.Cluster8764	41.70	Lachnospiraceae	24432	2635590
Sample_SCA69.Cluster6685cbin.1	44.70	Prevotella	65741	3565019
Sample_SCA69.mb.54	59.40	Clostridiales	86079	2071164
Sample_SCA69.mb.31	51.20	Firmicutes	35168	2217023
Sample_SCA69.mb.78	52.80	Clostridia	64869	2108323
Sample_SCA69.mb.100cbin.1	42.00	Lachnospiraceae	10840	2213885
Sample_SCA69.mb.46	63.00	Bifidobacteriaceae	41121	1953239
Sample_SCA69.mb.64cbin.1	61.30	Bacteria	21410	4483441
Sample_SCA69.mb.73	59.70	Clostridiales	67764	2213865
Sample_SCA69.mb.66	56.80	Clostridiales	5207	2080545
Sample_SCA69.mb.55cbin.1	51.40	Lactobacillales	7295	1527073
Sample_SCA69.Cluster7739cbin.1	43.30	Bacteria	73228	4149476
Sample_SCA69.Cluster7332	45.20	Bacteroidales	57757	4369857
Sample_SCA69.Cluster7186cbin.1	46.40	Bacteroidales	112543	4218592
Sample_SCA69.mb.52	41.60	Clostridiales	81020	3520020
Sample_SCA69.mb.75cbin.1	60.10	Bifidobacteriaceae	33970	2295609

Sample_SCA69.mb.86	60.30	Bacteroidetes	31075	2548679
Sample_SCA69.mb.87	58.50	Bacteria	38161	2856199
Sample_SCA69.Cluster1375cbin.1	50.30	Enterobacteriaceae	40769	4778128
Sample_SCA69.mb.72	43.50	Bacteroidales	121224	2750715
Sample_SCA69.mb.76	42.10	Bacteroidales	96658	4244974
Sample_SCA69.Cluster810	57.40	Enterobacteriaceae	102882	4920422
Sample_SCA70.Cluster4533cbin.1	53.20	Clostridia	39386	1891670
Sample_SCA70.Cluster1750cbin.1	59.40	Clostridiales	18720	1771899
Sample_SCA70.Cluster362cbin.1	62.00	Clostridiales	29434	1677199
Sample_SCA70.mb.109cbin.1	55.40	Clostridia	11875	2319737
Sample_SCA70.Cluster572cbin.1	56.00	Clostridiales	32983	2672085
Sample_SCA70.Cluster4600cbin.1	51.80	Clostridiales	105697	2892041
Sample_SCA70.Cluster10417cbin.1	44.70	Lachnospiraceae	105701	2871529
Sample_SCA70.Cluster4264cbin.1	49.90	Bacteroidales	17434	2473942
Sample_SCA70.mb.14	49.60	Clostridiales	31959	1851677
Sample_SCA70.mb.132cbin.1	36.40	Clostridiales	142616	1900756
Sample_SCA70.mb.103cbin.1	54.00	Clostridiales	28009	1961826
Sample_SCA70.Cluster4846	53.30	Clostridiales	139876	2096464
Sample_SCA70.Cluster7775	47.60	Selenomonadales	69133	2120249
Sample_SCA70.Cluster8429cbin.1	47.20	Clostridiales	56403	2588782
Sample_SCA70.Cluster5559cbin.1	49.10	Bacteroidales	159374	3067500
Sample_SCA70.mb.112	51.50	Bacteroidetes	49071	3099468
Sample_SCA70.mb.116	59.30	Clostridiales	31905	1794964
Sample_SCA70.Cluster2459cbin.1	52.80	Bacteroidales	63013	2382548
Sample_SCA70.Cluster11952cbin.1	41.50	Lachnospiraceae	34237	2699953
Sample_SCA70.Cluster10895cbin.1	45.20	Prevotella	36797	3540701
Sample_SCA70.mb.37cbin.1	57.10	Clostridiales	42758	2442009
Sample_SCA70.Cluster4626cbin.1	49.50	Bacteroidales	20047	2801022
Sample_SCA70.mb.34	60.00	Clostridiales	10820	2142531
Sample_SCA70.mb.115	63.10	Proteobacteria	7622	1866664
Sample_SCA70.mb.43cbin.1	56.80	Clostridiales	37982	2289568
Sample_SCA70.mb.87cbin.1	57.30	Clostridiales	17085	2980560
Sample_SCA70.mb.28	44.10	Lachnospiraceae	59172	2966972
Sample_SCA70.Cluster8153cbin.1	43.10	Bacteria	5660	3309193
Sample_SCA70.mb.47	44.70	Rhodospirillales	17537	2013282
Sample_SCA70.mb.18	41.00	Clostridiales	77929	2553678
Sample_SCA70.mb.36cbin.1	48.20	Bacteroidales	11474	3705654
Sample_SCA70.mb.40cbin.1	56.10	Clostridiales	10985	2472499
Sample_SCA70.mb.70cbin.1	46.80	Lachnospiraceae	53735	3254323
Sample_SCA70.mb.82cbin.1	31.30	Euryarchaeota	73006	1713250
Sample_SCA70.mb.69cbin.1	46.10	Prevotella	37943	2867121
Sample_SCA70.mb.83cbin.1	41.80	Clostridiales	129917	2434730
Sample_SCA70.mb.90	38.80	Clostridiales	81259	2049010
Sample_SCA70.mb.84cbin.1	44.50	Clostridiales	42828	2602063
Sample_SCA70.mb.7cbin.1	36.80	Clostridiales	90041	2901999
Sample_SCA70.mb.86cbin.1	50.10	Bacteroidales	56867	2431410
Sample_SCA70.Cluster527cbin.1	50.80	Enterobacteriaceae	19745	4406554
Sample_SCA70.mb.65cbin.1	42.30	Bacteroidales	33487	4866370
Sample_SCB55.Cluster15779	29.50	Bacteria	87829	1505158
Sample_SCB55.Cluster6784cbin.1	46.10	Clostridia	44705	1754948
Sample_SCB55.mb.132cbin.1	49.20	Bacteria	119649	1915279
Sample_SCB55.Cluster5577cbin.1	49.60	Clostridiales	11566	1763647
Sample_SCB55.mb.26	63.00	Bacteria	8031	1809583
Sample_SCB55.Cluster142	62.10	Clostridiales	10078	1404160
Sample_SCB55.Cluster15984	28.00	Euryarchaeota	126133	1775851
Sample_SCB55.mb.128	32.10	Bacteria	371778	1929374
Sample_SCB55.mb.123	36.40	Clostridiales	181940	1874662
Sample_SCB55.Cluster15282	33.60	Bacteria	18524	2004203

Sample_SCB55.Cluster5831	45.00	Clostridiales	118229	2487528
Sample_SCB55.mb.2	52.00	Clostridiales	17209	2028199
Sample_SCB55.mb.111	52.90	Clostridiales	39227	2143503
Sample_SCB55.mb.62	47.70	Clostridiales	307064	1469264
Sample_SCB55.mb.20	60.80	Clostridiales	55819	1821826
Sample_SCB55.Cluster1195	58.60	Veillonellaceae	376481	2350824
Sample_SCB55.Cluster13242	36.70	Gammaproteobacteria	80460	2244451
Sample_SCB55.mb.39	61.10	Clostridiales	59782	1903007
Sample_SCB55.Cluster9473cbin.1	44.50	Clostridiales	84458	2195553
Sample_SCB55.Cluster13869cbin.1	37.40	Clostridiales	85367	2587601
Sample_SCB55.mb.57	46.60	Clostridiales	75279	1885225
Sample_SCB55.mb.49cbin.1	51.50	Bacteroidetes	100714	3202315
Sample_SCB55.mb.52cbin.1	47.70	Lachnospiraceae	12077	2303235
Sample_SCB55.mb.25	53.90	Clostridiales	163647	1869565
Sample_SCB55.mb.96	58.40	Clostridiales	14334	2882431
Sample_SCB55.mb.1cbin.1	60.30	Bifidobacteriaceae	21779	1881671
Sample_SCB55.mb.107cbin.1	50.00	Lachnospiraceae	26253	2753285
Sample_SCB55.mb.58	43.70	Lachnospiraceae	25100	2715955
Sample_SCB55.mb.71	46.90	Clostridiales	10306	2073510
Sample_SCB55.mb.7	36.10	Clostridiales	157699	2376331
Sample_SCB55.mb.94cbin.1	38.80	Clostridiales	68421	2065479
Sample_SCB55.mb.97	44.10	Clostridiales	91069	2765225
Sample_SCB55.mb.83	43.40	Bacteroidales	33178	3022061
Sample_SCB55.mb.61cbin.1	40.70	Lachnospiraceae	55102	4901640
Sample_SCB55.mb.17cbin.1	55.90	Enterobacteriaceae	217857	5413308
Sample_SCB58.Cluster2238cbin.1	53.20	Clostridia	8201	1720881
Sample_SCB58.Cluster251cbin.1	59.60	Clostridiales	126901	2068199
Sample_SCB58.Cluster7841	48.70	Clostridia	372071	1490335
Sample_SCB58.Cluster17584cbin.1	59.30	Clostridiales	54614	2102354
Sample_SCB58.Cluster323cbin.1	56.40	Clostridiales	9214	1987319
Sample_SCB58.mb.102	41.20	Bacteria	17417	3390979
Sample_SCB58.Cluster16941cbin.1	34.10	Bacilli	39590	2721059
Sample_SCB58.Cluster4731	54.20	Actinobacteria	152194	1703612
Sample_SCB58.Cluster533	59.50	Clostridiales	47572	1861117
Sample_SCB58.Cluster14393cbin.1	42.00	Clostridiales	7235	2019148
Sample_SCB58.mb.100	58.40	Clostridia	14296	2613523
Sample_SCB58.Cluster14600cbin.1	40.90	Clostridiales	185266	2073635
Sample_SCB58.mb.21	26.60	Bacteria	67193	1224822
Sample_SCB58.Cluster11721cbin.1	44.50	Clostridiales	144704	2251802
Sample_SCB58.Cluster5432cbin.1	48.50	Firmicutes	35133	1768427
Sample_SCB58.mb.10	61.90	Clostridiales	71047	1695474
Sample_SCB58.Cluster1280	55.70	Bacteria	175223	2786268
Sample_SCB58.mb.103cbin.1	43.90	Clostridiales	19167	2740213
Sample_SCB58.mb.135	60.70	Actinobacteria	59577	1785303
Sample_SCB58.mb.126cbin.1	57.60	Clostridiales	51794	3272749
Sample_SCB58.Cluster3866cbin.1	59.40	Bacteroidetes	148322	2954661
Sample_SCB58.mb.43	26.20	Bacteria	72446	1163235
Sample_SCB58.mb.15	48.60	Clostridiales	73451	1768652
Sample_SCB58.Cluster6185	51.40	Clostridiales	161524	2166524
Sample_SCB58.Cluster11768	42.50	Lachnospiraceae	67600	2210435
Sample_SCB58.Cluster725	60.20	Bifidobacteriaceae	145651	2240692
Sample_SCB58.mb.132cbin.1	49.00	Clostridiales	13139	2455010
Sample_SCB58.mb.76	24.00	Bacteria	27395	895035
Sample_SCB58.mb.116	56.40	Clostridiales	12766	2370035
Sample_SCB58.mb.32cbin.1	61.40	Clostridiales	20225	1870887
Sample_SCB58.mb.59	58.80	Clostridiales	22468	2351935
Sample_SCB58.mb.9	53.70	Clostridia	41518	1689914

Sample_SCB58.mb.34cbin.1	61.90	Clostridiales	28872	2767597
Sample_SCB58.mb.39cbin.1	54.90	Bacteroidetes	174613	1968116
Sample_SCB58.mb.87cbin.1	37.00	Clostridiales	17027	2795949
Sample_SCB58.mb.31	59.90	Bacteroidetes	104783	2652747
Sample_SCB58.mb.35	44.00	Clostridiales	46533	2800178
Sample_SCB58.Cluster11034cbin.1	41.60	Bacteroidales	55582	4556945
Sample_SCB58.mb.7	56.90	Clostridiales	63827	2671458
Sample_SCB58.Cluster9840cbin.1	42.80	Bacteria	80243	4162187
Sample_SCB58.mb.49	57.60	Proteobacteria	57973	2172433
Sample_SCB58.mb.20	58.80	Bacteroidetes	164032	3093332
Sample_SCB58.mb.66	60.30	Clostridiales	29926	2444506
Sample_SCB58.mb.46	36.10	Clostridiales	23329	2419689
Sample_SCB58.mb.48	43.80	Clostridiales	80777	2768895
Sample_SCB58.Cluster6891cbin.1	42.60	Bacteria	14750	4907150
Sample_SCB58.Cluster9211	45.00	Bacteroidales	226527	4622203
Sample_SCB58.mb.111	45.40	Bacteroidales	110106	3921790
Sample_SCB58.mb.96cbin.1	64.00	Actinobacteria	9982	2439219
Sample_SCB58.mb.97cbin.1	43.30	Bacteroidales	158408	3098056
Sample_SCB58.mb.60	56.10	Enterobacteriaceae	56290	4767150
Sample_SCB59.Cluster161cbin.1	61.80	Clostridiales	14980	1725468
Sample_SCB59.Cluster480	58.70	Clostridiales	120101	2361164
Sample_SCB59.Cluster11926cbin.1	42.80	Clostridiales	151700	2003082
Sample_SCB59.mb.142	37.30	Clostridiales	28103	2475223
Sample_SCB59.mb.28	53.20	Clostridiales	7854	1631932
Sample_SCB59.Cluster12411	40.60	Clostridiales	132221	2134640
Sample_SCB59.mb.135cbin.1	27.40	Euryarchaeota	20407	1792066
Sample_SCB59.mb.124cbin.1	56.70	Clostridiales	22272	2238027
Sample_SCB59.mb.43cbin.1	49.20	Clostridiales	76297	1981785
Sample_SCB59.Cluster4224cbin.1	51.90	Clostridiales	132719	2346457
Sample_SCB59.mb.49	59.40	Clostridia	48765	2518553
Sample_SCB59.mb.17cbin.1	45.40	Actinobacteria	216632	1671109
Sample_SCB59.mb.25	43.90	Clostridiales	30373	2662972
Sample_SCB59.mb.107	58.40	Bacteroidetes	368914	2565264
Sample_SCB59.mb.16cbin.1	59.20	Clostridiales	10317	2197458
Sample_SCB59.mb.81	27.90	Bacteria	66775	928581
Sample_SCB59.mb.127	63.10	Actinobacteria	12303	2604673
Sample_SCB59.mb.109	44.40	Clostridiales	54297	2486112
Sample_SCB59.mb.118	31.10	Euryarchaeota	112722	1771141
Sample_SCB59.mb.4cbin.1	44.20	Clostridiales	48187	2361674
Sample_SCB59.mb.113cbin.1	58.30	Bacteria	26819	2999649
Sample_SCB59.mb.51	59.30	Clostridiales	21693	1709469
Sample_SCB59.Cluster32cbin.1	62.10	Deltaproteobacteria	11803	3094583
Sample_SCB59.mb.101cbin.1	58.70	Bifidobacteriaceae	35230	2321912
Sample_SCB59.mb.18	54.90	Bacteroidetes	56768	2083377
Sample_SCB59.mb.133cbin.1	39.70	Streptococcus	7271	1877785
Sample_SCB59.mb.94cbin.1	59.50	Actinobacteria	30930	1688736
Sample_SCB59.mb.63cbin.1	56.50	Clostridiales	29212	2551845
Sample_SCB59.mb.62cbin.1	51.90	Clostridiales	37558	2057018
Sample_SCB59.mb.44	45.40	Clostridiales	25749	2398529
Sample_SCB59.Cluster9671cbin.1	43.10	Bacteria	83134	4134808
Sample_SCB59.mb.40	43.70	Lachnospiraceae	110437	2764875
Sample_SCB59.Cluster7227	45.30	Bacteroidales	150367	4054771
Sample_SCB59.mb.73cbin.1	48.80	Firmicutes	32914	1860064
Sample_SCB59.mb.141cbin.1	47.20	Bacteroidales	18016	3735468
Sample_SCB59.mb.97	54.40	Bacteroidetes	524173	2611020
Sample_SCB59.mb.68	43.90	Bacteria	80518	4202131
Sample_SCB59.mb.9cbin.1	44.70	Bacteroidales	30559	4797597
Sample_SCB60.Cluster14735cbin.1	29.40	Bacteria	69222	1685131

Sample_SCB60.Cluster14408	32.20	Bacteria	78942	1885127
Sample_SCB60.Cluster2146cbin.1	38.80	Clostridiales	70111	2764910
Sample_SCB60.mb.15	24.20	Bacteria	61076	1018581
Sample_SCB60.mb.102cbin.1	32.80	Bacteria	95309	1913970
Sample_SCB60.mb.10	43.10	Clostridiales	53674	3091811
Sample_SCB60.Cluster242	59.80	Actinobacteria	111669	2177806
Sample_SCB60.Cluster11239	41.10	Clostridiales	118676	1747562
Sample_SCB60.mb.135	53.00	Clostridiales	45965	2341899
Sample_SCB60.Cluster3500cbin.1	56.50	Clostridiales	15282	2346691
Sample_SCB60.Cluster10627cbin.1	38.80	Clostridiales	8904	2408872
Sample_SCB60.Cluster1947	52.20	Firmicutes	59709	2387579
Sample_SCB60.Cluster10659	39.50	Clostridiales	98964	2136611
Sample_SCB60.Cluster350cbin.1	57.80	Proteobacteria	8968	2007223
Sample_SCB60.Cluster568cbin.1	57.30	Clostridiales	38427	3395923
Sample_SCB60.Cluster2276	55.60	Bacteria	183414	2681987
Sample_SCB60.mb.30cbin.1	58.50	Clostridiales	47030	2633568
Sample_SCB60.mb.17	55.60	Clostridiales	53313	2331968
Sample_SCB60.mb.100cbin.1	51.80	Clostridiales	11875	2020952
Sample_SCB60.mb.76	26.00	Bacteria	63995	1222152
Sample_SCB60.mb.103cbin.1	45.20	Clostridiales	76414	2103164
Sample_SCB60.mb.18cbin.1	46.90	Clostridiales	58329	2808597
Sample_SCB60.mb.36	59.30	Bacteria	64836	4153191
Sample_SCB60.mb.129	42.20	Clostridiales	40891	2491768
Sample_SCB60.mb.111cbin.1	59.40	Bacteroidetes	41903	3028579
Sample_SCB60.mb.24	36.50	Clostridiales	390413	1719074
Sample_SCB60.mb.57	62.10	Clostridiales	36327	2065863
Sample_SCB60.mb.72	61.80	Clostridiales	12248	1985026
Sample_SCB60.mb.67	49.40	Clostridiales	71320	1803129
Sample_SCB60.mb.32	54.30	Bacteroidetes	87975	2087466
Sample_SCB60.Cluster8880	43.40	Bacteroidales	152065	2827593
Sample_SCB60.mb.75	40.70	Clostridiales	17979	3763005
Sample_SCB60.mb.49cbin.1	41.80	Clostridiales	168642	2553289
Sample_SCB60.Cluster9407cbin.1	41.60	Clostridiales	14799	3590406
Sample_SCB60.mb.12cbin.1	43.40	Bacteria	42459	3956494
Sample_SCB60.mb.82	60.30	Bacteroidetes	19587	2419098
Sample_SCB60.mb.74cbin.1	40.50	Clostridiales	67526	2747245
Sample_SCB60.mb.81	49.50	Lachnospiraceae	97107	2799774
Sample_SCB60.Cluster1292cbin.1	57.20	Enterobacteriaceae	161665	4716094
Sample_SCB61.Cluster2650	47.40	Bacteria	42543	1690584
Sample_SCB61.mb.103	42.00	Bacteria	104871	2414395
Sample_SCB61.Cluster8139	33.40	Clostridiales	271842	2268691
Sample_SCB61.mb.67cbin.1	26.90	Bacteria	22443	2868720
Sample_SCB61.mb.27	38.50	Clostridiales	82991	2318227
Sample_SCB61.mb.34	58.60	Clostridiales	8426	2614031
Sample_SCB61.mb.40cbin.1	43.50	Clostridiales	11088	2843321
Sample_SCB61.mb.47	37.30	Clostridiales	28361	2489146
Sample_SCB61.Cluster1101cbin.1	54.20	Bacteroidetes	147326	1902956
Sample_SCB61.mb.114cbin.1	47.70	Clostridiales	103011	3034906
Sample_SCB61.Cluster6209	38.10	Clostridiales	94503	1865364
Sample_SCB61.Cluster5839	40.90	Clostridiales	177499	2016656
Sample_SCB61.Cluster112	55.40	Bacteria	190909	2677255
Sample_SCB61.mb.45	49.40	Clostridiales	16971	2293015
Sample_SCB61.mb.82	42.50	Clostridiales	58612	2456162
Sample_SCB61.Cluster523	57.30	Proteobacteria	109781	2350002
Sample_SCB61.mb.50cbin.1	46.40	Lachnospiraceae	77649	2652589
Sample_SCB61.Cluster4479	44.00	Clostridiales	199673	2752563
Sample_SCB61.mb.79	57.00	Clostridiales	8701	2284119
Sample_SCB61.mb.109cbin.1	44.10	Clostridiales	42245	3362797

Sample_SCB61.mb.44cbin.1	57.60	Clostridiales	41503	2315915
Sample_SCB61.mb.31cbin.1	40.70	Lachnospiraceae	86015	3065846
Sample_SCB61.mb.76cbin.1	57.30	Clostridiales	21480	2299866
Sample_SCB61.mb.88	60.10	Bacteroidetes	184030	2439045
Sample_SCB61.mb.3	43.60	Bacteria	7467	3244892
Sample_SCB61.mb.83	43.20	Lachnospiraceae	46241	2116464
Sample_SCB61.Cluster4578cbin.1	40.50	Clostridiales	33642	3908932
Sample_SCB61.mb.91cbin.1	59.80	Bacteroidetes	154605	2727629
Sample_SCB61.Cluster3478cbin.1	46.60	Bacteroidales	183164	4085554
Sample_SCB61.mb.94	37.50	Clostridiales	140595	2537953
Sample_SCB61.mb.99cbin.1	45.90	Bacteroidales	45942	3668752
Sample_SCB61.mb.90	45.00	Bacteroidales	197824	4465695
Sample_SCB61.mb.28cbin.1	50.50	Enterobacteriaceae	92614	4844286
Sample_SCB64.Cluster3052	49.30	Clostridiales	78069	1795394
Sample_SCB64.mb.29cbin.1	29.90	Bacteria	48711	2580270
Sample_SCB64.Cluster4934cbin.1	51.30	Clostridiales	11740	2200837
Sample_SCB64.Cluster7568	32.00	Clostridiales	103848	2825650
Sample_SCB64.Cluster4325	45.00	Clostridiales	102146	2588341
Sample_SCB64.Cluster7301cbin.1	42.10	Lachnospiraceae	15412	2014818
Sample_SCB64.mb.4	38.50	Clostridiales	10876	2065406
Sample_SCB64.Cluster5490cbin.1	41.60	Clostridiales	16517	2323362
Sample_SCB64.Cluster3178cbin.1	51.90	Firmicutes	41189	2525576
Sample_SCB64.Cluster328cbin.1	59.40	Bacteroidetes	170144	2578286
Sample_SCB64.Cluster1273	55.50	Proteobacteria	161474	2280828
Sample_SCB64.mb.31	57.50	Clostridiales	15686	1915006
Sample_SCB64.mb.124cbin.1	61.10	Clostridiales	38113	2682512
Sample_SCB64.Cluster4422cbin.1	46.20	Lachnospiraceae	68424	3530733
Sample_SCB64.mb.16	60.10	Actinobacteria	14861	1979535
Sample_SCB64.Cluster345	58.10	Bacteria	158222	3114389
Sample_SCB64.Cluster7227	40.50	Clostridiales	132547	2133843
Sample_SCB64.mb.54cbin.1	61.80	Clostridiales	25245	1705601
Sample_SCB64.mb.108cbin.1	59.90	Bifidobacteriaceae	89780	1896888
Sample_SCB64.mb.105cbin.1	56.70	Clostridiales	108547	2131009
Sample_SCB64.mb.34	56.30	Clostridiales	16734	2525343
Sample_SCB64.Cluster9343cbin.1	36.00	Clostridiales	133721	2600357
Sample_SCB64.mb.18cbin.1	56.30	Bifidobacteriaceae	127847	2170782
Sample_SCB64.mb.67	56.10	Bacteroidales	7788	2049782
Sample_SCB64.mb.55	54.50	Bacteroidetes	86542	2103666
Sample_SCB64.Cluster4386cbin.1	44.30	Bacteroidales	68498	3633190
Sample_SCB64.mb.28	60.30	Deltaproteobacteria	11035	3744551
Sample_SCB64.Cluster4283cbin.1	45.00	Bacteroidales	204278	4573376
Sample_SCB64.mb.8cbin.1	60.60	Bifidobacteriaceae	14799	1866873
Sample_SCB64.mb.57cbin.1	40.60	Bacteroidales	51255	3242110
Sample_SCB64.mb.92	44.10	Clostridiales	167716	2588658
Sample_SCB64.mb.89	57.20	Clostridiales	25310	2106242
Sample_SCB64.mb.69cbin.1	49.60	Lachnospiraceae	36030	2946435
Sample_SCB64.mb.95cbin.1	49.50	Proteobacteria	7699	1829030
Sample_SCB64.mb.122	43.10	Bacteroidales	127987	4959259
Sample_SCB64.mb.46cbin.1	45.40	Bacteroidales	77153	4076781
Sample_SCB64.mb.58cbin.1	47.40	Bacteroidales	38462	3744213
Sample_SCB64.mb.72	48.50	Bacteroidales	116733	3283109
Sample_SCB64.mb.81cbin.1	40.40	Lachnospiraceae	41859	4107393
Sample_SCB65.Cluster2978	49.40	Bacteria	62766	1774247
Sample_SCB65.mb.19	52.70	Bacteria	13953	1971828
Sample_SCB65.mb.43cbin.1	55.70	Clostridiales	7739	1994541
Sample_SCB65.mb.57	37.40	Clostridiales	6338	2040287
Sample_SCB65.mb.27cbin.1	54.30	Actinobacteria	30297	1601826
Sample_SCB65.Cluster3240	48.40	Clostridiales	107829	2772441

Sample_SCB65.Cluster2325cbin.1	44.20	Selenomonadales	7545	1913930
Sample_SCB65.mb.34	59.30	Bacteroidetes	6098	2037522
Sample_SCB65.mb.24cbin.1	50.60	Clostridiales	127656	2901638
Sample_SCB65.Cluster6701	39.00	Clostridiales	114593	1988870
Sample_SCB65.Cluster7656cbin.1	52.80	Clostridiales	214452	2077775
Sample_SCB65.Cluster123	55.40	Bacteria	150670	2826645
Sample_SCB65.mb.70cbin.1	59.80	Clostridiales	27820	2330437
Sample_SCB65.mb.54	42.90	Clostridiales	11315	2209417
Sample_SCB65.Cluster4782cbin.1	44.10	Clostridiales	114308	2740622
Sample_SCB65.Cluster98cbin.1	58.90	Bacteroidetes	95035	3032603
Sample_SCB65.mb.10	40.40	Clostridiales	143413	2578516
Sample_SCB65.mb.3	39.90	Streptococcus	19136	1994617
Sample_SCB65.mb.66	63.20	Actinobacteria	137464	2911208
Sample_SCB65.mb.79	61.30	Clostridiales	41097	2583912
Sample_SCB65.mb.84	59.20	Bacteroidetes	32590	2074089
Sample_SCB65.Cluster4891cbin.1	42.60	Lachnospiraceae	112033	3400663
Sample_SCB65.mb.68cbin.1	37.30	Clostridiales	96905	2565487
Sample_SCB65.mb.72cbin.1	58.30	Clostridiales	15442	2432271
Sample_SCB65.mb.9cbin.1	42.70	Clostridiales	73648	2474791
Sample_SCB65.Cluster4119cbin.1	42.30	Bacteria	10545	4029215
Sample_SCB65.mb.77cbin.1	48.40	Proteobacteria	36568	2551652
Sample_SCB65.mb.28cbin.1	41.60	Bacteroidales	44256	4725154
Sample_SCB65.mb.20	45.00	Bacteroidales	155251	4753409
Sample_SCB65.mb.37	41.90	Bacteroides	57522	4672102
Sample_SCB65.mb.4cbin.1	44.60	Bacteroidales	10224	5141288
Sample_SCB65.mb.74cbin.1	46.70	Bacteroidales	146744	4361538
Sample_SCB65.mb.65cbin.1	43.40	Bacteroidales	174079	5922940
Sample_SCB66.Cluster9757cbin.1	26.70	Bacteria	21815	727755
Sample_SCB66.Cluster3087	49.40	Clostridiales	96473	1985279
Sample_SCB66.Cluster9832cbin.1	26.00	Bacteria	81515	1358930
Sample_SCB66.mb.113	27.50	Bacteria	293700	1315577
Sample_SCB66.Cluster314cbin.1	58.70	Clostridiales	50980	2111596
Sample_SCB66.Cluster326cbin.1	59.20	Clostridiales	51916	2274283
Sample_SCB66.Cluster6cbin.1	62.50	Clostridiales	6996	2061457
Sample_SCB66.Cluster2065	52.40	Clostridiales	125431	2323295
Sample_SCB66.Cluster3667cbin.1	46.70	Lactobacillales	87802	2025680
Sample_SCB66.Cluster3687cbin.1	45.20	Firmicutes	181692	1686721
Sample_SCB66.Cluster2786cbin.1	43.90	Selenomonadales	10837	2132600
Sample_SCB66.Cluster7029	40.70	Clostridiales	185054	2079885
Sample_SCB66.Cluster332	55.60	Bacteria	204989	2715287
Sample_SCB66.Cluster1226	56.60	Bifidobacteriaceae	116818	1938995
Sample_SCB66.Cluster7847cbin.1	44.00	Clostridiales	166641	2077308
Sample_SCB66.mb.23cbin.1	48.30	Clostridiales	32328	2690658
Sample_SCB66.mb.36cbin.1	54.20	Actinobacteria	77059	1612860
Sample_SCB66.mb.65	52.90	Clostridia	44595	2111398
Sample_SCB66.mb.5cbin.1	58.20	Clostridiales	66714	2908856
Sample_SCB66.Cluster6263	32.60	Lactobacillales	51012	1915906
Sample_SCB66.Cluster5296cbin.1	41.80	Lachnospiraceae	52029	2455706
Sample_SCB66.Cluster5056	43.10	Clostridiales	137587	2612299
Sample_SCB66.Cluster1256cbin.1	59.50	Bacteroidetes	14775	2778601
Sample_SCB66.mb.107cbin.1	40.00	Streptococcus	23364	1892347
Sample_SCB66.mb.84	36.20	Clostridiales	196270	2006701
Sample_SCB66.Cluster9252cbin.1	26.70	Clostridiales	54283	2767135
Sample_SCB66.Cluster1136cbin.1	55.10	Proteobacteria	114877	2870622
Sample_SCB66.Cluster3927cbin.1	43.30	Bacteria	10955	3519182
Sample_SCB66.mb.59	54.80	Bacteroidetes	123345	2039893
Sample_SCB66.mb.51cbin.1	55.80	Clostridiales	12678	2517210
Sample_SCB66.mb.85	60.50	Clostridiales	20964	1897975

Sample_SCB66.Cluster5699	43.70	Bacteroidales	336434	3301060
Sample_SCB66.mb.95cbin.1	34.20	Bacilli	6879	2396772
Sample_SCB66.mb.40	59.50	Bacteroidetes	142711	2813964
Sample_SCB66.mb.80	47.10	Lachnospiraceae	26305	3032742
Sample_SCB66.mb.89cbin.1	57.80	Clostridiales	53004	2913139
Sample_SCB66.Cluster4043	45.20	Bacteroidales	102465	4302111
Sample_SCB66.mb.96	60.40	Bifidobacteriaceae	38591	1995155
Sample_SCB66.mb.62	44.70	Clostridiales	47720	2858068
Sample_SCB66.mb.87	44.30	Clostridiales	48881	2683804
Sample_SCB66.mb.76	58.80	Bifidobacteriaceae	103175	2396225
Sample_SCB66.mb.30	41.90	Bacteroides	64429	4439145
Sample_SCB67.Cluster3713cbin.1	53.00	Clostridia	30422	2072728
Sample_SCB67.Cluster1428	56.90	Clostridiales	141760	2511719
Sample_SCB67.mb.101	48.90	Bacteria	53407	2002458
Sample_SCB67.Cluster12262	45.00	Clostridiales	74108	2631221
Sample_SCB67.Cluster6508	50.90	Bacteroidales	57004	2141701
Sample_SCB67.Cluster13396cbin.1	48.00	Bacteroidales	43724	2819522
Sample_SCB67.Cluster10940cbin.1	46.50	Lachnospiraceae	72884	3004302
Sample_SCB67.Cluster3518	54.70	Bacteroidetes	110548	1990801
Sample_SCB67.Cluster19cbin.1	62.90	Proteobacteria	6221	1797209
Sample_SCB67.mb.121	62.30	Clostridiales	40298	1986077
Sample_SCB67.Cluster6180cbin.1	49.40	Bacteroidales	29572	2906624
Sample_SCB67.Cluster2274cbin.1	55.30	Bacteroidetes	43170	2518321
Sample_SCB67.Cluster15509	41.60	Clostridiales	172233	2357238
Sample_SCB67.mb.24cbin.1	36.60	Clostridiales	200362	1604743
Sample_SCB67.Cluster6745cbin.1	48.50	Firmicutes	34787	2035361
Sample_SCB67.mb.123cbin.1	54.90	Clostridiales	66311	2757152
Sample_SCB67.mb.59cbin.1	25.90	Bacteria	43991	1240717
Sample_SCB67.mb.142	58.10	Clostridiales	75072	2191413
Sample_SCB67.Cluster17310cbin.1	37.40	Clostridiales	7340	2478349
Sample_SCB67.mb.26	55.00	Clostridiales	6752	2179597
Sample_SCB67.mb.61	49.60	Clostridia	13595	1778541
Sample_SCB67.mb.10	43.00	Clostridiales	228553	2641647
Sample_SCB67.Cluster153cbin.1	59.30	Clostridiales	37471	3486946
Sample_SCB67.mb.12	38.70	Streptococcus	24973	1702288
Sample_SCB67.mb.138	60.70	Bacteroidetes	28110	2542160
Sample_SCB67.mb.34	56.20	Clostridiales	98557	2387787
Sample_SCB67.mb.45	51.60	Clostridiales	23552	2119592
Sample_SCB67.mb.58cbin.1	46.70	Clostridiales	78040	1708902
Sample_SCB67.mb.85	60.70	Clostridiales	28589	1709451
Sample_SCB67.mb.113	43.30	Bacteroidales	449157	3216735
Sample_SCB67.mb.7cbin.1	45.40	Clostridiales	9463	2122020
Sample_SCB67.mb.65cbin.1	51.90	Clostridiales	11467	1945469
Sample_SCB67.Cluster7159	45.20	Bacteroidales	17348	3958878
Sample_SCB67.mb.44	41.80	Lachnospiraceae	82440	2461281
Sample_SCB67.mb.76cbin.1	52.60	Bacteroidales	72828	2670318
Sample_SCB67.mb.117	45.50	Bacteroidales	7095	3382002
Sample_SCB67.mb.64cbin.1	43.80	Lachnospiraceae	22752	2601895
Sample_SCB67.mb.80	38.80	Clostridiales	61898	2058288
Sample_SCB67.mb.95cbin.1	45.20	Clostridiales	141579	1714693
Sample_SCB67.mb.81	44.60	Clostridiales	121990	2148163
Sample_SCB67.mb.87	43.10	Clostridiales	51394	2355597
Sample_SCB68.mb.52	26.80	Bacteria	47536	980451
Sample_SCB68.Cluster1534cbin.1	53.60	Clostridiales	56827	1740931
Sample_SCB68.mb.42	53.30	Clostridia	11192	1858558
Sample_SCB68.mb.29	52.50	Clostridiales	39516	1998707
Sample_SCB68.mb.8	26.40	Bacteria	186811	1232246
Sample_SCB68.mb.62cbin.1	49.70	Clostridiales	17807	1941622

Sample_SCB68.mb.16cbin.1	56.70	Clostridiales	34734	2411844
Sample_SCB68.mb.72	58.40	Clostridiales	65200	2865049
Sample_SCB68.Cluster1151cbin.1	54.20	Clostridiales	59386	2064074
Sample_SCB68.Cluster863cbin.1	56.40	Clostridiales	80541	2086945
Sample_SCB68.Cluster1667cbin.1	57.60	Clostridiales	11853	2070877
Sample_SCB68.mb.60cbin.1	57.40	Clostridiales	63259	2319367
Sample_SCB68.mb.49	48.20	Clostridiales	5234	1471399
Sample_SCB68.Cluster2701	54.00	Clostridiales	175548	1693333
Sample_SCB68.mb.10	59.80	Bacteroidetes	106788	2531482
Sample_SCB68.Cluster8682cbin.1	38.60	Clostridiales	82488	2077969
Sample_SCB68.Cluster2582cbin.1	50.90	Firmicutes	45794	2277471
Sample_SCB68.Cluster3722cbin.1	51.50	Clostridiales	162054	2133269
Sample_SCB68.mb.77	54.30	Clostridiales	5825	2569766
Sample_SCB68.Cluster290	59.20	Bacteroidetes	88582	2693597
Sample_SCB68.mb.47	41.80	Clostridiales	206907	2462610
Sample_SCB68.Cluster8783cbin.1	38.00	Lactobacillus	100414	1872763
Sample_SCB68.mb.51	41.40	Lachnospiraceae	68093	2839751
Sample_SCB68.mb.68	44.60	Clostridiales	123523	2343118
Sample_SCB68.Cluster8615cbin.1	41.70	Lachnospiraceae	64742	2582295
Sample_SCB68.Cluster3863cbin.1	46.00	Bacteroidales	10004	2916182
Sample_SCB68.mb.2	41.80	Bacteroidales	37979	4356467
Sample_SCB68.mb.33	46.30	Bacteroidales	106534	4475512
Sample_SCB68.mb.27cbin.1	50.70	Enterobacteriaceae	38030	4596198
Sample_SCB69.Cluster6357	49.50	Clostridiales	106769	1884540
Sample_SCB69.Cluster69	62.10	Actinobacteria	132406	1930478
Sample_SCB69.mb.12	53.00	Actinobacteria	41585	1775969
Sample_SCB69.mb.110	62.50	Clostridiales	81507	1779197
Sample_SCB69.mb.123cbin.1	41.20	Clostridiales	35362	3282699
Sample_SCB69.mb.46	51.00	Clostridia	6913	1462735
Sample_SCB69.mb.127	59.40	Clostridiales	107060	2248509
Sample_SCB69.mb.32	57.50	Clostridiales	36454	1723416
Sample_SCB69.mb.108cbin.1	57.60	Clostridiales	27644	3207407
Sample_SCB69.Cluster13611	30.90	Euryarchaeota	111563	1788269
Sample_SCB69.Cluster2393	56.30	Selenomonadales	109406	2163927
Sample_SCB69.mb.10	62.00	Bacteroidetes	38012	2536551
Sample_SCB69.mb.28	60.00	Actinobacteria	36007	2222719
Sample_SCB69.Cluster5203	50.50	Lactobacillales	38383	1917719
Sample_SCB69.mb.113	62.80	Bifidobacteriaceae	125604	1878253
Sample_SCB69.Cluster12889	32.60	Lactobacillales	20888	2040497
Sample_SCB69.Cluster10744cbin.1	37.70	Clostridiales	13997	2501490
Sample_SCB69.Cluster9183	44.40	Clostridiales	157984	2613500
Sample_SCB69.mb.25cbin.1	51.50	Clostridiales	112321	2015385
Sample_SCB69.mb.111cbin.1	60.20	Bifidobacteriaceae	34933	2172803
Sample_SCB69.Cluster1157cbin.1	58.40	Bifidobacteriaceae	155720	2322957
Sample_SCB69.Cluster10340cbin.1	41.40	Lachnospiraceae	89771	2942341
Sample_SCB69.mb.116	57.90	Clostridiales	68450	2251405
Sample_SCB69.Cluster13349cbin.1	41.50	Lachnospiraceae	7091	3063496
Sample_SCB69.mb.31	58.40	Bacteria	42962	2874972
Sample_SCB69.mb.78	59.20	Clostridiales	115316	1965212
Sample_SCB69.mb.87cbin.1	52.80	Clostridia	73140	2171311
Sample_SCB69.mb.15	43.50	Bacteroidales	127338	2972416
Sample_SCB69.Cluster6631cbin.1	43.20	Bacteria	66562	4075723
Sample_SCB69.mb.122cbin.1	43.20	Lachnospiraceae	30117	2764106
Sample_SCB69.mb.66cbin.1	56.30	Clostridiales	95076	2358682
Sample_SCB69.mb.72	54.40	Bacteroidales	17897	2343910
Sample_SCB69.mb.80cbin.1	61.20	Bacteria	15422	4485206
Sample_SCB69.mb.82cbin.1	60.30	Clostridiales	64151	2463951
Sample_SCB69.mb.54	45.20	Prevotella	84823	3195423

Sample_SCB69.mb.97	59.90	Clostridiales	64834	1866130
Sample_SCB69.mb.91cbin.1	52.40	Clostridiales	148687	2388406
Sample_SCB69.mb.47	41.70	Clostridiales	79933	3357273
Sample_SCB69.mb.101	42.10	Bacteroides	46917	4482880
Sample_SCB69.mb.89cbin.1	55.60	Bacteroidetes	10693	2584516
Sample_SCB69.mb.94	56.60	Clostridiales	6975	2320634
Sample_SCB69.mb.99cbin.1	51.10	Firmicutes	38381	2215153
Sample_SCB69.mb.74	59.70	Bacteroidetes	15607	2776303
Sample_SCB69.mb.95cbin.1	60.50	Bacteroidetes	42963	2370484
Sample_SCB69.mb.30	44.90	Bacteroidales	85947	4730221
Sample_SCB69.Cluster2719cbin.1	50.70	Enterobacteriaceae	35661	4818307
Sample_SCB69.mb.96	63.90	Deltaproteobacteria	14335	2427155
Sample_SCB69.mb.41cbin.1	46.20	Bacteroidales	114547	4182690
Sample_SCB69.mb.39	41.70	Bacteroidales	106527	4644753
Sample_SCB70.Cluster16715	24.10	Bacteria	80053	886856
Sample_SCB70.Cluster16251	25.20	Bacteria	69462	1349263
Sample_SCB70.Cluster403cbin.1	54.70	Clostridia	107000	2822266
Sample_SCB70.Cluster14535	37.40	Clostridiales	120535	2066480
Sample_SCB70.mb.28	27.60	Bacteria	50846	1084725
Sample_SCB70.mb.12	53.20	Clostridia	71837	1855402
Sample_SCB70.Cluster394	61.50	Clostridiales	59694	1659837
Sample_SCB70.Cluster73cbin.1	59.10	Clostridiales	51245	1819939
Sample_SCB70.Cluster3672	52.40	Clostridiales	99660	2414875
Sample_SCB70.Cluster14944	33.50	Bacteria	17390	2130456
Sample_SCB70.Cluster16059cbin.1	31.30	Euryarchaeota	121952	1492038
Sample_SCB70.mb.114	51.50	Clostridiales	7166	1746086
Sample_SCB70.Cluster6337cbin.1	47.60	Selenomonadales	89801	2126972
Sample_SCB70.mb.54	60.20	Clostridia	9914	2437237
Sample_SCB70.Cluster8cbin.1	64.30	Deltaproteobacteria	5812	1958584
Sample_SCB70.mb.74	53.70	Clostridia	44099	1811409
Sample_SCB70.Cluster4044	52.70	Clostridiales	134393	2288492
Sample_SCB70.mb.16	37.20	Clostridiales	9880	2080064
Sample_SCB70.mb.10	56.30	Clostridiales	13926	2464203
Sample_SCB70.mb.37	61.40	Clostridiales	27360	2185938
Sample_SCB70.Cluster8507cbin.1	44.10	Clostridiales	115941	2362040
Sample_SCB70.mb.112	43.50	Lachnospiraceae	68208	3438924
Sample_SCB70.mb.17cbin.1	36.40	Clostridiales	17954	1747572
Sample_SCB70.mb.14	44.50	Lachnospiraceae	108139	2963905
Sample_SCB70.mb.58	41.20	Clostridiales	10470	3041987
Sample_SCB70.mb.27cbin.1	41.80	Clostridiales	134434	2415535
Sample_SCB70.mb.52cbin.1	56.60	Clostridiales	41551	2477008
Sample_SCB70.mb.44	60.00	Actinobacteria	17738	2038608
Sample_SCB70.mb.63	51.60	Bacteroidetes	52386	2983187
Sample_SCB70.mb.77cbin.1	57.00	Clostridiales	32427	2237398
Sample_SCB70.mb.34cbin.1	38.60	Clostridiales	115418	1983831
Sample_SCB70.mb.29	41.70	Lachnospiraceae	53304	2492919
Sample_SCB70.mb.8	57.60	Clostridiales	84622	2114625
Sample_SCB70.mb.96	59.50	Clostridiales	141206	2448011
Sample_SCB70.mb.78cbin.1	52.80	Bacteroidales	64998	2365579
Sample_SCC55.mb.2	32.10	Bacteria	371778	1929681
Sample_SCC55.Cluster5346	42.00	Clostridiales	31712	2604161
Sample_SCC55.Cluster1346cbin.1	51.80	Clostridiales	30887	2142192
Sample_SCC55.Cluster2360	46.60	Clostridiales	49492	1837420
Sample_SCC55.mb.25	53.10	Clostridiales	14609	2018315
Sample_SCC55.Cluster2032cbin.1	51.50	Bacteroidetes	100714	3385379
Sample_SCC55.Cluster37	59.90	Actinobacteria	38452	1892565
Sample_SCC55.Cluster1341	51.40	Bacteroidales	55122	2332558
Sample_SCC55.Cluster4708cbin.1	45.00	Clostridiales	102627	2705166

Sample_SCC55.Cluster1689	52.10	Firmicutes	49957	1925391
Sample_SCC55.mb.72cbin.1	28.00	Euryarchaeota	59661	1755249
Sample_SCC55.mb.33cbin.1	36.60	Clostridiales	7737	1595764
Sample_SCC55.Cluster3849	43.50	Lachnospiraceae	8503	1795565
Sample_SCC55.Cluster7696cbin.1	37.80	Lactobacillales	10618	1624360
Sample_SCC55.mb.24cbin.1	59.00	Clostridiales	16755	2254503
Sample_SCC55.mb.19cbin.1	38.70	Clostridiales	68197	2140189
Sample_SCC55.Cluster435	58.20	Veillonellaceae	335488	2486379
Sample_SCC55.mb.28	41.90	Clostridiales	118045	2437771
Sample_SCC55.mb.75	33.70	Bacteria	14696	2166807
Sample_SCC55.mb.3cbin.1	44.00	Lachnospiraceae	20530	2445077
Sample_SCC55.mb.66	53.90	Clostridiales	194712	1807381
Sample_SCC55.mb.29	44.30	Clostridiales	95449	2651362
Sample_SCC55.Cluster7720cbin.1	41.30	Lachnospiraceae	60855	3055665
Sample_SCC55.mb.55	26.90	Clostridiales	26671	2232570
Sample_SCC55.mb.9	36.70	Gammaproteobacteria	87050	2151661
Sample_SCC55.mb.41	28.50	Clostridiales	68154	3218793
Sample_SCC55.mb.71	35.80	Clostridiales	95726	2589859
Sample_SCC55.Cluster680cbin.1	56.00	Enterobacteriaceae	277787	5219743
Sample_SCC58.mb.103	27.40	Bacteria	59542	931514
Sample_SCC58.mb.108cbin.1	26.40	Bacteria	89896	1539048
Sample_SCC58.mb.31	24.00	Bacteria	59798	959603
Sample_SCC58.Cluster37cbin.1	61.50	Clostridiales	13809	1819780
Sample_SCC58.Cluster161	59.80	Clostridiales	13597	1782010
Sample_SCC58.Cluster4392	54.20	Actinobacteria	170377	1619471
Sample_SCC58.mb.17cbin.1	49.80	Clostridiales	17572	1895313
Sample_SCC58.mb.37cbin.1	53.80	Clostridia	30427	1754205
Sample_SCC58.mb.26cbin.1	58.50	Clostridia	79334	2353649
Sample_SCC58.Cluster13902cbin.1	34.10	Bacilli	46338	2745172
Sample_SCC58.mb.19	38.40	Clostridiales	12091	2190396
Sample_SCC58.mb.20	58.10	Clostridiales	61576	3035836
Sample_SCC58.mb.68	25.60	Bacteria	37578	1155912
Sample_SCC58.Cluster2946	54.80	Bacteroidetes	149642	2036202
Sample_SCC58.Cluster11902cbin.1	40.90	Clostridiales	197398	1977503
Sample_SCC58.mb.32cbin.1	57.80	Clostridiales	28709	2520451
Sample_SCC58.mb.83	26.30	Bacteria	74335	1187207
Sample_SCC58.mb.109	60.60	Bacteroidetes	144992	2024653
Sample_SCC58.mb.66	49.50	Clostridiales	73977	1898861
Sample_SCC58.mb.63	58.50	Clostridia	12246	2899335
Sample_SCC58.Cluster5765cbin.1	51.50	Clostridiales	157337	2228294
Sample_SCC58.mb.41cbin.1	48.60	Clostridiales	13040	2735660
Sample_SCC58.mb.74	60.70	Actinobacteria	13743	1786456
Sample_SCC58.Cluster849	55.70	Bacteria	156029	2770254
Sample_SCC58.mb.95cbin.1	49.10	Clostridia	43084	1437069
Sample_SCC58.mb.80	48.50	Clostridiales	63664	1775123
Sample_SCC58.mb.106	44.40	Clostridiales	63453	2516818
Sample_SCC58.mb.79cbin.1	59.50	Clostridiales	46500	2366492
Sample_SCC58.mb.72	61.80	Clostridiales	16993	2449326
Sample_SCC58.Cluster10136	43.30	Bacteroidales	158704	3056788
Sample_SCC58.mb.34	43.50	Lachnospiraceae	71602	2850478
Sample_SCC58.mb.71	60.50	Clostridiales	53310	2348781
Sample_SCC58.mb.87cbin.1	59.80	Actinobacteria	29068	2130196
Sample_SCC58.mb.78cbin.1	58.00	Proteobacteria	8100	1914521
Sample_SCC58.mb.90	56.40	Clostridiales	14268	2462894
Sample_SCC58.Cluster4938cbin.1	45.50	Bacteroidales	16567	4161940
Sample_SCC58.Cluster7703cbin.1	45.60	Bacteroidales	50715	3968723
Sample_SCC58.mb.93cbin.1	59.90	Bifidobacteriaceae	125838	1940809

Sample_SCC58.mb.101	41.70	Bacteroidales	54911	4808549
Sample_SCC58.Cluster176cbin.1	56.60	Enterobacteriaceae	9294	4053673
Sample_SCC59.mb.27cbin.1	59.70	Actinobacteria	5019	1349896
Sample_SCC59.mb.36	59.60	Clostridia	50794	2624182
Sample_SCC59.Cluster7017cbin.1	44.90	Actinobacteria	59380	1520952
Sample_SCC59.mb.12cbin.1	36.90	Clostridiales	21681	2814161
Sample_SCC59.mb.84	27.10	Bacteria	25453	3167295
Sample_SCC59.mb.9	26.70	Bacteria	126572	1314473
Sample_SCC59.Cluster16cbin.1	59.20	Clostridiales	10140	2147135
Sample_SCC59.mb.70cbin.1	27.40	Euryarchaeota	45746	1702406
Sample_SCC59.Cluster8562	42.90	Clostridiales	165614	1858094
Sample_SCC59.mb.93	49.20	Clostridiales	81920	2008241
Sample_SCC59.mb.34	60.20	Clostridiales	26315	1969418
Sample_SCC59.Cluster3093cbin.1	51.70	Clostridiales	199794	2339255
Sample_SCC59.mb.48	58.80	Bacteroidetes	64607	2427089
Sample_SCC59.Cluster9725	40.50	Clostridiales	255037	2191615
Sample_SCC59.mb.24	31.20	Euryarchaeota	116376	1723080
Sample_SCC59.mb.47cbin.1	45.70	Clostridiales	6309	2064546
Sample_SCC59.mb.33	54.50	Bacteroidetes	105154	2880680
Sample_SCC59.mb.87	59.00	Clostridiales	10265	2246193
Sample_SCC59.mb.90	62.20	Clostridiales	163568	1992682
Sample_SCC59.Cluster6670cbin.1	43.20	Bacteria	34398	3944941
Sample_SCC59.mb.10	43.20	Lachnospiraceae	49341	2911581
Sample_SCC59.Cluster4295cbin.1	47.30	Bacteroidales	28255	3889731
Sample_SCC59.mb.71cbin.1	43.80	Bacteria	39610	4344464
Sample_SCC59.Cluster6696cbin.1	45.00	Bacteroidales	28540	4654287
Sample_SCC60.Cluster16100	24.10	Bacteria	61208	978575
Sample_SCC60.Cluster16043cbin.1	26.10	Bacteria	20485	1145233
Sample_SCC60.Cluster13861	29.40	Bacteria	42112	1619639
Sample_SCC60.mb.14	47.50	Clostridiales	50982	1479445
Sample_SCC60.mb.32	29.50	Bacteria	28456	1493583
Sample_SCC60.mb.12	50.00	Clostridiales	7648	1847983
Sample_SCC60.Cluster214cbin.1	60.20	Actinobacteria	11659	1906009
Sample_SCC60.mb.31	57.10	Clostridiales	15435	2052326
Sample_SCC60.mb.56	53.20	Clostridia	25456	1971980
Sample_SCC60.mb.10	56.90	Clostridiales	15589	2364194
Sample_SCC60.Cluster15816	31.10	Euryarchaeota	176455	1783190
Sample_SCC60.Cluster12433	40.60	Clostridiales	125486	2075345
Sample_SCC60.mb.24cbin.1	47.20	Clostridiales	70625	2505181
Sample_SCC60.mb.65	58.30	Clostridiales	48984	2846214
Sample_SCC60.mb.43	41.40	Clostridiales	6755	2449201
Sample_SCC60.Cluster11285cbin.1	39.50	Clostridiales	98969	2187446
Sample_SCC60.Cluster12028	41.70	Clostridiales	127423	2472923
Sample_SCC60.Cluster236	55.60	Bacteria	165290	2688913
Sample_SCC60.mb.57	59.60	Clostridiales	7524	1792445
Sample_SCC60.mb.28	49.70	Bacteroidales	21419	2825226
Sample_SCC60.mb.21	45.60	Clostridiales	15110	1789366
Sample_SCC60.mb.51	59.60	Bacteria	51525	4061558
Sample_SCC60.mb.35cbin.1	45.10	Clostridiales	56228	1669468
Sample_SCC60.mb.2	60.30	Bacteroidetes	41702	2460178
Sample_SCC60.mb.76	52.90	Clostridiales	103103	2350774
Sample_SCC60.mb.60	62.60	Clostridiales	29536	1935457
Sample_SCC60.mb.48cbin.1	57.50	Clostridiales	34979	3289425
Sample_SCC60.mb.45cbin.1	52.20	Firmicutes	50312	2425342
Sample_SCC60.mb.64cbin.1	56.40	Clostridiales	15830	2394328
Sample_SCC60.mb.84cbin.1	42.60	Clostridiales	45708	3365535
Sample_SCC60.Cluster9124cbin.1	43.40	Bacteroidales	145074	2937227
Sample_SCC60.mb.67	51.20	Clostridiales	102509	2174118

Sample_SCC60.mb.87	45.20	Clostridiales	80935	2381095
Sample_SCC60.mb.99	36.40	Clostridiales	153076	1967929
Sample_SCC60.Cluster5145cbin.1	43.30	Bacteria	28127	3924032
Sample_SCC60.mb.13	41.90	Clostridiales	33802	3268147
Sample_SCC60.mb.9	57.30	Bacteria	47671	2523753
Sample_SCC60.mb.75cbin.1	43.20	Lachnospiraceae	20379	2128385
Sample_SCC60.mb.86cbin.1	54.40	Bacteroidetes	106186	2152195
Sample_SCC60.mb.81	36.00	Clostridiales	40626	2349664
Sample_SCC60.Cluster6558cbin.1	46.70	Bacteroidales	47798	4201584
Sample_SCC60.mb.93	41.50	Lachnospiraceae	113870	2695583
Sample_SCC61.mb.33	47.30	Bacteria	46149	1754855
Sample_SCC61.Cluster4039	41.80	Clostridiales	83209	2676206
Sample_SCC61.mb.94	41.00	Bacteria	37365	3216241
Sample_SCC61.mb.29cbin.1	37.10	Clostridiales	42919	3076821
Sample_SCC61.Cluster213cbin.1	59.30	Bacteroidetes	8385	2102699
Sample_SCC61.mb.79cbin.1	33.60	Clostridiales	351073	2075784
Sample_SCC61.mb.12cbin.1	60.00	Clostridiales	8913	1914705
Sample_SCC61.mb.42	49.60	Clostridiales	14211	2257710
Sample_SCC61.mb.53cbin.1	48.10	Clostridiales	97885	2998385
Sample_SCC61.Cluster175	59.90	Bacteroidetes	235470	2686219
Sample_SCC61.Cluster3441cbin.1	43.60	Lachnospiraceae	22749	1882799
Sample_SCC61.mb.13	60.90	Clostridiales	25433	2086389
Sample_SCC61.Cluster307cbin.1	55.30	Bacteria	181025	2684675
Sample_SCC61.Cluster4758cbin.1	40.90	Clostridiales	178753	2022119
Sample_SCC61.Cluster3659	44.00	Clostridiales	275569	2743854
Sample_SCC61.Cluster620	57.30	Proteobacteria	109781	2349030
Sample_SCC61.mb.32	44.20	Clostridiales	47812	3299608
Sample_SCC61.mb.68	42.40	Clostridiales	55200	2389323
Sample_SCC61.mb.69cbin.1	46.60	Lachnospiraceae	85036	2914910
Sample_SCC61.mb.23cbin.1	57.80	Clostridiales	26763	2092551
Sample_SCC61.mb.78cbin.1	38.20	Clostridiales	52428	1876355
Sample_SCC61.mb.92cbin.1	59.90	Clostridiales	8990	2118655
Sample_SCC61.Cluster6030cbin.1	37.20	Clostridiales	119792	3004474
Sample_SCC61.Cluster4061cbin.1	41.60	Lachnospiraceae	39011	3650243
Sample_SCC61.Cluster3270cbin.1	43.30	Bacteria	13745	3728446
Sample_SCC61.mb.85cbin.1	26.90	Clostridiales	22775	2934716
Sample_SCC61.mb.41cbin.1	41.30	Bacteroidales	35684	4249544
Sample_SCC61.Cluster2650cbin.1	45.70	Bacteroidales	40911	3948441
Sample_SCC61.Cluster3036cbin.1	45.00	Bacteroidales	159855	4519323
Sample_SCC61.Cluster1752	50.70	Enterobacteriaceae	58387	4632836
Sample_SCC64.mb.26	49.50	Clostridiales	78074	1923290
Sample_SCC64.mb.22	58.30	Clostridiales	63603	2805537
Sample_SCC64.Cluster3622cbin.1	44.90	Clostridiales	66587	2575703
Sample_SCC64.mb.52cbin.1	62.20	Clostridiales	7320	1467173
Sample_SCC64.mb.25	61.40	Clostridiales	10550	2459091
Sample_SCC64.Cluster662cbin.1	52.60	Firmicutes	6799	1971881
Sample_SCC64.Cluster61cbin.1	59.40	Clostridiales	37241	2328675
Sample_SCC64.Cluster5107	40.90	Clostridiales	116065	2005020
Sample_SCC64.Cluster7354cbin.1	29.70	Bacteria	32266	2339524
Sample_SCC64.Cluster276cbin.1	56.40	Bifidobacteriaceae	52632	1818498
Sample_SCC64.mb.13cbin.1	60.10	Bacteroidetes	50621	2510444
Sample_SCC64.mb.11	60.00	Bifidobacteriaceae	104533	2167920
Sample_SCC64.Cluster820	55.50	Proteobacteria	161475	2276536
Sample_SCC64.Cluster6936cbin.1	35.80	Clostridiales	172146	2530932
Sample_SCC64.mb.63	44.90	Clostridiales	28354	2288763
Sample_SCC64.mb.18	41.80	Lachnospiraceae	50018	2590505
Sample_SCC64.mb.79	54.50	Bacteroidetes	103756	2228725
Sample_SCC64.mb.67	46.40	Lachnospiraceae	68771	3563060

Sample_SCC64.Cluster2663	47.90	Bacteroidales	100998	2789200
Sample_SCC64.Cluster7245	31.40	Firmicutes	50122	2319226
Sample_SCC64.mb.43cbin.1	57.60	Clostridiales	51322	2583381
Sample_SCC64.mb.54	37.20	Clostridiales	124635	2872449
Sample_SCC64.mb.45	49.00	Proteobacteria	12540	2302564
Sample_SCC64.mb.24	40.60	Bacteroidales	55292	3255452
Sample_SCC64.mb.38	43.40	Bacteroidales	162755	3093087
Sample_SCC64.mb.47cbin.1	41.10	Lachnospiraceae	54946	3626557
Sample_SCC64.Cluster2242	45.40	Bacteroidales	112893	4075472
Sample_SCC64.Cluster3650cbin.1	42.80	Bacteria	54465	4488505
Sample_SCC64.Cluster3045	45.00	Bacteroidales	180035	4537407
Sample_SCC64.mb.46cbin.1	46.90	Bacteroidales	42003	4031528
Sample_SCC64.mb.29	41.70	Bacteroidales	166563	4641649
Sample_SCC65.Cluster2480cbin.1	49.40	Bacteria	62766	1773897
Sample_SCC65.mb.51cbin.1	40.60	Bacteria	186583	2746275
Sample_SCC65.Cluster781cbin.1	55.70	Clostridiales	6642	1971094
Sample_SCC65.mb.22	38.20	Clostridiales	58819	2664900
Sample_SCC65.mb.32	54.30	Actinobacteria	31356	1575196
Sample_SCC65.Cluster2641	48.20	Clostridiales	106481	2901453
Sample_SCC65.mb.7cbin.1	42.40	Clostridiales	9333	2309936
Sample_SCC65.Cluster5070	40.50	Lachnospiraceae	134433	2748924
Sample_SCC65.mb.69cbin.1	61.90	Clostridiales	7879	2229830
Sample_SCC65.Cluster520	55.00	Bacteroidetes	122773	1966468
Sample_SCC65.mb.2	56.00	Bacteria	6422	2158663
Sample_SCC65.Cluster5719	38.70	Clostridiales	100215	2048021
Sample_SCC65.mb.70cbin.1	51.50	Clostridiales	131036	2607252
Sample_SCC65.mb.58cbin.1	37.20	Clostridiales	7200	2201356
Sample_SCC65.Cluster13cbin.1	63.30	Actinobacteria	57276	2901411
Sample_SCC65.mb.33cbin.1	41.30	Clostridiales	189426	2611112
Sample_SCC65.Cluster6213cbin.1	37.30	Clostridiales	97019	2782195
Sample_SCC65.mb.45	44.90	Clostridiales	104966	2702787
Sample_SCC65.mb.77cbin.1	59.30	Bacteroidetes	32195	2911331
Sample_SCC65.mb.28	48.40	Proteobacteria	28861	2613848
Sample_SCC65.mb.53cbin.1	42.20	Bacteria	6333	3631288
Sample_SCC65.mb.42cbin.1	42.10	Bacteroidales	45184	4073938
Sample_SCC65.Cluster3329cbin.1	46.50	Bacteroidales	141755	4431026
Sample_SCC65.mb.16	45.20	Bacteroidales	46924	4555689
Sample_SCC65.mb.52	43.50	Bacteroidales	169644	5892455
Sample_SCC66.Cluster3790	49.40	Clostridia	107272	1834397
Sample_SCC66.Cluster1441cbin.1	58.20	Clostridiales	50519	3017756
Sample_SCC66.mb.15	26.00	Bacteria	89979	1352507
Sample_SCC66.Cluster294cbin.1	58.40	Clostridiales	39272	2767071
Sample_SCC66.Cluster2799cbin.1	44.00	Selenomonadales	9467	1882874
Sample_SCC66.Cluster8cbin.1	62.30	Clostridiales	11332	2239094
Sample_SCC66.mb.118	36.40	Clostridiales	68418	1881212
Sample_SCC66.Cluster6670cbin.1	47.00	Lachnospiraceae	19523	3036224
Sample_SCC66.mb.6cbin.1	53.40	Clostridia	9481	1735970
Sample_SCC66.Cluster438	55.60	Bacteria	204838	2714050
Sample_SCC66.Cluster8261cbin.1	40.50	Clostridiales	278999	2193314
Sample_SCC66.mb.57cbin.1	54.80	Actinobacteria	7477	1325155
Sample_SCC66.Cluster8780	37.70	Clostridiales	32432	2348914
Sample_SCC66.mb.33	54.80	Bacteroidetes	123344	1995773
Sample_SCC66.mb.54	46.70	Lactobacillales	87969	1986295
Sample_SCC66.Cluster5669cbin.1	44.10	Bacteroidales	10132	2912860
Sample_SCC66.Cluster1357	55.40	Proteobacteria	114695	2698231
Sample_SCC66.Cluster1223cbin.1	59.00	Bacteroidetes	186027	3173294
Sample_SCC66.mb.32cbin.1	55.90	Clostridiales	12734	2534760
Sample_SCC66.mb.90cbin.1	59.10	Clostridiales	21419	1932843

Sample_SCC66.mb.114cbin.1	44.10	Clostridiales	52191	2816621
Sample_SCC66.mb.87	48.10	Clostridiales	34593	2883518
Sample_SCC66.mb.109cbin.1	42.50	Clostridiales	118919	2927059
Sample_SCC66.mb.107cbin.1	44.70	Clostridiales	26541	3000534
Sample_SCC66.Cluster5209	43.20	Bacteria	48129	3991226
Sample_SCC66.mb.100	42.10	Bacteroidales	48504	3468933
Sample_SCC66.mb.97cbin.1	52.20	Clostridiales	149240	2359627
Sample_SCC66.mb.27	58.50	Bifidobacteriaceae	137344	2327626
Sample_SCC66.mb.2cbin.1	40.90	Lachnospiraceae	42780	3145928
Sample_SCC66.mb.88cbin.1	44.20	Clostridiales	149608	2091988
Sample_SCC66.Cluster4739cbin.1	45.00	Bacteroidales	134771	4376084
Sample_SCC66.mb.82cbin.1	60.00	Bifidobacteriaceae	39133	2640794
Sample_SCC66.mb.86	59.40	Bacteroidetes	47569	2588001
Sample_SCC66.mb.7	43.70	Bacteroidales	337152	3290009
Sample_SCC67.Cluster4189cbin.1	53.20	Clostridiales	25865	1930671
Sample_SCC67.mb.12	26.10	Bacteria	56681	1375372
Sample_SCC67.Cluster3149cbin.1	52.00	Clostridiales	22577	1989646
Sample_SCC67.Cluster11226cbin.1	44.90	Clostridiales	84186	2536649
Sample_SCC67.mb.14	36.30	Clostridiales	203773	1914967
Sample_SCC67.mb.114	56.10	Clostridiales	18682	2215265
Sample_SCC67.mb.126	58.40	Clostridiales	35538	1944605
Sample_SCC67.mb.69	49.60	Clostridia	6381	1612232
Sample_SCC67.mb.28	61.10	Clostridiales	32817	1733454
Sample_SCC67.Cluster15193cbin.1	41.80	Clostridiales	178729	2435220
Sample_SCC67.Cluster11439	44.50	Clostridiales	329789	2134847
Sample_SCC67.mb.55cbin.1	49.30	Bacteria	10294	1736955
Sample_SCC67.Cluster15443	38.70	Clostridiales	78024	2165164
Sample_SCC67.mb.105cbin.1	51.10	Bacteroidales	52430	2573801
Sample_SCC67.Cluster8360cbin.1	52.50	Bacteroidales	67480	2741928
Sample_SCC67.Cluster45cbin.1	60.90	Bacteroidetes	10298	2511364
Sample_SCC67.mb.35	56.40	Clostridiales	68344	2280582
Sample_SCC67.Cluster8834cbin.1	44.90	Prevotella	17536	3201554
Sample_SCC67.mb.26cbin.1	55.90	Bacteroidetes	6569	2173996
Sample_SCC67.Cluster15804cbin.1	38.60	Streptococcus	30129	2026902
Sample_SCC67.Cluster11250	41.70	Lachnospiraceae	6178	2605401
Sample_SCC67.mb.18	56.40	Clostridiales	14053	2317352
Sample_SCC67.mb.5cbin.1	61.30	Clostridiales	56510	2078145
Sample_SCC67.mb.62	58.50	Clostridiales	6400	1855581
Sample_SCC67.mb.101cbin.1	43.00	Clostridiales	213310	2854274
Sample_SCC67.Cluster12373cbin.1	41.10	Lachnospiraceae	53048	3094945
Sample_SCC67.mb.2	43.10	Lachnospiraceae	33150	2119243
Sample_SCC67.mb.45	53.90	Clostridiales	10893	1884223
Sample_SCC67.mb.75	57.20	Clostridiales	105583	2340995
Sample_SCC67.mb.52cbin.1	51.80	Clostridiales	16190	1940214
Sample_SCC67.mb.103	59.20	Clostridiales	24207	3524372
Sample_SCC67.mb.73cbin.1	57.20	Clostridiales	17551	2351661
Sample_SCC67.mb.97cbin.1	62.00	Clostridiales	41201	2031303
Sample_SCC67.mb.131	43.40	Bacteroidales	82485	3201282
Sample_SCC67.mb.90cbin.1	47.80	Bacteroidales	16634	2940190
Sample_SCC67.mb.63	42.20	Lachnospiraceae	6175	1937881
Sample_SCC67.mb.95cbin.1	54.30	Bacteroidetes	110446	2269693
Sample_SCC67.mb.77cbin.1	43.80	Lachnospiraceae	24006	2775139
Sample_SCC67.mb.74cbin.1	42.60	Clostridiales	71737	2621763
Sample_SCC68.mb.39	53.20	Clostridia	8972	1862779
Sample_SCC68.mb.17	53.50	Clostridiales	36368	1753906
Sample_SCC68.Cluster3923	49.50	Clostridiales	101610	1997849
Sample_SCC68.mb.58	26.40	Bacteria	124727	1242084
Sample_SCC68.mb.23cbin.1	48.80	Clostridiales	7849	1472108

Sample_SCC68.mb.22cbin.1	58.60	Clostridiales	5366	2405802
Sample_SCC68.Cluster829cbin.1	59.20	Clostridiales	70746	2288181
Sample_SCC68.mb.38	58.20	Clostridiales	24801	2112158
Sample_SCC68.Cluster193cbin.1	57.00	Clostridiales	53287	1982061
Sample_SCC68.mb.33	57.70	Clostridiales	8694	2062348
Sample_SCC68.mb.18	52.80	Clostridiales	30585	2202779
Sample_SCC68.Cluster4067cbin.1	47.40	Lactobacillales	23354	1734091
Sample_SCC68.mb.41	54.50	Clostridiales	40092	2101823
Sample_SCC68.mb.61	60.30	Clostridiales	73656	1881337
Sample_SCC68.mb.72cbin.1	29.60	Bacteria	51997	1667013
Sample_SCC68.Cluster6989cbin.1	42.20	Clostridiales	5363	1765977
Sample_SCC68.mb.45cbin.1	59.80	Clostridiales	22668	2380055
Sample_SCC68.mb.50	60.70	Bacteria	105495	4738247
Sample_SCC68.mb.19cbin.1	59.80	Bacteroidetes	101361	2576495
Sample_SCC68.mb.28cbin.1	51.10	Firmicutes	37819	2136692
Sample_SCC68.mb.35	39.00	Clostridiales	17119	1959920
Sample_SCC68.Cluster9019cbin.1	37.70	Clostridiales	5244	2151750
Sample_SCC68.mb.68cbin.1	59.80	Clostridiales	71954	2060943
Sample_SCC68.mb.1	44.40	Clostridiales	142843	2285841
Sample_SCC68.mb.44cbin.1	51.50	Clostridiales	117357	2156080
Sample_SCC68.Cluster9947cbin.1	38.00	Lactobacillus	113554	1855726
Sample_SCC68.mb.59cbin.1	55.20	Clostridiales	68120	3608615
Sample_SCC68.mb.66	53.90	Clostridiales	49608	1866355
Sample_SCC68.mb.60	58.90	Bacteroidetes	86340	2902571
Sample_SCC68.mb.20	40.70	Lachnospiraceae	82888	3273680
Sample_SCC68.mb.10	46.80	Bacteroidales	83853	4013310
Sample_SCC68.mb.49	42.00	Bacteroidales	41653	4330874
Sample_SCC68.mb.24cbin.1	50.80	Enterobacteriaceae	25607	4474331
Sample_SCC69.mb.31	52.90	Clostridia	68512	2092306
Sample_SCC69.mb.56cbin.1	53.70	Clostridia	18162	1684712
Sample_SCC69.mb.37	58.30	Clostridiales	56069	3002733
Sample_SCC69.Cluster248cbin.1	59.80	Clostridiales	102074	1925109
Sample_SCC69.Cluster2319cbin.1	62.50	Clostridiales	90962	1872647
Sample_SCC69.mb.28cbin.1	62.30	Actinobacteria	164804	2280410
Sample_SCC69.mb.15cbin.1	41.60	Clostridiales	12308	3630009
Sample_SCC69.mb.29	61.50	Clostridiales	81947	2147830
Sample_SCC69.Cluster3524cbin.1	51.50	Clostridiales	119488	2551619
Sample_SCC69.mb.34cbin.1	59.50	Clostridiales	4977	1815383
Sample_SCC69.mb.57	53.50	Actinobacteria	57957	1601083
Sample_SCC69.mb.30cbin.1	56.10	Clostridiales	95076	2486743
Sample_SCC69.mb.32	58.10	Clostridiales	10354	2352745
Sample_SCC69.mb.22cbin.1	59.90	Actinobacteria	37673	2266242
Sample_SCC69.Cluster3324cbin.1	51.10	Firmicutes	37226	2246799
Sample_SCC69.Cluster10971	30.90	Euryarchaeota	93703	1788042
Sample_SCC69.Cluster10644	32.60	Lactobacillales	7263	1655406
Sample_SCC69.mb.67	49.60	Clostridiales	114414	1918279
Sample_SCC69.Cluster525cbin.1	59.70	Bifidobacteriaceae	111761	1900553
Sample_SCC69.Cluster1876	56.30	Selenomonadales	109439	2170438
Sample_SCC69.mb.25cbin.1	58.50	Bacteria	15845	2797401
Sample_SCC69.mb.62	57.30	Clostridiales	69387	1878182
Sample_SCC69.mb.46cbin.1	50.70	Lactobacillales	7369	1648042
Sample_SCC69.Cluster9042cbin.1	41.90	Lachnospiraceae	49824	2645354
Sample_SCC69.mb.35	60.10	Bifidobacteriaceae	30215	2206372
Sample_SCC69.mb.68cbin.1	57.30	Clostridiales	132753	1839892
Sample_SCC69.mb.55	62.80	Bifidobacteriaceae	123014	1994249
Sample_SCC69.Cluster6823cbin.1	44.60	Prevotella	61905	3709519
Sample_SCC69.mb.21	43.50	Bacteroidales	137044	3156269
Sample_SCC69.Cluster5299cbin.1	43.40	Bacteria	8953	3577489

Sample_SCC69.mb.58cbin.1	58.50	Clostridiales	58152	2551893
Sample_SCC69.mb.70cbin.1	51.60	Clostridiales	157225	2063079
Sample_SCC69.mb.52cbin.1	41.20	Lachnospiraceae	20109	3141173
Sample_SCC69.mb.60	41.80	Clostridiales	84225	3266613
Sample_SCC69.mb.9	55.20	Bacteroidetes	38803	2110662
Sample_SCC69.mb.75	44.50	Clostridiales	151633	2623332
Sample_SCC69.mb.83	43.20	Clostridiales	9809	2367932
Sample_SCC69.mb.69	43.60	Lachnospiraceae	24215	2520026
Sample_SCC69.mb.43cbin.1	42.00	Bacteroides	67119	4509918
Sample_SCC69.mb.72cbin.1	46.30	Bacteroidales	134245	4127606
Sample_SCC69.mb.81cbin.1	41.90	Bacteroidales	91509	4594420
Sample_SCC69.Cluster668cbin.1	57.10	Enterobacteriaceae	55643	5125082
Sample_SCC69.mb.7cbin.1	50.60	Enterobacteriaceae	29637	5191722
Sample_SCC70.mb.18	24.10	Bacteria	80062	955148
Sample_SCC70.Cluster7894	47.40	Clostridiales	495522	1560132
Sample_SCC70.mb.33	25.20	Bacteria	71846	1308088
Sample_SCC70.Cluster12265cbin.1	37.60	Clostridiales	192994	2412853
Sample_SCC70.mb.26	52.80	Clostridia	55404	2030941
Sample_SCC70.Cluster1298cbin.1	57.00	Clostridiales	43218	2295128
Sample_SCC70.mb.49	44.80	Bacteria	247093	1561856
Sample_SCC70.Cluster4194cbin.1	49.30	Bacteroidales	6973	2654828
Sample_SCC70.mb.60	27.70	Bacteria	53578	1128595
Sample_SCC70.mb.15cbin.1	59.20	Clostridiales	31905	1814297
Sample_SCC70.mb.110cbin.1	58.00	Clostridiales	37480	2094257
Sample_SCC70.Cluster6984	47.50	Lachnospiraceae	48642	2519287
Sample_SCC70.Cluster6586	47.60	Selenomonadales	89802	2125775
Sample_SCC70.mb.28	51.40	Bacteroidetes	49396	3140576
Sample_SCC70.Cluster12184	38.70	Clostridiales	80706	1972328
Sample_SCC70.mb.69cbin.1	49.50	Bacteria	8222	1604931
Sample_SCC70.mb.48	54.00	Clostridiales	12063	1619260
Sample_SCC70.Cluster8175cbin.1	49.20	Bacteroidales	44462	3067605
Sample_SCC70.mb.16	47.20	Clostridiales	77435	2562539
Sample_SCC70.mb.58	58.40	Clostridiales	18375	2853548
Sample_SCC70.Cluster4154	52.80	Clostridiales	122087	2267011
Sample_SCC70.Cluster9642cbin.1	41.90	Clostridiales	101602	2358046
Sample_SCC70.mb.105	31.20	Euryarchaeota	102764	1760231
Sample_SCC70.Cluster12153cbin.1	44.00	Clostridiales	116535	2409555
Sample_SCC70.mb.34	44.80	Rhodospirillales	55672	2039767
Sample_SCC70.mb.20cbin.1	43.90	Lachnospiraceae	105701	3077349
Sample_SCC70.Cluster6180	45.30	Clostridiales	21073	2807159
Sample_SCC70.Cluster11577cbin.1	41.40	Lachnospiraceae	30849	2771901
Sample_SCC70.mb.78cbin.1	36.40	Clostridiales	155214	1857047
Sample_SCC70.mb.91cbin.1	42.80	Clostridia	6207	1166021
Sample_SCC70.mb.102	42.40	Clostridiales	24346	2677249
Sample_SCC70.Cluster6980cbin.1	45.10	Prevotella	30476	3696268
Sample_SCC70.mb.50	56.60	Clostridiales	8924	2312550
Sample_SCC70.mb.71	57.80	Clostridiales	33958	1919995
Sample_SCC70.mb.109cbin.1	50.10	Bacteroidales	56867	2460378
Sample_SCC70.mb.52cbin.1	64.30	Deltaproteobacteria	5477	1877637
Sample_SCC70.mb.77	59.20	Clostridiales	12342	1763038
Sample_SCC70.mb.85	55.20	Clostridia	40386	2540644
Sample_SCC70.mb.40	50.40	Bacteroidales	7438	2131338
Sample_SCC70.mb.97	57.20	Clostridiales	47881	2418020
Sample_SCC70.mb.76cbin.1	52.80	Bacteroidales	64999	2429576
Sample_SCC70.mb.81	43.60	Lachnospiraceae	57817	3477129
Sample_SCC70.mb.93cbin.1	51.30	Clostridiales	82625	3044952
Sample_SCC70.mb.88	40.90	Clostridiales	229767	2355826
Sample_SCC70.mb.92	46.40	Prevotella	35253	2902092

Sample_SPA03.Cluster14037cbin.1	25.90	Bacteria	136763	1279079
Sample_SPA03.Cluster11746cbin.1	37.40	Clostridiales	255743	2168871
Sample_SPA03.mb.22	26.90	Bacteria	60429	1268246
Sample_SPA03.Cluster260cbin.1	60.80	Clostridiales	48646	1890545
Sample_SPA03.Cluster4871cbin.1	48.40	Clostridiales	142161	2669750
Sample_SPA03.mb.116	38.30	Clostridiales	27527	2387534
Sample_SPA03.mb.39	51.10	Bacteria	58713	1874357
Sample_SPA03.Cluster8013cbin.1	42.40	Clostridiales	50215	3604876
Sample_SPA03.Cluster1587	55.00	Bacteroidetes	87851	1940411
Sample_SPA03.mb.23cbin.1	57.90	Clostridiales	46640	3211972
Sample_SPA03.Cluster3083cbin.1	48.70	Firmicutes	18240	1881424
Sample_SPA03.mb.18	56.60	Clostridiales	24276	2535398
Sample_SPA03.Cluster3223cbin.1	48.90	Bacteroidales	116443	2986403
Sample_SPA03.Cluster35	65.20	Proteobacteria	38533	2398288
Sample_SPA03.mb.35	53.60	Clostridiales	18711	2448118
Sample_SPA03.Cluster8475cbin.1	43.00	Clostridiales	173059	2879040
Sample_SPA03.mb.118	53.50	Clostridiales	26182	2143326
Sample_SPA03.mb.119cbin.1	59.20	Bacteroidetes	107543	3036303
Sample_SPA03.mb.24	58.90	Clostridiales	18912	2379244
Sample_SPA03.mb.2	33.70	Clostridiales	21645	1999791
Sample_SPA03.mb.38	46.90	Lachnospiraceae	13164	2640034
Sample_SPA03.Cluster453cbin.1	56.00	Proteobacteria	8275	2465102
Sample_SPA03.mb.66cbin.1	41.90	Clostridiales	30701	2268864
Sample_SPA03.mb.11	59.10	Bacteroidetes	186665	2715641
Sample_SPA03.mb.50cbin.1	58.50	Clostridiales	20611	2529864
Sample_SPA03.mb.19	44.20	Clostridiales	157392	2763804
Sample_SPA03.Cluster6910cbin.1	43.40	Bacteroidales	421458	3149661
Sample_SPA03.mb.62cbin.1	41.40	Lachnospiraceae	73242	2215652
Sample_SPA03.mb.27	37.40	Clostridiales	51469	2648781
Sample_SPA03.Cluster6052cbin.1	43.30	Bacteria	24591	3897944
Sample_SPA03.mb.93	41.10	Clostridiales	33190	2596756
Sample_SPA03.mb.51cbin.1	56.20	Clostridiales	13391	2496120
Sample_SPA03.mb.54	60.50	Clostridiales	40266	2388302
Sample_SPA03.mb.69	45.50	Lachnospiraceae	29649	2376933
Sample_SPA03.mb.92cbin.1	44.90	Clostridiales	107523	2627269
Sample_SPA03.mb.7	52.80	Clostridiales	493272	2009618
Sample_SPA03.Cluster6964cbin.1	46.10	Bacteroidales	72826	3784325
Sample_SPA03.Cluster5354	45.30	Bacteroidales	79640	4312955
Sample_SPA03.mb.64	40.70	Clostridiales	6223	2444192
Sample_SPA03.mb.77	59.70	Bacteroidetes	137703	2579460
Sample_SPA03.Cluster5960	44.90	Bacteroidales	96387	4581501
Sample_SPA03.mb.98	35.80	Clostridiales	46011	2551675
Sample_SPA03.mb.90	49.90	Lachnospiraceae	75440	2817590
Sample_SPA03.mb.40	42.20	Bacteroidales	49194	4413723
Sample_SPA03.mb.47cbin.1	57.60	Enterobacteriaceae	77652	5165760
Sample_SPA04.Cluster15261cbin.1	36.20	Bacteria	47794	2857125
Sample_SPA04.mb.17	26.80	Bacteria	84722	1141751
Sample_SPA04.Cluster368cbin.1	58.70	Clostridiales	51624	2100936
Sample_SPA04.Cluster7492cbin.1	43.80	Clostridiales	38976	2756035
Sample_SPA04.mb.14cbin.1	49.50	Clostridiales	90490	1876046
Sample_SPA04.Cluster1305cbin.1	55.10	Bacteroidetes	29785	1903017
Sample_SPA04.Cluster6010cbin.1	47.50	Lachnospiraceae	34613	2657614
Sample_SPA04.Cluster1798cbin.1	57.30	Proteobacteria	35834	1834535
Sample_SPA04.Cluster5326	46.80	Selenomonadales	116162	2282812
Sample_SPA04.mb.42	26.10	Bacteria	98561	1257940
Sample_SPA04.Cluster12575	37.80	Clostridiales	201351	1925637
Sample_SPA04.Cluster88	58.50	Clostridiales	90657	2395575
Sample_SPA04.Cluster13556	37.40	Clostridiales	123096	2720220

Sample_SPA04.Cluster13447cbin.1	38.40	Clostridiales	104682	2699803
Sample_SPA04.mb.102	55.20	Bacteroidetes	50767	2603733
Sample_SPA04.Cluster14222	34.10	Bacilli	5928	2111042
Sample_SPA04.mb.119cbin.1	57.60	Clostridiales	22942	3016864
Sample_SPA04.mb.46	41.20	Clostridiales	13206	2855294
Sample_SPA04.mb.115cbin.1	29.80	Bacteria	66896	2551862
Sample_SPA04.mb.79cbin.1	52.90	Clostridia	72775	2041584
Sample_SPA04.mb.31	63.40	Actinobacteria	64739	2637909
Sample_SPA04.mb.89	53.00	Clostridiales	9684	1754805
Sample_SPA04.mb.71cbin.1	55.10	Clostridiales	70060	2323907
Sample_SPA04.mb.61	51.60	Clostridiales	20979	2081793
Sample_SPA04.mb.29	45.40	Clostridiales	19031	2324228
Sample_SPA04.mb.5	59.10	Clostridiales	26295	2243610
Sample_SPA04.mb.2	58.90	Bacteroidetes	132302	2761286
Sample_SPA04.mb.85	54.00	Actinobacteria	185255	1672639
Sample_SPA04.mb.73cbin.1	56.40	Clostridiales	38587	2541584
Sample_SPA04.mb.26	41.90	Lachnospiraceae	58339	2296107
Sample_SPA04.mb.88cbin.1	48.40	Clostridiales	31380	2719260
Sample_SPA04.mb.57	51.10	Clostridiales	234129	2292229
Sample_SPA04.mb.53cbin.1	59.10	Bacteroidetes	98928	2990673
Sample_SPA04.mb.94	45.00	Clostridiales	39637	2646147
Sample_SPA04.mb.43cbin.1	44.40	Prevotella	16129	3887347
Sample_SPA04.mb.75	42.70	Clostridiales	28000	2226916
Sample_SPA04.mb.64cbin.1	45.10	Bacteroidales	53217	4124134
Sample_SPA06.Cluster13396	26.10	Bacteria	70973	1071112
Sample_SPA06.Cluster2511cbin.1	49.90	Clostridiales	75829	2621544
Sample_SPA06.Cluster6148	41.80	Clostridiales	177579	2361202
Sample_SPA06.Cluster317cbin.1	61.90	Clostridiales	40070	2129530
Sample_SPA06.Cluster781	58.30	Clostridiales	55250	2876475
Sample_SPA06.Cluster4378cbin.1	46.00	Clostridiales	116556	2332381
Sample_SPA06.Cluster178cbin.1	58.60	Clostridiales	118290	2326819
Sample_SPA06.Cluster476cbin.1	58.60	Clostridiales	47543	2317398
Sample_SPA06.Cluster442	59.70	Actinobacteria	95817	2107603
Sample_SPA06.Cluster1690cbin.1	54.20	Clostridiales	185586	1719713
Sample_SPA06.Cluster528cbin.1	58.30	Clostridiales	46102	2440645
Sample_SPA06.mb.117	49.60	Clostridiales	111966	1970950
Sample_SPA06.Cluster2162	52.00	Clostridiales	187915	2237376
Sample_SPA06.Cluster346cbin.1	60.10	Bacteroidetes	44315	2513793
Sample_SPA06.Cluster465cbin.1	63.10	Actinobacteria	193037	2752174
Sample_SPA06.mb.20	26.50	Bacteria	62206	1320385
Sample_SPA06.Cluster341cbin.1	58.60	Clostridiales	68540	2978494
Sample_SPA06.mb.26	27.80	Bacteria	181823	1099964
Sample_SPA06.Cluster12015	36.10	Clostridiales	184689	2477150
Sample_SPA06.mb.119	62.80	Clostridiales	14535	1792169
Sample_SPA06.mb.114cbin.1	48.10	Clostridiales	109264	2930978
Sample_SPA06.Cluster5570cbin.1	42.30	Lachnospiraceae	11769	2173773
Sample_SPA06.Cluster625	58.20	Bacteria	189472	3001991
Sample_SPA06.mb.133	38.40	Clostridiales	33565	2872887
Sample_SPA06.Cluster6549cbin.1	43.70	Clostridiales	161691	2837264
Sample_SPA06.Cluster7016	43.00	Clostridiales	134690	2377918
Sample_SPA06.mb.134cbin.1	61.30	Clostridiales	69259	1622684
Sample_SPA06.Cluster12358	31.30	Firmicutes	29601	2224115
Sample_SPA06.mb.15	62.30	Clostridiales	11507	2262512
Sample_SPA06.mb.70	35.30	Clostridiales	11768	2199822
Sample_SPA06.mb.88cbin.1	42.60	Bacteria	34752	5970849
Sample_SPA06.mb.136cbin.1	49.10	Bacteroidales	51212	3237762
Sample_SPA06.mb.3cbin.1	56.30	Clostridiales	98794	2358418
Sample_SPA06.mb.92	34.10	Clostridiales	90070	2246573

Sample_SPA06.mb.27	47.80	Clostridiales	41658	1927270
Sample_SPA06.mb.5	41.00	Lachnospiraceae	138961	2483925
Sample_SPA06.mb.35	40.90	Clostridiales	10954	1851819
Sample_SPA06.Cluster5434	45.20	Bacteroidales	179583	4196315
Sample_SPA06.Cluster4291cbin.1	45.00	Bacteroidales	116132	4451631
Sample_SPA06.Cluster4973cbin.1	45.80	Lachnospiraceae	78062	5499852
Sample_SPA06.mb.93cbin.1	54.20	Actinobacteria	138347	1674768
Sample_SPA06.mb.95cbin.1	56.80	Clostridiales	62831	2478908
Sample_SPA06.mb.77cbin.1	38.00	Clostridiales	140623	1956589
Sample_SPA06.mb.7cbin.1	56.40	Clostridiales	14891	2415276
Sample_SPA06.mb.72cbin.1	43.60	Lachnospiraceae	72634	2706896
Sample_SPA06.Cluster4990cbin.1	51.20	Enterobacteriaceae	6190	3497519
Sample_SPA06.mb.99cbin.1	60.20	Bifidobacteriaceae	175674	2336489
Sample_SPA06.mb.59cbin.1	48.30	Clostridiales	58424	6216417
Sample_SPA07.Cluster12085	24.20	Bacteria	108132	956466
Sample_SPA07.Cluster12056	26.60	Bacteria	86080	1188498
Sample_SPA07.Cluster7043	43.70	Clostridiales	73366	2668736
Sample_SPA07.Cluster958cbin.1	58.20	Clostridiales	58638	2888024
Sample_SPA07.Cluster3082cbin.1	54.80	Clostridiales	39677	2440889
Sample_SPA07.Cluster544cbin.1	58.70	Clostridiales	77166	2450184
Sample_SPA07.mb.69cbin.1	47.70	Bacteria	9210	1497200
Sample_SPA07.Cluster99cbin.1	61.60	Clostridiales	116865	1989112
Sample_SPA07.Cluster356cbin.1	58.30	Clostridiales	47570	2708558
Sample_SPA07.mb.4	32.60	Clostridiales	132007	3116104
Sample_SPA07.mb.50	61.90	Clostridiales	38250	1561983
Sample_SPA07.mb.46cbin.1	57.10	Clostridiales	16045	2294085
Sample_SPA07.Cluster10244	38.70	Streptococcus	27677	1595473
Sample_SPA07.mb.9	26.30	Bacteria	124936	1132468
Sample_SPA07.Cluster6053cbin.1	44.40	Clostridiales	146081	1928175
Sample_SPA07.mb.54	56.70	Clostridiales	28000	2387532
Sample_SPA07.mb.30cbin.1	46.90	Clostridiales	71031	2614408
Sample_SPA07.mb.32cbin.1	64.10	Actinobacteria	6620	2124463
Sample_SPA07.mb.7cbin.1	37.70	Clostridiales	86483	2961277
Sample_SPA07.mb.37	54.40	Bacteroidetes	99717	2192064
Sample_SPA07.mb.43cbin.1	46.70	Lachnospiraceae	70954	2850255
Sample_SPA07.Cluster10547cbin.1	37.20	Clostridiales	43693	2749403
Sample_SPA07.mb.81cbin.1	58.00	Clostridiales	12715	1984386
Sample_SPA07.mb.31	59.80	Bacteroidetes	14604	2585211
Sample_SPA07.mb.62	29.90	Bacteria	27111	2353384
Sample_SPA07.mb.67	59.10	Bacteroidetes	141162	2966222
Sample_SPA07.Cluster8392cbin.1	40.50	Bacteroidales	52915	3536241
Sample_SPA07.mb.99cbin.1	60.10	Actinobacteria	10140	1820199
Sample_SPA07.mb.20cbin.1	44.30	Bacteroidales	68258	3539272
Sample_SPA07.mb.57	43.60	Bacteroidales	95026	2709803
Sample_SPA07.mb.82	49.80	Lachnospiraceae	82635	2645295
Sample_SPA07.mb.92	44.40	Clostridiales	161445	2628461
Sample_SPA07.Cluster5915cbin.1	45.40	Bacteroidales	109121	4272890
Sample_SPA07.mb.38cbin.1	47.00	Bacteroidales	25927	3823374
Sample_SPA07.mb.98cbin.1	41.30	Lachnospiraceae	91172	2980285
Sample_SPA08.Cluster4973	49.40	Clostridiales	100876	1981446
Sample_SPA08.mb.10	25.50	Bacteria	229447	1400332
Sample_SPA08.mb.105	26.20	Bacteria	56155	1132679
Sample_SPA08.mb.123cbin.1	41.50	Bacteria	72325	3097210
Sample_SPA08.mb.108	55.90	Clostridiales	66166	1856828
Sample_SPA08.Cluster744cbin.1	59.20	Clostridiales	13802	1984403
Sample_SPA08.Cluster60cbin.1	59.00	Clostridiales	28599	2208373
Sample_SPA08.Cluster216cbin.1	57.50	Clostridiales	20110	2711839
Sample_SPA08.Cluster8154	44.90	Actinobacteria	296366	1843477

Sample_SPA08.mb.101cbin.1	60.20	Clostridiales	43959	1943488
Sample_SPA08.mb.28cbin.1	51.50	Bacteria	29061	1822554
Sample_SPA08.mb.42	28.00	Bacteria	24808	1160233
Sample_SPA08.Cluster707cbin.1	55.50	Bacteroidetes	10475	1847902
Sample_SPA08.Cluster10169	40.90	Clostridiales	169190	1951087
Sample_SPA08.Cluster6153	46.40	Bacteroidetes	349055	2374613
Sample_SPA08.Cluster5908	43.50	Selenomonadales	316579	2402720
Sample_SPA08.Cluster6101cbin.1	46.20	Lachnospiraceae	57496	3290288
Sample_SPA08.mb.37	53.50	Clostridiales	14903	1592129
Sample_SPA08.Cluster1878	55.80	Proteobacteria	117173	2198906
Sample_SPA08.mb.119	62.40	Actinobacteria	35957	2100421
Sample_SPA08.mb.52	52.80	Clostridia	70417	2119327
Sample_SPA08.Cluster7637	44.40	Clostridiales	137918	2244946
Sample_SPA08.mb.115	61.00	Clostridiales	21405	2124174
Sample_SPA08.Cluster12217cbin.1	37.30	Clostridiales	59692	2775247
Sample_SPA08.Cluster5941cbin.1	45.40	Prevotella	53143	3301695
Sample_SPA08.mb.72	29.20	Bacteria	135241	1606653
Sample_SPA08.mb.29	53.60	Clostridiales	30538	1970868
Sample_SPA08.mb.13	54.60	Clostridia	135172	2536629
Sample_SPA08.mb.124	61.00	Bacteroidetes	13217	2267158
Sample_SPA08.mb.40	58.20	Clostridiales	34437	2102841
Sample_SPA08.mb.47cbin.1	48.90	Clostridiales	9582	2493059
Sample_SPA08.mb.120	40.90	Lachnospiraceae	52815	2372007
Sample_SPA08.mb.65cbin.1	61.50	Clostridiales	60217	1858675
Sample_SPA08.mb.17	53.40	Clostridiales	189725	1900988
Sample_SPA08.mb.56	56.60	Clostridiales	14479	2185002
Sample_SPA08.Cluster6551cbin.1	43.10	Bacteria	20891	3937276
Sample_SPA08.mb.75cbin.1	51.00	Clostridiales	25786	2299310
Sample_SPA08.mb.83cbin.1	56.40	Clostridiales	53542	2633390
Sample_SPA08.mb.70cbin.1	45.00	Clostridiales	13242	1860431
Sample_SPA08.mb.68	46.20	Clostridiales	27371	2009909
Sample_SPA08.mb.74	51.50	Clostridiales	151753	2117302
Sample_SPA08.mb.14cbin.1	41.90	Lachnospiraceae	69808	2448333
Sample_SPA08.mb.66	44.80	Lachnospiraceae	77768	2556661
Sample_SPA08.mb.64cbin.1	59.50	Clostridiales	18518	2179564
Sample_SPA08.mb.86	63.30	Actinobacteria	68405	2640701
Sample_SPA08.mb.4cbin.1	41.10	Lachnospiraceae	82175	2939584
Sample_SPA08.mb.90cbin.1	38.20	Lactobacillales	133054	1942286
Sample_SPA08.mb.125	46.20	Bacteroidales	191363	4321658
Sample_SPA08.mb.97cbin.1	58.30	Bacteroidetes	165220	3305564
Sample_SPA08.mb.80cbin.1	43.10	Bacteria	20239	4276963
Sample_SPA09.mb.27cbin.1	36.90	Bacteria	80714	3007954
Sample_SPA09.mb.2cbin.1	27.00	Bacteria	46121	2752542
Sample_SPA09.Cluster5439cbin.1	42.30	Clostridiales	8604	2345142
Sample_SPA09.mb.40cbin.1	41.10	Clostridiales	15990	2924725
Sample_SPA09.mb.10cbin.1	52.30	Clostridiales	161299	2188179
Sample_SPA09.mb.90	38.50	Clostridiales	7882	1868209
Sample_SPA09.Cluster6961	42.10	Clostridiales	63289	1734696
Sample_SPA09.Cluster404cbin.1	56.60	Clostridiales	15434	2462654
Sample_SPA09.mb.93cbin.1	37.20	Clostridiales	5950	2042855
Sample_SPA09.mb.63cbin.1	48.30	Clostridiales	23298	2698196
Sample_SPA09.mb.91	38.80	Clostridiales	34035	2656638
Sample_SPA09.Cluster1823	56.30	Bifidobacteriaceae	333591	2170563
Sample_SPA09.mb.58	49.40	Clostridiales	33535	2576988
Sample_SPA09.mb.48	43.30	Lachnospiraceae	38899	2133938
Sample_SPA09.mb.38	60.30	Bifidobacteriaceae	48256	2202543
Sample_SPA09.mb.80cbin.1	41.30	Lachnospiraceae	95990	3362225
Sample_SPA09.mb.67	47.00	Lachnospiraceae	28433	2691712

Sample_SPA09.Cluster10012cbin.1	42.90	Lachnospiraceae	90807	3093254
Sample_SPA09.mb.15cbin.1	41.20	Lachnospiraceae	93660	3000854
Sample_SPA09.mb.39	58.60	Bacteroidetes	201978	2867443
Sample_SPA09.Cluster6494cbin.1	43.40	Bacteroidales	317117	3168248
Sample_SPA09.mb.89	41.20	Clostridiales	70715	2490496
Sample_SPA09.mb.99cbin.1	54.50	Bacteroidetes	127840	2107385
Sample_SPA09.mb.20	46.20	Bacteroidales	161847	3268051
Sample_SPA09.mb.28	42.80	Lachnospiraceae	77792	3310384
Sample_SPA09.mb.88cbin.1	48.50	Proteobacteria	77270	2492999
Sample_SPA09.mb.82cbin.1	45.40	Bacteroidales	23594	4455333
Sample_SPA09.Cluster99cbin.1	50.90	Enterobacteriaceae	50103	4600681
Sample_SPA10.mb.16cbin.1	48.00	Clostridiales	103449	2961963
Sample_SPA10.mb.26	61.40	Clostridiales	80933	2572021
Sample_SPA10.mb.36cbin.1	56.80	Clostridiales	6898	2079446
Sample_SPA10.mb.42	49.00	Clostridiales	49710	2557711
Sample_SPA10.mb.19	38.10	Clostridiales	80428	1979895
Sample_SPA10.mb.53cbin.1	35.60	Clostridiales	13507	4403727
Sample_SPA10.mb.20cbin.1	58.50	Clostridiales	45386	2143247
Sample_SPA10.mb.29cbin.1	43.90	Lachnospiraceae	141523	2573027
Sample_SPA10.mb.61	38.80	Clostridiales	94863	2113999
Sample_SPA10.Cluster4817	35.90	Clostridiales	207491	2593651
Sample_SPA10.mb.48	46.40	Lachnospiraceae	25737	2523366
Sample_SPA10.mb.34	60.30	Deltaproteobacteria	27716	3756092
Sample_SPA10.mb.31cbin.1	43.80	Clostridiales	169245	2907990
Sample_SPA10.mb.55	42.20	Lachnospiraceae	68228	2170133
Sample_SPA10.Cluster1134cbin.1	48.40	Proteobacteria	74358	2585026
Sample_SPA10.mb.51cbin.1	41.70	Lachnospiraceae	83960	2372718
Sample_SPA10.Cluster25cbin.1	64.60	Actinobacteria	15808	3158875
Sample_SPA10.mb.15cbin.1	46.00	Bacteroidales	143586	3486899
Sample_SPA10.mb.18cbin.1	46.40	Bacteroidales	117024	4025242
Sample_SPA10.mb.17cbin.1	44.90	Bacteroidales	181401	4676751
Sample_SPA10.Cluster1582cbin.1	41.80	Bacteroides	28828	4479277
Sample_SPA10.Cluster2898	41.40	Bacteroidales	226243	5092758
Sample_SPA11.Cluster4973cbin.1	43.80	Clostridiales	9757	2591711
Sample_SPA11.Cluster127cbin.1	60.80	Clostridiales	12913	1762744
Sample_SPA11.Cluster6814cbin.1	41.90	Clostridiales	130911	2652117
Sample_SPA11.Cluster5732	45.30	Actinobacteria	180474	1930422
Sample_SPA11.Cluster231cbin.1	59.40	Clostridiales	51368	2182278
Sample_SPA11.Cluster440cbin.1	59.80	Actinobacteria	93951	2016922
Sample_SPA11.Cluster6967cbin.1	48.20	Clostridiales	105696	2802157
Sample_SPA11.Cluster79cbin.1	60.50	Clostridiales	57401	2428596
Sample_SPA11.Cluster12075cbin.1	33.30	Bacteria	21403	2831782
Sample_SPA11.Cluster73cbin.1	58.40	Clostridiales	122066	2503701
Sample_SPA11.Cluster5881cbin.1	46.40	Lachnospiraceae	64291	2939778
Sample_SPA11.Cluster3236	51.50	Clostridiales	217962	2136948
Sample_SPA11.Cluster5039	47.40	Selenomonadales	66931	2166592
Sample_SPA11.Cluster1285	55.60	Proteobacteria	149048	2231820
Sample_SPA11.mb.118	51.80	Clostridiales	20273	2051896
Sample_SPA11.Cluster2524cbin.1	53.30	Clostridiales	110144	2410935
Sample_SPA11.Cluster10740	38.50	Clostridiales	66257	2501321
Sample_SPA11.mb.55cbin.1	58.80	Bacteria	23443	2082026
Sample_SPA11.mb.39cbin.1	59.20	Clostridia	28876	2859572
Sample_SPA11.Cluster9675	40.60	Clostridiales	190116	2027379
Sample_SPA11.mb.12cbin.1	53.40	Clostridiales	8426	2405905
Sample_SPA11.mb.16cbin.1	57.40	Clostridiales	48051	2097515
Sample_SPA11.mb.14	59.20	Clostridiales	18065	2151195
Sample_SPA11.Cluster8660	41.40	Lachnospiraceae	83235	2796615
Sample_SPA11.mb.101cbin.1	37.40	Clostridiales	49624	2640016

Sample_SPA11.mb.78cbin.1	58.40	Clostridiales	42960	2962489
Sample_SPA11.mb.18cbin.1	60.00	Bacteroidetes	37100	2647740
Sample_SPA11.Cluster4412cbin.1	43.30	Bacteria	27894	4017397
Sample_SPA11.Cluster4833cbin.1	44.20	Bacteroidales	73314	3742874
Sample_SPA11.mb.21	44.00	Clostridiales	197213	2836039
Sample_SPA11.mb.111cbin.1	41.70	Lachnospiraceae	75912	2451682
Sample_SPA11.Cluster1799cbin.1	45.70	Bacteroidales	9488	3823449
Sample_SPA11.mb.41	54.40	Bacteroidetes	95059	2098583
Sample_SPA11.Cluster5091	45.40	Bacteroidales	139841	3979989
Sample_SPA11.mb.43	56.40	Clostridiales	11270	2372144
Sample_SPA11.mb.3	43.60	Clostridiales	13145	2799763
Sample_SPA11.mb.37	44.20	Clostridiales	162094	2567120
Sample_SPA11.mb.38cbin.1	42.80	Clostridiales	147603	2649873
Sample_SPA11.mb.93	41.00	Clostridiales	27504	3361947
Sample_SPA11.mb.85	61.90	Clostridiales	33857	2309186
Sample_SPA11.mb.58cbin.1	58.20	Clostridiales	8466	2220916
Sample_SPA11.mb.62	42.30	Streptococcus	29941	1949820
Sample_SPA11.mb.36	43.40	Bacteroidales	296971	3276034
Sample_SPA11.mb.9cbin.1	58.40	Bacteroidetes	140614	2595535
Sample_SPA11.mb.51cbin.1	44.30	Prevotella	14989	3918795
Sample_SPA11.mb.74cbin.1	45.80	Bacteroidales	75321	2958727
Sample_SPA11.mb.50	47.00	Bacteroidales	26769	3697184
Sample_SPA11.mb.92	42.20	Bacteroidales	31381	4065030
Sample_SPA12.Cluster24708cbin.1	26.00	Bacteria	71722	1263517
Sample_SPA12.Cluster11878	43.90	Clostridiales	10792	2569903
Sample_SPA12.Cluster259	61.20	Clostridiales	21370	1480570
Sample_SPA12.mb.127	29.40	Bacteria	35554	1433558
Sample_SPA12.Cluster10362	46.10	Clostridiales	101951	2156629
Sample_SPA12.mb.124cbin.1	49.90	Clostridiales	10315	1808921
Sample_SPA12.Cluster18234cbin.1	49.80	Clostridiales	67426	2264547
Sample_SPA12.Cluster17354cbin.1	41.40	Clostridiales	13186	3611413
Sample_SPA12.mb.101	52.00	Clostridiales	9260	1919430
Sample_SPA12.Cluster23502cbin.1	33.50	Bacteria	25344	2286292
Sample_SPA12.mb.117	37.30	Clostridiales	31513	2428314
Sample_SPA12.Cluster5511cbin.1	51.90	Bacteroidales	17749	2168990
Sample_SPA12.Cluster20418cbin.1	41.90	Clostridiales	118008	2329984
Sample_SPA12.mb.27	52.80	Clostridia	73144	2229291
Sample_SPA12.mb.21cbin.1	45.20	Actinobacteria	10132	1742194
Sample_SPA12.mb.147	44.50	Clostridiales	38254	2799076
Sample_SPA12.Cluster5034	55.60	Bacteria	180588	2682018
Sample_SPA12.mb.109cbin.1	45.90	Clostridiales	19895	2008596
Sample_SPA12.mb.22cbin.1	62.10	Clostridiales	20991	1983940
Sample_SPA12.mb.68cbin.1	45.60	Clostridiales	97059	1783989
Sample_SPA12.mb.73	27.20	Clostridiales	102760	1840883
Sample_SPA12.mb.52cbin.1	48.80	Clostridiales	20808	2559188
Sample_SPA12.mb.31	61.20	Clostridiales	10334	2034590
Sample_SPA12.mb.14cbin.1	38.60	Clostridiales	9096	2488010
Sample_SPA12.mb.89	60.40	Clostridiales	6811	2415327
Sample_SPA12.mb.34cbin.1	51.00	Firmicutes	54269	2215406
Sample_SPA12.mb.95	63.40	Clostridiales	11583	2069009
Sample_SPA12.mb.50	63.60	Actinobacteria	12590	2479741
Sample_SPA12.mb.36cbin.1	50.70	Bacteroidales	140687	2195609
Sample_SPA12.mb.80	56.40	Clostridiales	15050	2711179
Sample_SPA12.mb.90	41.10	Lachnospiraceae	103319	2363469
Sample_SPA12.mb.59	36.00	Clostridiales	19435	2691930
Sample_SPA12.mb.86	47.00	Prevotella	22410	2259126
Sample_SPA12.mb.96	47.80	Selenomonadales	33852	1960975
Sample_SPA12.mb.82	62.60	Proteobacteria	33888	2276277

Sample_SPA12.mb.94	42.80	Clostridiales	92795	2699425
Sample_SPA12.mb.9cbin.1	51.10	Clostridiales	127015	2242536
Sample_SPA12.mb.37	45.40	Bacteroidales	18684	4180507
Sample_SPA12.Cluster1195cbin.1	57.70	Enterobacteriaceae	129534	5137795
Sample_SPA12.mb.65	41.70	Bacteroides	82266	4955000
Sample_SPA13.Cluster985cbin.1	48.20	Clostridia	25964	1924874
Sample_SPA13.Cluster7268cbin.1	35.90	Clostridiales	227738	1983101
Sample_SPA13.Cluster230	60.60	Clostridiales	98380	1759266
Sample_SPA13.mb.38	57.90	Clostridiales	57027	2147556
Sample_SPA13.mb.12	36.70	Clostridiales	106436	2237955
Sample_SPA13.mb.61cbin.1	41.40	Bacteria	13394	6682366
Sample_SPA13.Cluster160	61.70	Actinobacteria	94802	2256520
Sample_SPA13.mb.46	54.40	Actinobacteria	102695	1603387
Sample_SPA13.mb.33	37.70	Clostridiales	49013	4079714
Sample_SPA13.Cluster5892	40.70	Clostridiales	197859	1972793
Sample_SPA13.mb.19cbin.1	43.80	Selenomonadales	18029	2299074
Sample_SPA13.Cluster518	56.20	Bifidobacteriaceae	68909	1922104
Sample_SPA13.Cluster288	59.30	Bifidobacteriaceae	114083	2210647
Sample_SPA13.Cluster281	60.00	Bifidobacteriaceae	216847	2300148
Sample_SPA13.Cluster824	54.20	Proteobacteria	101610	2392064
Sample_SPA13.mb.47cbin.1	31.60	Euryarchaeota	5406	1414765
Sample_SPA13.mb.62cbin.1	60.60	Clostridiales	86478	2449281
Sample_SPA13.Cluster4841	41.30	Lachnospiraceae	66283	2909485
Sample_SPA13.mb.3	58.60	Clostridiales	36628	3097449
Sample_SPA13.Cluster428cbin.1	56.90	Clostridiales	115233	2721915
Sample_SPA13.mb.74cbin.1	37.00	Clostridiales	65714	2852329
Sample_SPA13.mb.6	58.60	Bacteroidetes	152901	2404530
Sample_SPA13.mb.29	60.10	Actinobacteria	149370	3908526
Sample_SPA13.mb.36	49.10	Proteobacteria	12283	2178634
Sample_SPA13.Cluster2414cbin.1	45.80	Bacteroidales	119473	3596788
Sample_SPA13.mb.49	43.50	Clostridiales	8744	3139807
Sample_SPA13.mb.24	41.50	Clostridiales	48677	3153857
Sample_SPA13.Cluster3020	45.30	Bacteroidales	134524	3925165
Sample_SPA13.mb.50	43.30	Bacteroidales	279781	3157228
Sample_SPA13.Cluster2319cbin.1	46.60	Bacteroidales	106078	3961310
Sample_SPA13.mb.67cbin.1	41.70	Clostridiales	126939	3277112
Sample_SPA13.mb.65	64.60	Actinobacteria	17036	2639515
Sample_SPA13.mb.86	43.30	Lachnospiraceae	137804	2950270
Sample_SPA13.mb.69	41.70	Lachnospiraceae	64711	2538058
Sample_SPA13.mb.64cbin.1	45.20	Bacteroidales	131074	4759774
Sample_SPA15.Cluster10137	45.40	Clostridia	84321	1700065
Sample_SPA15.mb.22	52.80	Clostridia	68512	2089464
Sample_SPA15.Cluster344	61.60	Clostridiales	58649	1485345
Sample_SPA15.Cluster227cbin.1	59.10	Deltaproteobacteria	15155	2259092
Sample_SPA15.mb.23	58.40	Clostridiales	18657	1608057
Sample_SPA15.Cluster15391cbin.1	40.80	Clostridiales	10670	2746229
Sample_SPA15.mb.4	49.60	Clostridiales	114522	1953334
Sample_SPA15.mb.29	52.90	Actinobacteria	12471	1612495
Sample_SPA15.mb.102cbin.1	57.50	Clostridiales	11552	2002305
Sample_SPA15.Cluster523	57.50	Clostridiales	270695	1623773
Sample_SPA15.mb.24cbin.1	59.70	Clostridiales	74126	2260009
Sample_SPA15.mb.38	47.80	Clostridiales	23152	2026336
Sample_SPA15.mb.28	59.50	Clostridiales	68534	1998995
Sample_SPA15.mb.59cbin.1	53.70	Clostridia	11223	1666138
Sample_SPA15.Cluster3881	53.10	Clostridiales	208769	2163516
Sample_SPA15.mb.35	56.70	Clostridiales	20804	2089525
Sample_SPA15.Cluster7678cbin.1	46.70	Selenomonadales	57989	2526376
Sample_SPA15.mb.58	60.60	Clostridiales	36201	1861468

Sample_SPA15.mb.54	57.00	Clostridiales	21291	1924193
Sample_SPA15.mb.65	57.10	Clostridiales	77262	2117624
Sample_SPA15.mb.81	58.10	Clostridiales	14279	2238760
Sample_SPA15.mb.91cbin.1	51.90	Bacteroidetes	32591	2990301
Sample_SPA15.mb.63	48.20	Clostridiales	35169	1932216
Sample_SPA15.mb.55	46.00	Clostridiales	10645	2478773
Sample_SPA15.mb.66	51.50	Clostridiales	117060	2107389
Sample_SPA15.mb.71	33.70	Clostridiales	67126	2092438
Sample_SPA15.mb.88	44.20	Clostridiales	180533	2287692
Sample_SPA15.Cluster8224cbin.1	46.60	Bacteroidales	29894	4417384
Sample_SPA15.Cluster10540cbin.1	43.60	Bacteroidales	107193	4991493
Sample_SPA15.mb.13cbin.1	43.40	Bacteroidales	11952	5952366
Sample_SPA15.Cluster1062cbin.1	57.60	Enterobacteriaceae	190343	4991413
Sample_SPA15.mb.69cbin.1	51.00	Enterobacteriaceae	108131	4505189
Sample_SPA16.Cluster3423cbin.1	48.80	Clostridiales	22961	2158973
Sample_SPA16.Cluster10324cbin.1	33.60	Bacteria	49439	2205378
Sample_SPA16.Cluster9401cbin.1	43.40	Clostridiales	42165	2440103
Sample_SPA16.Cluster3843	46.40	Lactobacillales	141994	2000074
Sample_SPA16.Cluster2004cbin.1	50.00	Bacteroidales	39014	2450443
Sample_SPA16.mb.2cbin.1	34.20	Bacilli	46670	2674383
Sample_SPA16.mb.1	59.70	Actinobacteria	48685	2222828
Sample_SPA16.mb.117cbin.1	61.30	Clostridiales	13398	2035044
Sample_SPA16.mb.59	38.30	Clostridiales	33574	3056252
Sample_SPA16.Cluster967cbin.1	57.30	Proteobacteria	26558	1877694
Sample_SPA16.mb.38	47.40	Lachnospiraceae	10502	2375754
Sample_SPA16.mb.46	49.80	Clostridiales	13129	2092297
Sample_SPA16.mb.65cbin.1	35.60	Clostridiales	26265	3724532
Sample_SPA16.mb.42	43.60	Lachnospiraceae	40798	1724910
Sample_SPA16.Cluster6977cbin.1	41.30	Lachnospiraceae	83772	2832669
Sample_SPA16.Cluster9846cbin.1	38.60	Clostridiales	121638	2905305
Sample_SPA16.mb.77	49.30	Clostridiales	20294	2455059
Sample_SPA16.Cluster3151cbin.1	44.20	Bacteroidales	63538	3572094
Sample_SPA16.mb.39cbin.1	36.90	Clostridiales	81881	2954212
Sample_SPA16.mb.54	37.40	Clostridiales	126529	2627665
Sample_SPA16.mb.88	38.30	Clostridiales	9237	1605856
Sample_SPA16.mb.48	26.90	Clostridiales	87245	2691325
Sample_SPA16.mb.14cbin.1	41.30	Lachnospiraceae	17563	3512204
Sample_SPA16.mb.9	59.10	Clostridiales	19190	2559816
Sample_SPA16.mb.69	58.40	Bifidobacteriaceae	155051	2425821
Sample_SPA16.mb.99cbin.1	46.40	Bacillus	27744	3810275
Sample_SPA17.Cluster7053cbin.1	27.00	Bacteria	10800	2715480
Sample_SPA17.mb.52	47.80	Clostridiales	137449	1922881
Sample_SPA17.Cluster449	55.20	Clostridiales	81354	2110201
Sample_SPA17.mb.95cbin.1	46.70	Bacteria	120581	4036741
Sample_SPA17.Cluster1797	46.80	Lactobacillales	44701	1836758
Sample_SPA17.mb.44	48.10	Clostridiales	39628	3015373
Sample_SPA17.mb.17	49.60	Clostridiales	11204	2250227
Sample_SPA17.mb.79	61.80	Clostridiales	11367	1812548
Sample_SPA17.Cluster1537	43.50	Selenomonadales	267595	2339909
Sample_SPA17.mb.41cbin.1	54.60	Bacteroidetes	68858	2208455
Sample_SPA17.mb.3cbin.1	60.00	Actinobacteria	87802	2367105
Sample_SPA17.mb.39cbin.1	59.80	Bifidobacteriaceae	62657	1771397
Sample_SPA17.mb.78cbin.1	48.70	Clostridiales	95376	2633730
Sample_SPA17.mb.36cbin.1	29.40	Bacteria	57089	2284951
Sample_SPA17.mb.24	50.80	Bacteroidales	54483	2143680
Sample_SPA17.Cluster883cbin.1	57.20	Clostridiales	45990	2238151
Sample_SPA17.mb.33cbin.1	60.60	Deltaproteobacteria	7370	3182461
Sample_SPA17.mb.76	43.60	Lachnospiraceae	37086	1841669

Sample_SPA17.Cluster6320	35.90	Clostridiales	328199	2696095
Sample_SPA17.Cluster4831cbin.1	37.30	Clostridiales	21529	2640817
Sample_SPA17.mb.75cbin.1	57.40	Proteobacteria	56709	2379134
Sample_SPA17.mb.98	60.50	Clostridiales	44523	1887009
Sample_SPA17.mb.8cbin.1	32.50	Lactobacillales	224568	1918422
Sample_SPA17.Cluster6508	30.30	Bacteria	79675	3386714
Sample_SPA17.mb.114	41.10	Lachnospiraceae	45267	3540102
Sample_SPA17.mb.88cbin.1	48.60	Proteobacteria	28588	2221676
Sample_SPA17.mb.70	42.40	Bacteroides	56714	3898415
Sample_SPA17.mb.16cbin.1	45.30	Bacteroidales	131656	3981430
Sample_SPA17.mb.108cbin.1	41.80	Bacteroidales	94778	4239342
Sample_SPA17.Cluster2740	44.90	Bacteroidales	263469	4691645
Sample_SPA17.mb.97cbin.1	51.00	Enterobacteriaceae	173514	3725242
Sample_SPA20.mb.23	38.10	Clostridiales	23308	2606020
Sample_SPA20.mb.12cbin.1	37.20	Clostridiales	54872	2745327
Sample_SPA20.mb.36	33.60	Clostridiales	116680	2970189
Sample_SPA20.Cluster2573	46.90	Lactobacillales	121463	1863573
Sample_SPA20.mb.5	34.20	Bacteria	39073	1883296
Sample_SPA20.mb.32cbin.1	59.90	Actinobacteria	49254	2181210
Sample_SPA20.Cluster1446cbin.1	48.60	Firmicutes	32775	1944608
Sample_SPA20.mb.39cbin.1	52.30	Clostridiales	9225	1740734
Sample_SPA20.mb.61cbin.1	48.60	Clostridiales	14045	2681368
Sample_SPA20.Cluster130	60.00	Bifidobacteriaceae	195783	2194529
Sample_SPA20.mb.9	54.00	Clostridiales	71397	1831206
Sample_SPA20.mb.6cbin.1	40.90	Clostridiales	135024	1871842
Sample_SPA20.Cluster6883cbin.1	32.50	Lactobacillales	93959	1892566
Sample_SPA20.Cluster4938cbin.1	40.60	Lachnospiraceae	81319	2596336
Sample_SPA20.mb.24cbin.1	49.00	Proteobacteria	40542	2206204
Sample_SPA20.mb.43	44.50	Clostridiales	67821	2901578
Sample_SPA20.Cluster2412cbin.1	44.00	Bacteroidales	59407	3826577
Sample_SPA21.Cluster2186cbin.1	51.20	Bacteria	19431	1860231
Sample_SPA21.mb.117	27.60	Bacteria	296100	1275942
Sample_SPA21.Cluster11127cbin.1	37.20	Clostridiales	11252	2316297
Sample_SPA21.Cluster18	62.40	Clostridiales	9738	1340107
Sample_SPA21.mb.25	26.70	Bacteria	148895	1326539
Sample_SPA21.mb.113cbin.1	58.10	Clostridiales	49857	2970916
Sample_SPA21.Cluster4618cbin.1	46.30	Clostridiales	42347	2070708
Sample_SPA21.mb.103	57.10	Clostridiales	79333	2164862
Sample_SPA21.Cluster6505cbin.1	42.20	Clostridiales	68131	3592593
Sample_SPA21.Cluster7151cbin.1	45.10	Clostridiales	84684	2474161
Sample_SPA21.mb.65	41.00	Bacteria	17139	3184023
Sample_SPA21.Cluster10395cbin.1	38.70	Clostridiales	94796	2010745
Sample_SPA21.mb.110	46.90	Lachnospiraceae	66396	2836108
Sample_SPA21.Cluster6611	44.20	Clostridiales	159779	2658708
Sample_SPA21.mb.116cbin.1	54.70	Bacteroidetes	63543	2054808
Sample_SPA21.mb.26	60.80	Clostridiales	33139	2204568
Sample_SPA21.Cluster125cbin.1	58.60	Clostridiales	105649	3845425
Sample_SPA21.Cluster7969	41.10	Lachnospiraceae	62809	3004411
Sample_SPA21.mb.70cbin.1	59.60	Clostridiales	23531	1820827
Sample_SPA21.Cluster9049cbin.1	37.50	Clostridiales	100921	2803130
Sample_SPA21.mb.75cbin.1	61.00	Clostridiales	44394	2031201
Sample_SPA21.mb.118cbin.1	41.60	Lachnospiraceae	61315	2869050
Sample_SPA21.mb.11	43.60	Clostridiales	20429	3397106
Sample_SPA21.mb.37cbin.1	58.90	Bacteroidetes	92557	3006221
Sample_SPA21.mb.13cbin.1	49.30	Proteobacteria	57000	2066745
Sample_SPA21.mb.121	44.90	Bacteroidales	11411	2823245
Sample_SPA21.mb.53	29.70	Bacteria	47786	2501128
Sample_SPA21.mb.124	49.30	Lachnospiraceae	95562	3099360

Sample_SPA21.mb.61	44.10	Clostridiales	64644	3000328
Sample_SPA21.mb.76	63.40	Actinobacteria	36625	2677538
Sample_SPA21.mb.84	60.50	Bacteroidetes	18798	2562134
Sample_SPA21.mb.39cbin.1	48.60	Bacteroidales	112383	3398257
Sample_SPA21.mb.79	43.80	Bacteroidales	10908	2515676
Sample_SPA21.mb.2	46.70	Bacteroidales	76036	4000633
Sample_SPA21.mb.114cbin.1	41.50	Bacteroidales	213652	4942244
Sample_SPA21.mb.41cbin.1	45.30	Bacteroidales	42092	4090504
Sample_SPA21.mb.60	45.00	Bacteroidales	146994	4612489
Sample_SPA22.Cluster19998	24.00	Bacteria	63835	976686
Sample_SPA22.Cluster19730	29.60	Bacteria	141689	1246104
Sample_SPA22.Cluster13759	34.90	Bacteria	69807	1486345
Sample_SPA22.Cluster19925	28.40	Bacteria	470066	1672206
Sample_SPA22.Cluster10053	45.20	Clostridiales	154949	1685236
Sample_SPA22.Cluster10703cbin.1	43.80	Clostridiales	24817	1816224
Sample_SPA22.Cluster19293cbin.1	32.20	Bacteria	142830	1843180
Sample_SPA22.mb.10cbin.1	59.20	Bacteria	37679	2344591
Sample_SPA22.Cluster18334cbin.1	42.10	Lachnospiraceae	8002	1772393
Sample_SPA22.mb.108cbin.1	53.70	Clostridia	29574	1834606
Sample_SPA22.mb.21	26.70	Bacteria	114372	1264330
Sample_SPA22.Cluster358cbin.1	59.30	Clostridiales	38319	2186957
Sample_SPA22.mb.37	52.90	Clostridia	31019	1959131
Sample_SPA22.mb.28cbin.1	59.50	Clostridiales	119372	2121908
Sample_SPA22.Cluster926cbin.1	57.10	Clostridiales	95739	1930753
Sample_SPA22.Cluster221cbin.1	59.00	Bacteria	10638	2524815
Sample_SPA22.mb.14cbin.1	62.20	Clostridiales	8763	1420891
Sample_SPA22.Cluster14389cbin.1	37.30	Clostridiales	86303	2392240
Sample_SPA22.mb.8	27.20	Bacteria	116011	1138812
Sample_SPA22.Cluster14174cbin.1	43.40	Lachnospiraceae	21546	2890974
Sample_SPA22.Cluster8559	47.10	Selenomonadales	90692	2236051
Sample_SPA22.mb.4	57.60	Clostridiales	72686	2263071
Sample_SPA22.mb.120	60.70	Clostridiales	26313	2255917
Sample_SPA22.mb.32cbin.1	60.30	Actinobacteria	15198	2084383
Sample_SPA22.mb.36	45.60	Clostridiales	54920	2077862
Sample_SPA22.mb.91	28.00	Clostridiales	51840	1359696
Sample_SPA22.mb.2cbin.1	54.60	Bacteroidetes	80367	2033747
Sample_SPA22.Cluster1203cbin.1	55.30	Proteobacteria	19258	2719121
Sample_SPA22.mb.77	33.30	Clostridiales	165981	2594642
Sample_SPA22.mb.122	44.60	Clostridiales	138546	2298223
Sample_SPA22.mb.58	59.50	Clostridiales	104599	2313118
Sample_SPA22.mb.96	56.20	Clostridiales	11522	2027989
Sample_SPA22.mb.53	50.70	Clostridiales	13327	2529954
Sample_SPA22.mb.82	56.60	Clostridiales	76907	2140493
Sample_SPA22.Cluster14732cbin.1	41.80	Bacteroidales	53964	4727282
Sample_SPA22.mb.84	29.90	Bacteria	11762	2338726
Sample_SPA22.mb.93	51.40	Clostridiales	129010	2176206
Sample_SPA22.mb.98cbin.1	60.20	Bacteroidetes	33016	2336389
Sample_SPA22.mb.41	43.40	Bacteroidales	124265	3173758
Sample_SPA22.mb.110	46.30	Bacteroidales	36659	3749969
Sample_SPA25.Cluster129cbin.1	62.10	Bacteria	13527	1724423
Sample_SPA25.Cluster3919cbin.1	53.00	Clostridia	58781	2103243
Sample_SPA25.Cluster11307	32.80	Bacteria	168813	1838261
Sample_SPA25.Cluster5997	45.10	Actinobacteria	143482	1940661
Sample_SPA25.Cluster1390cbin.1	57.90	Clostridiales	7663	2218341
Sample_SPA25.Cluster3488	49.80	Clostridiales	177106	2076509
Sample_SPA25.mb.57cbin.1	47.20	Clostridiales	22588	2025557
Sample_SPA25.Cluster557	59.50	Actinobacteria	89277	1957392
Sample_SPA25.mb.13cbin.1	61.70	Clostridiales	11425	2149300

Sample_SPA25.mb.30cbin.1	62.10	Clostridiales	59686	1835981
Sample_SPA25.mb.42	57.60	Clostridiales	13060	1955280
Sample_SPA25.mb.68	58.00	Clostridiales	51217	3010250
Sample_SPA25.Cluster9308	38.90	Clostridiales	86935	1905201
Sample_SPA25.Cluster26cbin.1	60.40	Bifidobacteriaceae	7766	1902222
Sample_SPA25.Cluster3311cbin.1	51.40	Clostridiales	176093	2178502
Sample_SPA25.Cluster1972	55.50	Bacteria	183896	2660793
Sample_SPA25.Cluster5883	43.70	Lactobacillales	41411	2033797
Sample_SPA25.mb.7cbin.1	57.80	Clostridiales	49100	2715782
Sample_SPA25.mb.41	60.20	Bacteroidetes	32233	2654590
Sample_SPA25.mb.45cbin.1	35.90	Clostridiales	10280	2326795
Sample_SPA25.mb.23cbin.1	48.30	Lachnospiraceae	72048	6704437
Sample_SPA25.mb.71	63.50	Actinobacteria	39648	2633489
Sample_SPA25.mb.28	41.80	Lachnospiraceae	69236	2262547
Sample_SPA25.mb.86	58.60	Bacteroidetes	256183	2358979
Sample_SPA25.mb.92cbin.1	54.50	Bacteroidetes	97991	2188818
Sample_SPA25.mb.46cbin.1	61.90	Deltaproteobacteria	25397	3242802
Sample_SPA25.Cluster4646cbin.1	46.40	Bacteroidales	150608	3764981
Sample_SPA25.mb.81	41.70	Lachnospiraceae	93806	2512917
Sample_SPA25.mb.6cbin.1	58.40	Bacteroidetes	204544	3132296
Sample_SPA25.mb.99	44.90	Clostridiales	55525	2243153
Sample_SPA25.mb.95	43.40	Bacteroidales	207057	3081719
Sample_SPA26.Cluster15006	26.00	Bacteria	89564	1155319
Sample_SPA26.mb.104	26.60	Bacteria	108281	1318025
Sample_SPA26.Cluster14380cbin.1	32.10	Bacteria	33214	1940008
Sample_SPA26.Cluster6968cbin.1	48.10	Clostridiales	74460	1732252
Sample_SPA26.mb.36	25.20	Bacteria	149859	1262201
Sample_SPA26.Cluster48cbin.1	62.20	Clostridiales	15003	1661160
Sample_SPA26.mb.17	49.40	Clostridiales	75375	1914988
Sample_SPA26.Cluster2066	54.50	Clostridiales	103791	2092136
Sample_SPA26.Cluster2008cbin.1	49.10	Bacteroidales	8368	2313632
Sample_SPA26.mb.133	60.30	Clostridiales	80697	1910379
Sample_SPA26.Cluster4054cbin.1	48.30	Bacteroidales	53876	2466801
Sample_SPA26.mb.20cbin.1	57.00	Clostridiales	44973	1901491
Sample_SPA26.Cluster5890cbin.1	47.10	Proteobacteria	25623	1795808
Sample_SPA26.Cluster4627cbin.1	51.10	Firmicutes	38707	2229738
Sample_SPA26.Cluster1527cbin.1	57.90	Proteobacteria	15181	2101720
Sample_SPA26.mb.130cbin.1	55.40	Clostridiales	22912	3243913
Sample_SPA26.Cluster12075	40.80	Clostridiales	165279	2672824
Sample_SPA26.mb.30	45.10	Clostridiales	47176	2457582
Sample_SPA26.Cluster751	59.40	Bifidobacteriaceae	262553	2157017
Sample_SPA26.mb.27	59.80	Actinobacteria	79935	2250434
Sample_SPA26.Cluster9645	44.40	Clostridiales	180423	2354665
Sample_SPA26.mb.13cbin.1	51.40	Clostridiales	215002	2207154
Sample_SPA26.mb.79	58.10	Clostridia	36708	3127134
Sample_SPA26.mb.50	60.60	Clostridiales	37178	2579865
Sample_SPA26.mb.95	38.60	Clostridiales	5478	1815824
Sample_SPA26.Cluster986	58.80	Bacteroidetes	177259	2784790
Sample_SPA26.mb.23cbin.1	50.90	Bacteroidales	114719	2190235
Sample_SPA26.Cluster10164cbin.1	41.50	Lachnospiraceae	54923	2601045
Sample_SPA26.mb.45	53.00	Clostridiales	250215	1930643
Sample_SPA26.mb.15	44.90	Selenomonadales	120572	2073637
Sample_SPA26.mb.57cbin.1	45.70	Clostridiales	35088	2344427
Sample_SPA26.mb.37	61.90	Proteobacteria	16419	2252418
Sample_SPA26.mb.54	59.80	Bacteroidetes	146084	2421623
Sample_SPA26.mb.83	55.10	Bacteroidetes	42477	1884376
Sample_SPA26.mb.78	47.80	Clostridiales	122667	1861059
Sample_SPA26.mb.80cbin.1	38.60	Clostridiales	70075	2287255

Sample_SPA26.mb.89cbin.1	46.00	Prevotella	13054	2849469
Sample_SPA26.mb.74cbin.1	43.40	Bacteroidales	21257	3053643
Sample_SPA26.mb.59	49.00	Bacteroidales	42155	3335532
Sample_SPA26.mb.90	43.30	Bacteria	33120	3905712
Sample_SPA26.Cluster3042cbin.1	57.50	Enterobacteriaceae	129545	5034037
Sample_SPA27.Cluster2470cbin.1	41.90	Clostridiales	48045	2656072
Sample_SPA27.Cluster1062cbin.1	48.30	Clostridiales	81055	2841220
Sample_SPA27.mb.21cbin.1	61.20	Clostridiales	20139	2497991
Sample_SPA27.mb.27cbin.1	56.70	Clostridiales	6302	2085307
Sample_SPA27.Cluster283	54.90	Bacteroidetes	106277	1980805
Sample_SPA27.Cluster79cbin.1	56.90	Bifidobacteriaceae	8095	1573180
Sample_SPA27.Cluster925cbin.1	44.10	Selenomonadales	12102	2175519
Sample_SPA27.Cluster1827cbin.1	44.00	Clostridiales	92109	3296193
Sample_SPA27.Cluster213cbin.1	57.10	Clostridiales	45453	2434226
Sample_SPA27.Cluster2653cbin.1	41.10	Lachnospiraceae	86525	2988052
Sample_SPA27.Cluster135cbin.1	57.10	Clostridiales	48499	2779142
Sample_SPA27.mb.37	43.50	Clostridiales	11545	3327489
Sample_SPA27.mb.19	41.80	Lachnospiraceae	23755	2361572
Sample_SPA27.Cluster2782cbin.1	40.60	Lachnospiraceae	61295	3600625
Sample_SPA27.Cluster943	44.10	Bacteroidales	90193	3848802
Sample_SPA27.mb.1	45.30	Bacteroidales	83565	3828891
Sample_SPA27.mb.26	41.30	Clostridiales	29403	3758405
Sample_SPA27.Cluster2785cbin.1	41.80	Bacteroidales	43078	3935661
Sample_SPA27.mb.25	45.20	Bacteroidales	57457	4202289
Sample_SPA27.Cluster632cbin.1	51.00	Enterobacteriaceae	108381	4155652
Sample_SPA28.Cluster10584	27.00	Bacteria	125583	1419869
Sample_SPA28.Cluster9686cbin.1	33.40	Clostridiales	16094	1990579
Sample_SPA28.mb.87	55.10	Bacteria	17426	2022510
Sample_SPA28.mb.17cbin.1	61.10	Clostridiales	8364	1596446
Sample_SPA28.mb.61	58.50	Clostridiales	7180	2606906
Sample_SPA28.mb.27	43.30	Clostridiales	40300	2979519
Sample_SPA28.mb.19	58.00	Clostridiales	30672	2177356
Sample_SPA28.mb.57	36.30	Clostridiales	97746	2488731
Sample_SPA28.Cluster5010	45.10	Clostridiales	107887	2594843
Sample_SPA28.mb.77	38.00	Clostridiales	10664	2306720
Sample_SPA28.Cluster35cbin.1	60.90	Clostridiales	6728	2085753
Sample_SPA28.Cluster9763cbin.1	33.50	Bacteria	43832	2247509
Sample_SPA28.mb.51	41.90	Lachnospiraceae	49585	2108701
Sample_SPA28.Cluster3006cbin.1	49.30	Bacteroidales	141255	2865127
Sample_SPA28.Cluster5286	43.90	Clostridiales	256324	2000320
Sample_SPA28.mb.52	46.10	Clostridiales	40794	2151995
Sample_SPA28.mb.20cbin.1	48.50	Firmicutes	29980	1937632
Sample_SPA28.mb.24cbin.1	46.60	Clostridiales	102272	2716039
Sample_SPA28.Cluster8696cbin.1	38.60	Clostridiales	80944	2133615
Sample_SPA28.Cluster117	60.10	Bifidobacteriaceae	17404	2173633
Sample_SPA28.Cluster5599	42.00	Lachnospiraceae	5749	1902476
Sample_SPA28.mb.83cbin.1	48.20	Clostridiales	17063	2938189
Sample_SPA28.Cluster6267cbin.1	43.40	Lachnospiraceae	82860	2953114
Sample_SPA28.Cluster9539	35.90	Clostridiales	193311	2532633
Sample_SPA28.mb.21cbin.1	56.30	Bifidobacteriaceae	52656	2069606
Sample_SPA28.mb.70cbin.1	56.50	Clostridiales	14765	2383421
Sample_SPA28.mb.64	41.20	Clostridiales	337782	2690735
Sample_SPA28.mb.69	44.70	Clostridiales	65228	2397035
Sample_SPA28.mb.8cbin.1	31.60	Firmicutes	32318	2160528
Sample_SPA28.mb.37	47.90	Bacteroidales	15269	3410055
Sample_SPA28.mb.73cbin.1	58.30	Enterobacteriaceae	9746	4307245
Sample_SPA30.Cluster12070	27.60	Bacteria	669807	1263085
Sample_SPA30.mb.13	38.10	Clostridiales	182156	1881398

Sample_SPA30.mb.27	44.10	Clostridiales	63082	2582096
Sample_SPA30.Cluster444cbin.1	58.70	Clostridiales	61004	2069272
Sample_SPA30.mb.100	62.10	Clostridiales	177617	2017670
Sample_SPA30.Cluster87cbin.1	61.10	Clostridiales	47014	2193584
Sample_SPA30.Cluster1140cbin.1	53.20	Clostridiales	40141	2327585
Sample_SPA30.Cluster372cbin.1	60.90	Clostridiales	47770	2281365
Sample_SPA30.mb.41	58.40	Clostridiales	62266	2873253
Sample_SPA30.mb.22	60.70	Clostridiales	63247	1679527
Sample_SPA30.Cluster5439	45.40	Selenomonadales	282348	1947730
Sample_SPA30.Cluster218cbin.1	60.50	Clostridiales	33901	2380106
Sample_SPA30.Cluster6069	43.50	Selenomonadales	267532	2478522
Sample_SPA30.Cluster10669cbin.1	36.10	Clostridiales	5778	2094232
Sample_SPA30.mb.25	58.50	Bacteroidetes	49609	2374424
Sample_SPA30.Cluster371cbin.1	55.70	Bacteria	202516	2750401
Sample_SPA30.mb.102cbin.1	49.70	Clostridiales	105329	2408085
Sample_SPA30.Cluster5550cbin.1	45.20	Prevotella	54790	3274496
Sample_SPA30.Cluster7309cbin.1	42.80	Clostridiales	81823	2751574
Sample_SPA30.Cluster3188cbin.1	55.50	Proteobacteria	13292	2599541
Sample_SPA30.Cluster11505cbin.1	31.30	Firmicutes	47874	2268695
Sample_SPA30.Cluster4953	46.20	Bacteroidales	86774	3336394
Sample_SPA30.mb.63cbin.1	39.30	Clostridiales	71202	1993740
Sample_SPA30.mb.80	45.70	Clostridiales	11529	2125366
Sample_SPA30.mb.50	42.50	Bacteroidales	51034	3641968
Sample_SPA30.Cluster6094	45.40	Bacteroidales	206009	4004383
Sample_SPA30.mb.14cbin.1	42.00	Bacteroidales	68323	4783866
Sample_SPA31.Cluster8294cbin.1	26.00	Bacteria	35677	1230259
Sample_SPA31.mb.47	26.60	Bacteria	60742	1314590
Sample_SPA31.mb.15	59.90	Clostridia	16990	2406300
Sample_SPA31.mb.57cbin.1	53.70	Clostridia	19487	1764351
Sample_SPA31.Cluster1487cbin.1	57.60	Clostridiales	6217	2058962
Sample_SPA31.mb.11cbin.1	57.50	Clostridiales	46510	1851960
Sample_SPA31.mb.74	49.50	Clostridiales	43945	1898322
Sample_SPA31.Cluster403cbin.1	59.80	Actinobacteria	44035	2057372
Sample_SPA31.Cluster110	60.00	Bacteria	39552	2423573
Sample_SPA31.mb.69	61.80	Clostridiales	38365	1651571
Sample_SPA31.Cluster5253cbin.1	43.70	Clostridiales	82220	2069793
Sample_SPA31.Cluster2324cbin.1	51.20	Clostridiales	28594	2018948
Sample_SPA31.mb.40	54.50	Bacteroidetes	58131	2033380
Sample_SPA31.mb.45cbin.1	41.00	Clostridiales	14048	1886177
Sample_SPA31.Cluster4489cbin.1	46.40	Clostridiales	56441	2497483
Sample_SPA31.mb.62	57.20	Clostridiales	53766	2541410
Sample_SPA31.Cluster5882cbin.1	44.40	Clostridiales	142979	2327986
Sample_SPA31.Cluster7476	32.70	Lactobacillales	28095	1944481
Sample_SPA31.Cluster6198	43.00	Clostridiales	109811	2575166
Sample_SPA31.mb.79cbin.1	57.50	Clostridiales	18143	3126127
Sample_SPA31.mb.83	58.60	Bacteroidetes	218153	2261497
Sample_SPA31.mb.2cbin.1	41.50	Lachnospiraceae	86974	2972960
Sample_SPA31.mb.68	58.30	Bacteroidetes	155697	3081482
Sample_SPA31.mb.7cbin.1	37.20	Bacteria	39578	3090226
Sample_SPA31.mb.60cbin.1	48.10	Clostridiales	43785	6408952
Sample_SPA31.Cluster5757	43.00	Bacteroidales	142931	5103494
Sample_SPA31.mb.67	45.00	Bacteroidales	72442	4585176
Sample_SPA31.mb.82cbin.1	50.50	Enterobacteriaceae	142264	4952928
Sample_SPA31.mb.56cbin.1	57.50	Enterobacteriaceae	43715	5157157
Sample_SPA32.mb.10	47.50	Bacteria	60107	1627327
Sample_SPA32.Cluster8317cbin.1	33.50	Clostridiales	351073	2154330
Sample_SPA32.Cluster6554	37.20	Clostridiales	120880	2451398
Sample_SPA32.Cluster5430cbin.1	42.00	Clostridiales	78943	2838312

Sample_SPA32.mb.41cbin.1	37.20	Clostridiales	8725	2276644
Sample_SPA32.Cluster831cbin.1	55.00	Clostridiales	84132	2476258
Sample_SPA32.Cluster3859	45.20	Lachnospiraceae	154767	2213449
Sample_SPA32.Cluster1745	48.80	Clostridiales	84766	2600811
Sample_SPA32.mb.13	40.00	Lachnospiraceae	45164	2580181
Sample_SPA32.mb.59cbin.1	38.40	Clostridiales	25927	2532225
Sample_SPA32.mb.79cbin.1	34.30	Bacilli	14509	2353966
Sample_SPA32.Cluster317cbin.1	58.50	Bifidobacteriaceae	49512	2367668
Sample_SPA32.mb.58cbin.1	49.70	Clostridiales	17314	2734059
Sample_SPA32.mb.80	62.20	Clostridiales	43792	1960763
Sample_SPA32.mb.53	46.90	Lachnospiraceae	64669	3221421
Sample_SPA32.mb.2cbin.1	40.90	Clostridiales	23318	2629890
Sample_SPA32.mb.21	44.90	Clostridiales	70438	2739142
Sample_SPA32.mb.29cbin.1	62.70	Clostridiales	43190	3205824
Sample_SPA32.mb.57	59.60	Bacteroidetes	154602	2793928
Sample_SPA32.mb.67cbin.1	57.80	Proteobacteria	12784	2128602
Sample_SPA32.Cluster37cbin.1	64.60	Actinobacteria	12080	3101466
Sample_SPA32.mb.35	45.10	Clostridiales	47883	3563281
Sample_SPA32.mb.99cbin.1	54.50	Bacteroidetes	179563	2073776
Sample_SPA32.mb.75	41.90	Clostridiales	16123	2877196
Sample_SPA32.mb.92	60.30	Actinobacteria	14716	3550702
Sample_SPA32.mb.55cbin.1	45.00	Bacteroidales	167726	4542900
Sample_SPA34.Cluster14210	26.00	Bacteria	41153	1113366
Sample_SPA34.Cluster14283cbin.1	26.60	Bacteria	82738	1254767
Sample_SPA34.Cluster13844	32.70	Bacteria	141455	1908957
Sample_SPA34.Cluster13142cbin.1	33.40	Clostridiales	21626	2113815
Sample_SPA34.Cluster236cbin.1	61.80	Clostridiales	33662	1710130
Sample_SPA34.Cluster5308cbin.1	48.60	Clostridiales	19831	2599641
Sample_SPA34.Cluster10977cbin.1	41.10	Clostridiales	24120	3485799
Sample_SPA34.mb.100	57.00	Clostridiales	78299	1981110
Sample_SPA34.Cluster2863	54.70	Bacteroidetes	147329	1926949
Sample_SPA34.mb.104	58.60	Clostridiales	40905	2074494
Sample_SPA34.Cluster3950cbin.1	53.70	Clostridiales	148801	1912281
Sample_SPA34.mb.10cbin.1	57.60	Clostridiales	90884	2602322
Sample_SPA34.mb.27cbin.1	48.10	Clostridiales	28637	1968618
Sample_SPA34.Cluster1738cbin.1	56.10	Proteobacteria	16235	2026869
Sample_SPA34.mb.47	49.50	Clostridiales	8760	1696969
Sample_SPA34.Cluster2567	55.50	Bacteria	117958	2769751
Sample_SPA34.mb.57cbin.1	53.60	Clostridia	19356	1838666
Sample_SPA34.Cluster8839	44.50	Clostridiales	205321	2590812
Sample_SPA34.mb.106cbin.1	44.60	Clostridiales	7073	1911250
Sample_SPA34.Cluster10225	41.70	Lachnospiraceae	104159	2510340
Sample_SPA34.mb.105cbin.1	39.50	Clostridiales	95811	2003690
Sample_SPA34.mb.113	37.90	Clostridiales	166419	1811794
Sample_SPA34.mb.40	59.80	Clostridiales	62317	2310987
Sample_SPA34.mb.21	52.70	Firmicutes	13015	1956650
Sample_SPA34.Cluster17cbin.1	64.40	Actinobacteria	10914	2976498
Sample_SPA34.mb.55	32.20	Euryarchaeota	6035	1241499
Sample_SPA34.mb.62	61.30	Clostridiales	21379	1969649
Sample_SPA34.mb.102	59.30	Bacteroidetes	196370	2811224
Sample_SPA34.mb.93	59.30	Clostridiales	86756	1930968
Sample_SPA34.mb.53	60.40	Clostridiales	25327	2358707
Sample_SPA34.mb.94cbin.1	58.10	Clostridiales	36111	3058801
Sample_SPA34.mb.83	53.80	Clostridiales	17053	1801481
Sample_SPA34.mb.72cbin.1	55.60	Clostridiales	43990	2637640
Sample_SPA34.Cluster4722cbin.1	45.60	Bacteroidales	16189	3954405
Sample_SPA34.Cluster6895cbin.1	46.40	Bacteroidales	127079	4185940
Sample_SPA34.mb.79	50.00	Clostridiales	11024	1598422

Sample_SPA34.Cluster8898cbin.1	42.10	Bacteroidales	43518	4144680
Sample_SPA34.mb.90cbin.1	46.00	Lachnospiraceae	63997	3084305
Sample_SPA34.mb.92	44.20	Clostridiales	84011	2827970
Sample_SPA34.mb.97	46.10	Clostridiales	24035	2523493
Sample_SPA34.mb.56cbin.1	43.30	Bacteria	61492	4377478
Sample_SPA34.mb.61cbin.1	41.40	Bacteroidales	141982	4907501
Sample_SPA34.Cluster1818	55.10	Enterobacteriaceae	222287	5674361
Sample_SPA35.Cluster18259	25.30	Bacteria	55047	1291561
Sample_SPA35.Cluster17834cbin.1	28.10	Clostridiales	96193	1371900
Sample_SPA35.Cluster14601	37.00	Clostridiales	101975	2539597
Sample_SPA35.Cluster14221cbin.1	42.00	Clostridiales	24079	2217781
Sample_SPA35.mb.100cbin.1	54.00	Clostridia	10450	1702554
Sample_SPA35.Cluster9708	46.60	Clostridiales	269483	1534256
Sample_SPA35.mb.113	51.60	Bacteria	14843	1722060
Sample_SPA35.Cluster432	61.10	Clostridiales	20253	1465161
Sample_SPA35.mb.10	37.50	Clostridiales	72580	1953254
Sample_SPA35.mb.32	49.70	Clostridia	15495	1300436
Sample_SPA35.mb.22	51.30	Clostridia	114940	1757649
Sample_SPA35.Cluster16310cbin.1	33.40	Bacteria	8330	1992879
Sample_SPA35.mb.103cbin.1	59.80	Clostridiales	16301	2264644
Sample_SPA35.mb.47	45.50	Clostridia	129867	1701670
Sample_SPA35.Cluster13092	40.70	Clostridiales	126103	1890979
Sample_SPA35.Cluster1826cbin.1	54.00	Prevotella	72319	2501628
Sample_SPA35.mb.66	26.70	Bacteria	16124	1324541
Sample_SPA35.mb.46	58.60	Clostridia	8275	2937177
Sample_SPA35.Cluster4290cbin.1	51.90	Clostridiales	16004	2034133
Sample_SPA35.mb.50	56.90	Clostridiales	7136	2417858
Sample_SPA35.Cluster3665	55.60	Bacteria	134847	2694577
Sample_SPA35.mb.65cbin.1	52.80	Clostridiales	6513	1872846
Sample_SPA35.mb.71cbin.1	50.70	Clostridiales	76275	1627088
Sample_SPA35.mb.102cbin.1	40.60	Clostridiales	80266	2722622
Sample_SPA35.mb.30	59.80	Actinobacteria	55529	2143999
Sample_SPA35.mb.40cbin.1	58.00	Clostridiales	23517	2130964
Sample_SPA35.mb.49	57.10	Clostridiales	47967	2448756
Sample_SPA35.mb.58	53.60	Clostridiales	12007	1834897
Sample_SPA35.mb.23	30.90	Euryarchaeota	105049	1765641
Sample_SPA35.mb.3cbin.1	41.70	Clostridiales	23478	1512710
Sample_SPA35.mb.25	44.30	Clostridiales	21053	2579650
Sample_SPA35.mb.75cbin.1	49.30	Clostridiales	9283	2249215
Sample_SPA35.mb.114cbin.1	36.10	Clostridiales	30131	2495389
Sample_SPA35.mb.59	60.50	Clostridiales	95773	2498279
Sample_SPA35.mb.73	60.90	Clostridiales	7488	2225068
Sample_SPA35.mb.60cbin.1	59.50	Bifidobacteriaceae	381267	2166574
Sample_SPA35.mb.87	46.50	Clostridiales	9037	1889253
Sample_SPA35.mb.93	54.50	Proteobacteria	38400	2179732
Sample_SPA35.mb.98cbin.1	59.90	Bacteroidetes	6340	2619122
Sample_SPA35.Cluster1466	57.50	Enterobacteriaceae	258187	5119623
Sample_SPA36.Cluster12603	28.50	Bacteria	306479	1685675
Sample_SPA36.mb.2	53.40	Clostridia	42934	1934928
Sample_SPA36.Cluster108	61.90	Clostridiales	143640	1766752
Sample_SPA36.mb.40	34.40	Clostridiales	25659	1996685
Sample_SPA36.Cluster1221cbin.1	57.10	Clostridiales	33983	1998370
Sample_SPA36.mb.19cbin.1	49.00	Clostridiales	6517	2239662
Sample_SPA36.Cluster1524cbin.1	57.70	Clostridiales	16840	1871950
Sample_SPA36.Cluster1295cbin.1	56.60	Clostridiales	51178	2478429
Sample_SPA36.Cluster5207cbin.1	42.00	Lachnospiraceae	31120	1991685
Sample_SPA36.mb.61	58.60	Clostridiales	8358	2590279
Sample_SPA36.mb.20	62.80	Clostridiales	19036	1861382

Sample_SPA36.Cluster6225cbin.1	45.90	Clostridiales	45926	2080602
Sample_SPA36.mb.13cbin.1	52.30	Clostridiales	86117	2258398
Sample_SPA36.Cluster8708cbin.1	44.90	Clostridiales	76405	2783342
Sample_SPA36.Cluster10796	38.20	Clostridiales	216611	1824821
Sample_SPA36.Cluster5539cbin.1	43.40	Lachnospiraceae	18962	2182120
Sample_SPA36.Cluster12513	31.10	Euryarchaeota	171206	1750944
Sample_SPA36.Cluster3422	52.80	Clostridiales	149474	2279671
Sample_SPA36.mb.44cbin.1	60.00	Clostridiales	106538	3215882
Sample_SPA36.Cluster9710	40.60	Clostridiales	172210	2159863
Sample_SPA36.mb.62	59.00	Bacteroidetes	5828	2157321
Sample_SPA36.Cluster41	60.20	Bifidobacteriaceae	143226	2315451
Sample_SPA36.mb.31	41.20	Clostridiales	193440	2569164
Sample_SPA36.mb.28cbin.1	51.00	Clostridiales	263620	2250977
Sample_SPA36.mb.18cbin.1	44.90	Clostridiales	47715	2789678
Sample_SPA36.mb.27	57.80	Bacteria	130106	3077734
Sample_SPA36.mb.36	44.40	Clostridiales	207336	2650383
Sample_SPA36.Cluster10253cbin.1	44.80	Prevotella	60293	3655228
Sample_SPA36.Cluster1302cbin.1	56.10	Clostridiales	17343	2725913
Sample_SPA36.mb.43	57.90	Clostridiales	28816	2128960
Sample_SPA36.mb.7cbin.1	33.30	Bacteria	10297	2238279
Sample_SPA36.mb.68	46.90	Selenomonadales	68917	2254505
Sample_SPA36.mb.83cbin.1	31.30	Firmicutes	63917	2209952
Sample_SPA36.mb.81	45.00	Bacteroidales	78786	4650896
Sample_SPA37.Cluster17704	26.10	Bacteria	58011	1155474
Sample_SPA37.Cluster17805	25.20	Bacteria	121525	1263841
Sample_SPA37.mb.113	25.60	Bacteria	135499	1234695
Sample_SPA37.mb.2	53.20	Clostridia	95066	1930303
Sample_SPA37.mb.41	26.40	Bacteria	52722	1163628
Sample_SPA37.mb.25	49.50	Clostridiales	74437	1935731
Sample_SPA37.Cluster340cbin.1	59.50	Clostridiales	8386	2004514
Sample_SPA37.mb.32	45.10	Clostridiales	132540	1749515
Sample_SPA37.Cluster3961	54.70	Bacteroidetes	76826	2070463
Sample_SPA37.Cluster15419cbin.1	38.90	Clostridiales	65284	2083064
Sample_SPA37.mb.78cbin.1	53.80	Clostridia	12810	1655664
Sample_SPA37.Cluster6548	52.80	Clostridiales	140924	2254914
Sample_SPA37.mb.16cbin.1	48.50	Firmicutes	32960	1792667
Sample_SPA37.Cluster144cbin.1	58.70	Clostridiales	8397	2173746
Sample_SPA37.Cluster11952cbin.1	41.60	Lachnospiraceae	7369	2479318
Sample_SPA37.Cluster18	63.30	Deltaproteobacteria	10162	2382714
Sample_SPA37.mb.61	56.60	Clostridiales	55538	2376322
Sample_SPA37.mb.69	37.90	Clostridiales	49875	3453619
Sample_SPA37.mb.1	44.70	Clostridiales	18473	3055462
Sample_SPA37.Cluster6932cbin.1	50.00	Clostridiales	232233	5500404
Sample_SPA37.mb.40	37.50	Clostridiales	132372	2768859
Sample_SPA37.mb.30	59.00	Bacteroidetes	136387	2801498
Sample_SPA37.mb.108cbin.1	41.60	Lachnospiraceae	62520	2601171
Sample_SPA37.mb.8	59.10	Bacteroidetes	14646	2451438
Sample_SPA37.Cluster8131cbin.1	42.20	Bacteria	5702	4082404
Sample_SPA37.mb.60cbin.1	40.80	Lachnospiraceae	63471	3371928
Sample_SPA37.mb.72	60.00	Deltaproteobacteria	21380	4091254
Sample_SPA37.mb.75	43.70	Bacteroidales	117422	3013339
Sample_SPA37.Cluster15692cbin.1	42.00	Bacteroides	106952	4931329
Sample_SPA37.mb.11cbin.1	44.80	Bacteroidales	15777	5242596
Sample_SPA37.mb.94	50.70	Enterobacteriaceae	112333	4655427
Sample_SPA38.Cluster1699	57.20	Clostridiales	29165	1904290
Sample_SPA38.Cluster605	60.60	Clostridiales	59460	1790279
Sample_SPA38.Cluster9283cbin.1	41.10	Clostridiales	23012	3200440
Sample_SPA38.Cluster9cbin.1	59.60	Clostridiales	9860	2010368

Sample_SPA38.Cluster11102	40.60	Clostridiales	227443	2023306
Sample_SPA38.Cluster7150	43.50	Selenomonadales	132582	2370209
Sample_SPA38.Cluster647cbin.1	56.40	Clostridiales	13764	2545059
Sample_SPA38.Cluster3464	54.70	Bacteroidetes	203576	2868338
Sample_SPA38.mb.113	60.00	Actinobacteria	37345	2152146
Sample_SPA38.Cluster14202cbin.1	31.60	Firmicutes	19579	2008573
Sample_SPA38.mb.101cbin.1	38.90	Clostridiales	70916	2228696
Sample_SPA38.mb.26cbin.1	48.50	Clostridiales	22627	2608876
Sample_SPA38.mb.133	54.60	Bacteroidetes	91325	1935582
Sample_SPA38.Cluster11496cbin.1	41.90	Lachnospiraceae	10031	2856654
Sample_SPA38.mb.134	49.60	Clostridiales	174728	1855382
Sample_SPA38.Cluster8789cbin.1	41.80	Lachnospiraceae	26222	2566845
Sample_SPA38.mb.3cbin.1	59.90	Clostridiales	48704	1896787
Sample_SPA38.mb.57cbin.1	54.80	Actinobacteria	13477	1448906
Sample_SPA38.mb.88	47.10	Bacteria	50435	1753252
Sample_SPA38.Cluster5590cbin.1	48.80	Bacteroidales	114282	3339910
Sample_SPA38.mb.38	59.70	Bacteroidetes	150781	2690304
Sample_SPA38.mb.71cbin.1	61.90	Clostridiales	37028	1949409
Sample_SPA38.mb.103cbin.1	41.10	Lachnospiraceae	79756	2850924
Sample_SPA38.mb.66	61.30	Clostridiales	14326	2035681
Sample_SPA38.mb.70	38.30	Clostridiales	8575	1615389
Sample_SPA38.mb.40cbin.1	42.80	Bacteroidales	71668	2482343
Sample_SPA38.mb.87cbin.1	55.70	Clostridiales	58829	2884890
Sample_SPA38.mb.31cbin.1	43.30	Bacteroidales	290315	3257656
Sample_SPA38.mb.111cbin.1	45.20	Bacteroidales	94758	4186124
Sample_SPA38.mb.91	38.70	Bacteroidales	146273	4265340
Sample_SPA38.mb.74cbin.1	56.50	Bifidobacteriaceae	81449	2060387
Sample_SPA38.mb.97cbin.1	63.50	Actinobacteria	96933	2594464
Sample_SPA38.mb.124cbin.1	45.10	Bacteroidales	54725	4581078
Sample_SPA38.mb.80cbin.1	44.80	Bacteroidales	19919	2528653
Sample_SPA38.mb.41cbin.1	46.30	Bacteroidales	119451	3637197
Sample_SPA38.mb.64	42.20	Bacteroidales	31729	3695191
Sample_SPA39.mb.115	26.70	Bacteria	79873	1304752
Sample_SPA39.Cluster11734cbin.1	33.70	Clostridiales	93373	2146065
Sample_SPA39.Cluster251cbin.1	60.50	Clostridiales	122253	1944069
Sample_SPA39.mb.108	57.80	Bacteria	9752	1630901
Sample_SPA39.Cluster4992	47.80	Clostridiales	146711	2894877
Sample_SPA39.Cluster569cbin.1	58.50	Clostridiales	68307	2273555
Sample_SPA39.mb.134	58.40	Clostridiales	55952	2948357
Sample_SPA39.Cluster11372cbin.1	33.30	Bacteria	42796	2352002
Sample_SPA39.mb.135	61.20	Bacteria	22760	2877403
Sample_SPA39.Cluster1834cbin.1	54.30	Bacteroidetes	74264	2157955
Sample_SPA39.Cluster8588cbin.1	43.70	Selenomonadales	27062	2327861
Sample_SPA39.mb.12	60.20	Actinobacteria	48767	1971041
Sample_SPA39.Cluster3838	51.30	Clostridiales	225998	2224316
Sample_SPA39.Cluster11266	32.50	Lactobacillales	21281	1835358
Sample_SPA39.mb.30cbin.1	54.80	Bacteroidetes	140483	2388844
Sample_SPA39.Cluster6116cbin.1	45.30	Clostridiales	165476	2230103
Sample_SPA39.Cluster65cbin.1	59.90	Bifidobacteriaceae	48625	2033688
Sample_SPA39.mb.102cbin.1	48.30	Firmicutes	32691	2107846
Sample_SPA39.mb.29	58.20	Bacteria	32111	2352502
Sample_SPA39.Cluster366	59.90	Proteobacteria	97883	2454915
Sample_SPA39.mb.100cbin.1	59.90	Bifidobacteriaceae	34990	2231844
Sample_SPA39.mb.27	62.20	Actinobacteria	170969	2120110
Sample_SPA39.Cluster2121	53.20	Selenomonadales	111045	2289189
Sample_SPA39.mb.119	58.90	Bacteroidetes	148902	2741332
Sample_SPA39.mb.58cbin.1	38.40	Clostridiales	15831	2443129
Sample_SPA39.mb.33	41.20	Clostridiales	9022	3165183

Sample_SPA39.mb.128cbin.1	56.50	Bifidobacteriaceae	92709	2056379
Sample_SPA39.Cluster8650cbin.1	41.00	Lachnospiraceae	59258	2964132
Sample_SPA39.mb.136	44.00	Clostridiales	149104	2736982
Sample_SPA39.mb.46cbin.1	51.10	Clostridiales	26456	2283604
Sample_SPA39.mb.113	62.30	Clostridiales	39677	3419344
Sample_SPA39.mb.47	61.40	Clostridiales	109229	2579567
Sample_SPA39.mb.64	57.30	Clostridiales	13289	2171422
Sample_SPA39.mb.6	57.70	Clostridiales	76389	2406145
Sample_SPA39.mb.49cbin.1	56.20	Clostridiales	15621	2453860
Sample_SPA39.mb.122cbin.1	43.40	Bacteria	9782	3715419
Sample_SPA39.Cluster4019	48.80	Clostridiales	95793	6994033
Sample_SPA39.mb.40	43.20	Clostridiales	110943	2487816
Sample_SPA39.Cluster6486cbin.1	45.20	Bacteroidales	140248	4017448
Sample_SPA39.mb.68	59.90	Bacteroidetes	93195	2027921
Sample_SPA39.mb.81	54.50	Clostridia	58026	2445255
Sample_SPA39.Cluster6261	41.90	Bacteroidales	89459	4608356
Sample_SPA39.mb.89cbin.1	49.10	Bacteroidales	121713	3149785
Sample_SPA39.mb.80cbin.1	31.50	Firmicutes	76135	2301425
Sample_SPA39.mb.99cbin.1	58.70	Bacteroidetes	159104	2795355
Sample_SPA39.mb.96	44.40	Bacteroidales	63449	3091925
Sample_SPA39.mb.34cbin.1	44.70	Bacteroidales	73275	5067394
Sample_SPA41.Cluster6557cbin.1	48.00	Clostridia	248209	1688546
Sample_SPA41.Cluster455cbin.1	53.50	Clostridia	58505	1793898
Sample_SPA41.Cluster5568	49.60	Clostridiales	101964	1990909
Sample_SPA41.mb.21	53.50	Clostridiales	25153	1941098
Sample_SPA41.mb.11	59.80	Clostridia	17108	2523798
Sample_SPA41.mb.25cbin.1	53.00	Clostridia	53291	2049900
Sample_SPA41.Cluster9759	37.50	Clostridiales	152694	2176565
Sample_SPA41.Cluster11858	27.30	Euryarchaeota	14985	1645488
Sample_SPA41.Cluster519	60.80	Clostridiales	78410	1784109
Sample_SPA41.mb.12	57.40	Clostridiales	12777	1832199
Sample_SPA41.Cluster10261cbin.1	33.30	Bacteria	50244	2246219
Sample_SPA41.Cluster6650cbin.1	46.70	Lactobacillales	191845	2021598
Sample_SPA41.Cluster395cbin.1	56.20	Clostridiales	10020	2671950
Sample_SPA41.Cluster11436	31.80	Euryarchaeota	54937	1578345
Sample_SPA41.Cluster135cbin.1	60.40	Bacteria	9487	2191400
Sample_SPA41.Cluster8883cbin.1	39.20	Streptococcus	10958	1757858
Sample_SPA41.mb.28	46.30	Lachnospiraceae	98841	2576667
Sample_SPA41.mb.64	58.60	Clostridiales	6308	2441952
Sample_SPA41.Cluster10322cbin.1	32.50	Lactobacillales	34033	1818763
Sample_SPA41.mb.40	60.30	Actinobacteria	16177	1887628
Sample_SPA41.mb.52	60.40	Clostridiales	15818	1813753
Sample_SPA41.Cluster9423	38.60	Streptococcus	126855	1819711
Sample_SPA41.Cluster9689	36.50	Clostridiales	138982	2388848
Sample_SPA41.Cluster334cbin.1	58.40	Bifidobacteriaceae	148013	2358325
Sample_SPA41.mb.41	59.10	Clostridiales	8103	1863544
Sample_SPA41.mb.110cbin.1	57.60	Clostridiales	59894	2768547
Sample_SPA41.mb.50cbin.1	40.90	Clostridiales	25898	1875087
Sample_SPA41.mb.88	51.50	Bacteroidetes	49507	2897420
Sample_SPA41.mb.62cbin.1	46.00	Clostridiales	21196	1870513
Sample_SPA41.mb.51cbin.1	50.00	Clostridiales	57904	3003820
Sample_SPA41.mb.55cbin.1	59.80	Clostridiales	14088	3250676
Sample_SPA41.mb.7cbin.1	51.30	Clostridiales	124949	2284584
Sample_SPA41.mb.92cbin.1	60.20	Bifidobacteriaceae	7603	1882961
Sample_SPA41.mb.71	41.90	Lachnospiraceae	56012	2543783
Sample_SPA41.Cluster5952	41.70	Bacteroidales	58078	4670518
Sample_SPA41.Cluster7247	43.00	Bacteroidales	273843	4972160
Sample_SPA41.Cluster4243cbin.1	50.40	Enterobacteriaceae	86821	4997531

Sample_SPA42.mb.113	51.00	Clostridia	18611	1543590
Sample_SPA42.mb.44	59.30	Clostridia	7066	2013086
Sample_SPA42.Cluster102cbin.1	61.90	Clostridiales	27164	1678088
Sample_SPA42.Cluster500	59.50	Clostridiales	24074	2080168
Sample_SPA42.Cluster5135cbin.1	49.40	Clostridiales	84480	2276560
Sample_SPA42.Cluster16495cbin.1	34.00	Bacteria	23100	2263009
Sample_SPA42.mb.23	51.30	Clostridiales	8243	1938031
Sample_SPA42.Cluster5583cbin.1	49.30	Bacteroidales	123177	3020019
Sample_SPA42.mb.32cbin.1	59.40	Bacteria	110794	5445828
Sample_SPA42.Cluster10090	44.50	Clostridiales	158428	2218082
Sample_SPA42.mb.75cbin.1	52.80	Clostridia	60235	2003999
Sample_SPA42.mb.107cbin.1	40.70	Clostridiales	66399	2097095
Sample_SPA42.mb.72cbin.1	48.90	Bacteria	43701	1996587
Sample_SPA42.mb.29cbin.1	44.70	Prevotella	14799	3198091
Sample_SPA42.Cluster634	60.40	Bifidobacteriaceae	100380	1917649
Sample_SPA42.Cluster9771cbin.1	42.30	Lachnospiraceae	6562	2009313
Sample_SPA42.mb.82cbin.1	61.30	Clostridiales	86182	1981682
Sample_SPA42.mb.53cbin.1	46.00	Clostridiales	53731	2203722
Sample_SPA42.mb.4cbin.1	38.50	Selenomonadales	7860	1799135
Sample_SPA42.mb.33cbin.1	62.00	Proteobacteria	47312	2608050
Sample_SPA42.mb.64cbin.1	41.40	Clostridiales	18634	2284034
Sample_SPA42.mb.73cbin.1	51.60	Clostridiales	37873	2011430
Sample_SPA42.mb.74	54.40	Bacteroidetes	122663	2247906
Sample_SPA42.Cluster10643cbin.1	44.80	Bacteroidales	21489	3651414
Sample_SPA42.mb.88cbin.1	59.70	Bacteroidetes	81909	3002207
Sample_SPA42.mb.85	42.10	Lachnospiraceae	46725	2465641
Sample_SPA42.mb.7	43.40	Bacteroidales	449355	3217693
Sample_SPA42.mb.78cbin.1	42.30	Bacteria	5836	2781298
Sample_SPA42.mb.77	47.40	Bacteroidales	12138	3231628
Sample_SPA45.Cluster4144	51.40	Clostridiales	194974	1869529
Sample_SPA45.Cluster5872	45.90	Clostridiales	56886	2048429
Sample_SPA45.Cluster669cbin.1	58.30	Clostridiales	61980	2716864
Sample_SPA45.mb.122cbin.1	44.90	Clostridiales	30322	2339628
Sample_SPA45.mb.18cbin.1	55.20	Clostridiales	34338	2382037
Sample_SPA45.Cluster12792cbin.1	27.50	Clostridiales	6298	2201757
Sample_SPA45.mb.22cbin.1	57.00	Clostridiales	68157	1916403
Sample_SPA45.Cluster7259cbin.1	43.40	Lachnospiraceae	39010	1977123
Sample_SPA45.Cluster9319	40.90	Clostridiales	208213	1972759
Sample_SPA45.mb.105cbin.1	64.30	Deltaproteobacteria	10655	2297870
Sample_SPA45.mb.26	61.50	Actinobacteria	73878	2060642
Sample_SPA45.Cluster5932cbin.1	45.00	Prevotella	71162	3631918
Sample_SPA45.Cluster10018cbin.1	41.10	Lachnospiraceae	21704	2917341
Sample_SPA45.mb.45cbin.1	62.30	Clostridiales	146856	1863698
Sample_SPA45.Cluster8060cbin.1	43.60	Bacteroidales	9673	2425989
Sample_SPA45.mb.117cbin.1	41.20	Lachnospiraceae	29035	2397781
Sample_SPA45.mb.67	58.20	Clostridiales	65141	3015674
Sample_SPA45.mb.56	41.20	Clostridiales	31140	3234883
Sample_SPA45.mb.101	41.90	Lachnospiraceae	61178	2203925
Sample_SPA45.mb.65	42.30	Clostridiales	50290	2493294
Sample_SPA45.mb.23cbin.1	57.80	Clostridiales	16173	2210425
Sample_SPA45.mb.86	34.20	Clostridiales	29910	2082036
Sample_SPA45.mb.70	62.60	Clostridiales	5285	1991425
Sample_SPA45.mb.59cbin.1	55.60	Prevotella	10958	2158398
Sample_SPA45.mb.8cbin.1	38.00	Clostridiales	118907	2833587
Sample_SPA45.mb.48cbin.1	44.50	Clostridiales	140702	2542248
Sample_SPA45.mb.71cbin.1	48.40	Clostridiales	53962	2791740
Sample_SPA45.Cluster3337cbin.1	49.20	Clostridiales	18251	7019190
Sample_SPA45.mb.37	43.70	Lachnospiraceae	127987	2756900

Sample_SPA45.Cluster5564	45.50	Bacteroidales	61779	4038771
Sample_SPA45.mb.73cbin.1	59.80	Actinobacteria	70725	2200149
Sample_SPA45.mb.93	51.30	Bacteroidetes	89110	3595644
Sample_SPA45.mb.104cbin.1	46.60	Bacteroidales	63625	4074563
Sample_SPA45.mb.85cbin.1	47.30	Selenomonadales	36679	2194350
Sample_SPA45.mb.82cbin.1	31.30	Euryarchaeota	14334	1623014
Sample_SPA45.mb.72	36.00	Clostridiales	48059	2473513
Sample_SPA45.mb.90	59.90	Clostridiales	59428	2677892
Sample_SPA45.mb.98cbin.1	63.80	Actinobacteria	18187	2452172
Sample_SPA45.mb.91	29.20	Bacteria	44836	2524346
Sample_SPA45.mb.87	43.90	Bacteroidales	8532	2483587
Sample_SPA45.mb.61	49.00	Bacteroidales	15201	3304913
Sample_SPA45.mb.5cbin.1	44.70	Bacteroidales	80148	4401238
Sample_SPA45.mb.95cbin.1	50.50	Enterobacteriaceae	127113	4749855
Sample_SPA47.Cluster11721	26.00	Bacteria	101404	1222202
Sample_SPA47.Cluster4099	50.00	Bacteria	543477	1480694
Sample_SPA47.Cluster10668cbin.1	36.80	Clostridiales	14810	2948507
Sample_SPA47.Cluster8596cbin.1	42.70	Clostridiales	71516	2874994
Sample_SPA47.Cluster8125cbin.1	47.30	Bacteria	118005	1910820
Sample_SPA47.Cluster10912cbin.1	34.00	Bacteria	24347	1994988
Sample_SPA47.Cluster5842cbin.1	53.10	Clostridiales	15680	2258769
Sample_SPA47.Cluster54cbin.1	59.30	Clostridiales	10343	2121098
Sample_SPA47.Cluster5816	45.90	Selenomonadales	137853	1713844
Sample_SPA47.Cluster344cbin.1	57.40	Clostridiales	42675	3073843
Sample_SPA47.Cluster1713cbin.1	53.80	Clostridiales	293961	1913968
Sample_SPA47.Cluster251cbin.1	60.30	Clostridiales	36620	2457082
Sample_SPA47.mb.31	53.00	Clostridia	86456	1975519
Sample_SPA47.mb.119cbin.1	45.10	Actinobacteria	18495	1843610
Sample_SPA47.Cluster268	61.20	Proteobacteria	180422	2447975
Sample_SPA47.Cluster8012	40.90	Clostridiales	228005	2088156
Sample_SPA47.mb.102	62.40	Actinobacteria	31087	1825175
Sample_SPA47.mb.103cbin.1	49.00	Bacteroidales	123929	3224020
Sample_SPA47.mb.117	60.60	Clostridiales	128203	2501350
Sample_SPA47.Cluster903	55.30	Proteobacteria	224146	2402758
Sample_SPA47.Cluster980	56.40	Bifidobacteriaceae	149549	2057693
Sample_SPA47.Cluster10cbin.1	61.80	Clostridiales	31297	3337747
Sample_SPA47.mb.112cbin.1	56.60	Clostridiales	12359	2371388
Sample_SPA47.Cluster310cbin.1	59.30	Bacteroidetes	84953	2620513
Sample_SPA47.mb.16	60.10	Clostridiales	86715	2437418
Sample_SPA47.mb.30cbin.1	61.80	Clostridiales	80290	1828802
Sample_SPA47.mb.42	36.30	Clostridiales	33721	2497798
Sample_SPA47.Cluster89cbin.1	60.30	Bifidobacteriaceae	24021	2407318
Sample_SPA47.mb.36cbin.1	60.30	Clostridiales	35636	1899839
Sample_SPA47.Cluster6035	43.50	Bacteroidales	123957	2871788
Sample_SPA47.mb.29	45.70	Clostridiales	10933	2161452
Sample_SPA47.mb.49cbin.1	49.00	Clostridiales	14152	2209614
Sample_SPA47.mb.39cbin.1	54.20	Bacteroidetes	86617	2117135
Sample_SPA47.Cluster6029	43.20	Bacteria	104475	4053135
Sample_SPA47.mb.34	58.10	Clostridiales	8118	2172824
Sample_SPA47.mb.11	40.90	Bacteroidales	32428	3169943
Sample_SPA47.mb.84cbin.1	55.80	Clostridiales	17271	3137056
Sample_SPA47.mb.68	44.80	Clostridiales	16268	2737729
Sample_SPA47.Cluster4528	45.10	Bacteroidales	95444	4386373
Sample_SPA47.mb.87cbin.1	51.60	Clostridiales	22893	2038297
Sample_SPA47.mb.54cbin.1	59.80	Bacteroidetes	151509	2809094
Sample_SPA47.Cluster7793cbin.1	45.00	Bacteroidales	27954	4476842
Sample_SPA47.mb.27cbin.1	41.90	Lachnospiraceae	80430	2524882
Sample_SPA47.mb.74	60.00	Bacteroidetes	72340	2488341

Sample_SPA47.mb.64	44.10	Clostridiales	181169	2799391
Sample_SPA47.mb.86	49.70	Clostridiales	147126	2619594
Sample_SPA47.mb.43	41.80	Clostridiales	15827	3465404
Sample_SPA47.mb.6	42.90	Bacteria	122797	4620240
Sample_SPB03.Cluster3771	51.40	Bacteria	49780	1733601
Sample_SPB03.Cluster911cbin.1	58.10	Clostridiales	57825	3114608
Sample_SPB03.mb.105	41.80	Clostridiales	109417	2307174
Sample_SPB03.mb.3	37.50	Clostridiales	244098	2060834
Sample_SPB03.Cluster3812cbin.1	49.60	Clostridiales	95463	2233977
Sample_SPB03.mb.42	36.30	Clostridiales	73465	2587008
Sample_SPB03.mb.65cbin.1	49.40	Clostridiales	88598	1980255
Sample_SPB03.mb.4	40.40	Clostridiales	66445	2972480
Sample_SPB03.mb.50cbin.1	59.00	Clostridiales	8671	2130407
Sample_SPB03.mb.74cbin.1	48.90	Bacteria	35786	3445363
Sample_SPB03.Cluster36cbin.1	63.90	Actinobacteria	7962	2348002
Sample_SPB03.mb.56	43.00	Clostridiales	63871	3049467
Sample_SPB03.Cluster323	60.70	Bifidobacteriaceae	18277	1762442
Sample_SPB03.mb.48	54.00	Clostridiales	14145	1805784
Sample_SPB03.Cluster9294	43.10	Clostridiales	171829	2887744
Sample_SPB03.mb.47	33.90	Clostridiales	53007	1882583
Sample_SPB03.mb.64cbin.1	44.40	Selenomonadales	6162	1891844
Sample_SPB03.mb.5	52.60	Clostridiales	252404	2059181
Sample_SPB03.mb.22	43.60	Lachnospiraceae	117064	2898957
Sample_SPB03.mb.81	57.60	Clostridiales	8326	1982030
Sample_SPB03.mb.28	36.20	Clostridiales	16930	2598038
Sample_SPB03.mb.7	45.00	Clostridiales	87187	2679056
Sample_SPB03.mb.2cbin.1	48.50	Bacteroidales	117189	3431634
Sample_SPB03.mb.89	48.20	Clostridiales	70942	2884596
Sample_SPB03.mb.92	54.90	Bacteroidetes	69366	1846838
Sample_SPB03.mb.55	43.30	Bacteroidales	449395	3110152
Sample_SPB03.mb.67	49.40	Lachnospiraceae	67780	3112574
Sample_SPB03.mb.79cbin.1	43.30	Bacteria	14015	3717470
Sample_SPB03.mb.60cbin.1	45.30	Bacteroidales	21866	4192473
Sample_SPB03.mb.78	46.30	Bacteroidales	15769	3365392
Sample_SPB03.mb.100cbin.1	50.90	Enterobacteriaceae	51362	4343447
Sample_SPB03.mb.35cbin.1	57.90	Enterobacteriaceae	138610	4919136
Sample_SPB04.mb.16	26.70	Bacteria	8501	1147621
Sample_SPB04.mb.37	54.40	Bacteria	116820	2146650
Sample_SPB04.Cluster2864cbin.1	51.20	Clostridiales	116755	2586474
Sample_SPB04.mb.53cbin.1	57.10	Clostridiales	12553	2345116
Sample_SPB04.mb.15	60.50	Actinobacteria	4821	1918137
Sample_SPB04.Cluster3664cbin.1	49.40	Bacteroidales	64043	2727432
Sample_SPB04.Cluster4111	47.40	Selenomonadales	38241	2141026
Sample_SPB04.mb.61cbin.1	48.40	Clostridiales	41583	2823225
Sample_SPB04.mb.24	58.90	Clostridiales	27519	2131936
Sample_SPB04.Cluster4530cbin.1	42.90	Prevotella	34872	3017369
Sample_SPB04.Cluster383cbin.1	60.50	Bifidobacteriaceae	100917	1857572
Sample_SPB04.Cluster1539cbin.1	49.90	Bacteroidales	18917	2853624
Sample_SPB04.mb.69cbin.1	56.50	Clostridiales	11951	2479508
Sample_SPB04.Cluster9349cbin.1	37.40	Clostridiales	129673	2749613
Sample_SPB04.mb.44	44.20	Clostridiales	144228	2657980
Sample_SPB04.mb.56cbin.1	51.10	Clostridiales	275226	2315976
Sample_SPB04.mb.7cbin.1	46.80	Lachnospiraceae	39375	3284397
Sample_SPB04.Cluster2090cbin.1	55.40	Proteobacteria	25025	2696537
Sample_SPB04.mb.60cbin.1	43.20	Lachnospiraceae	58189	3288792
Sample_SPB04.mb.67cbin.1	44.30	Prevotella	15678	3783017
Sample_SPB04.Cluster4564cbin.1	45.40	Bacteroidales	42833	3908822
Sample_SPB04.mb.32cbin.1	45.10	Bacteroidales	27750	4613910

Sample_SPB04.Cluster3304cbin.1	50.80	Enterobacteriaceae	195272	4573920
Sample_SPB06.Cluster12077	27.90	Clostridiales	118035	1355587
Sample_SPB06.mb.101cbin.1	26.10	Bacteria	59839	1147710
Sample_SPB06.mb.109	26.60	Bacteria	73441	1215045
Sample_SPB06.Cluster305cbin.1	58.90	Clostridiales	8879	1836066
Sample_SPB06.Cluster460cbin.1	58.70	Clostridiales	53046	2358907
Sample_SPB06.Cluster5391cbin.1	48.10	Clostridiales	99549	2992114
Sample_SPB06.Cluster11221	33.70	Clostridiales	67157	1698604
Sample_SPB06.mb.121	42.30	Clostridiales	47257	2563003
Sample_SPB06.Cluster5816	44.60	Clostridiales	178567	2000115
Sample_SPB06.Cluster10276	32.60	Lactobacillales	7658	1710155
Sample_SPB06.Cluster11862	31.00	Firmicutes	81561	1557289
Sample_SPB06.Cluster230	60.30	Bifidobacteriaceae	100946	2027561
Sample_SPB06.mb.22cbin.1	58.30	Clostridiales	55563	2968322
Sample_SPB06.Cluster6548	44.10	Clostridiales	296576	2586816
Sample_SPB06.Cluster483	58.20	Bacteria	211576	3021624
Sample_SPB06.mb.124cbin.1	54.00	Clostridiales	20700	2235339
Sample_SPB06.Cluster79cbin.1	63.10	Actinobacteria	77546	2681273
Sample_SPB06.Cluster6857	43.80	Lachnospiraceae	100853	2487075
Sample_SPB06.Cluster11209	36.10	Clostridiales	228450	2675992
Sample_SPB06.mb.100	38.10	Clostridiales	121964	1902345
Sample_SPB06.mb.30cbin.1	59.70	Clostridiales	7110	1531563
Sample_SPB06.mb.27	38.30	Clostridiales	38632	2997920
Sample_SPB06.mb.139	61.00	Clostridiales	23423	2324094
Sample_SPB06.Cluster8017	41.20	Lachnospiraceae	77218	2818684
Sample_SPB06.mb.115cbin.1	60.20	Bacteroidetes	42052	2568949
Sample_SPB06.mb.132cbin.1	49.40	Bacteroidales	51528	2940119
Sample_SPB06.mb.126	44.50	Clostridiales	70329	2469401
Sample_SPB06.mb.66	49.70	Clostridiales	114460	1974386
Sample_SPB06.mb.55	61.40	Clostridiales	76403	1692397
Sample_SPB06.mb.20	60.40	Clostridiales	26035	1961024
Sample_SPB06.mb.33cbin.1	43.40	Lachnospiraceae	37782	1829804
Sample_SPB06.mb.48	58.70	Clostridiales	47050	2517457
Sample_SPB06.mb.46cbin.1	52.00	Clostridiales	208242	2248886
Sample_SPB06.Cluster5860cbin.1	45.20	Bacteroidales	180416	4416510
Sample_SPB06.Cluster6553cbin.1	42.10	Bacteroidales	105233	4190992
Sample_SPB06.mb.74cbin.1	44.70	Selenomonadales	19794	1828889
Sample_SPB06.mb.81	54.80	Clostridiales	8115	1424872
Sample_SPB06.Cluster7281cbin.1	45.10	Bacteroidales	150176	4494753
Sample_SPB06.mb.97	56.70	Clostridiales	50536	2497498
Sample_SPB06.mb.83cbin.1	59.70	Actinobacteria	119135	2125864
Sample_SPB06.mb.44	40.30	Clostridiales	75695	2914362
Sample_SPB06.mb.54cbin.1	39.30	Streptococcus	6645	2014615
Sample_SPB06.mb.93	47.80	Clostridiales	38858	1865450
Sample_SPB06.mb.65	43.80	Clostridiales	13175	2577241
Sample_SPB06.Cluster6571cbin.1	44.00	Bacteria	83932	5364619
Sample_SPB06.mb.96cbin.1	64.20	Deltaproteobacteria	11159	2265215
Sample_SPB06.mb.89cbin.1	48.40	Proteobacteria	38545	2559053
Sample_SPB06.mb.88cbin.1	46.50	Bacteroidales	42296	4172345
Sample_SPB06.mb.25	42.60	Bacteroidales	144830	6595634
Sample_SPB07.Cluster10588cbin.1	24.30	Bacteria	61976	961132
Sample_SPB07.Cluster10626	26.40	Bacteria	125110	1111497
Sample_SPB07.Cluster3324cbin.1	47.40	Bacteria	10856	1638213
Sample_SPB07.mb.19	26.60	Bacteria	90353	1327831
Sample_SPB07.Cluster127cbin.1	61.40	Clostridiales	51936	1768919
Sample_SPB07.Cluster2229cbin.1	51.40	Clostridiales	22874	2254571
Sample_SPB07.mb.109	62.20	Clostridiales	11099	1781015
Sample_SPB07.mb.105cbin.1	58.50	Clostridiales	9913	1935040

Sample_SPB07.mb.44	62.20	Clostridiales	33868	1757507
Sample_SPB07.mb.47	59.30	Clostridiales	13267	1866186
Sample_SPB07.Cluster1982	54.70	Bacteroidetes	132479	2865936
Sample_SPB07.Cluster1478	55.50	Proteobacteria	114204	2278325
Sample_SPB07.Cluster5497cbin.1	43.30	Lachnospiraceae	49263	2128875
Sample_SPB07.Cluster153cbin.1	58.60	Bacteria	16878	2879288
Sample_SPB07.mb.87cbin.1	29.90	Bacteria	40666	2516683
Sample_SPB07.mb.104	59.30	Clostridiales	18142	2541210
Sample_SPB07.Cluster1836	56.30	Bifidobacteriaceae	473789	2191524
Sample_SPB07.Cluster283	60.40	Bifidobacteriaceae	83907	1911526
Sample_SPB07.Cluster8450	39.50	Clostridiales	77535	2168308
Sample_SPB07.mb.31cbin.1	49.00	Firmicutes	32275	1811209
Sample_SPB07.mb.42	46.60	Lachnospiraceae	56403	2626066
Sample_SPB07.Cluster3014cbin.1	55.30	Bacteroidales	51978	2675541
Sample_SPB07.mb.2	44.10	Clostridiales	170198	2410727
Sample_SPB07.mb.50	60.30	Actinobacteria	9866	1752895
Sample_SPB07.Cluster7656	41.30	Lachnospiraceae	75490	2709841
Sample_SPB07.mb.38cbin.1	63.60	Actinobacteria	40604	2611692
Sample_SPB07.mb.30	59.30	Bacteroidetes	152133	2565908
Sample_SPB07.mb.66	54.40	Clostridiales	22151	2688861
Sample_SPB07.mb.4cbin.1	54.40	Bacteroidetes	94607	2202675
Sample_SPB07.mb.86	58.30	Clostridiales	55361	2798783
Sample_SPB07.mb.14cbin.1	37.30	Clostridiales	94976	2771063
Sample_SPB07.mb.48	44.10	Lachnospiraceae	14235	2854547
Sample_SPB07.mb.94cbin.1	49.10	Clostridiales	10496	2245182
Sample_SPB07.mb.49	45.10	Clostridiales	29864	2789041
Sample_SPB07.Cluster6051cbin.1	43.30	Bacteria	86032	4056458
Sample_SPB07.Cluster4507cbin.1	48.30	Bacteroidales	137138	3770281
Sample_SPB07.Cluster4015cbin.1	43.10	Bacteria	7223	4212262
Sample_SPB07.Cluster5231	45.40	Bacteroidales	179476	4124602
Sample_SPB07.mb.72	49.50	Lachnospiraceae	86601	2858333
Sample_SPB07.mb.75	47.30	Bacteroidales	22298	3041625
Sample_SPB07.mb.7cbin.1	44.30	Bacteroidales	68531	3512872
Sample_SPB07.mb.89	40.80	Bacteroidales	55518	3080307
Sample_SPB08.Cluster2354cbin.1	45.70	Bacteria	15069	4070868
Sample_SPB08.Cluster4881	49.40	Clostridiales	116352	1934789
Sample_SPB08.Cluster340cbin.1	58.40	Clostridiales	83396	2306060
Sample_SPB08.Cluster151	61.80	Clostridiales	80473	1602755
Sample_SPB08.mb.31	26.00	Bacteria	35513	1328662
Sample_SPB08.Cluster3815cbin.1	49.60	Clostridiales	23403	2643651
Sample_SPB08.Cluster8127	44.90	Actinobacteria	337380	1838228
Sample_SPB08.Cluster4330cbin.1	45.70	Selenomonadales	33208	1857192
Sample_SPB08.Cluster5760	45.70	Firmicutes	108854	1601779
Sample_SPB08.mb.46	57.50	Bacteria	29425	1766822
Sample_SPB08.mb.5	58.50	Clostridia	39674	2960042
Sample_SPB08.mb.13cbin.1	57.30	Clostridiales	55875	1784717
Sample_SPB08.Cluster2943cbin.1	53.10	Clostridiales	157971	1890799
Sample_SPB08.mb.25cbin.1	53.80	Clostridiales	46183	1834461
Sample_SPB08.Cluster6125	46.30	Bacteroidetes	287051	2351962
Sample_SPB08.mb.53	58.10	Clostridiales	52020	3012930
Sample_SPB08.mb.38	56.00	Clostridiales	11042	2256902
Sample_SPB08.mb.63	55.70	Clostridiales	130707	1912673
Sample_SPB08.mb.55	57.90	Clostridiales	91705	2017909
Sample_SPB08.Cluster7895	44.30	Clostridiales	152513	2103501
Sample_SPB08.Cluster6032cbin.1	44.90	Clostridiales	15616	2454159
Sample_SPB08.mb.116	55.40	Clostridiales	76976	3509350
Sample_SPB08.mb.114	54.30	Bacteroidetes	78909	2314717
Sample_SPB08.Cluster8080	43.60	Clostridiales	252391	2925515

Sample_SPB08.mb.50	61.00	Clostridiales	12492	2169313
Sample_SPB08.mb.7	57.60	Clostridiales	31458	1658697
Sample_SPB08.mb.58cbin.1	44.90	Selenomonadales	22636	1775491
Sample_SPB08.mb.70cbin.1	48.90	Clostridiales	8935	2426274
Sample_SPB08.mb.101cbin.1	37.40	Clostridiales	101053	2765190
Sample_SPB08.mb.65	56.60	Clostridiales	53542	2623848
Sample_SPB08.mb.59cbin.1	59.00	Clostridiales	27920	2093756
Sample_SPB08.mb.86	59.80	Clostridiales	96352	2197618
Sample_SPB08.mb.26cbin.1	43.30	Bacteria	31069	4012334
Sample_SPB08.Cluster8661cbin.1	45.50	Bacteroidales	22212	3681500
Sample_SPB08.Cluster4205cbin.1	41.90	Bacteroidales	18970	4404730
Sample_SPB08.mb.62	58.90	Bacteroidetes	107446	2761390
Sample_SPB08.mb.69	41.70	Lachnospiraceae	105615	2721989
Sample_SPB08.mb.74cbin.1	35.90	Clostridiales	49087	2449937
Sample_SPB08.mb.79	42.00	Lachnospiraceae	32017	2286407
Sample_SPB08.mb.87cbin.1	46.40	Bacteroidales	142848	4061936
Sample_SPB09.Cluster19cbin.1	62.20	Clostridiales	9585	1480569
Sample_SPB09.mb.30	58.40	Clostridiales	52049	2867055
Sample_SPB09.mb.19	42.70	Clostridiales	63033	3339233
Sample_SPB09.Cluster7564cbin.1	40.90	Clostridiales	66462	3484331
Sample_SPB09.mb.5cbin.1	36.50	Clostridiales	7311	2078678
Sample_SPB09.mb.20cbin.1	52.20	Clostridiales	130345	2294429
Sample_SPB09.Cluster332cbin.1	56.50	Clostridiales	15436	2552186
Sample_SPB09.mb.50cbin.1	57.90	Clostridiales	18834	2224528
Sample_SPB09.mb.2	41.90	Clostridiales	238572	1966669
Sample_SPB09.Cluster116	60.40	Bifidobacteriaceae	70017	1905780
Sample_SPB09.Cluster5574	43.60	Clostridiales	114828	2254440
Sample_SPB09.Cluster1064	54.20	Bacteroidales	84203	2387513
Sample_SPB09.mb.85cbin.1	49.10	Clostridiales	13881	2511248
Sample_SPB09.mb.27	41.10	Clostridiales	208653	2709700
Sample_SPB09.Cluster245cbin.1	58.60	Bacteroidetes	183898	2692179
Sample_SPB09.Cluster8333cbin.1	37.20	Clostridiales	60409	2765928
Sample_SPB09.mb.88cbin.1	49.20	Bacteroidales	42701	3220703
Sample_SPB09.mb.67	46.60	Lachnospiraceae	69246	2994010
Sample_SPB09.mb.61	54.50	Bacteroidetes	125839	2216928
Sample_SPB09.mb.32	41.40	Lachnospiraceae	117573	2795606
Sample_SPB09.mb.64	27.30	Clostridiales	22795	3422019
Sample_SPB09.mb.54cbin.1	48.50	Proteobacteria	73818	2596661
Sample_SPB09.mb.56	43.40	Bacteroidales	274935	3226542
Sample_SPB09.Cluster3968	42.20	Bacteria	26174	4335392
Sample_SPB09.mb.24cbin.1	46.20	Bacteroidales	168141	3971203
Sample_SPB09.mb.82cbin.1	41.70	Bacteroidales	26737	4090056
Sample_SPB09.mb.46cbin.1	44.90	Bacteroidales	26859	4362076
Sample_SPB09.mb.8cbin.1	57.90	Enterobacteriaceae	73258	4913944
Sample_SPB10.Cluster7361cbin.1	33.30	Clostridiales	144560	1861076
Sample_SPB10.Cluster4204cbin.1	41.90	Clostridiales	27689	2632507
Sample_SPB10.Cluster2637cbin.1	48.10	Clostridiales	98871	2877731
Sample_SPB10.mb.63	37.80	Clostridiales	287587	1872173
Sample_SPB10.Cluster1613cbin.1	47.70	Firmicutes	37494	1617323
Sample_SPB10.mb.73	57.70	Clostridiales	12839	1855234
Sample_SPB10.Cluster7560	29.90	Bacteria	64494	2160459
Sample_SPB10.Cluster5073cbin.1	40.10	Streptococcus	6193	1559399
Sample_SPB10.mb.60cbin.1	49.60	Clostridiales	33507	2132192
Sample_SPB10.mb.9	60.20	Clostridiales	151630	1944555
Sample_SPB10.Cluster192	60.50	Bifidobacteriaceae	55321	1887802
Sample_SPB10.Cluster6869cbin.1	35.90	Clostridiales	172320	2584888
Sample_SPB10.Cluster6006	37.50	Clostridiales	111736	2562713
Sample_SPB10.mb.71cbin.1	60.20	Clostridiales	58040	2990739

Sample_SPB10.mb.58	38.80	Clostridiales	78472	1996120
Sample_SPB10.mb.10cbin.1	58.00	Clostridiales	46765	2404798
Sample_SPB10.Cluster4063	41.40	Lachnospiraceae	89269	2866756
Sample_SPB10.mb.70cbin.1	56.40	Clostridiales	11578	2518798
Sample_SPB10.Cluster12cbin.1	64.80	Actinobacteria	6511	2653929
Sample_SPB10.mb.36cbin.1	61.50	Clostridiales	85150	3543281
Sample_SPB10.mb.6cbin.1	58.20	Clostridiales	48060	2552282
Sample_SPB10.mb.29	43.70	Lachnospiraceae	43559	2711895
Sample_SPB10.mb.77	43.20	Lachnospiraceae	115718	3003496
Sample_SPB10.mb.54cbin.1	48.70	Proteobacteria	11436	2436103
Sample_SPB10.Cluster2695cbin.1	46.50	Bacteroidales	132555	4055767
Sample_SPB10.mb.53	46.10	Bacteroidales	158860	3410303
Sample_SPB10.Cluster4934cbin.1	41.80	Bacteroides	58886	5618303
Sample_SPB10.mb.28	41.60	Bacteroidales	241519	5197870
Sample_SPB10.mb.67cbin.1	51.10	Enterobacteriaceae	6543	3909192
Sample_SPB11.Cluster5576	41.70	Clostridiales	147630	2792134
Sample_SPB11.Cluster2626	48.20	Clostridiales	101321	2771169
Sample_SPB11.mb.24	55.70	Clostridiales	9962	2155601
Sample_SPB11.Cluster14	62.30	Clostridiales	15018	2204787
Sample_SPB11.Cluster1613cbin.1	51.30	Clostridiales	21039	2323733
Sample_SPB11.mb.54	58.50	Clostridiales	24315	2850048
Sample_SPB11.Cluster96cbin.1	58.90	Clostridiales	10638	2243924
Sample_SPB11.mb.58	60.20	Clostridiales	10471	2402701
Sample_SPB11.Cluster2830	47.40	Selenomonadales	78745	1996728
Sample_SPB11.Cluster7987cbin.1	33.50	Bacteria	23429	2746736
Sample_SPB11.Cluster12cbin.1	61.00	Bifidobacteriaceae	6015	1510518
Sample_SPB11.Cluster6266	40.60	Clostridiales	187584	2058811
Sample_SPB11.Cluster1895	51.50	Clostridiales	222169	2128308
Sample_SPB11.mb.1	46.60	Lachnospiraceae	79271	2869806
Sample_SPB11.Cluster4339	44.00	Clostridiales	197389	2502136
Sample_SPB11.mb.4	49.50	Clostridiales	15351	2312300
Sample_SPB11.mb.3	54.40	Bacteroidetes	107408	1910689
Sample_SPB11.Cluster4404	43.70	Clostridiales	178809	2525565
Sample_SPB11.Cluster746	55.60	Proteobacteria	149048	2230334
Sample_SPB11.mb.63cbin.1	60.70	Clostridiales	50591	2364395
Sample_SPB11.mb.80cbin.1	45.20	Actinobacteria	23162	1861829
Sample_SPB11.mb.39	59.20	Clostridiales	9592	1846581
Sample_SPB11.mb.66	59.90	Actinobacteria	108706	2240605
Sample_SPB11.mb.10	41.60	Lachnospiraceae	74432	2615493
Sample_SPB11.Cluster7487cbin.1	36.30	Clostridiales	115781	3510474
Sample_SPB11.mb.53cbin.1	41.90	Lachnospiraceae	51849	2339415
Sample_SPB11.mb.13cbin.1	43.40	Bacteroidales	64286	3111157
Sample_SPB11.mb.47	46.00	Bacteroidales	37657	2979480
Sample_SPB11.Cluster2040cbin.1	44.10	Bacteroidales	72544	3617791
Sample_SPB11.mb.73cbin.1	42.90	Clostridiales	147522	2644703
Sample_SPB11.Cluster3214cbin.1	46.30	Bacteroidales	153095	4387432
Sample_SPB11.mb.74	40.30	Bacteroidales	34302	3293242
Sample_SPB11.mb.8cbin.1	44.50	Prevotella	19924	3990583
Sample_SPB11.Cluster1227cbin.1	41.70	Bacteroidales	25240	5224900
Sample_SPB12.Cluster19582	26.40	Bacteria	113389	1122008
Sample_SPB12.Cluster18884	32.10	Bacteria	299421	1827647
Sample_SPB12.Cluster6377cbin.1	49.70	Clostridia	55640	1792675
Sample_SPB12.Cluster3162cbin.1	54.50	Actinobacteria	20448	1484367
Sample_SPB12.mb.124	58.70	Clostridiales	10947	1892027
Sample_SPB12.mb.127	58.60	Clostridiales	9301	2670086
Sample_SPB12.Cluster4807cbin.1	50.70	Bacteroidales	19979	2275216
Sample_SPB12.mb.131	41.20	Clostridiales	16162	2881146
Sample_SPB12.mb.15	36.20	Clostridiales	248497	1919944

Sample_SPB12.Cluster13513cbin.1	41.40	Clostridiales	140551	2533968
Sample_SPB12.mb.138	51.50	Clostridiales	58924	2294673
Sample_SPB12.Cluster1418	55.60	Bacteria	174517	2689704
Sample_SPB12.Cluster18261	32.50	Lactobacillales	75394	1879386
Sample_SPB12.Cluster11654cbin.1	42.40	Lachnospiraceae	18721	2324458
Sample_SPB12.mb.34	45.60	Clostridiales	92790	1583580
Sample_SPB12.Cluster197	62.40	Proteobacteria	68391	2338404
Sample_SPB12.mb.101cbin.1	51.10	Clostridiales	147408	2233978
Sample_SPB12.mb.4	36.30	Clostridiales	93467	2575755
Sample_SPB12.Cluster9121cbin.1	43.50	Lachnospiraceae	20109	2722981
Sample_SPB12.mb.132cbin.1	59.80	Bifidobacteriaceae	30176	1762152
Sample_SPB12.mb.33	53.20	Clostridiales	11294	1987841
Sample_SPB12.mb.94	26.70	Bacteria	79276	1381268
Sample_SPB12.mb.121	60.50	Bifidobacteriaceae	83870	1881833
Sample_SPB12.mb.10cbin.1	44.90	Prevotella	13019	3076997
Sample_SPB12.mb.128cbin.1	50.60	Bacteroidales	143046	2200942
Sample_SPB12.mb.43cbin.1	48.40	Clostridiales	82839	2779786
Sample_SPB12.mb.29cbin.1	47.70	Selenomonadales	31129	2006099
Sample_SPB12.mb.35	61.20	Clostridiales	6619	1987049
Sample_SPB12.mb.23	42.70	Clostridiales	204061	2786119
Sample_SPB12.mb.21	44.30	Clostridiales	156140	2644062
Sample_SPB12.mb.51cbin.1	44.60	Clostridiales	146692	1660082
Sample_SPB12.mb.37	41.30	Clostridiales	96412	2542155
Sample_SPB12.mb.47	44.90	Lachnospiraceae	89155	2583189
Sample_SPB12.mb.52	44.00	Clostridiales	97053	2201312
Sample_SPB12.mb.99cbin.1	45.40	Clostridiales	102005	2260220
Sample_SPB12.Cluster6415cbin.1	45.50	Bacteroidales	28378	3957871
Sample_SPB12.mb.90cbin.1	51.20	Firmicutes	38668	2159361
Sample_SPB12.mb.87cbin.1	42.60	Clostridiales	104526	2795054
Sample_SPB12.Cluster1202cbin.1	57.40	Enterobacteriaceae	226235	5321391
Sample_SPB13.Cluster2582	46.50	Bacteria	114472	3968614
Sample_SPB13.Cluster286	60.30	Clostridiales	109440	1824937
Sample_SPB13.Cluster219cbin.1	59.00	Clostridiales	19386	2001857
Sample_SPB13.mb.28	37.10	Clostridiales	20173	2147681
Sample_SPB13.Cluster413	57.70	Clostridiales	69451	2218274
Sample_SPB13.mb.13	58.60	Bacteroidetes	178222	2424719
Sample_SPB13.Cluster3974cbin.1	42.60	Clostridiales	7700	2283232
Sample_SPB13.mb.43cbin.1	60.70	Clostridiales	72153	2355885
Sample_SPB13.Cluster4375cbin.1	46.80	Lachnospiraceae	58970	3162701
Sample_SPB13.Cluster6039	40.60	Clostridiales	175133	2120179
Sample_SPB13.Cluster3169	43.60	Selenomonadales	133862	2432873
Sample_SPB13.mb.32	54.70	Bacteroidetes	95933	2062241
Sample_SPB13.Cluster17cbin.1	60.40	Bifidobacteriaceae	13967	2093520
Sample_SPB13.Cluster418	59.40	Bifidobacteriaceae	124579	2168015
Sample_SPB13.Cluster946	54.20	Proteobacteria	101610	2394022
Sample_SPB13.mb.1	43.80	Lachnospiraceae	79386	2707282
Sample_SPB13.Cluster8cbin.1	61.40	Clostridiales	23769	3510948
Sample_SPB13.mb.81	58.50	Clostridiales	14183	1962098
Sample_SPB13.mb.83	41.60	Clostridiales	14803	2625544
Sample_SPB13.Cluster3792cbin.1	40.80	Bacteroidales	57155	3094999
Sample_SPB13.mb.69cbin.1	60.40	Clostridiales	127289	3122874
Sample_SPB13.mb.38cbin.1	41.30	Clostridiales	121957	3583236
Sample_SPB13.mb.66cbin.1	41.40	Lachnospiraceae	10666	2600521
Sample_SPB13.mb.4cbin.1	31.30	Firmicutes	63164	2478475
Sample_SPB13.Cluster2682cbin.1	45.80	Bacteroidales	118345	3619316
Sample_SPB13.Cluster2803cbin.1	45.40	Bacteroidales	68270	3798710
Sample_SPB13.mb.59	43.30	Bacteroidales	339293	3159156
Sample_SPB13.mb.67cbin.1	41.90	Lachnospiraceae	63154	2366680

Sample_SPB13.Cluster6576cbin.1	41.70	Bacteroidales	34613	4492546
Sample_SPB13.mb.74	59.20	Bacteroidetes	123012	2860748
Sample_SPB13.mb.31cbin.1	45.10	Bacteroidales	73843	4533994
Sample_SPB13.mb.63	48.50	Bacteroidales	174277	3600162
Sample_SPB15.Cluster11681	24.10	Bacteria	51749	938645
Sample_SPB15.mb.5cbin.1	25.30	Bacteria	59878	1247784
Sample_SPB15.Cluster5cbin.1	49.00	Clostridiales	29340	2129781
Sample_SPB15.mb.18	63.60	Clostridiales	10289	1858052
Sample_SPB15.mb.44cbin.1	53.60	Clostridia	22865	1781810
Sample_SPB15.Cluster10967	37.70	Clostridiales	242807	2208747
Sample_SPB15.Cluster34	62.30	Clostridiales	10699	1338409
Sample_SPB15.mb.67	51.80	Clostridiales	10204	1749994
Sample_SPB15.Cluster362cbin.1	60.10	Clostridiales	103640	1788391
Sample_SPB15.mb.65	59.90	Clostridiales	17993	2093119
Sample_SPB15.mb.22cbin.1	59.50	Clostridiales	19278	2027232
Sample_SPB15.mb.48	58.50	Clostridiales	17628	1681121
Sample_SPB15.mb.25	57.50	Clostridiales	11352	1945130
Sample_SPB15.mb.35	51.80	Bacteroidetes	36374	3011670
Sample_SPB15.Cluster4864cbin.1	49.60	Clostridiales	153353	2478688
Sample_SPB15.mb.33cbin.1	59.40	Clostridiales	10055	2305540
Sample_SPB15.mb.50cbin.1	57.00	Clostridiales	107095	1822576
Sample_SPB15.mb.64	63.90	Clostridiales	60910	2216108
Sample_SPB15.Cluster1660cbin.1	53.40	Clostridiales	30932	2019141
Sample_SPB15.mb.3	40.70	Lachnospiraceae	17133	2723097
Sample_SPB15.mb.53	38.60	Clostridiales	32444	2052143
Sample_SPB15.mb.60cbin.1	57.10	Clostridiales	203872	1884448
Sample_SPB15.mb.58cbin.1	45.80	Lachnospiraceae	34106	3312834
Sample_SPB15.mb.73cbin.1	45.70	Clostridiales	50267	2521484
Sample_SPB15.mb.84cbin.1	51.60	Clostridiales	175604	2214420
Sample_SPB15.mb.37	50.90	Enterobacteriaceae	140187	4631367
Sample_SPB16.mb.47	28.30	Bacteria	58609	1456494
Sample_SPB16.mb.43	26.90	Bacteria	29316	2936985
Sample_SPB16.mb.23cbin.1	48.90	Clostridiales	8055	2421276
Sample_SPB16.Cluster7479cbin.1	33.70	Bacteria	5881	1849358
Sample_SPB16.mb.66	59.30	Clostridiales	16445	1937108
Sample_SPB16.mb.58	45.10	Lachnospiraceae	187409	2415767
Sample_SPB16.Cluster975cbin.1	57.20	Proteobacteria	26558	2015373
Sample_SPB16.mb.40cbin.1	44.90	Clostridiales	11560	2258397
Sample_SPB16.Cluster7012	37.30	Clostridiales	257704	2139916
Sample_SPB16.mb.30cbin.1	44.80	Clostridiales	12230	2316563
Sample_SPB16.mb.53	41.90	Clostridiales	124578	2313021
Sample_SPB16.Cluster273	60.40	Bifidobacteriaceae	109653	1909350
Sample_SPB16.mb.74cbin.1	60.80	Clostridiales	9934	2324876
Sample_SPB16.Cluster8301	32.50	Lactobacillales	117526	1876781
Sample_SPB16.mb.37cbin.1	45.10	Prevotella	14371	3108234
Sample_SPB16.mb.19cbin.1	37.30	Clostridiales	132061	2627222
Sample_SPB16.Cluster7212cbin.1	40.00	Streptococcus	20248	2000123
Sample_SPB16.Cluster5972cbin.1	41.10	Lachnospiraceae	101652	3190854
Sample_SPB16.mb.4cbin.1	39.60	Pasteurellaceae	7819	1690453
Sample_SPB16.mb.5	41.50	Lachnospiraceae	62929	3370454
Sample_SPB16.Cluster3018cbin.1	44.10	Bacteroidales	67892	3884137
Sample_SPB16.Cluster6185cbin.1	42.50	Bacteroides	11740	4024715
Sample_SPB17.mb.23	60.20	Bifidobacteriaceae	34926	2040292
Sample_SPB17.Cluster126cbin.1	56.30	Bifidobacteriaceae	74471	2187213
Sample_SPB17.mb.15cbin.1	58.80	Bifidobacteriaceae	32976	2276157
Sample_SPB17.Cluster17cbin.1	57.80	Clostridiales	35590	2887662
Sample_SPB17.mb.18	38.00	Lactobacillales	26349	2504348
Sample_SPB17.mb.5cbin.1	42.70	Lachnospiraceae	96252	3077216

Sample_SPB17.mb.39cbin.1	45.20	Bacteroidales	104937	4275117
Sample_SPB17.Cluster866	44.80	Bacteroidales	217147	4537333
Sample_SPB17.mb.46cbin.1	42.00	Bacteroidales	107106	4756685
Sample_SPB20.mb.3	30.30	Bacteria	165171	1663990
Sample_SPB20.mb.5	26.70	Bacteria	71894	1221966
Sample_SPB20.mb.48	51.20	Clostridiales	15769	1775021
Sample_SPB20.mb.8	26.00	Bacteria	115990	1103713
Sample_SPB20.Cluster4644cbin.1	44.70	Clostridiales	14467	1712082
Sample_SPB20.Cluster2764cbin.1	48.70	Firmicutes	32775	1921612
Sample_SPB20.mb.54cbin.1	48.50	Clostridiales	26980	2689421
Sample_SPB20.Cluster56cbin.1	60.90	Bifidobacteriaceae	10460	1682857
Sample_SPB20.mb.90	38.30	Clostridiales	7172	2225605
Sample_SPB20.mb.22	54.80	Bacteroidetes	85438	2112583
Sample_SPB20.Cluster1848cbin.1	55.20	Bacteria	126487	2794681
Sample_SPB20.Cluster9206	40.50	Clostridiales	312808	2099710
Sample_SPB20.mb.24cbin.1	47.50	Lachnospiraceae	16431	2976033
Sample_SPB20.mb.93	48.50	Clostridiales	61684	2413188
Sample_SPB20.Cluster379	59.90	Bifidobacteriaceae	183492	2177293
Sample_SPB20.mb.64	34.20	Bacteria	25577	2136506
Sample_SPB20.mb.2	45.60	Clostridiales	59490	2473222
Sample_SPB20.mb.67	59.90	Actinobacteria	20324	2042560
Sample_SPB20.mb.92	56.40	Clostridiales	62717	2560167
Sample_SPB20.mb.107	37.60	Clostridiales	33704	2404194
Sample_SPB20.mb.46cbin.1	42.30	Clostridiales	7745	2273080
Sample_SPB20.mb.77	61.20	Clostridiales	17558	2056784
Sample_SPB20.mb.53	58.90	Clostridiales	21786	2016555
Sample_SPB20.Cluster7098cbin.1	42.40	Lachnospiraceae	92835	3182042
Sample_SPB20.mb.94cbin.1	51.00	Clostridiales	111087	2718168
Sample_SPB20.mb.33	26.80	Clostridiales	59396	2776684
Sample_SPB20.mb.87	54.20	Clostridiales	25792	1727702
Sample_SPB20.mb.62cbin.1	45.00	Prevotella	20222	3127584
Sample_SPB20.mb.45cbin.1	42.70	Lachnospiraceae	198625	3530398
Sample_SPB20.mb.44	43.60	Bacteroidales	187906	2839522
Sample_SPB20.mb.36	59.00	Bacteroidetes	134685	2980813
Sample_SPB20.mb.63	36.90	Clostridiales	41081	2805983
Sample_SPB20.mb.16	44.80	Bacteroidales	52936	3175129
Sample_SPB20.mb.72	40.40	Lachnospiraceae	78393	2712351
Sample_SPB20.mb.60	46.70	Bacteroidales	57205	3178601
Sample_SPB20.Cluster4561cbin.1	45.50	Bacteroidales	79847	3814164
Sample_SPB20.mb.95	40.60	Bacteroidales	34230	3056862
Sample_SPB20.Cluster566	57.70	Enterobacteriaceae	226484	5104542
Sample_SPB21.Cluster4534	50.10	Bacteria	87208	2064598
Sample_SPB21.mb.107cbin.1	29.60	Bacteria	10403	1328766
Sample_SPB21.Cluster14434	35.10	Bacteria	109223	2235037
Sample_SPB21.mb.14	49.40	Clostridiales	112791	1979794
Sample_SPB21.mb.1cbin.1	58.20	Clostridiales	55672	3106953
Sample_SPB21.Cluster7341cbin.1	46.60	Clostridiales	55585	1790678
Sample_SPB21.Cluster9839cbin.1	43.20	Clostridiales	52557	3157984
Sample_SPB21.mb.22	32.30	Clostridiales	90546	2794169
Sample_SPB21.mb.43	55.90	Clostridiales	12072	2036345
Sample_SPB21.mb.103cbin.1	45.10	Clostridiales	70340	2554410
Sample_SPB21.mb.95	61.70	Bacteria	44235	1753442
Sample_SPB21.mb.29cbin.1	48.30	Clostridiales	27036	2805952
Sample_SPB21.mb.48	43.90	Clostridiales	139516	2970711
Sample_SPB21.mb.38	41.40	Lachnospiraceae	17117	2354177
Sample_SPB21.mb.101	54.70	Bacteroidetes	149081	2098357
Sample_SPB21.mb.45cbin.1	58.10	Clostridiales	19741	2205560
Sample_SPB21.Cluster10842cbin.1	40.10	Clostridiales	129656	2321966

Sample_SPB21.mb.55	61.90	Clostridiales	51090	1841864
Sample_SPB21.Cluster10257cbin.1	42.70	Lachnospiraceae	41548	2430987
Sample_SPB21.Cluster563cbin.1	58.80	Bacteroidetes	103893	3005580
Sample_SPB21.mb.64	54.60	Clostridiales	17368	2655648
Sample_SPB21.mb.85	36.40	Clostridiales	139715	1869092
Sample_SPB21.mb.42	43.90	Selenomonadales	17228	2148614
Sample_SPB21.mb.20	40.60	Clostridiales	130002	2614381
Sample_SPB21.mb.52	56.20	Clostridiales	14966	2476129
Sample_SPB21.Cluster9992	41.30	Lachnospiraceae	67279	2893316
Sample_SPB21.mb.44	41.30	Clostridiales	201546	2527110
Sample_SPB21.Cluster8593cbin.1	43.70	Bacteroidales	133922	2709981
Sample_SPB21.mb.33	26.80	Clostridiales	41465	2677103
Sample_SPB21.mb.96	53.70	Clostridiales	143872	2377767
Sample_SPB21.mb.99	41.40	Lachnospiraceae	82545	3169572
Sample_SPB21.mb.57cbin.1	43.30	Lachnospiraceae	67753	2996794
Sample_SPB21.Cluster8466	43.20	Bacteria	25844	3925439
Sample_SPB21.mb.78cbin.1	59.10	Bacteroidetes	152166	3016675
Sample_SPB21.mb.40cbin.1	44.70	Bacteroidales	66971	3007944
Sample_SPB21.mb.86	44.50	Clostridiales	159822	2545584
Sample_SPB21.Cluster6847cbin.1	46.60	Bacteroidales	105482	3951458
Sample_SPB21.Cluster6428	45.10	Bacteroidales	49199	4430584
Sample_SPB21.mb.70	40.60	Bacteroidales	44839	3242507
Sample_SPB21.mb.83cbin.1	48.50	Bacteroidales	107958	3825815
Sample_SPB22.Cluster12841cbin.1	27.20	Bacteria	150205	1125243
Sample_SPB22.Cluster12951	28.40	Bacteria	408707	1667130
Sample_SPB22.mb.103	24.50	Bacteria	16177	930499
Sample_SPB22.Cluster12921cbin.1	28.30	Clostridiales	103855	1389779
Sample_SPB22.Cluster5512cbin.1	49.30	Clostridiales	96193	2018769
Sample_SPB22.Cluster7098cbin.1	45.00	Clostridiales	11652	1591135
Sample_SPB22.Cluster9389	34.90	Bacteria	69807	1486387
Sample_SPB22.mb.2cbin.1	61.40	Bacteria	47021	2145465
Sample_SPB22.mb.38	52.90	Clostridia	53757	2067248
Sample_SPB22.mb.11	56.70	Clostridiales	55762	2628849
Sample_SPB22.Cluster3071	54.50	Bacteroidetes	95663	1964889
Sample_SPB22.Cluster4341cbin.1	51.50	Clostridiales	116434	1959042
Sample_SPB22.mb.53	59.50	Clostridiales	136464	2145979
Sample_SPB22.mb.88	29.60	Bacteria	134723	1265673
Sample_SPB22.Cluster10662cbin.1	40.60	Clostridiales	112278	2132697
Sample_SPB22.mb.15cbin.1	51.80	Bacteria	9140	4983952
Sample_SPB22.mb.30cbin.1	57.70	Clostridiales	12201	2528994
Sample_SPB22.Cluster7923	44.00	Clostridiales	125030	2373993
Sample_SPB22.Cluster542	60.50	Bifidobacteriaceae	57871	1897590
Sample_SPB22.mb.86	52.50	Clostridiales	60169	2082653
Sample_SPB22.mb.28	46.70	Lachnospiraceae	68930	2922101
Sample_SPB22.mb.34	45.30	Clostridiales	6766	1747850
Sample_SPB22.mb.47	46.60	Clostridiales	16191	2288233
Sample_SPB22.mb.83	58.30	Clostridiales	57846	2881856
Sample_SPB22.Cluster1106cbin.1	55.40	Proteobacteria	9832	2509326
Sample_SPB22.mb.74	57.20	Clostridiales	73382	1961084
Sample_SPB22.Cluster888	57.70	Bacteria	203207	3075709
Sample_SPB22.mb.24	44.50	Clostridiales	136048	2265319
Sample_SPB22.mb.100	37.40	Clostridiales	104694	2643412
Sample_SPB22.mb.57	60.30	Bacteroidetes	77921	2546495
Sample_SPB22.mb.92	41.80	Lachnospiraceae	212621	2070537
Sample_SPB22.Cluster11789cbin.1	43.00	Bacteria	7903	3688322
Sample_SPB22.mb.42	60.60	Deltaproteobacteria	7564	3366540
Sample_SPB22.mb.80	46.00	Bacteroidetes	197257	2461142
Sample_SPB22.Cluster5885	45.50	Bacteroidales	46318	3768896

Sample_SPB22.mb.72	41.70	Lachnospiraceae	87246	2342927
Sample_SPB22.mb.55	43.40	Bacteroidales	124265	3168790
Sample_SPB22.mb.36	46.50	Bacteroidales	72878	3508060
Sample_SPB22.Cluster5410cbin.1	45.50	Bacteroidales	29491	4510906
Sample_SPB22.mb.73	41.90	Bacteroidales	53964	4183112
Sample_SPB25.Cluster14413	24.60	Bacteria	23923	948540
Sample_SPB25.Cluster6862	46.60	Clostridiales	317558	1597482
Sample_SPB25.Cluster2209cbin.1	62.50	Clostridia	19686	2893827
Sample_SPB25.Cluster557	60.20	Actinobacteria	87341	1641065
Sample_SPB25.mb.14	51.90	Clostridiales	10419	1886336
Sample_SPB25.Cluster13262cbin.1	33.50	Bacteria	26748	2289779
Sample_SPB25.mb.51	56.60	Clostridia	7219	2181301
Sample_SPB25.mb.12	54.40	Actinobacteria	147385	1685500
Sample_SPB25.mb.20cbin.1	59.10	Clostridiales	44866	2245901
Sample_SPB25.Cluster6772	44.50	Clostridiales	32023	2337580
Sample_SPB25.mb.52	58.20	Clostridiales	43015	2958305
Sample_SPB25.mb.26	54.80	Clostridiales	139133	3060127
Sample_SPB25.mb.18cbin.1	64.70	Deltaproteobacteria	5704	1939207
Sample_SPB25.Cluster13419cbin.1	27.00	Clostridiales	15602	2851409
Sample_SPB25.mb.32	41.00	Clostridiales	42331	1991210
Sample_SPB25.mb.48cbin.1	53.50	Clostridiales	76746	2391440
Sample_SPB25.Cluster11630cbin.1	38.20	Lactobacillales	100025	1976061
Sample_SPB25.mb.38	58.10	Bacteroidetes	111349	2570401
Sample_SPB25.mb.67	58.90	Clostridiales	9278	1608452
Sample_SPB25.mb.24cbin.1	42.90	Clostridiales	11426	2571523
Sample_SPB25.Cluster677	58.50	Bacteroidetes	110962	3085037
Sample_SPB25.Cluster228cbin.1	58.90	Bacteroidetes	108634	2817611
Sample_SPB25.mb.65	53.90	Clostridiales	8707	1767287
Sample_SPB25.mb.74	42.50	Lachnospiraceae	28277	1845048
Sample_SPB25.mb.91cbin.1	36.50	Clostridiales	28365	2969011
Sample_SPB25.mb.31	37.60	Lactobacillales	102201	2803410
Sample_SPB25.mb.92	47.80	Clostridiales	44684	1916159
Sample_SPB25.Cluster6849cbin.1	46.50	Bacteroidales	118622	4188219
Sample_SPB25.mb.83cbin.1	45.00	Clostridiales	123245	2608080
Sample_SPB25.mb.82cbin.1	45.10	Lactobacillales	32385	2092994
Sample_SPB25.mb.81	28.90	Clostridiales	54224	2566961
Sample_SPB25.mb.77	38.60	Lactobacillales	102139	3087238
Sample_SPB25.Cluster7636cbin.1	41.80	Bacteroidales	83690	4894818
Sample_SPB25.Cluster6850cbin.1	41.80	Bacteroides	101079	5165813
Sample_SPB25.mb.68	64.20	Actinobacteria	173393	3184157
Sample_SPB25.mb.62cbin.1	42.80	Lactobacillales	15479	3420545
Sample_SPB25.mb.95cbin.1	56.00	Enterobacteriaceae	48700	4758027
Sample_SPB26.Cluster13102	25.20	Bacteria	132268	1268239
Sample_SPB26.Cluster128cbin.1	58.30	Clostridiales	73800	1887161
Sample_SPB26.Cluster1230cbin.1	60.50	Euryarchaeota	157734	1853734
Sample_SPB26.Cluster1477cbin.1	57.10	Clostridiales	72834	1929545
Sample_SPB26.mb.27cbin.1	54.00	Clostridiales	15943	1658979
Sample_SPB26.Cluster12694	30.00	Euryarchaeota	29270	1573727
Sample_SPB26.Cluster7424	46.10	Bacteria	168999	2571837
Sample_SPB26.mb.30	52.90	Clostridia	75390	2083820
Sample_SPB26.mb.117	60.00	Clostridiales	72047	1971984
Sample_SPB26.Cluster11079	40.60	Clostridiales	186632	2027785
Sample_SPB26.Cluster4030	52.60	Clostridiales	137513	2317922
Sample_SPB26.Cluster787	58.60	Veillonellaceae	100618	2298580
Sample_SPB26.Cluster948cbin.1	59.60	Actinobacteria	79876	2251989
Sample_SPB26.Cluster503cbin.1	60.30	Bifidobacteriaceae	95915	1972091
Sample_SPB26.Cluster1166cbin.1	57.60	Bacteria	145355	3195654
Sample_SPB26.mb.103	59.80	Bacteroidetes	117994	2520917

Sample_SPB26.mb.65	58.30	Clostridiales	46240	2910421
Sample_SPB26.mb.37	59.70	Bacteria	72127	4172617
Sample_SPB26.Cluster11740cbin.1	36.40	Lactobacillus	56560	2183732
Sample_SPB26.mb.73cbin.1	49.40	Bacteria	9046	1742373
Sample_SPB26.Cluster96	63.10	Deltaproteobacteria	78434	2662176
Sample_SPB26.mb.12	51.50	Clostridiales	158664	2171616
Sample_SPB26.Cluster10643cbin.1	43.30	Bacteroidales	163551	2846544
Sample_SPB26.mb.112	50.60	Bacteroidales	25964	2216908
Sample_SPB26.mb.16	54.60	Bacteroidales	47638	2119015
Sample_SPB26.mb.7	55.40	Euryarchaeota	32046	1913960
Sample_SPB26.Cluster7918cbin.1	43.20	Bacteria	78777	4089311
Sample_SPB26.mb.88	52.00	Bacteria	9660	4289306
Sample_SPB26.mb.34cbin.1	41.20	Lachnospiraceae	107087	2973734
Sample_SPB26.mb.33	46.30	Bacteroidales	24928	2836907
Sample_SPB26.mb.92	55.40	Bacteroidetes	54405	2462643
Sample_SPB26.mb.23	43.90	Bacteroidales	61782	3365571
Sample_SPB26.mb.77	58.90	Bifidobacteriaceae	53781	2127265
Sample_SPB26.Cluster9006cbin.1	45.20	Bacteroidales	26433	4376239
Sample_SPB26.mb.91	43.90	Clostridiales	75615	2907450
Sample_SPB26.mb.69	45.60	Bacteroidales	22984	3262982
Sample_SPB26.Cluster1013cbin.1	56.70	Enterobacteriaceae	49023	5708667
Sample_SPB27.Cluster2043cbin.1	49.50	Clostridiales	101977	2079966
Sample_SPB27.Cluster3466cbin.1	44.20	Clostridiales	7137	1549445
Sample_SPB27.mb.25cbin.1	58.10	Clostridiales	60508	3053808
Sample_SPB27.mb.24cbin.1	37.10	Clostridiales	46675	2659603
Sample_SPB27.mb.40cbin.1	55.80	Clostridiales	6346	1951667
Sample_SPB27.mb.27cbin.1	59.80	Clostridiales	81443	2103055
Sample_SPB27.Cluster2426cbin.1	46.70	Lactobacillales	40233	1943122
Sample_SPB27.mb.19cbin.1	46.30	Clostridiales	39653	2069948
Sample_SPB27.Cluster1597cbin.1	48.60	Firmicutes	36707	1812760
Sample_SPB27.Cluster210cbin.1	55.80	Clostridiales	16237	2536304
Sample_SPB27.Cluster57cbin.1	60.30	Clostridiales	52184	2396674
Sample_SPB27.Cluster6184	38.40	Clostridiales	79985	2010368
Sample_SPB27.mb.21	54.70	Bacteroidetes	168153	2107882
Sample_SPB27.Cluster1327	52.80	Clostridiales	136896	2332374
Sample_SPB27.mb.42	59.30	Clostridiales	43073	2134455
Sample_SPB27.mb.22	41.20	Clostridiales	145631	2490140
Sample_SPB27.mb.45	49.00	Clostridiales	13088	2645033
Sample_SPB27.mb.26cbin.1	60.10	Bifidobacteriaceae	171006	2283044
Sample_SPB27.Cluster5753cbin.1	37.20	Clostridiales	99832	2938785
Sample_SPB27.mb.56	27.20	Clostridiales	10426	2661761
Sample_SPB27.mb.77cbin.1	60.60	Bacteria	9728	2172559
Sample_SPB27.mb.34cbin.1	49.00	Proteobacteria	48915	2020976
Sample_SPB27.mb.66cbin.1	51.20	Lachnospiraceae	87864	2938035
Sample_SPB27.mb.70	42.70	Clostridiales	13621	2304830
Sample_SPB27.Cluster2757cbin.1	43.30	Bacteria	7123	3510933
Sample_SPB27.mb.52cbin.1	42.70	Lachnospiraceae	113061	3220059
Sample_SPB27.mb.29	58.80	Bacteroidetes	124653	2687055
Sample_SPB27.mb.38	41.70	Lachnospiraceae	73794	2302639
Sample_SPB27.mb.88	60.00	Bacteroidetes	106328	2396295
Sample_SPB27.mb.63	58.80	Bacteroidetes	235890	2918846
Sample_SPB27.Cluster2220	49.20	Clostridiales	193235	7025057
Sample_SPB27.mb.10	46.60	Bacteroidales	172786	3929664
Sample_SPB27.Cluster4319cbin.1	45.40	Bacteroidales	79679	4483437
Sample_SPB27.mb.48cbin.1	44.40	Bacteroidales	74229	3901418
Sample_SPB27.mb.54	41.90	Bacteroides	86628	4566795
Sample_SPB28.mb.43	38.40	Clostridiales	12425	2466723
Sample_SPB28.Cluster2207cbin.1	44.60	Selenomonadales	5948	1709534

Sample_SPB28.Cluster212	59.60	Actinobacteria	69431	2192929
Sample_SPB28.mb.30cbin.1	48.60	Clostridiales	15748	2565071
Sample_SPB28.Cluster6615cbin.1	42.40	Clostridiales	11429	2485855
Sample_SPB28.Cluster7542	41.00	Clostridiales	80107	1989659
Sample_SPB28.Cluster6082	44.20	Clostridiales	277886	2691609
Sample_SPB28.mb.19	54.80	Bacteroidetes	42927	2124220
Sample_SPB28.Cluster9014cbin.1	38.90	Clostridiales	73326	2123106
Sample_SPB28.Cluster5597cbin.1	41.40	Lachnospiraceae	21636	2768993
Sample_SPB28.mb.47	56.30	Clostridiales	15698	2416352
Sample_SPB28.mb.70cbin.1	54.30	Clostridiales	27419	1700088
Sample_SPB28.mb.57	60.60	Clostridiales	46263	2293754
Sample_SPB28.mb.78cbin.1	33.80	Bacteria	41572	2420141
Sample_SPB28.mb.35	44.80	Clostridiales	41086	2817243
Sample_SPB28.Cluster4850cbin.1	49.10	Bacteroidales	10654	3075883
Sample_SPB28.mb.56	37.60	Clostridiales	30302	2511863
Sample_SPB28.mb.13	48.60	Proteobacteria	38842	2556966
Sample_SPB28.mb.51cbin.1	31.30	Firmicutes	48094	2067557
Sample_SPB28.mb.71cbin.1	43.40	Lachnospiraceae	92485	3080962
Sample_SPB28.mb.89cbin.1	56.70	Bifidobacteriaceae	30920	2039597
Sample_SPB28.mb.65	39.50	Pasteurellaceae	49308	1875575
Sample_SPB28.Cluster4528cbin.1	45.60	Bacteroidales	15494	3916207
Sample_SPB28.mb.32cbin.1	43.30	Bacteria	9840	3781860
Sample_SPB28.Cluster35	58.00	Enterobacteriaceae	110649	4831559
Sample_SPB30.Cluster3079cbin.1	45.10	Bacteria	10593	4274136
Sample_SPB30.Cluster4134	49.40	Clostridiales	96015	1976242
Sample_SPB30.Cluster814cbin.1	58.00	Clostridiales	62816	3012625
Sample_SPB30.Cluster9210	37.80	Clostridiales	90067	1854831
Sample_SPB30.Cluster3409cbin.1	48.00	Firmicutes	35228	1633438
Sample_SPB30.Cluster2181	54.90	Bacteroidetes	168681	1925907
Sample_SPB30.mb.19	59.50	Actinobacteria	103828	1656974
Sample_SPB30.mb.68	43.50	Clostridiales	5229	1818561
Sample_SPB30.Cluster3620	51.10	Clostridiales	316753	2228477
Sample_SPB30.mb.27cbin.1	58.80	Deltaproteobacteria	28417	2427290
Sample_SPB30.Cluster1481cbin.1	59.50	Bifidobacteriaceae	21303	2109618
Sample_SPB30.Cluster10678	32.70	Lactobacillales	45553	1806439
Sample_SPB30.Cluster422cbin.1	60.40	Bifidobacteriaceae	125212	1889943
Sample_SPB30.mb.22	58.40	Bacteroidetes	355189	2357254
Sample_SPB30.mb.35	41.10	Clostridiales	9895	1897697
Sample_SPB30.mb.34cbin.1	55.80	Clostridiales	13602	2385790
Sample_SPB30.mb.47cbin.1	60.90	Clostridiales	57249	2827986
Sample_SPB30.mb.70	62.80	Clostridiales	13536	1684892
Sample_SPB30.mb.5cbin.1	50.60	Clostridiales	28575	2507706
Sample_SPB30.mb.43	60.70	Clostridiales	12855	2359832
Sample_SPB30.mb.29	55.70	Bacteria	202254	2737149
Sample_SPB30.mb.80	59.20	Clostridiales	19975	2004257
Sample_SPB30.mb.31	45.50	Prevotella	28273	3019849
Sample_SPB30.mb.26	36.00	Clostridiales	68981	2543660
Sample_SPB30.mb.57cbin.1	59.40	Bacteroidetes	230334	2625000
Sample_SPB30.mb.58cbin.1	46.90	Proteobacteria	135688	1888030
Sample_SPB30.mb.41cbin.1	43.70	Lachnospiraceae	41989	3031369
Sample_SPB30.Cluster8968	37.30	Lactobacillales	40850	2719397
Sample_SPB30.Cluster4912cbin.1	43.30	Bacteria	54527	4165177
Sample_SPB30.mb.74	54.40	Bacteroidetes	323173	2614239
Sample_SPB30.mb.46cbin.1	59.30	Bacteroidetes	149383	2716624
Sample_SPB30.mb.81cbin.1	38.90	Clostridiales	76356	2023654
Sample_SPB30.mb.92	55.60	Clostridiales	58130	3338083
Sample_SPB30.mb.72	36.70	Lactobacillales	85236	1955979
Sample_SPB30.mb.93	44.20	Clostridiales	9877	2786430

Sample_SPB30.mb.9	44.00	Clostridiales	230339	2813418
Sample_SPB30.mb.94cbin.1	44.60	Clostridiales	20682	2549548
Sample_SPB30.mb.53	45.50	Bacteroidales	199485	3852797
Sample_SPB30.mb.10cbin.1	46.10	Bacteroidales	27535	4665134
Sample_SPB31.Cluster13260	24.20	Bacteria	18753	882658
Sample_SPB31.Cluster3560	49.40	Clostridiales	95773	1991124
Sample_SPB31.mb.50cbin.1	59.70	Bacteria	26562	2276016
Sample_SPB31.Cluster2308cbin.1	51.70	Clostridiales	10822	2022857
Sample_SPB31.mb.21	45.20	Clostridiales	153090	1767127
Sample_SPB31.Cluster89	61.90	Clostridiales	52947	1603996
Sample_SPB31.mb.4	49.60	Clostridiales	79228	2039356
Sample_SPB31.Cluster4714	47.10	Selenomonadales	79631	2278989
Sample_SPB31.mb.19	55.30	Bacteroidetes	30474	1726102
Sample_SPB31.mb.46	57.10	Clostridiales	37402	2376553
Sample_SPB31.mb.72cbin.1	53.90	Clostridia	14599	1789929
Sample_SPB31.mb.1	43.80	Clostridiales	145559	1779266
Sample_SPB31.mb.76	50.00	Clostridia	61573	1875219
Sample_SPB31.Cluster10043cbin.1	37.80	Clostridiales	8009	2255975
Sample_SPB31.mb.15	46.70	Lachnospiraceae	32686	2857504
Sample_SPB31.Cluster6381	44.50	Clostridiales	161144	2353454
Sample_SPB31.Cluster13048	32.50	Lactobacillales	92183	1908482
Sample_SPB31.Cluster291	60.40	Bifidobacteriaceae	83818	1910520
Sample_SPB31.mb.63	57.10	Clostridiales	12479	2253661
Sample_SPB31.mb.40	41.10	Clostridiales	39427	1879251
Sample_SPB31.mb.27cbin.1	51.90	Clostridiales	11816	1920416
Sample_SPB31.mb.86	53.30	Clostridiales	43182	1814198
Sample_SPB31.mb.64	56.90	Clostridiales	19678	2714385
Sample_SPB31.mb.25	46.30	Prevotella	41166	2591597
Sample_SPB31.Cluster8738	40.90	Lachnospiraceae	83316	2951172
Sample_SPB31.mb.107cbin.1	40.80	Bacteroidales	36294	2822587
Sample_SPB31.mb.42	43.00	Clostridiales	44758	2539019
Sample_SPB31.mb.90	44.90	Bacteroidales	13843	2208751
Sample_SPB31.mb.85cbin.1	44.40	Clostridiales	15020	2526712
Sample_SPB31.mb.93	45.10	Clostridiales	29693	2315762
Sample_SPB31.mb.23	27.90	Clostridiales	75047	3649376
Sample_SPB31.Cluster5530	44.80	Bacteroidales	138604	4942754
Sample_SPB31.mb.78	43.50	Bacteroidales	288270	3096668
Sample_SPB31.mb.41	41.80	Bacteroidales	43583	4706091
Sample_SPB31.Cluster35cbin.1	57.10	Enterobacteriaceae	139460	5407355
Sample_SPB32.mb.100	47.50	Bacteria	62212	1608376
Sample_SPB32.Cluster6265cbin.1	33.50	Clostridiales	360746	2178029
Sample_SPB32.mb.13cbin.1	38.30	Clostridiales	83080	2009641
Sample_SPB32.mb.11	40.80	Clostridiales	68472	2522700
Sample_SPB32.Cluster3095cbin.1	44.70	Clostridiales	17823	2486070
Sample_SPB32.Cluster2891cbin.1	45.10	Lachnospiraceae	132331	2405715
Sample_SPB32.Cluster4348cbin.1	40.40	Clostridiales	235960	2121590
Sample_SPB32.Cluster11cbin.1	59.10	Clostridiales	34108	2483303
Sample_SPB32.Cluster142	57.80	Proteobacteria	20218	2106450
Sample_SPB32.mb.95cbin.1	48.80	Clostridiales	15706	2071395
Sample_SPB32.Cluster110	60.40	Bifidobacteriaceae	147632	1905736
Sample_SPB32.mb.18	46.80	Lachnospiraceae	64669	3357183
Sample_SPB32.Cluster161cbin.1	59.70	Bacteroidetes	148051	2736345
Sample_SPB32.mb.53cbin.1	59.20	Clostridiales	17697	2203414
Sample_SPB32.Cluster2789	43.90	Clostridiales	58731	3266418
Sample_SPB32.Cluster5551cbin.1	29.70	Bacteria	56108	2525638
Sample_SPB32.mb.47	49.60	Clostridiales	26678	2775407
Sample_SPB32.Cluster4022cbin.1	39.50	Pasteurellaceae	5912	1500434
Sample_SPB32.mb.5	42.20	Clostridiales	67930	2445227

Sample_SPB32.mb.104cbin.1	45.00	Clostridiales	41784	2687806
Sample_SPB32.Cluster3715	37.10	Clostridiales	140582	2786392
Sample_SPB32.mb.39	38.30	Lachnospiraceae	23473	2771159
Sample_SPB32.mb.93cbin.1	54.70	Bacteroidetes	126524	1995010
Sample_SPB32.mb.29	26.80	Clostridiales	65602	2623114
Sample_SPB32.mb.57cbin.1	45.00	Clostridiales	72011	2613952
Sample_SPB32.mb.70cbin.1	38.50	Clostridiales	128296	2726907
Sample_SPB32.mb.64	42.40	Lachnospiraceae	100719	3173847
Sample_SPB32.Cluster3691cbin.1	40.20	Lachnospiraceae	21704	4417208
Sample_SPB32.Cluster1224cbin.1	45.60	Bacteroidales	23680	3865028
Sample_SPB32.mb.86cbin.1	44.90	Clostridiales	61962	3675108
Sample_SPB32.mb.96cbin.1	40.10	Lachnospiraceae	127780	3606449
Sample_SPB32.Cluster1850cbin.1	46.30	Bacteroidales	172786	4373665
Sample_SPB32.mb.107cbin.1	45.00	Bacteroidales	180303	4536245
Sample_SPB32.mb.78cbin.1	42.60	Bacteroidales	16564	3763253
Sample_SPB34.Cluster8235	41.00	Bacteria	94634	3060013
Sample_SPB34.Cluster2119cbin.1	49.50	Bacteroidales	50447	2440203
Sample_SPB34.Cluster219cbin.1	58.00	Clostridiales	14945	2982913
Sample_SPB34.Cluster8435	41.20	Clostridiales	117192	1362830
Sample_SPB34.Cluster11cbin.1	64.30	Deltaproteobacteria	6906	1995324
Sample_SPB34.Cluster1214cbin.1	54.10	Prevotella	59709	2492087
Sample_SPB34.Cluster5423cbin.1	44.50	Clostridiales	22077	2520221
Sample_SPB34.mb.10	54.90	Bacteroidetes	50579	1989307
Sample_SPB34.mb.63	34.20	Clostridiales	23837	2129315
Sample_SPB34.mb.103cbin.1	51.20	Clostridiales	75019	2026760
Sample_SPB34.Cluster1265cbin.1	54.80	Proteobacteria	104679	2507124
Sample_SPB34.Cluster6727cbin.1	44.00	Clostridiales	360313	2872563
Sample_SPB34.mb.43	60.10	Actinobacteria	42398	1932106
Sample_SPB34.Cluster9258	37.70	Clostridiales	9091	2157546
Sample_SPB34.Cluster6494cbin.1	43.00	Clostridiales	68434	2676486
Sample_SPB34.mb.58cbin.1	57.90	Clostridiales	43384	2212066
Sample_SPB34.Cluster6796cbin.1	41.50	Lachnospiraceae	34687	2804340
Sample_SPB34.mb.71	58.80	Clostridiales	76002	2140030
Sample_SPB34.Cluster2430cbin.1	46.70	Lachnospiraceae	28913	7078483
Sample_SPB34.mb.105	72.70	Actinomycetaceae	11513	2804838
Sample_SPB34.Cluster11702	28.90	Bacteria	82996	2653438
Sample_SPB34.mb.107	44.90	Bacteroidales	40740	2824707
Sample_SPB34.mb.8cbin.1	60.00	Clostridiales	6963	1903901
Sample_SPB34.mb.90	50.80	Clostridiales	37417	2124362
Sample_SPB34.Cluster9277cbin.1	43.10	Bacteria	74378	4091295
Sample_SPB34.mb.96cbin.1	48.30	Bacteroidales	48712	2456538
Sample_SPB34.mb.52	43.30	Bacteroidales	27091	2973105
Sample_SPB34.mb.80	60.30	Bacteroidetes	25876	2645189
Sample_SPB34.mb.60	49.70	Lachnospiraceae	53340	2948290
Sample_SPB34.mb.6	43.10	Lachnospiraceae	17872	3147250
Sample_SPB34.mb.2cbin.1	45.30	Bacteroidales	12812	4022882
Sample_SPB34.mb.78cbin.1	43.30	Lachnospiraceae	55315	2684295
Sample_SPB34.mb.91	42.00	Lachnospiraceae	24808	2300955
Sample_SPB34.mb.72cbin.1	45.50	Bacteroidales	55463	3951236
Sample_SPB35.Cluster13733	23.90	Bacteria	43709	946009
Sample_SPB35.Cluster13575	25.20	Bacteria	95002	1307457
Sample_SPB35.Cluster2519	49.30	Bacteria	18580	1703038
Sample_SPB35.Cluster10159	37.60	Clostridiales	112938	2191235
Sample_SPB35.mb.29	28.10	Bacteria	37057	1182743
Sample_SPB35.Cluster9089	41.90	Clostridiales	114824	2351623
Sample_SPB35.mb.118cbin.1	47.30	Clostridiales	272365	1570365
Sample_SPB35.mb.24cbin.1	53.80	Clostridia	11411	1582790
Sample_SPB35.mb.112	41.70	Clostridiales	156375	1881620

Sample_SPB35.Cluster5949cbin.1	56.50	Clostridiales	74600	2004785
Sample_SPB35.Cluster902cbin.1	53.60	Clostridiales	9477	1814117
Sample_SPB35.mb.107	61.20	Clostridiales	47106	1674337
Sample_SPB35.mb.47	26.90	Bacteria	42183	1287146
Sample_SPB35.Cluster47cbin.1	59.80	Actinobacteria	79322	2181392
Sample_SPB35.Cluster6306	45.80	Lachnospiraceae	63016	2613686
Sample_SPB35.Cluster932cbin.1	51.80	Bacteria	6470	4565701
Sample_SPB35.mb.114cbin.1	56.20	Clostridiales	55253	2660764
Sample_SPB35.Cluster11811cbin.1	27.20	Clostridiales	17829	2476429
Sample_SPB35.Cluster134	55.60	Bacteria	74936	2678948
Sample_SPB35.Cluster2001	54.40	Proteobacteria	75521	2233374
Sample_SPB35.Cluster9269	40.70	Clostridiales	125866	1805040
Sample_SPB35.mb.85cbin.1	27.40	Bacteria	141095	1188476
Sample_SPB35.Cluster495cbin.1	59.70	Bifidobacteriaceae	279079	2230826
Sample_SPB35.mb.106	59.10	Clostridiales	10094	2169528
Sample_SPB35.mb.17cbin.1	58.90	Clostridiales	37036	2589666
Sample_SPB35.Cluster9599	40.60	Clostridiales	85666	2675079
Sample_SPB35.mb.110	45.90	Lachnospiraceae	79905	2755046
Sample_SPB35.mb.100	30.80	Euryarchaeota	140108	1815562
Sample_SPB35.mb.54cbin.1	43.60	Clostridiales	13960	2808326
Sample_SPB35.mb.30	37.30	Clostridiales	31286	2166113
Sample_SPB35.mb.83	52.80	Clostridiales	8949	1756496
Sample_SPB35.mb.25cbin.1	46.90	Clostridiales	32527	2556912
Sample_SPB35.mb.8cbin.1	50.90	Clostridiales	84470	1688915
Sample_SPB35.mb.76cbin.1	37.10	Clostridiales	159452	2568252
Sample_SPB35.mb.45cbin.1	56.70	Clostridiales	63063	2370136
Sample_SPB35.mb.95	59.90	Clostridiales	103977	2043046
Sample_SPB35.mb.57cbin.1	46.90	Clostridiales	27065	1493162
Sample_SPB35.mb.49cbin.1	43.70	Clostridiales	22612	1867124
Sample_SPB35.mb.67cbin.1	36.90	Clostridiales	102182	2192054
Sample_SPB35.mb.41cbin.1	40.80	Clostridiales	20126	2868540
Sample_SPB35.mb.82cbin.1	51.60	Clostridiales	22774	2081427
Sample_SPB35.mb.66cbin.1	56.80	Bacteria	13046	2912267
Sample_SPB35.Cluster428	57.50	Enterobacteriaceae	243039	5169987
Sample_SPB36.Cluster48cbin.1	60.30	Clostridiales	7591	1943804
Sample_SPB36.mb.102	56.20	Clostridiales	13796	1672391
Sample_SPB36.mb.51	27.20	Bacteria	77015	1348553
Sample_SPB36.mb.39	53.10	Clostridiales	12055	1754196
Sample_SPB36.mb.4	32.80	Bacteria	152039	1924560
Sample_SPB36.mb.57cbin.1	58.00	Clostridiales	33289	3089274
Sample_SPB36.mb.74cbin.1	52.90	Clostridia	68276	2090455
Sample_SPB36.Cluster4543cbin.1	52.50	Firmicutes	15662	2034365
Sample_SPB36.Cluster3595	54.70	Bacteroidetes	146976	2013005
Sample_SPB36.Cluster4053cbin.1	53.60	Clostridiales	167505	2073051
Sample_SPB36.mb.50	56.60	Clostridiales	69492	2164468
Sample_SPB36.Cluster3243cbin.1	55.60	Bacteria	119712	2581998
Sample_SPB36.Cluster136cbin.1	58.90	Clostridiales	76856	2863458
Sample_SPB36.mb.58	56.60	Clostridiales	24954	2152355
Sample_SPB36.mb.15	51.20	Clostridiales	184046	2227956
Sample_SPB36.Cluster977	60.40	Bifidobacteriaceae	100913	1907705
Sample_SPB36.mb.76	61.60	Clostridiales	26584	1781267
Sample_SPB36.mb.23cbin.1	59.50	Bacteroidetes	88577	2781143
Sample_SPB36.mb.85	56.40	Clostridiales	38475	2749430
Sample_SPB36.mb.37	37.40	Clostridiales	37706	2736387
Sample_SPB36.mb.38	41.30	Lachnospiraceae	105786	2741189
Sample_SPB36.mb.67	44.90	Lachnospiraceae	21722	2085131
Sample_SPB36.mb.9cbin.1	39.40	Clostridiales	95811	2111525
Sample_SPB36.mb.106	46.50	Bacteroidales	154229	3797459

Sample_SPB36.mb.5	41.80	Bacteroides	80895	4603485
Sample_SPB36.mb.63cbin.1	42.00	Bacteroidales	47999	4737344
Sample_SPB36.Cluster9234cbin.1	50.70	Enterobacteriaceae	113134	4629981
Sample_SPB37.Cluster5612cbin.1	46.00	Bacteria	80127	3757856
Sample_SPB37.mb.131	59.90	Bacteria	93298	2433666
Sample_SPB37.Cluster444cbin.1	60.30	Clostridiales	123932	2006858
Sample_SPB37.mb.18	47.10	Clostridiales	94158	2047830
Sample_SPB37.mb.10	62.80	Clostridiales	40944	1748201
Sample_SPB37.mb.109	61.20	Clostridiales	130432	1760549
Sample_SPB37.Cluster11531	33.40	Bacteria	40406	2333487
Sample_SPB37.Cluster219cbin.1	60.00	Actinobacteria	57493	2118160
Sample_SPB37.mb.107	61.00	Clostridiales	66361	2150089
Sample_SPB37.Cluster3269cbin.1	54.30	Bacteroidetes	73355	2149431
Sample_SPB37.Cluster10324	41.00	Clostridiales	189904	2089778
Sample_SPB37.mb.103cbin.1	58.70	Clostridiales	20435	2372947
Sample_SPB37.mb.32	58.50	Clostridiales	14206	2774055
Sample_SPB37.Cluster4525	51.40	Clostridiales	199321	2224342
Sample_SPB37.Cluster285cbin.1	60.50	Bifidobacteriaceae	60391	1720147
Sample_SPB37.Cluster57cbin.1	63.70	Deltaproteobacteria	29674	2561835
Sample_SPB37.Cluster7811	44.00	Clostridiales	227078	1873571
Sample_SPB37.mb.141cbin.1	46.90	Lactobacillales	77833	1975951
Sample_SPB37.mb.142	44.90	Clostridiales	17479	2496613
Sample_SPB37.Cluster1449cbin.1	56.60	Bifidobacteriaceae	48999	2034897
Sample_SPB37.Cluster11556cbin.1	38.60	Streptococcus	121419	1858490
Sample_SPB37.Cluster399cbin.1	59.90	Bifidobacteriaceae	45457	2489144
Sample_SPB37.mb.127	56.40	Clostridiales	11950	2271524
Sample_SPB37.mb.105	44.80	Clostridiales	43893	2376003
Sample_SPB37.mb.112cbin.1	32.60	Lactobacillales	88341	1910217
Sample_SPB37.mb.29cbin.1	44.60	Clostridiales	71914	2790245
Sample_SPB37.mb.102	42.30	Lachnospiraceae	13858	2099578
Sample_SPB37.mb.30cbin.1	59.60	Bifidobacteriaceae	71924	1940881
Sample_SPB37.mb.17cbin.1	59.70	Bacteroidetes	25766	3165435
Sample_SPB37.mb.63	58.50	Clostridiales	69383	1960806
Sample_SPB37.mb.44cbin.1	54.10	Clostridiales	16266	2140681
Sample_SPB37.mb.13cbin.1	59.20	Bacteroidetes	207044	2742316
Sample_SPB37.mb.76cbin.1	48.20	Clostridiales	27098	2745665
Sample_SPB37.mb.40cbin.1	42.30	Lachnospiraceae	12057	2245215
Sample_SPB37.Cluster6256cbin.1	48.80	Clostridiales	59517	6920853
Sample_SPB37.mb.82cbin.1	57.50	Bacteria	52670	2404083
Sample_SPB37.mb.73cbin.1	60.70	Clostridiales	23887	2238384
Sample_SPB37.mb.39	59.60	Proteobacteria	29631	2848414
Sample_SPB37.mb.52	59.10	Bacteroidetes	161201	2586190
Sample_SPB37.mb.90cbin.1	53.00	Clostridia	101678	2846438
Sample_SPB37.mb.8cbin.1	49.10	Bacteroidales	189833	3095314
Sample_SPB37.mb.69cbin.1	39.80	Streptococcus	24914	2081881
Sample_SPB37.mb.75cbin.1	46.10	Clostridiales	12586	2569984
Sample_SPB37.Cluster6931cbin.1	45.10	Bacteroidales	62780	4666635
Sample_SPB37.mb.62cbin.1	54.10	Selenomonadales	28405	2012570
Sample_SPB37.mb.42	42.00	Bacteroidales	52586	4317141
Sample_SPB37.mb.4cbin.1	45.30	Bacteroidales	150216	4160518
Sample_SPB38.Cluster33cbin.1	60.50	Clostridiales	18244	1939575
Sample_SPB38.mb.29cbin.1	61.40	Clostridiales	14348	1882038
Sample_SPB38.Cluster9738cbin.1	34.20	Bacteria	29420	2028578
Sample_SPB38.Cluster404	60.10	Actinobacteria	96568	2128226
Sample_SPB38.mb.32cbin.1	44.80	Clostridiales	13194	2389434
Sample_SPB38.mb.4	48.40	Clostridiales	18485	2813653
Sample_SPB38.Cluster1235cbin.1	54.60	Prevotella	50636	2428880
Sample_SPB38.Cluster8110	40.60	Clostridiales	249606	2091477

Sample_SPB38.Cluster6151cbin.1	43.30	Lachnospiraceae	13406	2115051
Sample_SPB38.Cluster2495	54.60	Bacteroidetes	222506	2894612
Sample_SPB38.mb.5	40.50	Clostridiales	16955	2702786
Sample_SPB38.mb.26cbin.1	37.80	Clostridiales	218967	2032240
Sample_SPB38.mb.38cbin.1	63.60	Actinobacteria	10751	2418833
Sample_SPB38.Cluster51cbin.1	60.40	Deltaproteobacteria	8820	3511101
Sample_SPB38.mb.78	59.50	Clostridiales	9493	1761309
Sample_SPB38.Cluster7246cbin.1	41.20	Lachnospiraceae	111391	2956083
Sample_SPB38.mb.81	54.10	Actinobacteria	176088	1647837
Sample_SPB38.mb.67cbin.1	55.40	Bacteroidetes	30280	1818042
Sample_SPB38.Cluster5633	42.60	Bacteroidales	62414	2792758
Sample_SPB38.mb.60	53.10	Clostridiales	69091	2261711
Sample_SPB38.mb.83	51.60	Clostridiales	25410	2070906
Sample_SPB38.Cluster6964	43.30	Bacteroidales	245369	3276599
Sample_SPB38.mb.10cbin.1	46.50	Bacteroidales	146122	4121618
Sample_SPB38.mb.41cbin.1	41.70	Lachnospiraceae	81435	2630749
Sample_SPB38.mb.7	37.40	Clostridiales	124815	2793037
Sample_SPB38.mb.75	59.50	Bacteroidetes	79983	2814223
Sample_SPB38.mb.33cbin.1	43.80	Bacteroidales	14578	3438135
Sample_SPB38.mb.51	41.70	Clostridiales	38728	3396874
Sample_SPB38.mb.34	45.30	Bacteroidales	20324	4098357
Sample_SPB38.mb.93cbin.1	60.10	Bifidobacteriaceae	34573	2213893
Sample_SPB38.mb.92cbin.1	39.80	Streptococcus	17713	2068442
Sample_SPB38.mb.79	41.90	Bacteroidales	39198	4319199
Sample_SPB38.mb.91cbin.1	45.20	Bacteroidales	83255	4168968
Sample_SPB39.Cluster13847cbin.1	26.00	Bacteria	92187	1331914
Sample_SPB39.mb.20cbin.1	23.90	Bacteria	64411	819420
Sample_SPB39.Cluster7601	45.20	Clostridiales	165676	1720333
Sample_SPB39.mb.27	49.30	Clostridiales	18747	1882641
Sample_SPB39.Cluster12504	33.30	Bacteria	10668	1846440
Sample_SPB39.mb.15cbin.1	32.10	Clostridiales	24098	2672006
Sample_SPB39.mb.102	52.40	Bacteroidales	31225	2058737
Sample_SPB39.mb.64	59.30	Clostridiales	13497	1611216
Sample_SPB39.Cluster2146cbin.1	52.30	Clostridiales	20556	2026076
Sample_SPB39.mb.73	34.10	Clostridiales	117641	2517359
Sample_SPB39.Cluster119	62.70	Proteobacteria	68872	2252876
Sample_SPB39.mb.72	57.40	Clostridiales	11877	2103151
Sample_SPB39.Cluster8360	43.60	Clostridiales	356916	2949742
Sample_SPB39.Cluster2005cbin.1	53.70	Bacteroidales	98343	2580093
Sample_SPB39.Cluster7507cbin.1	44.20	Clostridiales	181731	2746865
Sample_SPB39.Cluster7097cbin.1	44.60	Prevotella	15968	3439677
Sample_SPB39.mb.50	53.60	Clostridiales	152452	1844484
Sample_SPB39.mb.19	43.80	Clostridiales	8007	2705292
Sample_SPB39.mb.100	60.20	Bacteroidetes	8294	2386873
Sample_SPB39.mb.69	59.90	Actinobacteria	21235	2060630
Sample_SPB39.Cluster5922cbin.1	49.40	Lachnospiraceae	65673	2907165
Sample_SPB39.mb.65	54.50	Bacteroidetes	75835	2252352
Sample_SPB39.mb.75cbin.1	61.00	Clostridiales	10910	2214903
Sample_SPB39.mb.71cbin.1	38.30	Clostridiales	58335	2373918
Sample_SPB39.mb.70	59.70	Bacteroidetes	36108	2722590
Sample_SPB39.mb.94cbin.1	48.60	Clostridiales	23030	2650725
Sample_SPB39.Cluster5967cbin.1	43.10	Bacteria	13312	3826393
Sample_SPB39.Cluster3079cbin.1	46.10	Bacteroidales	7533	3450681
Sample_SPB39.mb.4cbin.1	40.90	Lachnospiraceae	69274	3060781
Sample_SPB39.mb.18cbin.1	41.70	Bacteroidales	52537	4003897
Sample_SPB39.mb.9cbin.1	48.80	Firmicutes	35186	1802402
Sample_SPB39.mb.6	43.60	Bacteroidales	65186	3046295
Sample_SPB39.mb.87	43.90	Lachnospiraceae	109858	2689569

Sample_SPB39.Cluster9689cbin.1	45.00	Bacteroidales	10007	4078457
Sample_SPB39.mb.104cbin.1	41.70	Bacteroidales	28090	4405857
Sample_SPB39.mb.97	37.50	Clostridiales	125427	2705815
Sample_SPB39.mb.78	48.10	Bacteroidales	107360	3429009
Sample_SPB41.Cluster6743	47.30	Clostridiales	365671	1535203
Sample_SPB41.Cluster10038cbin.1	37.60	Clostridiales	168980	2164182
Sample_SPB41.mb.20	53.80	Clostridia	69753	1671058
Sample_SPB41.Cluster1256	57.20	Clostridiales	66115	1841805
Sample_SPB41.Cluster220cbin.1	57.10	Clostridiales	16441	2283769
Sample_SPB41.Cluster3956cbin.1	51.30	Bacteroidetes	52452	3163039
Sample_SPB41.mb.16	58.40	Clostridiales	6379	2235593
Sample_SPB41.mb.100cbin.1	60.60	Clostridiales	26188	1719535
Sample_SPB41.mb.12	59.70	Clostridiales	73593	2385753
Sample_SPB41.Cluster11656	31.70	Euryarchaeota	45236	1579723
Sample_SPB41.Cluster6965	46.70	Lactobacillales	191845	2016415
Sample_SPB41.mb.71	26.00	Bacteria	107170	1419736
Sample_SPB41.Cluster6251	46.70	Selenomonadales	115041	2341215
Sample_SPB41.Cluster1709cbin.1	56.80	Bacteroidetes	179817	2632435
Sample_SPB41.Cluster1542	55.30	Bacteria	218542	2729258
Sample_SPB41.mb.55	58.70	Clostridiales	6091	1584113
Sample_SPB41.mb.63	58.50	Clostridiales	35635	2849744
Sample_SPB41.Cluster500cbin.1	59.00	Bacteroidetes	71356	3052892
Sample_SPB41.mb.81	49.60	Clostridiales	65403	1964142
Sample_SPB41.Cluster706	60.50	Bifidobacteriaceae	154960	1985822
Sample_SPB41.mb.27	34.70	Lactobacillus	12022	1684376
Sample_SPB41.mb.19	36.40	Clostridiales	9265	1884235
Sample_SPB41.mb.84	36.30	Clostridiales	78821	1750468
Sample_SPB41.mb.30cbin.1	51.50	Clostridiales	93676	1981272
Sample_SPB41.Cluster335cbin.1	59.30	Clostridiales	112868	3603115
Sample_SPB41.mb.54	60.20	Actinobacteria	38635	1940010
Sample_SPB41.mb.65cbin.1	56.80	Clostridiales	68988	1992896
Sample_SPB41.mb.25	44.10	Clostridiales	57635	2726652
Sample_SPB41.mb.62cbin.1	54.40	Bacteroidetes	58109	2281337
Sample_SPB41.Cluster6618cbin.1	43.10	Bacteria	30292	4160105
Sample_SPB41.mb.7	38.50	Streptococcus	120564	1811158
Sample_SPB41.mb.88cbin.1	32.50	Lactobacillales	28208	1795125
Sample_SPB41.mb.67cbin.1	36.90	Lactobacillales	29315	1977597
Sample_SPB41.Cluster7206	43.10	Bacteroidales	157614	4906192
Sample_SPB41.mb.51cbin.1	42.30	Bacteroidales	38311	4406111
Sample_SPB41.mb.96	42.10	Bacteria	79173	4446675
Sample_SPB41.Cluster756cbin.1	50.70	Enterobacteriaceae	32849	4724289
Sample_SPB42.Cluster18534	25.20	Bacteria	116143	1220648
Sample_SPB42.Cluster10349	45.40	Clostridia	111763	1689266
Sample_SPB42.mb.127	26.00	Bacteria	45716	1167654
Sample_SPB42.mb.104cbin.1	49.40	Clostridiales	112983	1936513
Sample_SPB42.mb.11cbin.1	53.30	Clostridia	15923	1833508
Sample_SPB42.mb.106	58.70	Clostridiales	7276	2465874
Sample_SPB42.Cluster10307	46.10	Clostridiales	96973	2133988
Sample_SPB42.Cluster17197cbin.1	34.50	Clostridiales	60858	4341667
Sample_SPB42.Cluster188cbin.1	59.90	Bacteria	55799	5126616
Sample_SPB42.Cluster89	61.60	Clostridiales	26834	1658490
Sample_SPB42.Cluster656cbin.1	58.50	Clostridiales	16520	2149491
Sample_SPB42.Cluster15277cbin.1	38.50	Clostridiales	70702	1571223
Sample_SPB42.Cluster65cbin.1	59.40	Clostridiales	16997	2163119
Sample_SPB42.mb.10	57.00	Clostridiales	21240	2263970
Sample_SPB42.mb.32cbin.1	53.70	Clostridia	23105	1734054
Sample_SPB42.Cluster10714	44.50	Clostridiales	136121	2171517
Sample_SPB42.Cluster582	60.40	Bifidobacteriaceae	136599	1921494

Sample_SPB42.mb.53	52.90	Clostridiales	15439	1917874
Sample_SPB42.Cluster15132cbin.1	41.40	Lachnospiraceae	55558	2855116
Sample_SPB42.mb.140	61.90	Clostridiales	34196	2365617
Sample_SPB42.Cluster877	55.50	Bacteria	98804	2717471
Sample_SPB42.mb.20	61.50	Clostridiales	21457	2143765
Sample_SPB42.Cluster75cbin.1	62.70	Proteobacteria	25152	2320996
Sample_SPB42.mb.41	61.90	Clostridiales	20091	1959467
Sample_SPB42.mb.15	54.80	Bacteroidetes	153234	2093116
Sample_SPB42.Cluster8875cbin.1	46.80	Lactobacillus	33237	2769590
Sample_SPB42.mb.72cbin.1	51.90	Clostridiales	10553	2063904
Sample_SPB42.mb.129	57.90	Bacteroidetes	144590	3428726
Sample_SPB42.mb.116	43.30	Bacteroidales	259733	3219778
Sample_SPB42.mb.69cbin.1	63.80	Actinobacteria	9640	2375349
Sample_SPB42.mb.94cbin.1	45.20	Clostridiales	12396	2047761
Sample_SPB42.mb.95cbin.1	51.10	Clostridiales	74216	2200982
Sample_SPB42.mb.75cbin.1	37.60	Clostridiales	19317	2513464
Sample_SPB42.mb.91cbin.1	41.60	Lachnospiraceae	37646	3172347
Sample_SPB42.Cluster2849cbin.1	56.10	Enterobacteriaceae	351103	4647577
Sample_SPB42.mb.19cbin.1	56.60	Enterobacteriaceae	24029	5117098
Sample_SPB45.Cluster12822	24.10	Bacteria	78572	969526
Sample_SPB45.Cluster12958cbin.1	26.40	Bacteria	145561	1169645
Sample_SPB45.Cluster12862	26.70	Bacteria	89262	1122934
Sample_SPB45.Cluster1279cbin.1	53.80	Clostridia	8901	1504019
Sample_SPB45.Cluster3400cbin.1	52.90	Clostridia	60519	2090399
Sample_SPB45.mb.22cbin.1	49.10	Clostridia	55163	1393962
Sample_SPB45.Cluster2056	54.90	Clostridiales	201270	2582299
Sample_SPB45.mb.26cbin.1	49.40	Clostridiales	86027	1986190
Sample_SPB45.mb.16	58.20	Clostridiales	56720	2972023
Sample_SPB45.mb.45	60.10	Clostridia	5695	2420508
Sample_SPB45.Cluster10521cbin.1	37.40	Clostridiales	16196	2171405
Sample_SPB45.mb.11	57.40	Clostridiales	27279	1862080
Sample_SPB45.mb.19	38.30	Clostridiales	16474	3016025
Sample_SPB45.Cluster4014cbin.1	51.30	Bacteroidetes	75612	3583945
Sample_SPB45.mb.39cbin.1	54.90	Clostridiales	15999	2461925
Sample_SPB45.Cluster7580cbin.1	44.90	Clostridiales	78778	2564344
Sample_SPB45.Cluster6181	45.60	Clostridiales	63073	2012883
Sample_SPB45.mb.107cbin.1	57.10	Clostridiales	94639	1938807
Sample_SPB45.mb.62	25.90	Bacteria	40610	1234120
Sample_SPB45.Cluster5347cbin.1	47.20	Selenomonadales	8229	2053094
Sample_SPB45.mb.48cbin.1	60.70	Clostridiales	52834	1745334
Sample_SPB45.mb.25	61.20	Clostridiales	40971	2198357
Sample_SPB45.Cluster3720cbin.1	53.20	Clostridiales	192701	2092538
Sample_SPB45.mb.104cbin.1	59.60	Clostridiales	6865	2185341
Sample_SPB45.Cluster12776	31.10	Euryarchaeota	185799	1778236
Sample_SPB45.mb.5	58.80	Clostridiales	11160	2049936
Sample_SPB45.Cluster2337	55.60	Bacteria	152668	2764446
Sample_SPB45.Cluster508	60.40	Bifidobacteriaceae	96352	1907902
Sample_SPB45.Cluster6995cbin.1	45.70	Clostridiales	49733	2606151
Sample_SPB45.mb.20cbin.1	40.60	Clostridiales	121126	2066425
Sample_SPB45.mb.76cbin.1	53.10	Clostridiales	190010	2194883
Sample_SPB45.Cluster25cbin.1	61.70	Clostridiales	23839	3223573
Sample_SPB45.mb.93	58.70	Clostridia	78218	2785959
Sample_SPB45.mb.80cbin.1	58.30	Clostridiales	38829	1837146
Sample_SPB45.mb.75cbin.1	55.10	Clostridiales	41648	3157530
Sample_SPB45.mb.60	53.80	Clostridiales	117227	1791664
Sample_SPB45.mb.98	59.40	Clostridiales	97113	2333759
Sample_SPB45.mb.81	40.20	Clostridiales	27401	2785048
Sample_SPB45.mb.82	59.60	Actinobacteria	69436	2226411

Sample_SPB45.mb.78	44.30	Clostridiales	120317	1996538
Sample_SPB45.mb.54	43.40	Bacteroidales	26735	2958099
Sample_SPB45.Cluster6357	45.20	Bacteroidales	76487	4306653
Sample_SPB45.mb.95cbin.1	56.40	Clostridiales	8923	2422570
Sample_SPB45.mb.55cbin.1	47.10	Bacteroidales	45897	3480621
Sample_SPB45.mb.41cbin.1	44.80	Bacteroidales	22148	4632603
Sample_SPB45.mb.49	49.70	Clostridiales	31074	5616546
Sample_SPB45.mb.87cbin.1	43.20	Bacteroidales	156681	5017111
Sample_SPB47.Cluster12751cbin.1	26.20	Bacteria	26739	1136895
Sample_SPB47.Cluster3739	50.00	Bacteria	348485	1479977
Sample_SPB47.Cluster2178cbin.1	47.60	Bacteria	74199	1787689
Sample_SPB47.Cluster12	61.20	Clostridiales	8645	1531444
Sample_SPB47.Cluster22cbin.1	59.60	Clostridiales	6796	1939463
Sample_SPB47.Cluster1652cbin.1	50.60	Clostridiales	24095	2414434
Sample_SPB47.Cluster1233cbin.1	53.20	Clostridiales	46922	2338461
Sample_SPB47.Cluster2232cbin.1	51.90	Clostridiales	45533	1982762
Sample_SPB47.Cluster2856	50.10	Clostridiales	111640	2306562
Sample_SPB47.mb.36	26.60	Bacteria	74609	1305736
Sample_SPB47.Cluster48cbin.1	60.60	Clostridiales	131440	2573394
Sample_SPB47.Cluster1049cbin.1	54.90	Bacteroidetes	109540	1913335
Sample_SPB47.Cluster1765	53.40	Clostridiales	330177	1814096
Sample_SPB47.Cluster366cbin.1	56.10	Clostridiales	14399	2296704
Sample_SPB47.Cluster5597	45.70	Selenomonadales	115418	1881540
Sample_SPB47.Cluster29cbin.1	63.70	Deltaproteobacteria	25439	2471151
Sample_SPB47.mb.11	57.60	Clostridiales	47874	2262299
Sample_SPB47.Cluster1190cbin.1	56.60	Bifidobacteriaceae	155428	2120032
Sample_SPB47.Cluster1024	55.30	Proteobacteria	223388	2401843
Sample_SPB47.mb.112	58.30	Clostridiales	31498	2615933
Sample_SPB47.mb.25cbin.1	48.90	Clostridiales	17361	2540043
Sample_SPB47.Cluster12280cbin.1	31.70	Firmicutes	60672	1822274
Sample_SPB47.Cluster8455cbin.1	40.80	Clostridiales	177049	2168943
Sample_SPB47.Cluster7105cbin.1	44.10	Clostridiales	157876	2780749
Sample_SPB47.Cluster10640cbin.1	38.40	Bacteroidales	24129	3179829
Sample_SPB47.mb.44	61.60	Clostridiales	13025	2146420
Sample_SPB47.Cluster10470	38.00	Lactobacillales	51570	2760674
Sample_SPB47.Cluster8504	41.20	Lachnospiraceae	108027	2891302
Sample_SPB47.mb.86	52.90	Clostridia	85009	2057066
Sample_SPB47.mb.122	28.70	Clostridiales	23886	2506397
Sample_SPB47.mb.14	61.10	Proteobacteria	82520	2471398
Sample_SPB47.mb.43cbin.1	49.70	Bacteroidales	9650	2583529
Sample_SPB47.mb.41	60.20	Clostridiales	43135	2251169
Sample_SPB47.mb.30cbin.1	58.90	Bifidobacteriaceae	11426	2031612
Sample_SPB47.mb.37	60.10	Bacteroidetes	21236	2583960
Sample_SPB47.mb.125	42.10	Lachnospiraceae	12982	2131714
Sample_SPB47.mb.99	33.50	Clostridiales	29938	2028726
Sample_SPB47.mb.57	59.50	Clostridiales	44201	1969757
Sample_SPB47.Cluster7084cbin.1	43.20	Bacteria	103672	4170608
Sample_SPB47.Cluster4651	45.10	Bacteroidales	91996	4350109
Sample_SPB47.mb.79cbin.1	49.30	Clostridiales	11896	2545551
Sample_SPB47.mb.27	40.80	Bacteroidales	33167	3282283
Sample_SPB47.mb.26cbin.1	42.10	Bacteroidales	62111	3691531
Sample_SPB47.mb.49	48.70	Bacteroidales	7334	2814180
Sample_SPB47.mb.88cbin.1	53.70	Clostridiales	14305	2115801
Sample_SPB47.mb.93	59.90	Bacteroidetes	151284	2802001
Sample_SPB47.mb.97cbin.1	58.90	Bacteroidetes	103286	2900241
Sample_SPB47.mb.98	43.30	Bacteroidales	93077	3011351
Sample_SPB47.mb.84cbin.1	42.90	Bacteria	45813	4610245
Sample_SPB47.mb.61cbin.1	44.90	Bacteroidales	29635	4785957

Sample_SPC03.Cluster14085	25.90	Bacteria	136763	1275095
Sample_SPC03.Cluster3141cbin.1	50.00	Bacteria	60165	2136569
Sample_SPC03.Cluster11152	37.80	Clostridiales	170203	2231976
Sample_SPC03.mb.117	52.80	Clostridia	62085	1939381
Sample_SPC03.Cluster257	61.20	Clostridiales	27353	1517029
Sample_SPC03.Cluster9900	41.80	Clostridiales	197354	2302333
Sample_SPC03.Cluster260cbin.1	59.50	Clostridiales	9821	1943504
Sample_SPC03.Cluster7720	42.80	Clostridiales	127365	2506866
Sample_SPC03.mb.24cbin.1	48.70	Clostridiales	12374	2378206
Sample_SPC03.mb.49	52.70	Clostridiales	214386	2049843
Sample_SPC03.mb.92cbin.1	58.50	Bacteria	19217	2772045
Sample_SPC03.mb.22cbin.1	48.70	Lachnospiraceae	10940	2632924
Sample_SPC03.Cluster10177	40.70	Clostridiales	171656	2661963
Sample_SPC03.mb.48	62.50	Clostridiales	28893	1737994
Sample_SPC03.Cluster5710cbin.1	45.90	Clostridiales	62286	2476003
Sample_SPC03.mb.103	41.60	Lachnospiraceae	20128	3282121
Sample_SPC03.mb.104cbin.1	54.40	Bacteroidetes	72819	2424990
Sample_SPC03.mb.6cbin.1	58.00	Clostridiales	59952	3201971
Sample_SPC03.mb.106	59.40	Bacteroidetes	86325	2748097
Sample_SPC03.Cluster8613cbin.1	43.00	Clostridiales	179532	2612365
Sample_SPC03.mb.35cbin.1	43.70	Selenomonadales	13641	2336817
Sample_SPC03.mb.44	60.80	Clostridiales	34648	2169663
Sample_SPC03.mb.113cbin.1	48.40	Bacteroidales	120013	3492007
Sample_SPC03.mb.97cbin.1	61.20	Clostridiales	38092	1855148
Sample_SPC03.mb.57	34.00	Clostridiales	51199	1825788
Sample_SPC03.mb.2cbin.1	43.30	Lachnospiraceae	112565	3032738
Sample_SPC03.mb.86cbin.1	45.00	Clostridiales	81252	2569810
Sample_SPC03.mb.114	41.50	Lachnospiraceae	71114	3283189
Sample_SPC03.mb.12	46.60	Bacteroidales	43088	4199084
Sample_SPC03.mb.46	43.30	Bacteroidales	13679	2981963
Sample_SPC03.Cluster708	57.90	Enterobacteriaceae	172750	5014539
Sample_SPC04.Cluster214cbin.1	61.00	Clostridiales	39535	1852884
Sample_SPC04.Cluster376cbin.1	58.60	Clostridiales	53821	2030156
Sample_SPC04.mb.6	52.50	Clostridia	68513	2299491
Sample_SPC04.Cluster1117cbin.1	56.90	Clostridiales	92085	2144360
Sample_SPC04.mb.13	54.10	Actinobacteria	175952	1639829
Sample_SPC04.mb.76	26.10	Bacteria	81950	1254449
Sample_SPC04.mb.80	26.80	Bacteria	77303	1190138
Sample_SPC04.Cluster5731cbin.1	45.40	Clostridiales	7682	2151617
Sample_SPC04.mb.54cbin.1	57.80	Clostridiales	7236	1874670
Sample_SPC04.mb.34	60.30	Actinobacteria	9206	1800935
Sample_SPC04.mb.78cbin.1	49.60	Clostridiales	85698	1819410
Sample_SPC04.Cluster5253	46.90	Selenomonadales	126839	2280852
Sample_SPC04.Cluster120cbin.1	60.90	Bifidobacteriaceae	6869	1527673
Sample_SPC04.Cluster907cbin.1	57.30	Proteobacteria	20003	1918200
Sample_SPC04.mb.15cbin.1	63.60	Actinobacteria	16800	2678006
Sample_SPC04.mb.29cbin.1	54.20	Bacteroidetes	126174	2209986
Sample_SPC04.Cluster11760cbin.1	43.90	Clostridiales	111068	2774094
Sample_SPC04.Cluster8782	43.90	Clostridiales	191943	2775057
Sample_SPC04.mb.83cbin.1	58.10	Clostridiales	49464	3101593
Sample_SPC04.mb.22cbin.1	43.00	Prevotella	29864	3318643
Sample_SPC04.mb.47	37.80	Clostridiales	202346	1895224
Sample_SPC04.mb.59cbin.1	56.40	Clostridiales	12268	2470005
Sample_SPC04.Cluster5803cbin.1	44.20	Prevotella	13429	3535800
Sample_SPC04.mb.85	62.50	Clostridiales	35020	1984498
Sample_SPC04.Cluster3414cbin.1	55.50	Proteobacteria	11162	2532757
Sample_SPC04.mb.65cbin.1	50.50	Lachnospiraceae	8598	2404032
Sample_SPC04.mb.93	59.10	Bacteroidetes	5341	1988323

Sample_SPC04.mb.82cbin.1	51.70	Clostridiales	198401	2101425
Sample_SPC04.mb.87	54.70	Bacteroidetes	619008	2548926
Sample_SPC04.mb.81	43.60	Lachnospiraceae	28084	2921042
Sample_SPC04.mb.89	35.70	Clostridiales	72972	2874600
Sample_SPC04.mb.62cbin.1	27.90	Clostridiales	229902	3251121
Sample_SPC04.mb.69cbin.1	45.30	Bacteroidales	40175	4019238
Sample_SPC04.mb.88cbin.1	45.00	Bacteroidales	64944	4823707
Sample_SPC04.mb.92cbin.1	50.70	Enterobacteriaceae	128118	4603732
Sample_SPC06.Cluster3248cbin.1	49.40	Clostridia	123425	1515389
Sample_SPC06.Cluster1387cbin.1	53.30	Clostridia	39280	2067376
Sample_SPC06.mb.44cbin.1	27.30	Bacteria	632868	1104318
Sample_SPC06.Cluster694	58.30	Clostridiales	69104	2870881
Sample_SPC06.Cluster5415	41.80	Clostridiales	161610	2291717
Sample_SPC06.Cluster328cbin.1	57.80	Clostridiales	47468	2764307
Sample_SPC06.mb.48	61.90	Clostridiales	53859	2106449
Sample_SPC06.Cluster1832cbin.1	54.30	Clostridiales	209233	1724389
Sample_SPC06.mb.55	44.30	Clostridiales	68959	2752342
Sample_SPC06.mb.71cbin.1	61.20	Clostridiales	70166	1849043
Sample_SPC06.Cluster1121	52.40	Clostridiales	208242	1996185
Sample_SPC06.Cluster5959cbin.1	38.50	Clostridiales	117374	2020603
Sample_SPC06.mb.21cbin.1	46.00	Clostridiales	11969	2991236
Sample_SPC06.mb.13	40.80	Clostridiales	296856	1994691
Sample_SPC06.mb.8	49.80	Clostridiales	75742	2697495
Sample_SPC06.mb.74	58.80	Clostridiales	105292	2073213
Sample_SPC06.mb.78cbin.1	57.10	Clostridiales	60288	2138243
Sample_SPC06.mb.35	45.90	Clostridiales	12105	2188631
Sample_SPC06.Cluster1414	56.40	Bifidobacteriaceae	260290	2152961
Sample_SPC06.Cluster5280cbin.1	42.80	Lachnospiraceae	41390	2584680
Sample_SPC06.mb.86	56.60	Clostridiales	48867	2218837
Sample_SPC06.mb.81	41.00	Lachnospiraceae	40876	2784782
Sample_SPC06.mb.72cbin.1	56.30	Clostridiales	16390	2612311
Sample_SPC06.mb.59cbin.1	58.40	Bacteria	28201	2874987
Sample_SPC06.mb.65	50.90	Clostridiales	48239	2493101
Sample_SPC06.mb.58cbin.1	44.00	Clostridiales	15608	2616764
Sample_SPC06.mb.68cbin.1	59.90	Bacteroidetes	46448	2734681
Sample_SPC06.mb.27	41.90	Lachnospiraceae	60364	2525863
Sample_SPC06.Cluster4605	45.20	Bacteroidales	200827	4192553
Sample_SPC06.Cluster1874cbin.1	45.30	Bacteroidales	11002	4087168
Sample_SPC06.mb.38	47.00	Bacteroidales	25864	3656544
Sample_SPC07.Cluster12284	29.80	Bacteria	28150	2445658
Sample_SPC07.mb.117cbin.1	25.20	Bacteria	96125	1024301
Sample_SPC07.Cluster12882	32.30	Clostridiales	186627	2646836
Sample_SPC07.Cluster7505cbin.1	43.70	Clostridiales	10690	2437325
Sample_SPC07.Cluster187cbin.1	61.20	Clostridiales	110705	2035991
Sample_SPC07.Cluster5885cbin.1	48.60	Clostridiales	44936	2803520
Sample_SPC07.Cluster8839cbin.1	41.40	Clostridiales	6270	2941896
Sample_SPC07.Cluster82cbin.1	61.40	Clostridiales	24392	1836357
Sample_SPC07.Cluster907	58.10	Clostridiales	49518	1830295
Sample_SPC07.Cluster598cbin.1	58.10	Clostridiales	49834	2754068
Sample_SPC07.Cluster923cbin.1	56.80	Clostridiales	46306	2529899
Sample_SPC07.Cluster7094	45.30	Selenomonadales	140490	1981379
Sample_SPC07.Cluster4915cbin.1	48.60	Firmicutes	33738	1918340
Sample_SPC07.mb.107	55.40	Bacteroidales	22927	2578336
Sample_SPC07.mb.12	51.90	Clostridiales	18535	2019258
Sample_SPC07.Cluster3423	54.70	Bacteroidetes	221228	2868039
Sample_SPC07.mb.4	26.60	Bacteria	159125	1330363
Sample_SPC07.Cluster120	60.10	Bifidobacteriaceae	87863	2286855
Sample_SPC07.Cluster2242	55.50	Proteobacteria	161345	2278192

Sample_SPC07.Cluster12260	32.80	Lactobacillales	334979	1895779
Sample_SPC07.mb.120	46.90	Lachnospiraceae	56403	2473569
Sample_SPC07.mb.32cbin.1	48.90	Clostridiales	161931	2083020
Sample_SPC07.Cluster11122	37.20	Clostridiales	136702	2826365
Sample_SPC07.mb.108	40.70	Clostridiales	217424	2017874
Sample_SPC07.Cluster8152cbin.1	44.10	Clostridiales	167962	2662939
Sample_SPC07.mb.18	54.70	Clostridiales	32282	2573208
Sample_SPC07.mb.41	58.40	Clostridiales	59105	2873161
Sample_SPC07.mb.118cbin.1	53.60	Clostridiales	53408	2189369
Sample_SPC07.Cluster12891	31.30	Firmicutes	53966	2377190
Sample_SPC07.mb.25	59.20	Clostridiales	15692	2177532
Sample_SPC07.Cluster8463	43.50	Bacteroidales	192320	3023405
Sample_SPC07.mb.93	49.50	Clostridiales	78068	1954408
Sample_SPC07.mb.30	54.30	Bacteroidetes	97538	2119001
Sample_SPC07.Cluster5320cbin.1	48.60	Bacteroidales	59635	3664538
Sample_SPC07.mb.42	59.60	Actinobacteria	118902	2139090
Sample_SPC07.mb.82cbin.1	60.90	Clostridiales	56410	1915419
Sample_SPC07.mb.85	47.10	Clostridiales	59628	1620196
Sample_SPC07.mb.8	57.00	Clostridiales	76461	1990261
Sample_SPC07.Cluster8650cbin.1	43.30	Bacteria	44163	4022717
Sample_SPC07.mb.23	42.10	Lachnospiraceae	43286	2304317
Sample_SPC07.mb.63	47.00	Clostridiales	27762	2009699
Sample_SPC07.mb.88cbin.1	53.70	Clostridiales	17446	2291151
Sample_SPC07.Cluster6991	45.40	Bacteroidales	94633	4255990
Sample_SPC07.mb.75	57.50	Clostridiales	12123	1817475
Sample_SPC07.mb.47	58.40	Clostridiales	33073	2302177
Sample_SPC07.mb.96	59.30	Bacteroidetes	124830	2801739
Sample_SPC07.mb.78cbin.1	49.10	Lachnospiraceae	86602	3080795
Sample_SPC07.mb.71	45.40	Bacteroidales	62542	3995957
Sample_SPC07.mb.95	58.70	Bacteroidetes	215479	3006495
Sample_SPC08.Cluster17317	26.40	Bacteria	89838	1090539
Sample_SPC08.Cluster17466	24.00	Bacteria	87371	969083
Sample_SPC08.Cluster160cbin.1	59.40	Clostridiales	79101	2159496
Sample_SPC08.Cluster3332cbin.1	54.90	Clostridia	131999	2838474
Sample_SPC08.Cluster1267	58.10	Clostridiales	112868	1974893
Sample_SPC08.mb.118	25.50	Bacteria	146661	1338112
Sample_SPC08.Cluster351cbin.1	59.30	Clostridiales	136739	2251397
Sample_SPC08.mb.10	58.10	Clostridiales	50728	2977041
Sample_SPC08.Cluster5062cbin.1	50.20	Clostridiales	218747	2173629
Sample_SPC08.Cluster315cbin.1	58.00	Bacteroidetes	7960	2027439
Sample_SPC08.Cluster219cbin.1	61.00	Clostridiales	16660	2203895
Sample_SPC08.Cluster14570cbin.1	38.00	Clostridiales	58719	1847889
Sample_SPC08.Cluster4847cbin.1	51.40	Clostridiales	126730	2040574
Sample_SPC08.mb.29	29.30	Bacteria	147827	1535129
Sample_SPC08.Cluster7170	45.90	Selenomonadales	324776	1941873
Sample_SPC08.mb.110	50.10	Clostridiales	50264	2022036
Sample_SPC08.Cluster7553	46.40	Bacteroidetes	287051	2364620
Sample_SPC08.Cluster15847	35.80	Clostridiales	207465	2558013
Sample_SPC08.Cluster588cbin.1	56.00	Proteobacteria	20004	2099610
Sample_SPC08.Cluster6706cbin.1	43.50	Selenomonadales	316579	2409582
Sample_SPC08.mb.113	54.40	Clostridiales	11569	2648533
Sample_SPC08.Cluster509	60.40	Bifidobacteriaceae	66692	1909429
Sample_SPC08.mb.11	46.30	Lachnospiraceae	82090	2666905
Sample_SPC08.mb.66	28.00	Bacteria	33617	1149135
Sample_SPC08.mb.54	29.40	Bacteria	55047	1818150
Sample_SPC08.Cluster9250	44.40	Clostridiales	138732	2265630
Sample_SPC08.Cluster15302cbin.1	40.60	Lachnospiraceae	71599	3410357
Sample_SPC08.mb.26	60.90	Clostridiales	76089	2025719

Sample_SPC08.Cluster9757	43.90	Clostridiales	198514	2804396
Sample_SPC08.mb.124	46.70	Lachnospiraceae	12240	2723013
Sample_SPC08.mb.127	59.10	Clostridiales	10551	2175795
Sample_SPC08.Cluster9827cbin.1	45.30	Prevotella	60593	3339606
Sample_SPC08.mb.93	26.70	Bacteria	92921	1132717
Sample_SPC08.Cluster5156cbin.1	46.90	Bacteroidales	42628	2996348
Sample_SPC08.mb.56	39.90	Clostridiales	6184	1616308
Sample_SPC08.mb.91	52.90	Clostridia	74474	2050943
Sample_SPC08.mb.58	56.10	Clostridiales	14568	2163126
Sample_SPC08.mb.49	51.00	Bacteria	102907	2209582
Sample_SPC08.mb.18cbin.1	59.90	Bacteroidetes	52988	2550209
Sample_SPC08.mb.79	36.50	Clostridiales	11512	2233548
Sample_SPC08.Cluster7435cbin.1	43.20	Bacteria	15170	3812151
Sample_SPC08.mb.4	40.70	Clostridiales	9899	1945737
Sample_SPC08.mb.76	56.50	Clostridiales	48438	2508229
Sample_SPC08.mb.51	54.60	Bacteroidetes	86233	2168076
Sample_SPC08.mb.55	63.40	Actinobacteria	32373	2670467
Sample_SPC08.Cluster7176cbin.1	46.10	Bacteroidales	130677	4305575
Sample_SPC08.mb.61	42.10	Lachnospiraceae	14642	2143108
Sample_SPC08.mb.9	44.80	Lachnospiraceae	83185	2894630
Sample_SPC08.mb.41	56.10	Enterobacteriaceae	17004	4569273
Sample_SPC09.Cluster31cbin.1	62.00	Clostridiales	9673	1562209
Sample_SPC09.Cluster1936cbin.1	55.50	Clostridiales	52694	2379470
Sample_SPC09.Cluster90	60.80	Clostridiales	55271	1633513
Sample_SPC09.Cluster8082cbin.1	41.20	Lachnospiraceae	86388	3478642
Sample_SPC09.Cluster2849cbin.1	48.70	Firmicutes	32301	1939965
Sample_SPC09.mb.12	49.10	Clostridiales	22186	2465841
Sample_SPC09.mb.33	46.80	Lachnospiraceae	77829	2648893
Sample_SPC09.mb.16	60.00	Clostridiales	51783	2206655
Sample_SPC09.Cluster5456	43.70	Selenomonadales	172774	2325988
Sample_SPC09.mb.29	40.90	Clostridiales	153460	1983742
Sample_SPC09.mb.69	41.10	Clostridiales	9537	3027331
Sample_SPC09.mb.67cbin.1	52.20	Clostridiales	73673	2313626
Sample_SPC09.Cluster2177cbin.1	48.90	Proteobacteria	6332	2011699
Sample_SPC09.Cluster5059cbin.1	43.40	Lachnospiraceae	100745	3021833
Sample_SPC09.mb.21	41.30	Clostridiales	216384	2657366
Sample_SPC09.Cluster6794cbin.1	40.50	Clostridiales	27099	2909433
Sample_SPC09.Cluster6170	41.30	Lachnospiraceae	116139	2856461
Sample_SPC09.Cluster6340cbin.1	43.40	Bacteroidales	317455	3231604
Sample_SPC09.mb.87cbin.1	53.70	Bacteroidetes	108964	2324595
Sample_SPC09.Cluster5921cbin.1	42.00	Bacteroidales	24068	4184248
Sample_SPC09.mb.82	42.80	Lachnospiraceae	92515	3253840
Sample_SPC09.Cluster4566cbin.1	47.90	Enterobacteriaceae	233134	4491581
Sample_SPC09.mb.83	46.30	Bacteroidales	176901	4385809
Sample_SPC09.mb.73cbin.1	45.20	Bacteroidales	39300	4726584
Sample_SPC10.Cluster2969	41.80	Clostridiales	95246	2649710
Sample_SPC10.mb.10	61.50	Clostridiales	191935	2036074
Sample_SPC10.Cluster27	62.20	Clostridiales	237465	1883809
Sample_SPC10.Cluster2394cbin.1	48.10	Clostridiales	55743	2815487
Sample_SPC10.Cluster1785	47.10	Lactobacillales	23603	1666945
Sample_SPC10.mb.20	49.50	Clostridiales	15966	2286244
Sample_SPC10.mb.26	49.00	Clostridiales	59543	2516989
Sample_SPC10.Cluster4267cbin.1	38.80	Clostridiales	96900	2113818
Sample_SPC10.mb.11	38.50	Selenomonadales	18101	1752837
Sample_SPC10.Cluster5577cbin.1	35.90	Clostridiales	106531	2388287
Sample_SPC10.mb.23cbin.1	57.00	Proteobacteria	426618	2553360
Sample_SPC10.mb.12cbin.1	61.50	Clostridiales	41392	3434189
Sample_SPC10.mb.49cbin.1	60.30	Clostridiales	41693	1948065

Sample_SPC10.Cluster4683cbin.1	41.20	Lachnospiraceae	90856	3162184
Sample_SPC10.mb.46cbin.1	57.20	Clostridiales	38343	2331010
Sample_SPC10.mb.73	58.80	Clostridiales	47126	2028333
Sample_SPC10.mb.2	28.40	Clostridiales	12197	3372848
Sample_SPC10.mb.34	43.80	Lachnospiraceae	19559	2727914
Sample_SPC10.mb.59cbin.1	48.40	Proteobacteria	18121	2526340
Sample_SPC10.Cluster2004cbin.1	46.00	Bacteroidales	166177	3396701
Sample_SPC10.mb.37	41.30	Clostridiales	43298	3484938
Sample_SPC10.mb.29cbin.1	46.50	Bacteroidales	103539	3660915
Sample_SPC10.mb.1cbin.1	44.90	Bacteroidales	21643	4526016
Sample_SPC10.Cluster3694cbin.1	41.30	Bacteroides	50838	5804466
Sample_SPC10.mb.62cbin.1	41.40	Bacteroidales	233826	5177610
Sample_SPC10.Cluster140	57.60	Enterobacteriaceae	226510	5332752
Sample_SPC11.Cluster4048	48.90	Clostridia	137216	2029114
Sample_SPC11.Cluster2715cbin.1	48.30	Clostridiales	12921	1825477
Sample_SPC11.Cluster288	60.50	Clostridiales	99176	1577842
Sample_SPC11.Cluster5831	41.70	Clostridiales	177747	2825549
Sample_SPC11.Cluster4863	45.30	Actinobacteria	245551	1930517
Sample_SPC11.Cluster134cbin.1	59.20	Deltaproteobacteria	11487	2170356
Sample_SPC11.Cluster10732cbin.1	33.40	Bacteria	31593	2403241
Sample_SPC11.Cluster160	59.80	Actinobacteria	101576	2221869
Sample_SPC11.Cluster294cbin.1	60.80	Clostridiales	18771	2271339
Sample_SPC11.Cluster4327cbin.1	47.00	Selenomonadales	71380	2223967
Sample_SPC11.Cluster1525cbin.1	53.90	Clostridiales	11254	1936872
Sample_SPC11.Cluster6659cbin.1	44.90	Prevotella	7312	2476490
Sample_SPC11.Cluster2946	51.70	Clostridiales	224275	2183225
Sample_SPC11.Cluster83cbin.1	58.40	Clostridiales	68142	2388908
Sample_SPC11.mb.104	62.50	Clostridiales	9629	2110166
Sample_SPC11.Cluster1285	55.60	Proteobacteria	150094	2232840
Sample_SPC11.Cluster1447cbin.1	59.60	Bacteroidetes	73033	2824640
Sample_SPC11.mb.119	48.40	Clostridiales	35371	2806547
Sample_SPC11.mb.133cbin.1	62.40	Clostridiales	38277	1903361
Sample_SPC11.Cluster8333	40.60	Clostridiales	175458	1999321
Sample_SPC11.Cluster6575	43.70	Lachnospiraceae	106824	2822123
Sample_SPC11.Cluster10755cbin.1	35.60	Clostridiales	49695	2760232
Sample_SPC11.mb.53	26.70	Bacteria	140798	1297900
Sample_SPC11.Cluster6755cbin.1	43.00	Clostridiales	147522	2498876
Sample_SPC11.Cluster10067cbin.1	36.70	Clostridiales	124127	3113755
Sample_SPC11.mb.105	42.50	Streptococcus	22574	1715223
Sample_SPC11.mb.41	57.10	Clostridiales	20069	2367049
Sample_SPC11.mb.116cbin.1	60.40	Bifidobacteriaceae	81868	1898433
Sample_SPC11.Cluster6184	43.50	Bacteroidales	271351	3266432
Sample_SPC11.mb.40cbin.1	47.30	Lactobacillales	10267	1580729
Sample_SPC11.mb.10	41.60	Lachnospiraceae	83157	2760543
Sample_SPC11.mb.114	43.80	Clostridiales	158634	2812922
Sample_SPC11.mb.24	54.20	Bacteroidetes	111865	2143327
Sample_SPC11.mb.76	32.70	Bacteria	124465	1901296
Sample_SPC11.Cluster2437cbin.1	42.10	Bacteroidales	34624	4605519
Sample_SPC11.mb.136	41.90	Clostridiales	48239	2956248
Sample_SPC11.mb.2	56.50	Bifidobacteriaceae	107227	2037400
Sample_SPC11.mb.51	38.50	Clostridiales	38793	4034840
Sample_SPC11.mb.46	47.10	Lachnospiraceae	19644	2635379
Sample_SPC11.mb.64	56.10	Clostridiales	64418	2540970
Sample_SPC11.mb.3cbin.1	56.20	Clostridiales	13899	2441779
Sample_SPC11.Cluster4045cbin.1	45.00	Bacteroidales	156211	4223253
Sample_SPC11.mb.75	57.50	Clostridiales	20163	2689400
Sample_SPC11.mb.70cbin.1	45.90	Clostridiales	133504	2131277
Sample_SPC11.mb.82cbin.1	58.90	Clostridiales	28123	1930758

Sample_SPC11.mb.47	59.40	Bacteroidetes	119813	2867519
Sample_SPC11.mb.56	30.00	Bacteria	5306	2019574
Sample_SPC11.mb.110	40.60	Bacteroidales	36280	3409697
Sample_SPC11.mb.6	58.40	Bacteroidetes	134645	2577852
Sample_SPC11.mb.44	36.60	Lactobacillales	23950	2069245
Sample_SPC11.mb.66	44.30	Clostridiales	48866	2574340
Sample_SPC11.mb.95cbin.1	63.00	Bacteroidetes	15073	2270058
Sample_SPC11.mb.37cbin.1	45.60	Bacteroidales	12790	4045552
Sample_SPC11.mb.99	46.10	Bacteroidales	89387	2959829
Sample_SPC11.mb.73cbin.1	44.10	Bacteroidales	72544	3567801
Sample_SPC11.mb.86cbin.1	46.50	Bacteroidales	89600	4216496
Sample_SPC11.mb.94	43.30	Bacteria	34724	3912491
Sample_SPC12.mb.1	60.00	Bacteria	25356	1938643
Sample_SPC12.Cluster15328cbin.1	37.20	Clostridiales	5523	2146266
Sample_SPC12.Cluster5908cbin.1	53.90	Clostridia	13353	1716697
Sample_SPC12.Cluster17469	27.70	Euryarchaeota	5894	1222930
Sample_SPC12.mb.103	26.60	Bacteria	100427	1290544
Sample_SPC12.Cluster480	60.00	Bacteria	74673	3206878
Sample_SPC12.mb.126	52.80	Clostridia	69518	2056828
Sample_SPC12.Cluster2893cbin.1	56.90	Bacteroidetes	184726	2121579
Sample_SPC12.Cluster6801cbin.1	49.90	Clostridiales	130146	2352852
Sample_SPC12.Cluster13456	41.70	Clostridiales	122547	2398350
Sample_SPC12.Cluster2319	56.80	Clostridiales	128879	2100377
Sample_SPC12.Cluster14444cbin.1	38.40	Clostridiales	56188	2186557
Sample_SPC12.Cluster13847cbin.1	37.60	Clostridiales	9794	2293487
Sample_SPC12.Cluster11106cbin.1	45.20	Prevotella	12353	3052891
Sample_SPC12.mb.36	26.30	Bacteria	128570	1248956
Sample_SPC12.mb.138	57.80	Clostridiales	6872	2124461
Sample_SPC12.mb.131	55.10	Clostridiales	6819	2219244
Sample_SPC12.mb.107	56.50	Clostridiales	14498	2360253
Sample_SPC12.mb.117cbin.1	45.00	Clostridiales	128617	1656992
Sample_SPC12.Cluster12531cbin.1	41.10	Lachnospiraceae	80140	2605141
Sample_SPC12.mb.32	36.40	Clostridiales	182411	1856400
Sample_SPC12.mb.114	60.10	Bacteroidetes	24643	2651385
Sample_SPC12.mb.122cbin.1	31.00	Euryarchaeota	164912	1753125
Sample_SPC12.mb.113cbin.1	42.60	Lachnospiraceae	42302	2365451
Sample_SPC12.mb.43	42.10	Clostridiales	24402	2427175
Sample_SPC12.mb.82cbin.1	29.00	Bacteria	70008	1841249
Sample_SPC12.mb.133cbin.1	60.00	Bifidobacteriaceae	31243	1981533
Sample_SPC12.mb.20	55.00	Bacteroidetes	100602	2059523
Sample_SPC12.mb.56cbin.1	68.10	Actinobacteria	25568	1875474
Sample_SPC12.mb.73	58.00	Clostridiales	39696	3012528
Sample_SPC12.mb.132	59.40	Bacteroidetes	27425	2713690
Sample_SPC12.mb.22	45.90	Clostridiales	52851	2342857
Sample_SPC12.mb.86	62.00	Clostridiales	31026	1413500
Sample_SPC12.mb.61	44.80	Clostridiales	36578	2532227
Sample_SPC12.mb.8	57.10	Clostridiales	45278	2303990
Sample_SPC12.Cluster9084cbin.1	45.30	Bacteroidales	9329	3933257
Sample_SPC12.mb.72cbin.1	59.10	Deltaproteobacteria	13650	2248058
Sample_SPC12.mb.53	42.30	Bacteroidales	66004	3051418
Sample_SPC12.mb.81	60.00	Bacteroidetes	61500	2661241
Sample_SPC12.mb.137	47.00	Bacteroidales	21377	3740119
Sample_SPC12.mb.23	41.30	Lachnospiraceae	57821	3708494
Sample_SPC12.mb.60cbin.1	43.90	Clostridiales	45135	2987042
Sample_SPC12.mb.54	43.70	Bacteroidales	135263	2953224
Sample_SPC12.mb.4cbin.1	44.70	Bacteroidales	89579	3082891
Sample_SPC12.mb.91cbin.1	43.20	Bacteria	27660	4012709
Sample_SPC13.Cluster1460cbin.1	48.20	Clostridia	26450	1892514

Sample_SPC13.Cluster7173	37.30	Clostridiales	9050	1897016
Sample_SPC13.Cluster8354	33.20	Clostridiales	61716	2186718
Sample_SPC13.Cluster7899cbin.1	35.90	Clostridiales	198469	2004584
Sample_SPC13.Cluster37cbin.1	59.50	Clostridiales	7034	2529349
Sample_SPC13.Cluster265cbin.1	60.40	Bifidobacteriaceae	89210	1851556
Sample_SPC13.Cluster3818cbin.1	43.50	Selenomonadales	138405	2528604
Sample_SPC13.mb.37cbin.1	46.80	Clostridiales	77062	1726463
Sample_SPC13.Cluster6624	40.60	Clostridiales	193412	2163052
Sample_SPC13.Cluster4518cbin.1	44.50	Clostridiales	114430	2564623
Sample_SPC13.Cluster1373	54.20	Proteobacteria	101610	2386308
Sample_SPC13.mb.3	58.60	Bacteroidetes	138544	2383413
Sample_SPC13.mb.103	31.30	Euryarchaeota	21951	1748688
Sample_SPC13.mb.14cbin.1	59.80	Bifidobacteriaceae	215500	2066587
Sample_SPC13.mb.47	59.90	Clostridiales	115063	2077960
Sample_SPC13.Cluster5690cbin.1	41.20	Lachnospiraceae	63368	2943330
Sample_SPC13.mb.29	60.10	Bacteroidetes	72774	2590733
Sample_SPC13.mb.52cbin.1	60.50	Clostridiales	70988	2456418
Sample_SPC13.mb.43	61.90	Actinobacteria	25017	2155433
Sample_SPC13.mb.55	61.90	Clostridiales	38444	2387729
Sample_SPC13.mb.49	57.50	Clostridiales	33801	2731672
Sample_SPC13.mb.88	52.60	Clostridia	6172	1673310
Sample_SPC13.Cluster8560cbin.1	31.30	Firmicutes	67427	2446744
Sample_SPC13.mb.59	48.40	Clostridiales	32635	2748853
Sample_SPC13.mb.72	54.60	Actinobacteria	20716	1553417
Sample_SPC13.mb.24cbin.1	59.30	Bacteroidetes	102977	2711283
Sample_SPC13.mb.20	41.90	Lachnospiraceae	84512	2380042
Sample_SPC13.mb.15	43.30	Bacteroidales	293525	3156320
Sample_SPC13.Cluster3275cbin.1	45.90	Bacteroidales	118413	3473811
Sample_SPC13.mb.57	60.10	Bacteroidetes	106789	2691318
Sample_SPC13.mb.9	41.30	Clostridiales	24853	3199709
Sample_SPC13.mb.53	43.30	Lachnospiraceae	135214	3034808
Sample_SPC13.Cluster5341cbin.1	45.40	Bacteroidales	79608	4153032
Sample_SPC13.mb.48cbin.1	57.00	Clostridiales	59774	2516593
Sample_SPC13.Cluster3765cbin.1	46.80	Bacteroidales	62534	4103325
Sample_SPC13.mb.79	54.70	Bacteroidetes	95639	2125479
Sample_SPC13.mb.66cbin.1	49.50	Proteobacteria	5259	1635346
Sample_SPC13.mb.83	36.40	Clostridiales	81039	2105673
Sample_SPC13.Cluster2319cbin.1	45.10	Bacteroidales	54953	4698528
Sample_SPC13.mb.76	36.20	Clostridiales	65777	2530808
Sample_SPC13.mb.8	50.40	Clostridiales	15166	2736440
Sample_SPC13.mb.56	40.50	Bacteroidales	53838	2938770
Sample_SPC13.mb.54	59.00	Bacteroidetes	218648	3194810
Sample_SPC13.mb.77	43.50	Clostridiales	26149	3196856
Sample_SPC13.mb.84	42.20	Bacteroidales	74392	3614305
Sample_SPC13.Cluster411	57.70	Enterobacteriaceae	259701	5089895
Sample_SPC13.mb.70cbin.1	42.00	Bacteroidales	62641	4735986
Sample_SPC15.mb.26cbin.1	45.40	Clostridia	39517	1793647
Sample_SPC15.Cluster10481	37.60	Clostridiales	166181	2100755
Sample_SPC15.Cluster5266	42.00	Clostridiales	15886	2507425
Sample_SPC15.Cluster5521cbin.1	44.20	Bacteroidales	141132	2247102
Sample_SPC15.mb.22	61.10	Clostridiales	53876	1936476
Sample_SPC15.Cluster4826cbin.1	47.20	Lachnospiraceae	21753	2636176
Sample_SPC15.Cluster2952	47.20	Selenomonadales	72871	2261220
Sample_SPC15.Cluster6cbin.1	63.00	Proteobacteria	7676	1743006
Sample_SPC15.Cluster5604cbin.1	44.50	Clostridiales	12214	2363309
Sample_SPC15.mb.51	44.50	Bacteroidales	29292	2539245
Sample_SPC15.Cluster1367	53.00	Lactobacillales	51592	1752594
Sample_SPC15.Cluster11881	32.70	Lactobacillales	136457	1862847

Sample_SPC15.mb.31	34.70	Lactobacillus	8969	1642523
Sample_SPC15.mb.30	43.60	Lachnospiraceae	41183	3315649
Sample_SPC15.mb.66	51.20	Bacteroidales	51968	2289686
Sample_SPC15.mb.17	38.50	Selenomonadales	29793	2043372
Sample_SPC15.mb.59	44.50	Clostridiales	83947	2429741
Sample_SPC15.mb.40	43.30	Lachnospiraceae	16100	2951889
Sample_SPC15.mb.72cbin.1	56.70	Clostridiales	163790	2162216
Sample_SPC15.mb.55	50.60	Bacteroidales	118724	2195559
Sample_SPC15.mb.80cbin.1	45.10	Bacteroidales	43793	2877920
Sample_SPC15.mb.48	58.60	Bifidobacteriaceae	24696	2354208
Sample_SPC15.mb.83	51.50	Clostridiales	56750	2051698
Sample_SPC15.mb.25cbin.1	42.50	Bacteroides	92287	5865089
Sample_SPC15.Cluster2024cbin.1	50.90	Enterobacteriaceae	172271	4657877
Sample_SPC16.mb.2	36.70	Clostridiales	6408	2143002
Sample_SPC16.Cluster4036cbin.1	48.20	Clostridiales	30276	2881591
Sample_SPC16.mb.29cbin.1	58.10	Clostridiales	7346	1579621
Sample_SPC16.Cluster8896	33.30	Bacteria	56535	2293736
Sample_SPC16.Cluster3365	43.50	Selenomonadales	113579	2419140
Sample_SPC16.Cluster799cbin.1	57.20	Proteobacteria	30649	2007307
Sample_SPC16.mb.25cbin.1	46.60	Lachnospiraceae	22542	2899300
Sample_SPC16.Cluster10140	32.50	Lactobacillales	109275	1879687
Sample_SPC16.mb.37	49.00	Clostridiales	30237	2744185
Sample_SPC16.Cluster7549cbin.1	37.40	Clostridiales	135247	2740939
Sample_SPC16.Cluster5477cbin.1	40.60	Bacteroidales	35691	2857943
Sample_SPC16.mb.27cbin.1	41.40	Lachnospiraceae	90085	2886606
Sample_SPC16.Cluster7014cbin.1	41.30	Lachnospiraceae	59834	3515254
Sample_SPC16.mb.71	26.70	Clostridiales	126575	2708957
Sample_SPC16.Cluster3708	45.50	Bacteroidales	140398	4091399
Sample_SPC16.mb.62	44.20	Bacteroidales	71014	3419287
Sample_SPC17.mb.37	48.00	Clostridiales	93867	1952141
Sample_SPC17.Cluster1353	46.80	Lactobacillales	44701	1774024
Sample_SPC17.Cluster1055	48.70	Clostridiales	124392	2611213
Sample_SPC17.Cluster306cbin.1	53.10	Clostridiales	26353	2685819
Sample_SPC17.mb.41	54.80	Bacteroidetes	68843	2109939
Sample_SPC17.Cluster1142cbin.1	43.60	Selenomonadales	41417	2314974
Sample_SPC17.Cluster2779cbin.1	37.00	Clostridiales	9551	2550291
Sample_SPC17.mb.60	37.30	Lactobacillales	31795	1643805
Sample_SPC17.mb.1cbin.1	41.30	Lachnospiraceae	161084	2653461
Sample_SPC17.Cluster1240	45.40	Lactobacillales	21900	2161345
Sample_SPC17.Cluster3452cbin.1	34.60	Lactobacillus	12690	1823169
Sample_SPC17.mb.28cbin.1	60.20	Bifidobacteriaceae	25131	2133393
Sample_SPC17.Cluster48cbin.1	58.90	Bifidobacteriaceae	33087	1999540
Sample_SPC17.mb.64cbin.1	60.30	Bifidobacteriaceae	33816	1819188
Sample_SPC17.Cluster66cbin.1	59.70	Clostridiales	39466	2155794
Sample_SPC17.mb.51cbin.1	36.00	Clostridiales	22936	2437875
Sample_SPC17.mb.43cbin.1	48.70	Proteobacteria	29171	2245817
Sample_SPC17.mb.74cbin.1	58.80	Clostridiales	64090	2462355
Sample_SPC17.Cluster2305	38.80	Lactobacillales	60957	4407976
Sample_SPC17.Cluster887cbin.1	48.90	Bacteroidales	16805	3277673
Sample_SPC17.Cluster2479cbin.1	42.80	Lachnospiraceae	101177	2868703
Sample_SPC17.Cluster2998cbin.1	40.60	Lachnospiraceae	56222	3143458
Sample_SPC17.mb.2cbin.1	46.60	Bacteroidales	120581	3864729
Sample_SPC17.mb.45cbin.1	44.90	Bacteroidales	11173	4135760
Sample_SPC17.Cluster1300cbin.1	45.30	Bacteroidales	131877	4382426
Sample_SPC17.Cluster1839cbin.1	42.00	Bacteroidales	94778	4318815
Sample_SPC17.mb.73cbin.1	50.80	Enterobacteriaceae	139577	4612572
Sample_SPC20.Cluster11157	29.50	Bacteria	5239	1233172
Sample_SPC20.Cluster6091cbin.1	46.10	Clostridia	43043	1744271

Sample_SPC20.mb.26cbin.1	25.90	Bacteria	37188	1292988
Sample_SPC20.mb.23	51.00	Clostridia	20933	1612344
Sample_SPC20.Cluster8391cbin.1	42.30	Clostridiales	24943	2804218
Sample_SPC20.mb.90	26.60	Bacteria	33859	2653523
Sample_SPC20.Cluster2624cbin.1	51.10	Clostridiales	107984	2647035
Sample_SPC20.mb.93	49.40	Clostridiales	96139	1950849
Sample_SPC20.Cluster6543	44.70	Clostridiales	139110	2244985
Sample_SPC20.mb.82	48.50	Clostridiales	19839	2710311
Sample_SPC20.mb.24cbin.1	51.10	Clostridiales	487108	2059487
Sample_SPC20.Cluster8953cbin.1	40.80	Clostridiales	99094	2757667
Sample_SPC20.mb.41	47.10	Lachnospiraceae	38482	3023604
Sample_SPC20.mb.60cbin.1	54.60	Bacteroidetes	21281	2432974
Sample_SPC20.Cluster7734	43.30	Lachnospiraceae	210099	2821818
Sample_SPC20.Cluster7449cbin.1	41.10	Lachnospiraceae	76709	2909142
Sample_SPC20.Cluster4172cbin.1	48.70	Proteobacteria	28597	2396775
Sample_SPC20.mb.94	49.40	Clostridiales	25861	2665803
Sample_SPC20.mb.3	58.80	Bacteroidetes	50313	2862448
Sample_SPC20.mb.20	59.40	Bacteroidetes	88152	2886779
Sample_SPC20.mb.66	37.60	Clostridiales	67791	2459053
Sample_SPC20.mb.59	36.80	Clostridiales	84947	2867527
Sample_SPC20.mb.69	28.90	Clostridiales	42662	2316615
Sample_SPC20.mb.18	43.80	Bacteroidales	186505	2963172
Sample_SPC20.mb.30	46.50	Bacteroidales	56910	3066119
Sample_SPC20.mb.61	40.60	Lachnospiraceae	65643	3135693
Sample_SPC20.Cluster9809cbin.1	44.90	Bacteroidales	9061	4118522
Sample_SPC20.mb.65	41.90	Bacteroidales	51662	4769728
Sample_SPC21.mb.16cbin.1	46.80	Bacteria	94409	3737780
Sample_SPC21.Cluster3681	50.10	Bacteria	77162	2081787
Sample_SPC21.Cluster5073	49.50	Clostridiales	99611	1917625
Sample_SPC21.mb.30	32.40	Clostridiales	34250	2467950
Sample_SPC21.mb.29	44.70	Clostridiales	33776	2618748
Sample_SPC21.mb.23cbin.1	61.80	Clostridiales	29443	1752587
Sample_SPC21.mb.39	43.20	Clostridiales	53862	3026175
Sample_SPC21.mb.10	41.40	Lachnospiraceae	82545	3160969
Sample_SPC21.mb.35	54.40	Clostridiales	20692	2711458
Sample_SPC21.mb.33cbin.1	48.40	Clostridiales	27716	2808478
Sample_SPC21.mb.73cbin.1	35.20	Bacteria	186701	2281032
Sample_SPC21.mb.24cbin.1	53.20	Clostridiales	101089	2979775
Sample_SPC21.mb.84cbin.1	36.40	Clostridiales	39041	1814155
Sample_SPC21.mb.6	46.00	Clostridiales	17891	2043488
Sample_SPC21.mb.88	58.30	Clostridiales	60563	2861469
Sample_SPC21.mb.77	61.60	Clostridiales	78974	1947810
Sample_SPC21.Cluster9797cbin.1	40.20	Clostridiales	129656	2311772
Sample_SPC21.Cluster11874cbin.1	37.30	Clostridiales	81973	2599088
Sample_SPC21.mb.18	44.30	Clostridiales	183843	2662366
Sample_SPC21.mb.78cbin.1	45.10	Clostridiales	35430	2524495
Sample_SPC21.mb.62	56.60	Clostridiales	14410	2354055
Sample_SPC21.mb.70cbin.1	54.90	Bacteroidetes	149034	2003354
Sample_SPC21.mb.45cbin.1	42.90	Lachnospiraceae	36729	2364055
Sample_SPC21.mb.8cbin.1	57.40	Clostridiales	23969	2445612
Sample_SPC21.Cluster7727	43.50	Bacteroidales	133776	2633656
Sample_SPC21.mb.72	41.30	Clostridiales	145074	2508334
Sample_SPC21.Cluster4981cbin.1	44.70	Bacteroidales	65789	3128573
Sample_SPC21.mb.91	59.30	Bacteroidetes	185297	2884038
Sample_SPC21.Cluster6746	43.30	Bacteria	54857	3985791
Sample_SPC21.mb.87cbin.1	59.00	Bacteroidetes	98234	3038355
Sample_SPC21.Cluster7572cbin.1	48.00	Bacteroidales	107957	3923810
Sample_SPC21.mb.60	40.30	Bacteroidales	38706	3556489

Sample_SPC21.mb.93	40.70	Clostridiales	9227	2836600
Sample_SPC21.Cluster6987cbin.1	45.00	Bacteroidales	23847	4422272
Sample_SPC21.Cluster6056	45.10	Bacteroidales	97630	4468754
Sample_SPC21.mb.98	26.80	Clostridiales	41007	2835478
Sample_SPC21.mb.1	41.80	Bacteroidales	200826	4932183
Sample_SPC22.Cluster17904	26.40	Bacteria	126423	1157326
Sample_SPC22.Cluster7620cbin.1	51.20	Bacteria	115060	2215806
Sample_SPC22.Cluster16607	36.80	Clostridiales	135840	1313344
Sample_SPC22.Cluster7396	50.90	Bacteria	103486	1907425
Sample_SPC22.mb.117	27.40	Bacteria	116012	986406
Sample_SPC22.Cluster481	60.60	Actinobacteria	73239	1971926
Sample_SPC22.Cluster8892cbin.1	49.40	Clostridiales	108103	2003523
Sample_SPC22.Cluster110cbin.1	60.40	Clostridiales	22506	1785213
Sample_SPC22.Cluster184	58.50	Clostridiales	76000	2383507
Sample_SPC22.mb.107	54.10	Actinobacteria	143894	1653649
Sample_SPC22.mb.21	46.20	Bacteria	128481	3905754
Sample_SPC22.Cluster9571cbin.1	48.00	Clostridiales	102511	2722517
Sample_SPC22.mb.41	26.70	Bacteria	95679	1257497
Sample_SPC22.Cluster15262	37.60	Clostridiales	7860	2113768
Sample_SPC22.mb.12	55.50	Clostridiales	80712	2860698
Sample_SPC22.Cluster17294	32.50	Lactobacillales	109681	1834700
Sample_SPC22.Cluster17647cbin.1	26.90	Clostridiales	20964	2819151
Sample_SPC22.Cluster1719cbin.1	57.80	Bacteria	161495	3055854
Sample_SPC22.mb.140cbin.1	57.20	Clostridiales	76264	2697554
Sample_SPC22.mb.130cbin.1	52.40	Clostridiales	13285	2269993
Sample_SPC22.mb.33	58.10	Clostridiales	55498	3016645
Sample_SPC22.mb.103	53.60	Clostridiales	22323	2215333
Sample_SPC22.mb.31	43.90	Clostridiales	33515	2454535
Sample_SPC22.mb.113	60.20	Bifidobacteriaceae	76017	2174588
Sample_SPC22.Cluster1939cbin.1	58.60	Bifidobacteriaceae	137006	2555582
Sample_SPC22.Cluster14493cbin.1	38.20	Lactobacillales	12870	2545764
Sample_SPC22.mb.46	62.20	Clostridiales	9124	1486290
Sample_SPC22.mb.78	52.90	Clostridia	49630	2090446
Sample_SPC22.mb.143cbin.1	40.50	Clostridiales	112278	2172856
Sample_SPC22.mb.16	41.30	Clostridiales	199344	2611125
Sample_SPC22.mb.62	58.40	Clostridiales	8248	1903050
Sample_SPC22.mb.38	60.30	Bacteroidetes	69830	2497977
Sample_SPC22.mb.60cbin.1	55.70	Clostridiales	8565	2384860
Sample_SPC22.mb.65cbin.1	36.50	Clostridiales	44610	3634967
Sample_SPC22.Cluster7098cbin.1	49.10	Clostridiales	58173	6642387
Sample_SPC22.mb.126	43.40	Bacteroidales	124265	3218152
Sample_SPC22.mb.54	54.80	Bacteroidetes	104284	2359173
Sample_SPC22.mb.39cbin.1	60.70	Deltaproteobacteria	7921	3357113
Sample_SPC22.mb.30	58.60	Bacteroidetes	162049	2658271
Sample_SPC22.mb.98	61.80	Clostridiales	48382	1935539
Sample_SPC22.mb.43	46.90	Lachnospiraceae	39413	7068934
Sample_SPC22.mb.66	45.30	Clostridiales	18476	2560268
Sample_SPC22.mb.127	41.80	Bacteroides	86699	4747709
Sample_SPC22.mb.125cbin.1	50.50	Enterobacteriaceae	44065	4591802
Sample_SPC22.mb.45cbin.1	45.40	Bacteroidales	15793	3949308
Sample_SPC22.mb.86cbin.1	37.60	Lactobacillales	155045	2662997
Sample_SPC22.mb.68cbin.1	46.30	Bacteroidales	37785	3354208
Sample_SPC22.mb.81	41.80	Bacteroidales	60079	4278715
Sample_SPC22.mb.87cbin.1	57.30	Enterobacteriaceae	121810	5423636
Sample_SPC25.Cluster24cbin.1	61.60	Clostridiales	19931	2101099
Sample_SPC25.Cluster19206cbin.1	33.50	Bacteria	25892	2289360
Sample_SPC25.mb.5	60.60	Bacteria	32888	2549599
Sample_SPC25.Cluster5384cbin.1	51.90	Clostridiales	95504	2112900

Sample_SPC25.Cluster3917	51.30	Bacteroidales	44364	2353996
Sample_SPC25.mb.10	62.50	Clostridiales	81009	1583900
Sample_SPC25.Cluster14599cbin.1	38.60	Clostridiales	75497	2085782
Sample_SPC25.Cluster2875	54.40	Prevotella	57418	2421909
Sample_SPC25.Cluster13107cbin.1	41.90	Clostridiales	175692	2440210
Sample_SPC25.Cluster6801	47.20	Selenomonadales	60517	2183845
Sample_SPC25.Cluster11604cbin.1	44.30	Clostridiales	182633	2816857
Sample_SPC25.mb.27	56.30	Clostridiales	15675	2189426
Sample_SPC25.mb.67	26.00	Bacteria	130107	1250005
Sample_SPC25.Cluster33	63.60	Deltaproteobacteria	59876	2639707
Sample_SPC25.Cluster4444cbin.1	50.40	Bacteroidales	97801	2285152
Sample_SPC25.mb.107cbin.1	53.90	Clostridiales	7573	1837890
Sample_SPC25.mb.23cbin.1	44.50	Bacteroidales	26701	2583734
Sample_SPC25.Cluster17392cbin.1	37.40	Clostridiales	39972	2689681
Sample_SPC25.mb.118	56.70	Clostridiales	6874	2219716
Sample_SPC25.Cluster13379cbin.1	41.00	Lachnospiraceae	67670	3096755
Sample_SPC25.mb.117	57.10	Proteobacteria	36776	1906956
Sample_SPC25.mb.110cbin.1	51.30	Clostridiales	115553	2244530
Sample_SPC25.mb.102cbin.1	43.60	Clostridiales	140162	2109773
Sample_SPC25.mb.56	41.10	Clostridiales	33637	3192492
Sample_SPC25.mb.103cbin.1	44.40	Clostridiales	92382	2390445
Sample_SPC25.mb.66	60.10	Clostridiales	16371	2092976
Sample_SPC25.Cluster12091cbin.1	42.20	Bacteria	13471	3454118
Sample_SPC25.mb.47	46.10	Bacteroidetes	176765	2484168
Sample_SPC25.mb.59	58.10	Bacteroidetes	160485	2327723
Sample_SPC25.mb.87	61.70	Clostridiales	28680	1853296
Sample_SPC25.mb.82cbin.1	51.00	Clostridiales	25944	2355191
Sample_SPC25.mb.98	49.10	Lachnospiraceae	9927	2488571
Sample_SPC25.mb.85	46.90	Lachnospiraceae	45848	2634459
Sample_SPC25.mb.92cbin.1	33.20	Bacteria	10079	2562112
Sample_SPC25.mb.14cbin.1	45.20	Bacteroidales	10633	3979116
Sample_SPC25.mb.4cbin.1	57.50	Enterobacteriaceae	218800	5206090
Sample_SPC26.Cluster285cbin.1	61.50	Clostridiales	50150	1800910
Sample_SPC26.mb.126	52.70	Clostridiales	9749	1817738
Sample_SPC26.mb.12	32.90	Bacteria	73704	1840356
Sample_SPC26.Cluster664cbin.1	56.80	Clostridiales	90764	1889319
Sample_SPC26.Cluster5521	49.80	Clostridiales	116697	2076227
Sample_SPC26.mb.114	59.00	Clostridiales	57843	2007486
Sample_SPC26.Cluster4938cbin.1	48.70	Firmicutes	28464	1653031
Sample_SPC26.mb.21	58.40	Clostridiales	40122	2878521
Sample_SPC26.mb.105	50.00	Clostridiales	9792	1528545
Sample_SPC26.Cluster4153cbin.1	53.70	Clostridiales	149267	1880578
Sample_SPC26.Cluster446cbin.1	59.60	Bacteroidetes	119135	2209870
Sample_SPC26.Cluster8877	44.20	Clostridiales	185555	1922683
Sample_SPC26.Cluster522cbin.1	60.50	Bifidobacteriaceae	83839	1756699
Sample_SPC26.Cluster3107	54.30	Selenomonadales	84740	2219270
Sample_SPC26.Cluster20cbin.1	62.80	Proteobacteria	29344	2307573
Sample_SPC26.mb.41	47.10	Clostridiales	48933	1999198
Sample_SPC26.mb.118cbin.1	38.80	Clostridiales	72508	2024988
Sample_SPC26.mb.38	58.70	Clostridiales	6839	1705174
Sample_SPC26.mb.128	55.00	Bacteroidetes	121540	2694129
Sample_SPC26.mb.24cbin.1	49.40	Bacteroidales	81464	2660185
Sample_SPC26.Cluster8628	41.80	Lachnospiraceae	61785	2368516
Sample_SPC26.mb.29	58.20	Bacteroidetes	241320	2597586
Sample_SPC26.mb.20cbin.1	44.20	Clostridiales	150700	2726340
Sample_SPC26.mb.48	56.40	Clostridiales	62498	2203828
Sample_SPC26.mb.28cbin.1	51.50	Clostridiales	173750	2229543
Sample_SPC26.Cluster2762cbin.1	48.90	Bacteroidales	11604	3297824

Sample_SPC26.mb.109cbin.1	43.10	Bacteria	10860	3885597
Sample_SPC26.mb.15	43.30	Bacteroidales	175906	3021238
Sample_SPC26.Cluster7097	45.30	Bacteroidales	29641	4036502
Sample_SPC26.mb.71cbin.1	61.40	Clostridiales	41472	2606467
Sample_SPC26.mb.35	58.70	Bacteroidetes	136598	2763276
Sample_SPC26.mb.51	43.40	Clostridiales	11784	2130027
Sample_SPC26.mb.16cbin.1	46.00	Bacteroidales	18220	3701510
Sample_SPC26.mb.62cbin.1	46.10	Prevotella	48674	3247279
Sample_SPC26.mb.82	46.30	Prevotella	41611	2524400
Sample_SPC26.mb.53cbin.1	59.70	Bacteroidetes	10639	2816976
Sample_SPC26.mb.6cbin.1	40.80	Lachnospiraceae	96663	3336585
Sample_SPC26.mb.34	45.10	Bacteroidales	69684	4451219
Sample_SPC26.mb.50	43.50	Bacteroidales	129444	4481147
Sample_SPC27.Cluster3329cbin.1	49.50	Clostridiales	102160	2062437
Sample_SPC27.Cluster9815	28.50	Bacteria	63515	1535571
Sample_SPC27.Cluster546	58.30	Clostridiales	65797	2803432
Sample_SPC27.Cluster858cbin.1	55.10	Clostridia	32620	2503385
Sample_SPC27.Cluster6911	41.70	Clostridiales	132554	2793540
Sample_SPC27.Cluster228cbin.1	59.80	Clostridiales	82682	2065906
Sample_SPC27.Cluster4231	46.10	Clostridiales	116390	2084465
Sample_SPC27.Cluster103cbin.1	61.80	Clostridiales	44637	2462544
Sample_SPC27.mb.52	60.10	Bacteria	98440	2296104
Sample_SPC27.Cluster120cbin.1	60.10	Bacteria	35543	2379255
Sample_SPC27.Cluster5420cbin.1	43.60	Clostridiales	117224	1900378
Sample_SPC27.mb.107cbin.1	46.80	Lactobacillales	60969	2013544
Sample_SPC27.Cluster3062cbin.1	50.80	Clostridiales	315336	2359110
Sample_SPC27.mb.117	41.00	Clostridiales	40881	3409231
Sample_SPC27.mb.109cbin.1	57.40	Clostridiales	59138	2873643
Sample_SPC27.Cluster2068cbin.1	52.80	Clostridiales	152553	2323111
Sample_SPC27.Cluster7512	38.40	Clostridiales	92717	2061388
Sample_SPC27.Cluster5238cbin.1	44.10	Clostridiales	34862	3191310
Sample_SPC27.Cluster323cbin.1	60.40	Bifidobacteriaceae	111109	1928460
Sample_SPC27.mb.100cbin.1	60.50	Clostridiales	52686	2225947
Sample_SPC27.mb.20cbin.1	36.70	Clostridiales	11452	3284711
Sample_SPC27.Cluster66cbin.1	60.30	Bifidobacteriaceae	131495	2381615
Sample_SPC27.mb.121cbin.1	48.60	Firmicutes	33807	1849267
Sample_SPC27.mb.37	44.80	Clostridiales	59052	2437689
Sample_SPC27.Cluster1521cbin.1	58.60	Bacteroidetes	177411	3010921
Sample_SPC27.Cluster9403cbin.1	36.80	Clostridiales	13272	2980284
Sample_SPC27.mb.41	41.00	Lachnospiraceae	110628	2466216
Sample_SPC27.mb.120	43.50	Clostridiales	8358	2959404
Sample_SPC27.mb.54cbin.1	52.30	Clostridiales	48400	2728209
Sample_SPC27.mb.79cbin.1	48.00	Clostridiales	58615	2975785
Sample_SPC27.mb.28	43.60	Lachnospiraceae	104659	2862911
Sample_SPC27.mb.71	54.80	Bacteroidetes	180843	1896845
Sample_SPC27.mb.1cbin.1	49.20	Proteobacteria	48021	2065330
Sample_SPC27.Cluster3474	44.60	Bacteroidales	84356	3609652
Sample_SPC27.mb.30	58.90	Bacteroidetes	124707	2755075
Sample_SPC27.Cluster3889cbin.1	45.10	Bacteroidales	82714	4471266
Sample_SPC27.mb.93	49.00	Clostridiales	67428	2674281
Sample_SPC27.mb.81	61.90	Clostridiales	61111	2842981
Sample_SPC27.mb.85	40.00	Streptococcus	40652	1978587
Sample_SPC27.mb.123	46.70	Bacteroidales	172786	3969826
Sample_SPC27.mb.90	35.60	Clostridiales	84198	2925512
Sample_SPC27.mb.69cbin.1	42.00	Bacteroidales	87552	5011204
Sample_SPC28.Cluster11270	27.10	Bacteria	118041	1411319
Sample_SPC28.mb.2	28.10	Bacteria	27371	976352
Sample_SPC28.Cluster11306	27.10	Clostridiales	120950	1759372

Sample_SPC28.Cluster483cbin.1	58.00	Clostridiales	57123	3050072
Sample_SPC28.mb.20	34.30	Clostridiales	79030	2227408
Sample_SPC28.Cluster10398	33.50	Bacteria	46515	2229282
Sample_SPC28.mb.22	57.30	Clostridiales	13939	2238011
Sample_SPC28.Cluster3456cbin.1	48.40	Firmicutes	32264	1876376
Sample_SPC28.Cluster3153cbin.1	49.40	Bacteroidales	142363	2780746
Sample_SPC28.Cluster8cbin.1	60.40	Bacteroidetes	6302	2239627
Sample_SPC28.Cluster753cbin.1	54.30	Clostridiales	19624	1702707
Sample_SPC28.Cluster5150	44.20	Clostridiales	238218	1885466
Sample_SPC28.Cluster9cbin.1	60.30	Bifidobacteriaceae	7850	1825217
Sample_SPC28.Cluster5697cbin.1	44.30	Clostridiales	159113	2552759
Sample_SPC28.Cluster6428	44.10	Clostridiales	109858	2737212
Sample_SPC28.mb.24	41.20	Clostridiales	114167	2604788
Sample_SPC28.Cluster4642cbin.1	43.60	Bacteroidales	6264	2312258
Sample_SPC28.Cluster965	56.20	Bifidobacteriaceae	134229	2133424
Sample_SPC28.Cluster8548cbin.1	37.40	Clostridiales	20267	2601809
Sample_SPC28.mb.72cbin.1	46.70	Clostridiales	102272	2688118
Sample_SPC28.mb.92	44.30	Clostridiales	137179	3302646
Sample_SPC28.mb.67	60.50	Bacteroidetes	27576	2377599
Sample_SPC28.mb.33	26.70	Clostridiales	41885	2568532
Sample_SPC28.mb.40	50.30	Lachnospiraceae	17253	2549281
Sample_SPC28.mb.9cbin.1	48.80	Clostridiales	12278	2530372
Sample_SPC28.mb.13	48.40	Proteobacteria	57383	2573341
Sample_SPC28.mb.62cbin.1	35.90	Clostridiales	231014	2658124
Sample_SPC28.Cluster5040cbin.1	43.20	Bacteria	11808	3744151
Sample_SPC28.mb.77	41.50	Lachnospiraceae	68054	2853747
Sample_SPC28.mb.7cbin.1	43.40	Lachnospiraceae	79423	3032423
Sample_SPC28.mb.89cbin.1	40.50	Clostridiales	53167	2929585
Sample_SPC28.mb.43cbin.1	47.00	Bacteroidales	27274	3509814
Sample_SPC30.Cluster7389cbin.1	37.40	Clostridiales	6018	1970352
Sample_SPC30.Cluster1051	56.40	Clostridiales	111782	2218386
Sample_SPC30.Cluster3246	47.60	Clostridiales	135415	3060166
Sample_SPC30.mb.31	62.80	Clostridiales	8300	1712425
Sample_SPC30.Cluster236cbin.1	59.80	Actinobacteria	94250	2245600
Sample_SPC30.mb.108cbin.1	34.20	Bacilli	23555	2787307
Sample_SPC30.mb.100cbin.1	50.40	Clostridiales	25786	2546667
Sample_SPC30.mb.35	56.90	Clostridiales	36817	2429148
Sample_SPC30.Cluster4993cbin.1	44.20	Clostridiales	14324	2995269
Sample_SPC30.mb.24cbin.1	58.70	Clostridiales	21947	2307308
Sample_SPC30.Cluster7999cbin.1	37.90	Clostridiales	133865	2078088
Sample_SPC30.Cluster5293cbin.1	42.60	Clostridiales	74600	2698675
Sample_SPC30.mb.1	34.60	Lactobacillus	118341	1877988
Sample_SPC30.mb.43cbin.1	60.50	Clostridiales	44227	2383740
Sample_SPC30.Cluster7400cbin.1	37.60	Clostridiales	8841	2358307
Sample_SPC30.Cluster5295cbin.1	45.20	Prevotella	57580	3242125
Sample_SPC30.mb.48	39.90	Clostridiales	31294	2279916
Sample_SPC30.Cluster9176cbin.1	32.70	Lactobacillales	128322	2022573
Sample_SPC30.Cluster5217cbin.1	42.90	Lachnospiraceae	124235	3270227
Sample_SPC30.mb.87	42.00	Bacteria	67402	4461309
Sample_SPC30.mb.13	37.40	Lactobacillales	101377	1706604
Sample_SPC30.mb.73	58.40	Clostridiales	62576	2884028
Sample_SPC30.Cluster8831cbin.1	40.90	Lachnospiraceae	59230	3003543
Sample_SPC30.mb.11cbin.1	36.00	Clostridiales	179187	2585864
Sample_SPC30.mb.41	56.40	Clostridiales	14362	2446016
Sample_SPC30.Cluster3797cbin.1	42.90	Lachnospiraceae	8427	3212786
Sample_SPC30.mb.78	41.30	Clostridiales	26035	2872544
Sample_SPC30.mb.62	51.00	Lactobacillales	8298	1552879
Sample_SPC30.mb.34	58.60	Bifidobacteriaceae	191128	2499626

Sample_SPC30.mb.99	47.30	Lactobacillales	11556	1680569
Sample_SPC30.Cluster6113cbin.1	47.60	Clostridiales	129291	6963900
Sample_SPC30.mb.85	54.90	Bacteroidetes	141023	1939375
Sample_SPC30.Cluster3074	45.30	Bacteroidales	56811	4023637
Sample_SPC30.mb.71cbin.1	51.40	Clostridiales	26466	2099018
Sample_SPC30.mb.56cbin.1	44.90	Clostridiales	20821	2850275
Sample_SPC30.mb.82	39.00	Clostridiales	88333	2138750
Sample_SPC30.mb.75	44.00	Clostridiales	222689	2760629
Sample_SPC30.mb.52	44.20	Bacteria	7781	3476682
Sample_SPC30.mb.68cbin.1	31.20	Firmicutes	48279	2241399
Sample_SPC30.mb.80cbin.1	40.50	Bacteroidales	57029	3226343
Sample_SPC30.mb.74cbin.1	64.20	Actinobacteria	21068	3274243
Sample_SPC31.Cluster13517	24.10	Bacteria	37347	941253
Sample_SPC31.mb.19	26.60	Bacteria	70965	1353499
Sample_SPC31.Cluster5528cbin.1	49.40	Clostridiales	86265	1957922
Sample_SPC31.Cluster4761cbin.1	49.70	Clostridiales	81034	2015830
Sample_SPC31.Cluster7149	45.20	Clostridiales	87705	1706105
Sample_SPC31.mb.49	25.90	Bacteria	36330	1193699
Sample_SPC31.Cluster557cbin.1	57.80	Clostridiales	19010	1690924
Sample_SPC31.Cluster7073cbin.1	45.30	Clostridiales	6901	2066378
Sample_SPC31.Cluster23cbin.1	61.50	Bacteria	7829	4099330
Sample_SPC31.mb.36	61.90	Clostridiales	13459	1592080
Sample_SPC31.mb.64cbin.1	53.40	Clostridia	55760	1875273
Sample_SPC31.mb.43cbin.1	56.80	Clostridiales	27323	2465976
Sample_SPC31.Cluster1	59.60	Actinobacteria	129532	2220875
Sample_SPC31.mb.80cbin.1	43.00	Bacteria	14515	2481169
Sample_SPC31.mb.33	62.00	Clostridiales	23763	2267452
Sample_SPC31.Cluster851cbin.1	57.60	Clostridiales	41145	3044665
Sample_SPC31.mb.5	62.20	Clostridiales	10841	1640485
Sample_SPC31.mb.76	27.70	Clostridiales	80207	1480211
Sample_SPC31.Cluster9043cbin.1	43.60	Clostridiales	148048	2033068
Sample_SPC31.Cluster3927	53.00	Clostridiales	170394	2219996
Sample_SPC31.Cluster456	60.40	Bifidobacteriaceae	62302	1903772
Sample_SPC31.mb.100cbin.1	51.00	Clostridiales	175567	2233156
Sample_SPC31.mb.35cbin.1	45.10	Clostridiales	7269	2113737
Sample_SPC31.mb.60cbin.1	60.40	Clostridiales	28748	1847013
Sample_SPC31.mb.18cbin.1	44.40	Clostridiales	141744	2397269
Sample_SPC31.mb.65	58.00	Clostridiales	56930	2328943
Sample_SPC31.mb.42	40.90	Clostridiales	31489	2587013
Sample_SPC31.mb.58	55.20	Bacteroidetes	21486	1846453
Sample_SPC31.mb.83	59.70	Clostridiales	122937	2273257
Sample_SPC31.mb.39	41.30	Lachnospiraceae	90284	2783813
Sample_SPC31.mb.9	61.60	Clostridiales	52753	1868684
Sample_SPC31.mb.103	43.50	Bacteroidales	288270	3118869
Sample_SPC31.Cluster9271cbin.1	41.80	Clostridiales	6843	3162510
Sample_SPC31.mb.90	59.80	Bacteroidetes	63233	2508071
Sample_SPC31.mb.104	46.70	Bacteroidales	117888	4505166
Sample_SPC31.mb.12cbin.1	44.50	Bacteroidales	44936	4928245
Sample_SPC31.Cluster6989cbin.1	43.80	Bacteroidales	27724	5553525
Sample_SPC31.mb.68cbin.1	28.00	Clostridiales	142601	3480005
Sample_SPC31.Cluster446	58.00	Enterobacteriaceae	77550	4841033
Sample_SPC32.mb.18cbin.1	47.00	Bacteria	62037	1811368
Sample_SPC32.Cluster5908	33.90	Clostridiales	9489	1721186
Sample_SPC32.mb.23	38.30	Clostridiales	104957	2079869
Sample_SPC32.mb.51	38.20	Clostridiales	105284	2057190
Sample_SPC32.Cluster3302cbin.1	48.70	Clostridiales	21418	2304439
Sample_SPC32.mb.48cbin.1	42.20	Clostridiales	79160	2511651
Sample_SPC32.Cluster784	52.20	Clostridiales	103824	2255060

Sample_SPC32.Cluster14	61.30	Clostridiales	69766	2608665
Sample_SPC32.mb.3cbin.1	42.80	Clostridiales	79983	3356855
Sample_SPC32.Cluster4295	41.00	Lachnospiraceae	139934	2582132
Sample_SPC32.mb.33cbin.1	47.70	Clostridiales	108725	3073540
Sample_SPC32.mb.68cbin.1	37.20	Clostridiales	12460	2509748
Sample_SPC32.mb.9cbin.1	58.40	Clostridiales	12540	2983215
Sample_SPC32.Cluster38	60.70	Bifidobacteriaceae	11051	1718494
Sample_SPC32.Cluster5660cbin.1	40.40	Clostridiales	162791	2181677
Sample_SPC32.mb.34cbin.1	54.20	Bacteroidetes	105785	2253201
Sample_SPC32.Cluster329	57.30	Proteobacteria	109781	2347806
Sample_SPC32.Cluster2799cbin.1	43.10	Lachnospiraceae	25944	2193279
Sample_SPC32.Cluster6603cbin.1	27.10	Clostridiales	24033	2966667
Sample_SPC32.Cluster5317cbin.1	37.10	Clostridiales	134346	3017214
Sample_SPC32.mb.72cbin.1	60.00	Bacteroidetes	181781	2428865
Sample_SPC32.mb.75	44.20	Clostridiales	245598	2710934
Sample_SPC32.mb.35cbin.1	42.70	Lachnospiraceae	96430	3513492
Sample_SPC32.Cluster1330cbin.1	45.30	Bacteroidales	30881	4077875
Sample_SPC32.Cluster2341	45.00	Bacteroidales	262926	4513953
Sample_SPC32.mb.43cbin.1	46.50	Bacteroidales	163380	4155000
Sample_SPC32.mb.16cbin.1	42.90	Bacteroides	88615	5301577
Sample_SPC32.mb.55cbin.1	41.40	Bacteroidales	20143	5042501
Sample_SPC34.Cluster218cbin.1	54.00	Clostridia	15752	1752650
Sample_SPC34.Cluster6412cbin.1	49.50	Clostridiales	109590	1977458
Sample_SPC34.Cluster13592	32.70	Bacteria	152040	1907826
Sample_SPC34.mb.16	28.30	Bacteria	118489	3036596
Sample_SPC34.Cluster180	62.20	Clostridiales	33736	1433334
Sample_SPC34.Cluster784cbin.1	56.80	Clostridiales	102807	2363632
Sample_SPC34.mb.43	26.00	Bacteria	88724	1240704
Sample_SPC34.mb.24cbin.1	58.10	Clostridiales	40481	3062761
Sample_SPC34.Cluster6988cbin.1	45.90	Clostridiales	13516	1889741
Sample_SPC34.mb.39	38.00	Clostridiales	299762	1709573
Sample_SPC34.Cluster3041	54.90	Bacteroidetes	146976	1968187
Sample_SPC34.mb.32	53.70	Clostridiales	17951	1811652
Sample_SPC34.Cluster11066cbin.1	40.80	Clostridiales	130155	2086852
Sample_SPC34.mb.102	60.10	Actinobacteria	67982	2074196
Sample_SPC34.Cluster2616	55.50	Bacteria	120135	2768717
Sample_SPC34.Cluster400	60.40	Bifidobacteriaceae	60357	1899635
Sample_SPC34.mb.84cbin.1	53.10	Clostridia	66695	2037349
Sample_SPC34.mb.28	58.50	Bacteroidetes	19438	2514318
Sample_SPC34.Cluster10388cbin.1	42.50	Clostridiales	115798	2982058
Sample_SPC34.mb.30	52.40	Firmicutes	28010	2118561
Sample_SPC34.mb.36	54.10	Clostridiales	46644	1706377
Sample_SPC34.Cluster8564cbin.1	43.70	Lachnospiraceae	66133	2749211
Sample_SPC34.mb.21cbin.1	59.70	Clostridiales	37298	1726279
Sample_SPC34.mb.26cbin.1	45.20	Clostridiales	29124	2676798
Sample_SPC34.mb.61cbin.1	58.70	Clostridiales	45926	2504228
Sample_SPC34.mb.68	55.90	Clostridiales	28346	3092589
Sample_SPC34.mb.65	52.90	Clostridiales	24971	2697634
Sample_SPC34.mb.78cbin.1	63.20	Actinobacteria	184147	2616697
Sample_SPC34.mb.76	59.20	Bacteroidetes	60053	2803686
Sample_SPC34.mb.87	55.00	Bacteroidetes	172834	2704810
Sample_SPC34.mb.74cbin.1	41.20	Lachnospiraceae	93965	3004850
Sample_SPC34.mb.29	46.10	Bacteroidales	127865	3417263
Sample_SPC34.mb.75	43.40	Bacteroidales	6352	2622203
Sample_SPC34.mb.95cbin.1	58.80	Bacteroidetes	30168	3135363
Sample_SPC34.Cluster6455cbin.1	57.90	Enterobacteriaceae	8677	4370492
Sample_SPC34.mb.57	41.70	Bacteroides	69328	5038548
Sample_SPC35.Cluster4392cbin.1	50.50	Bacteria	82685	1960156

Sample_SPC35.Cluster13613	27.70	Clostridiales	104905	1420925
Sample_SPC35.Cluster4020cbin.1	52.80	Clostridia	74520	2163851
Sample_SPC35.Cluster6132	49.40	Clostridiales	102517	1910393
Sample_SPC35.mb.109cbin.1	28.40	Bacteria	115425	1295659
Sample_SPC35.Cluster12386cbin.1	37.50	Clostridiales	161824	2133789
Sample_SPC35.Cluster8829	37.00	Clostridiales	117233	2581965
Sample_SPC35.mb.113cbin.1	50.80	Clostridiales	74919	1692104
Sample_SPC35.Cluster548cbin.1	60.60	Clostridiales	27121	1815312
Sample_SPC35.Cluster9504	41.80	Clostridiales	68258	2240052
Sample_SPC35.Cluster12742cbin.1	33.40	Bacteria	13076	2165388
Sample_SPC35.Cluster8080cbin.1	44.90	Clostridiales	88449	2616195
Sample_SPC35.Cluster6587	46.20	Lachnospiraceae	37731	2556718
Sample_SPC35.mb.29	60.10	Clostridiales	16097	2197508
Sample_SPC35.mb.31	27.50	Euryarchaeota	83759	1717914
Sample_SPC35.Cluster6966cbin.1	44.50	Clostridiales	6293	1914666
Sample_SPC35.mb.77cbin.1	53.90	Clostridia	14461	1663157
Sample_SPC35.mb.10	60.00	Actinobacteria	31619	2049513
Sample_SPC35.mb.64	59.30	Clostridiales	14202	2170238
Sample_SPC35.Cluster4cbin.1	64.00	Deltaproteobacteria	11600	2379110
Sample_SPC35.mb.80	28.10	Clostridiales	95650	1351070
Sample_SPC35.mb.92	25.20	Bacteria	69716	1214906
Sample_SPC35.mb.74	27.20	Clostridiales	200023	1866688
Sample_SPC35.mb.41cbin.1	56.90	Clostridiales	105509	1992371
Sample_SPC35.mb.48cbin.1	48.30	Clostridiales	22335	2897615
Sample_SPC35.mb.63	59.20	Clostridiales	31033	1826691
Sample_SPC35.mb.13cbin.1	58.10	Proteobacteria	14962	2026548
Sample_SPC35.mb.73cbin.1	43.50	Clostridiales	22415	2919187
Sample_SPC35.Cluster1269	58.50	Bacteroidetes	163087	3056577
Sample_SPC35.mb.112cbin.1	60.40	Bifidobacteriaceae	82128	1808795
Sample_SPC35.mb.4cbin.1	45.70	Lachnospiraceae	193910	2668061
Sample_SPC35.mb.101cbin.1	35.90	Clostridiales	188392	2561049
Sample_SPC35.mb.71	49.10	Bacteria	201111	2483976
Sample_SPC35.mb.102	28.70	Clostridiales	69031	2587326
Sample_SPC35.mb.38cbin.1	54.40	Proteobacteria	65508	2285298
Sample_SPC35.mb.65cbin.1	53.70	Prevotella	66175	2587439
Sample_SPC35.Cluster7717	43.10	Bacteria	39346	4030866
Sample_SPC35.mb.43cbin.1	56.70	Clostridiales	14570	2677683
Sample_SPC35.Cluster8961cbin.1	42.80	Lachnospiraceae	45535	3035015
Sample_SPC35.mb.9	40.90	Clostridiales	28289	2556346
Sample_SPC35.mb.46cbin.1	43.60	Bacteroidales	79027	2911073
Sample_SPC35.mb.88	30.90	Euryarchaeota	112665	1818800
Sample_SPC35.mb.115	44.00	Bacteria	77520	4000623
Sample_SPC35.Cluster1343	57.50	Enterobacteriaceae	258053	5144333
Sample_SPC36.Cluster3901cbin.1	49.50	Clostridiales	86083	2021885
Sample_SPC36.Cluster9499	34.30	Clostridiales	7324	1912864
Sample_SPC36.mb.61	53.50	Clostridia	44585	1900474
Sample_SPC36.mb.4	58.50	Clostridiales	6823	2582721
Sample_SPC36.Cluster635cbin.1	55.80	Clostridiales	8860	2032257
Sample_SPC36.Cluster303	59.20	Clostridiales	49037	1747842
Sample_SPC36.Cluster790cbin.1	59.00	Clostridiales	60023	2038785
Sample_SPC36.Cluster1804	53.80	Clostridiales	29992	2124375
Sample_SPC36.mb.63cbin.1	45.10	Clostridiales	6748	2065847
Sample_SPC36.mb.75	62.80	Clostridiales	22936	1948887
Sample_SPC36.Cluster5498cbin.1	47.20	Selenomonadales	18273	2174893
Sample_SPC36.mb.35	60.70	Clostridiales	19207	2323473
Sample_SPC36.Cluster8252	40.70	Clostridiales	174402	2102920
Sample_SPC36.Cluster3019	52.80	Clostridiales	156599	2267718
Sample_SPC36.mb.62cbin.1	49.30	Bacteroidales	72947	2765499

Sample_SPC36.Cluster1077cbin.1	59.60	Bifidobacteriaceae	146740	1907587
Sample_SPC36.Cluster7181	44.00	Clostridiales	118115	2799095
Sample_SPC36.mb.20	41.20	Clostridiales	178804	2566803
Sample_SPC36.Cluster9568	36.30	Clostridiales	196759	2794081
Sample_SPC36.mb.58	42.80	Lachnospiraceae	38652	2428849
Sample_SPC36.mb.60cbin.1	45.10	Prevotella	67982	3429443
Sample_SPC36.mb.71	43.40	Lachnospiraceae	209162	3173658
Sample_SPC36.mb.87cbin.1	31.30	Firmicutes	75834	2379101
Sample_SPC36.mb.80cbin.1	56.10	Enterobacteriaceae	91577	4295282
Sample_SPC37.Cluster13187	26.30	Bacteria	77031	1091092
Sample_SPC37.Cluster13221	25.10	Bacteria	117840	1264274
Sample_SPC37.Cluster13116	29.40	Bacteria	87031	1495530
Sample_SPC37.Cluster4582cbin.1	53.10	Clostridia	93503	2022385
Sample_SPC37.mb.1cbin.1	53.80	Clostridia	8479	1545586
Sample_SPC37.Cluster7281	45.30	Clostridiales	118210	1663561
Sample_SPC37.Cluster100cbin.1	62.20	Clostridiales	7481	1493339
Sample_SPC37.mb.39cbin.1	48.90	Clostridia	8893	2319839
Sample_SPC37.mb.42cbin.1	49.40	Clostridiales	45013	2149138
Sample_SPC37.Cluster450cbin.1	58.60	Clostridiales	6285	2028811
Sample_SPC37.mb.10	56.80	Clostridiales	23657	2395573
Sample_SPC37.mb.31	56.90	Clostridiales	19992	2471910
Sample_SPC37.mb.34	56.50	Clostridiales	19633	2198410
Sample_SPC37.Cluster4758	52.80	Clostridiales	146604	2252818
Sample_SPC37.mb.20	47.10	Clostridiales	12929	2012118
Sample_SPC37.mb.38	58.90	Bacteroidetes	13071	2450302
Sample_SPC37.Cluster9270cbin.1	44.40	Clostridiales	141121	2241191
Sample_SPC37.mb.21	59.60	Clostridiales	46609	2290700
Sample_SPC37.mb.57cbin.1	48.00	Clostridiales	48805	3147231
Sample_SPC37.Cluster9106	43.90	Clostridiales	302061	2851437
Sample_SPC37.mb.93	49.50	Clostridiales	73382	1912231
Sample_SPC37.mb.68cbin.1	56.90	Clostridiales	28275	1858873
Sample_SPC37.Cluster2120cbin.1	53.90	Bacteroidales	60942	2544425
Sample_SPC37.Cluster7965cbin.1	42.00	Lachnospiraceae	6289	2018575
Sample_SPC37.mb.61	54.50	Clostridiales	8252	1542645
Sample_SPC37.mb.60cbin.1	41.10	Clostridiales	13562	1901779
Sample_SPC37.mb.67	59.90	Actinobacteria	27839	2065007
Sample_SPC37.mb.64	61.00	Clostridiales	8092	2146292
Sample_SPC37.mb.59	54.60	Bacteroidetes	95461	2298327
Sample_SPC37.Cluster9006cbin.1	42.50	Bacteroidales	11597	3757062
Sample_SPC37.mb.91cbin.1	48.60	Firmicutes	34576	1855257
Sample_SPC37.mb.44	46.40	Bacteroidales	93450	3303088
Sample_SPC37.mb.78	59.20	Bacteroidetes	73128	2901956
Sample_SPC37.mb.71	60.20	Deltaproteobacteria	12014	3912210
Sample_SPC37.mb.15cbin.1	45.50	Bacteroidales	11463	4152139
Sample_SPC37.mb.25	41.60	Bacteroidales	48938	4489710
Sample_SPC37.mb.9cbin.1	40.70	Lachnospiraceae	73076	3229347
Sample_SPC37.mb.8	43.50	Bacteroidales	80178	3141908
Sample_SPC37.mb.72	46.80	Bacteroidales	69619	4153587
Sample_SPC37.mb.16cbin.1	50.80	Enterobacteriaceae	25656	4823998
Sample_SPC37.mb.63cbin.1	41.40	Bacteroides	92301	4976639
Sample_SPC38.Cluster9960cbin.1	36.90	Clostridiales	15160	2612124
Sample_SPC38.Cluster169	59.60	Clostridiales	63590	1855362
Sample_SPC38.mb.34	36.30	Clostridiales	161885	2523913
Sample_SPC38.mb.95	26.90	Bacteria	37022	1288354
Sample_SPC38.mb.23	60.30	Clostridiales	28226	1958608
Sample_SPC38.mb.29	59.10	Clostridiales	25736	1976863
Sample_SPC38.mb.38cbin.1	56.90	Clostridiales	11048	2167687
Sample_SPC38.mb.36	61.70	Clostridiales	18247	1908162

Sample_SPC38.mb.81cbin.1	58.00	Clostridiales	51925	3119267
Sample_SPC38.Cluster3558	49.70	Clostridiales	174728	1804005
Sample_SPC38.Cluster8932	39.50	Clostridiales	97101	2047414
Sample_SPC38.mb.30	60.70	Clostridiales	16494	2287184
Sample_SPC38.mb.82	41.20	Clostridiales	49942	3285993
Sample_SPC38.mb.47	60.40	Bacteroidetes	133634	2249957
Sample_SPC38.Cluster128cbin.1	55.60	Bacteria	37755	2861461
Sample_SPC38.mb.50	37.70	Clostridiales	151955	2045011
Sample_SPC38.mb.78	54.90	Bacteroidetes	47643	1882205
Sample_SPC38.mb.63	43.50	Selenomonadales	132582	2370304
Sample_SPC38.Cluster5767cbin.1	45.00	Clostridiales	42794	2870941
Sample_SPC38.mb.12	41.20	Lachnospiraceae	125704	2652517
Sample_SPC38.mb.51	43.90	Clostridiales	244952	2876545
Sample_SPC38.mb.43	29.20	Bacteria	13163	2987950
Sample_SPC38.mb.39	49.30	Proteobacteria	29581	2149693
Sample_SPC38.mb.16	41.70	Lachnospiraceae	75991	2467270
Sample_SPC38.mb.11cbin.1	31.30	Firmicutes	51588	2381503
Sample_SPC38.mb.99cbin.1	54.10	Prevotella	67172	2593498
Sample_SPC38.mb.69	43.50	Bacteroidales	259527	2930534
Sample_SPC38.mb.9	43.00	Clostridiales	96582	2674577
Sample_SPC38.mb.68cbin.1	44.20	Bacteroidales	22679	3566984
Sample_SPC38.mb.17	41.80	Bacteroidales	37152	4474744
Sample_SPC38.mb.65cbin.1	42.20	Bacteroides	103913	4132451
Sample_SPC38.mb.42	46.30	Bacteroidales	121017	4438328
Sample_SPC38.mb.89	45.50	Bacteroidales	89407	3964836
Sample_SPC39.mb.28cbin.1	46.50	Bacteria	121997	3935013
Sample_SPC39.mb.54	62.10	Bacteria	14191	2238942
Sample_SPC39.Cluster11745cbin.1	34.10	Clostridiales	21917	2431654
Sample_SPC39.mb.37cbin.1	38.20	Clostridiales	26908	2524479
Sample_SPC39.Cluster7153cbin.1	47.30	Clostridiales	120075	3220885
Sample_SPC39.Cluster776cbin.1	57.30	Bacteria	61434	2456407
Sample_SPC39.mb.15	52.00	Clostridiales	21562	1953666
Sample_SPC39.mb.11cbin.1	46.90	Lactobacillales	77604	1978928
Sample_SPC39.Cluster5880cbin.1	48.50	Firmicutes	32107	2000862
Sample_SPC39.Cluster1686cbin.1	54.20	Bacteroidetes	68993	2230974
Sample_SPC39.mb.61cbin.1	61.80	Clostridiales	12664	1710165
Sample_SPC39.mb.52	60.80	Clostridiales	74365	2221598
Sample_SPC39.mb.33	60.10	Actinobacteria	10446	1945052
Sample_SPC39.Cluster5759	44.20	Clostridiales	269153	2028129
Sample_SPC39.mb.71cbin.1	57.50	Clostridiales	48556	3301563
Sample_SPC39.mb.63cbin.1	62.00	Clostridiales	61410	1928501
Sample_SPC39.mb.102cbin.1	57.60	Clostridiales	61439	3251777
Sample_SPC39.Cluster256	60.50	Bifidobacteriaceae	55315	1899247
Sample_SPC39.mb.27	37.70	Clostridiales	35510	2756257
Sample_SPC39.mb.56cbin.1	33.30	Bacteria	40406	2269195
Sample_SPC39.Cluster11623cbin.1	32.60	Lactobacillales	89704	1998146
Sample_SPC39.mb.21	38.50	Clostridiales	90712	2126440
Sample_SPC39.mb.5cbin.1	60.40	Clostridiales	18286	2372118
Sample_SPC39.Cluster9674cbin.1	37.40	Clostridiales	71154	2762775
Sample_SPC39.Cluster2073	53.20	Selenomonadales	102090	2274532
Sample_SPC39.mb.78cbin.1	49.60	Clostridiales	18163	2227172
Sample_SPC39.Cluster9455cbin.1	41.00	Lachnospiraceae	93786	3101943
Sample_SPC39.mb.74	43.20	Clostridiales	146268	2461867
Sample_SPC39.mb.104	41.70	Clostridiales	12769	3186751
Sample_SPC39.mb.79cbin.1	38.60	Streptococcus	94696	1858989
Sample_SPC39.Cluster5123	45.20	Bacteroidales	83918	3986562
Sample_SPC39.mb.97	44.80	Clostridiales	86391	3009864
Sample_SPC41.Cluster13347	24.30	Bacteria	61161	972204

Sample_SPC41.Cluster13293cbin.1	25.50	Bacteria	77162	1343783
Sample_SPC41.mb.18cbin.1	49.70	Clostridia	9675	1252752
Sample_SPC41.Cluster6548	47.20	Clostridiales	232002	1526132
Sample_SPC41.mb.23	42.80	Clostridia	25408	1310247
Sample_SPC41.mb.101cbin.1	53.70	Clostridiales	9681	1909077
Sample_SPC41.mb.20cbin.1	57.20	Clostridia	52912	2765391
Sample_SPC41.mb.1	58.10	Clostridiales	44760	2992540
Sample_SPC41.mb.102	37.50	Clostridiales	168780	2008737
Sample_SPC41.mb.5	53.40	Clostridia	40436	1830987
Sample_SPC41.mb.11	58.20	Clostridiales	30662	2522795
Sample_SPC41.mb.40	47.90	Clostridiales	126920	1783192
Sample_SPC41.mb.33	49.50	Clostridiales	75718	2124292
Sample_SPC41.mb.16cbin.1	33.30	Clostridiales	151286	2772130
Sample_SPC41.mb.69	47.20	Clostridia	26498	1971882
Sample_SPC41.mb.26	57.30	Clostridiales	56640	1947814
Sample_SPC41.mb.68	57.70	Clostridia	15272	3279527
Sample_SPC41.mb.87	55.00	Clostridia	181726	2450607
Sample_SPC41.Cluster12539	31.10	Euryarchaeota	10552	1537061
Sample_SPC41.mb.83	60.40	Clostridiales	77024	1867490
Sample_SPC41.mb.100	40.90	Clostridiales	14498	1854885
Sample_SPC41.mb.44	55.60	Bacteroidetes	8429	1769977
Sample_SPC41.mb.28	41.80	Clostridiales	38083	2892358
Sample_SPC41.Cluster8115	44.60	Clostridiales	141569	2258805
Sample_SPC41.mb.91	38.40	Clostridiales	22846	4066021
Sample_SPC41.mb.95cbin.1	59.60	Clostridiales	23992	2742663
Sample_SPC41.mb.80cbin.1	52.40	Firmicutes	50752	2229045
Sample_SPC41.mb.94	53.50	Clostridiales	14641	2020492
Sample_SPC41.mb.85	44.20	Clostridiales	135627	2666217
Sample_SPC41.mb.98cbin.1	51.00	Clostridiales	46523	2265717
Sample_SPC41.mb.99cbin.1	41.50	Bacteroidales	26684	5052750
Sample_SPC42.Cluster17162cbin.1	24.80	Bacteria	39140	1301426
Sample_SPC42.mb.101cbin.1	49.90	Clostridia	111248	1896417
Sample_SPC42.Cluster10367cbin.1	43.80	Clostridiales	131663	3057102
Sample_SPC42.mb.114	55.40	Clostridiales	27542	2273344
Sample_SPC42.Cluster7237	46.10	Clostridiales	126468	2198912
Sample_SPC42.Cluster76cbin.1	58.30	Clostridiales	114418	2462059
Sample_SPC42.mb.45	59.40	Clostridiales	7165	1750843
Sample_SPC42.mb.29cbin.1	41.80	Lachnospiraceae	106598	2262379
Sample_SPC42.mb.41	61.50	Clostridiales	139217	1973185
Sample_SPC42.Cluster7381	45.00	Lachnospiraceae	77989	2488385
Sample_SPC42.mb.67	52.00	Clostridiales	9473	1841812
Sample_SPC42.mb.68	38.70	Clostridiales	104881	2745765
Sample_SPC42.mb.112	62.90	Proteobacteria	6730	1747491
Sample_SPC42.Cluster8949cbin.1	43.60	Clostridiales	324771	2799350
Sample_SPC42.Cluster13963cbin.1	32.70	Lactobacillales	28082	2046530
Sample_SPC42.Cluster1138cbin.1	58.60	Bifidobacteriaceae	143711	2385353
Sample_SPC42.Cluster10175	41.30	Lachnospiraceae	89422	2923365
Sample_SPC42.mb.77	42.40	Clostridiales	19610	2471826
Sample_SPC42.mb.110	36.30	Clostridiales	12297	2209610
Sample_SPC42.mb.25	41.90	Clostridiales	88302	2375581
Sample_SPC42.mb.56	51.20	Bacteroidales	55091	2464091
Sample_SPC42.mb.115cbin.1	39.70	Clostridiales	17529	2263096
Sample_SPC42.mb.73cbin.1	61.40	Clostridiales	53876	1936443
Sample_SPC42.mb.62cbin.1	48.10	Bacteroidales	51760	2620930
Sample_SPC42.mb.54	63.50	Actinobacteria	42737	2556727
Sample_SPC42.mb.7cbin.1	47.50	Bacteroidales	24341	2438682
Sample_SPC42.mb.60cbin.1	45.00	Clostridiales	27597	2303408
Sample_SPC42.mb.28	49.40	Lachnospiraceae	67597	3034403

Sample_SPC42.mb.83cbin.1	54.30	Bacteroidetes	149731	2154714
Sample_SPC42.mb.78cbin.1	43.90	Lachnospiraceae	41014	2686630
Sample_SPC42.mb.81	29.20	Bacteria	43540	2658868
Sample_SPC45.Cluster1174cbin.1	57.20	Bacteria	63512	2038549
Sample_SPC45.Cluster1324cbin.1	58.00	Clostridiales	68016	3120709
Sample_SPC45.mb.21cbin.1	29.10	Bacteria	110032	1583542
Sample_SPC45.mb.104	33.60	Clostridiales	199157	1846969
Sample_SPC45.mb.110	49.40	Clostridiales	100142	1968331
Sample_SPC45.mb.11	36.60	Clostridiales	20606	1681820
Sample_SPC45.mb.48cbin.1	53.70	Clostridia	49205	1694179
Sample_SPC45.Cluster13128cbin.1	37.40	Clostridiales	30498	2107173
Sample_SPC45.Cluster2583	54.80	Bacteroidetes	125115	1997829
Sample_SPC45.Cluster7266	46.70	Selenomonadales	72421	2289221
Sample_SPC45.mb.28cbin.1	57.10	Clostridiales	92606	1921209
Sample_SPC45.mb.46cbin.1	61.80	Clostridiales	49926	1848481
Sample_SPC45.mb.18	54.30	Clostridiales	18268	1660773
Sample_SPC45.Cluster4017cbin.1	50.80	Bacteroidales	33936	2145975
Sample_SPC45.mb.7	53.10	Clostridia	54225	2024147
Sample_SPC45.Cluster1974	55.40	Bacteria	90493	2787234
Sample_SPC45.mb.1	39.10	Clostridiales	28161	2189755
Sample_SPC45.mb.65	44.30	Clostridiales	21051	2134391
Sample_SPC45.mb.67	50.00	Clostridiales	35351	2312379
Sample_SPC45.mb.91	44.20	Clostridia	21407	1961052
Sample_SPC45.mb.25	36.10	Clostridiales	31001	2394496
Sample_SPC45.mb.22	43.80	Lachnospiraceae	66382	2708104
Sample_SPC45.mb.77	53.70	Clostridiales	18250	1918846
Sample_SPC45.mb.49	51.40	Clostridiales	224379	2278805
Sample_SPC45.Cluster11681cbin.1	43.00	Bacteria	77179	4096766
Sample_SPC45.mb.84	46.70	Clostridiales	12775	2543679
Sample_SPC45.mb.73cbin.1	38.00	Clostridiales	75964	1858210
Sample_SPC45.mb.2cbin.1	48.70	Proteobacteria	20511	2468778
Sample_SPC45.mb.64	40.60	Clostridiales	192876	2129882
Sample_SPC45.mb.56cbin.1	41.00	Clostridiales	23609	2535666
Sample_SPC45.mb.99cbin.1	47.80	Clostridiales	111677	3114625
Sample_SPC45.mb.87	59.30	Clostridiales	60926	2300314
Sample_SPC45.mb.89	47.00	Lachnospiraceae	24129	3073036
Sample_SPC45.Cluster6907cbin.1	46.40	Bacteroidales	96819	4561818
Sample_SPC45.mb.98	46.80	Clostridiales	9491	1859295
Sample_SPC45.mb.17	45.50	Bacteroidales	104144	3881256
Sample_SPC45.mb.88cbin.1	58.40	Bacteroidetes	206071	3576339
Sample_SPC45.mb.72cbin.1	45.10	Bacteroidales	10474	4169422
Sample_SPC45.Cluster911cbin.1	50.80	Enterobacteriaceae	44526	4616174
Sample_SPC47.Cluster2599cbin.1	49.30	Clostridiales	10327	1820366
Sample_SPC47.Cluster3860cbin.1	48.00	Clostridiales	13966	1872142
Sample_SPC47.mb.11	34.30	Clostridiales	14414	2154140
Sample_SPC47.Cluster374cbin.1	57.90	Clostridiales	49611	2926406
Sample_SPC47.mb.31	61.00	Clostridiales	69256	2196058
Sample_SPC47.Cluster1897	53.80	Clostridiales	202102	1837072
Sample_SPC47.mb.10cbin.1	46.50	Selenomonadales	9468	1585807
Sample_SPC47.mb.5	61.90	Actinobacteria	7411	1964921
Sample_SPC47.mb.57cbin.1	48.10	Clostridiales	25443	2742274
Sample_SPC47.Cluster1045	55.50	Proteobacteria	148886	2367055
Sample_SPC47.mb.43	40.20	Clostridiales	31294	3171301
Sample_SPC47.mb.29cbin.1	38.90	Clostridiales	77301	1895372
Sample_SPC47.mb.101cbin.1	56.50	Bifidobacteriaceae	276407	2049818
Sample_SPC47.mb.21	44.20	Clostridiales	84206	2692371
Sample_SPC47.mb.61	58.60	Bacteroidetes	9342	2282157
Sample_SPC47.Cluster11991cbin.1	31.10	Firmicutes	87757	2066703

Sample_SPC47.mb.39cbin.1	60.10	Bifidobacteriaceae	24270	2247868
Sample_SPC47.Cluster170cbin.1	59.00	Bacteroidetes	19756	2669067
Sample_SPC47.mb.73cbin.1	58.70	Clostridiales	17594	2288036
Sample_SPC47.Cluster9834cbin.1	41.10	Lachnospiraceae	92829	3070413
Sample_SPC47.mb.77cbin.1	54.30	Bacteroidetes	80045	2039933
Sample_SPC47.mb.52	42.10	Clostridiales	10143	2384966
Sample_SPC47.mb.96cbin.1	56.90	Clostridiales	56112	2212957
Sample_SPC47.Cluster3686cbin.1	46.70	Bacteroidales	219727	3175786
Sample_SPC47.mb.12	43.40	Bacteroidales	81065	3036323
Sample_SPC47.mb.25	46.70	Bacteroidales	68001	3169370
Sample_SPC47.Cluster6069cbin.1	43.30	Bacteria	82085	4037013
Sample_SPC47.mb.66	61.70	Proteobacteria	46716	2373293
Sample_SPC47.mb.18cbin.1	43.00	Bacteria	8001	3809057
Sample_SPC47.Cluster3793	45.20	Bacteroidales	70829	4309533
Sample_SPC47.mb.63	42.10	Bacteroidales	63812	3562795
Sample_SPC47.Cluster5874cbin.1	45.20	Bacteroidales	20090	4356713
Sample_SPC47.mb.84	62.10	Clostridiales	15470	3375155
Sample_SPC47.mb.76cbin.1	41.60	Lachnospiraceae	67294	2531301
Sample_SPC47.mb.74	50.90	Enterobacteriaceae	29997	4407132
Sample_YPA1.mb.4cbin.1	45.40	Selenomonadales	14554	1687998
Sample_YPA1.mb.7	58.20	Clostridiales	79325	2358071
Sample_YPA1.Cluster3	60.10	Bifidobacteriaceae	56784	2141868
Sample_YPA1.Cluster770cbin.1	38.50	Selenomonadales	48583	2172466
Sample_YPA1.mb.17	49.10	Proteobacteria	34079	2096149
Sample_YPA1.Cluster2cbin.1	61.50	Clostridiales	33963	3611614
Sample_YPA1.Cluster14cbin.1	57.70	Clostridiales	52839	2885023
Sample_YPA1.Cluster736cbin.1	42.80	Lachnospiraceae	72529	3157188
Sample_YPA1.Cluster346	46.30	Bacteroidales	130327	3866823
Sample_YPA1.mb.5	48.20	Clostridiales	8946	4151092
Sample_YPA1.Cluster642cbin.1	42.70	Bacteroides	204710	5711805
Sample_YPA2.Cluster7617	37.50	Clostridiales	13742	1838092
Sample_YPA2.Cluster111	59.30	Clostridiales	8793	1877183
Sample_YPA2.Cluster444cbin.1	59.00	Clostridiales	43063	1936558
Sample_YPA2.mb.21	33.20	Clostridiales	46423	2557910
Sample_YPA2.mb.71	26.00	Bacteria	40239	1236524
Sample_YPA2.Cluster967cbin.1	58.30	Clostridiales	21281	1935974
Sample_YPA2.mb.17cbin.1	48.70	Clostridiales	23015	2529573
Sample_YPA2.mb.22	61.90	Clostridiales	8148	1918231
Sample_YPA2.mb.31	51.30	Clostridiales	22358	2275615
Sample_YPA2.mb.69cbin.1	58.00	Clostridiales	41577	3032722
Sample_YPA2.mb.6	36.30	Clostridiales	168562	2519624
Sample_YPA2.mb.55	41.30	Clostridiales	9129	3130879
Sample_YPA2.mb.7	57.40	Clostridiales	7572	1996521
Sample_YPA2.Cluster964cbin.1	54.60	Bacteroidetes	48576	2114830
Sample_YPA2.Cluster10058	37.70	Clostridiales	209030	2100509
Sample_YPA2.Cluster9010cbin.1	40.60	Clostridiales	357458	2043167
Sample_YPA2.mb.61	44.80	Clostridiales	32726	2566982
Sample_YPA2.mb.26	60.30	Clostridiales	58173	2461186
Sample_YPA2.mb.23cbin.1	56.40	Clostridiales	14821	2452117
Sample_YPA2.mb.45cbin.1	44.00	Selenomonadales	21023	2204637
Sample_YPA2.Cluster755	54.40	Bacteroidales	49223	2380938
Sample_YPA2.Cluster6851cbin.1	43.20	Clostridiales	129279	2480411
Sample_YPA2.mb.15cbin.1	56.60	Bifidobacteriaceae	16089	1995304
Sample_YPA2.Cluster7305	38.40	Bacteroidales	7743	2407749
Sample_YPA2.mb.5	44.90	Lachnospiraceae	95872	2455203
Sample_YPA2.Cluster8664cbin.1	37.30	Clostridiales	103604	2833055
Sample_YPA2.mb.18cbin.1	44.90	Clostridiales	60952	2831355
Sample_YPA2.mb.75	36.70	Clostridiales	55138	2095797

Sample_YPA2.mb.72cbin.1	41.30	Lachnospiraceae	86349	2981780
Sample_YPA2.mb.37	45.10	Bacteroidales	149934	4660367
Sample_YPA3.mb.22cbin.1	41.60	Bacteria	67435	2542625
Sample_YPA3.Cluster4908	43.80	Clostridiales	83804	2758527
Sample_YPA3.mb.55	38.00	Clostridiales	7771	2894615
Sample_YPA3.mb.40cbin.1	58.00	Clostridiales	5006	1910935
Sample_YPA3.mb.54cbin.1	54.50	Actinobacteria	23934	1552449
Sample_YPA3.mb.50	56.30	Clostridiales	31982	2737611
Sample_YPA3.mb.48cbin.1	60.80	Clostridiales	108130	2277263
Sample_YPA3.Cluster38cbin.1	58.50	Clostridiales	95584	2345348
Sample_YPA3.mb.61cbin.1	61.60	Clostridiales	24463	2104943
Sample_YPA3.Cluster4466	45.10	Clostridiales	154765	1640887
Sample_YPA3.Cluster2815cbin.1	46.00	Bacteroidales	115361	3301209
Sample_YPA3.mb.66cbin.1	57.60	Clostridiales	46056	2733661
Sample_YPA3.mb.4	38.10	Clostridiales	209589	1849484
Sample_YPA3.Cluster806cbin.1	55.20	Bacteroidetes	85839	2647948
Sample_YPA3.Cluster5432	40.80	Clostridiales	182836	2153218
Sample_YPA3.mb.5cbin.1	54.10	Bacteroidetes	91395	2346228
Sample_YPA3.mb.52cbin.1	51.30	Clostridiales	134165	2196610
Sample_YPA3.mb.19cbin.1	45.90	Clostridiales	50928	2621196
Sample_YPA3.Cluster1349	53.00	Clostridiales	53645	2621366
Sample_YPA3.mb.68	57.30	Clostridiales	85742	2779013
Sample_YPA3.mb.58	58.10	Clostridiales	22163	2355482
Sample_YPA3.mb.46	43.10	Lachnospiraceae	19952	3196648
Sample_YPA3.Cluster4437cbin.1	46.40	Bacteroidales	184702	4130127
Sample_YPA3.Cluster3753	45.10	Bacteroidales	170216	4724053
Sample_YPA3.mb.7	41.80	Bacteroides	99896	4474812
Sample_YPA4.mb.11cbin.1	42.60	Clostridiales	32715	2620200
Sample_YPA4.Cluster5859	43.70	Clostridiales	102075	2769630
Sample_YPA4.mb.19	57.00	Clostridiales	39304	2379728
Sample_YPA4.mb.30cbin.1	54.60	Actinobacteria	14275	1481790
Sample_YPA4.Cluster452cbin.1	60.40	Clostridiales	100134	1960256
Sample_YPA4.mb.1	58.50	Clostridiales	12597	1813807
Sample_YPA4.Cluster139	61.60	Clostridiales	54964	1974941
Sample_YPA4.mb.22cbin.1	48.60	Clostridiales	10334	2631577
Sample_YPA4.mb.26	61.10	Clostridiales	100612	2183007
Sample_YPA4.Cluster45cbin.1	58.40	Clostridiales	81645	2361444
Sample_YPA4.mb.47	37.30	Clostridiales	28952	3936673
Sample_YPA4.Cluster5769cbin.1	47.80	Firmicutes	37651	1408812
Sample_YPA4.Cluster3321cbin.1	55.60	Bacteroidetes	5877	2142569
Sample_YPA4.Cluster7833	38.20	Clostridiales	222540	1884159
Sample_YPA4.Cluster3723cbin.1	45.50	Bacteroidales	121144	3437045
Sample_YPA4.Cluster7082	40.60	Clostridiales	182836	2086502
Sample_YPA4.mb.50	58.50	Clostridiales	22090	2175704
Sample_YPA4.mb.52	55.00	Bacteroidetes	118107	1972884
Sample_YPA4.mb.46cbin.1	34.70	Lactobacillus	6469	1641634
Sample_YPA4.mb.58	56.60	Clostridiales	6812	2164695
Sample_YPA4.mb.65cbin.1	57.70	Clostridiales	54939	2885011
Sample_YPA4.Cluster7499	37.40	Clostridiales	59120	2748789
Sample_YPA4.mb.70	51.50	Clostridiales	108059	2047879
Sample_YPA4.mb.43cbin.1	55.70	Clostridiales	60544	3376711
Sample_YPA4.mb.9cbin.1	46.10	Clostridiales	46088	2611093
Sample_YPA4.mb.63cbin.1	41.70	Lachnospiraceae	61928	2601218
Sample_YPA4.mb.15cbin.1	46.40	Bacteroidales	161622	4171730
Sample_YPA4.mb.64cbin.1	45.00	Bacteroidales	138215	4732895
Sample_YPA5.Cluster28cbin.1	60.80	Clostridiales	22447	1810843
Sample_YPA5.Cluster1676cbin.1	50.90	Bacteroidales	11890	2066121
Sample_YPA5.mb.19cbin.1	59.40	Clostridiales	41695	2345720

Sample_YPA5.mb.14	56.20	Clostridiales	70673	2269613
Sample_YPA5.mb.16	51.00	Clostridiales	35452	2311337
Sample_YPA5.Cluster7017	40.70	Clostridiales	130461	1705605
Sample_YPA5.mb.22cbin.1	45.20	Clostridiales	103548	2681441
Sample_YPA5.Cluster3216	46.90	Proteobacteria	73892	1864301
Sample_YPA5.Cluster1060	55.60	Proteobacteria	195704	2236725
Sample_YPA5.Cluster19	63.50	Deltaproteobacteria	58843	2648937
Sample_YPA5.mb.52cbin.1	62.00	Clostridiales	165101	2023352
Sample_YPA5.mb.23	46.30	Bacteroidetes	22086	2422220
Sample_YPA5.mb.69	57.90	Clostridiales	69707	2219654
Sample_YPA5.Cluster577cbin.1	59.20	Bacteroidetes	164558	2676305
Sample_YPA5.mb.39	46.90	Lachnospiraceae	41159	3204646
Sample_YPA5.Cluster4724	41.70	Lachnospiraceae	93555	2913603
Sample_YPA5.mb.29	40.90	Clostridiales	21323	2505377
Sample_YPA5.mb.13cbin.1	50.20	Bacteroidales	69951	2387937
Sample_YPA5.mb.47cbin.1	51.00	Firmicutes	42643	2358502
Sample_YPA5.mb.8	41.00	Lachnospiraceae	90252	2543097
Sample_YPA5.mb.7	41.30	Clostridiales	188379	2564571
Sample_YPA5.mb.81	54.20	Bacteroidetes	66960	2101061
Sample_YPA5.Cluster1993cbin.1	48.50	Bacteroidales	34548	3490628
Sample_YPA5.mb.28	40.70	Bacteroidales	47629	3018383
Sample_YPA5.mb.43	41.90	Lachnospiraceae	57872	2384421
Sample_YPA5.mb.38cbin.1	55.60	Clostridiales	25878	3426417
Sample_YPA5.mb.68	49.50	Lachnospiraceae	124773	2941354
Sample_YPA5.mb.30cbin.1	44.10	Bacteroidales	36558	3701538
Sample_YPA5.mb.74cbin.1	37.00	Clostridiales	20300	3117866
Sample_YPA5.Cluster2191cbin.1	45.20	Bacteroidales	41990	4401295
Sample_YPA5.Cluster3451cbin.1	44.90	Bacteroidales	138341	4789321
Sample_YPA5.mb.40	46.70	Bacteroidales	122612	3925641
Sample_YPA6.Cluster5179cbin.1	48.60	Clostridia	57740	2207385
Sample_YPA6.mb.53	26.70	Bacteria	97525	1306762
Sample_YPA6.Cluster7899	45.30	Clostridiales	157844	1741174
Sample_YPA6.Cluster81cbin.1	61.70	Clostridiales	29528	1582467
Sample_YPA6.mb.10	59.30	Clostridiales	29437	2255426
Sample_YPA6.mb.42	36.20	Clostridiales	119088	2873882
Sample_YPA6.Cluster3578cbin.1	51.40	Clostridiales	130101	2189032
Sample_YPA6.mb.63	59.50	Clostridiales	11426	1734408
Sample_YPA6.mb.13	51.20	Clostridiales	199319	2222057
Sample_YPA6.mb.47	56.60	Clostridiales	7956	2406082
Sample_YPA6.mb.60	46.70	Lachnospiraceae	69964	2888020
Sample_YPA6.mb.43	44.60	Clostridiales	20881	2102931
Sample_YPA6.mb.28	41.00	Lachnospiraceae	7921	2631564
Sample_YPA6.Cluster1155	58.40	Bacteroidetes	279190	3232278
Sample_YPA6.mb.61	43.20	Lachnospiraceae	104675	2916751
Sample_YPA6.mb.27cbin.1	48.30	Proteobacteria	9762	2453660
Sample_YPA6.mb.12cbin.1	43.10	Bacteria	52868	4097638
Sample_YPA6.mb.34	46.70	Bacteroidales	230535	4203522
Sample_YPA6.mb.24	45.10	Bacteroidales	150063	4620584
Sample_YPA6.mb.58	41.90	Bacteroides	64775	4703976
Sample_YPA7.mb.29	24.90	Bacteria	48149	1538709
Sample_YPA7.mb.45	27.80	Bacteria	17593	996772
Sample_YPA7.mb.19	27.10	Clostridiales	230245	1818628
Sample_YPA7.mb.18	38.60	Clostridiales	24221	2454689
Sample_YPA7.mb.32cbin.1	52.10	Clostridiales	20948	2348196
Sample_YPA7.Cluster910cbin.1	50.00	Bacteroidales	35574	2428480
Sample_YPA7.Cluster251cbin.1	55.20	Bacteroidetes	13115	2562545
Sample_YPA7.mb.42	49.50	Clostridiales	7001	2187806
Sample_YPA7.mb.69	48.20	Clostridiales	6754	1607463

Sample_YPA7.Cluster3867cbin.1	44.10	Clostridiales	156519	2020786
Sample_YPA7.Cluster3122	43.50	Selenomonadales	285985	2419053
Sample_YPA7.mb.16	56.70	Clostridiales	13179	2365915
Sample_YPA7.mb.24	41.20	Clostridiales	47633	1943935
Sample_YPA7.mb.31cbin.1	59.70	Clostridiales	36077	2251483
Sample_YPA7.Cluster628	55.40	Bacteria	119094	2900186
Sample_YPA7.mb.26cbin.1	46.20	Clostridiales	47206	2379487
Sample_YPA7.mb.35	43.30	Lachnospiraceae	37293	2080585
Sample_YPA7.mb.1	43.80	Lachnospiraceae	112597	2617985
Sample_YPA7.mb.60cbin.1	60.40	Clostridiales	32859	3028282
Sample_YPA7.Cluster176cbin.1	58.80	Bacteroidetes	242731	3009176
Sample_YPA7.mb.9	59.70	Clostridiales	104081	2118706
Sample_YPA7.Cluster2391cbin.1	44.40	Bacteroidales	122773	3327453
Sample_YPA7.mb.7	60.50	Clostridiales	16725	2315512
Sample_YPA7.mb.54	37.50	Clostridiales	147031	2758510
Sample_YPA7.mb.80	51.30	Clostridiales	121128	2244710
Sample_YPA7.mb.85	41.10	Clostridiales	213749	2623949
Sample_YPA7.mb.63cbin.1	44.80	Prevotella	58511	3685937
Sample_YPA7.mb.81	41.60	Lachnospiraceae	74467	2681807
Sample_YPA7.Cluster3591cbin.1	45.20	Bacteroidales	100154	4326540
Sample_YPA7.Cluster7303cbin.1	42.90	Bacteria	11022	4594755
Sample_YPA7.Cluster2095	44.90	Bacteroidales	79955	4692852
Sample_YPA7.mb.64	41.00	Lachnospiraceae	24098	3853171
Sample_YPA7.mb.76	42.30	Bacteroidales	63608	3222367
Sample_YPA7.mb.22cbin.1	41.60	Bacteroidales	76712	4802492
Sample_YPA7.mb.67	41.40	Lachnospiraceae	50911	4150807
Sample_YPA7.mb.62	46.50	Bacteroidales	119232	4150367
Sample_YPB1.Cluster1591	46.70	Clostridiales	87871	1777021
Sample_YPB1.mb.28cbin.1	59.60	Clostridiales	101574	2050657
Sample_YPB1.Cluster327cbin.1	58.40	Clostridiales	133443	2157046
Sample_YPB1.Cluster1720	46.80	Lactobacillales	170385	2038631
Sample_YPB1.Cluster198cbin.1	56.40	Clostridiales	14032	2266106
Sample_YPB1.Cluster782cbin.1	48.40	Firmicutes	38366	1948463
Sample_YPB1.Cluster708	53.00	Clostridiales	220472	1922695
Sample_YPB1.Cluster3867	40.50	Clostridiales	160685	2143353
Sample_YPB1.mb.77	37.70	Clostridiales	34158	4635425
Sample_YPB1.Cluster425cbin.1	56.80	Bifidobacteriaceae	194170	1823499
Sample_YPB1.mb.6cbin.1	59.90	Actinobacteria	104547	2183754
Sample_YPB1.mb.16	58.50	Clostridiales	45503	2439835
Sample_YPB1.mb.21	62.90	Bifidobacteriaceae	130506	2088771
Sample_YPB1.mb.39	34.80	Lactobacillus	110268	1952441
Sample_YPB1.Cluster447cbin.1	58.60	Bacteroidetes	66811	3114729
Sample_YPB1.mb.23	60.10	Bifidobacteriaceae	55761	2077057
Sample_YPB1.mb.59cbin.1	56.50	Clostridiales	55483	2834807
Sample_YPB1.mb.38	43.20	Clostridiales	114640	2456379
Sample_YPB1.mb.8cbin.1	57.20	Proteobacteria	94918	2441976
Sample_YPB1.mb.17cbin.1	45.50	Prevotella	52834	3643269
Sample_YPB1.Cluster1122cbin.1	49.30	Lachnospiraceae	94440	3015152
Sample_YPB1.mb.36	44.60	Clostridiales	39743	3055189
Sample_YPB1.mb.49cbin.1	41.10	Lachnospiraceae	113359	3026480
Sample_YPB1.Cluster2259cbin.1	43.10	Bacteria	11131	3835938
Sample_YPB1.Cluster1036cbin.1	46.70	Bacteroidales	80151	4050920
Sample_YPB1.mb.32	41.60	Bacteroidales	36360	5182440
Sample_YPB1.mb.44cbin.1	41.70	Bacteroides	108420	5996150
Sample_YPB2.Cluster12916	25.50	Bacteria	197846	1137348
Sample_YPB2.Cluster12477	29.50	Bacteria	126671	1474787
Sample_YPB2.Cluster11555	36.50	Clostridiales	363704	1889485
Sample_YPB2.Cluster5718cbin.1	43.90	Clostridiales	20588	2697535

Sample_YPB2.mb.48	27.40	Bacteria	28409	1567463
Sample_YPB2.mb.21	38.30	Clostridiales	8506	2076674
Sample_YPB2.Cluster87	59.80	Clostridiales	38060	1758846
Sample_YPB2.Cluster2520	53.10	Clostridiales	126460	1833504
Sample_YPB2.mb.15	60.90	Clostridiales	36998	2319994
Sample_YPB2.mb.24cbin.1	53.00	Clostridiales	56193	2129023
Sample_YPB2.Cluster9399cbin.1	40.80	Lachnospiraceae	138548	2606803
Sample_YPB2.mb.32cbin.1	47.80	Clostridiales	129659	3093258
Sample_YPB2.Cluster1278	55.60	Proteobacteria	115829	2181164
Sample_YPB2.mb.4cbin.1	32.40	Clostridiales	8926	4162553
Sample_YPB2.mb.5cbin.1	57.90	Clostridiales	60293	3177614
Sample_YPB2.Cluster1528cbin.1	58.50	Bacteroidetes	89978	3224636
Sample_YPB2.mb.93cbin.1	49.50	Clostridiales	133304	1944001
Sample_YPB2.Cluster9659	40.20	Clostridiales	326056	2231698
Sample_YPB2.mb.56	45.10	Clostridiales	118636	2676025
Sample_YPB2.mb.1	42.90	Lachnospiraceae	44188	2332793
Sample_YPB2.mb.6	46.20	Bacteroidetes	12760	2210978
Sample_YPB2.mb.58cbin.1	56.50	Clostridiales	12552	2428577
Sample_YPB2.mb.73	60.00	Actinobacteria	29157	2134287
Sample_YPB2.mb.27cbin.1	40.80	Lachnospiraceae	72052	3182510
Sample_YPB2.mb.74	51.90	Firmicutes	31659	2216675
Sample_YPB2.mb.83	44.00	Lachnospiraceae	87814	2668258
Sample_YPB2.mb.67cbin.1	56.30	Bifidobacteriaceae	392571	2107752
Sample_YPB2.mb.69	45.30	Clostridiales	84029	2220425
Sample_YPB2.mb.72	46.80	Lachnospiraceae	26501	3292483
Sample_YPB2.Cluster3860	45.40	Bacteroidales	59549	3962070
Sample_YPB2.mb.75cbin.1	44.30	Prevotella	25371	3884175
Sample_YPB2.Cluster947	56.50	Pseudomonadales	278233	5173266
Sample_YPB3.Cluster3030cbin.1	43.90	Clostridiales	9244	2681939
Sample_YPB3.mb.31	58.10	Clostridiales	6914	1959217
Sample_YPB3.mb.59	58.30	Clostridia	30381	2958982
Sample_YPB3.mb.47cbin.1	44.80	Clostridiales	29272	2671141
Sample_YPB3.Cluster1cbin.1	66.30	Actinobacteria	218894	2060869
Sample_YPB3.Cluster1244	51.80	Clostridiales	120033	2327695
Sample_YPB3.Cluster2914	47.10	Lactobacillales	125528	1846833
Sample_YPB3.mb.39cbin.1	48.30	Clostridiales	39080	2904857
Sample_YPB3.mb.8	59.60	Clostridiales	7124	1673766
Sample_YPB3.mb.20	58.90	Bacteroidetes	15909	2368096
Sample_YPB3.Cluster4065	41.00	Clostridiales	182350	1836521
Sample_YPB3.mb.26	56.40	Clostridiales	14118	2518550
Sample_YPB3.Cluster145cbin.1	51.80	Clostridiales	66072	2260931
Sample_YPB3.Cluster33cbin.1	60.00	Bifidobacteriaceae	65363	2035029
Sample_YPB3.mb.40	45.30	Clostridiales	10681	2196423
Sample_YPB3.Cluster1000	56.30	Selenomonadales	129884	2216156
Sample_YPB3.Cluster867cbin.1	54.10	Selenomonadales	44988	2325139
Sample_YPB3.Cluster1114cbin.1	56.00	Bifidobacteriaceae	145711	2058229
Sample_YPB3.Cluster198	59.70	Bacteroidetes	32495	2750931
Sample_YPB3.Cluster2149cbin.1	51.40	Prevotella	87236	3127462
Sample_YPB3.Cluster2564	46.70	Bacteroidales	240487	4107133
Sample_YPB4.Cluster6185	27.80	Bacteria	309168	1318263
Sample_YPB4.mb.26cbin.1	38.40	Clostridiales	6922	2053041
Sample_YPB4.Cluster2405	44.00	Clostridiales	174255	2890254
Sample_YPB4.Cluster2143	41.60	Clostridiales	132931	2827176
Sample_YPB4.Cluster3798	40.90	Clostridiales	81253	3098333
Sample_YPB4.mb.5cbin.1	44.60	Clostridiales	19067	2606820
Sample_YPB4.Cluster1733	46.90	Lactobacillales	87825	1658709
Sample_YPB4.mb.68	48.20	Clostridiales	60375	2952667
Sample_YPB4.mb.8cbin.1	51.50	Clostridiales	120001	2450194

Sample_YPB4.Cluster16	60.30	Clostridiales	98185	2311180
Sample_YPB4.Cluster60cbin.1	56.20	Clostridiales	15752	2599965
Sample_YPB4.Cluster342	51.60	Clostridiales	58866	2194962
Sample_YPB4.mb.62	43.50	Lachnospiraceae	18289	1865402
Sample_YPB4.mb.43cbin.1	44.20	Clostridiales	8947	2907651
Sample_YPB4.Cluster4709cbin.1	37.80	Clostridiales	113816	2037245
Sample_YPB4.mb.19cbin.1	56.40	Selenomonadales	102929	2167489
Sample_YPB4.Cluster3580	40.60	Clostridiales	247706	2164376
Sample_YPB4.mb.30cbin.1	54.20	Selenomonadales	41950	2208062
Sample_YPB4.mb.34	34.60	Lactobacillus	325310	1806248
Sample_YPB4.Cluster5996	32.50	Lactobacillales	109568	1861715
Sample_YPB4.mb.29cbin.1	44.80	Clostridiales	25645	2507059
Sample_YPB4.Cluster170	56.10	Bifidobacteriaceae	283979	2162616
Sample_YPB4.mb.60cbin.1	60.30	Bifidobacteriaceae	103967	2382984
Sample_YPB4.mb.55	50.20	Lachnospiraceae	13668	2615758
Sample_YPB4.mb.51cbin.1	51.10	Prevotella	83514	3158703
Sample_YPB4.Cluster1428	46.40	Bacteroidales	222748	4376915
Sample_YPB4.mb.13	45.10	Bacteroidales	19154	4606859
Sample_YPB4.mb.11cbin.1	41.40	Bacteroidales	31144	5075459
Sample_YPB5.Cluster8314cbin.1	26.30	Bacteria	9432	1042359
Sample_YPB5.Cluster5620cbin.1	39.00	Clostridiales	7870	2300362
Sample_YPB5.Cluster3185cbin.1	44.60	Clostridiales	24774	2486333
Sample_YPB5.mb.25cbin.1	42.20	Clostridiales	10025	2363910
Sample_YPB5.mb.30cbin.1	58.00	Clostridiales	60083	2680845
Sample_YPB5.mb.42	36.20	Clostridiales	127704	2462170
Sample_YPB5.Cluster5686	40.90	Lachnospiraceae	92472	2569951
Sample_YPB5.mb.19cbin.1	58.40	Clostridiales	62083	2245962
Sample_YPB5.mb.24cbin.1	57.50	Clostridiales	71304	2425405
Sample_YPB5.Cluster488cbin.1	59.90	Actinobacteria	26129	2060528
Sample_YPB5.Cluster5204cbin.1	50.50	Clostridiales	152426	2676717
Sample_YPB5.mb.65cbin.1	53.10	Clostridia	14711	1930857
Sample_YPB5.Cluster2663cbin.1	47.90	Bacteroidales	10183	2736133
Sample_YPB5.Cluster1628cbin.1	49.40	Bacteroidales	93218	2717292
Sample_YPB5.Cluster5889cbin.1	38.80	Clostridiales	85322	1992875
Sample_YPB5.mb.36cbin.1	41.30	Clostridiales	7794	1586585
Sample_YPB5.mb.75	49.40	Clostridiales	88311	1973611
Sample_YPB5.mb.56	41.10	Clostridiales	48437	3007906
Sample_YPB5.Cluster1199cbin.1	51.00	Firmicutes	42201	2370887
Sample_YPB5.Cluster4488cbin.1	46.70	Lachnospiraceae	70471	3355139
Sample_YPB5.mb.23	60.60	Clostridiales	10923	2309697
Sample_YPB5.mb.57	61.50	Clostridiales	107591	1913378
Sample_YPB5.Cluster400cbin.1	55.80	Proteobacteria	35544	2133694
Sample_YPB5.mb.5	54.60	Bacteroidetes	95365	2123103
Sample_YPB5.Cluster1691cbin.1	50.40	Bacteroidales	66734	2302546
Sample_YPB5.mb.54cbin.1	45.20	Clostridiales	102984	2641797
Sample_YPB5.mb.63cbin.1	48.50	Clostridiales	23948	2786452
Sample_YPB5.Cluster7289cbin.1	37.20	Clostridiales	141438	2823259
Sample_YPB5.mb.38	41.80	Lachnospiraceae	81164	2550441
Sample_YPB5.mb.46	40.90	Clostridiales	29523	2560430
Sample_YPB5.mb.33	49.80	Lachnospiraceae	20209	2826130
Sample_YPB5.mb.74	44.80	Clostridiales	176839	1767304
Sample_YPB5.mb.47	45.00	Bacteroidales	21650	2901317
Sample_YPB5.mb.78	41.50	Clostridiales	36431	2434466
Sample_YPB5.Cluster6949cbin.1	40.60	Bacteroidales	40027	3833053
Sample_YPB5.Cluster2441cbin.1	44.70	Bacteroidales	89485	3616756
Sample_YPB5.mb.9	38.50	Clostridiales	115216	3025331
Sample_YPB5.mb.40	46.60	Bacteroidales	148303	4101365
Sample_YPB6.Cluster2046cbin.1	45.10	Bacteria	127526	4565766

Sample_YPB6.mb.27	58.40	Clostridiales	7229	2615458
Sample_YPB6.mb.42cbin.1	53.40	Clostridiales	32133	2535940
Sample_YPB6.mb.23cbin.1	55.80	Bacteroidetes	7208	1701347
Sample_YPB6.mb.37cbin.1	59.80	Clostridiales	85431	3179040
Sample_YPB6.mb.30cbin.1	56.50	Clostridiales	6344	2243112
Sample_YPB6.mb.47cbin.1	50.40	Bacteroidales	61789	2700648
Sample_YPB6.mb.2	52.50	Firmicutes	50566	2406007
Sample_YPB6.Cluster3534	40.70	Clostridiales	302021	2103463
Sample_YPB6.mb.12	32.60	Bacteria	4600	2946295
Sample_YPB6.mb.13cbin.1	48.40	Proteobacteria	17025	2565476
Sample_YPB6.Cluster3334cbin.1	44.20	Bacteroidales	87463	3779030
Sample_YPB6.mb.24	46.60	Bacteroidales	98064	3297282
Sample_YPB6.Cluster1198cbin.1	49.10	Clostridiales	112463	6807916
Sample_YPB6.Cluster1316cbin.1	49.30	Clostridiales	281383	5935773
Sample_YPB6.Cluster2752cbin.1	41.70	Bacteroides	86480	5948014
Sample_YPB6.mb.51	42.70	Bacteroidales	80049	6604219
Sample_YPB7.Cluster12975	26.70	Bacteria	37434	1054950
Sample_YPB7.Cluster12994	26.00	Bacteria	72405	1198076
Sample_YPB7.Cluster1523cbin.1	54.00	Clostridia	7317	1448646
Sample_YPB7.mb.11	53.10	Clostridia	62100	1982230
Sample_YPB7.Cluster6653	45.40	Clostridiales	76846	1744650
Sample_YPB7.Cluster613	59.40	Clostridiales	65718	2025977
Sample_YPB7.mb.12	60.10	Clostridiales	17013	2170309
Sample_YPB7.Cluster244cbin.1	58.60	Clostridiales	12081	2435364
Sample_YPB7.Cluster3668cbin.1	56.90	Clostridiales	86985	2386872
Sample_YPB7.mb.33	61.60	Clostridiales	10157	2109584
Sample_YPB7.Cluster5070cbin.1	51.40	Clostridiales	163136	2158940
Sample_YPB7.mb.34	44.70	Clostridiales	20111	2060301
Sample_YPB7.mb.39	58.40	Clostridiales	13365	3138219
Sample_YPB7.mb.28	40.70	Lachnospiraceae	82104	2732026
Sample_YPB7.mb.69	38.90	Clostridiales	88803	1971022
Sample_YPB7.mb.7cbin.1	55.60	Clostridiales	14313	2526715
Sample_YPB7.mb.29	48.50	Proteobacteria	10041	2376523
Sample_YPB7.Cluster1361	58.30	Bacteroidetes	277662	3293629
Sample_YPB7.mb.23	43.40	Bacteroidales	44481	3093266
Sample_YPB7.mb.76	60.10	Clostridiales	47619	2224047
Sample_YPB7.Cluster10119cbin.1	45.30	Bacteroidales	14497	3607315
Sample_YPB7.mb.50cbin.1	43.10	Bacteria	53440	4060885
Sample_YPB7.mb.40cbin.1	46.60	Bacteroidales	42843	4374354
Sample_YPB7.mb.20	42.60	Bacteroidales	71115	5198816
Sample_YPB7.mb.67	45.10	Bacteroidales	151794	4646730
Sample_YPB7.Cluster8264cbin.1	50.60	Enterobacteriaceae	98226	4770210
Sample_YPB7.mb.65cbin.1	55.60	Enterobacteriaceae	118099	4514568
Sample_YPC1.Cluster3916	27.50	Bacteria	686357	1279636
Sample_YPC1.Cluster2324cbin.1	41.10	Clostridiales	84939	2359151
Sample_YPC1.mb.55	36.30	Clostridiales	30232	2518128
Sample_YPC1.Cluster838	46.50	Clostridiales	45469	1881001
Sample_YPC1.mb.52cbin.1	62.10	Clostridiales	260320	1945325
Sample_YPC1.Cluster22cbin.1	59.50	Clostridiales	135375	2166803
Sample_YPC1.Cluster992cbin.1	48.00	Clostridiales	155371	3093850
Sample_YPC1.mb.58	60.70	Clostridiales	43655	2311524
Sample_YPC1.mb.45	60.00	Actinobacteria	168792	2002454
Sample_YPC1.Cluster62cbin.1	60.40	Clostridiales	108879	2108434
Sample_YPC1.mb.63cbin.1	45.80	Firmicutes	48291	1658113
Sample_YPC1.Cluster479cbin.1	49.10	Clostridiales	72697	2926833
Sample_YPC1.Cluster214cbin.1	58.00	Clostridiales	73850	2494535
Sample_YPC1.Cluster1720	40.40	Clostridiales	242811	2243226
Sample_YPC1.mb.51	62.90	Bifidobacteriaceae	27528	2079084

Sample_YPC1.mb.19cbin.1	56.80	Bifidobacteriaceae	105305	2108585
Sample_YPC1.Cluster77	57.00	Proteobacteria	426676	2507820
Sample_YPC1.Cluster10cbin.1	59.60	Clostridiales	49124	2186401
Sample_YPC1.Cluster1799	42.90	Clostridiales	109570	2634769
Sample_YPC1.mb.33	38.50	Selenomonadales	80887	2122617
Sample_YPC1.mb.32	45.00	Clostridiales	24793	2611105
Sample_YPC1.Cluster660cbin.1	49.20	Proteobacteria	44904	2138818
Sample_YPC1.mb.49	61.80	Clostridiales	37576	3440932
Sample_YPC1.mb.13	45.30	Bacteroidales	185470	4130009
Sample_YPC1.mb.4cbin.1	46.20	Bacteroidales	111265	4251566
Sample_YPC2.Cluster12313	26.00	Bacteria	81319	1248554
Sample_YPC2.Cluster11916	29.50	Bacteria	45270	1417650
Sample_YPC2.Cluster2866cbin.1	53.00	Clostridia	86453	2038186
Sample_YPC2.Cluster11967cbin.1	33.30	Clostridiales	358172	1886999
Sample_YPC2.mb.2	46.40	Clostridiales	18567	1934026
Sample_YPC2.Cluster314	59.00	Clostridiales	33026	1989358
Sample_YPC2.mb.4	57.50	Clostridiales	12573	1792808
Sample_YPC2.mb.72	25.10	Bacteria	20138	1451294
Sample_YPC2.mb.58	58.20	Clostridiales	27935	2973239
Sample_YPC2.mb.28	61.20	Clostridiales	43322	2085675
Sample_YPC2.mb.45	60.00	Clostridiales	76421	2009346
Sample_YPC2.mb.42cbin.1	47.90	Clostridiales	98484	3050748
Sample_YPC2.mb.11	53.70	Clostridiales	306636	1706490
Sample_YPC2.Cluster623cbin.1	56.20	Clostridiales	14429	2536879
Sample_YPC2.mb.21	55.40	Bacteroidetes	36067	1897905
Sample_YPC2.mb.49cbin.1	56.00	Clostridiales	14547	2806726
Sample_YPC2.Cluster2726cbin.1	51.90	Clostridiales	116756	2258001
Sample_YPC2.Cluster9181	40.70	Clostridiales	214303	2108520
Sample_YPC2.mb.10	55.70	Bacteria	143244	2538119
Sample_YPC2.mb.66	62.20	Clostridiales	147648	1944001
Sample_YPC2.Cluster1309	54.60	Bacteroidales	72745	2294165
Sample_YPC2.Cluster752cbin.1	56.60	Bifidobacteriaceae	21249	2021463
Sample_YPC2.mb.39cbin.1	43.50	Selenomonadales	34868	2420804
Sample_YPC2.mb.77	58.90	Clostridiales	56877	2076792
Sample_YPC2.mb.25cbin.1	40.90	Clostridiales	12758	2520068
Sample_YPC2.mb.9cbin.1	56.90	Clostridiales	27618	1904606
Sample_YPC2.mb.31	57.20	Clostridiales	20054	3073850
Sample_YPC2.Cluster11303cbin.1	41.10	Lachnospiraceae	86166	3233635
Sample_YPC2.Cluster10628	38.40	Bacteroidales	64989	3103153
Sample_YPC2.mb.54cbin.1	43.20	Clostridiales	122596	2514264
Sample_YPC2.Cluster6818cbin.1	43.40	Bacteroidales	288695	3109358
Sample_YPC2.mb.59cbin.1	45.00	Clostridiales	56134	2899846
Sample_YPC2.Cluster5220cbin.1	48.50	Bacteroidales	173705	3571672
Sample_YPC2.Cluster7809cbin.1	44.80	Bacteroidales	73386	3869861
Sample_YPC2.Cluster5530	45.10	Bacteroidales	163477	4568647
Sample_YPC3.mb.11	58.90	Clostridia	6412	2501260
Sample_YPC3.Cluster277	59.90	Actinobacteria	202501	2321807
Sample_YPC3.mb.16	56.50	Clostridiales	53862	2767848
Sample_YPC3.Cluster3714cbin.1	48.10	Clostridiales	82620	2918619
Sample_YPC3.Cluster3877	47.00	Lactobacillales	129948	1863239
Sample_YPC3.mb.1	61.00	Clostridiales	15930	2815922
Sample_YPC3.Cluster4271cbin.1	44.60	Clostridiales	74262	2737248
Sample_YPC3.Cluster65	60.80	Clostridiales	103231	2097786
Sample_YPC3.Cluster333cbin.1	56.40	Clostridiales	15196	2423073
Sample_YPC3.Cluster3516	44.00	Selenomonadales	72759	2288678
Sample_YPC3.Cluster1980cbin.1	51.60	Clostridiales	59819	2195134
Sample_YPC3.Cluster5558	40.70	Clostridiales	439535	2155673
Sample_YPC3.Cluster1901cbin.1	56.80	Clostridiales	82490	2772015

Sample_YPC3.mb.47cbin.1	55.20	Selenomonadales	15798	1969889
Sample_YPC3.mb.3	34.60	Lactobacillus	325310	1809315
Sample_YPC3.mb.37cbin.1	40.10	Streptococcus	6813	1674145
Sample_YPC3.Cluster19	60.30	Bifidobacteriaceae	100296	2297096
Sample_YPC3.Cluster1253	56.20	Bifidobacteriaceae	391208	2154015
Sample_YPC3.mb.19	58.80	Bacteroidetes	168144	2876991
Sample_YPC3.mb.50	43.00	Clostridiales	162548	2781524
Sample_YPC3.mb.45cbin.1	59.00	Clostridiales	52045	2550536
Sample_YPC3.mb.23	44.20	Clostridiales	16021	3292325
Sample_YPC3.Cluster3672cbin.1	45.30	Bacteroidales	120879	4264281
Sample_YPC3.mb.13cbin.1	46.40	Bacteroidales	171847	4286962
Sample_YPC4.Cluster7056	43.60	Clostridiales	82261	2651486
Sample_YPC4.Cluster2418cbin.1	54.30	Actinobacteria	22395	1581634
Sample_YPC4.Cluster3903cbin.1	48.80	Clostridiales	7879	2499475
Sample_YPC4.Cluster699	60.50	Clostridiales	70942	1786204
Sample_YPC4.Cluster795cbin.1	56.30	Clostridiales	37595	2624808
Sample_YPC4.Cluster448cbin.1	61.00	Clostridiales	108084	2154662
Sample_YPC4.mb.52	62.00	Clostridiales	10401	1889584
Sample_YPC4.mb.71cbin.1	59.30	Clostridiales	6769	1782709
Sample_YPC4.mb.12cbin.1	48.70	Firmicutes	30417	1874691
Sample_YPC4.Cluster4622cbin.1	45.50	Bacteroidales	116576	3534520
Sample_YPC4.mb.27	51.50	Clostridiales	236332	2071565
Sample_YPC4.Cluster8779	38.10	Clostridiales	209589	1923899
Sample_YPC4.Cluster8162	40.50	Clostridiales	148195	2174043
Sample_YPC4.mb.65cbin.1	57.90	Clostridiales	44211	2610525
Sample_YPC4.mb.30	55.20	Bacteroidetes	64066	2640748
Sample_YPC4.mb.7cbin.1	61.40	Clostridiales	65994	2112432
Sample_YPC4.mb.15cbin.1	46.30	Clostridiales	59537	2492689
Sample_YPC4.Cluster8636cbin.1	37.40	Clostridiales	28608	2723328
Sample_YPC4.mb.36cbin.1	55.80	Clostridiales	50792	3301712
Sample_YPC4.mb.80	55.00	Bacteroidetes	117948	1972747
Sample_YPC4.mb.35	44.40	Bacteria	7392	3477884
Sample_YPC4.mb.47cbin.1	41.60	Lachnospiraceae	64609	2457930
Sample_YPC4.mb.74cbin.1	57.30	Clostridiales	30123	2483033
Sample_YPC4.Cluster4564cbin.1	42.60	Lachnospiraceae	35888	3776397
Sample_YPC4.mb.83cbin.1	46.40	Bacteroidales	144412	4122114
Sample_YPC4.mb.9cbin.1	45.00	Bacteroidales	165588	4721912
Sample_YPC5.Cluster10878	29.50	Bacteria	159896	1074323
Sample_YPC5.Cluster1749cbin.1	53.10	Clostridia	52007	2164198
Sample_YPC5.Cluster3557	49.30	Clostridiales	96829	1942892
Sample_YPC5.mb.27cbin.1	36.30	Clostridiales	222559	1930757
Sample_YPC5.Cluster10250cbin.1	33.30	Clostridiales	153862	3501426
Sample_YPC5.Cluster149cbin.1	61.20	Clostridiales	78480	1965311
Sample_YPC5.mb.56cbin.1	58.20	Clostridiales	45016	2976173
Sample_YPC5.Cluster480cbin.1	61.50	Clostridiales	69178	2064472
Sample_YPC5.Cluster11cbin.1	60.90	Clostridiales	103535	2065614
Sample_YPC5.mb.31	62.30	Clostridiales	28074	2042724
Sample_YPC5.mb.24	56.60	Clostridiales	12280	2121845
Sample_YPC5.Cluster5474cbin.1	45.30	Clostridiales	87610	2770553
Sample_YPC5.Cluster3768cbin.1	46.90	Proteobacteria	81954	1871824
Sample_YPC5.mb.38cbin.1	56.30	Clostridiales	18115	2541091
Sample_YPC5.Cluster3cbin.1	60.30	Bifidobacteriaceae	9089	2114457
Sample_YPC5.Cluster12cbin.1	63.90	Deltaproteobacteria	12053	2426257
Sample_YPC5.mb.3	54.30	Bacteroidetes	85088	2532875
Sample_YPC5.mb.53	38.80	Clostridiales	58643	1979436
Sample_YPC5.mb.36	41.30	Clostridiales	117661	2550634
Sample_YPC5.mb.85	36.30	Clostridiales	60371	2408355
Sample_YPC5.mb.51cbin.1	51.00	Firmicutes	42432	2342417

Sample_YPC5.mb.80	61.10	Clostridiales	12638	2020415
Sample_YPC5.mb.37cbin.1	37.40	Clostridiales	122729	2563590
Sample_YPC5.mb.25cbin.1	42.00	Lachnospiraceae	40231	2624430
Sample_YPC5.mb.69	46.20	Bacteroidetes	106743	2395009
Sample_YPC5.Cluster7905cbin.1	41.80	Lachnospiraceae	76747	2373919
Sample_YPC5.mb.79cbin.1	51.20	Clostridiales	136561	2254868
Sample_YPC5.mb.48	44.90	Bacteroidales	36172	3202798
Sample_YPC5.mb.84cbin.1	40.10	Clostridiales	223886	2381439
Sample_YPC5.Cluster5166cbin.1	43.00	Bacteria	48753	4254584
Sample_YPC5.mb.62	40.60	Bacteroidales	46724	3066155
Sample_YPC5.Cluster2626	45.70	Bacteroidales	30295	3766760
Sample_YPC5.mb.73cbin.1	44.70	Clostridiales	81008	3035214
Sample_YPC5.Cluster4418cbin.1	46.20	Bacteroidales	130978	4508038
Sample_YPC5.mb.70	45.00	Bacteroidales	123002	4700477
Sample_YPC6.Cluster7961	42.90	Clostridiales	91069	2069021
Sample_YPC6.Cluster8978cbin.1	38.10	Clostridiales	71529	2789171
Sample_YPC6.mb.26	57.20	Clostridiales	87429	1738453
Sample_YPC6.Cluster445	59.60	Clostridiales	37725	1825496
Sample_YPC6.Cluster10275	33.60	Bacteria	7179	1613996
Sample_YPC6.Cluster206	59.10	Clostridiales	11644	1999264
Sample_YPC6.mb.44cbin.1	57.70	Clostridiales	25386	1718212
Sample_YPC6.mb.66	59.30	Clostridiales	14059	1832604
Sample_YPC6.mb.19cbin.1	52.30	Clostridiales	9002	1915613
Sample_YPC6.mb.12	54.50	Clostridiales	8768	1576099
Sample_YPC6.mb.69	60.80	Clostridiales	34519	2269785
Sample_YPC6.mb.5	60.00	Actinobacteria	85629	2227602
Sample_YPC6.mb.21	44.20	Clostridiales	10740	2795682
Sample_YPC6.Cluster4134cbin.1	51.70	Clostridiales	184999	2154854
Sample_YPC6.mb.15	56.20	Clostridiales	16869	2538886
Sample_YPC6.mb.56	55.10	Bacteroidetes	23118	2671336
Sample_YPC6.mb.71	54.50	Selenomonadales	56801	2233808
Sample_YPC6.mb.67	41.00	Lachnospiraceae	64973	2663925
Sample_YPC6.Cluster781	58.40	Bacteroidetes	179582	3185175
Sample_YPC6.mb.57cbin.1	48.50	Proteobacteria	25069	2298887
Sample_YPC6.mb.68cbin.1	43.10	Bacteria	53907	4149223
Sample_YPC6.mb.41	46.90	Bacteroidales	43047	4236821
Sample_YPC6.mb.42cbin.1	41.90	Bacteroidales	66703	4908680
Sample_YPC6.mb.50	45.00	Bacteroidales	141658	4758912
Sample_YPC6.mb.9	42.70	Bacteroidales	130438	5312946
Sample_YPC6.Cluster2250cbin.1	50.70	Enterobacteriaceae	143177	4749113
Sample_YPC7.mb.15	43.50	Clostridiales	9479	2330615
Sample_YPC7.mb.10	43.80	Clostridiales	89988	2620679
Sample_YPC7.mb.23	61.10	Clostridiales	103004	2136736
Sample_YPC7.Cluster3550cbin.1	58.50	Clostridiales	94926	2331606
Sample_YPC7.mb.63	54.50	Actinobacteria	22442	1503331
Sample_YPC7.Cluster1606cbin.1	57.70	Clostridiales	56974	3014041
Sample_YPC7.Cluster3409cbin.1	48.50	Firmicutes	32188	1892095
Sample_YPC7.Cluster8892	38.20	Clostridiales	197505	1860140
Sample_YPC7.Cluster3787cbin.1	51.20	Clostridiales	133703	2147802
Sample_YPC7.mb.3	45.00	Clostridiales	217672	1717316
Sample_YPC7.mb.81	56.60	Clostridiales	32264	2513401
Sample_YPC7.Cluster7783cbin.1	37.50	Clostridiales	5955	2251054
Sample_YPC7.mb.34cbin.1	54.10	Bacteroidetes	94658	2355378
Sample_YPC7.mb.60cbin.1	55.30	Bacteroidetes	26629	2598569
Sample_YPC7.mb.42	46.20	Clostridiales	50979	2451606
Sample_YPC7.mb.78cbin.1	45.70	Bacteroidales	116777	3425626
Sample_YPC7.Cluster4349cbin.1	43.90	Bacteria	7599	3743109
Sample_YPC7.Cluster5553cbin.1	43.40	Lachnospiraceae	21489	2970734

Sample_YPC7.mb.6cbin.1	41.90	Lachnospiraceae	64470	2437213
Sample_YPC7.mb.18cbin.1	46.50	Bacteroidales	154265	4061493
Sample_YPC7.Cluster5024	45.00	Bacteroidales	121699	4590884
Sample_YPC7.mb.27	42.00	Bacteroides	103143	4550475
Sample_YPC7.mb.40	43.20	Bacteroidales	215785	5091450

Supplementary Table 4 Genomic information of 691 species-level genome bins (SGBs)

MAG ID	SGBs	Genome information			
	Rename_ID	Number of contigs	Genome size (Mbp)	N50 length (bp)	N90 length (bp)
Sample_LPB05.mb.50	SGB_486	281	2732861	13267	4774
Sample_LCB11.mb.63	SGB_554	44	2790653	133506	38796
Sample_SCB59.mb.4cbin.1	SGB_434	78	2361674	48187	15857
Sample_LPA03.mb.15	SGB_473	113	2487693	39651	8633
Sample_SPC08.mb.49	SGB_7	36	2209582	102907	28807
Sample_SPC21.mb.24cbin.1	SGB_204	47	2979775	101089	32985
Sample_LCC12.mb.103cbin.1	SGB_207	49	2028878	83732	17512
Sample_SPB27.mb.66cbin.1	SGB_406	44	2938035	87864	38796
Sample_YPC3.Cluster277	SGB_640	18	2321807	202501	59350
Sample_YPA1.mb.4cbin.1	SGB_62	153	1687998	14554	5251
Sample_LCC22.mb.58	SGB_40	83	2493468	42060	14740
Sample_LPA04.mb.76	SGB_492	47	1854336	64897	19465
Sample_SCB66.Cluster1136cbin.1	SGB_34	45	2870622	114877	29863
Sample_SPC06.mb.44cbin.1	SGB_84	6	1104318	632868	153903
Sample_SPA36.Cluster8708cbin.1	SGB_184	69	2783342	76405	20467
Sample_SPB09.mb.2	SGB_327	11	1966669	238572	73590
Sample_SCB59.mb.17cbin.1	SGB_633	14	1671109	216632	48516
Sample_SCA70.mb.36cbin.1	SGB_594	454	3705654	11474	3518
Sample_SPA45.mb.67	SGB_477	79	3015674	65141	21027
Sample_LPA03.mb.30	SGB_659	322	3000735	12969	4465
Sample_LCA11.mb.88	SGB_153	22	1727017	154033	31306
Sample_SPA34.Cluster14283cbin.1	SGB_106	38	1254767	82738	17818
Sample_SCA59.mb.86	SGB_553	8	2813988	517187	290324
Sample_LPB03.mb.50	SGB_561	60	2976634	79731	25119
Sample_SPC13.mb.88	SGB_506	312	1673310	6172	2869
Sample_SPB31.mb.86	SGB_508	68	1814198	43182	14467
Sample_SPB35.mb.107	SGB_244	51	1674337	47106	17842
Sample_SCC55.mb.41	SGB_159	99	3218793	68154	15490
Sample_LPC03.Cluster1576cbin.1	SGB_560	54	2383028	69771	21230
Sample_LPB03.mb.71cbin.1	SGB_108	23	1295902	154057	31253
Sample_SPC17.Cluster306cbin.1	SGB_430	151	2685819	26353	7891
Sample_YPC2.mb.31	SGB_315	230	3073850	20054	6367
Sample_SPB06.mb.44	SGB_396	77	2914362	75695	26073
Sample_LCC21.Cluster163	SGB_626	160	1539610	14043	4892
Sample_SPA16.mb.69	SGB_644	23	2425821	155051	43264
Sample_SPB17.mb.15cbin.1	SGB_645	111	2276157	32976	9800
Sample_SPB06.mb.139	SGB_213	142	2324094	23423	8048
Sample_SPB39.mb.64	SGB_298	179	1611216	13497	4165
Sample_SPC41.mb.11	SGB_208	111	2522795	30662	10846
Sample_LPA05.mb.17	SGB_49	58	1768719	41777	14797
Sample_SPB47.Cluster10470	SGB_134	91	2760674	51570	16188
Sample_LPC05.mb.16cbin.1	SGB_427	64	2994676	75146	20604
Sample_SPC35.mb.65cbin.1	SGB_577	61	2587439	66175	23458
Sample_YPC5.Cluster3768cbin.1	SGB_48	44	1871824	81954	18080
Sample_YPB7.mb.76	SGB_198	78	2224047	47619	15905
Sample_LPC03.Cluster8563	SGB_498	10	1697679	168841	118835
Sample_SPC41.mb.20cbin.1	SGB_483	116	2765391	52912	11370
Sample_SPB16.mb.58	SGB_363	20	2415767	187409	54510
Sample_SPA03.mb.7	SGB_336	10	2009618	493272	142434
Sample_LCC17.Cluster16367	SGB_107	13	1208669	175020	53700
Sample_SPC35.mb.88	SGB_654	19	1818800	112665	53977
Sample_SPA06.mb.70	SGB_395	265	2199822	11768	3711

Sample_SPC06.mb.8	SGB_152	55	2697495	75742	28182
Sample_SPA07.mb.7cbin.1	SGB_346	57	2961277	86483	24387
Sample_SPB35.mb.76cbin.1	SGB_349	29	2568252	159452	53541
Sample_LPC01.mb.111	SGB_393	38	2242505	103446	28935
Sample_SPB09.mb.27	SGB_410	31	2709700	208653	52181
Sample_YPA5.mb.28	SGB_624	96	3018383	47629	18868
Sample_SCC68.mb.50	SGB_11	78	4738247	105495	34543
Sample_SPA39.mb.135	SGB_12	199	2877403	22760	6853
Sample_LPB03.mb.93	SGB_628	42	2159130	103701	24198
Sample_LPB04.mb.12	SGB_348	29	2226051	131334	42373
Sample_LCC20.mb.63	SGB_161	225	1533973	8841	3392
Sample_SCB58.Cluster6891cbin.1	SGB_568	438	4907150	14750	5459
Sample_SCC66.Cluster3790	SGB_496	21	1834397	107272	56464
Sample_LCA10.Cluster63	SGB_646	21	2104755	138410	54211
Sample_LPC01.mb.90	SGB_356	53	2623352	81988	25991
Sample_SPA47.Cluster4099	SGB_677	5	1480694	543477	139136
Sample_SPA45.mb.26	SGB_676	34	2060642	73878	34904
Sample_LCC21.mb.107cbin.1	SGB_441	18	2886613	180933	82826
Sample_LPC03.mb.9cbin.1	SGB_285	67	2607915	74330	18881
Sample_SPA35.mb.59	SGB_209	46	2498279	95773	26610
Sample_SPC03.Cluster7720	SGB_171	24	2506866	127365	53914
Sample_SCA60.mb.13cbin.1	SGB_300	48	1994476	68944	24268
Sample_SPC12.mb.36	SGB_105	32	1248956	128570	21490
Sample_SCB66.Cluster5699	SGB_570	15	3301060	336434	134902
Sample_YPA3.Cluster4908	SGB_415	52	2758527	83804	28991
Sample_SCB67.mb.123cbin.1	SGB_170	85	2757152	66311	14669
Sample_SPB26.Cluster11740cbin.1	SGB_123	73	2183732	56560	16855
Sample_YPC2.mb.2	SGB_509	145	1934026	18567	6381
Sample_SPC13.mb.77	SGB_449	209	3196856	26149	7775
Sample_SPC07.Cluster7505cbin.1	SGB_414	315	2437325	10690	3709
Sample_SCC70.mb.92	SGB_575	120	2902092	35253	12228
Sample_SPB10.mb.53	SGB_603	43	3410303	158860	40882
Sample_SPC08.mb.66	SGB_86	46	1149135	33617	13461
Sample_LCC20.Cluster427cbin.1	SGB_269	100	2379266	39455	12415
Sample_SPC21.mb.73cbin.1	SGB_525	42	2281032	186701	24523
Sample_SPB25.mb.83cbin.1	SGB_335	43	2608080	123245	26099
Sample_SCC68.mb.72cbin.1	SGB_110	60	1667013	51997	12053
Sample_SPA37.mb.69	SGB_400	145	3453619	49875	10889
Sample_SPC45.mb.65	SGB_288	160	2134391	21051	6853
Sample_SPB20.mb.94cbin.1	SGB_179	41	2718168	111087	37269
Sample_SPC06.Cluster5280cbin.1	SGB_387	90	2584680	41390	13324
Sample_LCA12.Cluster4404cbin.1	SGB_572	41	2475525	88177	29944
Sample_SCB55.Cluster6784cbin.1	SGB_504	49	1754948	44705	17996
Sample_SCC64.mb.43cbin.1	SGB_201	88	2583381	51322	14604
Sample_SCC60.mb.28	SGB_598	172	2825226	21419	8656
Sample_LPC01.mb.41	SGB_471	72	2600389	67469	20790
Sample_SPA13.Cluster428cbin.1	SGB_199	47	2721915	115233	29591
Sample_LCB11.Cluster802	SGB_648	25	2114414	179302	60643
Sample_SPB07.Cluster4507cbin.1	SGB_597	36	3770281	137138	63931
Sample_SPB38.Cluster5633	SGB_619	63	2792758	62414	27697
Sample_SPB07.mb.66	SGB_266	177	2688861	22151	7358
Sample_SPA21.mb.25	SGB_98	19	1326539	148895	31192
Sample_YPA2.Cluster10058	SGB_328	18	2100509	209030	42051
Sample_SPC35.mb.71	SGB_5	22	2483976	201111	54446
Sample_SPB25.mb.51	SGB_485	350	2181301	7219	3345
Sample_YPB5.mb.56	SGB_425	95	3007906	48437	16341
Sample_SPB25.Cluster557	SGB_680	21	1641065	87341	33809
Sample_LPB08.Cluster4247	SGB_462	61	3021040	87170	26592

Sample_SCB69.mb.12	SGB_630	87	1775969	41585	8521
Sample_SPA22.mb.53	SGB_162	245	2529954	13327	4990
Sample_SPA03.Cluster35	SGB_36	103	2398288	38533	10598
Sample_SCB66.mb.84	SGB_329	22	2006701	196270	54345
Sample_LCC13.mb.29	SGB_245	87	1897324	39290	10195
Sample_SPC15.mb.17	SGB_57	113	2043372	29793	8307
Sample_SCA65.Cluster3577cbin.1	SGB_51	36	1775109	64523	26840
Sample_SCB70.mb.14	SGB_422	53	2963905	108139	35094
Sample_YPB6.mb.51	SGB_614	139	6604219	80049	23135
Sample_SCC55.mb.2	SGB_523	7	1929681	371778	138707
Sample_LCA20.mb.34cbin.1	SGB_283	41	2160390	85333	30346
Sample_SCC55.Cluster680cbin.1	SGB_24	42	5219743	277787	56577
Sample_SPA41.Cluster9689	SGB_325	31	2388848	138982	33668
Sample_SPA17.mb.8cbin.1	SGB_129	19	1918422	224568	62010
Sample_SCA55.Cluster10485	SGB_117	107	1677449	27657	8603
Sample_LCB17.Cluster10309cbin.1	SGB_301	17	1609210	151377	54744
Sample_LPC08.mb.10	SGB_465	75	3187118	68255	24417
Sample_LCA15.mb.46	SGB_392	32	2394224	118508	33352
Sample_SPA06.Cluster4973cbin.1	SGB_386	144	5499852	78062	17654
Sample_YPB3.Cluster1cbin.1	SGB_691	19	2060869	218894	68351
Sample_SPB03.mb.2cbin.1	SGB_601	51	3431634	117189	32912
Sample_SPB09.mb.88cbin.1	SGB_557	108	3220703	42701	17719
Sample_YPB6.Cluster1316cbin.1	SGB_421	42	5935773	281383	84286
Sample_SCC55.Cluster435	SGB_69	21	2486379	335488	49048
Sample_YPC3.Cluster4271cbin.1	SGB_365	56	2737248	74262	25370
Sample_SPC09.Cluster1936cbin.1	SGB_299	76	2379470	52694	14542
Sample_LPA02.Cluster184cbin.1	SGB_221	79	2184193	40366	14026
Sample_SCC60.Cluster13861	SGB_77	61	1619639	42112	11771
Sample_SPB34.Cluster11702	SGB_56	54	2653438	82996	23280
Sample_SPC47.mb.63	SGB_616	99	3562795	63812	20538
Sample_SPA38.mb.134	SGB_311	20	1855382	174728	47800
Sample_SPC28.mb.72cbin.1	SGB_202	49	2688118	102272	25922
Sample_SPA41.Cluster9423	SGB_118	23	1819711	126855	50317
Sample_SPC06.mb.21cbin.1	SGB_381	325	2991236	11969	4608
Sample_SPC22.Cluster16607	SGB_352	19	1313344	135840	32019
Sample_SPB25.mb.68	SGB_629	34	3184157	173393	42244
Sample_SCB59.Cluster11926cbin.1	SGB_337	20	2003082	151700	51692
Sample_SPC21.mb.77	SGB_275	38	1947810	78974	28577
Sample_SCB58.mb.15	SGB_293	40	1768652	73451	24477
Sample_SCB61.Cluster8139	SGB_467	15	2268691	271842	89005
Sample_SCA70.Cluster4626cbin.1	SGB_556	209	2801022	20047	6582
Sample_SPC09.mb.33	SGB_438	57	2648893	77829	24702
Sample_LPA02.mb.63	SGB_96	6	1150263	257512	135336
Sample_YPA4.mb.15cbin.1	SGB_600	40	4171730	161622	50008
Sample_LPB04.Cluster2445	SGB_1	34	3045963	143811	43440
Sample_SPC42.mb.29cbin.1	SGB_353	36	2262379	106598	26022
Sample_LCC19.Cluster5834cbin.1	SGB_376	51	2617018	89126	27141
Sample_SPC27.Cluster5420cbin.1	SGB_330	22	1900378	117224	52675
Sample_LCC17.mb.119	SGB_4	199	2306101	15778	5444
Sample_SPB45.mb.75cbin.1	SGB_265	151	3157530	41648	9271
Sample_YPB4.Cluster6185	SGB_87	12	1318263	309168	173193
Sample_SPB45.Cluster2056	SGB_491	23	2582299	201270	52000
Sample_SCA61.mb.16	SGB_420	175	3076604	28321	8099
Sample_SPC41.mb.40	SGB_687	20	1783192	126920	61304
Sample_SCB58.mb.43	SGB_100	33	1163235	72446	23539
Sample_SPA11.Cluster231cbin.1	SGB_257	64	2182278	51368	17360
Sample_YPB6.mb.12	SGB_143	705	2946295	4600	2374
Sample_SPB35.mb.85cbin.1	SGB_92	19	1188476	141095	39875

Sample_SPA07.Cluster10244	SGB_113	82	1595473	27677	9216
Sample_SPA34.mb.104	SGB_271	67	2074494	40905	18337
Sample_SPA06.Cluster5434	SGB_533	34	4196315	179583	58340
Sample_SCA70.mb.70cbin.1	SGB_464	88	3254323	53735	21966
Sample_SPA30.mb.27	SGB_345	64	2582096	63082	23533
Sample_YPB1.mb.17cbin.1	SGB_588	108	3643269	52834	17389
Sample_LCB13.mb.71	SGB_264	88	2002002	30489	11030
Sample_YPA1.mb.7	SGB_200	45	2358071	79325	25077
Sample_LPA03.Cluster907cbin.1	SGB_479	61	4064562	124555	34436
Sample_SPA11.Cluster12075cbin.1	SGB_139	208	2831782	21403	6215
Sample_SCB64.Cluster3178cbin.1	SGB_63	95	2525576	41189	12814
Sample_SPA26.Cluster2008cbin.1	SGB_585	338	2313632	8368	3358
Sample_YPB2.mb.83	SGB_440	42	2668258	87814	36566
Sample_SCA70.mb.116	SGB_206	87	1794964	31905	10088
Sample_SCA69.mb.54	SGB_253	41	2071164	86079	30859
Sample_SCB65.mb.4cbin.1	SGB_534	658	5141288	10224	3787
Sample_LCA10.Cluster26cbin.1	SGB_225	281	1986601	8375	3597
Sample_SPA41.Cluster6557cbin.1	SGB_499	18	1688546	248209	62314
Sample_YPB1.mb.77	SGB_399	306	4635425	34158	5348
Sample_SPC32.Cluster5660cbin.1	SGB_324	21	2181677	162791	108424
Sample_SPA06.mb.136cbin.1	SGB_578	89	3237762	51212	18847
Sample_LPA04.mb.37	SGB_47	93	1635647	28338	9084
Sample_YPC4.mb.7cbin.1	SGB_251	51	2112432	65994	23241
Sample_LCA11.Cluster13155	SGB_147	80	3612470	81472	23547
Sample_SPA06.Cluster6148	SGB_442	24	2361202	177579	51941
Sample_LPA01.Cluster3463cbin.1	SGB_182	49	2193194	77571	22736
Sample_LCA20.Cluster6765	SGB_333	22	1918207	135599	54595
Sample_SPA26.mb.15	SGB_59	28	2073637	120572	36499
Sample_LPB08.Cluster4577cbin.1	SGB_384	82	2757817	61570	16776
Sample_YPA7.Cluster3867cbin.1	SGB_338	16	2020786	156519	100145
Sample_SPC15.mb.80cbin.1	SGB_576	86	2877920	43793	17135
Sample_SPB35.mb.66cbin.1	SGB_18	293	2912267	13046	5004
Sample_SPC34.mb.30	SGB_67	110	2118561	28010	10525
Sample_SPA37.Cluster6932cbin.1	SGB_382	53	5500404	232233	72197
Sample_SPA39.Cluster366	SGB_39	31	2454915	97883	36075
Sample_YPB5.mb.9	SGB_444	65	3025331	115216	37294
Sample_LCA20.mb.55	SGB_25	44	5042511	229737	52724
Sample_LCC19.Cluster32	SGB_27	74	5556779	146518	35963
Sample_SPB22.Cluster9389	SGB_76	34	1486387	69807	19263
Sample_LPA04.Cluster8097cbin.1	SGB_388	178	3757709	50020	8709
Sample_SPC32.mb.23	SGB_326	32	2079869	104957	28216
Sample_LPC04.mb.99	SGB_490	224	2142352	12753	4746
Sample_SPA16.mb.39cbin.1	SGB_374	51	2954212	81881	37331
Sample_SPA22.mb.36	SGB_163	62	2077862	54920	17413
Sample_LCC14.mb.37	SGB_229	53	2610633	75898	23968
Sample_SPA39.mb.96	SGB_621	82	3091925	63449	23392
Sample_SPA13.mb.29	SGB_672	58	3908526	149370	38479
Sample_SPA38.Cluster3464	SGB_552	23	2868338	203576	59341
Sample_SPA08.Cluster6153	SGB_550	10	2374613	349055	95938
Sample_LCC11.mb.52	SGB_252	48	1860481	74942	19870
Sample_LPB03.mb.74	SGB_94	41	1393834	52101	19466
Sample_LPC05.mb.69cbin.1	SGB_54	54	2032967	60352	17434
Sample_SPC12.mb.53	SGB_537	70	3051418	66004	24377
Sample_SPC37.mb.39cbin.1	SGB_513	347	2319839	8893	2970
Sample_SPA07.mb.62	SGB_145	130	2353384	27111	8981
Sample_SPC08.mb.56	SGB_168	305	1616308	6184	2709
Sample_LCC13.mb.20cbin.1	SGB_391	100	2852337	51169	19420
Sample_LCB11.mb.45	SGB_238	50	1788916	59772	20089

Sample_LCA17.mb.24	SGB_618	53	2957054	90369	36092
Sample_LCA12.mb.95	SGB_210	35	2285778	100173	38877
Sample_SPA35.mb.102cbin.1	SGB_433	57	2722622	80266	23357
Sample_LPB05.mb.47	SGB_661	71	2140419	43244	17247
Sample_SPB32.Cluster4022cbin.1	SGB_23	306	1500434	5912	2633
Sample_LPC04.mb.101cbin.1	SGB_15	133	2204451	25324	7638
Sample_SPC30.mb.43cbin.1	SGB_272	89	2383740	44227	14298
Sample_YPC3.mb.23	SGB_448	305	3292325	16021	4621
Sample_SCB60.mb.57	SGB_242	76	2065863	36327	14593
Sample_SPB35.mb.29	SGB_99	72	1182743	37057	6125
Sample_SCA67.mb.90	SGB_284	57	2111722	62630	20986
Sample_SCA58.Cluster4693	SGB_631	8	1719666	328091	106330
Sample_SPB12.mb.23	SGB_409	25	2786119	204061	53645
Sample_LCA10.Cluster1830cbin.1	SGB_142	240	3703031	24887	7155
Sample_SPA22.Cluster19293cbin.1	SGB_524	28	1843180	142830	33584
Sample_LPC04.mb.32	SGB_318	37	2762812	105606	41134
Sample_SPA28.Cluster3006cbin.1	SGB_574	29	2865127	141255	44200
Sample_YPB7.Cluster3668cbin.1	SGB_258	55	2386872	86985	27438
Sample_YPC3.mb.16	SGB_268	88	2767848	53862	15986
Sample_SPA13.mb.12	SGB_169	60	2237955	106436	19236
Sample_SPB42.Cluster8875cbin.1	SGB_128	137	2769590	33237	9451
Sample_YPC1.Cluster10cbin.1	SGB_197	65	2186401	49124	18382
Sample_SPA37.mb.113	SGB_88	18	1234695	135499	44350
Sample_SPA41.Cluster7247	SGB_608	35	4972160	273843	76580
Sample_YPB2.Cluster9399cbin.1	SGB_437	28	2606803	138548	59625
Sample_SPB06.Cluster6571cbin.1	SGB_567	103	5364619	83932	24582
Sample_SPA25.mb.46cbin.1	SGB_519	203	3242802	25397	7026
Sample_YPB2.mb.32cbin.1	SGB_383	45	3093258	129659	33830
Sample_SCA70.Cluster4600cbin.1	SGB_205	52	2892041	105697	26995
Sample_SPB16.mb.47	SGB_156	37	1456494	58609	20479
Sample_LPC02.mb.23	SGB_234	59	1967808	47144	16790
Sample_SPC26.Cluster3107	SGB_75	35	2219270	84740	27686
Sample_SPC39.Cluster1686cbin.1	SGB_546	51	2230974	68993	22494
Sample_YPC3.mb.3	SGB_121	11	1809315	325310	85856
Sample_SPA15.mb.71	SGB_322	60	2092438	67126	17939
Sample_SPA11.mb.62	SGB_115	113	1949820	29941	8110
Sample_LPC02.mb.80cbin.1	SGB_296	112	2403694	33559	10038
Sample_SCC61.mb.41cbin.1	SGB_599	213	4249544	35684	8681
Sample_SPB39.mb.102	SGB_558	97	2058737	31225	10827
Sample_SPB39.mb.15cbin.1	SGB_371	213	2672006	24098	4771
Sample_SPC22.mb.86cbin.1	SGB_130	36	2662997	155045	44596
Sample_SPA21.mb.76	SGB_635	122	2677538	36625	9996
Sample_LCA21.mb.13	SGB_419	120	3157133	43020	14943
Sample_SPA38.mb.91	SGB_573	79	4265340	146273	24363
Sample_SPB08.mb.5	SGB_482	125	2960042	39674	10652
Sample_SPA35.mb.49	SGB_270	96	2448756	47967	12794
Sample_SCB65.mb.65cbin.1	SGB_606	59	5922940	174079	65975
Sample_SPA31.mb.7cbin.1	SGB_141	151	3090226	39578	9507
Sample_SPC17.Cluster2305	SGB_135	116	4407976	60957	17342
Sample_LCA12.mb.49	SGB_510	87	1762286	33744	9381
Sample_LCC17.mb.120	SGB_312	49	2455685	81530	25218
Sample_YPC6.Cluster7961	SGB_344	34	2069021	91069	26173
Sample_YPC4.Cluster4622cbin.1	SGB_580	47	3534520	116576	48825
Sample_SPB45.mb.80cbin.1	SGB_247	79	1837146	38829	10108
Sample_YPB7.mb.65cbin.1	SGB_33	54	4514568	118099	39214
Sample_SPB25.mb.26	SGB_218	39	3060127	139133	34461
Sample_LPB02.mb.26cbin.1	SGB_460	131	2563012	33031	10570

Sample_LCB23.Cluster597cbin.1	SGB_255	113	2921785	57418	9971
Sample_SPA12.mb.95	SGB_306	237	2069009	11583	4303
Sample_SPA21.Cluster125cbin.1	SGB_316	63	3845425	105649	31899
Sample_LPC03.Cluster5010cbin.1	SGB_541	14	2578641	268193	123403
Sample_SCA65.mb.10	SGB_665	86	2051640	42222	13150
Sample_YPA6.Cluster5179cbin.1	SGB_515	59	2207385	57740	17383
Sample_LCB19.mb.1	SGB_611	150	4034019	49334	12529
Sample_SPC22.Cluster184	SGB_220	45	2383507	76000	24338
Sample_LCB25.mb.2cbin.1	SGB_70	68	2589349	53025	19603
Sample_LCB14.Cluster4494	SGB_158	25	2831525	187541	62168
Sample_SPC27.Cluster6911	SGB_373	31	2793540	132554	50134
Sample_SPA15.mb.81	SGB_216	226	2238760	14279	4939
Sample_SPB42.Cluster17197cbin.1	SGB_684	134	4341667	60858	17450
Sample_SPA15.Cluster10540cbin.1	SGB_609	102	4991493	107193	25044
Sample_SCC64.mb.47cbin.1	SGB_368	126	3626557	54946	14049
Sample_LCB22.mb.50cbin.1	SGB_237	44	2195657	101268	20791
Sample_YPC1.mb.49	SGB_232	145	3440932	37576	12265
Sample_SPA26.mb.78	SGB_313	27	1861059	122667	40081
Sample_LPA01.Cluster90cbin.1	SGB_222	39	2984495	139335	52207
Sample_SPB22.mb.88	SGB_102	19	1265673	134723	40689
Sample_SPB27.Cluster2220	SGB_380	48	7025057	193235	96256
Sample_SPC12.mb.56cbin.1	SGB_686	116	1875474	25568	8435
Sample_LCC09.Cluster6301cbin.1	SGB_565	68	4481218	100817	39265
Sample_SPA32.mb.13	SGB_361	80	2580181	45164	16515
Sample_SPA08.Cluster8154	SGB_634	8	1843477	296366	189265
Sample_SPA36.Cluster12603	SGB_111	11	1685675	306479	61861
Sample_LCB10.mb.44cbin.1	SGB_125	67	2078042	44896	14033
Sample_LCC12.mb.99	SGB_263	108	2533175	36580	12500
Sample_YPB1.Cluster198cbin.1	SGB_189	228	2266106	14032	4494
Sample_SCC70.mb.102	SGB_398	169	2677249	24346	7985
Sample_LPB05.Cluster4298cbin.1	SGB_2	28	2941868	127223	60826
Sample_SPC15.mb.26cbin.1	SGB_503	73	1793647	39517	11524
Sample_LPA04.mb.52cbin.1	SGB_497	16	1872101	248839	69702
Sample_SPB03.mb.4	SGB_439	99	2972480	66445	13138
Sample_LPB08.Cluster5246cbin.1	SGB_347	62	2889446	77340	21517
Sample_SPB26.mb.88	SGB_17	568	4289306	9660	3716
Sample_SCC69.mb.60	SGB_453	70	3266613	84225	26437
Sample_SCC69.Cluster1876	SGB_74	32	2170438	109439	35082
Sample_SPB36.mb.9cbin.1	SGB_321	38	2111525	95811	24129
Sample_SPB13.Cluster946	SGB_43	33	2394022	101610	35009
Sample_SPB30.mb.72	SGB_116	33	1955979	85236	40072
Sample_SPB41.Cluster11656	SGB_652	45	1579723	45236	18184
Sample_LPC04.mb.111	SGB_489	61	2214337	61468	18121
Sample_SPC04.Cluster5731cbin.1	SGB_186	343	2151617	7682	2892
Sample_SCA68.Cluster215cbin.1	SGB_294	64	2201846	79211	13854
Sample_LCA22.mb.29	SGB_240	37	2088623	75294	28329
Sample_LPA08.mb.37	SGB_146	82	2678454	48443	16385
Sample_SCC60.Cluster12028	SGB_411	23	2472923	127423	63888
Sample_SPB47.mb.14	SGB_38	55	2471398	82520	21727
Sample_SPC13.Cluster8354	SGB_468	70	2186718	61716	14226
Sample_SPA30.mb.100	SGB_277	21	2017670	177617	39299
Sample_SPC12.Cluster2893cbin.1	SGB_538	23	2121579	184726	43015
Sample_SPA25.mb.23cbin.1	SGB_401	169	6704437	72048	20885
Sample_LCC17.Cluster16446	SGB_80	21	1048374	58879	25540
Sample_SPA17.Cluster6508	SGB_55	72	3386714	79675	26442
Sample_LCA13.mb.86	SGB_167	45	2312046	79376	30459
Sample_SPA25.Cluster5997	SGB_632	22	1940661	143482	47476
Sample_SPB37.mb.4cbin.1	SGB_532	50	4160518	150216	51090

Sample_SPB10.mb.73	SGB_226	208	1855234	12839	4015
Sample_SPB45.mb.39cbin.1	SGB_280	221	2461925	15999	4968
Sample_LCA25.mb.26	SGB_369	78	3565096	90930	21631
Sample_LPC04.mb.91cbin.1	SGB_212	43	2522894	75485	37195
Sample_LCB20.Cluster9862	SGB_104	11	1134637	223391	67128
Sample_YPA1.Cluster642cbin.1	SGB_602	51	5711805	204710	49540
Sample_SCA64.Cluster336	SGB_3	35	3119347	185474	42521
Sample_LPB03.mb.40	SGB_455	300	2585384	11498	4095
Sample_SPA31.Cluster110	SGB_20	104	2423573	39552	9560
Sample_SPB12.mb.52	SGB_308	28	2201312	97053	42533
Sample_LPC08.mb.5	SGB_101	20	1463455	140182	52194
Sample_LCA12.mb.112	SGB_539	20	2358159	155090	61548
Sample_YPC7.mb.27	SGB_615	91	4550475	103143	24992
Sample_SPA08.mb.10	SGB_109	14	1400332	229447	55695
Sample_SPB26.mb.37	SGB_13	97	4172617	72127	20832
Sample_SCB68.Cluster8783cbin.1	SGB_124	39	1872763	100414	21691
Sample_YPC2.mb.66	SGB_278	25	1944001	147648	44788
Sample_SPA39.Cluster2121	SGB_61	37	2289189	111045	26531
Sample_SCB67.Cluster13396cbin.1	SGB_581	97	2819522	43724	15458
Sample_YPB1.Cluster1122cbin.1	SGB_370	45	3015152	94440	31370
Sample_SPC13.mb.49	SGB_223	141	2731672	33801	8439
Sample_SCB70.mb.112	SGB_423	89	3438924	68208	20395
Sample_LPC01.mb.119	SGB_359	102	3033591	43180	13717
Sample_SPC15.mb.22	SGB_248	52	1936476	53876	22829
Sample_SPA07.Cluster99cbin.1	SGB_276	26	1989112	116865	50545
Sample_LPB05.mb.94	SGB_662	230	2837737	19589	5630
Sample_LCC12.mb.117	SGB_291	119	1769813	25315	6873
Sample_LPB05.Cluster12286cbin.1	SGB_354	189	3099658	30077	6550
Sample_YPA3.mb.19cbin.1	SGB_302	85	2621196	50928	14055
Sample_SPB47.Cluster1233cbin.1	SGB_181	86	2338461	46922	14207
Sample_SPA42.mb.53cbin.1	SGB_304	78	2203722	53731	16189
Sample_SPB25.mb.62cbin.1	SGB_131	355	3420545	15479	4401
Sample_SPB25.mb.95cbin.1	SGB_31	162	4758027	48700	13833
Sample_SPA47.mb.6	SGB_679	53	4620240	122797	47064
Sample_SPC42.mb.101cbin.1	SGB_502	34	1896417	111248	23087
Sample_SPC30.Cluster6113cbin.1	SGB_379	100	6963900	129291	39495
Sample_LPB03.Cluster8614cbin.1	SGB_459	329	1801825	6532	2731
Sample_SPA21.mb.70cbin.1	SGB_236	117	1820827	23531	7481
Sample_LCA23.mb.4cbin.1	SGB_37	85	2609997	47402	14046
Sample_SPB45.Cluster12822	SGB_85	21	969526	78572	19960
Sample_LPB05.Cluster3708cbin.1	SGB_307	24	2327367	205757	49338
Sample_YPC6.mb.9	SGB_607	70	5312946	130438	51218
Sample_LPB04.mb.33	SGB_545	152	3082185	30823	9857
Sample_LPC05.Cluster9183	SGB_397	26	2276256	171888	51447
Sample_SPA17.mb.79	SGB_230	205	1812548	11367	4273
Sample_SPB26.mb.16	SGB_555	73	2119015	47638	15620
Sample_SPC42.Cluster10367cbin.1	SGB_358	50	3057102	131663	44565
Sample_YPA7.mb.67	SGB_367	163	4150807	50911	10558
Sample_SPA10.mb.48	SGB_389	147	2523366	25737	8133
Sample_SCA55.mb.44	SGB_651	15	1776570	169592	52208
Sample_SPB25.mb.74	SGB_404	118	1845048	28277	7270
Sample_SPC41.mb.28	SGB_166	122	2892358	38083	11044
Sample_SPB27.Cluster1327	SGB_341	20	2332374	136896	86553
Sample_SPA13.mb.24	SGB_447	107	3153857	48677	15732
Sample_SPB09.Cluster7564cbin.1	SGB_378	95	3484331	66462	21532
Sample_SPB15.mb.67	SGB_474	222	1749994	10204	3929
Sample_SCA58.mb.77	SGB_625	56	1818609	47400	21284

Sample_SPB12.Cluster197	SGB_35	62	2338404	68391	16844
Sample_SPB45.Cluster4014cbin.1	SGB_530	66	3583945	75612	28132
Sample_LPC05.mb.95cbin.1	SGB_287	40	2352355	95294	31038
Sample_LCA16.Cluster1219	SGB_16	363	4205078	16887	5773
Sample_SPB20.mb.3	SGB_78	25	1663990	165171	51390
Sample_LCA10.Cluster54	SGB_641	17	2307108	268425	60067
Sample_SPB16.Cluster975cbin.1	SGB_45	113	2015373	26558	7720
Sample_SPB42.mb.19cbin.1	SGB_28	357	5117098	24029	5905
Sample_SPC17.mb.1cbin.1	SGB_405	35	2653461	161084	45220
Sample_SPC41.mb.23	SGB_511	84	1310247	25408	7385
Sample_SPB25.mb.77	SGB_133	82	3087238	102139	14137
Sample_SPB34.Cluster2430cbin.1	SGB_402	406	7078483	28913	8883
Sample_LCC20.mb.21	SGB_457	26	2620320	160795	46973
Sample_SPC42.mb.7cbin.1	SGB_593	144	2438682	24341	9505
Sample_LCA13.mb.9cbin.1	SGB_564	67	4256297	113950	28522
Sample_SPA39.mb.30cbin.1	SGB_675	25	2388844	140483	63645
Sample_LCC14.mb.83	SGB_122	9	1861909	509809	104858
Sample_SPC41.mb.69	SGB_476	135	1971882	26498	5838
Sample_SPB32.mb.29	SGB_470	70	2623114	65602	23121
Sample_LPB02.Cluster10625cbin.1	SGB_180	44	2173839	159676	28527
Sample_SPC25.mb.23cbin.1	SGB_591	141	2583734	26701	8841
Sample_SCB70.mb.16	SGB_350	272	2080064	9880	3628
Sample_SPB26.Cluster96	SGB_518	59	2662176	78434	19689
Sample_SPB45.mb.76cbin.1	SGB_286	34	2194883	190010	30132
Sample_SPC41.mb.95cbin.1	SGB_259	164	2742663	23992	8173
Sample_LCC16.Cluster2870cbin.1	SGB_446	102	3767556	58941	18063
Sample_LPC04.mb.90	SGB_481	79	2640543	58839	18330
Sample_SPC35.mb.109cbin.1	SGB_93	16	1295659	115425	44237
Sample_SPB22.mb.74	SGB_215	37	1961084	73382	28237
Sample_SPB30.mb.27cbin.1	SGB_517	120	2427290	28417	10871
Sample_YPA4.mb.1	SGB_689	207	1813807	12597	3864
Sample_YPB5.Cluster2663cbin.1	SGB_595	352	2736133	10183	3519
Sample_LPB04.Cluster6113	SGB_176	23	2311880	144604	52966
Sample_SPC17.mb.74cbin.1	SGB_194	67	2462355	64090	21356
Sample_SPA06.Cluster317cbin.1	SGB_246	79	2129530	40070	15529
Sample_SPA35.mb.47	SGB_512	22	1701670	129867	45752
Sample_SPA47.mb.102	SGB_678	101	1825175	31087	7774
Sample_LCC14.mb.50cbin.1	SGB_362	598	3972058	8806	3151
Sample_LPC04.mb.20	SGB_663	227	2431694	15163	4646
Sample_SPC45.Cluster13128cbin.1	SGB_173	105	2107173	30498	9682
Sample_SPC11.mb.95cbin.1	SGB_542	219	2270058	15073	4886
Sample_LPA04.mb.23	SGB_660	109	2206631	30586	11418
Sample_YPC6.mb.67	SGB_390	75	2663925	64973	19927
Sample_SPB28.mb.65	SGB_22	67	1875575	49308	14620
Sample_LPB08.mb.16	SGB_372	52	2791785	132194	27620
Sample_YPB1.mb.59cbin.1	SGB_196	116	2834807	55483	12934
Sample_SPA16.mb.2cbin.1	SGB_136	86	2674383	46670	15427
Sample_SCB67.Cluster1428	SGB_668	22	2511719	141760	73857
Sample_LCA12.mb.79cbin.1	SGB_589	42	3592439	141886	48512
Sample_LCA11.mb.105cbin.1	SGB_617	72	4499374	117272	34399
Sample_SPA17.mb.78cbin.1	SGB_418	41	2633730	95376	35760
Sample_SPA22.mb.91	SGB_155	49	1359696	51840	9728
Sample_LCC22.Cluster6998	SGB_331	9	1750722	479723	56377
Sample_SPC17.mb.37	SGB_469	30	1952141	93867	31511
Sample_LCC14.Cluster994	SGB_127	61	1800233	45338	14291
Sample_SPC16.Cluster3365	SGB_72	31	2419140	113579	37967

Sample_YPB2.Cluster947	SGB_690	33	5173266	278233	62766
Sample_SPB10.mb.77	SGB_463	61	3003496	115718	35181
Sample_SPB22.mb.47	SGB_174	198	2288233	16191	5519
Sample_LPC05.Cluster4101cbin.1	SGB_613	130	3318434	41200	12432
Sample_SPC15.Cluster5521cbin.1	SGB_571	23	2247102	141132	48313
Sample_SPC39.Cluster5880cbin.1	SGB_66	91	2000862	32107	9778
Sample_LCA14.Cluster1346	SGB_505	16	2420397	315366	73307
Sample_SPC08.mb.54	SGB_149	59	1818150	55047	13964
Sample_SPC22.mb.12	SGB_219	64	2860698	80712	21029
Sample_LCA16.Cluster3119cbin.1	SGB_566	376	4066686	14575	5327
Sample_SCA60.Cluster874	SGB_638	20	2187569	146638	56473
Sample_SPC35.mb.31	SGB_650	32	1717914	83759	32925
Sample_LCB23.Cluster647cbin.1	SGB_559	61	2691326	58120	23992
Sample_SPC42.mb.68	SGB_357	39	2745765	104881	47750
Sample_SCC68.mb.59cbin.1	SGB_256	147	3608615	68120	11103
Sample_SPB31.mb.4	SGB_281	42	2039356	79228	20387
Sample_LPC04.mb.30	SGB_480	355	3192611	11946	4063
Sample_SCC67.mb.105cbin.1	SGB_583	67	2573801	52430	18765
Sample_LCA10.Cluster2071cbin.1	SGB_126	21	2090005	195396	52323
Sample_SPB25.Cluster6772	SGB_431	98	2337580	32023	12871
Sample_LCA16.Cluster252cbin.1	SGB_10	77	5033228	102925	31816
Sample_SCA65.mb.82	SGB_540	155	2145927	19701	6799
Sample_SCC59.mb.71cbin.1	SGB_140	160	4344464	39610	12965
Sample_SPC11.mb.136	SGB_454	130	2956248	48239	8420
Sample_SCB59.mb.94cbin.1	SGB_667	82	1688736	30930	10296
Sample_YPA1.mb.5	SGB_417	591	4151092	8946	3392
Sample_LPB02.mb.67	SGB_366	134	2581119	36887	8714
Sample_SPA13.mb.3	SGB_172	121	3097449	36628	14379
Sample_SPB45.mb.45	SGB_487	479	2420508	5695	2705
Sample_SPC35.mb.4cbin.1	SGB_424	27	2668061	193910	62843
Sample_SPB30.mb.68	SGB_343	389	1818561	5229	2635
Sample_SCB69.mb.72	SGB_669	173	2343910	17897	7031
Sample_LCB20.mb.30	SGB_531	70	4231455	130825	30587
Sample_SCC65.mb.28	SGB_42	139	2613848	28861	8905
Sample_SPC35.mb.113cbin.1	SGB_493	35	1692104	74919	28759
Sample_LCB10.mb.120cbin.1	SGB_68	69	1722237	54195	9729
Sample_LPC02.mb.70cbin.1	SGB_292	30	1992037	129559	36629
Sample_YPC5.mb.51cbin.1	SGB_65	109	2342417	42432	8396
Sample_LPC04.Cluster1402cbin.1	SGB_19	38	2727673	137420	35111
Sample_SPB25.mb.82cbin.1	SGB_120	141	2092994	32385	5701
Sample_SPC27.mb.93	SGB_429	59	2674281	67428	22297
Sample_LCC25.mb.5cbin.1	SGB_643	19	2425739	236859	80532
Sample_SPB35.mb.17cbin.1	SGB_243	98	2589666	37036	14956
Sample_SPB36.mb.51	SGB_83	21	1348553	77015	37928
Sample_SPB37.mb.18	SGB_165	39	2047830	94158	29967
Sample_SPA41.mb.28	SGB_436	36	2576667	98841	40972
Sample_YPC6.mb.50	SGB_536	57	4758912	141658	40064
Sample_SPB21.mb.78cbin.1	SGB_548	53	3016675	152166	25362
Sample_SPA47.Cluster8125cbin.1	SGB_53	28	1910820	118005	34735
Sample_SPB35.mb.47	SGB_82	45	1287146	42183	14530
Sample_SPB26.Cluster12694	SGB_653	92	1573727	29270	7039
Sample_LCB10.Cluster2330cbin.1	SGB_340	18	2276375	205067	61970
Sample_SPA35.mb.50	SGB_484	410	2417858	7136	3091
Sample_SCB67.mb.142	SGB_267	57	2191413	75072	19810
Sample_SPA35.mb.46	SGB_478	443	2937177	8275	3361
Sample_SPA35.mb.22	SGB_495	33	1757649	114940	23671
Sample_SPA03.Cluster8475cbin.1	SGB_412	22	2879040	173059	77582
Sample_YPC1.Cluster660cbin.1	SGB_41	80	2138818	44904	12407

Sample_SPA47.Cluster903	SGB_44	22	2402758	224146	47311
Sample_SCB64.mb.105cbin.1	SGB_309	35	2131009	108547	23769
Sample_SPC27.mb.85	SGB_114	85	1978587	40652	11607
Sample_YPA3.Cluster1349	SGB_342	95	2621366	53645	17805
Sample_SPB47.Cluster48cbin.1	SGB_227	44	2573394	131440	27175
Sample_SPA16.mb.99cbin.1	SGB_673	217	3810275	27744	7876
Sample_SPC30.mb.56cbin.1	SGB_456	197	2850275	20821	6435
Sample_SPC39.Cluster776cbin.1	SGB_8	61	2456407	61434	18912
Sample_SPC45.mb.91	SGB_688	121	1961052	21407	8656
Sample_SPC08.mb.41	SGB_685	386	4569273	17004	5569
Sample_LPB05.mb.82	SGB_231	70	2758924	78100	20208
Sample_SPC35.mb.92	SGB_103	24	1214906	69716	34700
Sample_LCB11.Cluster7542cbin.1	SGB_413	69	2739724	64046	18732
Sample_LPC03.Cluster4989cbin.1	SGB_590	44	3165168	118556	40877
Sample_SCA64.mb.33	SGB_214	44	2344695	101972	26748
Sample_SPB25.Cluster13262cbin.1	SGB_148	114	2289779	26748	10579
Sample_SPA34.Cluster1818	SGB_26	28	5674361	222287	140879
Sample_SPC17.Cluster1240	SGB_132	152	2161345	21900	6240
Sample_SPC07.mb.71	SGB_610	100	3995957	62542	22380
Sample_SCA70.Cluster572cbin.1	SGB_274	143	2672085	32983	8737
Sample_LCA12.mb.139cbin.1	SGB_89	27	1163787	112841	18857
Sample_SCC70.mb.49	SGB_671	11	1561856	247093	122471
Sample_SPC08.Cluster9827cbin.1	SGB_587	85	3339606	60593	20887
Sample_YPC1.Cluster479cbin.1	SGB_428	57	2926833	72697	27315
Sample_SPA21.mb.75cbin.1	SGB_250	76	2031201	44394	13250
Sample_SCC70.mb.71	SGB_217	80	1919995	33958	12501
Sample_SPC45.Cluster1174cbin.1	SGB_249	49	2038549	63512	23600
Sample_LCC17.mb.22	SGB_551	265	2185682	10137	4272
Sample_SPA45.Cluster8060cbin.1	SGB_620	295	2425989	9673	4234
Sample_LCC09.mb.93	SGB_458	89	2566091	44049	16143
Sample_SPA21.mb.114cbin.1	SGB_604	51	4942244	213652	47842
Sample_SPA41.Cluster8883cbin.1	SGB_112	210	1757858	10958	3882
Sample_YPA7.mb.29	SGB_90	51	1538709	48149	12839
Sample_SPC31.Cluster1	SGB_637	25	2220875	129532	46305
Sample_LCB11.mb.55	SGB_526	26	2088177	116918	39648
Sample_SPA32.Cluster5430cbin.1	SGB_355	62	2838312	78943	23126
Sample_SPC32.mb.3cbin.1	SGB_426	66	3356855	79983	27125
Sample_SPA15.mb.13cbin.1	SGB_535	652	5952366	11952	4275
Sample_SPB08.mb.46	SGB_528	98	1766822	29425	8490
Sample_SPB36.mb.67	SGB_364	158	2085131	21722	5646
Sample_SPB07.Cluster283	SGB_647	34	1911526	83907	30279
Sample_SCC68.mb.61	SGB_262	48	1881337	73656	19125
Sample_LPB05.mb.3	SGB_527	93	1934074	33055	10134
Sample_SPB26.Cluster1230cbin.1	SGB_682	18	1853734	157734	76988
Sample_SPA13.Cluster985cbin.1	SGB_475	133	1924874	25964	6384
Sample_SPA27.Cluster135cbin.1	SGB_195	101	2779142	48499	13905
Sample_LPC04.Cluster8345	SGB_500	4	1550073	388913	311056
Sample_SPC13.mb.8	SGB_332	266	2736440	15166	4743
Sample_SPC11.Cluster4048	SGB_501	17	2029114	137216	46739
Sample_SPB25.mb.12	SGB_681	13	1685500	147385	93795
Sample_SPC08.mb.124	SGB_466	290	2723013	12240	4223
Sample_SPC07.Cluster7094	SGB_71	17	1981379	140490	57974
Sample_SPB07.Cluster1836	SGB_642	11	2191524	473789	87970
Sample_SPB07.mb.104	SGB_188	200	2541210	18142	6496
Sample_SPC22.Cluster481	SGB_627	52	1971926	73239	21494
Sample_SPC06.Cluster328cbin.1	SGB_260	119	2764307	47468	9512
Sample_YPC5.mb.73cbin.1	SGB_451	77	3035214	81008	26567
Sample_SPA42.Cluster16495cbin.1	SGB_138	142	2263009	23100	7634

Sample_SCC67.Cluster8360cbin.1	SGB_579	63	2741928	67480	21154
Sample_LCB21.mb.18cbin.1	SGB_592	55	2492854	74863	22971
Sample_SPC09.Cluster8082cbin.1	SGB_416	62	3478642	86388	32486
Sample_SCA70.mb.103cbin.1	SGB_235	142	1961826	28009	5771
Sample_LPB05.Cluster6861	SGB_6	11	2226192	309211	123745
Sample_SPB34.mb.105	SGB_683	322	2804838	11513	4355
Sample_LPA05.mb.50cbin.1	SGB_228	410	3191661	10262	3897
Sample_YPB1.mb.44cbin.1	SGB_605	98	5996150	108420	31855
Sample_SCC66.mb.109cbin.1	SGB_177	60	2927059	118919	21542
Sample_SPA10.mb.31cbin.1	SGB_452	35	2907990	169245	47862
Sample_SPB32.mb.47	SGB_351	158	2775407	26678	8217
Sample_LPC03.mb.117cbin.1	SGB_563	34	2499121	106409	41051
Sample_YPA4.mb.65cbin.1	SGB_261	91	2885011	54939	17683
Sample_SPC25.mb.82cbin.1	SGB_183	155	2355191	25944	6982
Sample_SPA07.mb.20cbin.1	SGB_622	75	3539272	68258	25724
Sample_SPB20.mb.48	SGB_282	154	1775021	15769	5947
Sample_LCC09.mb.77	SGB_407	99	2294816	39143	11278
Sample_SCA55.mb.95	SGB_193	112	2340306	40506	9845
Sample_LPA04.mb.19	SGB_211	118	2559405	39894	10153
Sample_SPB35.mb.106	SGB_187	276	2169528	10094	3869
Sample_SPA47.mb.34	SGB_317	330	2172824	8118	3138
Sample_SCA60.mb.67cbin.1	SGB_14	76	4109085	75588	34010
Sample_SCA65.Cluster164cbin.1	SGB_254	54	2003443	57863	17031
Sample_SPC34.Cluster784cbin.1	SGB_305	41	2363632	102807	25449
Sample_SPC09.Cluster4566cbin.1	SGB_29	29	4491581	233134	79182
Sample_SPC27.mb.81	SGB_233	74	2842981	61111	20150
Sample_LCA16.mb.56	SGB_178	99	2243930	36811	9908
Sample_SPC07.mb.32cbin.1	SGB_164	21	2083020	161931	44706
Sample_SCB70.Cluster6337cbin.1	SGB_73	42	2126972	89801	24409
Sample_SPC06.mb.74	SGB_241	28	2073213	105292	34072
Sample_SPC12.mb.60cbin.1	SGB_445	115	2987042	45135	13150
Sample_SCB58.mb.34cbin.1	SGB_666	150	2767597	28872	9408
Sample_YPB2.mb.74	SGB_64	122	2216675	31659	8787
Sample_SPC37.mb.21	SGB_310	69	2290700	46609	21414
Sample_SPB25.mb.92	SGB_160	66	1916159	44684	16156
Sample_SPC04.mb.92cbin.1	SGB_30	68	4603732	128118	32179
Sample_SPA03.Cluster11746cbin.1	SGB_443	21	2168871	255743	42988
Sample_SPC31.mb.68cbin.1	SGB_472	41	3480005	142601	46284
Sample_LCC09.Cluster2953cbin.1	SGB_658	14	1708442	149944	51613
Sample_SPA22.mb.96	SGB_290	218	2027989	11522	4887
Sample_LPA02.Cluster15863	SGB_91	21	1347461	96892	29935
Sample_LPB05.mb.117	SGB_319	78	2456228	56957	15196
Sample_LCB14.mb.43	SGB_203	57	2617531	72329	19861
Sample_LPC04.mb.74	SGB_664	134	2538102	27874	10181
Sample_SPB20.mb.2	SGB_303	67	2473222	59490	18926
Sample_LCB20.mb.46	SGB_279	363	1696126	5328	2659
Sample_SCC70.mb.28	SGB_529	97	3140576	49396	15263
Sample_SPB15.mb.64	SGB_224	50	2216108	60910	21538
Sample_SPC27.mb.90	SGB_375	65	2925512	84198	22038
Sample_SPA04.Cluster14222	SGB_137	410	2111042	5928	2553
Sample_LCB10.Cluster3344	SGB_612	43	3746935	151809	42787
Sample_SPA08.mb.66	SGB_461	43	2556661	77768	32064
Sample_SCA58.mb.25cbin.1	SGB_514	12	1521699	372070	43217
Sample_SCA61.mb.26	SGB_239	52	2297952	66387	23470
Sample_SPC32.mb.18cbin.1	SGB_50	48	1811368	62037	17942
Sample_SPA34.mb.10cbin.1	SGB_273	46	2602322	90884	29570
Sample_YPC5.Cluster10250cbin.1	SGB_432	44	3501426	153862	32088
Sample_SCC67.mb.12	SGB_79	39	1375372	56681	16489

Sample_SCA55.Cluster11573cbin.1	SGB_21	52	2283824	87029	24526
Sample_SPB25.Cluster11630cbin.1	SGB_119	29	1976061	100025	29165
Sample_SPB26.Cluster7424	SGB_9	27	2571837	168999	38421
Sample_LCA20.mb.98	SGB_656	428	3242367	9930	3855
Sample_LCC14.Cluster234	SGB_547	22	3304664	257562	97421
Sample_LCB17.mb.81cbin.1	SGB_623	99	2968979	46867	17078
Sample_SPC32.Cluster6603cbin.1	SGB_157	207	2966667	24033	5834
Sample_SPA35.Cluster17834cbin.1	SGB_154	19	1371900	96193	30804
Sample_LCA12.Cluster6897cbin.1	SGB_507	16	2336834	241705	70896
Sample_SPA17.mb.3cbin.1	SGB_639	44	2367105	87802	31499
Sample_SPA03.mb.24	SGB_192	170	2379244	18912	7136
Sample_YPA2.mb.71	SGB_97	50	1236524	40239	12458
Sample_SPA41.mb.71	SGB_408	74	2543783	56012	20176
Sample_SCA59.mb.65	SGB_488	96	2845867	44358	14641
Sample_SPA26.mb.80cbin.1	SGB_323	46	2287255	70075	27880
Sample_YPA4.mb.11cbin.1	SGB_151	134	2620200	32715	9808
Sample_LPA03.mb.36	SGB_494	33	2112493	112149	26576
Sample_SPB21.Cluster563cbin.1	SGB_544	41	3005580	103893	46881
Sample_LPB04.mb.91cbin.1	SGB_377	45	2876587	140942	44617
Sample_LPC01.Cluster396cbin.1	SGB_191	191	2212442	16217	5395
Sample_LCC22.mb.27	SGB_522	31	1948482	87603	36472
Sample_SPC13.mb.54	SGB_549	27	3194810	218648	79627
Sample_LCA24.mb.55	SGB_60	50	2226660	78560	18184
Sample_SPC07.Cluster12882	SGB_394	36	2646836	186627	34236
Sample_SPB42.Cluster2849cbin.1	SGB_32	35	4647577	351103	55866
Sample_SPA25.Cluster5883	SGB_674	79	2033797	41411	11505
Sample_SPB08.mb.63	SGB_289	30	1912673	130707	25570
Sample_SCC58.mb.71	SGB_190	69	2348781	53310	17709
Sample_SPA26.Cluster2066	SGB_175	28	2092136	103791	29154
Sample_SPA39.mb.89cbin.1	SGB_586	43	3149785	121713	42186
Sample_SPA34.Cluster14210	SGB_81	38	1113366	41153	14391
Sample_SCC70.mb.34	SGB_670	60	2039767	55672	15250
Sample_SPC04.mb.22cbin.1	SGB_584	175	3318643	29864	9758
Sample_SPA15.Cluster523	SGB_314	20	1623773	270695	67533
Sample_SPC10.mb.23cbin.1	SGB_46	13	2553360	426618	81642
Sample_SPB26.mb.7	SGB_649	83	1913960	32046	11718
Sample_SCC70.mb.109cbin.1	SGB_562	53	2460378	56867	25982
Sample_SCC70.Cluster12153cbin.1	SGB_334	42	2409555	116535	32497
Sample_SPC28.mb.92	SGB_360	41	3302646	137179	56175
Sample_SCB70.mb.28	SGB_95	32	1084725	50846	19875
Sample_SPA37.mb.72	SGB_521	271	4091254	21380	6968
Sample_LCB14.mb.45	SGB_657	296	2815818	12617	4769
Sample_SPB37.mb.17cbin.1	SGB_543	208	3165435	25766	7224
Sample_SPB32.mb.39	SGB_385	203	2771159	23473	5833
Sample_SPB12.mb.138	SGB_297	59	2294673	58924	23539
Sample_SPA38.Cluster5590cbin.1	SGB_596	51	3339910	114282	35294
Sample_SPB06.mb.46cbin.1	SGB_339	24	2248886	208242	33888
Sample_LCB09.mb.46cbin.1	SGB_403	292	2828857	13957	4315
Sample_LCA14.Cluster308cbin.1	SGB_655	38	2998515	131226	45095
Sample_LCB23.Cluster10438	SGB_144	121	2432101	31318	9098
Sample_YPA6.Cluster3578cbin.1	SGB_185	28	2189032	130101	45168
Sample_LPC04.mb.65cbin.1	SGB_295	106	2401899	34165	11967
Sample_LCB20.Cluster552cbin.1	SGB_582	52	2440384	68717	23276
Sample_LPC05.Cluster7412cbin.1	SGB_450	80	3321657	72109	26230
Sample_SPC06.mb.55	SGB_435	60	2752342	68959	21802
Sample_LCC17.Cluster13753cbin.1	SGB_569	75	3358006	65491	25911

Sample_SPA08.mb.119	SGB_636	90	2100421	35957	11364
Sample_SPC10.mb.11	SGB_58	162	1752837	18101	4512
Sample_SCA58.Cluster6503cbin.1	SGB_320	64	1809777	47294	12965
Sample_SPA37.Cluster18	SGB_516	316	2382714	10162	3794
Sample_SPA08.mb.13	SGB_150	30	2536629	135172	42795
Sample_LCB17.mb.100	SGB_52	44	1819040	74524	26257
Sample_LCB21.mb.96	SGB_520	139	3578815	39632	11273

Genome information		CheckM estimation		
Max length (bp)	GC content (%)	Completeness (%)	Contamination (%)	Checkm lineage
49968	62.72	90.79	2.71	Clostridia
443710	57.93	100.00	0.00	Bacteroidetes
233123	44.26	88.60	0.00	Clostridiales
132091	58.59	92.63	2.80	Clostridiales
268346	51.08	93.91	0.68	Bacteria
503057	53.23	98.65	1.68	Clostridiales
202786	56.74	88.36	0.04	Clostridiales
262829	51.23	97.66	2.81	Lachnospiraceae
305951	59.91	99.19	0.81	Actinobacteria
70866	45.49	97.85	2.02	Selenomonadales
215133	61.62	97.37	1.25	Proteobacteria
191510	53.58	99.19	1.08	Clostridia
224185	55.18	100.00	0.00	Proteobacteria
632868	27.37	94.94	1.12	Bacteria
196568	44.95	98.99	0.00	Clostridiales
408938	41.92	97.98	0.00	Clostridiales
661303	45.45	99.19	0.12	Actinobacteria
56069	48.29	87.19	3.76	Bacteroidales
286082	58.26	99.30	0.93	Clostridiales
47077	42.99	90.12	1.48	Clostridiales
316327	27.15	80.02	1.34	Clostridiales
183828	26.62	81.46	3.37	Bacteria
743981	54.90	100.00	0.00	Bacteroidetes
196112	46.15	98.11	1.10	Bacteroidales
27515	52.65	80.15	1.01	Clostridia
103759	53.31	89.82	0.58	Clostridiales
126469	61.24	86.07	1.34	Clostridiales
186791	28.52	93.48	4.58	Clostridiales
167578	52.99	98.93	0.00	Bacteroidales
216216	25.26	92.41	1.12	Bacteria
94778	53.15	97.31	0.36	Clostridiales
65183	57.22	95.52	2.57	Clostridiales
239866	40.37	98.65	0.00	Clostridiales
48011	59.66	88.04	2.02	Actinobacteria
604059	58.50	99.90	0.45	Bifidobacteriaceae
177633	58.80	93.08	1.78	Bifidobacteriaceae
98368	61.06	94.40	0.68	Clostridiales
87556	59.37	81.43	0.92	Clostridiales
120370	58.25	93.84	0.67	Clostridiales
143497	46.56	96.77	0.00	Bacteria
161733	38.07	98.11	0.75	Lactobacillales
250622	43.64	99.77	0.67	Clostridiales
179528	53.78	97.14	0.00	Prevotella
235268	46.92	98.02	1.25	Proteobacteria
155154	60.12	96.92	1.87	Clostridiales
366528	46.11	85.45	0.67	Clostridiales
134209	57.29	91.53	1.88	Clostridia
562904	45.12	98.49	0.24	Lachnospiraceae
531531	52.84	97.31	0.00	Clostridiales
267944	26.57	91.57	4.49	Bacteria
252379	30.90	100.00	0.00	Euryarchaeota
41757	35.34	92.23	1.53	Clostridiales

162991	49.83	96.15	1.05	Clostridiales
336753	37.70	97.98	1.01	Clostridiales
216053	37.11	97.76	0.34	Clostridiales
243846	33.23	98.65	0.00	Clostridiales
478966	41.15	99.32	0.00	Clostridiales
176033	40.75	94.37	0.31	Bacteroidales
310593	60.79	94.59	2.03	Bacteria
57262	61.21	90.59	2.70	Bacteria
190615	62.26	96.77	0.00	Actinobacteria
230645	50.37	95.74	0.67	Clostridiales
36668	51.17	86.46	0.00	Clostridiales
68730	42.66	93.98	1.43	Bacteria
209493	49.41	95.85	0.81	Clostridia
406168	62.91	99.53	0.00	Bifidobacteriaceae
192538	46.14	91.27	1.34	Clostridiales
613181	50.04	96.62	0.00	Bacteria
193844	61.54	99.19	0.60	Actinobacteria
555942	43.56	98.65	0.00	Clostridiales
189533	48.79	96.42	1.34	Clostridiales
187448	60.59	96.64	0.81	Clostridiales
314831	42.83	97.98	0.67	Clostridiales
181889	45.42	95.97	0.00	Clostridiales
198307	26.37	91.57	0.16	Bacteria
606694	43.74	99.62	0.00	Bacteroidales
367307	43.87	98.32	0.67	Clostridiales
220297	54.94	97.31	0.67	Clostridiales
128153	36.47	99.02	0.00	Lactobacillus
74892	46.43	90.49	0.67	Clostridiales
69898	43.53	97.46	0.65	Clostridiales
52959	43.80	88.60	3.42	Clostridiales
95991	46.40	94.66	1.86	Prevotella
387254	46.10	97.41	0.19	Bacteroidales
152756	28.08	91.57	1.69	Bacteria
134196	58.35	92.39	1.01	Clostridiales
305975	35.23	88.88	0.28	Bacteria
345924	45.01	97.76	0.00	Clostridiales
108719	29.63	100.00	0.11	Bacteria
203744	37.96	99.32	2.06	Clostridiales
66146	44.40	93.49	1.34	Clostridiales
361372	51.00	97.98	0.17	Clostridiales
133434	42.86	99.41	0.58	Lachnospiraceae
258595	48.19	95.94	0.37	Bacteroidales
197421	46.17	92.74	1.61	Clostridia
222173	57.61	97.03	1.70	Clostridiales
105175	49.73	92.09	1.00	Bacteroidales
211249	28.78	97.55	1.77	Clostridiales
221330	56.94	99.31	0.37	Clostridiales
271931	59.77	100.00	0.00	Bifidobacteriaceae
315385	48.37	99.25	0.00	Bacteroidales
127155	42.69	93.42	0.37	Bacteroidales
78360	54.40	98.03	0.22	Clostridiales
216393	26.78	92.41	0.00	Bacteria
240161	37.77	98.65	0.00	Clostridiales
463148	49.15	94.59	0.68	Bacteria
24925	56.62	84.34	0.81	Clostridia
155212	41.14	97.42	1.34	Clostridiales
194948	60.24	97.58	0.81	Actinobacteria
405371	41.13	99.51	0.00	Lachnospiraceae

120152	53.04	97.01	0.00	Actinobacteria
62322	50.75	91.44	3.37	Clostridiales
161268	65.23	97.98	1.55	Proteobacteria
604421	36.27	98.65	0.00	Clostridiales
102761	57.25	91.21	3.36	Clostridiales
87996	38.58	99.36	1.90	Selenomonadales
253722	49.48	97.84	0.00	Bacteria
320146	44.53	98.38	3.96	Lachnospiraceae
291132	42.76	95.26	1.44	Bacteroidales
778381	32.12	89.74	0.85	Bacteria
221002	46.11	93.73	0.89	Clostridiales
421019	56.08	96.41	1.35	Enterobacteriaceae
360667	36.59	99.32	0.34	Clostridiales
536808	32.57	99.47	0.00	Lactobacillales
87185	37.76	98.50	1.04	Lactobacillales
204103	40.57	92.61	0.00	Clostridiales
178154	46.30	99.51	1.45	Lachnospiraceae
329711	34.27	99.32	0.67	Clostridiales
252072	45.89	97.75	0.58	Lachnospiraceae
459526	66.31	85.48	0.69	Actinobacteria
236225	48.58	93.92	0.19	Bacteroidales
120795	49.27	92.61	2.33	Bacteroidales
620036	49.31	98.70	0.00	Clostridiales
511148	58.27	99.43	0.08	Veillonellaceae
207859	44.69	97.46	1.27	Clostridiales
177804	55.51	99.32	0.67	Clostridiales
98837	58.80	94.29	0.67	Clostridiales
144643	29.42	97.75	0.00	Bacteria
260918	29.00	98.87	1.12	Bacteria
238934	42.16	95.66	0.56	Bacteroidales
482463	49.63	94.63	0.00	Clostridiales
177962	46.74	97.98	0.00	Clostridiales
219689	38.61	99.58	0.07	Streptococcus
69148	46.05	91.38	0.63	Clostridiales
211810	36.83	89.03	0.00	Clostridiales
443010	64.26	100.00	1.15	Actinobacteria
305312	42.84	97.31	0.00	Clostridiales
163348	61.68	94.63	0.34	Clostridiales
292508	48.62	87.80	0.00	Clostridiales
622737	33.42	99.32	1.34	Clostridiales
75116	49.58	96.08	0.88	Bacteroidales
173204	46.88	96.81	1.60	Lachnospiraceae
461576	26.70	93.53	0.00	Bacteria
479183	46.44	99.25	0.19	Bacteroidales
450191	55.88	97.95	1.36	Bacteria
282057	41.83	97.41	2.75	Lachnospiraceae
299072	35.98	99.32	0.50	Clostridiales
384443	43.66	96.64	0.00	Clostridiales
59538	57.78	92.68	2.03	Bacteria
151330	55.14	99.10	0.02	Clostridiales
551912	27.83	93.25	1.28	Bacteria
480926	54.99	98.60	0.14	Clostridiales
89926	44.16	97.46	0.00	Clostridiales
404233	47.93	92.39	0.00	Clostridiales
150713	26.30	91.57	2.25	Bacteria
175566	59.48	91.72	3.69	Clostridiales
17195	32.61	85.12	3.20	Bacteria
165343	27.44	91.57	1.12	Bacteria

105377	38.72	90.62	0.95	Streptococcus
122585	58.66	92.63	0.67	Clostridiales
385285	45.22	99.19	0.19	Bacteroidales
133592	46.86	98.56	2.42	Lachnospiraceae
165765	44.13	97.31	0.34	Clostridiales
243443	45.59	96.45	1.76	Prevotella
112064	57.36	94.78	0.67	Clostridiales
330627	58.26	98.56	0.68	Clostridiales
266481	57.16	98.38	2.25	Clostridia
85574	33.37	97.30	0.13	Bacteria
146662	51.98	100.00	0.00	Firmicutes
97806	49.14	80.58	1.59	Bacteroidales
157053	44.05	95.89	2.17	Lachnospiraceae
162366	59.31	87.69	0.00	Clostridiales
328792	59.47	91.94	1.02	Clostridiales
102674	44.65	89.30	1.35	Bacteroidales
35165	60.46	80.06	0.67	Clostridiales
384817	48.09	97.55	4.84	Clostridia
197701	37.78	100.00	2.01	Clostridiales
426251	40.40	99.32	0.00	Clostridiales
150685	49.11	99.53	0.31	Bacteroidales
123612	48.54	94.31	0.63	Proteobacteria
188987	61.40	94.96	4.14	Clostridiales
302418	30.87	100.00	0.94	Bacteria
251367	41.81	100.00	0.00	Clostridiales
138811	45.99	97.98	0.00	Clostridiales
282937	40.64	97.09	0.00	Clostridiales
190034	44.98	98.80	0.00	Selenomonadales
229976	41.43	99.41	0.00	Lachnospiraceae
277822	44.15	97.98	0.34	Clostridiales
216413	45.14	94.14	0.74	Bacteroidales
44873	56.81	93.44	0.00	Bacteria
99748	52.45	99.36	0.63	Firmicutes
512656	50.10	99.34	0.95	Clostridiales
371089	59.97	95.65	0.16	Proteobacteria
237720	38.51	99.36	0.00	Clostridiales
596940	57.74	99.35	0.08	Enterobacteriaceae
266293	56.23	96.39	0.77	Enterobacteriaceae
124823	34.98	98.87	1.12	Bacteria
287798	42.06	99.41	0.49	Lachnospiraceae
283449	38.36	97.65	0.00	Clostridiales
38080	59.42	90.45	0.00	Clostridia
205869	36.98	95.11	0.00	Clostridiales
121701	45.62	97.87	0.00	Clostridiales
258911	61.30	97.31	0.67	Clostridiales
122306	44.46	91.82	2.59	Bacteroidales
392717	60.13	100.00	0.00	Actinobacteria
322817	54.72	100.00	0.00	Bacteroidetes
613077	46.40	100.00	0.48	Bacteroidetes
152525	60.85	87.58	0.67	Clostridiales
186997	25.73	94.38	3.37	Bacteria
129752	50.55	97.84	0.00	Bacteria
197639	42.40	90.31	0.77	Bacteroidales
46124	48.93	87.29	3.66	Clostridia
133956	29.93	99.05	0.00	Bacteria
27810	39.94	80.48	0.84	Clostridiales
129685	43.38	98.01	0.86	Lachnospiraceae
214376	58.42	97.31	1.34	Clostridiales

342677	41.37	97.44	2.80	Bacteroidales
291135	59.84	90.60	0.00	Clostridiales
233844	40.67	96.64	0.00	Clostridiales
143676	52.42	97.76	0.00	Clostridiales
17999	39.58	82.36	3.31	Pasteurellaceae
75798	47.98	86.45	1.53	Bacteria
132296	60.56	94.46	0.67	Clostridiales
100791	44.25	91.53	3.38	Clostridiales
122824	62.11	90.91	3.02	Clostridiales
115346	28.15	84.26	3.75	Bacteria
133035	52.53	97.76	0.00	Clostridiales
601448	54.26	100.00	0.12	Actinobacteria
361291	42.75	97.31	0.00	Clostridiales
165595	44.39	98.11	0.00	Bacteria
342223	32.24	89.74	1.14	Bacteria
264799	58.45	98.63	1.68	Clostridiales
298071	49.34	98.88	0.00	Bacteroidales
214846	56.94	94.63	4.14	Clostridiales
119261	56.52	98.43	0.00	Clostridiales
202735	36.73	95.97	0.00	Clostridiales
87264	46.82	98.19	0.29	Lactobacillus
167825	59.65	99.36	0.74	Clostridiales
178028	25.63	88.48	0.00	Bacteria
637625	43.05	99.25	0.00	Bacteroidales
290528	40.87	99.03	1.22	Lachnospiraceae
507737	44.01	99.46	0.54	Bacteria
67787	61.97	98.22	0.00	Deltaproteobacteria
307910	47.83	98.94	0.63	Clostridiales
234063	51.86	97.98	0.67	Clostridiales
130590	28.36	96.49	3.51	Bacteria
202625	53.39	90.93	0.67	Clostridiales
327921	54.32	98.20	0.00	Selenomonadales
147367	54.25	99.51	0.96	Bacteroidetes
753688	34.69	99.22	0.00	Lactobacillus
217851	33.71	98.65	0.00	Clostridiales
93584	42.39	90.25	4.31	Streptococcus
149520	56.14	94.74	2.01	Clostridiales
177542	41.40	89.76	1.18	Bacteroidales
69802	52.47	95.91	0.38	Bacteroidales
146124	32.16	92.72	0.22	Clostridiales
260158	37.62	99.62	0.00	Lactobacillales
160356	63.44	100.00	0.00	Actinobacteria
117747	43.16	100.00	0.32	Clostridiales
410843	38.77	95.82	3.83	Bacteroidales
131959	58.57	98.38	3.23	Clostridia
118072	57.15	88.49	3.36	Clostridiales
372012	43.44	95.81	1.50	Bacteroidales
105622	37.29	96.22	4.72	Bacteria
177550	38.89	89.57	4.74	Lactobacillales
89972	45.98	90.99	2.42	Clostridia
141640	57.79	92.61	0.67	Clostridiales
274785	42.98	93.62	0.00	Clostridiales
328650	45.55	98.88	0.23	Bacteroidales
243201	58.35	91.27	2.03	Clostridiales
492424	55.67	99.63	0.18	Enterobacteriaceae
453051	54.87	99.32	0.67	Clostridiales
97444	38.53	91.25	0.54	Lachnospiraceae

183832	55.74	94.36	2.35	Clostridiales
52924	63.42	87.89	0.81	Clostridiales
378979	58.62	98.65	1.34	Clostridiales
441583	56.93	99.03	1.44	Bacteroidetes
133650	52.67	97.42	1.47	Bacteria
165176	48.65	91.93	0.03	Clostridia
146171	42.51	86.10	2.41	Bacteroides
473331	58.60	98.32	0.67	Clostridiales
214338	31.25	100.00	0.63	Firmicutes
480631	28.47	96.47	0.70	Clostridiales
484519	41.80	99.16	0.00	Clostridiales
50242	58.20	85.79	3.60	Clostridiales
201593	34.54	98.29	3.26	Clostridiales
246184	43.60	97.39	1.49	Bacteroidales
163636	41.14	99.27	0.03	Lachnospiraceae
338848	60.10	97.98	0.67	Clostridiales
108401	61.84	99.32	0.13	Clostridiales
242429	47.84	92.61	0.00	Clostridiales
368723	60.04	98.65	0.02	Clostridiales
210542	29.61	92.13	0.56	Bacteria
473043	49.25	99.34	1.58	Clostridiales
66126	68.13	97.17	1.57	Actinobacteria
272924	42.39	99.46	0.00	Bacteria
127427	40.03	90.66	1.14	Lachnospiraceae
367825	44.96	99.19	0.81	Actinobacteria
389686	28.57	98.66	0.11	Bacteria
197980	49.89	98.89	0.00	Lactobacillales
120821	56.69	95.07	0.92	Clostridiales
42347	56.40	98.41	0.68	Clostridiales
67824	42.40	94.63	0.67	Clostridiales
367684	55.44	97.95	0.00	Bacteria
101655	45.45	92.13	0.81	Clostridia
385242	62.17	87.33	2.69	Clostridia
365895	40.49	97.76	0.67	Clostridiales
183501	43.01	97.09	0.67	Clostridiales
35025	52.07	87.74	3.38	Bacteria
238308	41.83	98.73	0.63	Clostridiales
186606	56.33	99.38	0.60	Selenomonadales
284348	39.47	98.65	0.34	Clostridiales
408784	54.28	98.75	0.63	Proteobacteria
215744	36.78	100.00	0.64	Lactobacillales
240989	31.80	100.00	0.00	Euryarchaeota
180922	63.53	89.51	0.00	Clostridia
47486	45.46	88.68	2.91	Clostridiales
187882	59.62	91.49	0.00	Clostridiales
172924	61.15	93.62	0.67	Clostridiales
151689	29.07	99.05	0.00	Bacteria
326703	41.78	99.32	0.00	Clostridiales
371048	61.18	97.50	0.63	Proteobacteria
208225	33.29	94.63	0.89	Clostridiales
398552	62.18	92.61	0.04	Clostridiales
276647	56.96	96.63	0.00	Bacteroidetes
236616	48.32	99.42	3.12	Lachnospiraceae
182279	26.62	93.82	0.00	Bacteria
267115	30.40	100.00	2.25	Bacteria
242342	49.90	95.30	0.67	Clostridiales
244220	45.14	99.19	0.12	Actinobacteria
604172	45.33	99.61	0.00	Bacteroidales

60150	57.75	88.93	1.40	Clostridiales
73340	54.93	88.97	2.01	Clostridiales
182353	42.73	99.03	0.85	Lachnospiraceae
158308	59.68	94.25	3.62	Clostridiales
355365	29.35	91.57	1.12	Bacteria
615990	42.73	97.33	0.19	Bacteroides
600548	58.12	97.95	0.00	Bacteria
45506	43.31	87.99	3.21	Clostridiales
178816	60.00	100.00	0.00	Bacteria
260026	44.10	97.98	0.00	Clostridiales
511615	27.05	92.13	1.85	Bacteria
309875	61.27	98.07	1.44	Bacteroidetes
224692	42.02	98.10	0.59	Bacteroides
254035	25.50	91.57	1.69	Bacteria
303710	59.78	93.91	2.70	Bacteria
256880	38.09	98.94	0.00	Lactobacillus
311834	62.23	93.28	0.04	Clostridiales
259349	53.26	100.00	0.00	Selenomonadales
135105	48.02	94.19	0.74	Bacteroidales
397834	49.38	99.09	0.72	Lachnospiraceae
177474	57.54	97.98	0.67	Clostridiales
226856	43.56	98.38	2.56	Lachnospiraceae
145450	36.64	97.48	0.00	Clostridiales
98894	61.17	93.95	0.67	Clostridiales
263221	61.60	93.02	0.00	Clostridiales
87054	58.85	97.86	1.21	Deltaproteobacteria
106740	48.41	90.87	0.67	Clostridiales
134145	39.84	97.15	1.27	Clostridiales
104018	45.91	94.63	0.67	Clostridiales
142280	53.28	97.31	0.67	Clostridiales
144847	46.03	95.30	0.00	Clostridiales
53616	42.83	94.64	4.60	Lactobacillales
169370	56.09	98.37	1.11	Enterobacteriaceae
463355	42.98	99.46	0.00	Bacteria
278911	49.93	94.35	0.00	Clostridia
425066	47.65	99.36	0.95	Clostridiales
33737	37.02	80.74	2.89	Clostridiales
68807	59.67	88.54	4.87	Clostridiales
149910	63.16	96.27	0.62	Proteobacteria
193535	24.15	94.94	1.28	Bacteria
489957	53.24	98.65	0.00	Clostridiales
401575	42.74	98.34	2.43	Bacteroidales
107199	59.95	93.78	1.52	Bacteroidetes
391712	43.74	97.76	0.00	Clostridiales
43485	61.82	85.90	2.35	Clostridiales
104456	54.66	95.28	0.94	Bacteroidales
259072	43.86	98.65	0.34	Clostridiales
233756	41.42	98.06	2.78	Lachnospiraceae
113913	46.46	92.49	1.99	Lachnospiraceae
301777	27.98	97.60	0.00	Euryarchaeota
85584	42.51	80.44	2.07	Lachnospiraceae
167034	41.82	96.64	0.00	Clostridiales
488322	52.81	97.04	0.00	Clostridiales
128264	41.58	98.39	1.16	Clostridiales
208962	40.97	98.10	1.58	Clostridiales
37554	51.90	84.64	0.67	Clostridiales
104601	60.76	100.00	0.00	Actinobacteria

201408	62.43	99.37	0.62	Proteobacteria
246388	51.30	94.60	1.35	Bacteroidetes
296099	57.60	93.73	2.35	Clostridiales
56797	52.28	89.23	2.03	Bacteria
385263	30.30	98.50	0.00	Bacteria
618116	62.61	100.00	0.00	Actinobacteria
107697	57.26	98.12	0.00	Proteobacteria
108630	56.64	93.52	1.14	Enterobacteriaceae
428351	41.31	95.32	0.10	Lachnospiraceae
70265	42.90	90.21	2.09	Clostridia
235790	38.70	98.12	1.47	Lactobacillales
94440	46.78	97.66	3.74	Lachnospiraceae
421562	41.83	99.32	0.00	Clostridiales
62858	47.55	91.88	0.74	Bacteroidales
490264	43.51	99.46	0.00	Bacteria
266621	54.80	96.77	0.00	Bacteroidetes
841953	34.59	99.22	0.00	Lactobacillus
130711	47.22	97.71	1.61	Clostridia
252150	26.82	96.77	0.00	Clostridiales
280798	45.53	96.64	0.00	Clostridiales
174242	44.54	93.83	0.00	Bacteroidales
41345	37.23	88.28	1.48	Clostridiales
136084	63.17	99.11	0.59	Deltaproteobacteria
268859	53.12	91.05	0.67	Clostridiales
103273	59.60	91.27	2.68	Clostridiales
243762	40.92	91.77	1.61	Clostridiales
168257	61.35	96.40	0.27	Clostridia
398220	28.47	91.01	2.81	Bacteria
186252	57.27	88.25	0.00	Clostridiales
111875	58.90	92.61	1.97	Deltaproteobacteria
35871	58.58	87.36	3.83	Clostridiales
58403	47.98	86.24	2.57	Bacteroidales
330399	49.88	96.64	0.00	Clostridiales
191520	58.85	93.28	0.68	Clostridiales
93685	61.92	95.13	2.01	Clostridiales
413981	45.55	90.45	0.00	Clostridia
98098	62.41	90.28	0.81	Actinobacteria
41611	49.59	89.06	4.75	Clostridiales
98089	40.81	93.73	0.67	Clostridiales
148908	37.45	97.31	0.67	Clostridiales
73708	63.03	89.97	0.96	Bacteroidetes
166698	69.23	91.21	0.84	Bacteria
189953	41.01	98.53	0.58	Lachnospiraceae
180394	39.56	98.63	0.27	Pasteurellaceae
487224	36.05	98.65	0.00	Clostridiales
141520	56.56	95.37	2.72	Clostridiales
170047	34.26	91.60	1.84	Bacilli
495994	56.93	95.97	0.00	Clostridiales
308631	55.75	98.88	0.37	Bacteroidales
223289	42.16	99.24	0.19	Bacteroidales
230808	48.78	99.36	0.00	Clostridiales
242390	28.02	80.16	1.75	Clostridiales
549211	41.54	95.97	0.00	Clostridiales
304978	48.01	97.98	0.00	Clostridiales
128661	52.79	99.18	0.00	Lactobacillales
291132	43.55	99.98	0.90	Selenomonadales

696379	56.52	99.67	0.77	Pseudomonadales
295671	43.27	99.21	1.21	Lachnospiraceae
65497	46.61	94.29	0.67	Clostridiales
121416	45.60	88.16	3.81	Bacteroidales
468562	44.30	90.84	0.00	Bacteroidales
122807	48.58	98.73	0.02	Firmicutes
766001	51.88	93.95	0.83	Clostridia
144046	29.41	89.93	1.33	Bacteria
219523	55.58	99.01	0.84	Clostridiales
54088	42.58	94.62	2.15	Bacteria
408573	59.89	100.00	0.81	Actinobacteria
204303	27.54	97.60	0.00	Euryarchaeota
171564	55.33	98.46	0.38	Bacteroidales
261562	38.76	98.65	0.00	Clostridiales
237671	55.28	94.63	1.34	Clostridiales
261706	49.65	95.39	0.00	Clostridiales
62968	59.29	96.90	1.21	Clostridia
133078	51.19	97.03	0.37	Bacteroidales
275862	46.63	99.18	0.00	Lactobacillales
110434	44.51	97.31	0.67	Clostridiales
306540	59.91	93.91	2.70	Bacteria
69904	59.34	94.23	1.60	Bacteroidetes
166746	43.84	100.00	0.00	Bacteria
199103	41.92	97.91	0.95	Clostridiales
138931	59.53	100.00	0.81	Actinobacteria
65243	48.28	88.97	0.00	Clostridiales
104934	46.37	93.95	2.95	Clostridiales
298584	58.65	97.93	0.14	Clostridiales
23978	60.20	80.55	2.02	Clostridia
624506	45.73	93.23	1.28	Lachnospiraceae
27634	43.60	82.63	0.56	Clostridiales
79545	54.43	94.63	0.09	Bacteroidales
295621	45.88	98.52	0.38	Bacteroidales
89860	48.48	100.00	0.11	Proteobacteria
187466	50.83	89.16	0.70	Clostridiales
273587	45.47	97.15	0.63	Firmicutes
184058	45.23	92.17	0.00	Clostridiales
165547	51.01	100.00	0.63	Firmicutes
224789	58.53	100.00	0.00	Bacteria
69224	45.16	93.45	0.97	Lactobacillales
165080	49.03	94.30	0.00	Clostridiales
375918	60.18	100.00	0.00	Bifidobacteriaceae
141725	58.94	92.95	4.42	Clostridiales
226590	27.22	91.57	0.56	Bacteria
207952	47.14	94.40	0.00	Clostridiales
233902	46.38	98.55	1.55	Lachnospiraceae
369842	45.07	99.42	0.00	Bacteroidales
414333	59.20	100.00	0.48	Bacteroidetes
352589	47.37	95.60	0.00	Bacteria
114090	26.96	93.82	1.12	Bacteria
136029	30.05	98.40	0.80	Euryarchaeota
562128	51.21	97.27	0.00	Clostridiales
23822	56.96	83.28	1.40	Clostridiales
124223	58.18	97.42	0.49	Clostridiales
35211	58.68	92.76	1.88	Clostridia
393307	51.37	96.77	0.00	Clostridia
326874	43.05	99.32	0.00	Clostridiales
210745	49.21	97.46	0.00	Proteobacteria

638445	55.36	98.75	0.63	Proteobacteria
254081	56.74	96.64	0.67	Clostridiales
140797	40.08	96.84	1.32	Streptococcus
170079	53.05	96.64	4.05	Clostridiales
277882	60.65	97.31	1.28	Clostridiales
193549	46.49	98.48	1.74	Bacillus
81638	44.99	95.99	1.29	Clostridiales
185858	57.38	95.27	0.68	Bacteria
72256	44.23	90.65	0.81	Clostridia
84644	56.18	96.36	3.34	Enterobacteriaceae
159991	59.82	94.29	0.34	Clostridiales
188312	25.25	91.01	1.12	Bacteria
603123	44.23	100.00	0.17	Clostridiales
216238	51.22	94.25	0.68	Prevotella
158100	56.46	89.93	0.34	Clostridiales
82056	33.58	99.05	0.00	Bacteria
577628	55.20	99.10	0.11	Enterobacteriaceae
93466	45.42	94.35	1.15	Lactobacillales
163584	45.41	91.72	0.31	Bacteroidales
118099	56.08	98.43	0.00	Clostridiales
239004	27.51	91.29	1.69	Bacteria
307668	44.83	96.62	1.12	Bacteria
225256	45.38	98.64	0.68	Prevotella
169842	49.13	98.10	0.00	Clostridiales
174977	61.05	91.94	3.38	Clostridiales
123622	57.82	84.64	1.07	Clostridiales
148011	57.23	98.27	4.55	Bacteria
40820	51.12	90.73	1.06	Bacteroidetes
50258	43.66	84.95	1.03	Bacteroidales
204565	37.01	99.32	0.67	Clostridiales
541835	41.60	96.12	0.37	Bacteroidales
64186	39.21	92.02	2.27	Streptococcus
203736	24.95	93.82	2.97	Bacteria
242254	59.69	100.00	0.81	Actinobacteria
339925	48.74	99.26	0.00	Bacteria
221704	42.08	98.65	0.67	Clostridiales
311424	42.84	99.10	0.67	Clostridiales
52326	43.43	93.14	1.76	Bacteroidales
61109	57.53	94.85	0.00	Bacteria
77380	44.95	90.96	3.27	Lachnospiraceae
384478	60.47	99.55	0.00	Bifidobacteriaceae
201156	60.39	93.95	0.67	Clostridiales
150562	56.10	96.50	0.00	Bacteria
396438	60.57	98.79	0.81	Euryarchaeota
128335	48.22	97.17	0.81	Clostridia
215352	57.16	97.53	0.00	Clostridiales
534856	46.62	94.38	1.40	Clostridiales
67385	50.46	97.22	0.00	Clostridiales
653659	48.92	95.96	0.00	Clostridia
331104	54.46	98.38	0.00	Actinobacteria
117211	46.70	83.47	3.99	Lachnospiraceae
246019	45.35	99.40	0.60	Selenomonadales
644042	56.40	100.00	0.23	Bifidobacteriaceae
65513	59.35	93.92	0.99	Clostridiales
118622	60.67	100.00	1.41	Actinobacteria
172181	57.88	92.61	0.67	Clostridiales
179472	44.75	94.30	0.95	Clostridiales
84336	34.06	93.66	1.65	Bacteria

161923	52.60	98.05	0.00	Bacteroidales
179984	50.29	81.22	0.19	Bacteroidales
238576	41.29	98.16	3.64	Lachnospiraceae
78023	54.02	88.59	2.84	Clostridiales
577464	48.66	91.21	0.68	Bacteria
42580	72.77	93.56	4.55	Actinomycetaceae
60664	61.66	94.28	1.48	Clostridiales
280203	41.70	97.73	1.54	Bacteroides
313870	42.55	99.31	0.00	Clostridiales
354493	43.85	96.28	0.63	Clostridiales
70052	49.61	95.56	0.63	Clostridiales
201721	53.84	98.86	0.38	Bacteroidales
159820	57.77	94.63	1.34	Clostridiales
124426	51.02	97.31	0.00	Clostridiales
198396	44.37	97.48	0.56	Bacteroidales
48089	51.30	91.10	2.01	Clostridiales
170967	41.79	94.63	0.61	Lachnospiraceae
102112	58.79	95.10	2.04	Clostridiales
119502	62.49	90.15	1.83	Clostridiales
47634	59.15	88.70	4.31	Clostridiales
29006	58.11	83.41	0.28	Clostridiales
240052	59.43	93.91	2.36	Bacteria
208203	60.41	93.28	0.67	Clostridiales
221255	56.84	88.59	0.67	Clostridiales
889727	47.97	98.52	0.67	Enterobacteriaceae
143748	61.98	92.72	0.67	Clostridiales
143136	49.68	96.97	0.34	Clostridiales
402958	48.96	95.89	0.00	Clostridiales
203933	47.62	100.00	0.90	Selenomonadales
246564	58.90	91.27	0.67	Clostridiales
146895	43.95	97.78	3.71	Clostridiales
83609	61.99	95.80	3.02	Clostridiales
87350	51.94	95.56	0.00	Firmicutes
171088	59.68	91.05	0.00	Clostridiales
139437	47.84	96.09	0.24	Clostridiales
403665	50.75	99.96	0.04	Enterobacteriaceae
380018	37.47	99.32	0.00	Clostridiales
785241	28.01	100.00	0.09	Clostridiales
784595	52.89	97.17	0.00	Clostridia
33945	56.25	85.18	1.51	Clostridiales
223645	24.88	92.69	2.81	Bacteria
173093	56.16	97.85	2.75	Clostridiales
226827	52.96	97.98	0.00	Clostridiales
113541	41.17	93.71	2.70	Bacteria
158317	45.70	95.30	1.34	Clostridiales
15993	61.26	83.02	1.23	Clostridiales
147622	51.45	93.17	0.63	Bacteroidetes
168147	63.97	87.24	1.34	Clostridiales
215292	35.67	98.51	0.67	Clostridiales
33212	34.11	80.09	1.31	Bacilli
358443	44.65	98.52	0.37	Bacteroidales
218463	44.86	95.57	0.00	Lachnospiraceae
573640	48.70	92.33	0.81	Clostridia
211357	58.12	98.65	0.00	Clostridiales
157085	47.06	96.77	0.00	Bacteria
269705	57.62	95.74	1.34	Clostridiales
395016	33.32	99.32	1.68	Clostridiales
148317	26.12	93.33	0.00	Bacteria

171681	36.81	98.79	0.29	Gammaproteobacteria
359503	38.24	99.61	0.00	Lactobacillales
385809	46.11	94.59	1.35	Bacteria
38595	48.53	87.57	2.20	Lachnospiraceae
515577	58.21	99.27	0.48	Bacteroidetes
119252	46.11	92.74	0.75	Bacteroidales
90330	27.15	98.94	1.06	Clostridiales
278938	28.16	81.91	2.33	Clostridiales
502089	48.13	96.37	0.00	Clostridia
198416	60.06	100.00	0.81	Actinobacteria
92483	58.98	92.30	1.19	Clostridiales
131902	26.07	93.33	1.33	Bacteria
110103	41.93	97.07	0.88	Lachnospiraceae
99836	58.83	98.38	1.08	Clostridia
239486	38.62	98.65	0.34	Clostridiales
85746	42.65	95.97	1.40	Clostridiales
196381	52.78	99.19	0.81	Clostridia
168082	58.88	100.00	0.00	Bacteroidetes
365099	37.16	98.65	0.00	Clostridiales
141309	59.65	87.27	0.85	Clostridiales
394436	32.83	88.88	0.85	Bacteria
551659	59.02	99.40	0.00	Bacteroidetes
274650	54.06	100.00	0.00	Selenomonadales
479553	32.40	99.32	1.34	Clostridiales
610102	56.20	98.63	0.77	Enterobacteriaceae
132972	43.74	97.92	1.20	Lactobacillales
393329	55.79	89.26	0.00	Clostridiales
202378	60.57	97.27	0.31	Clostridiales
245012	54.55	97.14	0.00	Clostridiales
302851	49.11	99.24	0.00	Bacteroidales
93272	26.05	92.69	2.25	Bacteria
216711	44.81	81.42	0.79	Rhodospirillales
153913	43.10	91.69	1.25	Prevotella
321648	57.55	85.90	0.81	Clostridiales
516079	57.02	96.87	0.00	Proteobacteria
121896	55.45	98.15	0.81	Euryarchaeota
161358	50.15	97.54	0.00	Bacteroidales
264763	44.05	97.09	0.00	Clostridiales
348708	44.31	97.98	0.67	Clostridiales
123548	27.63	91.01	0.00	Bacteria
164921	60.01	99.11	0.59	Deltaproteobacteria
37915	59.66	93.17	1.36	Clostridiales
86146	59.72	92.25	1.87	Bacteroidetes
120337	38.37	87.52	1.17	Lachnospiraceae
396020	51.54	98.32	0.67	Clostridiales
179475	48.90	98.74	0.37	Bacteroidales
399720	52.01	97.27	0.00	Clostridiales
58531	48.83	92.22	4.63	Lachnospiraceae
323227	59.62	95.23	0.23	Clostridiales
139185	28.20	91.50	0.00	Bacteria
300329	51.45	98.65	0.67	Clostridiales
109945	53.01	96.42	0.34	Clostridiales
200208	50.99	97.93	1.01	Prevotella
200197	45.38	96.83	3.16	Clostridiales
343803	44.36	100.00	0.12	Clostridiales
152513	38.43	99.05	0.00	Bacteroidales

181885	62.41	99.19	0.81	Actinobacteria
53256	38.57	88.25	4.91	Selenomonadales
108476	48.00	95.07	0.00	Clostridiales
26320	63.38	93.48	1.30	Deltaproteobacteria
215501	54.60	81.29	1.53	Clostridia
194712	45.70	98.92	0.00	Bacteria
186894	62.59	100.00	1.38	Deltaproteobacteria

Taxonomic position	
Phylum	Species
Firmicutes	Christensenellales sp. CAG6
Bacteroidota	Alistipes sp. CAG:3
Firmicutes	uncultured Ruminococcus sp.
Firmicutes	Christensenellales sp. CAG3
Verrucomicrobiota	Coraliomargarita sp. CAG:1
Firmicutes	Ruminococcaceae bacterium
Firmicutes	Angelakisella sp.CAG1
Firmicutes	Lachnoclostridium sp. An118
Actinobacteriota	Coriobacteriaceae bacterium
Firmicutes	Megasphaera micronuciformis
Proteobacteria	Sutterella sp. CAG3
Firmicutes	Clostridia bacterium
Proteobacteria	Sutterella wadsworthensis
Firmicutes	Clostridium sp. CAG:710
Firmicutes	Eubacterium siraeum
Firmicutes	Clostridiales bacterium 41_21_two_genomes
Actinobacteriota	Eggerthella sp. CAG:368
Bacteroidota	Bacteroides sp, CAG1
Firmicutes	Clostridiales bacterium
Firmicutes	unclutured Clostridiales sp. CAG:51
Firmicutes	Clostridium sp. CAG:492
Firmicutes	Clostridium sp. CAG:609
Bacteroidota	Alistipes indistinctus
Bacteroidota	Muribaculaceae sp. CAG4
Firmicutes	Clostridiales bacterium
Firmicutes	Clostridiales bacterium
Firmicutes	Oscillospiraceae sp. CAG5
Firmicutes	uncultured Clostridium sp.
Bacteroidota	Prevotella sp. CAG:485
Firmicutes	Mycoplasma sp. CAG:472
Firmicutes	Clostridium sp. CAG:58
Firmicutes	Ruminococcaceae sp. CAG4
Firmicutes	Lachnospiraceae bacterium
Actinobacteriota	Coriobacteriia bacterium
Actinobacteriota	Bifidobacterium dentium
Actinobacteriota	Bifidobacterium breve
Firmicutes	Clostridiales sp. CAG:9
Firmicutes	Butyricicoccus sp. OM04-18BH
Firmicutes	Clostridiales bacterium
Proteobacteria	Azospirillum sp. CAG1
Firmicutes	Enterococcus faecium
Firmicutes	Firmicutes bacterium CAG:882
Bacteroidota	Prevotella sp.
Proteobacteria	Sutterella sp. CAG:521
Firmicutes	Faecalibacterium prausnitzii
Firmicutes	Firmicutes bacterium CAG:475
Firmicutes	Clostridiales sp. CAG:40
Firmicutes	Roseburia sp. AM16-25
Firmicutes	Clostridiales bacterium
Firmicutes	Firmicutes bacterium CAG:460
Euryarchaeota	Methanobrevibacter smithii CAG:186
Firmicutes	Eubacterium sp. AF15-50

Firmicutes	Clostridiales bacterium
Firmicutes	uncultured Clostridium sp.
Firmicutes	Clostridiales bacterium
Firmicutes	Eubacterium sp. AF36-5BH
Firmicutes	uncultured Coprococcus sp.
Bacteroidota	Bacteroides coprocola CAG:162
Verrucomicrobiota	Lentisphaeria bacterium
Verrucomicrobiota	Victivallis sp. CAG:3
Actinobacteriota	Eggerthella sp. AM16-19
Firmicutes	Clostridiales sp. CAG:35
Firmicutes	Mogibacterium sp. CAG1
Bacteroidota	Odoribacter sp. AF21-41
Firmicutes	Clostridiales bacterium
Actinobacteriota	Bifidobacterium bifidum
Firmicutes	Roseburia sp. AM59-24XD
Elusimicrobia	uncultured Elusimicrobium sp. CAG2
Actinobacteria	uncultured Slackia sp. CAG1
Firmicutes	Lachnospiraceae bacterium
Firmicutes	Clostridiales sp. CAG:30
Firmicutes	Clostridiales sp. CAG:6
Firmicutes	Clostridiales sp. CAG:5
Firmicutes	Ruminococcaceae bacterium
Firmicutes	Bacilli sp. CAG2
Bacteroidota	Bacteroides sp. CAG:20
Firmicutes	Coprococcus catus
Firmicutes	Clostridiales sp. CAG:4
Firmicutes	Lactobacillus crispatus
Firmicutes	Clostridiales sp. CAG:47
Firmicutes	uncultured Blautia sp.
Firmicutes	Coprococcus catus
Bacteroidota	Prevotella copri
Bacteroidota	Bacteroides stercoris
Firmicutes	Clostridium sp. CAG:451
Firmicutes	Clostridiales sp. CAG:23
Cyanobacteria	Candidatus Gastranaerophilales bacterium HUM_10
Firmicutes	Ruminococcaceae bacterium
Firmicutes	Acholeplasmatales bacterium
Firmicutes	uncultured Eubacterium sp.
Firmicutes	Clostridiales sp. CAG:31
Firmicutes	Ruminococcus sp. CAG3
Firmicutes	Ruminococcus lactaris
Bacteroidota	Bacteroidaceae sp. CAG1
Firmicutes	Acidiphilium sp. CAG:1
Firmicutes	Faecalibacterium prausnitzii
Bacteroidota	Paraprevotella sp. CAG1
Firmicutes	Clostridium sp. CAG:265
Firmicutes	Faecalibacterium prausnitzii
Actinobacteriota	Bifidobacterium adolescentis
Bacteroidota	Paraprevotella clara CAG:116
Bacteroidota	Bacteroides sp. CAG:1076
Firmicutes	Clostridiales sp. CAG:21
Firmicutes	Firmicutes bacterium
Firmicutes	Eubacterium sp. CAG:251
Verrucomicrobiota	Opitutales sp. CAG:2
Firmicutes	Christensenellales sp. CAG4
Firmicutes	Eubacterium sp. CAG:3
Actinobacteria	uncultured Eggerthella sp.CAG2
Firmicutes	Eubacterium rectale

Actinobacteriota	Eggerthella sp. CAG:209
Firmicutes	Anaerovoracaceae sp. CAG2
Proteobacteria	Sutterella parvirubra
Firmicutes	Firmicutes bacterium CAG:341
Firmicutes	Oscillibacter sp. CAG3
Firmicutes	Veillonella parvula
Proteobacteria	Azospirillum sp. CAG:239
Firmicutes	Lachnospiraceae bacterium OM04-12BH
Bacteroidota	uncultured Bacteroides sp.
Cyanobacteria	Cyanobacteria bacterium UBA10660
Firmicutes	Clostridiales sp. CAG:29
Proteobacteria	Raoultella ornithinolytica
Firmicutes	uncultured Ruminococcus sp.
Firmicutes	Lactobacillus salivarius
Firmicutes	Streptococcus infantarius
Firmicutes	Ruminococcaceae sp. CAG19
Firmicutes	uncultured Clostridium sp.
Firmicutes	Eubacterium ventriosum
Firmicutes	Hungatella hathewayi
Actinobacteria	uncultured Olsenella sp. CAG1
Bacteroidota	Bacteroides sp. CAG:598
Bacteroidota	Muribaculaceae sp. CAG5
Firmicutes	Enterocloster bolteae
Firmicutes	Mitsuokella multacida
Firmicutes	uncultured Blautia sp.
Firmicutes	Butyricicoccus sp. AM28-25
Firmicutes	Ruminiclostridium sp. CAG1
Firmicutes	Firmicutes bacterium CAG:345
Fusobacteriota	Fusobacterium mortiferum
Bacteroidota	Bacteroides sp. AM16-15
Firmicutes	Ruminococcaceae sp. CAG7
Firmicutes	uncultured Ruminococcus sp.
Firmicutes	Streptococcus anginosus
Firmicutes	Lachnospiraceae bacterium AM48-27BH
Firmicutes	Eubacterium sp. CAG:4
Actinobacteriota	Eggerthella lenta
Firmicutes	Ruminococcaceae sp. CAG17
Firmicutes	Clostridiales sp. CAG:26
Firmicutes	Clostridiales bacterium
Firmicutes	Eubacterium sp. CAG:274
Bacteroidota	Muribaculaceae bacterium Isolate-102 (HZI)
Firmicutes	Roseburia sp. CAG:182
Firmicutes	Bacilli sp. CAG1
Bacteroidota	Bacteroides uniformis
Verrucomicrobiota	Akkermansia sp. BIOML-A61
Firmicutes	Lachnospiraceae bacterium
Firmicutes	Eubacterium sp. CAG:76
Firmicutes	Anaerotruncus sp. CAG:528
Verrucomicrobiota	Verrucomicrobia bacterium CAG:312_58_20
Firmicutes	Clostridiales sp. CAG:20
Firmicutes	Clostridium sp. CAG:417
Firmicutes	Clostridia bacterium
Firmicutes	Clostridium sp. TM06-18
Firmicutes	unclutured Clostridiales sp. CAG:32
Firmicutes	Clostridium sp. CAG:628
Firmicutes	Clostridia bacterium
Firmicutes	Massilimicrobiota sp. An134
Firmicutes	Clostridium sp. CAG:7

Firmicutes	Streptococcus thermophilus
Firmicutes	Clostridiales sp. CAG:25
Bacteroidota	Parabacteroides johnsonii
Firmicutes	uncultured Clostridium sp.
Firmicutes	Clostridium sp. CAG:9
Bacteroidota	Prevotella copri
Firmicutes	Clostridiales sp. CAG:19
Firmicutes	Faecalibacterium prausnitzii
Firmicutes	Clostridiales sp. CAG:37
Firmicutes	Holdemanella biformis
Firmicutes	Dialister succinatiphilus
Bacteroidota	Prevotella sp. CAG:1058
Firmicutes	Lachnospiraceae bacterium
Firmicutes	Angelakisella sp.CAG2
Firmicutes	Firmicutes bacterium CAG:176
Bacteroidota	Parabacteroides gordonii
Firmicutes	Oscillibacter sp. CAG5
Firmicutes	Clostridiales bacterium
Firmicutes	uncultured Eubacterium sp.
Firmicutes	Ruminococcus bromii
Bacteroidota	Prevotella sp. 885
Proteobacteria	Sutterella sp. CAG2
Firmicutes	Oscillibacter sp.
Firmicutes	Coprobacillus cateniformis
Firmicutes	Butyrivibrio sp. CAG:318
Firmicutes	Ruminococcus sp.
Firmicutes	Clostridium sp. CAG:964
Firmicutes	Veillonella sp. CAG:1
Firmicutes	uncultured Ruminococcus sp.
Firmicutes	Eubacterium sp. CAG:180
Bacteroidota	Prevotella sp. CAG1
Synergistota	Synergistes sp. 3_1_syn1
Firmicutes	Dialister sp.
Firmicutes	Hungatella hathewayi
Proteobacteria	Sutterella sp. CAG1
Firmicutes	Firmicutes bacterium AM10-47
Proteobacteria	Klebsiella pneumoniae
Proteobacteria	Klebsiella oxytoca
Firmicutes	Clostridium sp. CAG:568
Firmicutes	Ruminococcus gnavus
Firmicutes	Eubacterium sp. CAG:1
Firmicutes	Clostridiales bacterium
Firmicutes	Eubacterium sp. CAG:252
Firmicutes	Firmicutes bacterium CAG:238
Firmicutes	Lawsonibacter asaccharolyticus
Bacteroidota	Bacteroides plebeius
Actinobacteria	uncultured Eggerthella sp.CAG1
Bacteroidota	Alistipes sp. AF17-16
Bacteroidota	Alistipes sp. CAG:1
Firmicutes	Oscillibacter sp.
Firmicutes	Clostridium sp. CAG:914
Proteobacteria	Azospirillum sp. 51_20
Bacteroidota	Parabacteroides sp. CAG1
Firmicutes	Clostridiales sp. CAG:50
Firmicutes	Coprobacillus sp. 8_1_38FAA
Firmicutes	Ruminococcus sp. CAG2
Firmicutes	Eubacterium ramulus
Firmicutes	Oscillibacter sp.

Bacteroidota	Bacteroides sp. CAG:2
Firmicutes	Clostridiales bacterium
Firmicutes	Clostridium sp. CAG:590
Firmicutes	uncultured Ruminococcaceae sp.CAG1
Proteobacteria	Haemophilus parainfluenzae
Verrucomicrobiota	Lentisphaeria sp. CAG2
Firmicutes	Clostridiales bacterium
Firmicutes	uncultured Blautia sp.
Firmicutes	Oscillospiraceae sp. CAG2
Firmicutes	Firmicutes bacterium
Firmicutes	Ruminococcaceae sp. CAG10
Actinobacteriota	Eggerthella sp. CAG:298
Firmicutes	Coprococcus eutactus
Firmicutes	Clostridium innocuum
Cyanobacteria	Fusobacterium sp. CAG:815
Firmicutes	Ruminococcaceae sp. CAG2
Bacteroidota	Prevotella stercorea
Firmicutes	Clostridiales bacterium
Firmicutes	Firmicutes bacterium CAG:110
Firmicutes	uncultured Ruminococcus sp.
Firmicutes	Lactobacillus rhamnosus
Firmicutes	Faecalibacterium prausnitzii
Firmicutes	Clostridium sp. CAG:5
Bacteroidota	Bacteroides fragilis
Firmicutes	uncultured Roseburia sp.
Bacteroidota	Butyricimonas sp. CAG1
Desulfobacterota	Desulfovibrio fairfieldensis
Firmicutes	Fusicatenibacter saccharivorans
Firmicutes	Eubacterium sp. CAG:786
Firmicutes	Clostridium sp. CAG:3
Firmicutes	Firmicutes bacterium CAG:24053_14
Firmicutes	Acidaminococcus sp. CAG:542
Bacteroidota	Alistipes putredinis
Firmicutes	Lactobacillus gasseri
Firmicutes	Eubacterium sp. CAG:581
Firmicutes	Streptococcus parasanguinis
Firmicutes	Ruminococcaceae bacterium AM28-23LB
Bacteroidota	Bacteroides sp. CAG:1
Bacteroidota	Muribaculaceae sp. CAG3
Firmicutes	Eubacterium sp. CAG:5
Firmicutes	Enterococcus faecalis
Actinobacteriota	Asaccharobacter celatus
Firmicutes	Clostridium sp. TM06-18
Bacteroidota	Bacteroides sp. AF16-49
Firmicutes	Clostridiales sp. CAG:39
Firmicutes	Clostridiales sp. CAG:24
Bacteroidota	Bacteroides oleiciplenus
Firmicutes	uncultured Clostridium sp.
Firmicutes	Enterococcus avium
Firmicutes	Clostridiales sp. CAG:48
Firmicutes	Ruminococcaceae sp. CAG13
Firmicutes	Clostridium sp. CAG:10
Bacteroidota	Prevotella sp. CAG:255
Firmicutes	Oscillibacter sp. CAG2
Proteobacteria	Enterobacter cloacae
Firmicutes	Clostridiales sp. CAG:13
Firmicutes	Eubacterium sp. CAG:2

Firmicutes	Clostridiales sp. CAG:16
Firmicutes	Ruminococcaceae sp. CAG11
Firmicutes	Ruminococcaceae sp. CAG20
Bacteroidota	Bacteroidales bacterium 55_9
Bacteroidetes	uncuitured Flavobacteriales sp. CAG2
Firmicutes	Clostridiales bacterium
Bacteroidota	Bacteroides finegoldii
Firmicutes	Evtepia gabavorous
Firmicutes	Megamonas rupellensis
Firmicutes	Intestinibacter bartlettii
Firmicutes	Eubacterium sp. CAG:38
Firmicutes	Clostridiales sp. CAG:11
Firmicutes	unclutured Cellulosilyticum sp. CAG1
Bacteroidota	Bacteroides fragilis
Firmicutes	Roseburia inulinivorans
Firmicutes	Firmicutes bacterium CAG:129
Firmicutes	Flavonifractor plautii
Firmicutes	Ruminococcaceae sp. CAG8
Firmicutes	uncultured Oscillibacter sp.
Firmicutes	Coprobacillus sp. CAG:605
Firmicutes	Hungatella hathewayi
Actinobacteriota	uncultured Olsenella sp. CAG1
Bacteroidota	Butyricimonas virosa
Firmicutes	Roseburia sp. CAG:1
Actinobacteriota	Eggerthella sp. CAG:1427
Firmicutes	Acholeplasmatales bacterium
Firmicutes	Lactobacillus oris
Firmicutes	Clostridiales sp. CAG:18
Firmicutes	Ruminococcaceae bacterium
Firmicutes	Clostridiales bacterium
Verrucomicrobiota	Akkermansia muciniphila
Firmicutes	Acidiphilium sp. CAG:727
Firmicutes	Christensenellales sp. CAG1
Firmicutes	Roseburia sp. CAG:303
Firmicutes	Clostridiales sp. CAG:34
Verrucomicrobiota	Lentisphaeria sp. CAG1
Firmicutes	Ruminococcus sp. AF21-42
Firmicutes	Acidaminococcus sp. CAG:542
Firmicutes	Eubacterium sp. OM08-24
Proteobacteria	Sutterella sp. AM11-39
Firmicutes	Streptococcus mutans
Euryarchaeota	Methanobrevibacter sp. CAG1
Firmicutes	Christensenellales sp. CAG5
Firmicutes	Clostridium sp. CAG:242
Firmicutes	Ruminococcaceae bacterium
Firmicutes	Firmicutes bacterium CAG:176_63_11
Firmicutes	Coprobacillus sp. AM29-13
Firmicutes	Coprococcus sp. OM04-5BH
Proteobacteria	Sutterella sp. CAG4
Firmicutes	Eubacterium sp. CAG:274
Firmicutes	Clostridiales bacterium
Bacteroidota	Alistipes sp.
Firmicutes	Eisenbergiella sp. OF01-20
Firmicutes	Bacilli sp. CAG3
Fusobacteriota	Fusobacterium ulcerans
Firmicutes	Clostridium sp. CAG:11
Actinobacteriota	Cryptobacterium sp. CAG:338
Bacteroidota	Parabacteroides merdae

Firmicutes	Oscillibacter sp. CAG4
Firmicutes	Clostridiales sp. CAG:28
Firmicutes	Roseburia intestinalis
Firmicutes	Clostridiales sp. CAG:8
Firmicutes	Clostridium sp. CAG:533
Bacteroidota	Bacteroides thetaiotaomicron
Verrucomicrobiota	Akkermansia muciniphila
Firmicutes	uncultured Ruminococcus sp.
Synergistota	Pyramidobacter piscolens
Firmicutes	Ruminococcaceae sp. CAG21
Firmicutes	Mycoplasma sp. CAG:956
Bacteroidota	Alistipes sp. CAG:4
Bacteroidota	Bacteroides caccae
Firmicutes	Clostridium sp. CAG:524
Verrucomicrobiota	Victivallis sp. CAG:1
Firmicutes	Lactobacillus amylovorus
Firmicutes	Clostridiales bacterium
Firmicutes	Megasphaera elsdenii
Bacteroidota	Prevotella sp. CAG:592
Firmicutes	Roseburia hominis
Firmicutes	Oscillibacter sp. 57_20
Firmicutes	Lachnospiraceae bacterium
Firmicutes	Clostridium sp. AM27-31LB
Firmicutes	Oscillospiraceae sp. CAG4
Firmicutes	Clostridiales bacterium
Proteobacteria	Desulfovibrio desulfuricans
Firmicutes	Eubacterium sp. CAG:6
Firmicutes	Tyzzerella nexilis
Firmicutes	Ruminococcaceae sp. CAG12
Firmicutes	Ruminococcus sp. CAG:330
Firmicutes	Ruminococcaceae sp. CAG1
Firmicutes	Enterococcus casseliflavus
Proteobacteria	Enterobacter asburiae
Bacteroidetes	Butyricimonas synergistica
Firmicutes	Clostridiales sp. CAG:45
Firmicutes	Hungatella hathewayi
Firmicutes	Anaerostipes hadrus
Firmicutes	Oscillibacter sp. CAG1
Proteobacteria	Sutterella megalosphaeroides
Firmicutes	Mycoplasma sp. CAG:611
Firmicutes	Ruminococcaceae bacterium
Bacteroidota	Bacteroides intestinalis
Bacteroidota	Alistipes timonensis
Firmicutes	Clostridium sp. CAG:167
Firmicutes	Lawsonibacter sp. CAG1
Bacteroidota	Muribaculaceae sp. CAG1
Firmicutes	Clostridium sp. OM07-9AC
Firmicutes	Roseburia sp. OF03-24
Firmicutes	Lachnospiraceae bacterium AM40-2BH
Euryarchaeota	Methanosphaera cuniculi
Firmicutes	Dorea sp. CAG:317
Firmicutes	Ruminococcus sp. CAG1
Firmicutes	Clostridium sp. CAG:413
Firmicutes	Blautia obeum
Firmicutes	uncultured Lachnospira sp.
Firmicutes	Ruminococcaceae sp. CAG15
Actinobacteriota	Coriobacteriia sp.CAG1

Proteobacteria	Sutterella wadsworthensis
Bacteroidota	Alistipes sp. CAG:435
Firmicutes	Ruminococcaceae bacterium
Verrucomicrobiota	Lentisphaeria bacterium
Firmicutes	Clostridium sp. CAG:288
Actinobacteriota	Olsenella sp. AF21-51
Proteobacteria	Sutterella sp. CAG:397
Proteobacteria	Klebsiella grimontii
Firmicutes	Dorea sp. OM07-5
Firmicutes	Firmicutes bacterium CAG:552_39_19
Firmicutes	Enterococcus mundtii
Firmicutes	Eisenbergiella tayi
Firmicutes	Bacteroides pectinophilus
Bacteroidota	Prevotella sp. CAG:891
Bacteroidota	Odoribacter splanchnicus
Firmicutes	unclutured Alistipes sp. CAG:5
Firmicutes	Lactobacillus gasseri
Firmicutes	Ruminococcaceae sp. CAG14
Firmicutes	Clostridium sp. CAG:12
Firmicutes	Ruminococcus sp. CAG:403
Bacteroidota	Prevotella sp. CAG2
Firmicutes	Lachnospiraceae sp. CAG1
Desulfobacterota	Desulfovibrio piger
Firmicutes	Ruminococcaceae bacterium
Firmicutes	Oscillospiraceae sp. CAG6
Firmicutes	Ruminococcus sp. AF37-6AT
Firmicutes	Clostridiales sp. CAG:38
Firmicutes	Clostridium sp. CAG:8
Firmicutes	Clostridiales sp. CAG:10
Desulfobacterota	Desulfovibrio sp.
Firmicutes	unnamed Ruminococcaceae sp. CAG1
Bacteroidota	Bacteroides sp. OF04-15BH
Firmicutes	Ruminococcus sp. CAG:579
Firmicutes	Faecalibacterium prausnitzii
Firmicutes	Oscillospiraceae sp. CAG1
Firmicutes	Clostridiales sp. CAG:49
Actinobacteria	Collinsella intestinalis
Firmicutes	Lachnospiraceae bacterium OF09-33XD
Firmicutes	uncultured Ruminococcaceae sp.CAG2
Firmicutes	Ruminococcus sp. CAG:624
Bacteroidota	Alistipes dispar
Verrucomicrobia	uncultured Verrucomicrobia sp. CAG2
Firmicutes	Ruminococcus torques
Proteobacteria	Haemophilus parainfluenzae
Firmicutes	Eubacterium sp. CAG:86
Firmicutes	Faecalibacterium prausnitzii
Firmicutes	Turicibacter sanguinis
Firmicutes	unclutured Clostridium sp. CAG:9
Bacteroidota	Prevotella sp. CAG3
Bacteroidota	Bacteroides vulgatus
Firmicutes	Clostridium sp. AM28-20LB
Firmicutes	Clostridiales bacterium
Firmicutes	Anaerotruncus sp. CAG:528
Firmicutes	Tyzzerella sp.
Firmicutes	Lactobacillus fermentum
Firmicutes	Phascolarctobacterium sp. CAG:207

Proteobacteria	Pseudomonas helleri
Firmicutes	Roseburia faecis
Firmicutes	Ruminococcus sp. CAG1
Bacteroidota	Bacteroides clarus
Bacteroidota	Prevotellaceae bacterium
Firmicutes	Dialister sp. CAG:357
Firmicutes	Candidatus Borkfalkia ceftriaxoniphila
Firmicutes	Clostridium sp. CAG:307
Firmicutes	Clostridiales sp. CAG:14
Bacteroidota	Butyricimonas faecalis
Actinobacteriota	Collinsella aerofaciens
Euryarchaeota	Methanosphaera sp. A6
Bacteroidota	Muribaculaceae sp. CAG2
Firmicutes	Clostridium sp. CAG:230
Firmicutes	Firmicutes bacterium CAG:124
Firmicutes	Ruminococcus sp. CAG:382
Firmicutes	Christensenellales sp. CAG2
Bacteroidota	Prevotella sp. CAG:520
Firmicutes	Lactobacillus mucosae
Firmicutes	Clostridium sp. CAG:632
Verrucomicrobiota	Victivallis vadensis
Bacteroidota	Alistipes sp .CAG:1
Firmicutes	Clostridiaceae bacterium OM02-2AC
Firmicutes	Blautia obeum
Actinobacteria	uncultured Coriobacteriia sp.CAG1
Firmicutes	uncultured Clostridium sp.
Firmicutes	Blautia sp. KGMB01111
Firmicutes	Ruthenibacterium lactatiformans
Firmicutes	Clostridiales sp. CAG:42
Firmicutes	Firmicutes bacterium CAG:534
Firmicutes	Clostridium sp. CAG:13
Firmicutes	uncultured Prevotella sp. CAG:1
Bacteroidota	Parabacteroides merdae
Proteobacteria	Burkholderiales bacterium
Firmicutes	Clostridia sp. CAG1
Firmicutes	Dialister invisus
Firmicutes	Eubacterium sp. CAG:841
Firmicutes	Dialister succinatiphilus
Synergistota	Cloacibacillus sp. CAG1
Firmicutes	Weissella cibaria
Firmicutes	uncultured Clostridium sp.
Actinobacteriota	Bifidobacterium longum
Firmicutes	Oscillospiraceae sp. CAG3
Firmicutes	Mycoplasma sp. CAG:1
Firmicutes	Clostridiales sp. CAG:3
Firmicutes	Clostridium sp. CAG:510
Bacteroidota	Parabacteroides distasonis
Bacteroidota	Alistipes communis
Proteobacteria	Acetobacter sp. 46_36
Firmicutes	Clostridium sp. CAG:6
Euryarchaeota	Methanobrevibacter woesei
Firmicutes	Ruminococcaceae bacterium
Firmicutes	Clostridiales sp. CAG:41
Firmicutes	Clostridiales sp. CAG:22
Firmicutes	Clostridiales sp. CAG:36
Firmicutes	Clostridium sp. CAG:2
Firmicutes	Coprococcus eutactus CAG:665
Proteobacteria	Proteobacteria bacterium CAG:139

Proteobacteria	Sutterella sp. AM11-39
Firmicutes	Ruminococcaceae sp. CAG22
Firmicutes	Streptococcus salivarius
Firmicutes	uncultured Ruminococcus sp.
Firmicutes	uncultured Flavonifractor sp.
Firmicutes	Bacillus velezensis
Firmicutes	Ruminococcus sp. AF20-12LB
Verrucomicrobiota	Verrucomicrobia bacterium CAG:312_58_20
Firmicutes	unclutured Clostridiales sp. CAG:26
Proteobacteria	Leclercia adecarboxylata
Firmicutes	uncultured Clostridium sp.
Firmicutes	Clostridium sp. CAG:4
Firmicutes	Coprococcus sp. BIOML-A1
Bacteroidota	Prevotella sp.
Firmicutes	Clostridiales bacterium
Firmicutes	Catenibacterium sp. AM22-15
Proteobacteria	Klebsiella oxytoca
Firmicutes	Enterococcus asini
Bacteroidota	Bacteroides fluxus
Firmicutes	Firmicutes bacterium CAG:110
Firmicutes	Clostridium sp. CAG:594
Elusimicrobia	uncultured Elusimicrobium sp. CAG1
Bacteroidota	Prevotella copri
Firmicutes	Clostridium sp. AF20-17LB
Firmicutes	Oscillibacter sp.
Firmicutes	Clostridiales sp. CAG:12
Firmicutes	Oscillibacter sp.
Bacteroidota	Alistipes sp. CAG:2
Bacteroidota	Bacteroides sp. CAG:530
Firmicutes	Anaerostipes hadrus
Bacteroidota	Bacteroides salyersiae
Firmicutes	Streptococcus vestibularis
Firmicutes	Firmicutes bacterium
Actinobacteriota	Collinsella sp. AM34-10
Bacteroidota	Flavobacteriales bacterium
Firmicutes	Clostridium sp. OM05-6BH
Firmicutes	Eubacterium sp.
Bacteroidota	Parabacteroides sp. AM58-2XD
Bacteroidota	Flavobacteriales sp. CAG1
Firmicutes	Dorea sp. OM02-2LB
Actinobacteriota	Bifidobacterium animalis
Firmicutes	Clostridiales sp. CAG:17
Bacteroidota	Flavobacteriales sp. CAG2
Candidatus Thermoplasmatota	unclutured Methanomassiliicoccaceae sp. CAG1
Firmicutes	Selenomonadales bacterium
Firmicutes	Faecalibacterium prausnitzii
Firmicutes	Clostridiales sp. CAG:44
Firmicutes	uncultured Ruminococcus sp.
Firmicutes	Clostridiales bacterium
Bacteroidota	uncultured CAG1
Firmicutes	Lachnospiraceae bacterium
Firmicutes	Phascolarctobacterium sp. CAG:266
Actinobacteriota	Bifidobacterium pseudocatenulatum
Firmicutes	Gemmiger sp. CAG2
Actinobacteriota	Coriobacteriia bacterium
Firmicutes	Firmicutes bacterium CAG:170
Firmicutes	Blautia sp. OM07-19
Firmicutes	Eubacterium sp. AM28-29

Bacteroidota	Prevotella sp. CAG:474
Bacteroidota	Prevotella sp. CAG:5226
Firmicutes	Lachnospiraceae bacterium
Firmicutes	Clostridiales sp. CAG:15
Verrucomicrobiota	Opitutales sp. CAG:1
Actinobacteria	Actinomyces radicidentis
Firmicutes	Pseudoflavonifractor sp. An184
Bacteroidota	Bacteroides ovatus
Firmicutes	uncultured Ruminococcus sp.
Firmicutes	Blautia sp. CAG:257
Firmicutes	Clostridium sp. AF27-2AA
Bacteroidota	Prevotella sp. CAG:1031
Firmicutes	Firmicutes bacterium CAG:124
Firmicutes	Ruminococcus sp. OM06-36AC
Bacteroidota	Bacteroides plebeius
Firmicutes	Clostridiales bacterium
Firmicutes	Dorea longicatena
Firmicutes	Gemmiger sp. CAG1
Firmicutes	Clostridiales sp. CAG:7
Firmicutes	Gemmiger sp. CAG5
Firmicutes	Ruminococcaceae sp. CAG18
Verrucomicrobiota	Victivallis sp. CAG:2
Firmicutes	Firmicutes bacterium CAG:83
Firmicutes	Ruminococcaceae sp. CAG9
Proteobacteria	Enterobacteriaceae bacterium 9_2_54FAA
Firmicutes	Flavonifractor sp. CAG1
Firmicutes	Ruminococcus sp. CAG4
Firmicutes	Clostridiales sp. CAG:2
Firmicutes	Phascolarctobacterium succinatutens
Firmicutes	Oscillibacter sp.
Firmicutes	Ruminococcus sp. AF46-10NS
Firmicutes	Pseudoflavonifractor sp. An184
Firmicutes	Dialister sp.CAG1
Firmicutes	Ruminococcaceae sp. CAG6
Firmicutes	Clostridiales bacterium
Proteobacteria	Escherichia coli
Firmicutes	Butyrivibrio sp.
Firmicutes	Clostridium perfringens
Firmicutes	uncultured Firmicutes sp.CAG1
Firmicutes	Clostridiales sp. CAG:33
Firmicutes	Firmicutes bacterium
Firmicutes	Ruminococcaceae sp. CAG5
Firmicutes	Eubacterium sp. CAG:115
Verrucomicrobia	uncultured Verrucomicrobia sp. CAG1
Firmicutes	Ruminococcaceae sp. CAG16
Firmicutes	Clostridiales sp. CAG:27
Bacteroidota	Alistipes sp. CAG:435
Firmicutes	Oscillibacter sp.CAG1
Firmicutes	Eubacterium sp.
Firmicutes	Turicibacter sanguinis
Bacteroidota	Bacteroides eggerthii
Firmicutes	Roseburia sp. CAG1
Firmicutes	Subdoligranulum sp. CAG:314
Firmicutes	Oscillibacter sp.
Proteobacteria	Azospirillum sp.
Firmicutes	Firmicutes bacterium CAG:137
Firmicutes	uncultured Eubacterium sp.
Firmicutes	Clostridium sp. CAG:1

Proteobacteria	Succinatimonas sp. CAG:777
Firmicutes	Lactococcus garvieae
Verrucomicrobiota	Coraliomargarita sp. CAG:312
Firmicutes	Roseburia sp. CAG:2
Bacteroidota	Alistipes onderdonkii
Bacteroidota	Bacteroides coprophilus
Firmicutes	Romboutsia ilealis
Firmicutes	Clostridium sp. CAG:452
Firmicutes	Clostridiales sp. CAG:46
Actinobacteriota	Collinsella aerofaciens
Firmicutes	Gemmiger sp. CAG4
Firmicutes	Firmicutes bacterium CAG:321
Firmicutes	Dorea longicatena
Firmicutes	Clostridiales sp. CAG:43
Firmicutes	Ruminococcaceae bacterium
Firmicutes	Clostridiales sp. CAG:1
Firmicutes	Clostridium sp. CAG:226
Bacteroidota	Alistipes shahii
Firmicutes	Lachnospira eligens
Firmicutes	Gemmiger sp. CAG3
Cyanobacteria	Candidatus Gastranaerophilales bacterium HUM_2
Bacteroidota	Alistipes finegoldii CAG:68
Firmicutes	Megasphaera sp. NM10
Firmicutes	Eubacterium sp. CAG:156
Proteobacteria	Enterobacter cloacae
Firmicutes	Streptococcus downei
Firmicutes	Clostridiales sp. CAG:32
Firmicutes	Subdoligranulum sp. 60_17
Firmicutes	Ruminococcus sp. CAG:379
Bacteroidota	Prevotella sp. AG:487_50_53
Firmicutes	Acholeplasma sp. CAG:878
Proteobacteria	unclutured Alphaproteobacteria sp. CAG1
Bacteroidota	Prevotella sp. BCRC 81118
Firmicutes	Clostridia bacterium
Proteobacteria	Mesosutterella multiformis
Thermoplasmatota	Methanomassiliicoccaceae sp. CAG1
Bacteroidota	Prevotella sp. CAG:279
Firmicutes	Ruminococcus sp. CAG:563
Firmicutes	Clostridium sp. OM07-9AC
Firmicutes	Clostridium sp. CAG:762
Desulfobacterota	Bilophila wadsworthia
Firmicutes	uncultured Pseudoflavonifractor sp. CAG1
Bacteroidota	Alistipes timonensis
Firmicutes	uncultured Clostridium sp.
Firmicutes	Butyricimonas sp. CAG1
Bacteroidota	Paraprevotella xylaniphila
Firmicutes	Ruminococcaceae bacterium
Firmicutes	uncultured Clostridium sp.
Firmicutes	Gemmiger sp. An50
Firmicutes	Clostridium spiroforme
Firmicutes	Clostridium sp. CAG:169
Firmicutes	Firmicutes bacterium CAG:272
Bacteroidota	Prevotella sp. CAG:1320
Firmicutes	uncultured Blautia sp.
Firmicutes	uncultured Ruminococcus sp.
Bacteroidota	Coprobacter fastidiosus

Actinobacteriota	uncultured Collinsella sp.
Firmicutes	Veillonella dispar
Firmicutes	Ruminococcaceae sp. CAG3
Proteobacteria	Desulfovibrio sp. AM18-2
Firmicutes	Peptococcus sp. CAG:1
Proteobacteria	Proteobacteria bacterium CAG:495
Proteobacteria	Bilophila sp. 4_1_30

Supplementary Table 5 Significant differential indicators for asthma identified during/after the trial Remarks: P, Probio-M8 group; C, placebo group; A, 0d; B, 1M; C, 3M

Probiotic group									
	**Result statistics**						***P*-value, T-test**		
Differential indicators	Mean_PA	Mean_PB	Mean_PC	SD_PA	SD_PB	SD_PC	PA vs PB	PA vs PC	PB vs PC
Mini-mental State Examination (MMSE) score	25.26	26.33	26.63	4.01	3.69	3.75	<0.001	<0.001	0.04
Hamilton Anxiety Scale (HAMA) score	15.65	13.23	11.17	8.31	7.11	5.94	<0.001	<0.001	<0.001
Hamilton Depression Scale-17 (HADM-17) score	9.17	7.46	6.63	6.13	4.28	4.26	<0.001	<0.001	<0.001
Parkinson's Disease Sleep Scale (PDSS) score	111.08	116.63	121.02	20.59	20.51	17.19	<0.01	<0.001	<0.01
Visual Analogue Scale (VAS) score	0.77	0.56	0.58	1.60	1.27	1.15	0.24	0.21	0.87
Unified PD Rating Scale-III (UPDRS- III) score	19.38	17.97	17.45	9.38	9.24	9.15	0.04	0.02	0.02
Activities of daily living (ADL) score	20.38	19.72	19.52	7.81	7.62	7.54	0.14	0.11	0.41
Patient-Assessment of Constipation Quality Of Life (PAC-QCL) score	72.52	60.02	54.88	17.72	13.15	12.02	<0.001	<0.001	<0.001
Bristol scores	2.73	3.40	3.58	1.06	0.64	0.57	<0.001	<0.001	0.07
Difficulty in defecation	2.50	1.81	1.63	0.65	0.53	0.60	<0.001	<0.001	0.01
Hardness of feces	2.27	1.63	1.35	0.91	0.73	0.69	<0.001	<0.001	<0.01
Incomplete defecation	1.65	0.85	0.58	1.18	0.89	0.70	<0.001	<0.001	0.02
Assisted defecation by hand	0.92	0.52	0.25	0.93	0.71	0.48	<0.001	<0.001	0.00
Number/week of spontaneous defecation	3.13	4.00	4.24	1.66	1.61	1.73	<0.001	<0.001	0.12
Number/week of completed defecation	1.42	2.84	3.33	1.44	1.80	1.71	<0.001	<0.001	<0.01

Placebo group									
	**Result statistics**						***P*-value, T-test**		
Differential indicators	Mean_CA	Mean_CB	Mean_CC	SD_CA	SD_CB	SD_CC	CA vs CB	CA vs CC	CB vs CC
Mini-mental State Examination (MMSE) score	25.47	25.71	25.88	4.37	4.32	4.41	0.03	<0.01	0.21
Hamilton Anxiety Scale (HAMA) score	14.91	14.21	13.85	5.72	5.61	5.28	0.15	<0.001	0.44
Hamilton Depression Scale-17 (HADM-17) score	9.21	8.68	8.56	5.52	5.00	4.98	<0.001	<0.001	0.10
Parkinson's Disease Sleep Scale (PDSS) score	126.41	127.47	127.85	15.51	13.94	14.15	0.15	0.10	0.60
Visual Analogue Scale (VAS) score	0.50	0.47	0.47	1.09	1.01	1.01	0.33	0.33	1.00
Unified PD Rating Scale-III (UPDRS- III) score	18.12	18.00	18.53	6.80	6.86	6.97	0.40	0.07	<0.01
Activities of daily living (ADL) score	22.44	22.29	22.41	10.05	9.75	9.70	0.33	0.88	0.33
Patient-Assessment of Constipation Quality Of Life (PAC-QCL) score	69.71	69.53	69.38	15.22	15.70	15.07	0.89	0.84	0.85
Bristol scores	2.68	2.79	2.91	1.13	0.80	0.66	0.33	0.07	0.04

Difficulty in defecation	2.32	2.26	2.21	0.72	0.78	0.80	0.33	0.16	0.33
Hardness of feces	2.06	2.12	1.97	0.87	0.76	0.89	0.42	0.37	0.02
Incomplete defecation	1.38	1.18	1.06	1.03	0.82	0.80	<0.01	<0.001	0.04
Assisted defecation by hand	0.79	0.56	0.53	1.02	0.91	0.88	0.03	0.02	0.33
Number/week of spontaneous defecation	2.78	2.91	3.01	1.40	1.27	1.29	0.18	0.02	0.03
Number/week of completed defecation	1.25	1.22	1.40	1.25	1.21	1.16	0.70	0.26	0.09

**Probiotic group vs Placebo group**			
	***P*-value, Wilcox-test**		
Differential indicators	CA vs PA	CB vs PB	CC vs PC
Mini-mental State Examination (MMSE) score	0.71	0.57	0.44
Hamilton Anxiety Scale (HAMA) score	0.91	0.35	0.02
Hamilton Depression Scale-17 (HADM-17) score	0.93	0.49	0.16
Parkinson’s Disease Sleep Scale (PDSS) score	<0.01	0.02	0.09
Visual Analogue Scale (VAS) score	0.64	0.89	0.80
Unified PD Rating Scale-III (UPDRS- III) score	0.85	0.51	0.26
Activities of daily living (ADL) score	0.52	0.29	0.18
Patient-Assessment of Constipation Quality Of Life (PAC-QCL) score	0.53	0.00	<0.001
Bristol scores	0.80	<0.001	<0.001
Difficulty in defecation	0.24	0.00	0.00
Hardness of feces	0.20	0.00	0.00
Incomplete defecation	0.30	0.08	0.01
Assisted defecation by hand	0.38	0.75	0.22
Number/week of spontaneous defecation	0.41	<0.01	<0.01
Number/week of completed defecation	0.98	<0.001	<0.001

Supplementary Table 6 Significant differential species-level genome bins (SGBs) between probiotic and placebo groups at different time points Remarks:P, Probio-M8 group; C, placebo group; A, 0d; B, 1M; C, 3M

Probiotic group										
**Differential SGBs**		**Result statistics (RPKM)**						**P-value, T-test**		
SGBs ID	Taxonomy	Mean_PA	Mean_PB	Mean_PC	SD_PA	SD_PB	SD_PC	PA vs TB	PB vs PC	PA vs PC
SGB_120	Weissella cibaria	0.04	0.14	0.00	0.11	0.92	0.00	0.45	0.30	0.03
SGB_174	Ruminococcus sp. CAG:1	0.00	0.07	0.06	0.03	0.24	0.18	0.10	0.83	0.05
SGB_220	Evtepia gabavorous	0.67	0.34	0.58	1.11	0.38	1.13	0.05	0.19	0.68
SGB_224	Oscillibacter sp.CAG:1	0.02	0.15	0.08	0.07	0.57	0.18	0.15	0.47	0.04
SGB_240	Firmicutes bacterium CAG:176_63_11	0.39	0.26	1.12	0.97	0.74	2.84	0.53	0.05	0.11
SGB_255	Clostridiales sp. CAG:16	0.42	0.27	0.76	0.65	0.44	1.21	0.21	0.01	0.10
SGB_261	Firmicutes bacterium CAG:124	3.55	1.38	4.16	7.02	2.66	7.27	0.05	0.02	0.70
SGB_285	Clostridiales sp. CAG:30	0.04	0.09	0.14	0.11	0.23	0.32	0.26	0.44	0.01
SGB_309	Ruminococcaceae sp. CAG:22	0.02	0.08	0.32	0.06	0.17	1.32	0.03	0.23	0.13
SGB_332	uncultured Ruminococcus sp.	0.31	0.04	0.16	0.93	0.10	0.37	0.05	0.05	0.30
SGB_384	uncultured Ruminococcus sp.	1.98	0.93	1.87	2.43	1.88	3.24	0.04	0.13	0.85
SGB_403	uncultured Clostridium sp.	0.22	0.33	0.32	0.27	0.28	0.33	0.05	0.79	0.14
SGB_430	Clostridium sp. CAG:58	0.13	0.08	0.25	0.31	0.19	0.45	0.37	0.03	0.16
SGB_444	Firmicutes bacterium AM10-47	0.57	0.47	0.24	1.04	1.00	0.37	0.65	0.13	0.04
SGB_465	uncultured Clostridium sp.	1.10	1.48	0.49	2.03	3.16	0.95	0.39	0.04	0.07
SGB_536	Parabacteroides distasonis	1.87	1.15	1.23	1.74	1.06	1.21	0.03	0.94	0.03
SGB_647	Bifidobacterium animalis	0.16	4.57	2.51	1.06	10.66	7.86	<0.01	0.33	0.05
SGB_689	unnamed Ruminococcaceae sp. CAG:1	0.15	0.04	0.16	0.49	0.09	0.42	0.12	0.05	0.95
SGB_83	Mycoplasma sp. CAG:1	0.22	0.43	0.07	0.69	1.16	0.33	0.28	0.05	0.20

Placebo group										
**Differential SGBs**		**Result statistics (RPKM)**						**P-value, T-test**		
SGBs ID	Taxonomy	Mean_CA	Mean_CB	Mean_CC	SD_CA	SD_CB	SD_CC	CA vs CB	CBvs CC	CA vs CC
SGB_567	Butyricimonas sp. CAG:1	0.03	0.02	0.00	0.08	0.06	0.00	0.77	0.05	0.09

Between the probiotic and placebo groups										
**Differential SGBs**		**Result statistics (RPKM)**								
SGBs ID	Taxonomy	Mean_PA	Mean_CA	Mean_PB	Mean_CB	Mean_PC	Mean_CC	SD_PA	SD_CA	SD_PB
SGB_368	Roseburia inulinivorans	1.07	1.42	0.80	1.76	1.08	1.59	1.54	1.27	1.22
SGB_392	Eubacterium ventriosum	0.43	0.41	0.25	0.41	0.50	0.40	0.60	0.55	0.52
SGB_438	Roseburia sp. CAG:182	0.00	0.03	0.00	0.06	0.08	0.04	0.02	0.11	0.03
SGB_375	Eubacterium sp.	0.09	0.11	0.04	0.12	0.18	0.14	0.36	0.20	0.22

SGB_27	Klebsiella oxytoca	0.00	0.22	0.00	0.47	0.11	0.44	0.00	0.80	0.00
SGB_127	Lactobacillus fermentum	0.00	0.25	0.00	0.26	0.08	0.22	0.02	0.92	0.00
SGB_589	Prevotella sp. CAG:3	0.25	0.25	0.00	0.28	0.00	0.12	1.67	0.79	0.00
SGB_50	Azospirillum sp.	0.37	0.19	0.25	0.22	0.07	0.17	1.81	0.51	1.53
SGB_654	Methanobrevibacter smithii CAG:186	0.23	0.50	0.25	0.57	0.57	1.11	0.62	1.07	0.94
SGB_407	Dorea longicatena	0.34	0.60	0.24	0.48	0.36	0.54	0.49	0.87	0.40
SGB_195	Faecalibacterium prausnitzii	8.24	8.38	5.47	7.62	7.05	6.84	10.70	7.54	5.72
SGB_647	Bifidobacterium animalis	0.16	0.08	4.57	0.06	2.51	0.04	1.06	0.35	10.66
SGB_377	Lachnospira eligens	2.48	0.87	2.36	0.90	1.86	1.08	4.89	0.81	3.02
SGB_614	uncultured Bacteroides sp.	0.85	0.27	0.71	0.20	0.50	0.19	1.57	0.53	1.23
SGB_421	Enterocloster bolteae	0.11	0.17	0.28	0.11	0.11	0.15	0.25	0.70	1.12
SGB_342	uncultured Ruminococcus sp.	0.20	0.01	0.07	0.00	0.19	0.02	0.68	0.05	0.21
SGB_317	Ruminococcaceae sp. CAG:18	0.04	0.01	0.04	0.00	0.01	0.01	0.17	0.05	0.12
SGB_33	Enterobacter cloacae	0.14	0.08	0.90	0.09	0.01	0.09	0.62	0.22	5.74
SGB_462	Eubacterium rectale	5.24	7.06	4.56	7.69	4.89	7.38	9.23	9.51	8.26
SGB_607	Bacteroides intestinalis	0.51	0.40	0.62	0.36	0.86	0.26	0.98	1.13	1.03
SGB_316	Ruminococcaceae sp. CAG:20	0.36	0.35	0.28	0.30	0.36	0.26	0.71	0.69	0.85
SGB_567	Butyricimonas sp. CAG:1	0.06	0.03	0.06	0.02	0.04	0.00	0.16	0.08	0.20
SGB_425	Eubacterium sp. CAG:3	0.05	0.01	0.14	0.04	0.09	0.00	0.18	0.06	0.54
SGB_275	Clostridiales sp. CAG:26	0.50	0.18	0.45	0.16	0.84	0.21	0.77	0.29	1.00
SGB_226	Oscillibacter sp. CAG:4	0.26	0.19	0.20	0.16	0.32	0.13	0.38	0.27	0.34
SGB_100	Clostridium sp. CAG:628	0.09	0.53	0.52	0.40	0.44	0.45	0.53	2.38	1.99
SGB_395	Eubacterium sp. AF15-50	0.04	0.03	0.03	0.04	0.09	0.05	0.15	0.15	0.11
SGB_378	uncultured Lachnospira sp.	0.93	0.51	0.97	0.56	0.99	0.50	1.13	0.52	1.43

			P-value, Wilcox-test		
SD_CB	SD_PC	SD_CC	PA vs CA	PB vs CB	PC vs CC
1.62	1.68	2.02	no_sig	<0.01	0.05
0.45	1.05	0.39	no_sig	<0.01	0.16
0.15	0.37	0.14	no_sig	0.01	0.78
0.25	0.57	0.33	no_sig	0.02	0.53

1.48	0.57	1.45	no_sig	0.02	0.47
1.00	0.39	0.88	no_sig	0.02	0.88
0.95	0.00	0.37	no_sig	0.02	0.02
0.55	0.44	0.46	no_sig	0.02	0.05
1.40	2.23	2.65	no_sig	0.03	0.39
0.60	0.57	0.62	no_sig	0.03	0.06
5.58	7.96	4.41	no_sig	0.04	0.24
0.26	7.86	0.16	no_sig	<0.001	<0.001
0.83	2.76	1.29	no_sig	0.03	0.33
0.33	0.73	0.42	no_sig	0.04	0.01
0.54	0.22	0.70	no_sig	0.04	0.05
0.00	0.65	0.05	no_sig	0.04	0.35
0.00	0.06	0.03	no_sig	0.04	0.84
0.29	0.08	0.28	no_sig	0.52	0.04
9.66	9.33	9.43	no_sig	0.06	0.05
0.94	2.11	0.64	no_sig	0.10	0.01
0.62	0.42	0.58	no_sig	0.71	0.01
0.06	0.10	0.00	no_sig	0.45	0.02
0.22	0.27	0.00	no_sig	0.20	0.03
0.29	2.21	0.36	no_sig	0.54	0.04
0.21	0.38	0.20	no_sig	0.81	0.04
1.71	3.00	1.96	no_sig	0.72	0.05
0.14	0.18	0.22	no_sig	0.98	0.05
0.62	1.49	0.48	no_sig	0.16	0.05

Supplementary Table 7 Distributions of species-level genome bins (SGBs) and predicted metabolic modules across different phyla

Module ID	Annotation	Total number of SGBs
GMM015	p-Cresol synthesis	291
GMM059	Secondary bile acid biosynthesis	23
GMM037	Inositol synthesis	37
GMM038	Inositol degradation	114
GMM016	p-Cresol degradation	1
GMM039	g-Hydroxybutyric acid (GHB) degradation	52
GMM017	IPA synthesis (Clostridium sporogenes pathway)	5
GMM011	Cortisol degradation	198
GMM033	Quinolinic acid degradation	441
GMM055	Propionate synthesis III	63
GMM034	Isovaleric acid synthesis I (KADH pathway)	5
GMM012	Dopamine synthesis	1
GMM056	Propionate degradation I	1
GMM057	Corrinoid dependent enzymes	599
GMM035	Isovaleric acid synthesis II (KADC pathway)	110
GMM036	S-Adenosylmethionine (SAM) synthesis	461
GMM058	Secondary bile acid biosynthesis	23
GMM052	Butyrate synthesis I	118
GMM030	Polysaccharide A	16
GMM031	17-beta-Estradiol degradation	423
GMM053	Butyrate synthesis II	2
GMM054	Propionate synthesis II	48
GMM010	Histamine degradation	5
GMM019	GABA degradation	61
GMM048	Propionate synthesis I	5
GMM026	Nitric oxide synthesis II (nitrite reductase)	2
GMM005	Tryptophan synthesis	36
GMM049	Tryptophan degradation	45
GMM027	Nitric oxide degradation I (NO dioxygenase)	31
GMM006	Glutamate synthesis I	428
GMM029	ClpB (ATP-dependent chaperone protein)	414
GMM007	Glutamate synthesis II	7
GMM022	GABA synthesis III	51
GMM044	Acetate synthesis II	2
GMM001	Serotonin synthesis I	1
GMM045	Acetate synthesis III	26.5
GMM023	Dopamine degradation	12
GMM024	DOPAC synthesis	4
GMM047	Acetate degradation	150
GMM003	Melatonin synthesis	38
GMM025	Nitric oxide synthesis I (NO synthase)	1
GMM040	Menaquinone synthesis (vitamin K2) I	81
GMM041	Menaquinone synthesis (vitamin K2) II (futalosine pathway)	6
GMM042	PUFAs synthesis (AA, EPA, DHA)	217
GMM020	GABA synthesis I	8
GMM043	Acetate synthesis I	467
GMM021	GABA synthesis II	6
GMM009	Histamine synthesis	14

Percentage (%)	Actinobacteria	Bacteroidetes	Candidatus	Cyanobacteria	Elusimicrobia
42.11	5	53	1	2	2
3.33	1	2	0	0	0
5.35	1	33	0	0	0
16.50	1	15	0	0	0
0.14	0	0	0	0	0
7.53	0	5	0	1	0
0.72	0	0	0	0	0
28.65	1	36	0	0	0
63.82	17	78	2	1	2
9.12	0	50	0	0	0
0.72	0	0	0	0	0
0.14	0	0	0	0	0
0.14	0	0	0	0	0
86.69	23	97	2	1	2
15.92	4	7	1	0	0
66.71	10	75	2	1	2
3.33	0	10	0	0	0
17.08	0	50	0	0	2
2.32	0	8	0	0	0
61.22	14	71	1	0	1
0.29	0	0	0	0	0
6.95	0	0	0	0	0
0.72	0	0	0	0	0
8.83	0	0	0	0	0
0.72	0	0	0	0	0
0.29	0	0	0	0	0
5.21	0	0	0	0	0
6.51	0	32	0	0	0
4.49	1	1	0	0	0
61.94	14	86	0	1	2
59.91	9	79	2	1	0
1.01	0	0	0	0	0
7.38	2	36	0	0	0
0.29	0	0	0	0	0
0.14	0	0	0	0	0
3.84	0	11	0	1.5	0
1.74	0	0	0	0	0
0.58	0	0	0	0	0
21.71	7	45	0	0	0
5.50	8	0	0	0	0
0.14	0	0	0	0	0
11.72	5	54	0	0	0
0.87	0	2	0	0	0
31.40	1	40	0	0	0
1.16	0	0	0	0	0
67.58	8	78	0	0	1
0.87	0	0	0	0	0
2.03	0	8	0	0	0

Euryarchaeota	Firmicutes	Fusobacteria	Lentisphaerae	Proteobacteria	Synergistetes
0	185	3	4	31	0
0	20	0	0	0	0
2	1	0	0	0	0
0	86	0	2	9	0
0	0	0	0	1	0
0	40	1	0	5	0
0	5	0	0	0	0
0	146	2	1	12	0
5	294	3	3	25	2
0	9	0	0	0	0
0	5	0	0	0	0
0	1	0	0	0	0
0	0	0	0	1	0
3	409	3	3	39	2
0	89	0	2	6	1
5	326	3	2	27	0
0	9	2	0	1	1
0	65	1	0	0	0
0	7	0	0	0	0
0	287	2	1	31	0
0	2	0	0	0	0
0	40	2	1	4	1
0	0	0	0	5	0
0	49	0	1	11	0
0	1	0	0	4	0
0	0	0	0	2	0
0	28	0	0	8	0
0	6	0	0	5	0
0	12	1	0	16	0
5	286	2	4	20	1
0	267	3	4	35	0
0	7	0	0	0	0
0	8	0	0	3	0
0	2	0	0	0	0
0	1	0	0	0	0
0	13	1	0	0	0
0	4	0	0	8	0
0	2	0	0	2	0
6	86	0	2	4	0
0	15	0	0	15	0
0	1	0	0	0	0
0	9	0	0	13	0
0	0	0	0	1	0
0	142	1	4	28	1
0	0	0	0	8	0
0	340	2	3	22	1
0	0	0	0	6	0
0	1	1	0	1	0

Tenericutes	Verrucomicrobia
0	5
0	0
0	0
0	1
0	0
0	0
0	0
0	0
2	7
0	4
0	0
0	0
0	0
6	9
0	0
4	4
0	0
0	0
0	1
6	9
0	0
0	0
0	0
0	0
0	0
0	0
0	0
0	2
0	0
1	6
6	8
0	0
0	2
0	0
0	0
0	0
0	0
0	0
0	0
0	0
0	0
0	0
0	3
0	0
0	0
6	6
0	0
0	3

Supplementary Table 8 Significant differential serum metabolites between the probiotic and placebo groups Remarks:P, Probio-M8 group; C, placebo group; A, 0d; B, 1M; C, 3M

Differential metabolites	Between the probiotic and placebo groups											
	Mean_PA	Mean_CA	Mean_PB	Mean_CB	Mean_PC	Mean_CC	SD_PA	SD_CA	SD_PB	SD_CB	SD_PC	SD_CC
stearoyl ethanolamide	3.84E-05	3.51E-05	3.63E-05	3.28E-05	3.60E-05	3.62E-05	1.04E-05	9.58E-06	8.11E-06	6.47E-06	1.01E-05	8.87E-06
chenodeoxycholate	3.85E-04	4.08E-04	4.20E-04	3.95E-04	3.18E-04	4.21E-04	2.03E-04	2.28E-04	1.75E-04	2.45E-04	2.01E-04	1.92E-04
trimethyllysine	3.30E-04	3.50E-04	3.28E-04	3.62E-04	3.20E-04	3.71E-04	1.06E-04	7.77E-05	8.68E-05	9.84E-05	8.37E-05	9.72E-05
bilirubin	3.51E-04	3.49E-04	3.56E-04	3.46E-04	3.33E-04	3.49E-04	3.50E-05	3.92E-05	2.93E-05	3.32E-05	3.13E-05	3.79E-05
erythronic acid	1.14E-04	1.21E-04	1.18E-04	1.16E-04	1.05E-04	1.19E-04	2.10E-05	2.65E-05	2.07E-05	2.37E-05	2.39E-05	2.68E-05
creatine	5.86E-04	6.69E-04	6.77E-04	6.67E-04	4.77E-04	7.03E-04	2.95E-04	2.83E-04	3.59E-04	3.36E-04	2.37E-04	4.18E-04
ADMA	1.35E-04	1.54E-04	1.55E-04	1.56E-04	1.36E-04	1.57E-04	3.93E-05	4.73E-05	4.01E-05	4.99E-05	4.41E-05	5.06E-05
cholate	2.34E-03	2.45E-03	2.55E-03	2.47E-03	1.99E-03	2.56E-03	8.17E-04	8.72E-04	8.16E-04	9.95E-04	8.12E-04	8.30E-04
C20:4 carnitine	1.14E-04	1.16E-04	1.12E-04	1.17E-04	1.27E-04	1.12E-04	2.11E-05	2.94E-05	2.21E-05	3.22E-05	2.47E-05	2.77E-05
cholestenone	1.02E-03	9.96E-04	9.07E-04	9.85E-04	1.11E-03	9.75E-04	3.08E-04	3.54E-04	2.47E-04	3.47E-04	2.98E-04	3.60E-04
N-acetylglutamic acid	5.96E-05	6.11E-05	5.86E-05	6.19E-05	6.47E-05	5.84E-05	1.36E-05	1.55E-05	1.30E-05	1.56E-05	1.30E-05	1.39E-05
urobilin	2.72E-04	2.75E-04	2.47E-04	2.89E-04	3.40E-04	2.67E-04	1.38E-04	1.67E-04	1.42E-04	1.78E-04	1.47E-04	1.60E-04

Differential metabolites	Wilcoxon.test		
	PA vs CA	PB vs CB	PCvs CC
stearoyl ethanolamide	no sig	0.05	0.70
chenodeoxycholate	no sig	0.24	0.02
trimethyllysine	no sig	0.14	0.03
bilirubin	no sig	0.14	0.03
erythronic acid	no sig	0.98	0.03
creatine	no sig	0.88	0.03
ADMA	no sig	0.94	0.05
cholate	no sig	0.52	0.01
C20:4 carnitine	no sig	0.51	0.02
cholestenone	no sig	0.45	0.04
N-acetylglutamic acid	no sig	0.27	0.05
urobilin	no sig	0.35	0.05

Supplementary Table 9 Four differential serum metabolites identified by LC-MS Remarks:P, Probio-M8 group; C, placebo group; A, 0d; B, 1M; C, 3M

**Serum Metabolites**	**Intensity**											
Metabolites	Mean_PA	Mean_CA	Mean_PB	Mean_CB	Mean_PC	Mean_CC	SD_PA	SD_CA	SD_PB	SD_CB	SD_PC	SD_CC
Tryptophan	9036.25	9793.08	9381.63	10399.70	8954.96	11240.35	2793.15	3383.19	2840.07	3285.38	2477.05	3221.58
Glutamine	1695.42	1808.30	1502.99	1795.52	1670.76	1753.01	594.41	572.13	429.70	477.83	582.71	546.43
3_Hydroxytyramine	4.35	3.56	6.10	4.09	5.93	6.10	3.25	2.45	3.15	2.10	5.86	2.46
Acetic_acid	1.46	0.95	1.41	0.44	1.38	0.55	0.65	0.82	0.58	0.22	0.55	0.31

**Serum Metabolites**	**Wilcoxon.test**		
Metabolites	PA vs CA	PB vs CB	PCvs CC
Tryptophan	no sig	0.27	0.04
Glutamine	no sig	0.05	0.43
3_Hydroxytyramine	no sig	0.04	0.17
Acetic_acid	no sig	<0.001	<0.001
